# Visible-light-induced oxidant and metal-free dehydrogenative cascade trifluoromethylation and oxidation of 1,6-enynes with water†
†Electronic supplementary information (ESI) available: Experimental details, spectroscopic data, ^1^H, ^13^C, ^19^F, mass spectra, DFT calculations and X-ray crystallographic data. CCDC 152606 0 (**2c**), 1526059 (**4k**), 1526057 (**6c**), and 1526058 (**6d**). For ESI and crystallographic data in CIF or other electronic format see DOI: 10.1039/c7sc02556d



**DOI:** 10.1039/c7sc02556d

**Published:** 2017-07-10

**Authors:** Sadhan Jana, Ajay Verma, Rahul Kadu, Sangit Kumar

**Affiliations:** a Department of Chemistry , Indian Institute of Science Education and Research (IISER) Bhopal , Bhopal By-pass Road, Bhauri , Bhopal-462066 , India . Email: sangitkumar@iiserb.ac.in ; http://home.iiserbhopal.ac.in/∼sangitkumar/

## Abstract

Generally, oxy-trifluoromethylation in olefins is achieved using oxidants and transition metal catalysts. However, labile olefins remain unexplored due to their incompatibility with harsh reaction conditions. Here, unprecedented light-induced oxidant and metal-free tandem radical cyclization–trifluoromethylation and dehydrogenative oxygenation of 1,6-enynes have been achieved using a photoredox catalyst, CF_3_SO_2_Na, and phenanthrene-9,10-dione (**PQ**), Langlois’ reagent (CF_3_SO_2_Na) and water as the oxygen source. This benign protocol allows for access to various CF_3_-containing C_3_-aryloyl/acylated benzofurans, benzothiophenes, and indoles. Moreover, the oxidized undesired products, which are inherently formed by the cleavage of the vinylic carbon and heteroatom bond, have been circumvented under oxidant free conditions. The mechanistic investigations by UV-visible and ESR spectroscopy, electrochemical studies, isotope labelling and density functional theory (DFT) suggest that light induced **PQ** produced a CF_3_ radical from CF_3_SO_2_Na. The generated CF_3_ radical adds to the alkene, followed by cyclization, to provide a vinylic radical that transfers an electron to **PQ** and generates a vinylic cation. Alternatively, electron transfer may occur from the CF_3_-added alkene moiety, forming a carbocation, which would undergo cationic cyclization to generate a vinylic carbocation. The subsequent addition of water to the vinylic cation, followed by the elimination of hydrogen gas, led to the formation of trifluoromethylated C_3_-aryloyl/acylated heterocycles.

## Abstract 2

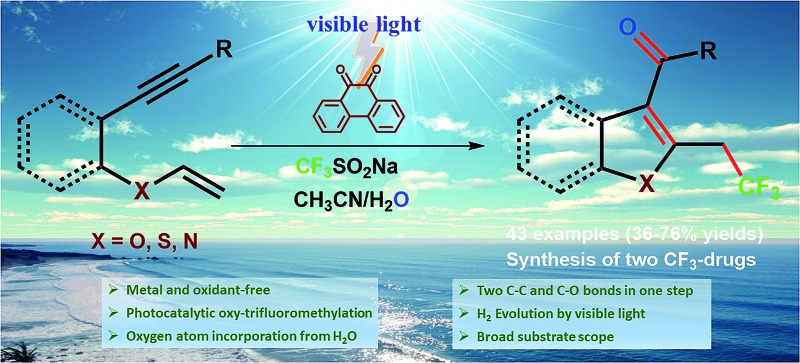
Unprecedented light-induced oxidant and metal-free tandem radical cyclization–trifluoromethylation and dehydrogenative oxygenation of 1,6-enynes have been achieved using a photoredox catalyst, CF_3_SO_2_Na, and water as the oxygen source.

## Introduction

**Fig. 1:**
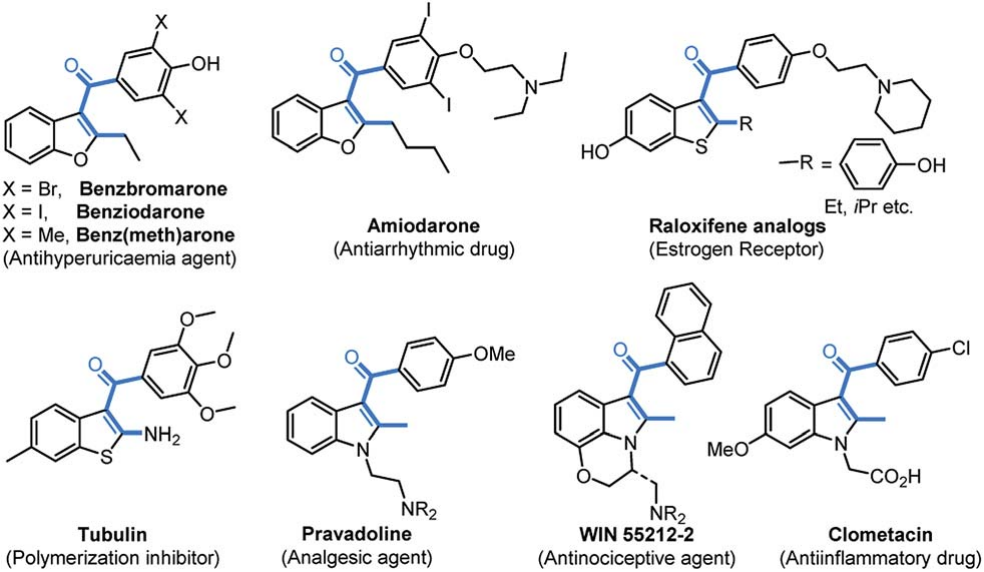
C_3_-Aryloylated heterocyclic drugs.

C_3_-Aryloylated benzofurans, benzothiophenes, indoles, and related fused polyheterocycles are privileged structures with numerous applications in materials, drugs, and biology (Fig. 1).^1–5^ Benzbromarone^2^ is being used for the treatment of gout as a urate lowering drug (ULD) and amiodarone^3^ is a potent inhibitor of the human cytochrome P-450 (CYP)_2_C_19_ responsible for metabolizing commonly prescribed drugs. The C_3_-aryloylated benzothiophenes,^4^ raloxifene and tubulin, which are antimitotic agents, act as an estrogen receptor and inhibitor for the polymerization of tubulin and growth of tumour cells, respectively. Similarly, 2-methyl-C_3_-aryloylated indoles,^5^ namely pravadoline, WIN55212-2, and clometacin, are nonsteroidal anti-inflammatory drugs, which are used against pain and inflammation. However, the construction of C_3_-aryloylated benzofuran, indole, and thiophene scaffolds is still challenging (*vide infra*) despite the availability of advanced synthetic strategies.^15–17^

The incorporation of a trifluoromethyl group (–CF_3_) into drug molecules can dramatically change their properties, such as their solubility, lipophilicity, metabolic stability, *etc.* ^6^ As a result, several trifluoromethylated heterocycles, such as efavirenz and celecoxib, are used as potential drugs for the treatment of various diseases.^7^ It is worth noting that the synthesis of trifluoromethylated C_3_-aryloyl heterocycles (shown in Fig. 1) has not been reported to date.

**Scheme 1:**
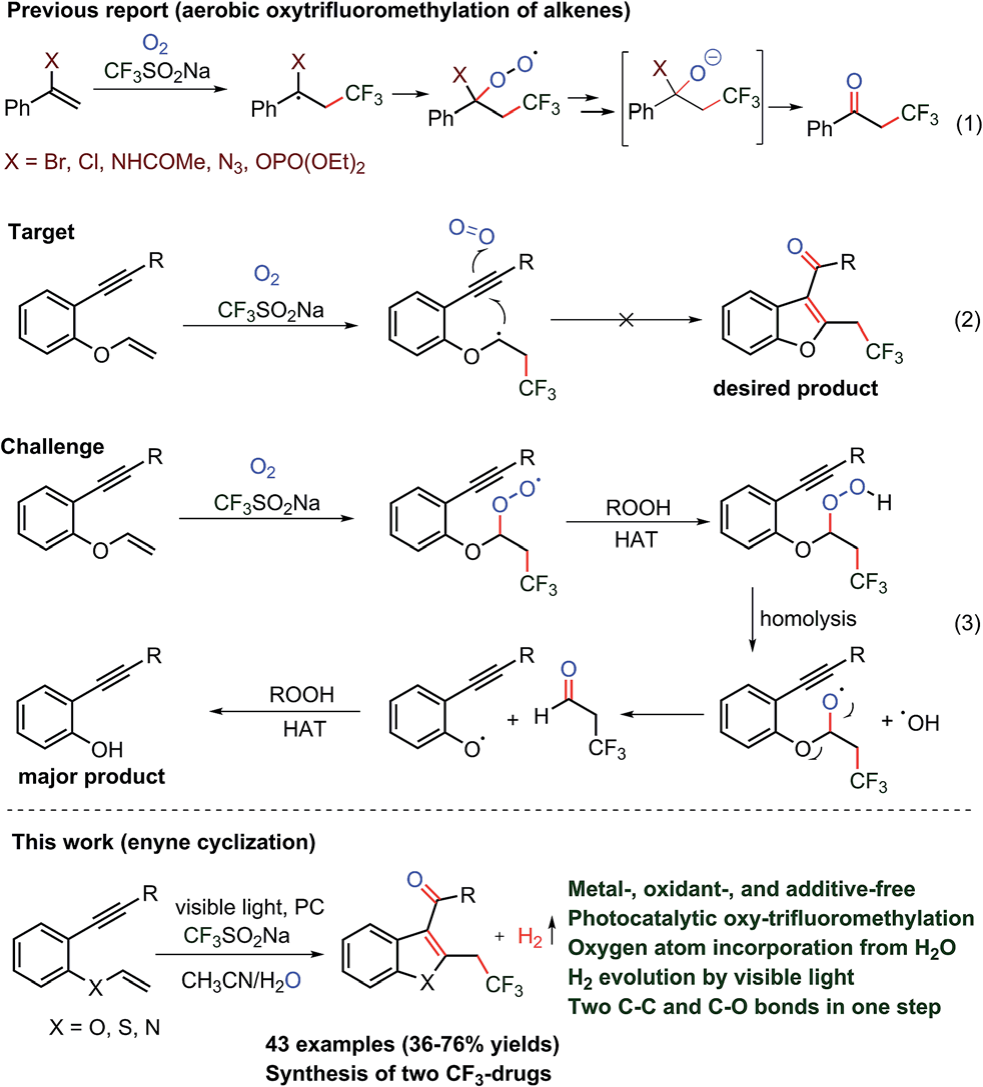
Oxy-trifluoromethylation of alkenes.

The trifluoromethylation and oxygenation of alkene and alkyne substrates have been achieved using various trifluoromethylating reagents and peroxides, persulfates, hypervalent iodine salts and oxygen as the oxidant and/or transition metal catalytic system (Scheme 1, eqn (1)).^8–14^ Although different valuable approaches appear to have been incorporated into the –CF_3_ group, still there is demand for mild and sustainable synthetic methods that avoid oxidants and heavy metals and are applicable to sensitive substrates for the construction of functionalized advanced heterocycles.

The cyclization of 1,6-enynes can be a powerful transformation that allows for the construction of C_3_-aryloyl/acylated heterocycles (Scheme 1, eqn (2)).^15–17^ 1,6-Enyne substrates under transition metal (TM)-catalyzed reaction conditions are rearranged to benzofuran,^15^ benzothiophene^16^ and indole^17^ heterocycles, and thus restrict the use of TM to access the desired C_3_-aryloylated heterocycles. On the other hand, the use of oxidants such as hypervalent iodine reagents, peroxides, and oxygen, which are required for the generation of the CF_3_ radical and as a source of oxygen under TM-free conditions, led to the cleavage of the vinylic carbon–heteroatom (oxygen, sulfur, nitrogen, *etc.*)^8*c*^ bond and thus provided undesired phenol, thiophenol and aniline as the main side products, respectively (eqn (3)). It is a natural inherent property of oxygen (bi-radical) to form a peroxide that leads to the cleavage of the labile carbon–heteroatom bond and thus imposes a major challenge to functionalize such a vinylic carbon–carbon double bond.^8*f*^ Consequently, keeping the ether linkage intact throughout the trifluoromethylation of the vinylic C–C double bond adjacent to the heteroatom has not been reported to date, despite the presence of this skeleton in various molecules of pharmaceuticals, agrochemicals, materials, and fine chemicals.

Photocatalyzed cascade radical reactions have gained much attention in recent times as these reactions provide access to complex molecules in one pot with step and atom economy.^18,19^ Although several photocatalyzed trifluoromethylation reactions *viz.* aromatic trifluoromethylation,^20*a*^ conversion of alkynes into tetra-substituted trifluoromethylated alkenes,^20*b*^ and radical trifluoromethylation/cyclization cascade for CF_3_-containing pyrazolines and isoxazolines^20*c*^ have been described, cascade oxy-trifluoromethylation has not been explored. Water is the most abundant reactant that can be used as an oxygen source in photocatalytic reactions. The incorporation of water for the oxygenation of substrates requires a strong one-electron oxidant. Alternatively, water can be added as a nucleophile to an organic substrate followed by its oxidation by weaker oxidants under light driven conditions, which could lead to the oxygenation of organic molecules.^21^

In the continuation of our research interest in TM-free C–C and C–heteroatom coupling reactions,^22^ here, we disclose a photocatalyzed reaction that not only activates CF_3_SO_2_Na for the generation of the CF_3_ radical but also facilitates water towards the oxygenation of organic molecules. This approach enables the oxygenation of labile 1,6-enyne substrates along with the generation of hydrogen gas under oxidant-free conditions, which circumvents an undesired, over-oxidized product. The mechanistic investigations by labelling experiments, UV-visible and ESR spectroscopy and cyclic voltammetry studies, corroborated by DFT calculations, have been carried out to understand the role of the photoredox catalyst (PC) in the oxy-trifluoromethylation reaction of 1,6-enynes under oxidant and metal-free conditions.

## Results and discussion

### Initial screening and photo-initiated conditions with and without oxidants

Initially, 1-(phenylethynyl)-2-(vinyloxy)benzene substrate **1a** was reacted with an economical amount of trifluoromethylating Langlois’ reagent (CF_3_SO_2_Na) under an oxygen atmosphere and heated conditions (Table 1). Disappointingly, cleavage of the vinylic carbon–oxygen bond occurred, which yielded undesired 2-(phenylethynyl)phenol **2a′** instead of C_3_-aryloylated-2-trifluoromethylated benzofuran **2a** (Table 1, entry 1). Similarly, various oxidants, such as hydrogen peroxide, benzyl peroxide, *tert*-butyl hydroperoxide, di-*tert* butyl peroxide, iodine, (diacetoxyiodo)benzene, persulfate salts, oxone, ceric ammonium nitrate, 2,3-dichloro-5,6-dicyano-*p*-benzoquinone and the radical initiators azobisisobutyronitrile and azobis(2,4-dimethylvaleronitrile), provided the undesired phenol **2a′** as a major product and **2a** was noticed as a minor product (<20% yields, see ESI, page S3 and S4†). Next, we performed the reaction at room temperature using oxygen, and the reaction provided **2a** and **2a′** in <10 and 15% yields, respectively (Table 1, entry 2). However, poor conversion was observed despite the continuation of the reaction for a longer time (24 h) and 70% of substrate **1a** was recovered from the reaction.

**Table 1:** Optimization of the reaction conditions^*a*^

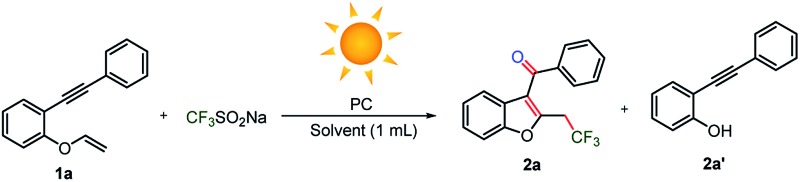				
Entry	PC (mol%)	Gas	Solvent	Yield^*b*^ **2a**/**2a′**
1	–(90 °C)	O_2_	DMSO	Trace/74
2	–(25 °C)	O_2_	DMSO	<10/15
3	—	O_2_	Acetone	Trace/20
4	**A**	O_2_	Acetone	Trace/15
5	**B**	O_2_	Acetone	nd/10
6	**C**	O_2_	Acetone	5/15
7	**D**	O_2_	Acetone	5/15
8	**PQD**	O_2_	Acetone	3/25
9	**PQ**	O_2_	Acetone	9/30
10	**PQ**	Ar	Acetone	Trace/—
11	**PQ**	Ar	Acetone : H_2_O (9 : 1)	20/8
12	**PQ**	Ar	EtOH : H_2_O (9 : 1)	13/5
13	**PQ**	Ar	DMF : H_2_O (9 : 1)	35/10
14	**PQ**	Ar	DMSO : H_2_O (9 : 1)	48/12
**15**	**PQ**	**Ar**	**CH**_**3**_ **CN : H**_**2**_ **O (9 : 1)**	**76/trace**
16	**PQ**	Ar	THF : H_2_O (9 : 1)	32/5
17^*c*^	**PQ**	Ar	CH_3_CN : H_2_O (9 : 1)	69/trace
18	**PQ**	O_2_	CH_3_CN : H_2_O (9 : 1)	24/40

^*a*^The reactions were carried out using 0.1 mmol of **1a**, 0.3 mmol of CF_3_SO_2_Na and 0.01 mmol of photocatalyst (PC) in 1 mL of solvent at 30 °C in a 5 mL round bottom flask under O_2_ or Ar atmosphere, unless otherwise stated; the progress of the reaction was monitored by TLC.

^*b*^Percentage isolated yield.

^*c*^Under a household CFL bulb (23 W). nd = not detected.

**Fig. 2:**
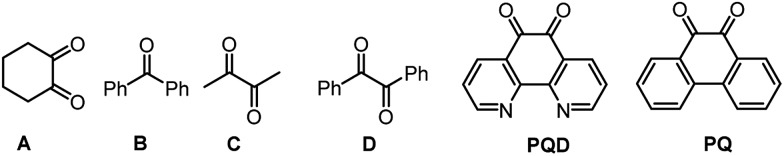
Screened diketones and *ortho*-quinones.

Next, we envisaged that the UV-visible irradiation of the reaction system in the presence of diketones would bring about oxy-trifluoromethylation in 1,6-enyne as n → π* triplet-excited ketones have been successfully exploited in several photoinduced chemical transformations.^23^ Consequently, α-diketones (**A–D**) and *ortho*-quinones 1,10-phenanthroline-5,6-dione (**PQD**) and phenanthrene-9,10-dione (**PQ**) were investigated for their UV-visible absorption properties (Fig. 2). **A–D** show strong absorption in the ultraviolet range below 380 nm, whereas *ortho*-quinones **PQD** and **PQ** exhibit absorption bands in the visible range (380–420 nm). The absorption bands for **PQ** appeared in the visible light region (420 and 505 nm)^24^ in acetone solvent, which shows a slight red shift in acetonitrile (ESI, page S12 and S13†). The electronic excitation would lead to a first triplet excited state (T1) of the carbonyl group having a diradicaloid nature. The triplet states of aromatic ketones are long-lived, and also the electronic character and reactivity of the lowest triplet state can be tuned by the solvent polarity.^25^ 9,10-Phenanthrenequinone **PQ** shows an n → π* transition of an aromatic ketone that could reverse the charges on the C=O group, thus making the oxygen atom electron deficient. Due to the electron-deficient oxygen atom and “radical-like” characteristic of carbonyl in **PQ**, its reduction is enabled^26^ by the abstraction of an electron from CF_3_SO_2_Na and the generation of a CF_3_ radical, which can indeed initiate a cascade reaction.

The reaction system under UV-irradiation in acetone, which acts as a solvent and radical initiator,^20*n*^ and under oxygen atmosphere provided only traces of **2a** (Table 1, entry 3). Similar results were obtained with **A–C** under UV-irradiation and also with **D**, **PQD** and **PQ** under visible light and oxygen atmosphere (Table 1, entries 4–9). In the absence of oxygen, no product formation was realized, as expected (Table 1, entry 10). Surprisingly, when the reaction was performed in the absence of oxygen in a mixture of an organic and water solvent system, a noticeable increase in the yield of the desired product **2a** was observed, moreover, the formation of undesired side product was minimized to <10% yield (Table 1, entries 11–16). The reactions presented here were optimized under sun-light irradiation. In a separate experiment, a household CFL bulb (23 W) was used, which provided nearly the same yield of **2a** (69% *vs.* 76% in sunlight) although a longer time (6 h for sunlight *vs.* 16 h for one 23 W CFL bulb) is required (Table 1, entry 17). A trifluoromethylation reaction under oxygen atmosphere, instead of argon, under optimized conditions, afforded 24% yield of the desired product **2a** (Table 1, entry 18). Among the various diketones screened (**A–D**, **PQD**, **PQ**) (Table 1, entries 3–17), **PQ**, which has been explored for the first time,^27^ provided optimum yield of the desired oxy-trifluoromethylated benzofuran **2a** under sunlight irradiation.

### The substrate scope of the light-induced oxy-trifluoromethylation reaction to phenolic 1,6-enynes

**Scheme 2:**
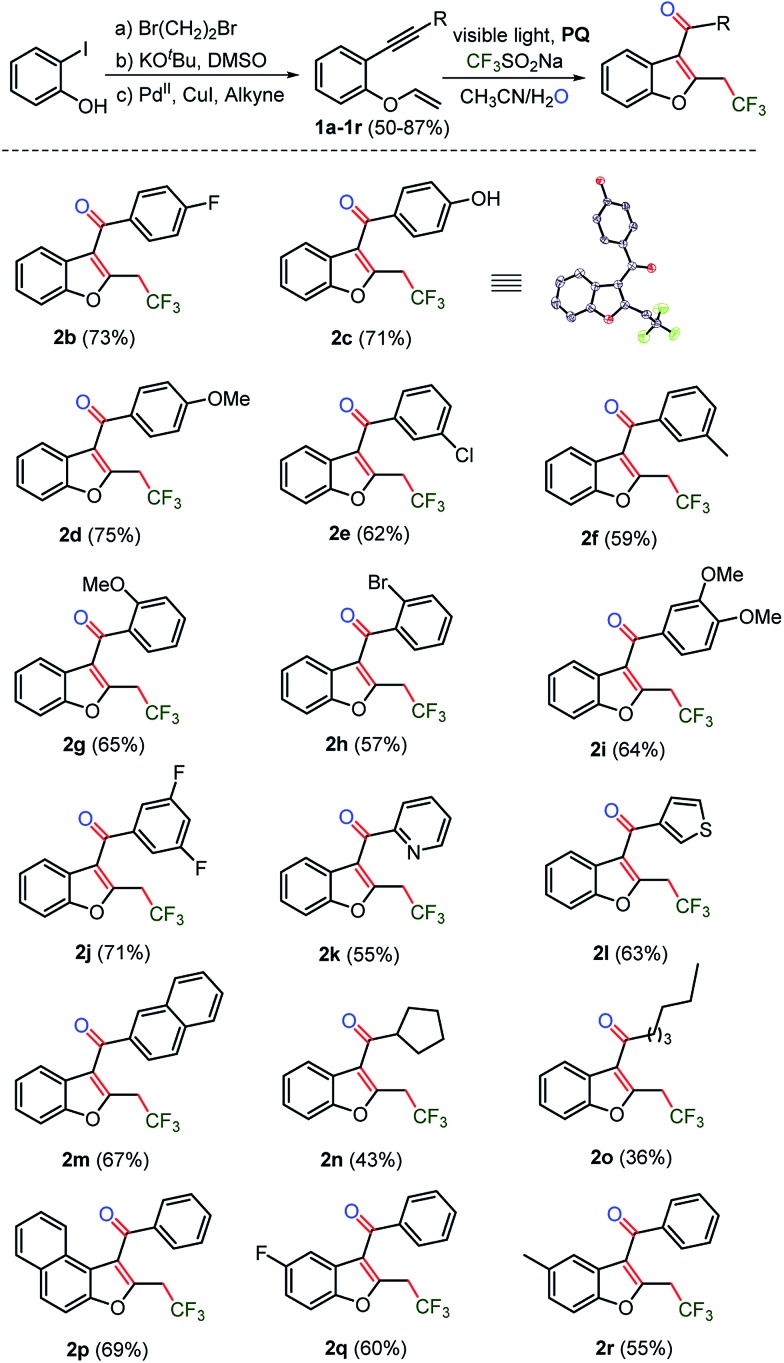
CF_3_-Benzo[*b*]furans: the scope with regards to the ethynyl and vinyloxy rings; the reactions were carried out at 0.1 mmol of **1a** using 0.3 mmol of CF_3_SO_2_Na and 0.01 mmol of photocatalyst **PQ** in CH_3_CN + H_2_O (0.9 + 0.1 mL) under Ar, and the progress of the reaction was monitored by TLC for 4–8 h.

Next, the substrate scope was studied on phenolic 1,6-enynes (**1a–1r**)^28^ under the optimized reaction conditions (Table 1, entry 15). Substrates **1b–1j**, with electron donating as well as electron withdrawing substituents on the ethynyl ring, showed compatibility with the optimized reaction conditions and afforded respective trifluoromethylated benzofurans **2b–2j** in 57–75% yields (Scheme 2). Moreover, the phenolic-1,6-enyene substrate **1c** with OH functionality and an acidic proton was tolerated to provide hydroxyl benzofuran **2c** in good yield. The heteroaromatics pyridyl and thiophenyl and other aromatic naphthyl, as the ethynyl ring containing substrates **1k–1m**, were also amenable to the reaction and transformed into the respective benzofurans **2k–2m**. Next, the *n*- and *sec*-alkyl substituted substrates **1n–1o** underwent an oxy-trifluoromethylation reaction to afford the acyl-benzofurans **2n–2o**, albeit in low yields. Substitution on the phenolic ring was also explored under the optimized conditions. Substrate **1p**, containing a naphthyl ring, and substrates **1q–1r**, having fluoro and methyl substituents on the phenolic ring, afforded respective trifluoromethylated benzofurans **2p** and **2q–2r** without any noticeable loss in the yields (55–69%).

### Substrate scope with regard to thiophenolic 1,6-enynes

**Scheme 3:**
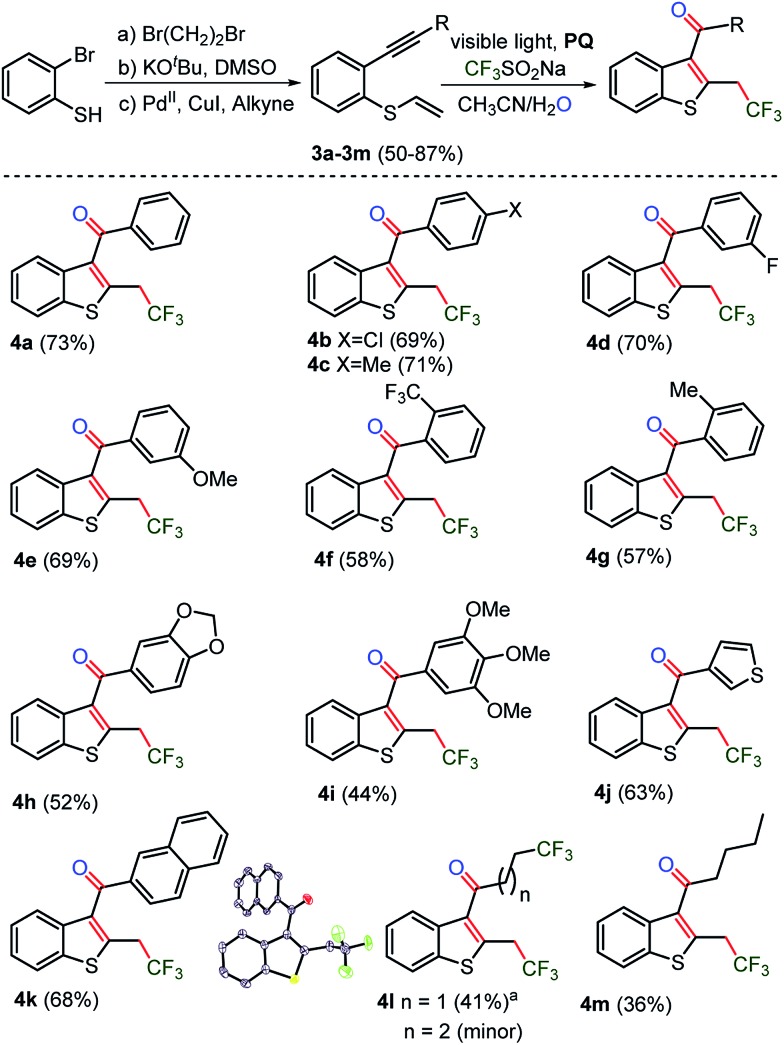
Synthesis of CF_3_-benzo[*b*]thiophenes. The reaction of 0.1 mmol of **3a**, 0.3 mmol of CF_3_SO_2_Na and 0.01 mmol of photocatalyst **PQ** in CH_3_CN and H_2_O was irradiated under Ar and the progress of the reaction was monitored by TLC. **4l** was obtained from cyclopropane substituted substrate **3l**, as the major along with the minor (*n* = 1) product (see the ESI, page S56 and S57, and the spectra on pages S203–S207†).

In order to explore the synthesis of trifluoromethylated C_3_-aryloyl benzothiophenes, thio-linked 1,6-enyne substrates **3a–3m** were prepared from 2-bromo-benzenethiols in moderate yields (Scheme 3, for details see the ESI, page S28 and S29†). In general, the sulfur-containing substrates showed sluggish reactivity under TM-catalyzed reaction conditions due to the poisoning of the catalyst by sulfur. Earlier synthetic methods involve inter or intramolecular coupling of alkynyl and sulfoxide in the presence of high loading of the Au, Hg and Pd-catalysts.^16d,e^ To our delight, a TM-free cyclization reaction yielded C_3_-aryloyl trifluoromethylated benzothiophenes **4a–4m** in nearly the same yields (73–36%) as those obtained for benzofurans. Moreover, a similar substrate scope was realized as thio-linked 1,6-enyne substrates **3b–3m** with electron donating methyl, methoxy, [1,3]-dioxole, and tri- methoxy and electron withdrawing CF_3_ and F and also naphthyl, thiophenyl, and *n*-butyl substituents provided unaltered yields of 2-trifluoromethyl C_3_-aryloyl/acylated benzothiophenes **4b–4m**. The cyclopropyl substituted substrate **3l** also underwent ring opening of the cyclopropyl ring by the trifluoromethyl radical to yield an unexpected di-trifluoromethylated benzothiophene **4l** as the major product and a di-trifluoromethylated product as the minor product.

### Substrate scope with regard to anilinic 1,6-enynes

**Scheme 4:**
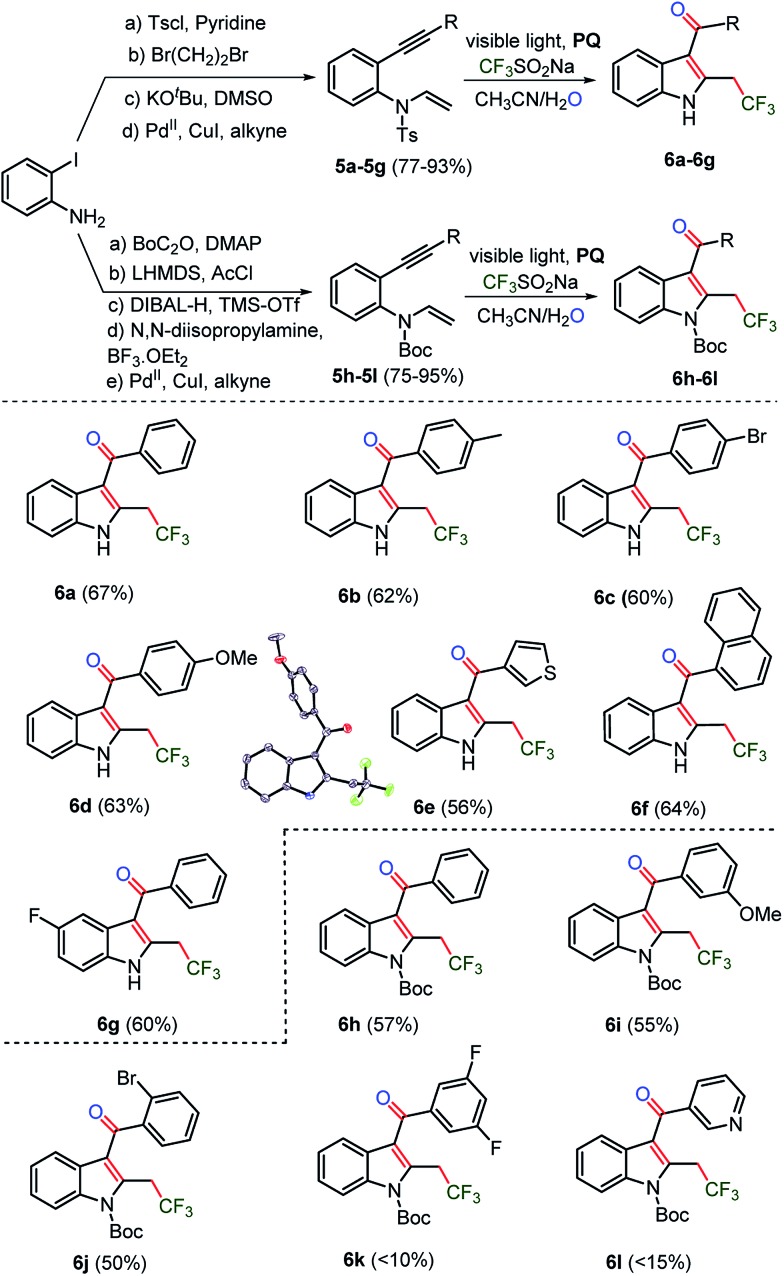
Synthesis of CF_3_-indoles. The reaction of **5a** (0.1 mmol), 0.3 mmol of CF_3_SO_2_Na and 0.01 mmol of photocatalyst **PQ** in CH_3_CN and H_2_O was irradiated for 4–8 h under argon atmosphere.

Next, *N*-tosyl (Ts) and *N-tert*-butyloxycarbonyl (Boc) protected 1,6-enyne^29^ substrates were prepared to construct trifluoromethylated C_3_-aryloyl indoles, which are prevalent heterocycles in many drugs and materials (Scheme 4).^5a,b^ Indeed, *N*-tosylated substrates **5a–5g** underwent an oxy-trifluoromethylation reaction. Moreover, the removal of the tosyl (Ts) group was realized in the same pot leading to *N*-unprotected trifluoromethylated C_3_-aryloyl indoles **6a–6g** in 67–56% yields. Next, we sought for the synthesis of *N*-protected trifluoromethylated C_3_-aryloyl indoles. Substrates **5h–5l**, which were protected by a *N-tert*-butyloxycarbonyl (Boc) group, provided trifluoromethylated indoles in moderate (10–57%) yields. Both *N*-Ts and Boc-protected 1,6-enyne substrates, having bromo, fluoro, difluoro, methoxy, methyl and pyridyl, naphthyl and thiophenyl rings, were amenable to the oxidant and metal-free oxy-trifluoromethylation reactions. A more efficient electron delocalization occurs with the *N*-protected carbonyl group of carbamate rather than the sufonyl moiety of the tosyl group. The weak mesomeric effect indicates that the sulfur-centered group had increased electron density on the nitrogen, which reflected the higher aromaticity of the indole.^30^ As a consequence, tosyl becomes a better leaving group than carbamate. Thus the deprotection of the tosyl protecting group under the optimized reaction conditions is attributed to its labile nature. The C_3_-aryloyl trifluoromethylated indoles **6c** and **6d**, benzofuran **2c**, and benzothiophene **4k** are also characterized by X-ray single crystal structure analysis (for details, see ESI, pages S80–S106†).

### Synthesis of CF_3_-bearing drugs

**Scheme 5:**
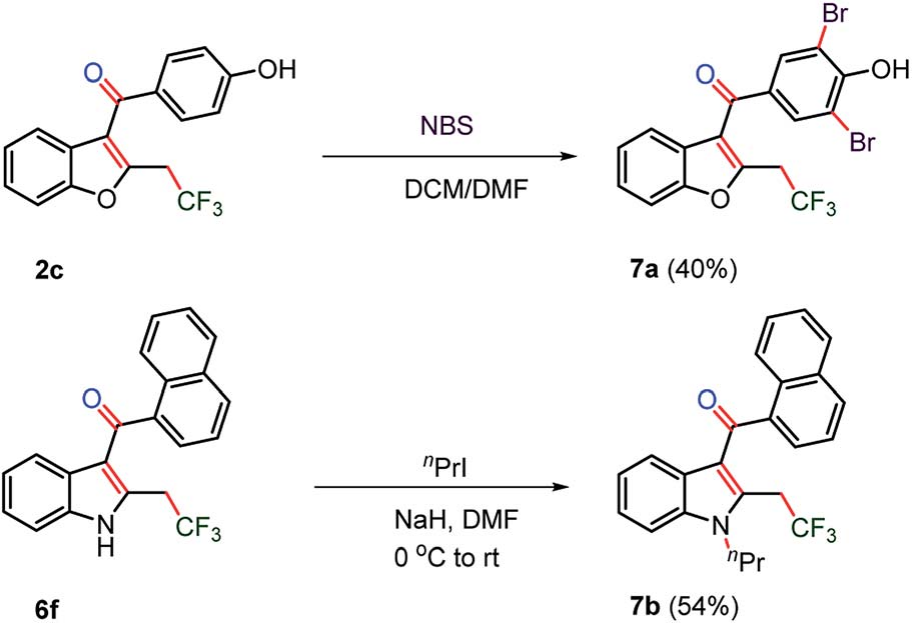
CF_3_-bearing drugs by post-modification. NBS (0.40 mmol) and **2c** (0.20 mmol) were used for **7a**. ^*n*^PrI (0.12 mmol) and **6f** (0.16 mmol) in DMF were used for **7b**.

The synthesis of trifluoromethylated drug molecules was explored from synthesized 2-trifluoromethyl-C_3_-aryloyl benzofuran **2c** and indole **6f** by late-stage functionalization (Scheme 5). The bromination of benzofuran **2c** using *N*-bromosuccinimide afforded novel trifluoromethylated benzbromarone **7a** in 40% yield. The *N*-alkylation of the synthesized oxy-trifluoromethylated indole **6f** was observed to be difficult by known methods using KOH in DMSO, Cs_2_CO_3_ in DMSO or K_2_CO_3_ in DMF and failed to yield *N*-alkylated indole **7b** and instead a decomposed product was realized.^31^ The addition of NaH in DMF along with *n*-propyl iodide at 0 °C provided the trifluoromethylated JWH-105 **7b** drug in moderate yield (54%).

## Mechanistic study

### Labelling and ^1^H NMR experiments

**Scheme 6:**
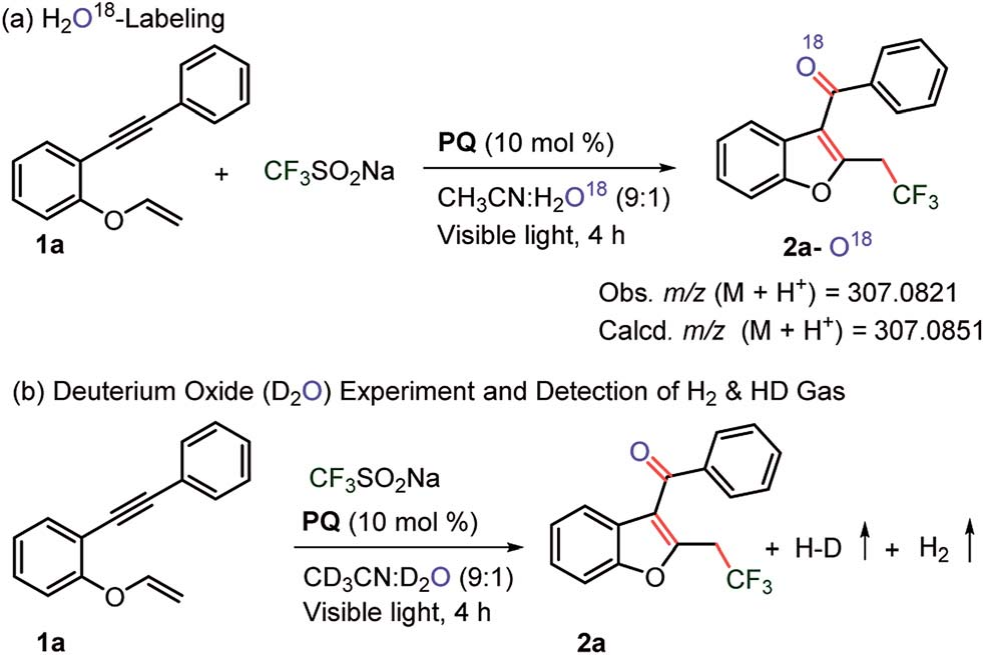
Labelling experiments. (a) 0.1 mmol of **1a**, 0.3 mmol of CF_3_SO_2_Na and 0.01 mmol of **PQ** in CH_3_CN + H_2_^18^O (900 + 100 μL). (b) The reaction was performed in an NMR tube at 0.05 mmol of **1a** using 0.15 mmol of CF_3_SO_2_Na and 0.005 mmol of **PQ** in CH_3_CN + D_2_O (450 + 50 μL).

A labelling experiment was performed using H_2_O^18^ under the optimized reaction conditions (Scheme 6). Mass analysis revealed the formation of O^18^-labelled trifluoromethylated C_3_-aryloyl benzofuran **2a** (M + H^+^ = 307.0821; calcd 307.0851).

**Fig. 3:**
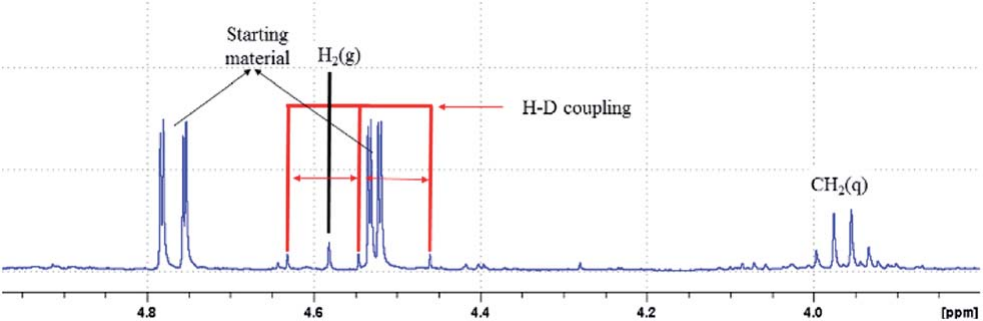
^1^H NMR spectrum of the reaction showing H_2_ & HD evolution.

^1^H NMR experiments were performed on the reaction mixture to gain further insights. ^1^H NMR spectroscopy of the reaction mixture shows a peak (*δ* = 4.57 ppm) indicative of hydrogen evolution in the reaction mixture (see the ESI, page S7–S8 for details†).^32^ Next, a reaction was carried out in CH_3_CN-*d*_3_ to study the role of the solvent. Expectedly, ^1^H NMR spectroscopy shows the formation of H_2_, which suggests that acetonitrile does not participate in the reaction. When D_2_O was used in the reaction, the formation of H–D (*δ* = 4.55 ppm, *J* = 42.8 Hz) and H_2_ was realized in the ^1^H NMR spectrum, revealing the involvement of water in the hydrogen gas evolution (Fig. 3 and Scheme 6). The formation of H_2_ gas is attributed to H_2_O in deuterated solvents and it is the result of hydrogen and deuterium exchange (page S8†).

### ESR investigation

**Scheme 7:**
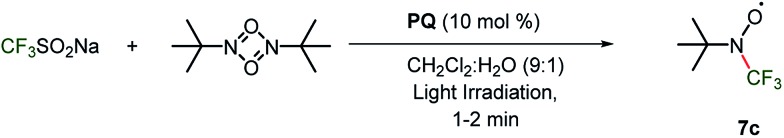
The reaction with a radical trapping reagent. The reaction was carried out using CF_3_SO_2_Na (0.3 mmol), **1a** (0.1 mmol) and MNP (0.2 mmol) in an EPR tube in CH_2_Cl_2_/H_2_O.

To investigate the reaction pathways, EPR experiments were conducted on the reaction mixture. For this purpose, the generated reactive CF_3_ radical in the reaction was trapped by the 2-methyl-2-nitrosopropane (MNP) dimer, and its EPR spectrum was monitored (Scheme 7).

**Fig. 4:**
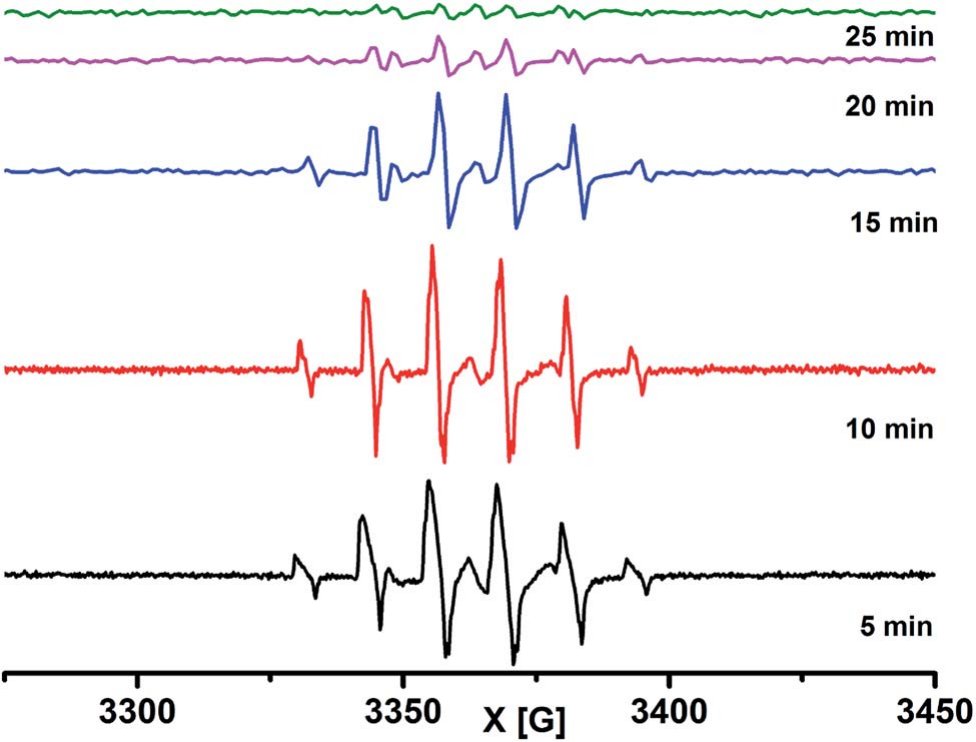
EPR spectra of the reaction mixture with MNP at different time intervals.

CF_3_SO_2_Na and MNP in the presence of CH_3_CN/H_2_O under light irradiation provided a triplet centred at 3364.5 G with a coupling constant 14.7 G, which is attributed to the dissociation of MNP to a *tert*-butyl nitroxide radical (see the ESI, Fig. S6†). The formation of the *tert*-butyl nitroxide radical is largely suppressed in CH_2_Cl_2_/H_2_O and the formation of the *tert*-butyl-trifluoromethyl nitroxide radical **7c** (Scheme 7) is observed predominantly as the EPR spectrum shows a sextet centered at *g* = 2.0054 with a coupling constant 12.27 G.^33^ A reaction mixture of MNP, CF_3_SO_2_Na and **1a** under dark conditions was realized to be EPR silent. Upon light irradiation, the reaction mixture shows a similar well-resolved sextet centered at *g* = 2.0089 with a coupling constant 12.38 G (Scheme 7 and Fig. 4). The intensity of the EPR signals gradually decreased with time and completely diminished after 25 minutes. The second time irradiation of the same reaction mixture again showed a sextet signal in the EPR spectrum. This suggests that continuous irradiation of the reaction mixture is necessary for the generation of the CF_3_ radical to achieve maximum conversion.

### Absorption spectra

**Fig. 5:**
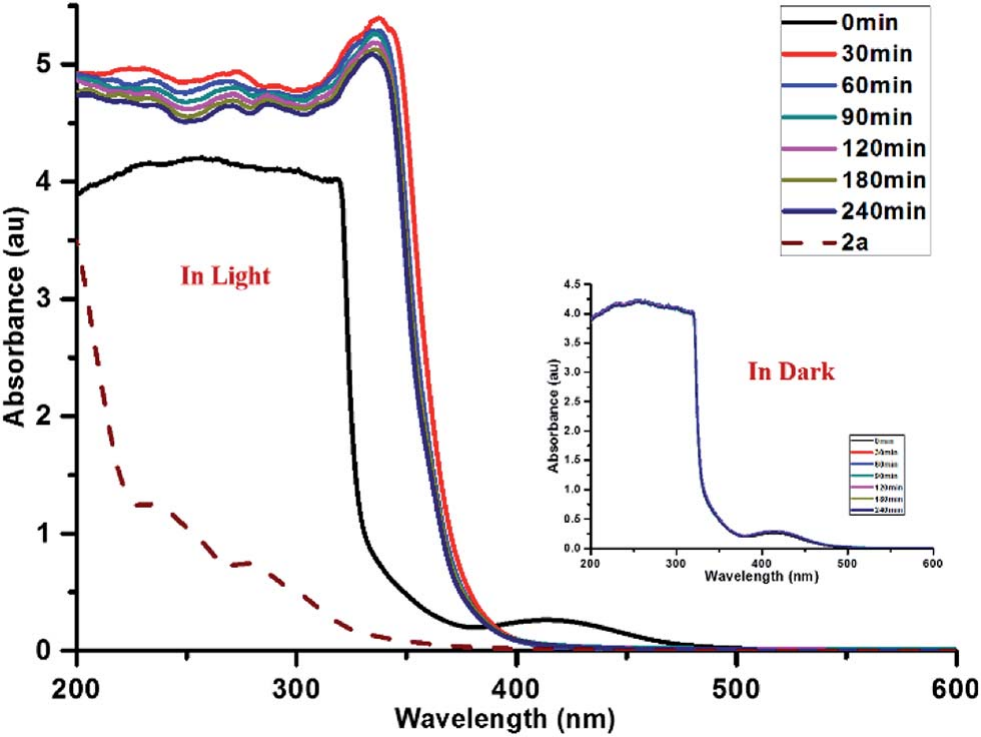
Absorption spectra of **PQ** : CF_3_SO_2_Na : **1a** = 0.1 : 3 : 1 under visible light and dark conditions at different time intervals.

UV-visible spectroscopic studies were performed on the reaction mixture to understand the role of **PQ**. The spectra of **PQ** shows well-resolved absorption maxima at 420 and 510 nm in CH_3_CN and H_2_O (9 : 1) mixtures (Fig. 5). An equimolar mixture of **PQ** and CF_3_SO_2_Na did not lead to any change in the absorption spectrum under dark conditions.

The reaction mixture of **PQ**, CF_3_SO_2_Na and substrate **1a** provided a similar absorption spectrum under dark conditions. Further, the impact of light on the reaction progress was studied for 3 h at 30 min time intervals. Upon sunlight irradiation (30 min) of the reaction mixture, the characteristic peaks of **PQ** completely disappeared (Fig. 5) suggesting the involvement of **PQ** in the oxy-trifluoromethylation reaction.

The absorption spectra of the standard reaction mixture remained nearly unchanged at various time intervals. It seems that photocatalyst **PQ** is transformed into another species, presumably phenanthrene-9,10-diol (**PQH_2_**), which might be the dominant species observable by UV-visible spectroscopy under the reaction conditions (Fig. S13, see the ESI page S14†). A slight increase in the absorption at 420 nm was observed with an increase in time, which could be attributed to the partial regeneration of **PQ** after the completion of the reaction. Further, the regeneration of **PQ** is confirmed by ^13^C NMR spectroscopy and mass spectrometry (ESI, page S16 and S17†).

### Cyclic voltammetric study

**Fig. 6:**
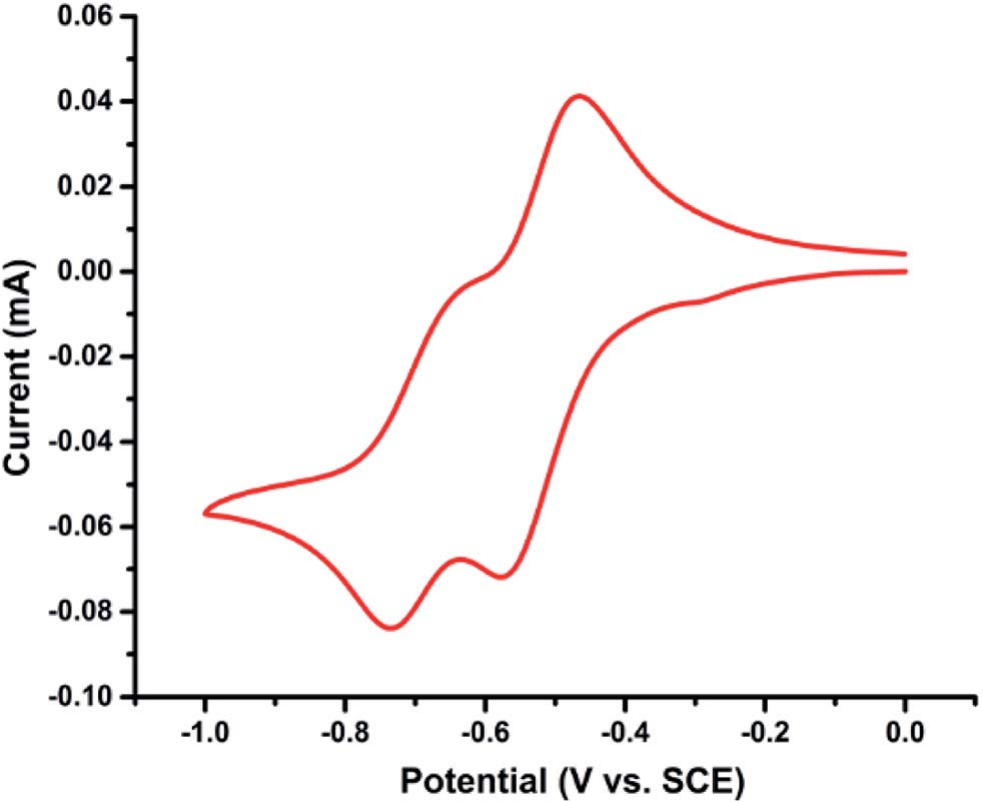
The cyclic voltammogram of **PQ** in CH_3_CN/H_2_O (9 : 1) *vs.* SCE.

**Scheme 8:**
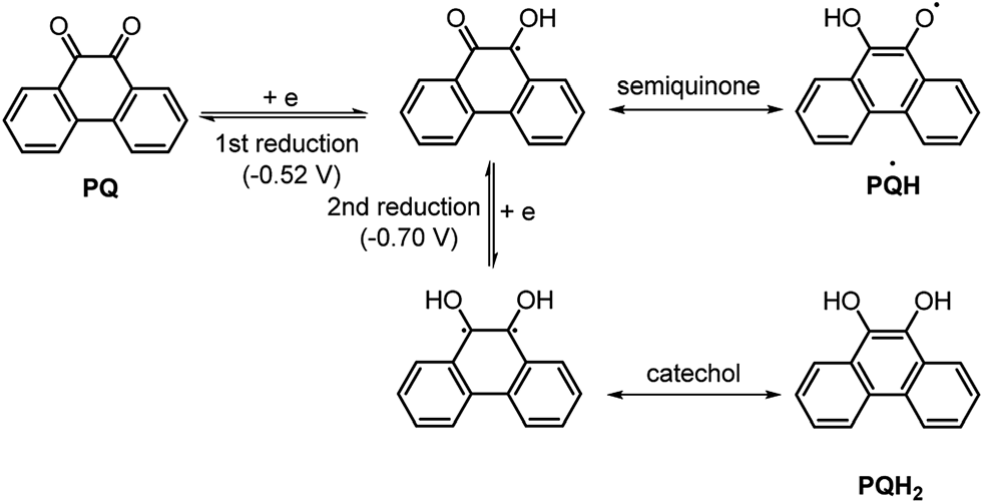
The electrochemical reduction of **PQ** in CH_3_CN/H_2_O (9 : 1).

To gain insights into the redox behaviour of photocatalyst **PQ**, a cyclic voltammetry study was performed (Fig. 6). The cyclic voltammogram (CV) of **PQ** shows reversible two-electron reduction processes at –0.52 and –0.70 V (*E*red1/2) attributed to the quinone → semiquinone and semiquinone → catechol redox couples, respectively (Scheme 8).^35^

The considerably lower reduction potentials of **PQ**, presumably due to the presence of conjugation adjacent to the 9 and 10-positions of the C=O group, provide stability to the radical **PQ˙H** and diol **PQH_2_**. Also, photocatalyst **PQ** exhibited high stability under the electrochemical redox process as it underwent 12-cycles without any loss in the redox activity.

Photocatalyst **PQ** has a triplet excited state energy of 2.116 V.^36*a*^ Thus, the excited state reduction potential *E*red1/2* (^3^ PQ*/**PQ˙^–^**) of **PQ** is 1.6 V (ref. 27*f* and 36*b*) and Langlois’ reagent exhibits an oxidation potential of 1.05 V (*vs.* SCE),^37^ which suggests that **PQ** is strong enough for the oxidation of CF_3_SO_2_Na by single electron transfer (Δ*G*_PET_ = –12.7 kcal mol^–1^).

### Control experiments

**Scheme 9:**
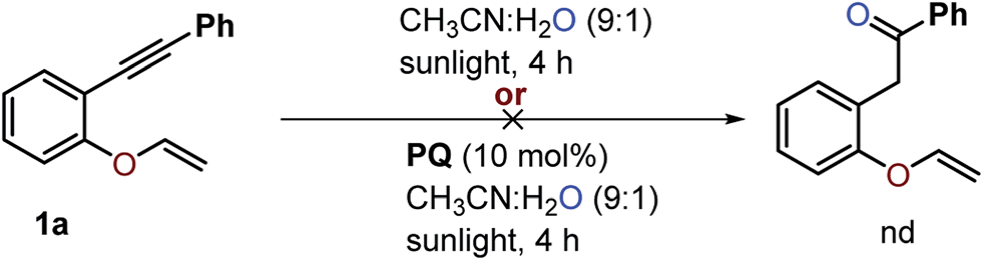
The attempted hydroxylation of 1,6-enyne **1a**. The reaction was carried out using 0.1 mmol of **1a**, 0.3 mmol of CF_3_SO_2_Na and 0.01 mmol of **PQ** in CH_3_CN and H_2_O in a 5 mL round bottom flask.

To study whether water alone is enough to initiate the reaction, substrate **1a** was treated with water in the absence of CF_3_SO_2_Na under optimized reaction conditions (Scheme 9). The hydroxylation of the alkene or alkyne was not observed and 1,6-enyne **1a** was recovered quantitatively. It seems that the substrate does not undergo photohydration of the alkynes^34^ to provide 1-phenyl-2-(2-(vinyloxy)phenyl)ethan-1-one, which is suggestive of the reaction procession being less likely *via* oxygenation followed by trifluoromethylation of substrate **1a**.

When the reaction was performed in the presence of a radical scavenger, TEMPO, the formation of **2a** was not observed. Instead, the coupling between CF_3_ and TEMPO was realized (see ESI, S11†).

### Mechanism, quantum yield and DFT calculations

**Scheme 10:**
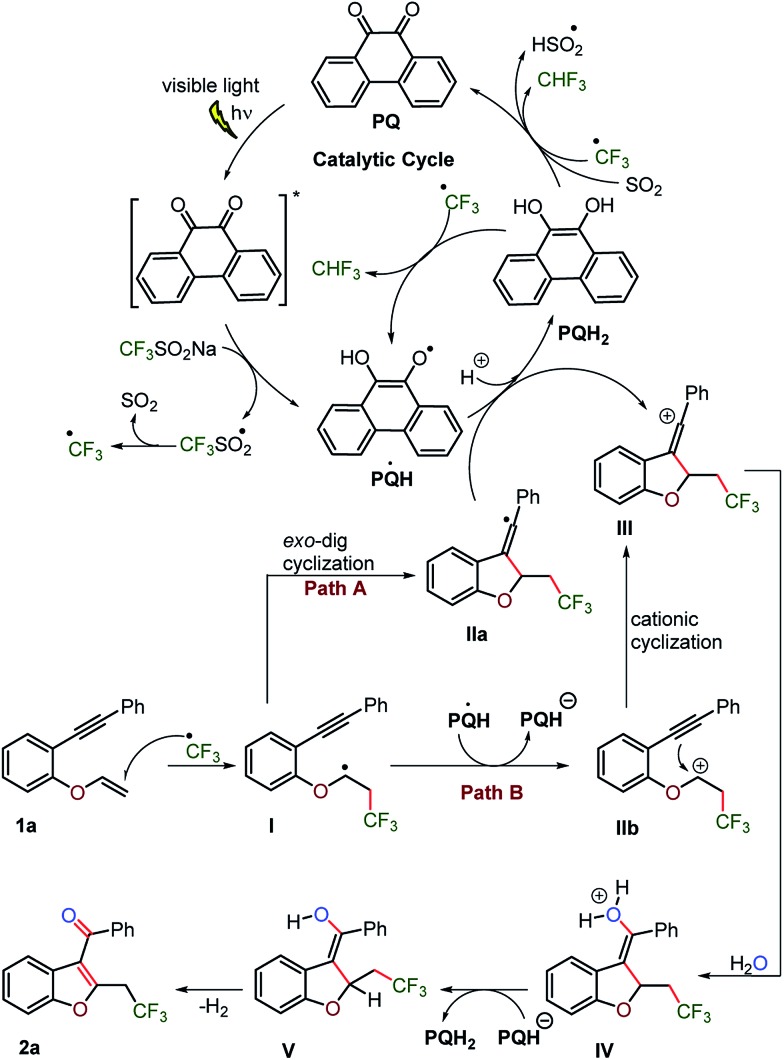
Proposed mechanism for C_3_-aryloyl benzofuran.

Based on the control and labelling experiments, it is reasonable to assume that the photoredox catalyst **PQ**, excited by visible light, activates CF_3_SO_2_Na by single electron transfer to produce a CF_3_ radical and SO_2_ (Scheme 10). The trifluoromethylated radical would add to the vinylic carbon–carbon double bond of the substrate **1a**, forming a radical species **I**, which intramolecularly translocated to the alkyne bond *via* 5-*exo-dig* cyclization and thus rearranged to the vinylic radical **IIa**. The electron transfer from vinylic radical **IIa** to **PQ** would lead to vinylic carbocation **III** and **PQH_2_**. Although, vinylic carbocations have low thermodynamic stability due to the sp-hybridization of the carbenium centre and as a consequence show poor *S*1N-reactivity. However, the sp–sp^2^ rehybridization in the high energy state could account for its electrophilic nature.^38^ The second electron accepting ability of the photoredox catalyst **PQ** would facilitate the formation of vinylic carbocation **III**.

Alternatively, the alkyl ether radical **I** may transfer an electron to **PQ˙H** intermolecularly and convert into carbocation **IIb**, which may proceed by an intramolecular cationic cyclization to provide vinylic carbocation **III**.^39^

The slow addition of water to vinylic carbocation **III** shall provide aquated intermediate **IV**, which upon release of the proton converts into enol intermediate **V**. Further, the photoaromatization of enol **V** would furnish 2-trifluromethylation C_3_-aryloyl benzofuran **2a** along with the concomitant release of hydrogen gas.^40^ As inferred from the UV-visible study (*vide supra*), **PQH_2_** would be the predominant species in the catalytic cycle. **PQH_2_** could regenerate to **PQ** by the transfer of its electrons to sulfur dioxide^37^ and/or the trifluoromethyl radical to form a HSO_2_ radical and/or fluoroform, respectively.

The quantum yield (QY) can in particular provide valuable insight into the mechanistic understanding of the photocatalytic reaction, which involves radical chain propagation. The QY of the developed reaction, substrate **1a** to product **2a**, was studied using the photodecomposition of potassium ferrioxalate, which is a well-explored chemical actinometer.^41^ The determined QY is *φ* = 27 (for details, see the ESI page S17†) which suggests that 27 equivalents of product **2a** are formed for every photon absorbed by the photocatalyst **PQ**. Therefore, the reaction may proceed *via* a chain mechanism. The generated HSO_2_ radical propagates the radical chain by reacting with Langlois’ reagent, which again provides a CF_3_ radical, thus continuing the radical chain reaction.

**Fig. 7:**
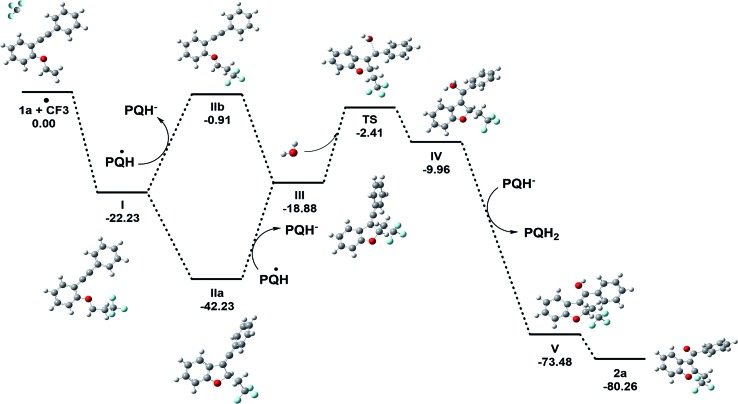
The calculated relative Gibbs’ free energy of the reaction (Δ*G*° in kcal mol^–1^) obtained at a DFT-B3LYP/6-31+G(d)/CPCM(acetonitrile) level of theory.

DFT calculations were explored to examine the mechanism of the reaction (Fig. 7 and Scheme 10). The thermodynamic feasibility of the intermediates and oxygenation by water were computed using DFT-B3LYP/6-31+G(d) in a Gaussian 09 suite in CH_3_CN (see the ESI, page S65–S80†). The Gibbs free energy of the reaction suggests that the proposed intermediates **I–V** are stable under the reaction conditions.^38^ The vinylic cation **III** could be obtained from radical **I** by two paths A and B (Fig. 7) as the energy difference between them is 2.03 kcal mol^–1^. The attack of a water molecule on vinylic cation **III** could be the key step in the transformation and may occur through a transition state **TS**. The energy barrier (Δ*G* ^#^) for the step is +16.47 kcal mol^–1^, which could be feasible under the reaction conditions. Further, the abstraction of the proton from the hydronium ion **IV** by **PQH^–^** provides a stabilization to intermediate **V** by the energy difference of 63.52 kcal mol^–1^ and the subsequent removal of hydrogen gas from the intermediate **V** lowers the energy by 6.78 kcal mol^–1^.

## Conclusions

In summary, we have unveiled an oxidant and TM-free visible light induced oxy-trifluoromethylation of enynes that enables access to biologically important carboxy-trifluoromethylated benzofurans, thiophenes, and indoles. The mild reaction conditions tolerate electronically diverse substrates, regardless of the substitution pattern on either ethynylic or vinylic arene, and as a consequence the methyl group in benzbromarone and JWH015 drugs has been substituted by a trifluoromethyl group. This protocol relies on the universal solvent as a source of oxygen for the oxygenation of enynes. The use of a highly practical 9,10-phenanthrequinone photoredox catalyst, which has a two electron redox property, seems crucial for the transformation as it not only generates trifluoromethyl radicals from the Langlois’ reagent by an electron transfer, but it also brings about one electron oxidation of enynes by a second electron transfer, which in turn facilitates oxygenation utilizing water followed by hydrogen gas evolution under oxidant and TM-free mild conditions. Moreover, we have shown that the di-functionalization of the vinylic double bond adjacent to the heteroatom, which is a formidable task due to the cleavage of the labile carbon–heteroatom bond, can be achieved under the developed conditions. The finding of oxy-trifluoromethylation of enyne substrates under metal and oxidant-free conditions opens a new avenue for the synthesis of trifluoromethylated advance heterocyclic molecules under atom and step economical pathways.
