# Supplementary material for: Visible-light-induced oxidant and metal-free dehydrogenative cascade trifluoromethylation and oxidation of 1,6-enynes with water
Source: Chem Sci. 2017 Jul 10;8(9):6633–44. doi: 10.1039/c7sc02556d (PMC5625288; doi:10.1039/c7sc02556d)
Supplement: Supplementary file 1 [file SC-008-C7SC02556D-s001.pdf]

Electronic Supplementary Material (ESI) for Chemical Science.
This journal is © The Royal Society of Chemistry 2017

Supporting Information

Visible-Light-Induced Oxidant and Metal-Free Dehydrogenative Cascade Trifluoromethylation and Oxidation of 1,6-Enynes with Water

Sadhan Jana, Ajay Verma, Rahul Kadu and Sangit Kumar*

Department of Chemistry, Indian Institute of Science Education and Research (IISER) Bhopal,

Bhopal By-pass Road, Bhauri, Bhopal, Madhya Pradesh, India, 462 066

E-mail: sangitkumar@iiserb.ac.in

Table of Contents

	Page
Table of Contents	S1
General experimental details	S2
Optimization table	S3-S4
Mechanistic investigation	S5-S17
Determination of quantum yield	S17-S19
Synthesis of substrates for	
Benzofuran, benzothiophene and indole	S20-S43
Synthesis of CF_3-containing	
C_3-Aryloyl/acylated benzo[*b*]furans, benzo[*b*]thiophenes and Indoles	S43-S63
Further transformations	S63-S65
Computational: Energy, Cartesian coordinates and methods	S65-S80
Crystal structure details **2c**, **4k**, **6c** and **6d**	
(CCDC NO. 1526060, 1526059, 1526057, and 1526058)	S81-S107
References	S108

General Experimental Details

All reactions were performed in an oven dried glassware containing a magnetic stir bar and sealed with septum. Anhydrous acetonitrile, substituted phenyl acetylenes, $PdCl_2(PPh_3)$ and Langlois' reagent were purchased from Sigma Aldrich. 1,2-Dibromoethane, KO^{t}Bu and Boc_2O were purchased from Spectrochem Pvt. Ltd. Distilled water was used for the reactions. All the photo-induced reactions were performed using borosilicate glassware (5/10 mL RBF) under sunlight/CFL bulb (24W). Reactions were monitored by ^{19}F NMR Spectroscopy and thin-layer Chromatography (TLC). For optimization, reported yields were determined chromatographically and spectroscopically, whereas for substrate scope yields are for the isolated products. NMR experiments were carried out on Bruker 400/500/700 MHz spectrometer in $CDCl_3$/DMSO-d_6/CD_3CN-D_2O/CD_3CN-H_2O solvents and chemical shifts are reported in ppm. The abbreviations used to indicate multiplicity are s (singlet), brs (broad singlet), d (doublet), t (triplet), q (quartet), dd (doublet of doublets) td (triplet of doublet) and m (multiplet). High resolution mass spectroscopic (HRMS) analysis is performed on quadrupole-time-of-flight Bruker MicroTOF-Q II mass spectrometer equipped with an ESI and APCI source; GC-MS analysis is performed on Agilent 7200/Agilent Technologies MS-S975C inert XLEI/CIMSD with triple axis detector. UV-Vis study was performed on Agilent Technologies Cary (5000) series UV-Vis-NIR spectrophotometer. EPR and electrochemical studies were carried out on Bruker EMX microX CW-EPR (34 GHz) series spectrophotometer and CH instrument (Model 700E series), respectively. Single crystal XRD data was collected on a Bruker D8 VENTURE diffractometer equipped with CMOS Photon 100 detector and Mo-Kα (λ = 0.71073 Å) radiation was used. Silica gel (100-200 mesh size) was used for column chromatography purchased from RANKEM Pvt. Ltd. India. TLC plates (Merck silica gel (^{60} F254) plates) used for monitoring the reactions were purchased from Merck.

Table S1. Optimization of reaction conditions using various oxidants[a]

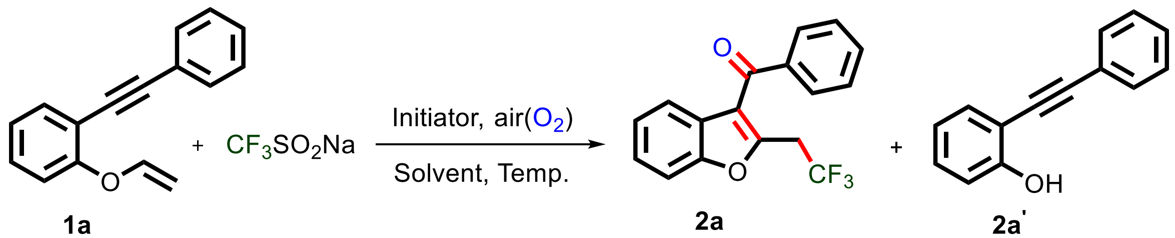

entry	CF_3SO_2Na (equiv.)	oxidants/initiators (equiv.)	additives (mol%)	solvents (2 mL)	T (°C)	Yield[b] **2a** (%)	Yield[b] **2a′** (%)
1	3	$K_2S_2O_8$ (3)	-	DMSO	90	trace	80
2	3	$K_2S_2O_8$ (3)	-	DMSO	50	12	48
3	3	$K_2S_2O_8$ (3)	-	DMSO	25	18	24
4	3	$K_2S_2O_8$ (3)	-	DMF	75	15	62
5	3	$K_2S_2O_8$ (3)	-	CH_3CN	75	10	71
6	3	$K_2S_2O_8$ (2)	-	CH_3CN/H_2O(1:1)	50	nd	trace
7	3	$K_2S_2O_8$ (3)	-	DCE	75	16	55
8	3	$Na_2S_2O_8$ (3)	-	DMSO	75	11	51
9	3	$(NH_4)_2S_2O_8$ (3)	-	DMSO	75	17	49
10	3	Oxone (3)	-	DMSO	75	10	48
11	3	DDQ (3)	-	DMSO	75	nd	nd
12	3	1,4-BQ (3)	-	DMSO	75	nd	nd
13	3	H_2O_2 (3)	-	DMSO	75	12	55
14	3	$K_2S_2O_8$ (2)	TBAI (20)	DMF	90	19	Trace
15	3	TBHP (5)	-	DMF	90	trace	31
16	3	TBHP(5)	TBAI (20)	DMF	90	15	Trace
17	3	DTBP (5)	-	DMF	90	trace	37
18	3	DTBP (5)	I_2(50)	DMF	90	nd	40
19	3	$PhI(OAc)_2$	-	DMF	90	nd	nd
20	3	-	-	NMP	25	14	trace
21	4	-	-	NMP	40	18	15
22	4	-	-	NMP	75	trace	62
23	4	-	KI(20)	THF	25	nd	nd
24[c]	3	AIBN (3)	-	DMSO	75	17	35
25[d]	3	AMVN (3)	-	DMSO	75	13	29
26	4	Vitamine-E (3)	-	DMSO	75	nd	nd

27	4	TEMPO (3)	-	DMSO	75	nd	nd
28	3	-	-	Et_3B THF (1M)	25	nd	nd

[a]All reactions were carried out at **0.2** mmol of **1a** using 0.60 mmol of oxidant/Initiator in 2 mL solvent in a Schlenk tube under oxygen balloon in various temperature and progress of the reaction was monitored by TLC up to 24 h. [b] Yields determined chromatographically and ^{19}F NMR. [c] 2′2-Azobis-2,4-dimethylvaleronitrile (AMVN). [d] Azobis-*iso*butyronitrile (AIBN). nd = not detected

The reaction of 1-(phenylethynyl)-2-(vinyloxy) benzene **1a** with potassium persulphate ($K_2S_2O_8$) (3 equiv) and Langlois' reagent (CF_3SO_2Na) was examined in DMSO under oxygen balloon at 90℃ (Table 1, entry 1). We observed the formation of desired product **2a** as trace along with the formation of 2-(phenylethynyl)phenol **2a**′ in 80% yield. The variation in temperature and solvents fixing $K_2S_2O_8$ as the oxidant (Table 1, entries 2-7) led no further improvement in the yield. With persistent motivation to improve the yield, we changed various persulphates such as $Na_2S_2O_8$, $(NH_4)_2S_2O_8$, oxone (Table 1, entries 8-10), but failed to observe any significant improvement. Various oxidants such as 2,3-dichloro-5,6-dicyano-1,4-benzoquinone (DDQ), 1,4-benznoquninone (BQ), H_2O_2 (30% w/w in H_2O), *tert*-butyl hydroperoxide (TBHP), di-*tert*-butyl peroxide (DTBP), diacetoxyiodobenezene [PhI(OAc)$_2$] gave desired trifluoromethylated product **2a** in low yield (Table 1, entries 10-19). Then we turned our attention to radical initiators; azobis-*iso*butyronitrile (AIBN) and 2'2-azo-bis(2,4-dimethylvaleronitrile (AMVN) in DMSO (Table 1, entries 24-25). Unfortunately, we did not get the good yield of desired product **2a** in both the cases. The reaction was also performed in *N*-methyl-2-pyrrolidone (NMP) solvent, which is effective for the generation of CF_3 radical in the presence of oxygen, at different temperature under oxygen atmosphere and no progress was observed in yield of **2a**. Similarly, various initiators vitamin E, TEMPO and Et_3B (1M solution in THF), known for peroxide generation, realized to be inefficient for the promotion of oxy-trifluoromethylation of 1,6-enyne.

Mechanistic Investigations

In order to get mechanistic insights into visible light-induced oxidant and metal-free dehydrogenative cascade trifluoromethylation and oxidation of enynes with H_2O, following controlled experiments were performed.

Scheme S1. H_2O^{18}-isotope labeling experiment

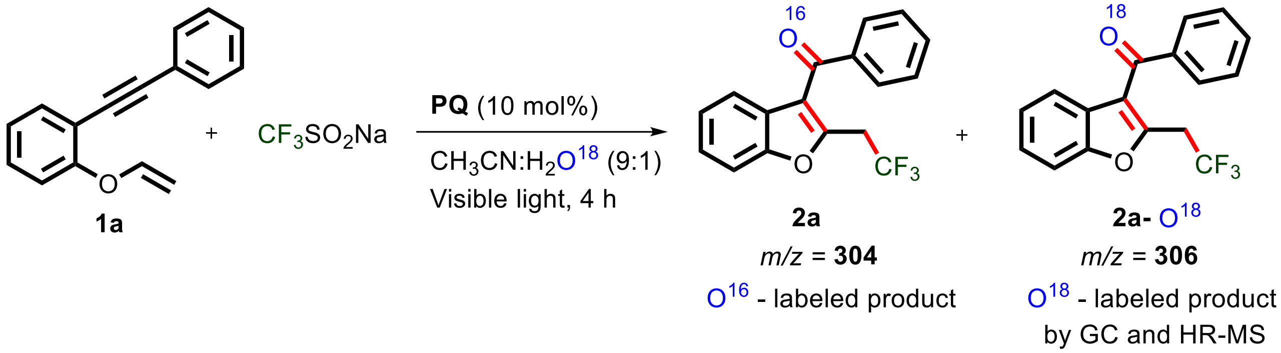

To identify source of oxygen in C_3-aryloyl product, O^{18} labeling experiment was performed using H_2O^{18} under the optimized reaction condition, subsequent mass analysis revealed the formation of O^{18}–labelled trifluoromethylated carbonylatative product **2a**.

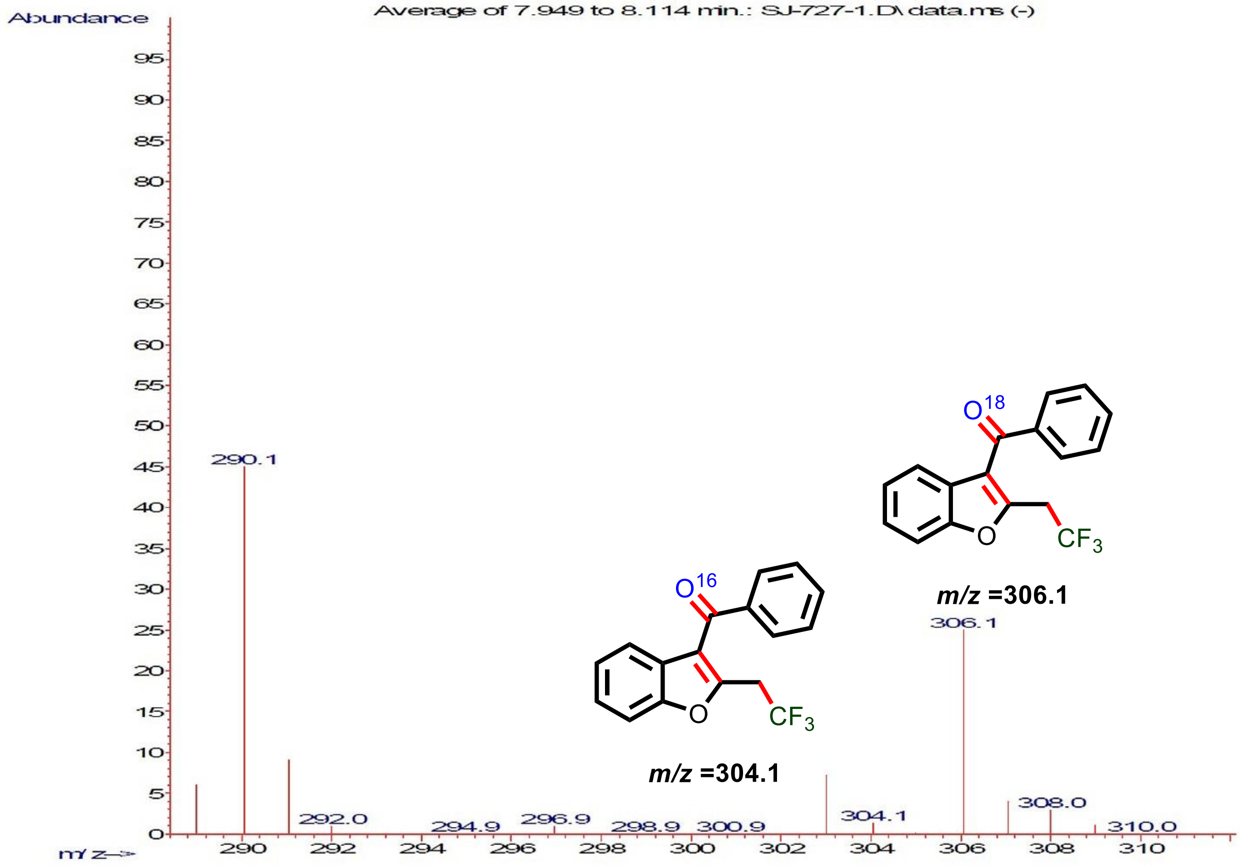

Figure S1. GC-MS Spectra

Acquisition Parameter

Source Type	ESI	Ion Polarity	Positive	Set Nebulizer	1.0 Bar
Focus	Active	Set Capillary	4500 V	Set Dry Heater	250 °C
Scan Begin	50 m/z	Set End Plate Offset	-500 V	Set Dry Gas	7.0 l/min
Scan End	3000 m/z	Set Collision Cell RF	130.0 Vpp	Set Divert Valve	Waste

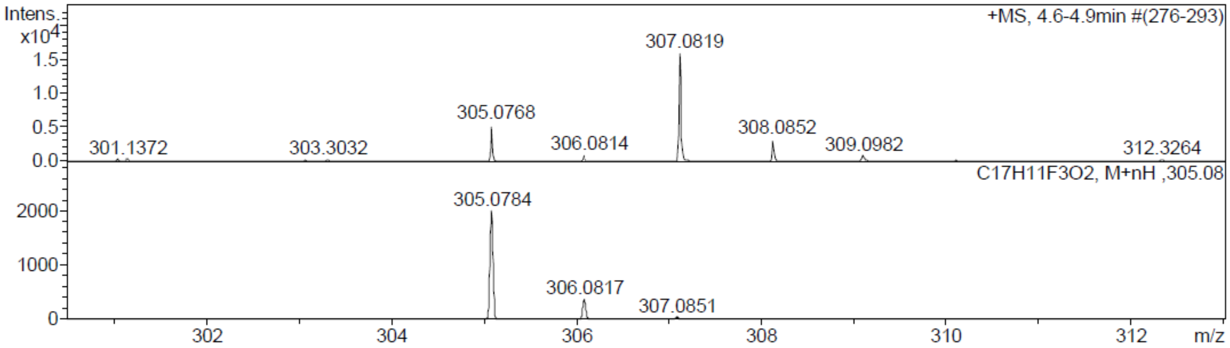

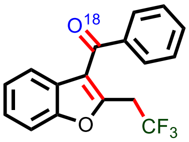

$C_{17}H_{11}F_3O^{16}O^{18}$, (M + H) = 307.0851

Acquisition Parameter

Source Type	ESI	Ion Polarity	Positive	Set Nebulizer	1.0 Bar
Focus	Active	Set Capillary	4500 V	Set Dry Heater	250 °C
Scan Begin	50 m/z	Set End Plate Offset	-500 V	Set Dry Gas	7.0 l/min
Scan End	3000 m/z	Set Collision Cell RF	130.0 Vpp	Set Divert Valve	Waste

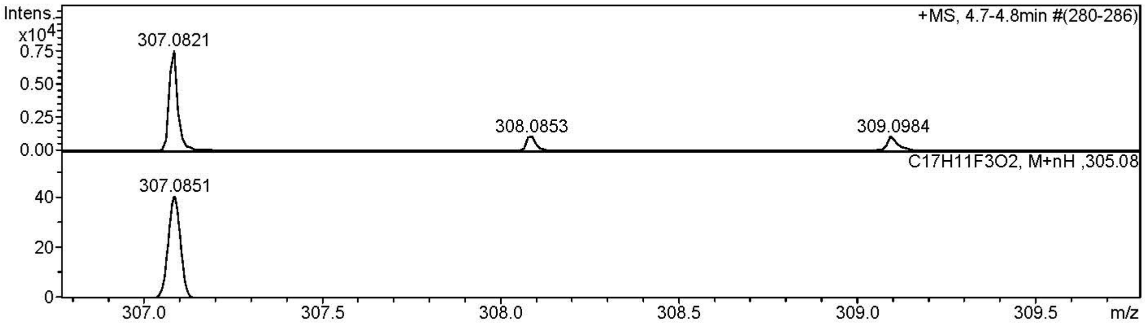

Figure S2. HRMS (ESI), *m/z* calcd for $C_{17}H_{11}F_3O^{16}$ O^{18} $[M+H]^+$ 307.0851, found 307.0821

Scheme S2. Hydrogen evolution experiment

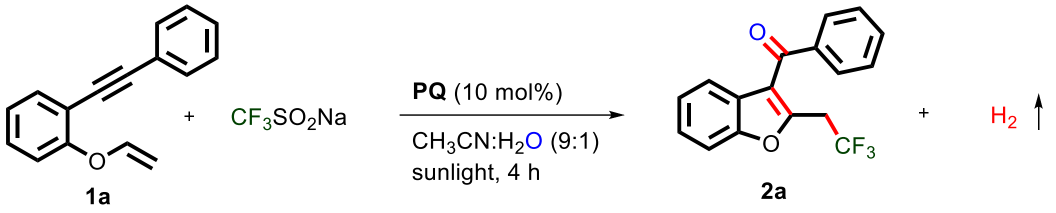

The reaction was carried out at 0.05 mmol of **1a** using 0.15 mmol of CF_3SO_2Na and 0.005 mmol of photocatalyst **PQ** in a Duran NMR tube. The NMR tube was evacuated and backfilled with argon and then CH_3CN+H_2O (450+50μL) was added to the mixture. The reaction mixture was irradiated under visible light for 4h and then NMR spectra was recorded. NMR study suggests the formation of hydrogen gas in the reaction.

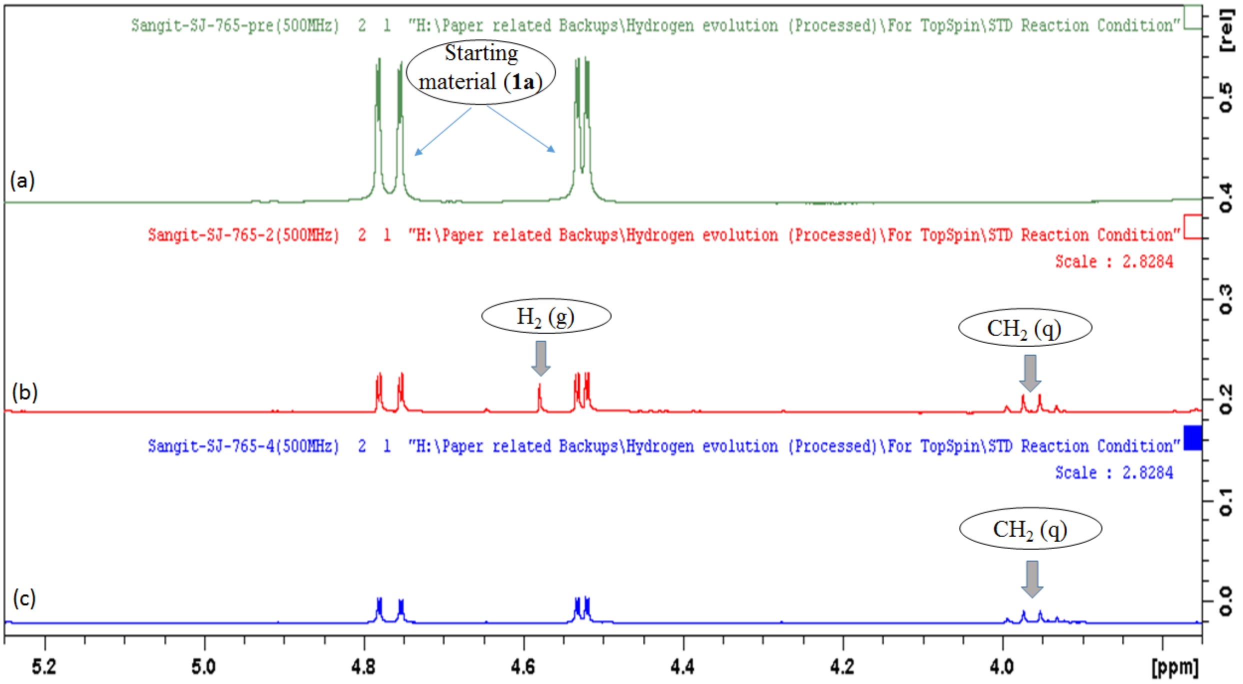

Figure S3. ^{1}H NMR spectra for H_2 gas detection (a) NMR was recorded at the starting of reaction. (b) The reaction mixture was irradiated under visible light for 4h and then NMR spectra was recorded. (c) In the reaction mixture nitrogen gas was bubbled to remove hydrogen gas and recorded NMR spectrum.

Scheme S3. Deuterium oxide (D_2O) labeling experiment

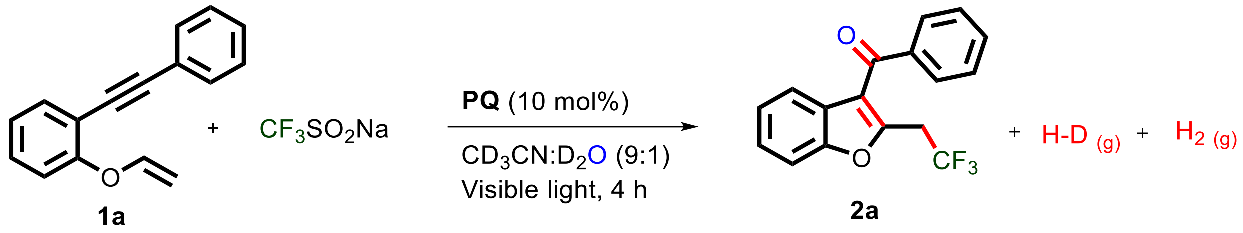

The reaction was carried out at 0.05 mmol of **1a** using 0.15 mmol of CF_3SO_2Na and 0.005 mmol of photocatalyst **PQ** in a Duran NMR tube. The NMR tube was evacuated and backfilled with argon and then CD_3CN+D_2O (450+50µL) was added to the mixture. The reaction mixture was irradiated under visible light for 4h and then NMR spectra was recorded.

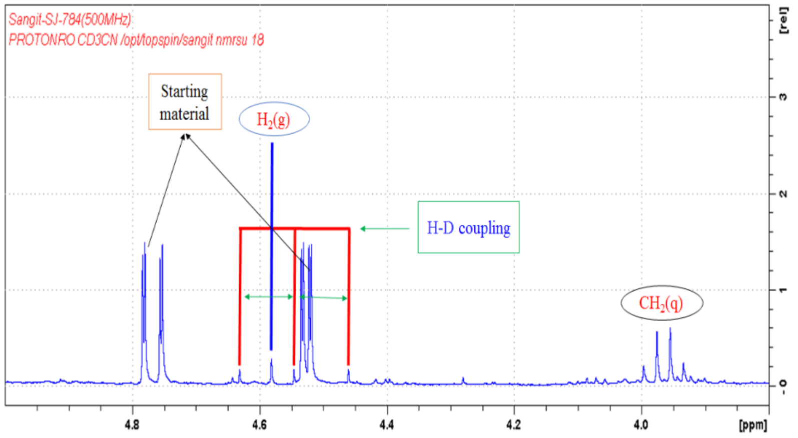

Figure S4. ^{1}H NMR Spectra for the detection of H_2 and HD gases

Existence of HD signal in NMR spectrum suggests the involvement of water in hydrogen gas evolution, as one H could be from substrate **1a** and another H (D) from H_2O (D_2O) molecule.

Scheme S4: Possible exchange of hydrogen and deuterium

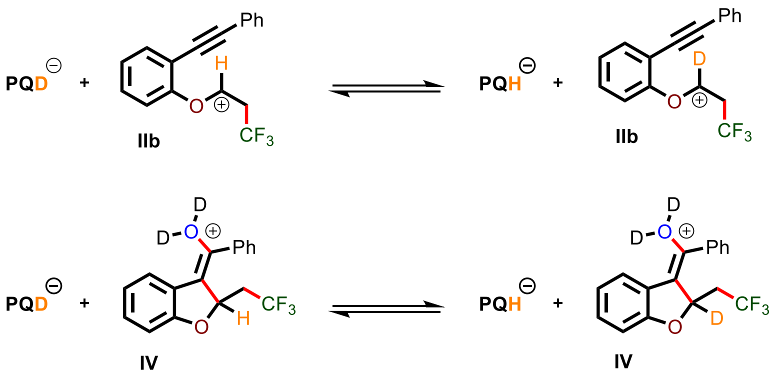

The formation of hydrogen gas in ^{1}H NMR spectrum of Fig S4 due to the presence of H_2O in deuterated solvents (CD_3CN/D_2O) mixture and because of hydrogen and deuterium exchange[1] as shown in Scheme S4.

EPR Investigation

First, EPR experiment was performed on Langlois' reagent and radical trapping reagent 2-methyl-2-nitrosopropane (MNP) dimer under known solvent system.[2a,b]

Scheme S5. Reaction with radical trapping reagent in dichloromethane/water mixture

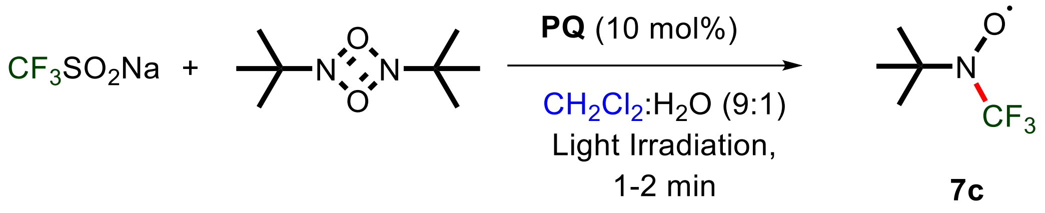

The reaction was carried out at 0.2 mmol of 2-methyl-2-nitrosopropane using 0.3 mmol of CF_3SO_2Na and 0.01 mmol of photocatalyst **PQ** in an EPR tube. The EPR tube was evacuated and backfilled with argon and then CH_2Cl_2+H_2O (450+50µL) was added to the mixture. The reaction mixture was irradiated under light for 1-2 min and then EPR spectra was recorded. EPR signal shows a well resolved sextet with a coupling constant ($g = 2.0054$, $a_N = a_F = 12.27$ G) which indicates trifluoromethyl *t*-butyl nitroxide radical **7c** as reported earlier.[2] Traces of triplet signal is also realized in CH_2Cl_2 and water mixture (see a small triplet at 3363.5G in below Figure S5).

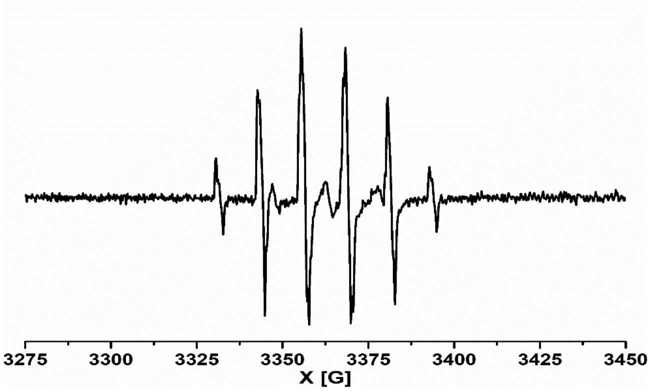

Figure S5. EPR spectra of the reaction (**PQ**+**CF_3SO_2**Na) in dichloromethane/water mixture

Next, EPR experiments were performed under optimized solvent of our photocatalyzed oxy-trifluoromethylation reaction. For this purpose, reaction was carried out at 0.2 mmol of MNP dimer using 0.3 mmol of CF_3SO_2Na and 0.01 mmol of photocatalyst **PQ** in an EPR tube in CH_3CN+H_2O. The EPR tube was evacuated and backfilled with argon and then CH_3CN+H_2O (450+50µL) was added to the mixture. The reaction mixture was irradiated under light for 1-2 min and then EPR spectra was recorded. The EPR signal of the mixture changed from sextet to triplet with a coupling constant 14.7G presumably attributed to dissociated MNP dimer. An additional peak at 3351G also observed and could be due to the interaction between CF_3 radical and photocatalyst **PQ**.

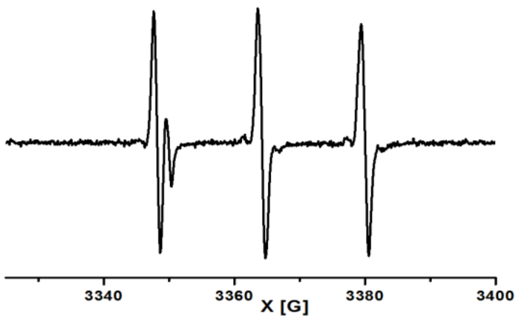

Figure S6. EPR spectra of the reaction (**PQ**+**CF_3SO_2N**a) in acetonitrile/water mixture

Scheme S6. Reaction with radical trapping reagent in acetonitrile/water mixture

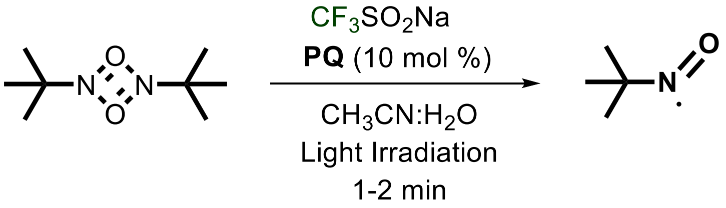

Because the formation of dissociated MNP dimer was observed in acetonitrile/water mixture and the formation of CF_3-trapped radical was not observed in the EPR spectrum. Therefore, further EPR experiments were conducted in dichloromethane/water mixture.

Reaction mixture of MNP dimer, Langlois' reagent and 1,6-enyne **1a** in dark conditions realized to be EPR silent. Upon light irradiation, reaction mixture shows a similar well resolved sextet centered at 2.0089 g with a coupling constant 12.38, which seems attributed to *tert*-butyl-trifluoromethyl nitroxide radical **7c**.

Control Experiment with TEMPO

Scheme S7.

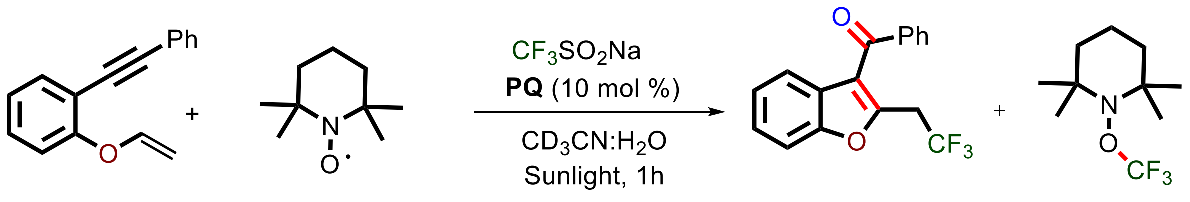

1a TEMPO **2a** (not observed) **7d** (31%)

TEMPO (2 equiv. 0.2 mmol, 31.2 mg) was added to the standard reaction conditions. The reaction mixture was stirred for 1h then internal standard (α,α,α-trifluoro toluene) 0.2 mmol was added. ^{19}F NMR analysis of the reaction mixture showed no desired product (**2a**) was observed; instead, 31% TEMPO-CF_3 (-55.87 ppm) was formed.[2c]

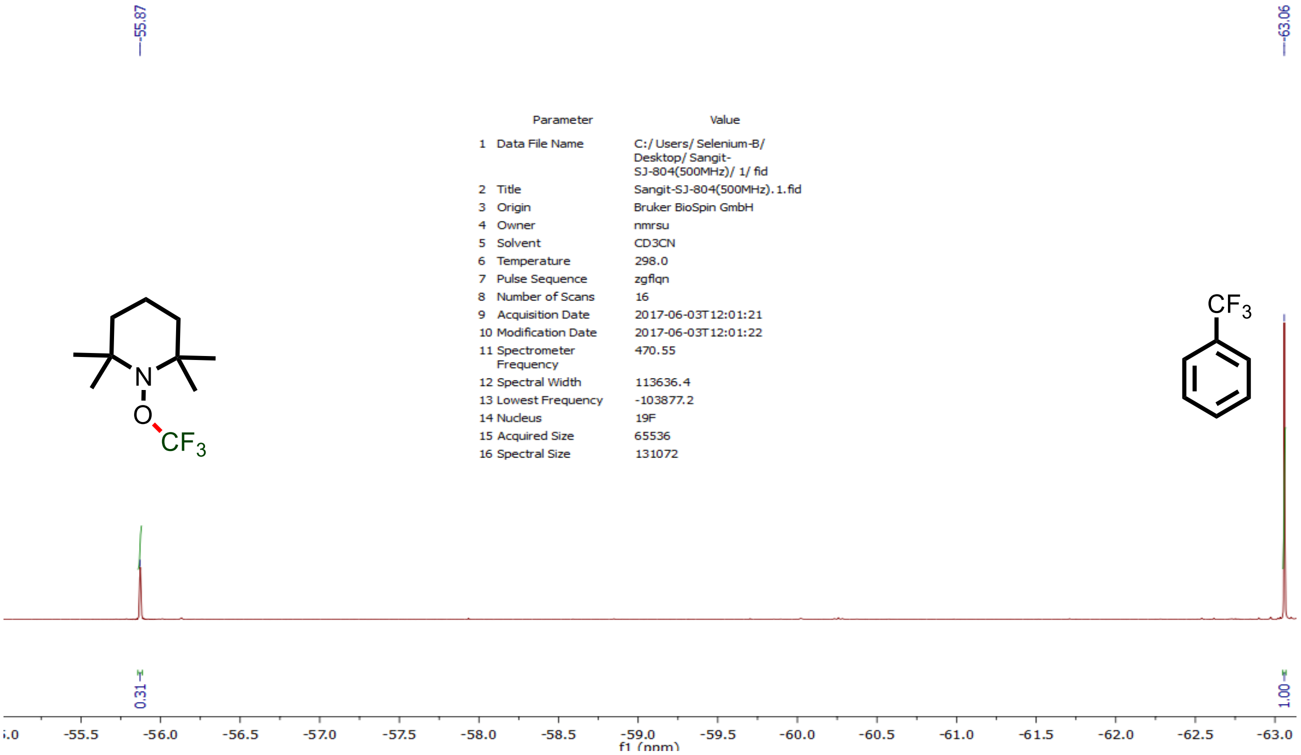

Figure S8. ^{19}F NMR of the crude reaction mixture containing TEMPO

UV-Visible Study

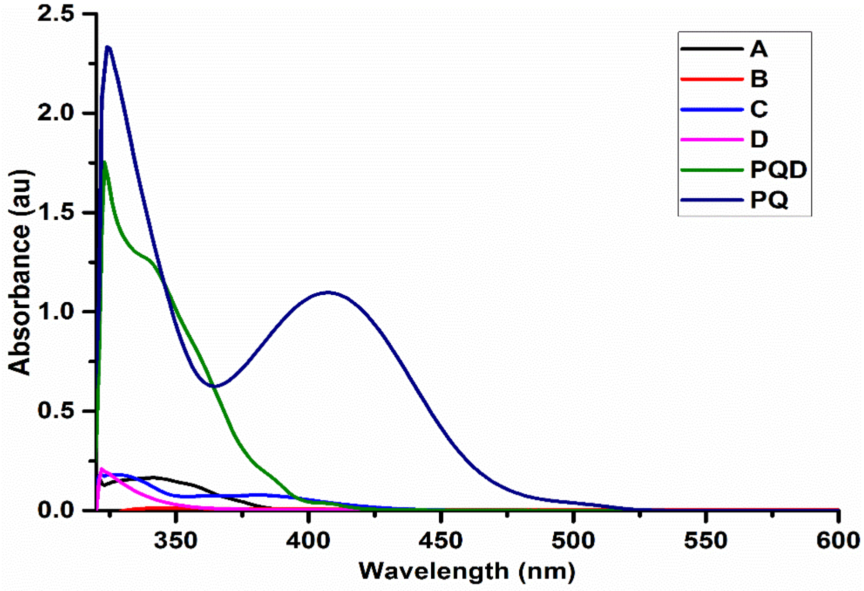

Figure S9. Absorption spectra of various diketones **A-D, PQD** and **PQ** using 1 x 10^{-3} M solution in acetone

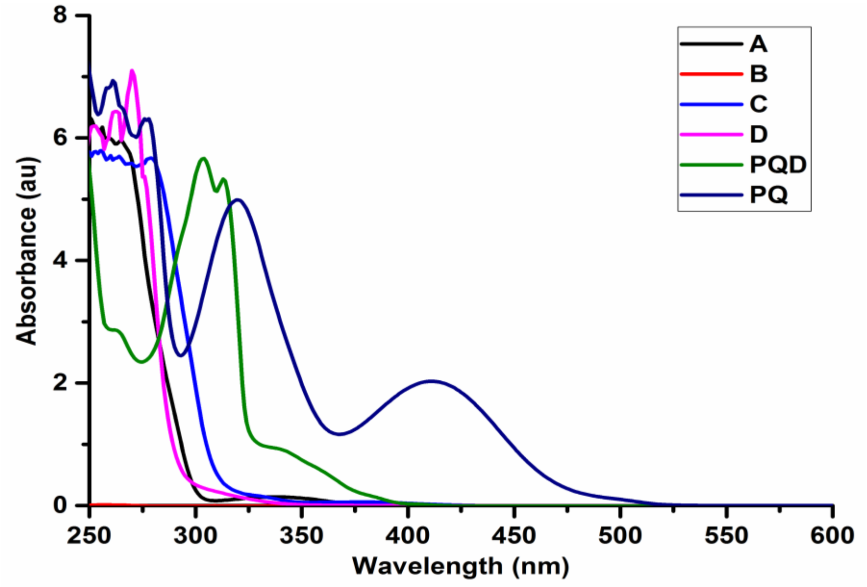

Figure S10. UV-visible spectrum for diketones **A-D, PQD** and **PQ** in acetonitrile (1 x 10^{-3} M)

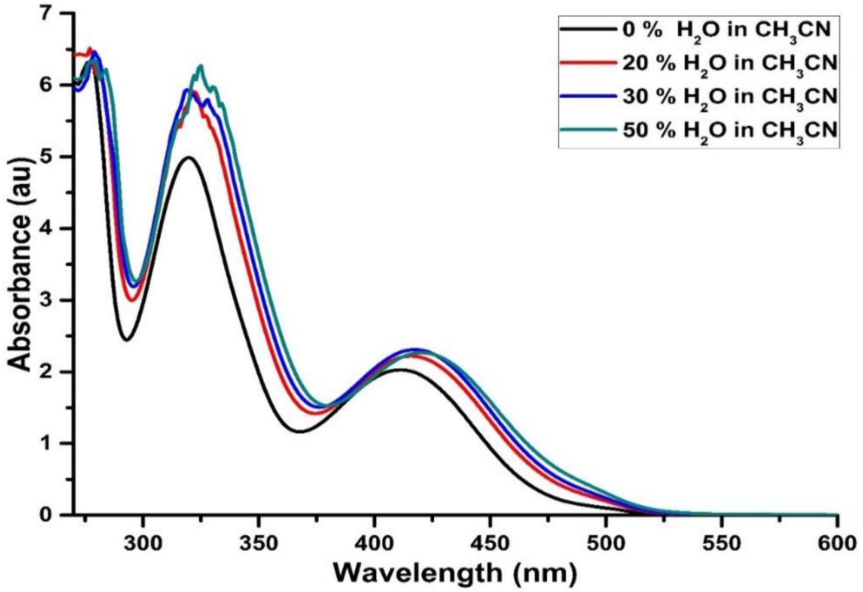

Figure S11. UV-visible spectrum of **PQ** in various acetonitrile/water ratio (1.6 x 10^{-3} M)

This UV-visible study enabled to choose suitable photocatalyst and solvent system in visible light-induced reactions. **PQD** and **PQ** in acetone absorb light in the visible range while in acetonitrile only **PQ** could absorb visible light. With increasing water content in acetonitrile (CH_3CN/H_2O) absorption increases up to 30% and then further increase in water content in acetonitrile decreases the absorbance. This suggests that the <30% water in acetonitrile solvent shall be optimum for absorption of light by **PQ**.

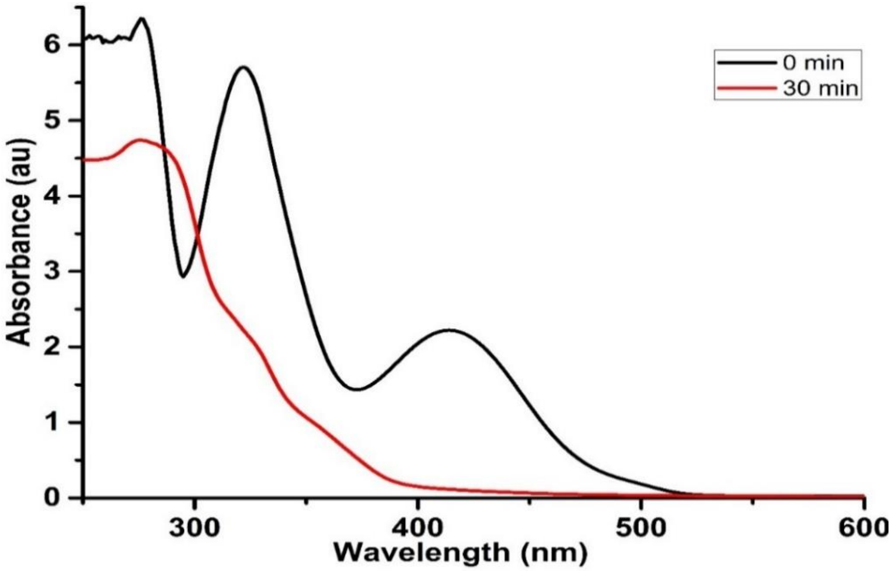

Figure S12. Absorption spectra of reaction mixture between photocatalyst **PQ** and CF_3SO_2Na in acetonitrile/water mixture (3 x 10^{-2} M) at 0 and 30 min, respectively.

Above spectrum shows disappearance of the characteristic peak of **PQ** at 420 nm after visible light irradiation for 30 min, which reveals the photoexcitation of **PQ** and its subsequent reaction with CF_3SO_2Na.

In order to gain more insight on photocatalyst **PQ** in the oxy-trifluoromethylation reaction, absorption spectra of the reaction mixture of substrate **1a**, CF_3SO_2Na and **PQ** in CH_3CN/H_2O (9:1) was studied for every 30 min under light irradiation (Figure S13).

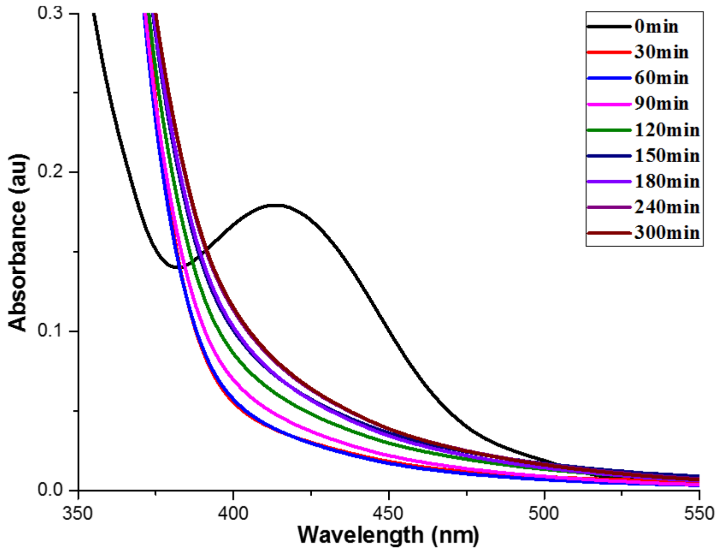

Figure S13. Photocatalyst **PQ** under the Standard Reaction Conditions

Absorption spectra of the optimized reaction mixture observed to be the same at various time intervals (Figure S13). The slight increase in absorption at 420 nm observed with an increase in time. An increase in absorbance could be due to the partial regeneration of **PQ** after completion of the reaction.

Electrochemical analysis of photocatalyst PQ

Electrochemical (cyclic voltammetry (CV) and differential pulse volatmetry (DPV) experiments were carried out by three electrode configuration with a glassy carbon (GC) working electrode, a platinum counter electrode, and standard calomel electrode (SCE) or an $Ag/AgNO_3$ reference electrode.

Reaction condition: 9,10-phenenthrenequinone **PQ** (25 mM) in 5 mL CH_3CN/H_2O (9:1) containing 0.1 M tetrabutylammonium hexafluorophosphate ($TBAPF_6$) with scan rate 50 m Vs^{-1}.

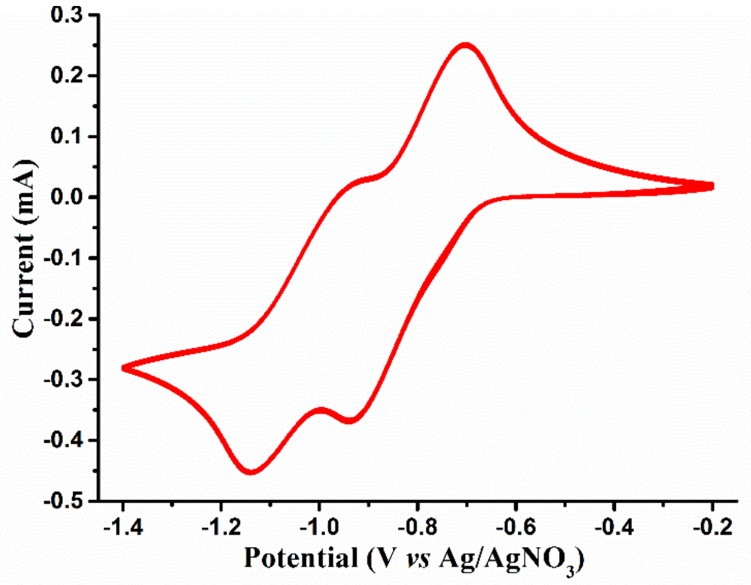

Figure S14. Cyclic Voltammogram of **PQ** in MeCN/ H_2O (9:1) for 12-cycle

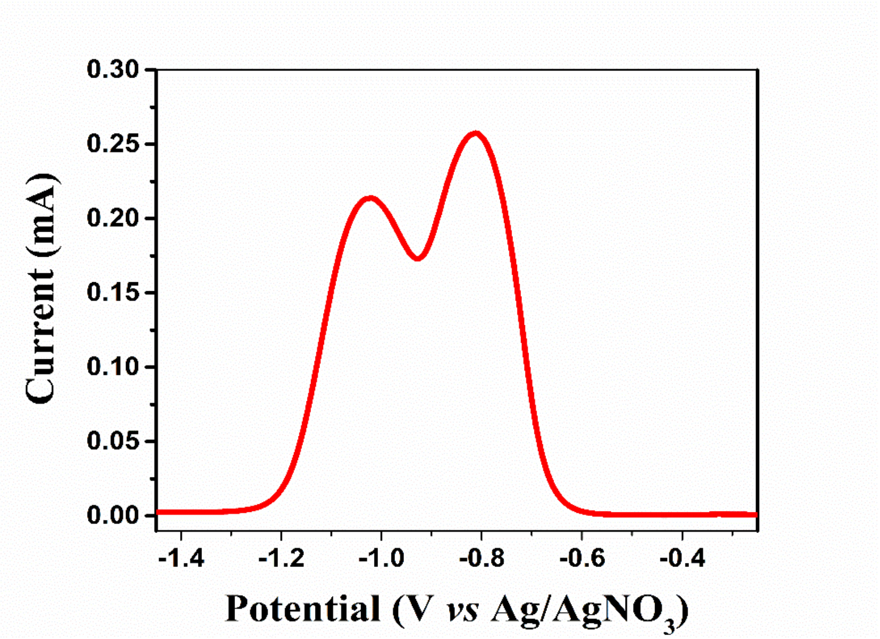

Figure S15. Differential pulse voltammogram of **PQ**

Peak Potential E_{p1} = - 0.80 V E_{p2} = - 1.03 V using Ag/$AgNO_3$

Peak Potential E_{p1} = - 0.52 V E_{p2} = - 0.70 V using SCE

Analysis of regenerated photocatalyst PQ in the reaction by ^{13}C NMR and Mass spectrometry

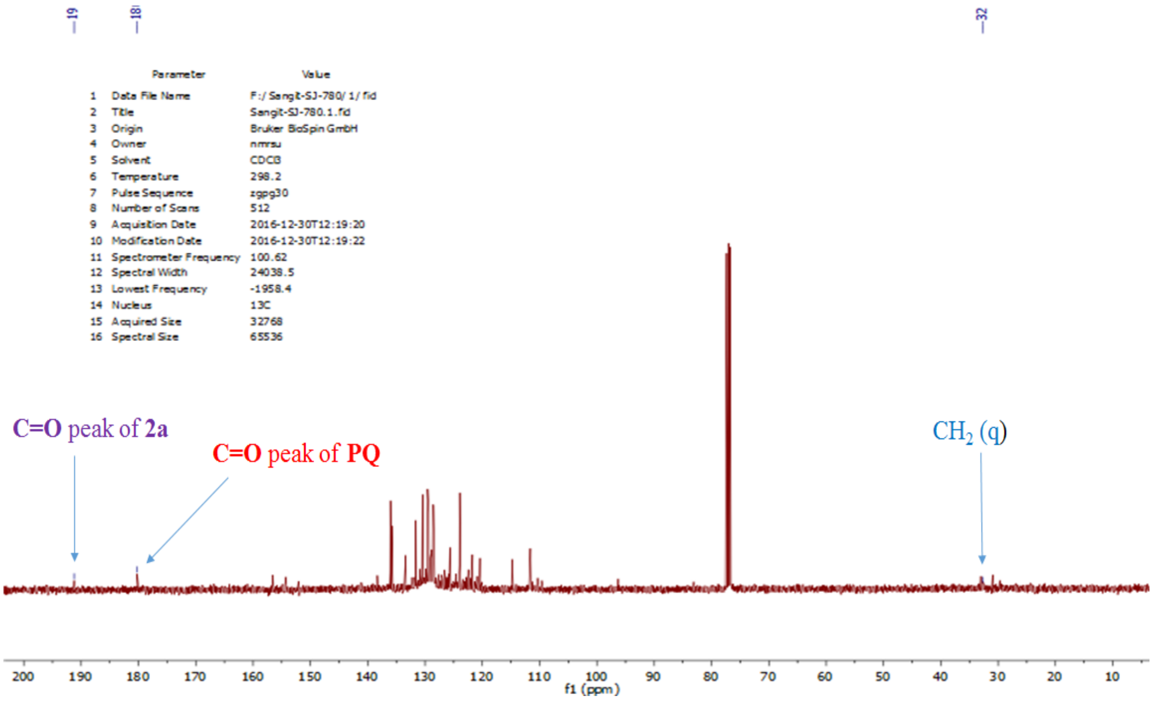

Figure S16. ^{13}C NMR of the crude reaction containing **1a**, Langlois' reagent, and **PQ** after completion of the reaction

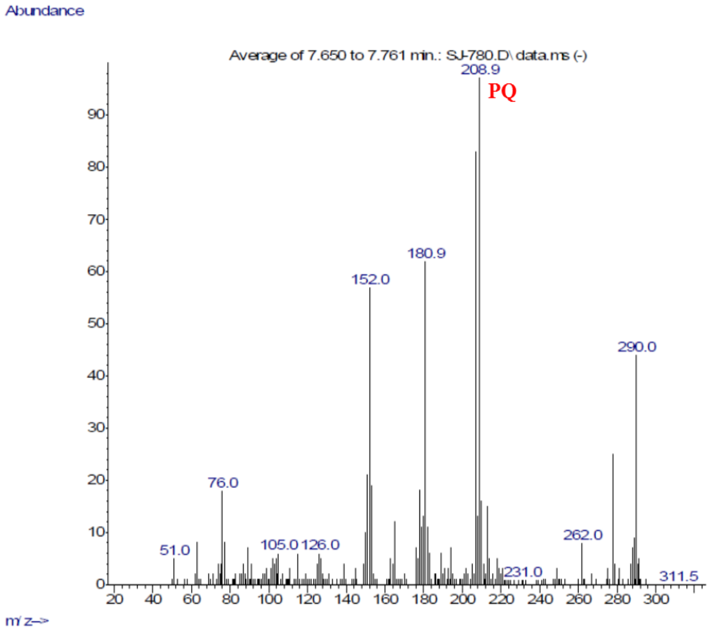

Figure S17. GC-MS of the crude reaction containing **1a**, Langlois' reagent, and **PQ** after completion of the reaction

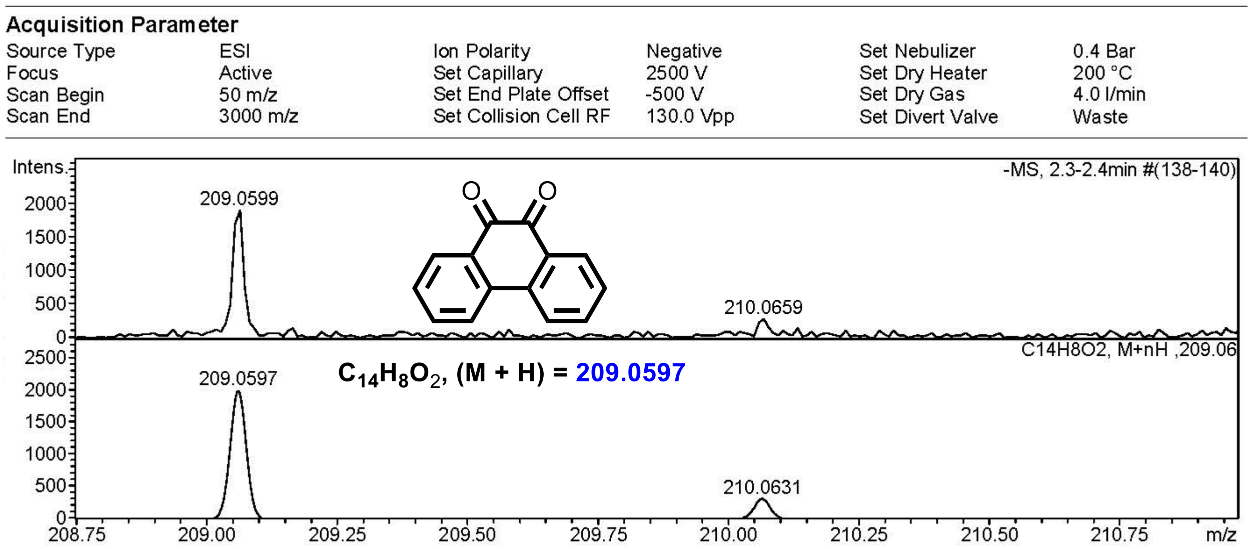

Figure S18. HRMS of the crude reaction containing **1a**, Langlois' reagent, and **PQ** after completion of the reaction

Regeneration of **PQ** was confirmed by the appearance of characteristic ^{13}C-NMR signal at 180.2 ppm form crude reaction mixture and peak at *m/z* 208.9 in GC-MS spectrum of crude reaction mixture. HRMS (ESI), *m/z* calcd for $C_{14}H_8O_2$ $[M+H]^+$ 209.0597, found 209.0599.

Determination of Quantum Yield (QY)[3]

Determination of the light intensity at 436 nm: The photon flux of the spectrometer was determined by standard ferrioxalate actinometry. For this, two solutions were prepared. A 0.15 M solution of ferrioxalate was prepared by dissolving 736.6 mg of potassium ferrioxalate hydrate in 10 mL of 0.05 M H_2SO_4. A buffer solution of 1,10-phenanthroline was prepared by dissolving 16.77 mg of 1,10-phenenthroline and 3.75 g of sodium acetate in 16.7 mL of 0.05 M H_2SO_4. Both solution were stored in dark.

To determine the photon flux of the spectrophotometer, 1.0 mL of the ferrioxalate solution was placed in a cuvette and irradiated for 30 min at λ = 436 nm. After irradiation, 0.175 mL of the 1,10-phenanthroline solution was added to the cuvette. The solution was then rested for 1 h so that ferrous ions completely coordinate to the 1,10-phenanthroline. The absorbance of the solution was

measured at 510 nm. A non-irradiated sample was also prepared and the absorbance at 510 nm measured. Conversion was calculated using eq 1 .

$$\text{mol } Fe^{2+} = V \bullet \Delta A / l \bullet \varepsilon \qquad (1)$$

Where V is the total volume (1.175 mL = 0.001175 L) of the solution after addition of phenanthroline, ΔA is the difference in absorbance at 510 nm between the irradiated and non-irradiated solutions, l is the path length (1.000 cm), and ε is the molar absorptivity at 510 nm (11,100 L mol^{-1} cm^{-1}). The photon flux can be calculated using eq. 2

$$\text{photon flux} = \text{mol } Fe^{2+} / \Phi \bullet t \bullet f \qquad (2)$$

Where Φ is the quantum yield for the ferrioxalate actinometer (1.01 for a 0.15 M solution at λ = 436 nm), t is the time (1800.0 s), and f is the fraction of light absorbed at λ = 436 nm (0.998187, *vide infra*). The photon flux was calculated to be 4.9375 × 10^{-11} einstein s^{-1}.

Calculations:

$$\text{mol } Fe^{2+} = 1.175 \text{ x } 10^{-3} \text{ L} \bullet 0.84608/1.000 \text{ cm} \bullet 11{,}100 \text{ L mol}^{-1} \text{cm}^{-1}$$
$$= 8.96 \times 10^{-8} \text{ mol}$$

$$\text{Photon flux} = 8.96 \times 10^{-8} \text{ mol}/1.01 \bullet 1800.0 \text{ s} \bullet 0.998187$$
$$= 4.9375 \times 10^{-11} \text{ einstein s}^{-1}$$

Determination of fraction of light absorbed at 436 nm for the ferrioxalate solution:

The absorbance of the above ferrioxalate solution at 436 nm was measured to be 2.7416. The fraction of light absorbed (f) by this solution was calculated using eq 3, where A is the measured absorbance at 436 nm.

$$f = 1 - 10^{-A} = 0.998187\ldots\ldots\ldots\ldots\ldots(3)$$

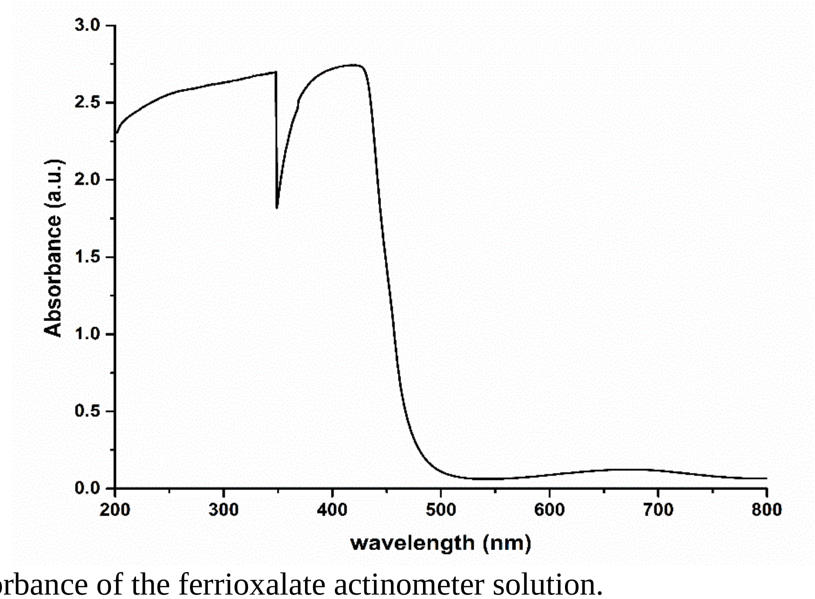

Figure 19. Absorbance of the ferrioxalate actinometer solution.

Determination of Quantum Yield of Optimized Reaction Condition

Scheme 8. Reaction of **1a**, CF_3SO_2Na and **PQ**

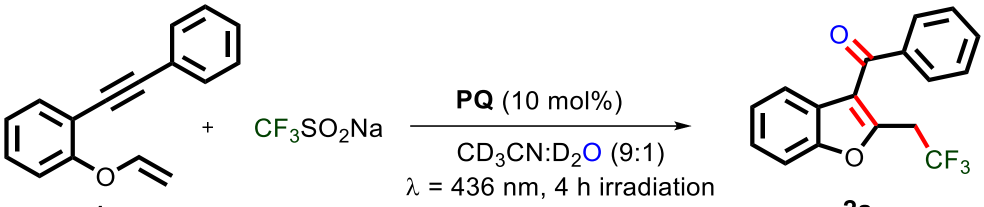

A cuvette was charged with 1-(phenylethynyl)-2-(vinyloxy)benzene **1a** (22 mg, 0.1 mmol, 1.0 equiv.), CF_3SO_2Na (47 mg, 0.6 mmol), photocatalyst (PQ) (2.1 mg, 0.01 mmol, 0.1 equiv.), and 1.0 mL CD_3CN/D_2O (9:1). The cuvette was then capped with a PTFE stopper. The sample was irradiated ($\lambda = 436$ nm) for 14400 s (4 h). After irradiation, the solution was passed through a silica plug. The yield of product formed was determined by ^{19}F NMR based on a standard 4-fluorotoluene. The quantum yield was determined using eq 4. Essentially all incident light ($f > 0.999$, *vide infra*) is absorbed by the photocatalyst **PQ** at the reaction conditions described above.

$$\Phi = \text{mol product/flux} \bullet t \bullet f \qquad (4)$$

The yielded 21% of desired product **2a** and quantum yield (Φ) = 27

$$\Phi = 1.9068 \times 10^{-5} \text{ mol} / 4.9375 \times 10^{-11} \text{ einstein s}^{-1} \bullet 14400 \text{ s} \bullet 1.0$$
$$= 27.$$

General Procedure for the Synthesis of Substrates

Scheme S9. Preparation of 1-(phenylethynyl)-2-(vinyloxy)benzene **1a**

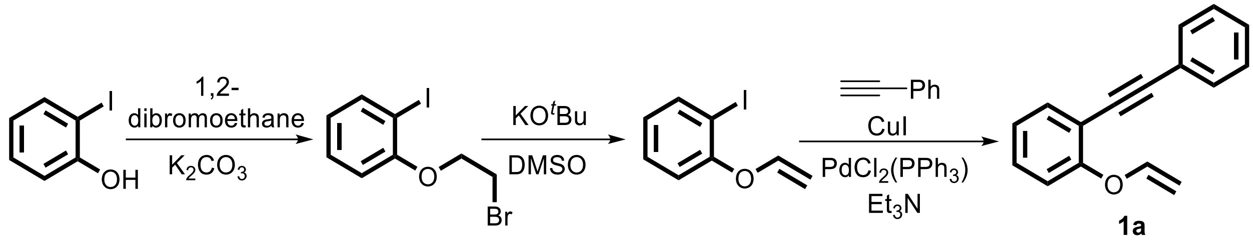

Substrates 1-(phenylethynyl)-2-(vinyloxy) benzenes **1a-1r** were prepared by the literature procedures.[4]

Synthesis of 1-(2-Bromoethoxy)-2-iodobenzene from 2-Iodophenol

To a stirred solution of 2-iodophenol (4.55 mmol, 1.00 g) and 1,2-dibromoethane (22.75 mmol, 2 mL) in acetone (50 mL) was added K_2CO_3 (9.10 mmol, 1.26 g). The resulting mixture was stirrer at room temperature for overnight. The reaction was quenched with water (10 mL) and extracted with CH_2Cl_2 (20 mL x 3). The organic layer was washed with brine (10 mL), dried over Na_2SO_4 and concentrated by rotary evaporator under reduced pressure. The crude product was purified by column chromatography over silica gel using hexane on silica gel. A white solid of 1-(2-bromoethoxy)-2-iodobenzene was obtained.[4] Yield (1.13 g, 76%), ^{1}H-NMR (500 MHz, $CDCl_3$), δ 7.82 (dd, *J* = 7.8, 1.7, 1H), 7.34-7.30 (m, 1H), 6.82-6.76 (m, 2H), 4.31 (t, *J* = 6.4 Hz, 2H), 3.70 (t, *J* = 6.4 Hz, 2H), ^{13}C NMR (125 MHz, $CDCl_3$), δ 156.7, 139.7, 129.7, 123.4, 112.9, 87.0, 69.2, 29.1.

Synthesis of 1-Iodo-2-(vinyloxy)benzene from 1-(2-Bromoethoxy)-2-iodobenzene

A solution of 1-(2-bromoethoxy)-2-iodobenzene (1.33 mmol, 436 mg) in DMSO (10 mL) was stirred at 0 ^{0}C. To this stirrer solution was added KO^{t}Bu (2.0 mmol, 224 mg) in portions under nitrogen. The resulting mixture was stirred at room temperature for 2 h. The reaction was quenched with water (100 mL) and extracted with CH_2Cl_2 (50 mL x 4). The combined organic layer was washed with brine (100 mL), dried over Na_2SO_4 and concentrated by rotary evaporator under reduced pressure. The crude product was purified by column chromatography on silica gel using hexane. 1-Iodo-2-(vinyloxy)benzene was obtained as a yellow oil.[4] Yield (415 mg, 68%), ^{1}H-NMR (500 MHz, $CDCl_3$), δ 7.84 (dd, *J* = 7.8, 1H), 7.36-7.33 (m, 1H), 7.01 (dd, *J* = 8.2, 1.4 Hz, 1H), 687

(td, J = 7.6 Hz, 1H), 6.61 (dd, J = 6.1, 14.0 Hz, 1H), 4.80 (dd, J = 13.7, 2.04 Hz, 1H), 4.54 (dd, J = 6.1, 2.0 Hz, 1H), ^{13}C NMR (125 MHz, $CDCl_3$), δ 155.8, 148.0, 139.7, 129.5, 125.1, 117.3, 95.8, 87.4.

Synthesis of 1-(Phenylethynyl)-2-(vinyloxy) benzene (1a) from 1-Iodo-2-(vinyloxy)benzene

To a solution of 1-iodo-2-(vinyloxy)benzene (1.00 mmol, 246 mg) and phenyl acetylene (1.10 mmol, 112 mg) in trimethylamine (degassed, 8 mL) was added $PdCl_2(PPh_3)$ (0.02 mmol, 14 mg) and CuI (0.04 mmol, 8 mg) under nitrogen. The resulting mixture was stirrer at room temperature for 6 h. The reaction mixture was filtered and washed with diethyl ether. The combined filtrate was concentrate under reduced pressure and the residue was purified by column chromatography over silica gel using hexane. A light greenish oil of **1a** was obtained.[4] Yield (187 mg, 85%), ^{1}H-NMR (500 MHz, $CDCl_3$), δ 7.60-7.56 (m, 3H), 7.40-7.32 (m, 4H), 7.12 (td, J = 7.5, 0.7 Hz, 1H), 7.07 (d, J = 8.3 Hz, 1H), 6.75-6.71 (m, 1H), 4.83 (dt, J = 13.8, 1.6 Hz, 1H), 4.51 (dd, J = 6.1, 1.6 Hz, 1H), ^{13}C NMR (125 MHz, $CDCl_3$), δ 157.0, 148.6, 133.5, 131.6, 129.6, 128.33, 128.31, 123.39, 123.36, 117.2, 114.6, 96.0, 94.1, 85.0.

By using different substituted phenyl acetylene, various substrates (**1b**, **1c**, **1d**, **1e**, **1f**, **1g**, **1h**, **1i**, **1j**, **1k**, **1l**, **1m**, **1n**, **1o**, **1p**, **1q**, **1r**, and **1s**) were synthesized. Yields and analytical data for these substrates listed below.

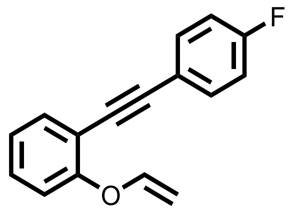

1-((4-Fluorophenyl)ethynyl)-2-(vinyloxy)benzene (1b):[5] White viscous liquid, yield (214 mg, 90%), ^{1}H-NMR (400 MHz, $CDCl_3$), δ 7.53-7.50 (m, 3H), 7.30 (td, J = 7.8, 1.6 Hz, 1H), 7.08 (dd, J = 7.6, 0.8 Hz, 1H), 7.03 (t, J = 8.6 Hz, 3H), 6.67 (dd, J = 13.7, 6.1 Hz, 1H), 4.78 (td, J = 13.7, 1.7 Hz, 1H), 4.47 (dd, J = 6.1, 1.7 Hz, 1H), ^{13}C NMR (100 MHz, $CDCl_3$), δ 163.8, 161.3, 157.0, 148.6, 133.5, 133.4 (d, J = 3.5 Hz), 129.7, 123.3, 119.4 (d J = 3.5 Hz), 117.2, 115.7, 115.4, 114.4, 96.1, 92.9, 84.6.

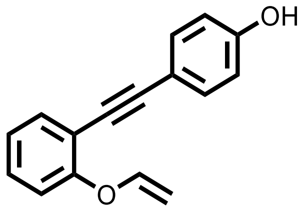

4-((2-(Vinyloxy)phenyl)ethynyl)phenol (1c): Yellow solid, yield (154 mg, 65%), ^{1}H-NMR (400 MHz, $CDCl_3$), δ 7.53-7.49 (m, 3H), 7.28 (td, *J* = 7.9, 1.5 Hz, 1H), 7.10-7.05 (m, 3H), 7.02 (d, *J* = 8.2 Hz, 1H), 6.68 (dd, *J* = 13.8, 6.1 Hz, 1H), 5.74 (s, 1H), 4.79 (dd, *J* = 13.8, 1.7 Hz, 1H), 4.47 (dd, *J* = 6.1, 1.6 Hz, 1H), ^{13}C NMR (100 MHz, $CDCl_3$), δ 156.9, 156.8, 148.7, 133.4, 133.1, 129.4, 123.3, 117.5, 117.3, 116.3, 114.8, 94.9, 93.8, 84.2. HRMS (APCI), *m/z* calcd for $C_{16}H_{12}O_2$ [M-H]$^{+}$ 235.0754, found 235.0732.

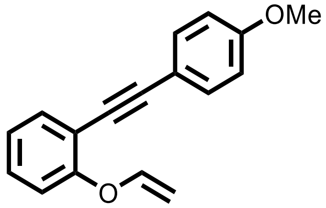

1-((4-Methoxyphenyl)ethynyl)-2-(vinyloxy)benzene (1d):[5] White solid, yield (221 mg, 88%), ^{1}H-NMR (400 MHz, $CDCl_3$), δ 7.51-7.46 (m, 3H), 7.29-7.24 (m, 1H), 7.06 (td, *J* = 7.5, 0.8 Hz, 1H), 7.01 (d, *J* = 8.1 Hz, 1H), 6.86 (d, *J* = 8.8 Hz, 2H), 6.67 (dd, *J* = 13.8, 6.0 Hz, 1H), 4.77 (dd, *J* = 13.8, 1.7 Hz, 1H), 4.4 (dd, *J* = 6.0, 1.7 Hz, 1H), 3.8 (s 3H), ^{13}C NMR (100 MHz, $CDCl_3$), δ 159.6, 156.8, 148.7, 133.4, 133.0, 129.2, 123.3, 117.3, 115.5, 115.0, 113.9, 94.8, 94.1, 83.5, 53.3.

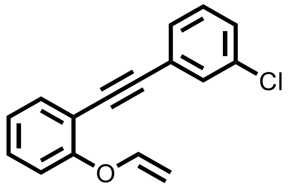

1-((3-Fluorophenyl)ethynyl)-2-(vinyloxy)benzene (1e): Light yellow viscous liquid, yield (244 mg, 96%), ^{1}H-NMR (400 MHz, $CDCl_3$), δ 7.54-7.49 (m, 2H), 7.42 (dt, *J* = 7.2, 1.3 Hz, 1H), 7.34-7.26 (m, 3H), 7.08 (td, *J* = 7.6, 0.7 Hz, 1H), 7.03 (d, *J* =8.2 Hz, 1H), 6.7(dd, *J* = 13.9, 6.1 Hz, 1H), 4.80 (dd, *J* = 13.9, 1.7 Hz, 1H), 4.49 (dd, *J* = 6.1, 1.7 Hz, 1H), ^{13}C NMR (125 MHz, $CDCl_3$), δ 157.1, 148.4, 134.1, 133.6, 131.4, 130.0, 129.7, 129.5, 128.5, 125.1, 123.3, 117.1, 114.1, 95.3, 92.5, 86.2, HRMS (ESI), *m/z* calcd for $C_{16}H_{11}ClO$ [M+H]$^{+}$ 255.0571, found 255.0547.

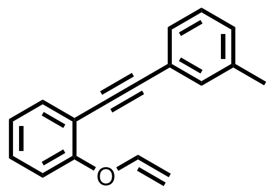

1-(*m*-Tolylethynyl)-2-(vinyloxy)benzene (1f):[6] White viscous liquid, yield (238 mg, 97%), ^{1}H-NMR (500 MHz, $CDCl_3$), δ 7.54 (dd, *J* = 7.7, 1.6 Hz, 1H), 7.37 (d, *J* = 9.5 Hz, 2H), 7.30 (td, *J* = 7.9, 1.6 Hz, 1H), 7.24 (t, *J* = 7.6 Hz, 1H), 7.15 (d, *J* = 7.6, 1H), 7.1 (td, *J* = 7.6, 0.9 Hz, 1H), 7.0 (d, *J* = 8.2 Hz, 1H), 6.7(dd, *J* = 13.8, 6.1 Hz, 1H), 4.8 (dd, *J* = 13.8, 1.7 Hz, 1H), 4.5 (dd, *J* = 6.1, 1.7 Hz, 1H), 2.36 (s, 3H), ^{13}C NMR (125 MHz, $CDCl_3$), δ 157.0, 148.7, 137.9, 133.6, 132.2, 129.5, 129.2, 128.7, 128.2, 123.3, 123.2, 117.2, 114.8, 95.0, 94.3, 84.6, 21.2.

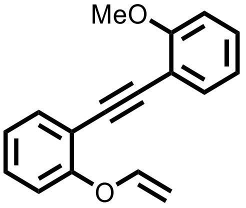

1-Methoxy-2-((2-(vinyloxy)phenyl)ethynyl)benzene (1g):[5] White solid, yield (245 mg, 98%), ^{1}H-NMR (500 MHz, $CDCl_3$), δ 7.57 (dd, *J* = 7.7, 1.6 Hz, 1H), 7.53 (dd, *J* = 7.7, 1.7 Hz, 1H), 7.31-7.26 (m, 2H), 7.07 (td, *J* = 7.6, 1.0 Hz, 1H), 7.0 (d, *J* = 8.2 Hz, 1H), 6.94 (td, *J* = 7.6, 0.71 Hz, 1H), 6.88 (d, *J* = 8.3 Hz, 1H), 6.71 (dd, *J* = 13.9, 6.0 Hz, 1H), 4.82 (dd, *J* =13.8, 1.7 Hz, 1H), 4.47 (dd, *J* = 6.0, 1.7 Hz, 1H), 3.90 (s, 3H), ^{13}C NMR (125 MHz, $CDCl_3$), δ 160.0, 157.0, 148.7, 133.5 (d, *J* = 4.6 Hz), 129.8 129.5, 123.2, 120.5, 117.0, 114.0, 114.9, 112.6, 110.9, 96.0, 90.6, 89.0, 55.9.

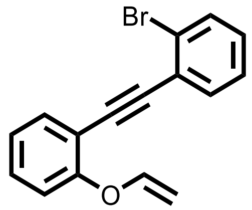

1-Bromo-2-((2-(vinyloxy)phenyl)ethynyl)benzene (1h): Brown viscous liquid, yield (266 mg, 89%), ^{1}H-NMR (400 MHz, $CDCl_3$), δ 7.61-7.55 (m, 3H), 7.32 (td, *J* = 7.9, 1.6 Hz, 1H), 7.27 (td, *J* = 7.6, 1.1, Hz, 1H), 7.16 (td, *J* = 7.8, 1.6 Hz, 1H), 7.08 (td, *J* = 7.5, 0.9 Hz, 1H), 7.03 (d, *J* = 8.3, Hz, 1H), 6.7 (dd, *J* = 13.8, 6.1 Hz, 1H), 4.79 (dd, *J* = 13.8, 1.7 Hz, 1H), 4.47 (dd, *J* = 6.2, 1.7 Hz, 1H), ^{13}C NMR (125 MHz, $CDCl_3$), δ 157.1, 148.4, 133.7, 133.3, 132.4, 130.0, 129.4, 126.9, 125.5,

123.2, 117.0, 114.2, 96.2, 92.5, 89.6, HRMS (ESI), *m/z* calcd for $C_{16}H_{11}BrO$ [M+H]$^+$ 299.0066, found 299.0036.

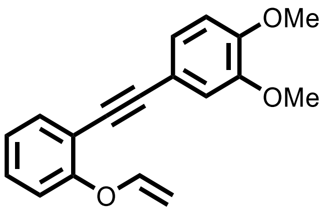

1,2-Dimethoxy-4-((2-(vinyloxy)phenyl)ethynyl)benzene (1i): yellow viscous liquid, yield (278 m g, 99%), ^{1}H-NMR (400MHz, $CDCl_3$), δ 7.50 (dd, *J* = 7.7, 1.6 Hz, 1H), 7.25 (td, *J* = 7.7, 1.6 Hz, 1H), 7.13 (dd, *J* = 8.3, 1.8 Hz, 1H), 7.06–7.03 (m, 2H), 6.99 (d, *J* = 8.3 Hz, 1H), 6.80 (d, *J* = 8.3 Hz, 1H), 6.66 (dd, *J* = 13.8, 6.0 Hz, 1H), 4.78 (dd, *J* = 13.8, 1.7 Hz, 1H), 4.45 (dd, *J* = 6.0, 1.7 Hz 1H), 3.86 (d, *J* = 2.9 Hz, 6H), ^{13}C NMR (100 MHz, $CDCl_3$), δ 156.9, 149.5, 148.7, 148.6, 133.4, 129.3, 124.9, 123.3, 117.2, 115.5, 114.8, 114.3, 111.0, 96.0, 94.3, 64.3, 83.6, 55.9, HRMS (ESI), *m/z* calcd for $C_{18}H_{16}O_3$ [M+Na]$^+$ 303.0992, found 303.0994.

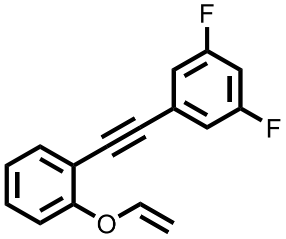

1,3-Difluoro-5-((2-(vinyloxy)phenyl)ethynyl)benzene (1j): Light yellow viscous, yield (244 mg, 95%), ^{1}H-NMR (400 MHz, $CDCl_3$), δ 7.51 (dd, *J* = 7.7, 1.5 Hz, 1H), 7.33 (td, *J* = 7.9, 1.6 Hz, 1H), 7.09 (dd, *J* = 7.7, 0.9 Hz, 1H), 7.07–7.02 (m, 3H), 6.79 (tt, *J* = 9.0, 2.3 Hz, 1H), 6.66 (dd, *J* = 13.8, 6.1 Hz, 1H), 4.80 (dd, *J* = 13.8, 1.7 Hz, 1H), 4.49 (dd, *J* = 6.1, 1.8 Hz, 1H), ^{13}C NMR (100 MHz, $CDCl_3$), δ 163.9 (d, *J* = 13.4 Hz), 161.4 (d, *J* = 13.3 Hz), 157.2, 148.3, 133.6, 130.3, 126.0 (t, *J* = 11.7 Hz), 123.3, 117.1, 114.6, 114.3, 113.6, 104.4 (t, *J* = 25.4 Hz), 96.5, 91.6 (t, *J* = 3.94 Hz), 87.0, HRMS (APCI), *m/z* calcd for $C_{16}H_{10}F_2O$ [M+H]$^+$ 257.0772, found 257.0761.

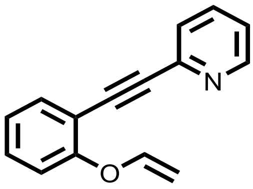

2-((2-(Vinyloxy)phenyl)ethynyl)pyridine (1l): Black viscous liquid, yield (215 mg, 97%), ^{1}H-NMR (400 MHz, $CDCl_3$), δ 8.59 (d, *J* = 4.8 Hz, 1H), 7.64 (td, *J* = 7.7 Hz, 1H), 7.57 (dd, *J* = 7.5, 1.6 Hz, 1H), 7.52 (d, *J* = 7.8, Hz, 1H), 7.3 (td, *J* = 7.9, 1.7, Hz, 1H), 7.22–7.18 (m, 1H), 7.06 (td, *J* = 7.5, 0.9 Hz, 1H), 7.00 (d, *J* = 8.3 Hz, 1H), 6.65 (dd, *J* = 13.8, 6.0 Hz, 1H), 4.79 (dd, *J* = 13.8, 1.8 Hz, 1H), 4.47 (dd, *J* = 6.0, 1.8 Hz, 1H),^{13}C NMR (100 MHz, $CDCl_3$), δ 157.4, 150.0, 148.3, 143.5, 136.0, 134.0, 130.3, 127.3, 123.2, 122.7, 116.9, 113.5, 95.6, 93.1, 84.9, HRMS (ESI), *m/z* calcd for $C_{15}H_{11}NO$ $[M+H]^+$ 222.0913, found 222.0904.

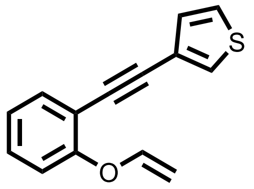

3-((2-(Vinyloxy)phenyl)ethynyl)thiophene (1m):[7] White viscous liquid, yield (266 mg, 80%), ^{1}H-NMR (400 MHz, $CDCl_3$), δ 7.55–7.52 (m, 2H), 7.33–7.28 (m, 2H), 7.23 (dd, *J* = 5.0 Hz, 1H), 7.08 (td, *J* = 7.5, 0.9 Hz, 1H), 7.03 (d, *J* = 8.1 Hz, 1H), 6.69 (dd, *J* = 13.7, 6.0 Hz, 1H), 4.82 (dd, *J* = 13.8, 1.7 Hz, 1H), 4.49 (dd, *J* = 6.0, 1.7 Hz, 1H), ^{13}C NMR (100 MHz, $CDCl_3$), δ 157.0, 148.6, 133.5, 129.9, 129.6, 128.7, 125.3, 123.3, 122.4, 117.1, 114.5, 96.2, 89.2, 84.5.

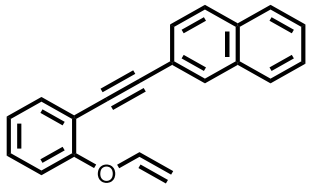

2-((2-(Vinyloxy)phenyl)ethynyl)naphthalene (1n): White viscous liquid, yield (201 mg, 74%), ^{1}H NMR (400 MHz, $CDCl_3$), δ 8.10 (s, 1H), 7.85–7.81 (m, 3H), 7.65 – 7.60 (m, 2H), 7.52–7.49 (m, 2H), 7.33 (td, *J* = 7.9, 1.7 Hz, 1H), 7.12 (td, *J* = 7.5, 0.8 Hz, 1H), 7.07 (d, *J* = 8.2 Hz, 1H), 6.75 (dd, *J* =13.7, 6.1 Hz, 1H), 4.88 (dd, *J* = 13.7, 1.7 Hz, 1H), 4.54 (dd, *J* = 6.1, 1.7 Hz, 1H), ^{13}C NMR (100 MHz, $CDCl_3$), δ 157.1, 148.7, 133.7, 133.0, 132.9, 131.5, 129.7, 128.5, 128.0, 127.8, 126.7, 126.5, 123.4, 120.7, 117.2, 114.7, 96.2, 94.6, 85.4, HRMS (APCI), *m/z* calcd for $C_{20}H_{14}O$ $[M+H]^+$ 271.1117, found 271.1118.

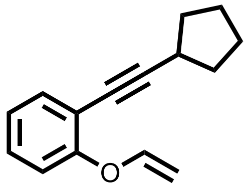

1-(Cyclopentylethynyl)-2-(vinyloxy)benzene (1o): White viscous liquid, yield (208 mg, 98%), ^{1}H-NMR (400 MHz, $CDCl_3$), δ 7.40-7.37 (m, 1H), 7.24-7.19 (m, 1H), 7.03-6.94 (m, 2H), .6.67-6.58 (m, 1H), 4.70 (d, *J* = 14.0 Hz, 1H), 4.42-4.37 (m, 1H), 2.91-2.82 (m, 1H), 2.02-1.95 (m, 2H), 1.79-1.73 (m, 3H), 1.64-1.55 (m, 2H), ^{13}C NMR (100 MHz, $CDCl_3$) δ 156.9, 148.9, 133.5, 133.8, 128.6, 123.2, 117.3, 115.5, 99.7, 94.3, 75.4, 33.8, 31.0, 25.0, HRMS (ESI), *m/z* calcd for $C_{15}H_{16}O$ $[M+H]^+$ 213.1274, found 213.1256.

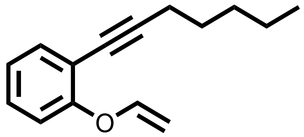

1-(Hept-1-yn-1-yl)-2-(vinyloxy)benzene (1p):[7] Light yellow viscous liquid, yield (262 mg, 94%), ^{1}H-NMR (400 MHz, $CDCl_3$), δ 7.39 (dd, *J* = 1.4, 7.7 Hz, 1H), 7.22 (td, *J* = 7.9, 1.6 Hz, 1H), 7.00 (t, *J* = 7.6 Hz, 1H), 6.95 (d, *J* = 8.2 Hz, 1H), 6.62 (dd, *J* = 13.8, 6.1 Hz, 1H), 4.7 (dd, *J* = 13.8, 1.6 Hz, 1H), 4.4 (dd, *J* = 6.1, 1.6 Hz, 1H), 2.48 (t, *J* = 7.0, Hz, 2H), 1.65-1.57 (m, 2H), 1.48-1.41 (m, 2H), 1.38-1.31 (m, 2H), 0.91 ((t, *J* = 7.2 Hz, 3H), ^{13}C NMR (100 MHz, $CDCl_3$), δ 156.9, 148.7, 133.6, 128.7, 123.2, 117.1, 115.4, 96.5, 94.6, 75.9, 31.0, 28.3, 22.2, 19.6, 14.0.

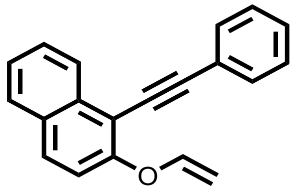

1-(Phenylethynyl)-2-(vinyloxy)naphthalene (1q):[4] Yellow viscous liquid, yield (258 mg, 73%), ^{1}H-NMR (400 MHz, $CDCl_3$), δ 8.46 (d, *J* = 8.4 Hz, 1H), 7.83 (t, *J* = 8.6 Hz, 2H), 7.71 (d, *J* = 7.2 Hz, 2H), 7.64 (t, *J* = 8.6 Hz, 1H), 7.49 (t, *J* = 7.5, Hz, 1H), 7.44-7.38 (m, 3H), 7.30 (d, *J* = 8.9 Hz, 1H), 6.80 (dd, *J* = 13.8, 6.1 Hz, 1H), 4.84 (d, *J* = 13.8 Hz, 1H), 4.53 (d, *J* = 6.0 Hz, 1H), ^{13}C NMR (100 MHz, $CDCl_3$), δ 155.6, 149.4, 134.3, 131.6, 130.1, 128.5, 128.4, 128.2, 127.5, 125.8, 125.4, 123.6, 118.3, 109.9, 99.6, 94.5, 83.2.

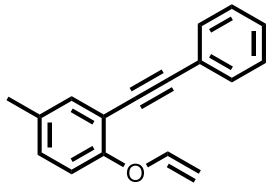

4-Methyl-2-(phenylethynyl)-1-(vinyloxy)benzene (1r):[5] yellow viscous liquid, yield (417 mg, 64%), ^{1}H-NMR (400 MHz, $CDCl_3$), δ 7.62-7.59 (m, 2H), 7.39-7.35 (m, 4H), 7.12 (dd, *J* = 8.4, 1.8 Hz, 1H), 6.96 (d, *J* = 8.3 Hz, 1H), 6.71 (dd, *J* = 13.8, 6.0 Hz, 1H), 4.80 (dd, *J* = 13.8, 1.7 Hz, 1H), 4.47 (dd, *J* = 6.1, 1.7, 1H), 2.34 (s, 3H), ^{13}C NMR (100 MHz, $CDCl_3$), δ 154.9, 149.2, 133.9, 133.0, 131.6, 130.4, 128.4, 128.3, 123.5, 117.5, 114.5, 94.3, 93.8, 85.3, 20.5.

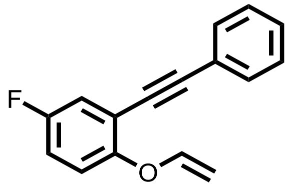

4-Fluoro-2-(phenylethynyl)-1-(vinyloxy)benzene (1s): White viscous liquid, yield (322 mg, 66%), ^{1}H-NMR (500 MHz, $CDCl_3$), 7.55-7.53 (m, 2H), 7.37-7.33 (m, 3H), 7.22 (dd, *J* = 8.7, 2.3 1H), 7.02-6.97 (m, 2H), 6.63 (dd, *J* = 13.7, 6.1 Hz, 1H), 4.71 (dd, *J* = 13.7, 1.8 Hz, 1H), 4.44 (dd, *J* = 6.2, 1.9 Hz, 1H), ^{13}C NMR (125 MHz, $CDCl_3$), δ 159.5, 157.0, 153.1 (d, *J* = 2.6 Hz), 149.2, 131.7, 128.6, 128.3, 122.8, 119.6 (d, *J* = 24.5 Hz), 119.2 (d, *J* = 8.8 Hz), 116.4-116.2 (m), 95.0, 94.5, 83.9 (d, *J* = 2.9 Hz), HRMS (ESI), *m/z* calcd for $C_{16}H_{11}FO$ $[M+H]^+$ 239.0867, found 239.0875.

Scheme S10. Preparation of (2-(Phenylethynyl)phenyl)(vinyl)sulfane (**3a**)

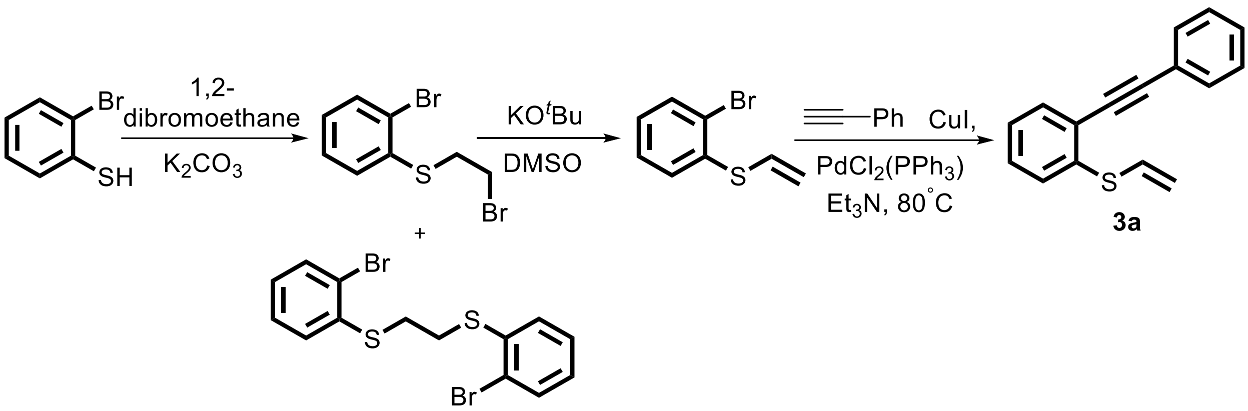

Preparation of (2-Bromoethyl)(2-bromophenyl)sulfane from 2-Bromothiophenol

To a stirred solution of 2-bromothiophenol (5.29 mmol, 1.00 g) and 1, 2-dibromoethane (26.45 mmol, 2.3 mL) in acetone (50 mL) was added K_2CO_3 (10.58 mmol, 1.46 g). The resulting mixture was stirrer at room temperature for overnight. The reaction was quenched with water (10 mL) and extracted with CH_2Cl_2 (20 mL x 3). The organic layer was washed with brine (50 mL), dried over Na_2SO_4 and concentrate by rotary evaporator under vacuum. The crude product was purified by column chromatography on silica gel using hexane. A white solid was obtained as major product and 1,2-bis ((2-bromophenyl)thio)ethane was minor. (2-Bromoethyl)(2-bromophenyl)sulfane (SJ-458-P-1) Yield (1.24 g, 79%), ^{1}H-NMR (500 MHz, $CDCl_3$), δ 7.61 (dd, J = 8.0, 1.3 Hz, 1H), 7.37 (dd, J = 7.9, 1.6 Hz, 1H), 7.32 (td, J = 7.5, 1.4 Hz, 1H), 7.12 (td, J = 7.5, 1.6 Hz, 1H), 3.54-3.50 (m, 2H), 3.39-3.36 (m, 2H) ^{13}C NMR (100 MHz, $CDCl_3$), δ 135.4, 133.5, 129.8, 128.0, 127.9, 125.2, 35.1, 29.2.

1,2-Bis ((2-bromophenyl)thio)ethane (SJ-458-P-2) White solid, Yield (236 mg, 10%), ^{1}H-NMR (500 MHz, $CDCl_3$), δ 7.60 (d, J = 8.0 Hz, 2H), 7.29-7.27 (m, 4H), 7.12-7.08 (m, 2H), 31.9 (s, 4H), ^{13}C NMR (100 MHz, $CDCl_3$), δ 136.2, 133.3, 129.3, 127.9, 127.5, 124.8, 32.1.

Preparation of (2-Bromophenyl)(vinyl)sulfane from (2-Bromoethyl)(2-bromophenyl)sulfane

A solution of (2-bromoethyl)(2-bromophenyl)sulfane (3.89 mmol, 1.15 g) in DMSO (10 mL) was stirred at 0 °C. To this stirrer solution was added KO^{t}Bu (5.06 mmol, 0.568 g) in portions under nitrogen. The resulting mixture was stirrer at room temperature for 2h. The reaction was quenched with water (100 mL) and extracted with CH_2Cl_2 (50 mL x 4). The organic layer was washed with brine (100 mL) dried over Na_2SO_4 and concentrate by rotary evaporator under vacuum. The crude

product was purified by column chromatography on silica gel using hexane. A yellow oil of 2-Bromophenyl)(vinyl)sulfane was obtained. Yield (560 mg, 67%), ^{1}H-NMR (400 MHz, $CDCl_3$), δ 7.55 (dd, J = 8.0, 1.0 Hz, 1H), 7.33 (dd, J = 8.0, 1.6 Hz, 1H), 7.27 (td, J = 7.5, 1.2 Hz, 1H), 7.07 (td, J = 7.6, 1.7 Hz, 1H), 6.50 (dd, J = 17.5, 9.5 Hz, 1H), 5.54-5.49 (m, 2H), ^{13}C NMR (100 MHz, $CDCl_3$), δ 136.4, 133.1, 129.9, 129.7, 127.9, 127.7, 123.7, 119.0. HRMS (ESI), *m/z* calcd for C_8H_7BrS $[M+H]^+$ 216.9504, found 216.9509.

Preparation of (2-(Phenylethynyl)phenyl)(vinyl)sulfane from(2-Bromophenyl)(vinyl)sulfane

1-(Phenylethynyl)-2-(vinyloxy) benzene (**3a-3m**) substrates were prepared by the literature procedure.[8]

To a solution of (2-bromophenyl)(vinyl)sulfane (1.5 mmol, 323 mg) and phenyl acetylene (2.25 mmol, 230 mg) in trimethylamine (degassed, 8 mL) was added $PdCl_2(PPh_3)$ (0.045 mmol, 32 mg,) and CuI (0.09 mmol, 18 mg) under nitrogen. The resulting mixture was stirrer at 80℃ for 5 h, progress of reaction was monitored by TLC. After completion, reaction allowed to cool to room temperature. The reaction mixture was filtered and washed with diethyl ether. The combined filtrate was concentrate and the residue was purified by column chromatography on silica gel using hexane. A yellow oil (2-(Phenylethynyl)phenyl)(vinyl)sulfane **3a** was obtained. Yield (250 mg, 70%), ^{1}H NMR (400 MHz, $CDCl_3$), δ 7.59-7.57 (m, 1H), 7.56 (d, J = 1.9 Hz, 1H), 7.53 (dd, J = 7.6, 1.2 Hz, 1H), 7.38-7.33 (m, 4H), 7.29 (td, J = 7.6, 1.4 Hz, 1H), 7.20 (td, J = 7.5, 1.2 Hz, 1H), 6.61 (dd, J = 17.0, 9.4 Hz, 1H), 5.55 (d, J = 16.7 Hz, 1H), 5.50 (d, J = 9.5 Hz, 1H), ^{13}C NMR (100 MHz, $CDCl_3$), δ 138.0, 132.6, 131.6, 130.3, 128.7, 128.6, 128.5, 128.3, 126.2, 123.3, 123.1, 118.1, 95.6, 87.0, HRMS (ESI), *m/z* calcd for $C_{16}H_{12}S$ $[M+H]^+$ 237.0732, found 237.0733.

By using different analogues of substituted phenyl acetylene, substrates **3b**, **3c**, **3d**, **3e**, **3f**, **3g**, **3h**, **3i**, **3j**, **3k**, **3l** and **3m** were synthesized. Yields and analytical data for these substrates presented below.

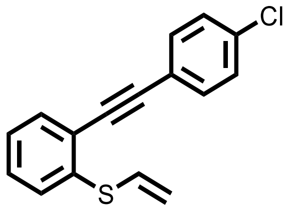

(2-((4-Chlorophenyl)ethynyl)phenyl)(vinyl)sulfane (3b): Light brown liquid, yield (253 mg, 62%), ^{1}H-NMR (400 MHz, DMSO-d_6), δ 7.56-7.51 (m, 3H), 7.48-7.44 (m, 2H), 7.40-7.36 (m, 2H), 7.29-7.25 (m, 1H), 6.74 (dd, *J* = 17.4, 9.5 Hz, 1H), 5.55 (d, *J* = 8.0 Hz, 1H), 15.08 (d, *J* = 15.1 Hz, 1H), ^{13}C NMR (100 MHz, $CDCl_3$), δ 137.9, 134.2, 133.4, 133.0, 130.2, 129.8, 129.4, 128.4, 127.0, 121.7, 121.3, 119.3, 94.5, 88.2, HRMS (APCI), *m/z* calcd for $C_{16}H_{11}ClS$ $[M+H]^+$ 271.0343, found 271.0349.

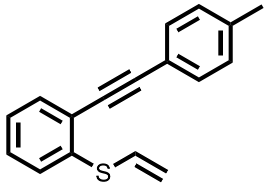

(2-(*p*-Tolylethynyl)phenyl)(vinyl)sulfane (3c): Light viscous liquid, yield (287 mg, 76%), ^{1}H-NMR (400 MHz, $CDCl_3$), δ 7.52 (dd, *J* = 7.6, 1.2 Hz, 1H), 7.47 (d, *J* = 8.0 Hz, 2H), 7.36 (d, *J* = 8.0 Hz, 1H), 7.28 (td, *J* = 7.6, 1.4 Hz, 1H), 7.21 (dd, *J* = 7.6, 1.2 Hz, 1H), 7.16 (d, *J* = 7.9 Hz, 2H), 6.61 (dd, *J* = 17.6, 9.4 Hz, 1H), 5.56 (d, *J* = 16.6, Hz, 1H), 5.50 (d, *J* = 9.4 Hz, 1H), 2.37 (s, 3H), ^{13}C NMR (100 MHz, $CDCl_3$), δ 138.7, 137.9, 132.5, 134.1, 131.5, 130.4, 129.1, 128.6, 128.5, 126.2, 123.5, 120.0, 118.0, 96.9, 86.4, 21.5, HRMS (APCI), *m/z* calcd for $C_{17}H_{14}S$ $[M+H]^+$ 251.0889, found 251.0884.

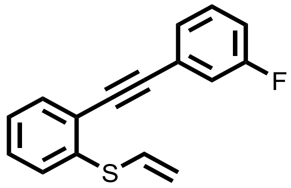

(2-((3-Fluorophenyl)ethynyl)phenyl)(vinyl)sulfane (3d): Light yellow viscous liquid, yield (267 mg, 70%), ^{1}H-NMR (400 MHz, $CDCl_3$), δ 7.52 (dd, *J* = 7.6, 1.2 Hz, 1H), 7.37-7.25 (m, 5H), 7.20 (td, *J* = 7.5, 1.4 Hz, 1H), 7.06-7.01 (m, 1H), 6.59 (dd, *J* = 17.0, 9.6 Hz, 1H), 5.54 (d, *J* = 16.7 Hz, 1H), 5.50 (d, *J* = 9.6 Hz, 1H), ^{13}C NMR (100 MHz, $CDCl_3$), δ 162.4 (d, *J* = 246.1 Hz), 138.2, 132.7, 130.2, 129.8 (d, *J* = 8.5 Hz), 129.1, 128.7, 127.5 (d, *J* = 3.1 Hz), 126.3, 124.9 (d, *J* = 9.6 Hz), 122.8, 118.4, 118.2, 115.8 (d, *J* = 21.4 Hz), 94.1 (d, *J* = 3.4 Hz), 87.8, HRMS (APCI), *m/z* calcd for $C_{16}H_{11}FS$ $[M+H]^+$ 255.0638, found 255.0647.

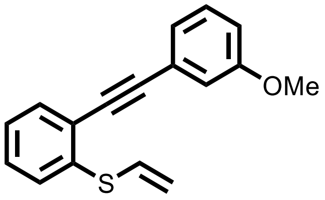

(2-((3-Methoxyphenyl)ethynyl)phenyl)(vinyl)sulfane (3e): Light viscous liquid, yield (350 mg, 87%), ^{1}H-NMR (500 MHz, $CDCl_3$), δ 7.54 (dd, *J* = 7.7, 1.2 Hz, 1H), 7.37 (dd, *J* = 7.9, 0.9 Hz, 1H), 7.31-7.27 (m, 2H), 7.23-7.18 (m, 2H), 7.13-7.11 (m, 1H), 6.91 (ddd, *J* =8.2, 2.6, 0.9 Hz, 1H), 6.62 (dd, *J* = 16.7, 9.6 Hz, 1H), 5.57 (d, *J* = 16.7 Hz, 1H), 5.52 (d, *J* = 9.6 Hz, 1H), 3.82 (s, 3H), ^{13}C NMR (100 MHz, $CDCl_3$), δ 159.4, 138.1, 132.6, 130.4, 129.4, 128.9, 128.6, 126.3, 124.2, 124.1, 123.2, 118.1, 116.4, 115.1, 96.5, 86.8, 55.3, HRMS (ESI), *m/z* calcd for $C_{17}H_{14}OS$ $[M+H]^+$ 267.0838, found 267.0817.

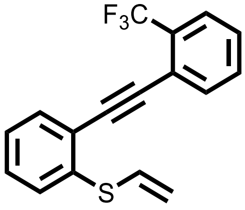

(2-((2-(Trifluoromethyl)phenyl)ethynyl)phenyl)(vinyl)sulfane (3f): Light yellow viscous liquid, yield (264 mg, 58%), ^{1}H-NMR (400 MHz, $CDCl_3$), δ 7.73 (d *J* = 7.7 Hz, 1H), 7.68 (d, *J* = 7.8 Hz, 1H), 7.56 (dd, *J* = 7.7, 1.2 Hz, 1H), 7.52 (t, *J* = 7.7, 1H), 7.41 (t, *J* = 7.8 H, 1H), 7.37 (dd, *J* =7.8, 1.0 Hz, 1H), 7.31 (td, *J* = 7.8, 1.4 Hz, 1H), 7.22 (td, *J* = 7.4, 1.3 Hz, 1H), 6.60 (dd, *J* = 17.3, 9.5 Hz, 1H), 5.54 (d, *J* = 16.7 Hz, 1H), 5.50 (d, *J* = 9.5 Hz, 1H), ^{13}C NMR (100 MHz, $CDCl_3$), δ 138.1, 134.1, 133.1, 131.4, 130.3, 129.3, 128.7, 128.1, 126.3, 125.9 (q, *J* = 5.1 Hz), 124.9, 122.9, 122.2, 121.3 (q, *J* = 2.9 Hz), 138.1, 92.4, 91.1, HRMS (ESI), *m/z* calcd for $C_{17}H_{11}F_3S$ $[M+H]^+$ 305.0606, found 305.0578.

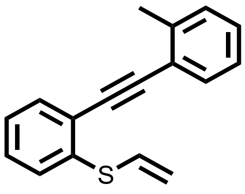

(2-(*o*-Tolylethynyl)phenyl)(vinyl)sulfane (3g): Light yellow viscous liquid, yield (248 mg, 66%), ^{1}H-NMR (400 MHz, $CDCl_3$), δ 7.55-7.52 (m, 2H), 7.36 (d, *J* = 7.9 Hz, 1H), 7.28 (td, *J* = 7.6, 1.4 Hz, 1H), 7.24-7.20 (m, 3H), 7.19-7.14 (m, 1H), 6.60 (dd, *J* = 16.8, 9.6 Hz, 1H), 5.52 (d, *J*

= 15.7 Hz, 1H), 5.49 (d, *J* = 8.6 Hz, 1H), 2.56 (s, 3H), ^{13}C NMR (100 MHz, $CDCl_3$), δ 140.4, 137.7, 132.7, 132.0, 130.3, 129.5, 128.7, 128.5, 126.2, 125.5, 123.6, 122.8, 118.1, 94.5, 90.7, 20.9, HRMS (ESI), *m/z* calcd for $C_{17}H_{14}S$ $[M+H]^+$ 250.0889, found 250.0890.

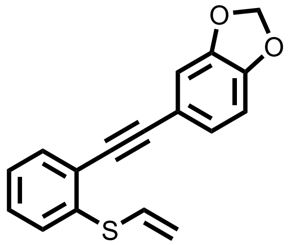

5-((2-(Vinylthio)phenyl)ethynyl)benzo[*d*][1,3]dioxole (3h): White solid, yield (201 mg, 50%), ^{1}H-NMR (400 MHz, $CDCl_3$), δ 7.49 (dd, *J* = 7.6, 1.2 Hz, 1H), 7.34 (d, *J* = 7.9 Hz, 1H), 7.26 (td, *J* = 7.7, 1.1 Hz, 1H), 7.18 (td, *J* = 7.5, 1.1 Hz, 1H), 7.10 (dd, *J* = 8.0, 1.4 Hz, 1H), 7.01 (dd, *J* = 1.4 Hz, 1H), 6.78 (dd, *J* = 8.0 Hz, 1H), 6.59 (dd, *J* = 16.9, 9.5 Hz, 1H), 5.97 (s, 2H), 5.53 (d, *J* = 16.7 Hz, 1H), 5..49 (d, *J* = 9.5 Hz, 1H), ^{13}C NMR (100 MHz, $CDCl_3$), δ 148.1, 147.4, 137.8, 137.4, 130.4, 128.6, 128.5, 126.4, 126.3, 123.4, 118.0, 116.3, 111.5, 108.5, 101.3, 96.6, 85.5, HRMS (ESI), *m/z* calcd for $C_{17}H_{12}O_2S$ $[M+H]^+$ 281.0631, found 281.0625.

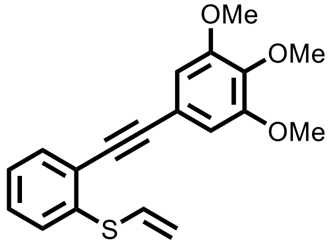

(2-((3,4,5-Trimethoxyphenyl)ethynyl)phenyl)(vinyl)sulfane (3i): Light brown viscous liquid, yield (268 mg, 54%), ^{1}H-NMR (400 MHz, $CDCl_3$), δ 7.51(dd, *J* = 7.7, 1.2 Hz, 1H), 7.34 (dd, *J* = 7.8, 0.8 Hz, 1H), 7.27 (td, *J* = 7.8, 1.4 Hz, 1H), 7.19 (td, *J* = 7.5, 1.9 Hz, 1H), 6.79 (s, 2H), 6.60 (dd, *J* = 17.5, 9.5 Hz, 1H), 5.54 (d, *J* = 16.6 Hz, 1H), 5.49 (d, *J* = 9.5 Hz, 1H), 3.86 (s, 6H), 3.85 (s, 3H), ^{13}C NMR (100 MHz, $CDCl_3$), δ 153.1, 139.1, 137.9, 130.5, 130.4, 128.7, 128.5, 126.2, 123.1, 118.1, 118.1, 108.0, 96.6, 86.0, 60.9, 56.2, HRMS (ESI), *m/z* calcd for $C_{19}H_{18}O_3S$ $[M+H]^+$ 327.1049, found 327.1057.

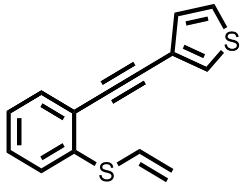

3-((2-(Vinylthio)phenyl)ethynyl)thiophene (3j): Light yellow viscous liquid, yield (258 mg, 71%), ^{1}H-NMR (400 MHz, $CDCl_3$), δ 7.56 (dd, *J* = 3.0, 1.0 Hz, 1H), 7.51 (dd, *J* = 7.7, 1.1 Hz, 1H), 7.35 (d, *J* = 8.0 Hz, 1H), 7.31-7.26 (m, 2H), 7.23-7.21 (m, 1H), 7.18 (dd, *J* = 7.4, 1.2 Hz, 1H), 6.60 (dd, *J* = 16.6 Hz, 9.5 Hz, 1H), 5.55 (d, *J* = 16.7 Hz, 1H), 5.50 (d, *J* = 9.5 Hz, 1H), ^{13}C NMR (100 MHz, $CDCl_3$), δ 137.9, 132.5, 130.4, 129.8, 128.9, 128.7, 128.6, 126.3, 125.4, 123.3, 122.1, 118.1, 90.7, 86.5, HRMS (ESI), *m/z* calcd for $C_{14}H_{10}S_2$ $[M+H]^+$ 243.0297, found 243.0278.

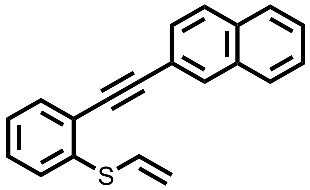

(2-(Naphthalen-2-ylethynyl)phenyl)(vinyl)sulfane (3k): White solid, yield (196 mg, 60%), ^{1}H-NMR (400 MHz, $CDCl_3$), δ 8.08 (s, 1H), 7.84-7.79 (m, 3H), 7.61 (dd, *J* = 8.5, 1.4 Hz, 1H), 7.57 (dd, *J* = 7.7, 1.2 Hz, 1H), 7.50-7.48 (m, 2H), 7.38 (d, *J* = 7.9 Hz, 1H), 7.30 (td, *J* = 7.7, 1.3 Hz, 1H), 7.22 (td, *J* = 7.5, 1.3 Hz, 1H), 6.63 (dd, *J* = 17.4, 9.5 Hz, 1H), 5.58 (d, *J* = 16.7 Hz, 1H), 5.52 (d, *J* = 9.5 Hz, 1H), ^{13}C NMR (100 MHz, $CDCl_3$), δ 138.1, 133.0, 132.9, 132.6, 131.5, 130.3, 128.8, 128.5, 128.3, 128.0, 127.8, 127.7, 126.7, 126.5, 126.3, 123.3, 120.4, 118.2, 96.0, 87.3, HRMS (APCI), *m/z* calcd for $C_{20}H_{14}S$ $[M+H]^+$ 287.0889, found 287.0887.

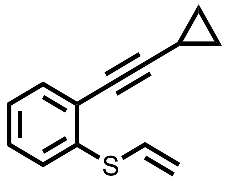

(2-(Cyclopentylethynyl)phenyl)(vinyl)sulfane (3l): Light yellow viscous liquid, yield (252 mg, 84%), ^{1}H-NMR (400 MHz, $CDCl_3$), δ 7.37 (dd, *J* = 7.7, 1.2 Hz, 1H), 7.27 (dd, *J* = 7.9, 1.0 Hz, 1H), 7.20 (td, *J* = 7.8, 1.3 Hz, 1H), 7.12 (td, *J* = 7.4, 1.1 Hz, 1H), 6.55 (dd, *J* = 16.8, 9.5 Hz, 1H), 5.51 (d, *J* = 16.7 Hz, 1H), 5.47 (d, *J* = 9.5 Hz, 1H), 1.53-1.47 (m, 1H), 0.91-0.85 (m, 4H), ^{13}C NMR (100 MHz, $CDCl_3$), δ 137.7, 132.5, 130.4, 128.1, 128.0, 126.0, 123.8, 117.9, 100.3, 73.3, 8.9, 0.4, HRMS (ESI), *m/z* calcd for $C_{13}H_{12}S$ $[M+H]^+$ 201.0732, found 201.0715.

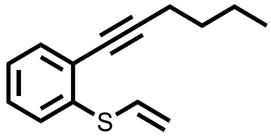

(2-(Hex-1-yn-1-yl)phenyl)(vinyl)sulfane (3m): Light yellow viscous liquid, yield (186 mg, 57%), ^{1}H-NMR (400 MHz, $CDCl_3$), δ 7.4 (dd, *J* = 7.7, 1.0 Hz, 1H), 7.28 (d, *J* = 7.8 Hz, 1H), 7.21 (td, *J* = 7.7 , 1.3 Hz, 1H), 7.12 (td, *J* = 7.5, 1.1 Hz, 1H), 6.56 (dd, *J* = 16.5, 9.5 Hz, 1H), 5.51 (d, *J* = 16.7 Hz, 1H), 5.47 (d, *J* = 9.6 Hz, 1H), 2.47 (t, *J* = 7.00 Hz, 2H), 1.65-1.58 (m, 2H), 1.55-1.46 (m, 2H), 0.95 (t, *J* = 7.2 Hz, 3H), ^{13}C NMR (100 MHz, $CDCl_3$), δ 137.6, 132.6, 130.4, 128.1, 128.0, 126.0, 123.9, 117.9, 97.2, 78.2, 30.7, 22.0, 19.3, 13.6, HRMS (ESI), *m/z* calcd for $C_{14}H_{16}S$ $[M+H]^+$ 255.0604, found 255.0599.

Scheme S11. Preparation of 4-Methyl-*N*-(2-(phenylethynyl)phenyl)-*N*-vinylbenzenesulfonamide

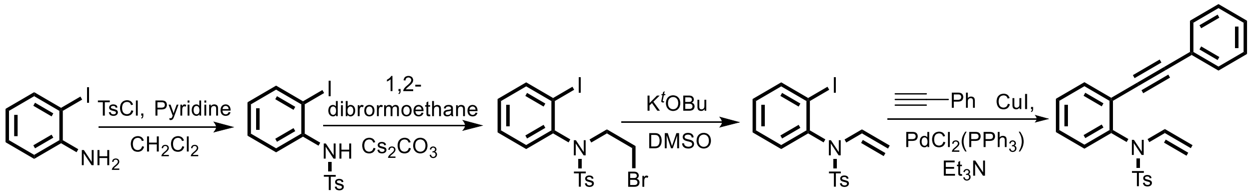

Typical Procedure

Preparation of *N*-(2-Iodophenyl)-4-methylbenzenesulfonamide from 2-Iodoaniline

To a stirrer solution of 2-iodoaniline (10 mmol, 2.19 g) and pyridine (20 mmol, 1.6 ml) in DCM (30 mL) was added TsCl (11 mmol, 2.01 g) at 0 °C. The reaction mixture was stirrer at 0 °C for 1h and then allowed to warm to room temperature and stirred overnight. The reaction mixture was diluted by 100 mL of CH_2Cl_2, washed with 1 M aqueous HCl (50 mL), saturated $NaHCO_3$ (50 mL) and brine (20 mL). The organic layer was separated, dried over Na_2SO_4 and concentrated under *vacuo*. The resulting mixture was purified by column chromatography on silica gel using hexane/EtOAc (95/5) eluent which resulted a white solid *N*-(2-Iodophenyl)-4-methylbenzenesulfonamide.[9] Yield (6.22 g, 83%), ^{1}H-NMR (500 MHz, $CDCl_3$), δ 7.68-7.65 (m, 4H), 7.32 (t, *J* = 7.8 Hz, 1H), 7.23 (d, *J* = 8.1 Hz, 2H), 6.86-6.83 (m, 2H), 2.40 (s, 3H), ^{13}C NMR (125 MHz, $CDCl_3$), δ 144.2, 139.1, 137.5, 135.9, 129.6, 129.5, 127.4, 126.8, 122.4, 21.6.

Preparation of *N*-(2-Bromoethyl)-N-(2-iodophenyl)-4-methylbenzenesulfonamide from *N*-(2-Iodophenyl)-4-methylbenzenesulfonamide

Substrate *N*-(2-Bromoethyl)-*N*-(2-iodophenyl)-4-methylbenzenesulfonamide was prepared by the following literature procedure.[9]

To a stirrer solution of (16.08 mmol, 6.0 g) and 1, 2-dibromoethane (160.8 mmol, 14 mL) in 30 mL acetonitrile was added Cs_2CO_3 (40.2 mmol, 13.01 g). The mixture was heated to reflux and stirred until the starting material was finished (monitored by TLC) and then allowed to cool to room temperature. Next, the reaction was quenched with water (50 mL) and extracted with CH_2Cl_2 (50 mL x 4). The organic layer was washed with brine (50 mL), dried over Na_2SO_4 and concentrate by rotary evaporator under vacuum. The crude product was purified by column chromatography on silica gel using hexane/EtOAc (95/5) eluent. A white solid was obtained.[11] Yield (5.93 g, 77%), ^{1}H-NMR (400 MHz, $CDCl_3$), δ 7.85 (d, *J* = 8.3 Hz, 1H), 7.56 (d, *J* = 8.2 Hz, 2H), 7.30-7.25 (m, 3H), 7.30-7.25 (m, 3H), 7.03-7.00 (m, 2H), 3.95-3.88 (m, 1H), 3.81-3.74 (m 1H), 3.53-3.47 (m, 1H), 3.44-3.36 (m, 1H), 2.39 (s 3H), ^{13}C NMR (100 MHz, $CDCl_3$), δ 144.1, 141.1, 140.6, 135.9, 131.1, 130.3, 129.7, 129.1, 128.0, 102.2, 53.2, 28.5, 21.6.

Preparation of *N*-(2-Iodophenyl)-4-methyl-N-vinylbenzenesulfonamide from *N*-(2-Bromoethyl)-N-(2-iodophenyl)-4-methylbenzenesulfonamide

Substrate *N*-(2-Iodophenyl)-4-methyl-N-vinylbenzenesulfonamide was prepared by the literature procedures.[10] The following representative procedures

A solution of *N*-(2-bromoethyl)-N-(2-iodophenyl)-4-methylbenzenesulfonamide (20.55 mmol, 9.87 g) in DMSO (25 mL) was stirred at 0 ^{0}C. To this stirrer solution was added KO^{t}Bu (20.55 mmol, 3.5 g) in portions under nitrogen. The resulting mixture was stirrer at room temperature for 2 h. The reaction was quenched with water (100 mL) and extracted with CH_2Cl_2 (50 mL x 4). The organic layer was washed with brine (50 mL), dried over Na_2SO_4 and concentrate by rotary evaporator under vacuum. The crude product was purified by column chromatography on silica gel using hexane/ EtOAc (95/5). A yellow oil was obtained.[11] Yield (5.58 g, 68%), ^{1}H-NMR (400 MHz, $CDCl_3$), δ 7.88 (d, *J* = 8.0 Hz, 1H), 7.58 (d, *J* = 8.3 Hz, 2H), 7.26-7.22 (m, 3H), 7.10 (dd, *J* = 15.3, 8.8 Hz, 1H), 7.04-6.99 (m, 1H), 4.25 (d, *J* = 8.8 Hz, 1H), 3.61 (d, *J* = 15.5 Hz, 1H), 2.36 (s, 3H), ^{13}C NMR (100 MHz, $CDCl_3$), δ 144.4, 140.8, 138.5, 136.2, 133.1, 130.7, 130.5, 129.9, 129.3, 127.6, 102.1, 94.9, 21.7.

Preparation of 4-Methyl-*N*-(2-(phenylethynyl)phenyl)-*N*-vinylbenzenesulfonamide from *N*-(2-Iodophenyl)-4-methyl-N-vinylbenzenesulfonamide

To a solution of *N*-(2-iodophenyl)-4-methyl-N-vinylbenzenesulfonamide (1.0 mmol, 399 mg) and phenyl acetylene (1.5 mmol, 0.16 mL) in trimethylamine (degassed, 5 mL) was added $PdCl_2(PPh_3)$ (0.03 mmol, 21 mg) and CuI (0.06 mmol, 12 mg) under nitrogen. The resulting mixture was stirrer at room temperature for 6 h. The reaction mixture was filtered and washed with diethyl ether (10 mL x 3). The combined filtrate was concentrate and the residue was purified by column chromatography on silica gel using hexane/NEt_3 (99/1). 4-Methyl-*N*-(2-(phenylethynyl)phenyl)-*N*-vinylbenzenesulfonamide (**5a**) was obtained as a yellow oil. Yield (350 mg, 93%), ^{1}H-NMR (400 MHz, $CDCl_3$), δ 7.61 (d, *J* = 8.3 Hz, 3H), 7.38-7.34 (m, 2H), 7.31-7.28 (m, 5H), 7.21-7.15 (m, 2H), 7.09 (d, *J* = 8.1 Hz, 2H), 4.31 (d, *J* = 8.8 Hz, 1H), 3.83 (d, *J* = 15.5 Hz, 1H), 2.22 (s 3H), ^{13}C NMR (100 MHz, $CDCl_3$), δ 143.8, 140.7, 136.8, 136.6, 133.4, 133.2, 131.6, 131.5, 129.7, 129.6, 128.4, 128.1, 127.5, 124.8, 122.9, 94.2, 93.9, 85.8, 21.4, HRMS (ESI), *m/z* calcd for $C_{23}H_{19}NO_2S$ $[M+H]^+$ 374.1209, found 374.1207.

By using different analogues of substituted phenyl acetylene, various substrates (**5b**, **5c**. **5d**, **5e**, **5f** and **5g**) were synthesized. Yields and analytical data for these substrates presented below.

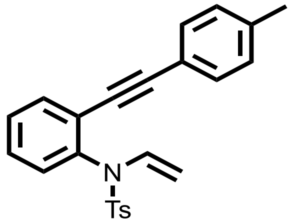

4-Methyl-N-(2-(p-tolylethynyl)phenyl)-N-vinylbenzenesulfonamide (5b): Light yellow solid, yield (355 mg, 91%), ^{1}H-NMR (400 MHz, $CDCl_3$), δ 7.60 (d, *J* = 8.3 Hz, 2H), 7.54-7.52 (m, 1H), 7.36-7.32 (m, 2H), 7.29 (t, *J* = 8.3 Hz, 1H), 7.20 (d, *J* = 8.0 Hz, 2H), 7.17-7.14 (m, 1H), 7.11-7.08 (m, 4H), 4.29 (d, *J* = 8.9 Hz, 1H), 3.82 (d, *J* = 15.5 Hz, 1H), 2.34 (s, 3H), 2.23 (s, 3H), ^{13}C NMR (100 MHz, $CDCl_3$), δ 143.7, 140.7, 138.6, 136.7, 136.6, 133.4, 133.2, 131.5, 129.6, 129.0, 128.8, 127.5, 125.0, 119.9, 94.5, 93.8, 85.2, 21.5, 21.4, HRMS (ESI), *m/z* calcd for $C_{24}H_{21}NO_2S$ $[M+H]^+$ 388.1366, found 388.1394.

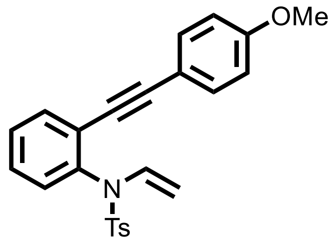

N-(2-((4-Methoxyphenyl)ethynyl)phenyl)-4-methyl-N-vinylbenzenesulfonamide (5c): Brown viscous liquid, yield (365 mg, 90%), ^{1}H-NMR (400 MHz, $CDCl_3$), δ 7.61 (d, *J* = 8.2 Hz, 2H), 7.52 (dd, *J* = 7.2, 2.0 Hz, 1H), 7.37-7.30 (m, 2H), 7.25 (d, *J* = 5.6 Hz, 2H), 7.19-7.10 (m, 4H), 6.82 (d, *J* = 8.8 Hz, 2H), 4.29 (d, *J* = 8.9 Hz, 1H), 3.84-3.80 (m, 4H), 2.25 (s, 3H), ^{13}C NMR (100 MHz, $CDCl_3$), δ 159.7, 143.7, 136.7, 136.6, 133.2, 133.1, 131.4, 129.5, 129.0, 128.7, 127.5, 125.2, 115.1, 113.7, 94.4, 93.8, 84.6, 55.3, 21.4, HRMS (ESI), *m/z* calcd for $C_{24}H_{21}NO_3S$ $[M+H]^+$ 404.1315, found 404.1303.

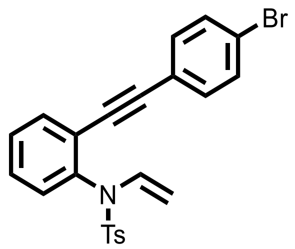

***N*-(2-((4-Bromophenyl)ethynyl)phenyl)-4-methyl-N-vinylbenzenesulfonamide (5d):** Yellow solid, yield (414 mg, 91%), ^{1}H-NMR (400 MHz, $CDCl_3$), δ 7.64-7.59 (m, 3H), 7.58-7.55 (m, 2H), 7.50-7.45 (m, 2H), 7.38 (d, *J* = 8.0 Hz, 1H), 7.26 (d, *J* = 8.3 Hz, 2H), 7.23-7.20 (m, 1H), 7.15 (dd, *J* = 8.9, 13.9 Hz, 1H), 7.03 (dd, *J* = 5.7, 3.4 Hz, 1H), 4.30 (d, *J* = 8.6 Hz, 1H), 3.65 (d, *J* = 15.5 Hz, 1H), 3.32 (s, 3H), ^{13}C NMR (100 MHz, $CDCl_3$), δ 144.5, 141.2, 140.7, 137.1, 136.5, 133.7, 133.5, 132.1, 130.4, 130.0, 128.2, 127.5, 124.2, 122.8, 121.7, 94.6, 92.9, 87.5, 21.4, HRMS (ESI), *m/z* calcd for $C_{23}H_{18}BrNO_2S$ $[M+H]^+$ 452.0314, found 452.0319.

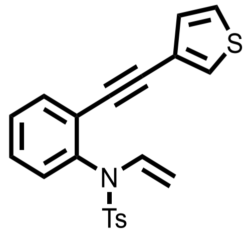

4-Methyl-N-(2-(thiophen-3-ylethynyl)phenyl)-N-vinylbenzenesulfonamide (5e): Light viscous liquid, yield (340 mg, 89%), ^{1}H-NMR (400 MHz, $CDCl_3$), δ 7.60 (d, *J* = 8.2 Hz, 2H), 7.54-

7.52 (m, 1H), 7.37-7.32 (m, 3H), 7.27 (d, *J* = 8.1 Hz, 1H), 7.23 (dd, *J* = 4.8, 1.6 Hz, 1H), 7.15-7.12 (m, 3H), 6.99 (d, *J* = 4.8 Hz, 1H), 4.28 (d, *J* = 8.9 Hz, 1H), 3.81 (d, *J* = 15.5 Hz, 1H), 2.27 (s, 3H), ^{13}C NMR (100 MHz, $CDCl_3$), δ 143.8, 140.7, 136.7, 136.6, 133.3, 133.2, 131.4, 129.8, 129.6, 129.1, 129.0, 128.1, 127.5, 125.0, 122.0, 93.9, 89.4, 85.3, 21.5, HRMS (ESI), *m/z* calcd for $C_{21}H_{17}NO_2S_2$ [M+H]$^+$ 380.0773, found 380.0803.

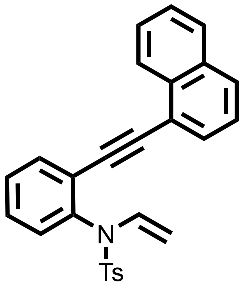

4-Methyl-N-(2-(naphthalen-1-ylethynyl)phenyl)-*N*-vinylbenzenesulfonamide (5f): Dark brown viscous liquid, yield (565 mg, 88%), ^{1}H-NMR (400 MHz, $CDCl_3$), δ 8.33 (d, *J* = 8.3 Hz, 1H), 7.83 (t, *J* = 8.3, Hz, 2H), 7.69 (dd, *J* = 7.1, 2.2 Hz, 1H), 7.60-7.51 (m, 5H), 7.41 (t, *J* = 7.3 Hz, 3H), 7.33 (dd, *J* = 15.3, 9.0 Hz, 1H), 7.20 (dd, *J* = 7.3, 2.0 Hz, 1H), 6.90 (d, *J* = 8.1 Hz, 2H), 4.41(dd, *J* = 9.0, 0.9 Hz, 1H), 3.93(dd, *J* = 15.4, 0.9 Hz, 1H), 1.98 (s, 3H), ^{13}C NMR (100 MHz, $CDCl_3$), δ 143.8, 136.6, 136.4, 133.8, 133.6, 133.1, 131.6, 130.6, 129.5, 129.4, 129.2, 128.9, 128.1, 127.5, 126.8, 126.7, 126.4, 125.1, 125.0, 120.7, 93.9, 92.3, 90.5, 77.3, 21.2, HRMS (ESI), *m/z* calcd for $C_{27}H_{21}NO_2S$ [M+H]$^+$ 424.1366, found 424.1389.

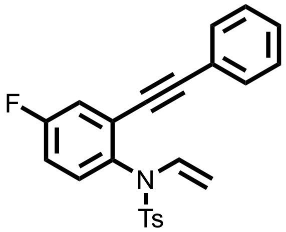

N-(4-Fluoro-2-(phenylethynyl)phenyl)-4-methyl-N-vinylbenzenesulfonamide (5g): Light brown solid, yield (301 mg, 77%), ^{1}H-NMR (x00 MHz, $CDCl_3$), δ 7.72 (d, *J* = 8.3 Hz, 1H), 7.59 (d, *J* = 8.3, Hz, 2H), 7.30 (s, 4H), 7.23-7.07 (m, 6H), 4.31 (dd, *J* = 8.9, 1.2 Hz, 1H), 3.83 (dd, *J* = 15.5, 1.2 Hz, 1H), 2.23 (s, 3H), ^{13}C NMR (100 MHz, $CDCl_3$), δ 162.1 (d, *J* = 250.1), 145.1, 143.9, 136.3, 135.0, 133.3-133.1(m), 132.8 (d, *J* = 3.5 Hz), 131.7, 129.9, 129.6, 128.8, 128.1, 127.5, 126.7, 122.4, 118.2 (dd, *J* = 349.4, 24.8 Hz), 113.7 (dd, *J* = 194.3, 25.5Hz), 108 (d, *J* = 4.2 Hz),

106.7 (d J = 24.1 Hz), 95.1, 93.8, 84.7, 21.4, HRMS (ESI), *m/z* calcd for $C_{23}H_{18}FNO_2S$ $[M+H]^+$ 392.1115, found 392.1122.

Scheme S12. Preparation of *tert*-Butyl (2-(phenylethynyl)phenyl)(vinyl)carbamate(**5h**)

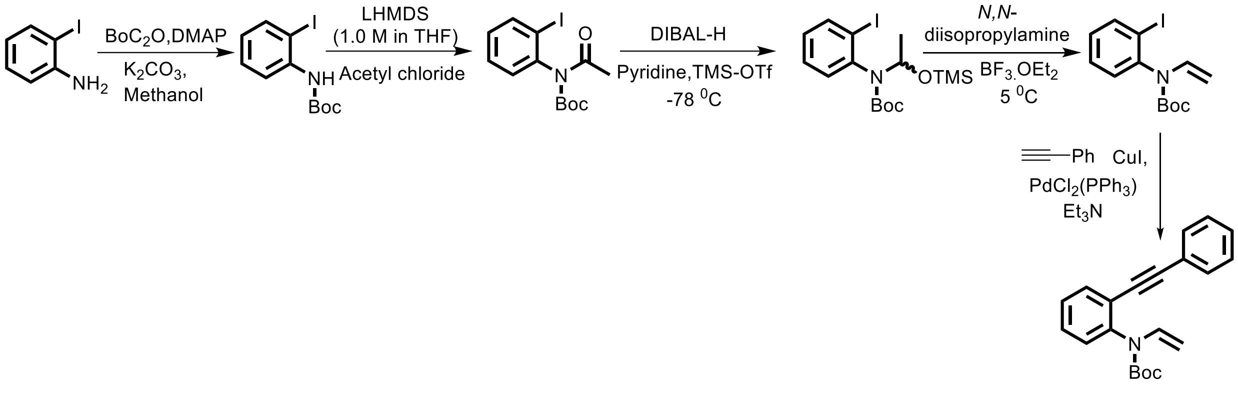

Typical Procedure

Preparation of *tert*-Butyl (2-(phenylethynyl)phenyl)(vinyl)carbamate from 2-Iodoaniline

To a solution of 2-iodoaniline (9.69 mmol, 2.12 g) in anhydrous THF (100 mL) was added Boc_2O (29 mmol, 6.33 g) followed by DMAP (0.97 mmol, 0.119). The solution was stirred at reflux for 2-12h. The concentrated to dryness and partitioned between 0.5 N HCl (100 mL) and EtOAc (100 mL). The organic phases (EtOAc) were washed with brine (50 mL), dried over Na_2SO_4, filtered and concentrated to afford the crude di-Boc product as brown solid. The crude material was dissolved in methanol (100 mL), treated with K_2CO_3 (29 mmol, 4.0 g) and stirred at refluxed for 2-12 h. The mixture was concentrated to dryness, the residue was purified by column chromatography on silica gel using hexane/EtoAc (95/5). A white solid mono-Boc was obtained.[12] Yield (2.89 g, 94%), ^{1}H-NMR (500 MHz, $CDCl_3$), δ 8.08 (d, J = 8.2 Hz, 1H), 7.77 (dd, J = 7.9, 1.5 Hz, 1H), 7.34 (td, J = 7.8, 1.4 Hz, 1H), 6.85 (s, 1H), 6.79 (td, J = 7.8, 1.4 Hz, 1H), 1.56 (s, 9H), ^{13}C NMR (100 MHz, $CDCl_3$), δ 152.5, 138.8, 129.1, 124.6, 120.1, 88.7, 81.0, 28.3.

Preparation of *tert*-Butyl acetyl(2-iodophenyl)carbamate from *tert*-Butyl (2-(phenylethynyl)phenyl)(vinyl)carbamate

To a stirred solution of *tert*-butyl (2-(phenylethynyl)phenyl)(vinyl)carbamate (7.43 mmol, 2.37 g) in THF (15 mL) was added LHMDS (1.0 M in THF) (11.15 mmol) at – 78 0 ^{0}C and stirred for 1h. Acetyl chloride (15.0 mmol, 1.1 mL) was added at – 78 ^{0}C and stirred for 10 min. The reaction mixture was allowed to – 5 ^{0}C and stirred for 2 h. Then quenched with saturated $NaHCO_3$ solution and extracted with ethyl acetate (EtOAc). The organic layer was washed with brine (50 mL), dried over Na_2SO_4 and concentrated under reduced pressure. The crude product was purified by silica gel column chromatography hexane/EtOAc (95/5) to afford a white colored amide.[12] Yield (1.79 g, 67%), ^{1}H-NMR (500 MHz, $CDCl_3$), δ 7.89 (dd, *J* = 7.9, 1.3 Hz, 1H), 7.40 (td, *J* = 7.6, 1.3 Hz, 1H), 7.18 (dd, *J* = 7.9, 1.6 Hz, 1H), 7.06 (td, *J* = 7.6, 1.6 Hz, 1H), 2.66 (s, 3H), 1.41 (s, 9H), ^{13}C NMR (125 MHz, $CDCl_3$), δ 172.2, 151.5, 141.7, 139.2, 129.3, 129.2, 129.1, 99.4, 83.4, 27.8, 26.6.

Preparation of *tert*-Butyl-(2-iodophenyl)(1-((trimethylsilyl)oxy)ethyl)carbamate from *tert*-Butyl-acetyl(2-iodophenyl)carbamate

To a solution of amide (4.87 mmol) in CH_2Cl_2 (30 mL) was added DIBAL-H (9.47 mmol, 10 mL) at -78 ^{0}C after stirring for 1h, the reaction mixture was treated with pyridine (14.61 mmol, 1.2 mL) and then TMS-OTf (12.18 mmol, 2.20 mL). The reaction mixture was stirrer at -78 ^{0}C for 10 min and then slowly warmed to -40 ^{0}C and stirrer for 1h and then quenched with 15 % aqueous sodium potassium tartrate solution and diluted with diethyl ether (15 mL). The resulting solution was warmed to room temperature and stirrer vigorously until two layers were completely separated. The mixture was extracted with diethyl ether (10 mL x 3) and the organic layer was washed with brine (50 mL) and dried over Na_2SO_4 and concentrated under reduced pressure and the white oil residue was used for next step.[12]

Preparation of *tert*-Butyl (2-iodophenyl)(vinyl)carbamate from tert-Butyl (2-iodophenyl)(1-((trimethylsilyl)oxy)ethyl)carbamate

Amide (*tert*-butyl (2-iodophenyl)(1-((trimethylsilyl)oxy)ethyl)carbamate) (4.32 mmol, 1.88 g) was dissolved in dry CH_2Cl_2 (20 mL) and added *N*,*N*-diisopropylamine (12.96 mmol, 2.3 mL), $BF_3.OEt_2$ (10.8 mmol, 1.4 mL) -5 ^{0}C and stirrer for 10 min then allowed to room temperature. After stirring for 2h, the reaction mixture was quenched with Et_3N (2 mL) and extracted with CH_2Cl_2 (15 mL x 3). The organic layer was dried over Na_2SO_4 and concentrated under reduced

pressure. The crude product was purified by silica gel column chromatography using hexane/ EtOAc (98/2). A white solid enamine was recovered.[12] Yield (1039 mg, 70%), ^{1}H-NMR (500 MHz, $CDCl_3$), δ 7.90 (d, *J* = 7.8 Hz, 1H), 7.39 (t, *J* = 7.5, 1H), 7.32-7.25 (m, 1H), 7.21-7.16 (m, 1H), 7.03 (t, *J* = 7.6 Hz, 1H), 4.23 (d, *J* = 7.8 Hz, 1H), 3.65 (d, *J* = 15.8 HZ, 1H), 1.35 (s, 9H), ^{13}C NMR (125 MHz, $CDCl_3$), δ 151.6, 140.8, 139.7, 138.8, 133.1, 129.7, 129.3, 99.6, 93.3, 81.4, 28.0.

Preparation of *tert*-Butyl (2-(phenylethynyl)phenyl)(vinyl)carbamate from *tert*-Butyl-(2-iodophenyl)(vinyl)carbamate

To a solution of *tert*-butyl-(2-iodophenyl)(vinyl)carbamate (1.0 mmol, 345 mg) and phenyl acetylene (1.5 mmol, 0.17 mL) in trimethylamine (degassed, 5 mL) was added $PdCl_2(PPh_3)$ (0.03 mmol, 21 mg) and CuI (0.06 mmol, 12 mg) under nitrogen. The resulting mixture was stirrer at room temperature for 6 h. The reaction mixture was filtered and washed with diethyl ether (10 mL). The combined filtrate was concentrate and the residue was purified by column chromatography on silica gel using (hexane/Et_3N). A yellow oil *tert*-Butyl (2-(phenylethynyl)phenyl)(vinyl)carbamate (**5h**) was obtained. Yield (227 mg, 93%), ^{1}H-NMR (400 MHz, $CDCl_3$), δ 7.60 (d, *J* = 7.3 Hz, 1H), 7.48-7.45 (m, 2H), 7.39-7.30 (m, 6H), 7.23-7.16 (m, 1H), 4.21 (d, *J* = 8.8 Hz, 1H), 3.78 (d, *J* = 15.8 Hz, 1H) 1.24 (s, 9H), ^{13}C NMR (100 MHz, $CDCl_3$), δ 152.5, 139.9, 134.1, 132.6, 131.7, 129.1, 129.0, 128.4, 128.3, 127.8, 123.3, 123.0, 93.5, 93.1, 85.7, 28.0, HRMS (ESI), *m/z* calcd for $C_{21}H_{21}NO_2$ $[M+H]^+$ 320.1645, found 320.1641.

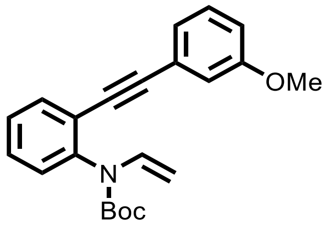

Tert-butyl (2-((3-methoxyphenyl)ethynyl)phenyl)(vinyl)carbamate (5i): Yellow viscous liquid, yield (333 mg, 95%), ^{1}H-NMR (400 MHz, $CDCl_3$), δ 7.60 (d, *J* = 7.3 Hz, 1H), 7.42 (t, *J* = 7.7 Hz, 1H), 7.34 (t, *J* = 7.7 Hz, 2H), 7.21 (d, *J* = 7.3 Hz, 1H), 6.98-6.96 (m, 2H), 6.77 (tt, *J* = 8.9, 2.3 Hz, 1H), 4.22 (d, *J* = 8.7 Hz, 1H), 3.76 (d, *J* = 15.5 Hz, 1H), 1.35 (s, 9H), ^{13}C NMR (100 MHz, $CDCl_3$), δ 163.9, 161.5, 140.2, 139.7, 134.1, 132.8, 129.8, 129.3, 127.9, 114.6, 114.4, 104.5, 93.2, 87.6, 28.0, HRMS (ESI), *m/z* calcd for $C_{22}H_{23}NO_3$ $[M+H]^+$ 350.1751, found 350.1770.

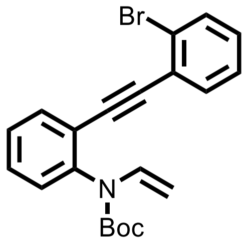

***tert*-Butyl (2-((2-bromophenyl)ethynyl)phenyl)(vinyl)carbamate (5j):** Light green viscous liquid, yield (319 mg, 80%), ^{1}H-NMR (400 MHz, $CDCl_3$), δ 7.67 (d, *J* = 7.5 Hz, 1H), 7.57 (dd, *J* = 8.0, 0.8 Hz, 1H), 7.49 (dd, *J* = 7.7, 1.5 Hz, 1H), 7.42-7.32 (m, 3H), 7.25 (td, *J* = 7.5, 1.1 Hz, 1H), 7.22-7.18 (m, 1H), 7.14 (td, *J* = 7.8, 1.6 Hz, 1H), 4.22 (d, *J* = 8.3 Hz, 1H), 3.78 (d, *J* = 15.8 Hz, 1H), 1.33 (s, 9H), ^{13}C NMR (100 MHz, $CDCl_3$), δ 152.3, 139.8, 134.3, 133.6, 133.0, 132.4, 129.5, 127.8, 127.0, 125.3, 122.9, 93.1, 91.7, 90.1, 81.2, 28.0, HRMS (ESI), *m/z* calcd for $C_{21}H_{20}BrNO_2$ $[M+H]^+$ 398.0750, found 398.0744.

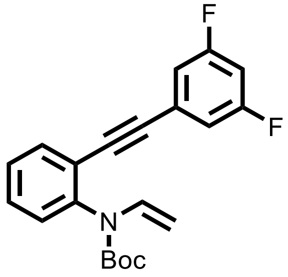

***tert*-Butyl (2-((3,5-difluorophenyl)ethynyl)phenyl)(vinyl)carbamate (5k):** Yellowish brown viscous, liquid (336 mg, 94%), ^{1}H-NMR (400 MHz, $CDCl_3$), δ 7.60 (d, *J* = 7.3 Hz, 1H), 7.40-7.30 (m, 1H), 7.21 (t, *J* = 7.9 Hz, 2H), 7.07 (d, *J* = 7.6 Hz, 1H), 7.01-6.99 (m, 2H), 6.86 (dd, *J* = 8.5, 2.5 Hz, 1H), 4.21 (d, *J* = 8.5 Hz, 1H), 3.79 (d, *J* = 15.9 Hz, 1H), 1.34 (s, 9H), ^{13}C NMR (100 MHz, $CDCl_3$), δ 163.9, 161.4, 140.2, 139.7, 134.1, 132.8, 129.8, 129.3, 127.9, 114.6, 114.4, 104.5, 93.2, 87.6, 28.0, HRMS (ESI), *m/z* calcd for $C_{21}H_{19}F_2NO_2$ $[M+H]^+$ 356.1457, found 356.1462.

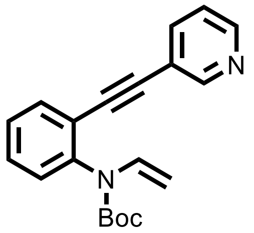

***tert*-Butyl (2-(pyridin-3-ylethynyl)phenyl)(vinyl)carbamate (5l)**: Light brown viscous liquid, yield (241 mg, 75%), ^{1}H-NMR (400 MHz, $CDCl_3$), δ 8.68 (d, *J* = 1.4 Hz, 1H), 8.51 (dd, *J* = 5.0, 1.4 Hz, 1H), 7.73 (dt, *J* = 7.9, 1.8 Hz, 1H), 7.61 (d, *J* = 7.3 Hz, 1H), 7.41(t, *J* = 7.5 Hz, 1H), 7.34 (t, *J* = 7.5 Hz, 2H), 7.25-7.20 (m, 2H), 4.21 (d, *J* = 8.9 Hz, 1H), 3.76 (d, *J* = 15.8 Hz, 1H), 1.32 (s, 9H), ^{13}C NMR (100 MHz, $CDCl_3$), δ 152.1, 148.9, 148.7, 138.6, 134.1, 132.7, 131.9, 130.3, 127.9, 123.1, 123.0, 122.2, 120.2, 117.8, 93.2, 89.8, 89.0, 28.0, HRMS (ESI), *m/z* calcd for $C_{20}H_{20}N_2O_2$ $[M+H]^+$ 321.1598, found 321.1608.

General Experimental Procedure for Phenyl(2-(2,2,2-trifluoroethyl)benzofuran (2a)

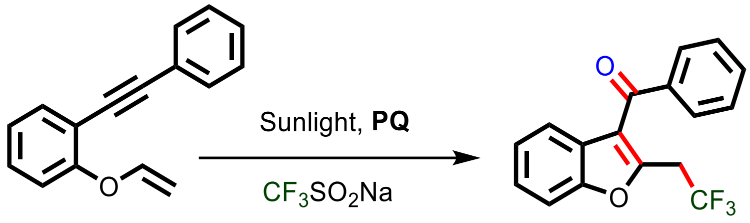

1a **2a**

Reaction Setup in Day Light **Reaction Setup under CFL Light**

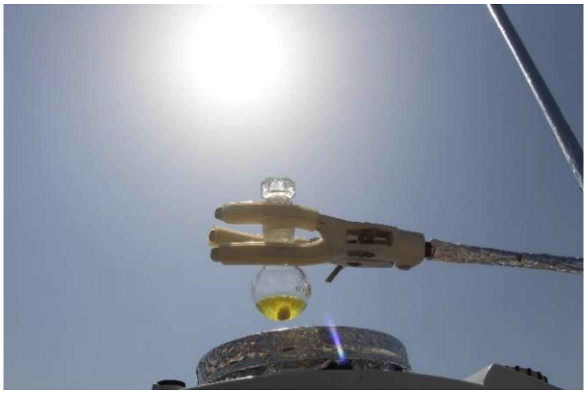

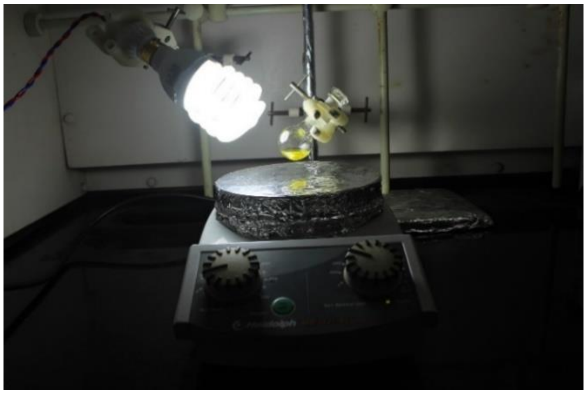

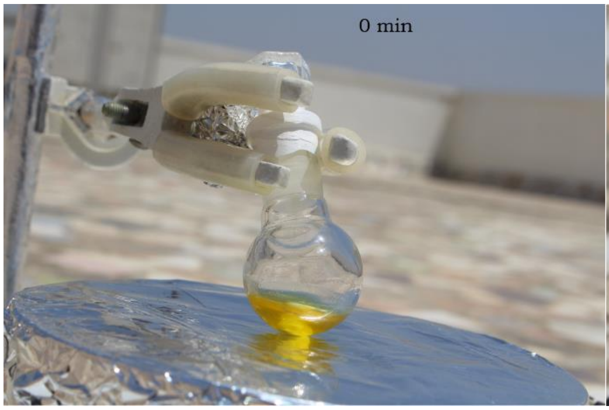

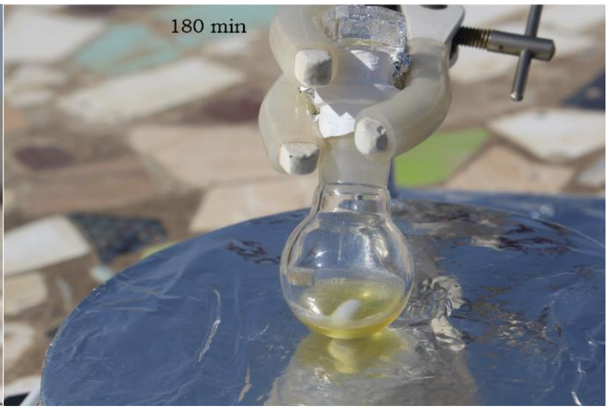

Reaction at 0 min **Reaction after 3 hours**

1-(Phenylethynyl)-2-(vinyloxy)benzene **1a** (22 mg, 0.1 mmol, 1.0 equiv.), CF_3SO_2Na (47 mg, 0.6 mmol), and photocatalyst (**PQ**) (2.1 mg, 0.01 mmol, 0.1 equiv.) were added to a 5 mL round bottom flask with magnetic stir bar. The flask was evacuated and backfilled with argon and then $CH_3CN + H_2O$ (900 + 100 µL) was added to the mixture. The reaction mixture was stirred up to 4-6 h under sunlight. The progress of the reaction was monitored by TLC. After completion of the reaction, solvent was removed on rotary evaporator under vacuum. The residue was washed with 1 N NaOH (2 mL) and extracted with diethyl ether (5 mL x 3). The combined organic layers were washed with brine (10 mL), dried over Na_2SO_4, and the residue was purified by flash column chromatography on silica gel (n-Hexane: Dichloromethane = 9: 1) to afford the desired product **2a**.

Experimental Procedure under CFL Light: The above reaction mixture was stirred up to 16-24 h under house hold CFL (23 W) bulb at room temperature. After completion of reaction, work-up was done by following above mention procedure.

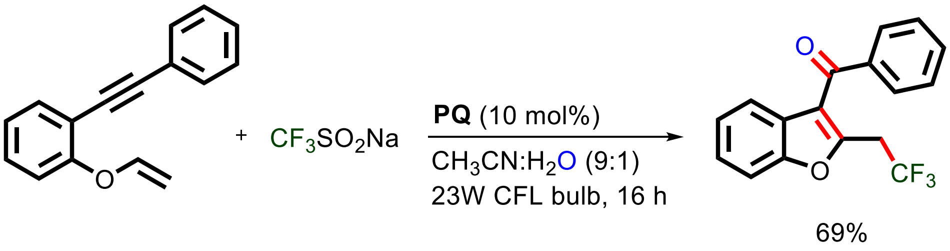

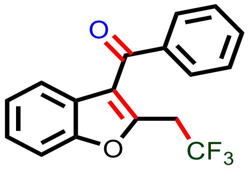

Phenyl(2-(2,2,2-trifluoroethyl)benzofuran-3-yl)methanone (2a): White viscous liquid, yield (23.1 mg, 76%), ^{1}H NMR (400 MHz, $CDCl_3$), δ 7.85-7.82 (m, 2H), 7.63 (t, *J* = 7.6 Hz, 1H), 7.56 (d, *J* = 8.3 Hz, 1H), 7.49 (t, *J* = 7.6 Hz, 2H), 7.37-7.34 (m, 1H), 7.22-7.18 (m, 2H), 3.92 (q, *J* = 10.0 Hz, 2H), ^{13}C NMR (100 MHz, $CDCl_3$), δ 191.1, 154.2, 139.2, 138.3, 133.3, 131.6, 130.1 (q, $J_{C, F}$ = 279.8 Hz, CF_3), 129.3, 128.6 125,9 125.6, 123.9, 121.7, 111.6, 32.8 (q, $J_{C, F}$ = 32.3 Hz, CH_2CF_3), ^{19}F NMR (376 MHz, $CDCl_3$), δ -63.8 (t, *J* = 10.0 Hz, 3F), HRMS (ESI), *m/z* calcd for $C_{17}H_{11}F_3O_2$ $[M+H]^+$ 305.0784, found 305.0797.

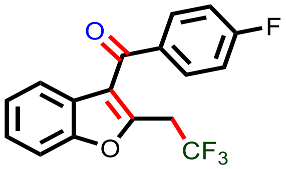

(4-Fluorophenyl)(2-(2,2,2-trifluoroethyl)benzofuran-3-yl)methanone (2b): White viscous liquid, yield (23.5 mg, 73%), ^{1}H-NMR (400 MZ, $CDCl_3$), δ 7.90-7.86 (m, 2H), 7.56 (d, *J* = 8.3 Hz, 1H), 7.36 (td, *J* = 7.6, 1.6 Hz, 1H), 7.21-7.14 (m, 4H), 3 92 (q, *J* = 10.0 Hz, 2H), ^{13}C NMR (100 MHz, $CDCl_3$), δ 189.5, 167.2, 164.6, 154.2, 139.2, 134.5, 132.1 (d, *J* = 9.5 Hz), 125.7, 124.1 (q, $J_{C,F}$ = 278.1 Hz, CF_3), 124.0, 121.4, 115.8 (d. $J_{C,F}$ = 22.0 Hz), 114.0, 111.7, 32.7 (q, *J* = 31.9 Hz, CH_2CF_3), ^{19}F NMR (376 MHz, $CDCl_3$), δ -63.9 (t, *J* = 10.1 Hz, 3F), -104.1 to -104.2 (m, F), HRMS (ESI), *m/z* calcd for $C_{17}H_{10}F_4O_2$ $[M+H]^+$ 323.0690, found 323.0680.

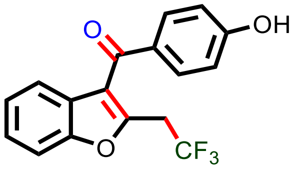

(4-Hydroxyphenyl)(2-(2,2,2-trifluoroethyl)benzofuran-3-yl)methanone (2c): White solid, yield (22.7 mg, 71%), ^{1}H-NMR (400 MHz, $CDCl_3$), δ 7.80 (d, *J* = 8.7 Hz, 2H), 7.54 (d, *J* = 8.3 Hz, 1H), 7.35 (td, *J* = 7.7, 1.2 Hz, 1H), 7.27 (d, *J* = 7.7, 1H), 7.20 (t, *J* = 7.3 Hz, 1H), 6.90 (d, *J* = 8.7 Hz, 2H), 3.88 (q, *J* = 10 0 Hz 2H), ^{13}C NMR (100 MHz, $CDCl_3$), δ 189.8, 160.7, 154.2, 151.2 (q, *J* = 3.6 Hz), 139.6, 132.3, 130.9, 126.0, 125.5, 124.5 (q, $J_{C, F}$ = 278.4 Hz, CF_3), 121.6, 115.5,

111.6, 32.7 (q, $J_{C, F}$ = 27.3 Hz, CH_2CF_3), ^{19}F NMR (376 MHz, $CDCl_3$), δ -63.9 (t, $J_{C, F}$ = 10.2 Hz, 3F), HRMS (ESI), *m/z* Calcd for $C_{17}H_{11}F_3O_3$ $[M+H]^+$ 321.0733, found 321.0716.

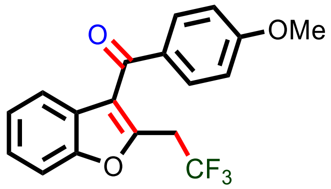

(4-Methoxyphenyl)(2-(2,2,2-trifluoroethyl)benzofuran-3-yl)methanone (2d): White viscous liquid, yield (25.1 mg, 75%), ^{1}H-NMR (400 MHz, $CDCl_3$), δ 7.85 (d, *J* = 8.8 Hz, 2H), 7.55 (d, *J* = 8.4 Hz, 1H), 7.35 (td, *J* = 7.7, 1.2 Hz, 1H), 7.27 (d, *J* = 7.6 Hz, 1H), 7.21 (t, *J* = 7.7 Hz, 1H), 6.96 (d, *J* = 8.8 Hz, 2H), 3.93-3.86 (m, 5H), ^{13}C NMR (100 MHz, $CDCl_3$), δ 189.5, 163.9, 154.2, 151.2, 131.9, 130.9, 126.1, 125.5, 124.5 (q, $J_{C, F}$ = 277.0 Hz, CF_3), 123.8, 121.7, 113.8, 111.6, 55.5, 32.7 (q, $J_{C, F}$ = 32.4, Hz, CH_2CF_3), ^{19}F NMR (376 MHz, $CDCl_3$), δ -63.9 (t, *J* = 10.0 Hz, 3F), HRMS (ESI), *m/z* calcd for $C_{18}H_{13}F_3O_3$ $[M+H]^+$ 335.0890, found 335.0886.

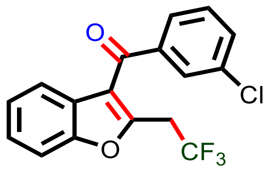

(3-Chlorophenyl)(2-(2,2,2-trifluoroethyl)benzofuran-3-yl)methanone (2e): White viscous liquid, yield (21.0 mg, 62%), ^{1}H-NMR (500 MHz, $CDCl_3$), δ 7.86 (t, *J* = 1.8 Hz, 1H), 7.73 (dt, *J* = 7.6, 1.2 Hz, 1H), 7.64 (ddd, *J* = 7.9, 2.1, 0.9 Hz, 1H), 7.61 (d, *J* = 8.4 Hz, 1H), 7.47 (t, *J* = 7.9 Hz, 1H), 7.41 (td, *J* = 7.7, 1.3 Hz, 1H), 7.26 (d, *J* = 7.1 Hz, 1H), 7.22 (d, *J* = 7.7 Hz, 1H), 3.97 (q *J* = 10.0 Hz, 2H), ^{13}C NMR (176.0 MHz, $CDCl_3$), δ 189.7, 154.2, 152.5, 139.8, 135.0, 133.3, 130.3, 129.1, 127.5, 125.8, 125.5, 124.4, 124.1 (q, $J_{C, F}$ = 279.4 Hz, CF_3), 121.5, 120.0, 111.8, 32.9 (q, $J_{C, F}$ = 32.4 Hz, CH_2CF_3), ^{19}F NMR (476 MHz, $CDCl_3$), δ -63.8 (t, *J* = 10.4 Hz, 3F), HRMS (APCI), *m/z* Calcd for $C_{17}H_{10}ClF_3O_2$ $[M+H]^+$ 339.0394, found 339.0391.

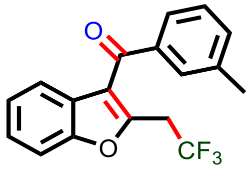

***m*-Tolyl(2-(2,2,2-trifluoroethyl)benzofuran-3-yl)methanone (2f)**: White viscous liquid, yield (18.8 mg, 59%), ^{1}H-NMR (400 MHz, $CDCl_3$), δ 7.67 (s, 1H), 7.61 (d, *J* = 7.37 Hz, 1H), 7.55 (d, *J*

= 8.4, 1H), 7.4 (d, J = 7.5 Hz, 1H), 7.38-7.33 (m, 2H), 7.25-7.18 (m, 2H), 3.90 (q, J = 10.0 Hz, 2H), 2.40 (s, 3H), ^{13}C NMR (100 MHz, $CDCl_3$), δ 191.3, 154.2, 151.9, 139.2, 138.5 (d J = 19.7 Hz), 134.1, 129.6, 128.5, 127.0 (q $J_{C, F}$ = 278.5 Hz, CF_3), 126.6, 126.0, 125.5, 123.9, 121.7, 114.0, 116.6, 32.8 (q, $J_{C,F}$ = 32.3.2 Hz, CH_2CF_3), 21.2, ^{19}F NMR (376 MHz, $CDCl_3$), δ -63.7 (t, J = 10.0 Hz, 3F), HRMS (ESI), *m/z* calcd for $C_{18}H_{13}F_3O_2$ $[M+H]^+$ 319.0940, found 319.0922.

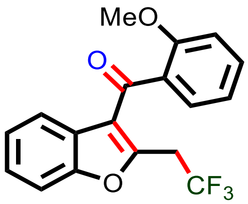

(2-Methoxyphenyl)(2-(2,2,2-trifluoroethyl)benzofuran-3-yl)methanone (2g): White viscous liquid, yield (21.7 mg, 65%), ^{1}H-NMR (400 MHz, $CDCl_3$), δ 7.53-7.48 (m, 2H), 7.44 (d, J = 7.5 Hz, 1H), 7.30 (t, J = 7.7 Hz, 1H), 7.14 (t, J = 7.7 Hz, 1H), 7.07 (t, J = 7.5 Hz, 2H), 6.99 (d, J = 8.4 Hz, 1H), 3.93 (q, J = 10.0 Hz, 2H), 3.61 (s, 3H), ^{13}C NMR (100 MHz, $CDCl_3$), δ 190.7, 157.4, 154.2, 152.6, 132.9 129.8, 129.3 125.5, 125.3, 123.9, 123.4 (q, J = 278.1 Hz, CF_3), 121.4, 121.2, 120.8, 111.5, 111.4, 44.6, 33.0 (q, J = 32.3 Hz, CH_2CF_3), ^{19}F NMR (376 MHz, $CDCl_3$), δ -63.5 (t, J = 9.9 Hz, 3F), HRMS (ESI), *m/z* calcd for $C_{18}H_{13}F_3O_3$ $[M+H]^+$ 335.0890, found 335.0871.

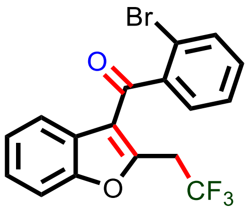

(2-Bromophenyl)(2-(2,2,2-trifluoroethyl)benzofuran-3-yl)methanone (2h): Light yellow viscous liquid, yield (21.8 mg, 57%), ^{1}H-NMR (400 MHz, $CDCl_3$), δ 7.69 (d, J = 7.2 Hz, 1H), 7.53 (d, J = 8.4 Hz, 1H), 7.47-7.40 (m, 3H), 7.33 (t, J = 7.8 Hz, 1H), 7.16 (t, J = 7.6 Hz, 1H), 6.91 (d, J = 7.9 Hz, 1H), 3.96 (q, J = 9.9 Hz, 2H), ^{13}C NMR (100 MHz, $CDCl_3$), δ 190.5, 154.4, 154.0, 141.5, 136.3, 133.5, 131.9, 128.6, 127.8, 125.8, 125.0, 124.4, 124.2 (q, J = 277.6 Hz, CF_3), 121.2, 119.0, 111.6, 33.2 (q, J = 32.4Hz, CH_2CF_3), ^{19}F NMR (376 MHz, $CDCl_3$), δ -63.2 (t, J = 10.0 Hz, 3F), HRMS (ESI), *m/z* calcd for $C_{17}H_{10}BrF_3O_2$ $[M+H]^+$ 382.9889, found 382.9873.

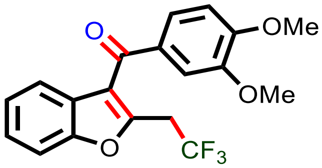

(3,4-Dimethoxyphenyl)(2-(2,2,2-trifluoroethyl)benzofuran-3-yl)methanone (2i): White viscous liquid, yield (23.3 mg, 64%), ^{1}H-NMR (400 MHz, $CDCl_3$), δ 7.55 (d, *J* = 8.3 Hz, 1H), 7.49 (d, *J* = 2.0 Hz, 1H), 7.45 (dd, *J* = 8.3, 2.0 Hz, 1H), 7.37–7.31 (m, 2H), 7.21 (t, *J* = 7.6 Hz, 1H), 6.87 (d, *J* = 8.4 Hz, 1H), 3.96 (s, 3H), 3.93–3.85 (m, 5H), ^{13}C NMR (100 MHz, $CDCl_3$), δ 189.5, 154.2, 153.7, 151.2 (d, *J* = 3.7 Hz), 149.2, 130.9, 126.2, 125.5, 124.9, 124.3 (q, $J_{C, F}$ = 278.6 Hz, CF_3) 123.8, 121.7, 120.8, 111.6, 111.2, 110.0, 56.1, 56.0, 30.8 (q, $J_{C, F}$ = 32.2 Hz, CH_2CF_3), ^{19}F NMR (376 MHz, $CDCl_3$), δ -63.9 (t, *J* = 10.2 Hz, 3F), HRMS (ESI), *m/z* calcd for $C_{19}H_{15}F_3O_4$ $[M+H]^+$ 365.0995, found 365.0983.

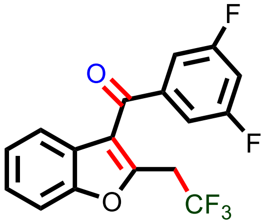

(3,5-Difluorophenyl)(2-(2,2,2-trifluoroethyl)benzofuran-3-yl)methanone. (2j): White viscous liquid, yield (24.2 mg, 71%), ^{1}H-NMR (400 MHz, $CDCl_3$), δ 7.57 (d, *J* = 8.3 Hz, 1H), 7.40–7.38 (m, 1H), 7.36-7.32 (m, 2H), 7.24 (t, *J* = 7.5 Hz, 1H), 7.18 (d, *J* = 7.6 Hz, 1H), 7.08 (tt, *J* = 8.4, 2.3, Hz, 1H), 3.93 (q, *J* = 10.0 Hz, 2H), ^{13}C NMR (100 MHz, $CDCl_3$), δ 188.4, 164.2 (d, *J* = 11.8 Hz), 161.7 (d, *J* = 11.7 Hz), 154.3, 152.8 (q, *J* = 3.3 Hz), 141.1(q, *J* = 7.8 Hz), 132.1 (d, *J* = 9.9 Hz), 128.5 (d *J* = 12.1 Hz), 125.9, 125.2, 124.3, 124.0 (q, $J_{C, F}$ = 278.5 Hz, CF_3), 121.3, 119.6, 112.2 (dd, *J* = 18.7, 7.4 Hz), 111.8, 30.8 (q, *J* = 32.3 Hz, CH_2CF_3) ^{19}F NMR (376 MHz, $CDCl_3$), δ -63.8 (t, *J* = 9 9 Hz, 3F), -107.3 to -107.4 (m, 2F), HRMS (ESI), *m/z* calcd for $C_{17}H_9F_5O_2$ $[M+H]^+$ 341.0595, found 341.0576.

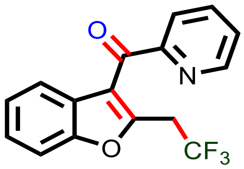

Pyridin-2-yl(2-(2,2,2-trifluoroethyl)benzofuran-3-yl)methanone (2k): White viscous liquid yield (16.8 mg, 55%), ^{1}H-NMR (400 MHz, $CDCl_3$), δ 8.70 (d, *J* = 4.5 Hz, 1H), 8.08 (d, *J* = 7.8 Hz, 1H), 7.95 (td, *J* = 7.7, 1.3 Hz, 1H), 7.56-7.52 (m, 2H), 7.42 (d, *J* = 8.0, Hz, 1H), 7.34 (t, *J* = 7.7 Hz, 1H), 7.22 (d, *J* = 7.7 Hz, 1H), 3.97 (q, *J* = 10.0 Hz, 2H), ^{13}C NMR (100 MHz, $CDCl_3$), δ 190.0, 154.8, 154.3, 148.9, 137.4, 127.2, 126.1, 125.6, 124.3 (q, $J_{C,\ F}$ = 278.6 Hz, CF_3), 123.9, 122.4, 111.4, 33.7 (q, $J_{C, F}$ = 32.7 Hz, CH_2CF_3), ^{19}F NMR (376 MHz, $CDCl_3$), δ -63.7 (t, *J* = 10.2 Hz, 3F), HRMS (ESI), *m/z* calcd for $C_{16}H_{10}F_3NO_2$ $[M+H]^+$ 306.0736, found 306.0733.

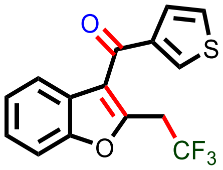

Thiophen-3-yl(2-(2,2,2-trifluoroethyl)benzofuran-3-yl)methanone (2l): Light yellow viscous liquid, yield (19.5 mg, 63%), ^{1}H-NMR (400 MHz, $CDCl_3$), δ 8.01 (s, 1H), 7.58-7.55 (m, 2H), 7.53-7.35 (m, 3H), 7.28-7.27 (m, 1H), 3.91 (q, *J* = 10.0 Hz 2H), ^{13}C NMR (100 MHz, $CDCl_3$), δ 184.1, 154.3, 142.3, 139.2, 134.2, 127.7, 126.6, 125.6, 124.4 (q, $J_{C, F}$ = 278.5 Hz, CF_3), 124.0, 123.9, 121.5, 114.0, 111.7, 32.7 (q, *J* = 31.6 Hz, CH_2CF_3), ^{19}F NMR (376 MHz, $CDCl_3$), δ -63.8 (t, *J* = 10.2 Hz, 3F), HRMS (ESI), *m/z* calcd for $C_{15}H_9F_3O_2S$ $[M+H]^+$ 309.0192, found 309.0175.

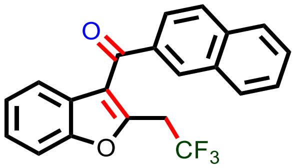

Naphthalen-2-yl(2-(2,2,2-trifluoroethyl)benzofuran-3-yl)methanone (2m): Yellow viscous liquid, yield (22.9 mg, 67%), ^{1}H-NMR (400 MHz, $CDCl_3$), δ 8.34 (s, 1H), 7.95 (s, 2H), 7.92 (d, *J* = 8.2 Hz, 1H), 7.87 (d, *J* = 8.2, Hz, 1H), 7.63 (t, *J* = 7.6 Hz, 1H), 7.57 (t, *J* = 8.2 Hz, 2H), 7.36 (t, *J* = 7.6 Hz, 1H), 7.23 (d, *J* = 8.0 Hz, 1H), 7.17 (t, *J* = 7.6 Hz, 1H), 3.96 (q, *J* = 10.0 Hz, 2H), ^{13}C NMR (100 MHz, $CDCl_3$), δ 190.9, 154.3, 151.9 (q, *J* = 3.6 Hz), 135.7, 135.5, 132.4, 131.4, 129.5, 128.7, 128.7, 127.9, 127.0, 126.6, 126.0 (q, $J_{C, F}$ = 279.8 Hz, CF_3), 125.6, 124.8, 124.0, 121.7, 120.7, 111.6, 32.9 (q, *J* = 32.2 Hz, CH_2CF_3), ^{19}F NMR (376 MHz, $CDCl_3$), δ -63.7 (t, *J* = 10.2 Hz, 3F), HRMS (ESI), *m/z* calcd for $C_{21}H_{13}F_3O_2$ $[M+H]^+$ 355.0940, found 355.0927.

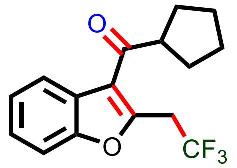

Cyclopentyl(2-(2,2,2-trifluoroethyl)benzofuran-3-yl)methanone (2n): White viscous liquid, yield (12.7 mg, 43%), ^{1}H-NMR (500MHz, $CDCl_3$), δ 7.89-7.87 (m, 1H), 7.60-7.57 (m, 1H), 7.44-7.38 (m, 2H), 4.16 (q, *J* = 10.1 Hz, 2H), 3.72-3.66 (m, 1H), 2.03-1.99 (m, 3H), 1.79-1.70 (m, 4H), ^{13}C NMR (125 MHz, $CDCl_3$), δ 199.6, 154.4, 153.1, 154.4, 153.1, 125.4, 124.5, 124.4 (q, $J_{C,F}$ = 278.8 Hz, CF_3), 124.3, 121.3, 119.8, 111.9, 50.7 33.2 (q, *J* = 32.0 Hz, CH_2CF_3), 29.0, 26.0 ^{19}F NMR (476 MHz, $CDCl_3$), δ -63.6 (t, *J* = 10.4 Hz, 3F), HRMS (ESI), *m/z* calcd for $C_{16}H_{15}F_3O_2$ $[M+H]^+$ 297.1097, found 297.1069.

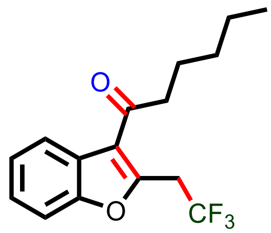

1-(2-(2,2,2-Trifluoroethyl)benzofuran-3-yl)hexan-1-one (2o): White viscous liquid, yield (8.9 mg, 36%), ^{1}H-NMR (400 MHz, $CDCl_3$), δ 7.85-7.83 (m, 1H), 7.57-7.52 (m, 1H), 7.42-7.34 (m, 2H), 4.12 (q, *J* = 10.1 Hz, 2H), 3.01 (t, *J* = 7.3 Hz, 2H), 1.77 (t, *J* = 7.3 Hz, 2H), 1.41-1.37 (m, 4H), 0.91 (t, *J* = 7.0 Hz, 3H), ^{13}C NMR (100 MHz, $CDCl_3$), δ 196.9, 154.4, 125.5, 124.7 (q, *J* = 277.8 Hz, CF_3) 124.5, 124.3, 121.4, 119.9, 43.6, 33.2 (q, *J* = 32.3 Hz, CH_2CF_3), 31.9, 31.4, 29.1, 23.1, 22.5, 13.9 ^{19}F NMR (376 MHz, $CDCl_3$), δ -63.5 (t, *J* = 9.9 Hz, 3F), HRMS (ESI), *m/z* calcd for $C_{16}H_{17}F_3O_2$ $[M+H]^+$ 299.1253, found 299.1282.

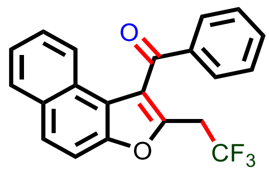

Phenyl(2-(2,2,2-trifluoroethyl)naphtho[2,1-*b*]furan-1-yl)methanone (2p): Light yellow viscous, yield (24.4 mg, 69%), ^{1}H-NMR (400 MHz, $CDCl_3$), δ 7.94 (d, *J* = 8.0 Hz, 2H), 7.91 (d, *J* = 8.2 Hz, 1H), 7.83 (d, *J* = 9.0 Hz, 1H), 7.70 (d, *J* = 9.0 Hz, 1H), 7.60 (t, *J* =7.4 Hz, 1H), 7.54 (d, *J* = 8.4 Hz, 1H), 7.44 (t, *J* = 7.8 Hz, 2H), 7.39 (d, *J* = 7.6 Hz, 1H), 7.27 (d, *J* = 7.7 Hz, 1H), 3.70 (q, *J* = 9.9 Hz, 2H), ^{13}C NMR (100 MHz, $CDCl_3$), δ 192.6, 152.3, 147.1, 137.6, 134.1, 131.1,

129.8, 129.0, 127.6, 126.9, 126.6, 124.9, 124.5, 124.1 (q, $J_{\text{C, F}}$ = 277.6 Hz, CF_3), 122.8, 120.7, 112.1, 32.7 (q $J_{\text{C, F}}$ = 32.4 Hz, CH_2CF_3), ^{19}F NMR (376 MHz, $CDCl_3$), δ -64.1 (t, J = 10.2 Hz, 3F), HRMS (ESI), *m/z* calcd for $C_{21}H_{13}F_3O_2$ $[M+H]^+$ 355.0940, found 355.0930.

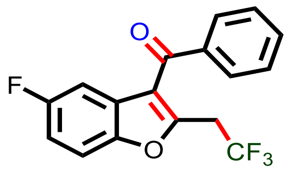

(5-Fluoro-2-(2,2,2-trifluoroethyl)benzofuran-3-yl)(phenyl)methanone (2q): White viscous liquid, yield (17.5 mg, 55%), ^{1}H-NMR (400 MHz, $CDCl_3$), δ 7.80 (d, J = 7.6 Hz, 2H), 7.65 (t, J = 7.5 Hz, 1H), 7.52 (d, J = 7.8 Hz, 2H), 7.50-7.47 (m, 1H), 7.07 (td, J = 9.0, 2.5 Hz, 1H), 6.86 (dd, J = 8.6, 2.5 Hz, 1H), 3.90 (q, J = 9.9 Hz, 2H), ^{13}C NMR (100 MHz, $CDCl_3$), δ 190.6, 160.3, 153.7, 150.4, 138.0, 133.5, 129.2, 128.8, 124.1, (q, J = 278.0 Hz, CF_3), 113.7 (d, J = 26.2 Hz), 112.5 (d, J = 9.6 Hz), 107.5 (d, J = 26.2 Hz), 32.9 (q, J = 32 4 Hz , CH_2CF_3), ^{19}F NMR (376 MHz, $CDCl_3$), δ -63.7 (t, J = 10.2 Hz, 3F), -118.0 to -118.1 (m, 1F), HRMS (ESI), *m/z* calcd for $C_{17}H_{10}F_4O_2$ $[M+H]^+$ 323.0690, found 323.0678.

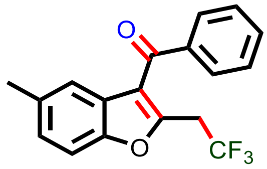

(5-Methyl-2-(2,2,2-trifluoroethyl)benzofuran-3-yl)(phenyl)methanone (2r): Light yellow viscous, yield (19.3 mg, 60%), ^{1}H-NMR (400 MHz, $CDCl_3$), δ 7.88 (dd, J = 8.3, 1.4 Hz, 2H), 7.67 (tt, J = 7.4, 1.2 Hz, 1H), 7.54 (t, J = 7.6 Hz, 2H), 7.46 (d, J = 8.5 Hz, 1H), 7.20 (dd, J = 8.5, 1.3 Hz, 1H), 7.04 (s, 1H), 3.90 (q, J = 10.0 Hz, 2H), 2.37 (s, 3H), ^{13}C NMR (100 MHz, $CDCl_3$), δ 191.3, 152.7, 151.9 (q, J = 3.6 Hz), 138.3, 133.6, 133.3, 129.3, 128.6, 126.9, 126.0, 124.1 (q, $J_{\text{C, F}}$ = 279.1 Hz, CF_3), 121.4, 120.4, 111.1, 32.9 (q $J_{\text{C, F}}$ = 32.2 Hz, CH_2CF_3), 21.3, ^{19}F NMR (376 MHz, $CDCl_3$), δ -63.8 (t, J = 10.2 Hz, 3F), HRMS (ESI), *m/z* calcd for $C_{18}H_{13}F_3O_2$ $[M+H]^+$ 319.0940, found 319.0909.

General Experimental Procedure for Phenyl(2-(2,2,2-trifluoroethyl)benzo[*b*]thiophen-3-yl)methanone (4a)

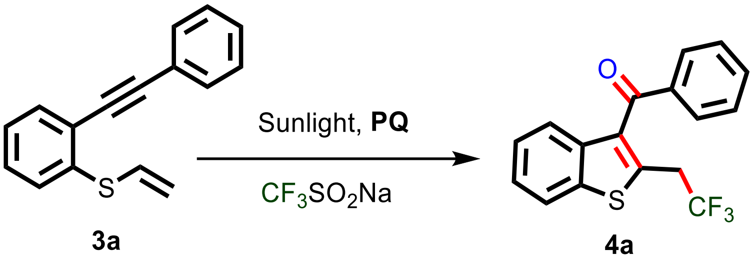

(2-(Phenylethynyl)phenyl)(vinyl)sulfane (**3a**) (22 mg, 0.1 mmol, 1.0 equiv.), CF_3SO_2Na (47 mg, 0.6 mmol), and photocatalyst (**PQ**) (2.1 mg, 0.01 mmol, 0.1 equiv.) were added to a 5 mL round bottom flask with magnetic stir bar. The flask was evacuated and backfilled with argon and then CH_3CN + H_2O (900 + 100 µL) was added to the mixture. The reaction mixture was stirred up to 4-6 h under sunlight. The progress of the reaction was monitored by TLC. After completion of the reaction, the solvent was removed on rotary evaporator under vacuum. The residue was washed with 1N NaOH (2 mL) and extracted with diethyl ether (3 x 5 mL). The combined organic layers were washed with brine (10 mL), dried over Na_2SO_4, and the residue was purified by flash column chromatography on silica gel using n-Hexane: Dichloromethane (9: 1) solvent system to afford the desired product **4a**.

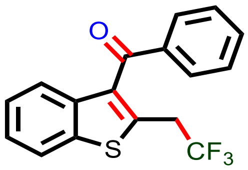

Phenyl(2-(2,2,2-trifluoroethyl)benzo[*b*]thiophen-3-yl)methanone (4a): Light yellow viscous liquid, yield (23.4 mg, 73%), ^{1}H-NMR (400 MHz, $CDCl_3$), δ 7.85 (d, *J* = 8.15 Hz, 1H), 7.81 (d, *J* = 8.1 Hz, 2H), 7.61 (t, *J* = 7.4 Hz, 1H), 7.45 (t, *J* = 7.80, Hz, 2H), 7.36 (td, *J* = 7.5, 1.0 Hz, 1H), 7.31 (d, *J* = 8.0 Hz, 1H), 7.25 (t, *J* = 7.6 Hz, 1H), 3.83 (q, *J* = 10.2 Hz, 2H), ^{13}C NMR (100 MHz, $CDCl_3$), δ 193.1, 138.8, 138.0, 137.8, 136.5, 136.2 (t, *J* = 2.9 Hz), 133.8, 129.8, 128.7, 125.3, 124.9, 124.6 (q, *J* = 278.8 Hz, CF_3), 124.0, 122.0, 33.6 (q, *J* = 32.1 Hz, CH_2CF_3), ^{19}F NMR (376 MHz, $CDCl_3$), δ -65.1 (t, *J* = 10.2 Hz, 3F), HRMS (ESI), *m/z* calcd for $C_{17}H_{11}F_3OS$ $[M+H]^+$ 321.0555, found 321.0582.

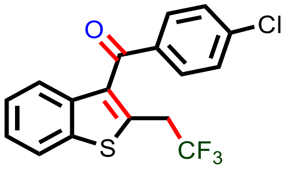

(4-Chlorophenyl)(2-(2,2,2-trifluoroethyl)benzo[*b*]thiophen-3-yl)methanone (4b): Light yellow viscous liquid, yield (24.5 mg, 69%), ^{1}H-NMR (400 MHz, $CDCl_3$), δ 8.85 (d, *J* = 8.1 Hz, 1H), 7.74 (d, *J* = 8.5 Hz, 2H), 7.43 (d, *J* = 8.5 Hz, 2H), 7.39-7.35 (m, 1H), 7.28-7.26 (m. 2H), 7.83 (q, *J* = 10.3 Hz, 2H), ^{13}C NMR (100 MHz, $CDCl_3$), δ 191.8, 140.4, 138.8, 137.7, 136.1, 136.0, 135.6 (d, *J* = 2.9 Hz), 131.2, 129.1, 125.4, 125.0, 124.5 (q, *J* = 278.6 Hz, CF_3), 123.8, 122.1, 31.7 (q, *J* = 31.3 Hz, CH_2CF_3), ^{19}F NMR (376 MHz, $CDCl_3$), δ -65.1 (t, *J* = 10.2 Hz, 3F), HRMS (ESI): *m/z* calcd for $C_{17}H_{10}ClF_3OS$ $[M+H]^+$ 355.0166, found 355.0150.

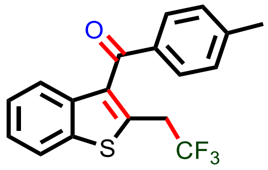

***p*-Tolyl(2-(2,2,2-trifluoroethyl)benzo[*b*]thiophen-3-yl)methanone (4c)**: Yellow viscous liquid, yield (23.7 mg, 71%), ^{1}H-NMR (400 MHz, $CDCl_3$), δ 7.84 (d, *J* = 8.0 Hz, 1H), 7.71 (d, *J* = 8.1 Hz, 2H), 7.37-7.33 (m, 2H), 7.27-7.23(m, 3H), 3.81 (q, *J* = 10.2 Hz, 2H), 2.42 (s, 3H), ^{13}C NMR (100 MHz, $CDCl_3$), δ 192.7, 144.9, 138.8, 138.0, 136.8, 136.2, 134.6 (d, *J* = 3.0 Hz), 130.0, 129.4, 125.2, 124.8, 124.6 (q, *J* = 278.0 Hz, CF_3), 124.0, 122.0, 33.6 (q, *J* = 32.2 Hz, CH_2CF_3), 21.8, ^{19}F NMR (376 MHz, $CDCl_3$), δ -65.1 (t, *J* = 10.2 Hz, 3F), HRMS (ESI), *m/z* calcd for $C_{18}H_{13}F_3OS$ $[M+H]^+$ 335.0712, found 335.0721.

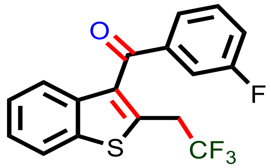

(3-Fluorophenyl)(2-(2,2,2-trifluoroethyl)benzo[*b*]thiophen-3-yl)methanone (4d): Light yellow viscous liquid, yield (23.7 mg, 70%), ^{1}H-NMR (400 MHz, $CDCl_3$), δ 7.85 (d, *J* = 8.0 Hz, 1H), 7.55-7.52 (m, 2H), 7.43-7.35 (m, 2H), 7.32-7.26 (m, 3H), 3.83 (q, *J* = 10.2 Hz, 2H), ^{13}C NMR (100 MHz, $CDCl_3$), δ 191.8, 162.7 (d, *J* = 249 3Hz), 139.9 (d, *J* = 6.4 Hz), 139.0, 137.7, 136.8 (d, *J* = 2.8 Hz), 130.5 (d, *J* = 7.6 Hz), 125.8 (d, *J* = 2.8 Hz), 125.4, 125.1, 124.5 (q, *J* = 277.9 Hz, CF_3), 123.8, 122.1, 120.8 (d, *J* = 21.5 Hz), 116.1 (d, *J* = 22.4 Hz), 144.0, 33.5 (q, *J* = 32.2 Hz, CH_2CF_3), ^{19}F NMR (376 MHz, $CDCl_3$), δ -65.1 (t, *J* = 10.2 Hz, 3F) -111.2 to -111.3 (m, 1F), HRMS (ESI), *m/z* calcd for $C_{17}H_{10}F_4OS$ $[M+H]^+$ 339.0461, found 339.0447.

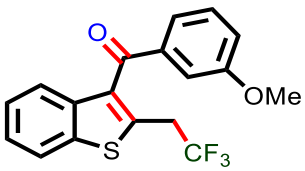

(3-Methoxyphenyl)(2-(2,2,2-trifluoroethyl)benzo[*b*]thiophen-3-yl)methanone (4e): Pale yellow viscous liquid, yield (24 mg, 69%), ^{1}H-NMR (400 MHz, $CDCl_3$), δ 7.84 (d, *J* = 8.3 Hz, 1H), 7.43-7.40 (m, 1H), 7.37-7.26 (m, 5H), 7.17-7.14 (m, 1H), 3.84-3.79 (m, 5H), ^{13}C NMR (100 MHz, $CDCl_3$), δ 192.9, 159.9, 139.1, 138.8, 138.0, 136.5, 135.1, 129.7, 125.3, 124.9, 124.6 (q, *J* = 277.7 Hz, CF_3), 124.0, 122.9, 122.0, 120.5, 113.3, 55.5, 33.6 (q, *J* = 31.8 Hz, CH_2CF_3), ^{19}F NMR (376 MHz, $CDCl_3$), δ -65.1 (t, *J* = 10.2 Hz, 3F), HRMS (ESI), *m/z* calcd for $C_{18}H_{13}F_3O_2S$ $[M+H]^+$ 351.0661, found 351.0640

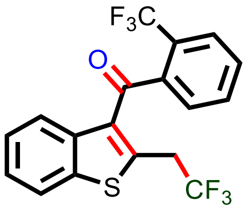

(2-(2,2,2-Trifluoroethyl)benzo[*b*]thiophen-3-yl)(2-(trifluoromethyl)phenyl)methanone (4f): Yellow viscous liquid, yield (22.5 mg, 58%), ^{1}H-NMR (400 MHz, $CDCl_3$), δ 7.88 (d, *J* = 7.8, Hz, 1H), 7.82 (d, *J* = 8.1 Hz, 1H), 7.66 (t, *J* = 7.8 Hz, 1H), 7.56 (t, *J* = 7.6 Hz, 1H), 7.39 (d, *J* = 7.6 Hz, 1H), 7.33 (td, *J* = 7.6, 1.0 Hz, 1H), 7.20 (td, *J* = 7.6 Hz, 0.9 Hz, 1H), 7.10 (d, *J* = 8.2 Hz, 1H), 4.02 (q, *J* = 10.2 Hz, 2H), ^{13}C NMR (100 MHz, $CDCl_3$), δ 191.0, 140.8 (q, *J* = 3.1 Hz), 139.2 (q, *J* = 1.8 Hz), 138.4, 137.3, 134.4, 132.0, 131.3, 129.5, 127.4 (q, *J* = 5.2 Hz), 125.4 (d, *J* = 1.1 Hz), 124.5 (q, *J* = 277.8 Hz, CF_3), 123.7, 122.1, 33.5(q, *J* = 32.2 Hz, CH_2CF_3), ^{19}F NMR (376 MHz, $CDCl_3$), δ -64.8 (t, *J* = 10.2 Hz, 3F), HRMS (ESI), *m/z* calcd for $C_{18}H_{10}F_6OS$ $[M+Na]^+$ 411.0249, found 411.0225.

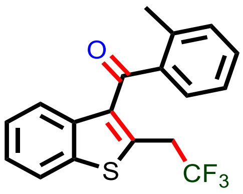

***o*-Tolyl(2-(2,2,2-trifluoroethyl)benzo[*b*]thiophen-3-yl)methanone (4g):** Light yellow viscous liquid, yield (19.1 mg, 57%), ^{1}H-NMR (400 MHz, $CDCl_3$), δ 7.82 (d, *J* = 8.1 Hz, 1H), 7.43 (t, *J* =

7.4 Hz, 1H), 7.35-7.32 (m, 3H), 7.28-7.22 (m, 3H), 7.18 (t, *J* = 7.7 Hz, 1H), 3.85 (q, *J* = 10.2 Hz, 2H), 2.50 (s, 3H), ^{13}C NMR (100 MHz, $CDCl_3$), δ 194.2, 139.3, 138.6, 138.5, 138.4, 137.8, 137.0, 132.1, 131.9, 130.5, 126.0, 125.3, 125.1, 124.7 (q, *J* = 278.4 Hz, CF_3), 123.9, 122.0, 33.5 (q, *J* = 32.0 Hz, CH_2CF_3), 20.4, ^{19}F NMR (376 MHz, $CDCl_3$), δ -64.9 (t, *J* = 10.2 Hz, 3F), HRMS (ESI), *m/z* calcd for $C_{18}H_{13}F_3OS$ $[M+H]^+$ 335.0712, found 335.0687.

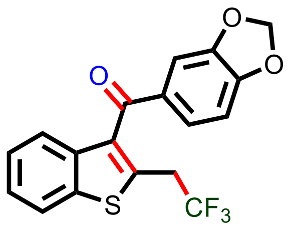

Benzo[*d*][1,3]dioxol-5-yl(2-(2,2,2-trifluoroethyl)benzo[*b*]thiophen-3-yl)methanone (4h): Yellow viscous liquid, yield (19.9 mg, 52%), ^{1}H-NMR (400 MHz, $CDCl_3$), δ 7.83 (d, *J* = 8.0, Hz, 1H), 7.39-7.27 (m, 5H), 6.79 (d, *J* = 8.1 Hz, 1H), 6.06 (s, 2H), 3.79 (q, *J* = 10.2 Hz, 2H), ^{13}C NMR (100 MHz, $CDCl_3$), δ 191.2, 152.6, 148.4, 139.1, 138.0, 136.8, 132.4, 127.3, 125.3, 124.9, 124.6 (q, *J* = 277.6 Hz, CF_3), 123.9, 122.0, 114.0, 108.9, 108.1, 102.0, 33.6 (q, *J* = 32.0 Hz, CH_2CF_3), ^{19}F NMR (376 MHz, $CDCl_3$), δ -65.1 (t, *J* = 10.2 Hz, 3F), HRMS (ESI), *m/z* calcd for $C_{18}H_{11}F_3O_3S$ $[M+H]^+$ 365.0454, found 365.0447.

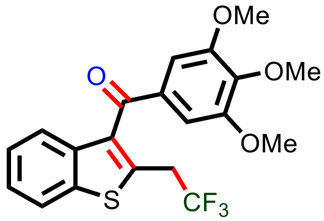

(2-(2,2,2-Trifluoroethyl)benzo[*b*]thiophen-3-yl)(3,4,5-trimethoxyphenyl)methanone (4i): Light yellow viscous liquid, yield (18.1 mg, 44%), ^{1}H-NMR (500 MHz, $CDCl_3$), δ 7.89 (d, *J* = 8.2, Hz, 1H), 7.45 (d, *J* = 8.2 Hz, 1H), 7.41 (td, *J* = 7.6, 1.2 Hz, 1H), 7.33 (td, *J* = 7.6, 1.0 Hz, 1H), 7.11 (s, 2H), 3.98 (s, 3H), 3.85 (q, *J* = 10.5 Hz, 2H), 3.80 (s, 6H), ^{13}C NMR (176 MHz, $CDCl_3$), δ 191.9, 153.2, 143.2, 138.8, 137.9, 136.4, 134.7 (q, *J* = 3.2 Hz), 132.7, 125.4, 124.9, 124.6 (q, *J* = 277.2 Hz, CF_3), 124.0, 122.0, 107.3, 61.0, 56.3, 33.7 (q, *J* = 32.7 Hz, CH_2CF_3), ^{19}F NMR (470 MHz, $CDCl_3$), δ -65.0 (t, *J* = 10.3 Hz, 3F), HRMS (ESI), *m/z* calcd for $C_{20}H_{17}F_3O_4S$ $[M+H]^+$ 411.0872, found 411.0873.

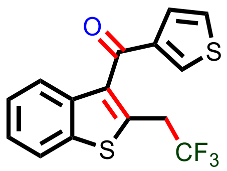

Thiophen-3-yl(2-(2,2,2-trifluoroethyl)benzo[*b*]thiophen-3-yl)methanone (4j): Pale yellow viscous liquid, yield (20.6 mg, 63%), ^{1}H-NMR (500 MHz, $CDCl_3$), δ 7.92 (dd, *J* = 2.9, 1.2 Hz, 1H), 7.88 (d, *J* = 8.1 Hz, 1H), 7.58 (dd, *J* = 5.2, 1.1 Hz, 1H), 7.52 (d, *J* = 8.1 Hz, 1H), 7.43-7.39 (m, 2H), 7.34 (td, *J* = 7.6, 0.9 Hz, 1H), 3.87 (q, *J* = 10.2 Hz, 2H), ^{13}C NMR (125 MHz, $CDCl_3$), δ 186.3, 142.6, 138.9, 137.8, 137.1, 135.5, 134.4 (q, *J* = 3.0 Hz), 127.6, 126.7, 125.4, 124.9, 124.7 (q, *J* = 278.5 Hz, CF_3), 123.8, 122.0, 33.6 (q, *J* = 31.9 Hz, CH_2CF_3), ^{19}F NMR (376 MHz, $CDCl_3$), δ -65.1 (t, *J* = 10.3 Hz, 3F), HRMS (ESI), *m/z* calcd for $C_{15}H_9F_3OS_2$ $[M+H]^+$ 327.0120, found 327.0118.

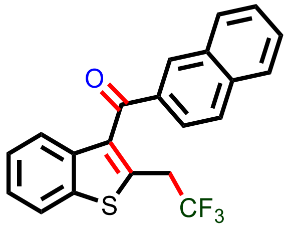

Naphthalen-2-yl(2-(2,2,2-trifluoroethyl)benzo[*b*]thiophen-3-yl)methanone (4k): Yellow solid, yield (25.2 mg, 68%), ^{1}H-NMR (400 MHz, $CDCl_3$), δ 8.25 (s 1H), 7.97-7.83 (m, 5H), 7.62 (td, *J* = 7.6, 1.0 Hz, 1H), 7.52 (td, *J* = 7.6, 1.0 Hz, 1H), 7.38-7.34 (m, 2H), 7.22 (td, *J* = 7.6, 0.9 Hz, 1H), 3.85 (q, *J* = 10.2 Hz, 2H), ^{13}C NMR (125 MHz, $CDCl_3$), δ 193.0, 138.9, 138.1, 136.7, 136.0, 135.1, 132.4 132.3, 129.8, 128.9, 128.8, 127.9, 126.9, 125.3, 125.0, 124.9 (q, *J* = 278.8 Hz, CF_3), 124.7, 124.0, 122.1, 33.7, (q, *J* = 32.5 Hz, CH_2CF_3), ^{19}F NMR (376 MHz, $CDCl_3$), δ -65.0 (t, *J* = 10.2 Hz, 3F), HRMS (ESI), *m/z* calcd for $C_{21}H_{13}F_3OS$ $[M+H]^+$ 371.0712, found 371.0689.

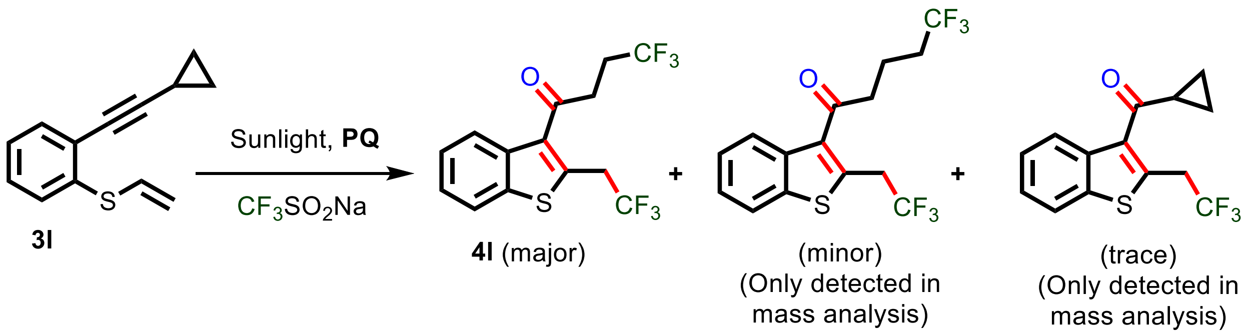

4,4,4-trifluoro-1-(2-(2,2,2-trifluoroethyl)benzo[b]thiophen-3-yl)butan-1-one (4l): Light yellow viscous liquid, yield (11.7 mg, 41%), ^{1}H-NMR (400 MHz, $CDCl_3$), δ 7.90 (t, *J* = 6.8 Hz, 2H), 7.53 (t, *J* = 7.8 Hz, 1H), 7.47 (td, *J* = 7.8, 1.1, 1H), 4.01 (q, *J* = 10.2 Hz, 2H), 3.29 (t, *J* = 7.5 Hz, 2H), 2.74-2.64 (m, 2H), ^{13}C NMR (100 MHz, $CDCl_3$), δ 196.3, 139.3, 138.9, 136.2, 136.1, 126.8 (q, *J* = 278.1 Hz, CF_3) 125.7, 125.5, 124.5 (q, *J* = 278.1 Hz, CF_3), 122.9, 122.6, 114.0, 33.5 (q, *J* = 32.0 Hz, CH_2CF_3), 29.1, 28.3 (*J* =30.1 Hz, CH_2CF_3) ^{19}F NMR (376 MHz, $CDCl_3$), δ -65.1 (t, *J* = 10.2 Hz, 3F), δ -66.3 (t, *J* = 10.4 Hz, 3F), HRMS (ESI), *m/z* calcd for $C_{14}H_{10}OSF_6$ $[M+H]^+$ 341.0429 found 341.0408.

5,5,5-trifluoro-1-(2-(2,2,2-trifluoroethyl)benzo[b]thiophen-3-yl)pentan-1-one (minor): HRMS (ESI), *m/z* calcd for $C_{15}H_{12}OSF_6$ $[M+H]^+$ 355.0586 found 355.0574.

Cyclopropyl(2-(2,2,2-trifluoroethyl)benzo[b]thiophen-3-yl)methanone (trace): HRMS (ESI), *m/z* calcd for $C_{14}H_{11}OSF_3$ $[M+H]^+$ 285.0555 found 285.0572.

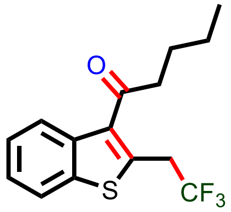

1-(2-(2,2,2-trifluoroethyl)benzo[*b*]thiophen-3-yl)ethan-1-one (4m): Light brown viscous liquid, yield (10.8 mg, 36%), ^{1}H-NMR (500 MHz, $CDCl_3$), δ 7.91 (d, *J* = 8.2 Hz, 1H), 7.87 (d, *J* = 7.9 Hz, 1H), 7.48 (t, *J* = 7.5 Hz, 1H), 7.44 (t, *J* = 7.5 Hz, 1H), 3.98 (q, *J* = 10.2 Hz, 2H), 3.01 (t, *J* = 7.5 Hz, 2H), 1.82-1.76 (m, 2H), 1.43-1.40 (m, 2H), 0.98 (t, *J* = 7.4 Hz, 3H), ^{13}C NMR (100 MHz, $CDCl_3$), δ 201.0, 138.9, 137.7, 136.6, 136.3, 125.3 (d, *J* = 4.5 Hz), 124.8(q, *J* = 278.9 Hz, CF_3), 123.2, 122.4, 116.1, 33.5 (q, *J* = 32.0 Hz, CH_2CF_3), 30.3, 26.3, 22.4, 19.3, ^{19}F NMR (376 MHz, $CDCl_3$), δ -65.0 (t, *J* = 10.3 Hz, 3F), HRMS (APCI), *m/z* calcd for $C_{15}H_{15}F_3OS$ $[M+H]^+$ 301.0868, found 301.0855.

General Experimental Procedure for Phenyl(2-(2,2,2-trifluoroethyl)-1H-indol-3-yl)methanone (5a)

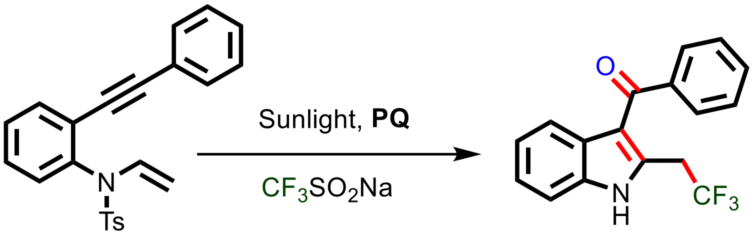

5a **6a**

4-Methyl-*N*-(2-(phenylethynyl)phenyl)-*N*-vinylbenzenesulfonamide (**5a**) (22 mg, 0.1 mmol, 1.0 equiv.), CF_3SO_2Na (47 mg, 0.6 mmol), and photocatalyst (**PQ**) (2.1 mg, 0.01 mmol, 0.1 equiv.) were added to a 5 mL round bottom flask with magnetic stir bar. The flask was evacuated and backfilled with argon and then CH_3CN + H_2O (900 + 100 µL) was added to the mixture. The reaction mixture was stirred up to 4-6 h under sunlight. The progress of the reaction was monitored by TLC. After completion of the reaction, the solvent was removed on rotary evaporator under vacuum. The residue was washed with 1N NaOH (2 mL) and extracted with diethyl ether (3 x 5 mL). The combined organic layers were washed with brine (10 mL), dried over Na_2SO_4, and the residue was purified by flash column chromatography on silica gel using n-Hexane: Dichloromethane (9: 1) to afford the desired product **6a**.

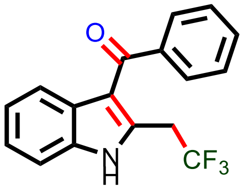

Phenyl(1-tosyl-2-(2,2,2-trifluoroethyl)-1H-indol-3-yl)methanone (6a): Yellow viscous liquid, yield (20.3 mg, 67%), ^{1}H-NMR (500 MHz, $CDCl_3$), δ 8.91 (brs, 1H), 7.83 (dd, *J* = 8.4, 1.3 Hz, 2H), 7.62 (tt, *J* = 7.4, 1.2 Hz, 1H), 7.50 (t, *J* = 7.6 Hz, 2H), 7.46 (d, *J* = 8.2 Hz, 1H), 7.28-7.26 (m, 1H), 7.18 (d, *J* = 8.1 Hz, 1H), 7.11-7.08 (m, 1H), 4.09 (q, *J* = 10.6 Hz, 2H), ^{13}C NMR (125 MHz, $CDCl_3$), δ 192.9, 140.2, 136.0, 135.1, 132.2, 130.5, 129.6, 129.2, 128.3, 126.4, 125.1(q, *J* = 277.0 Hz, CF_3), 123.9, 123.5, 121.7, 121.3, 116.7, 111.2, 31.9 (q, *J*.= 31.1 Hz, CH_2CF_3), ^{19}F NMR (470.5MHz, $CDCl_3$), δ -63.8 (t, *J* = 10.4 Hz, 3F), HRMS (ESI), *m/z* calcd for $C_{17}H_{12}F_3NO$ $[M+H]^+$ 304.0944, found 304.0944.

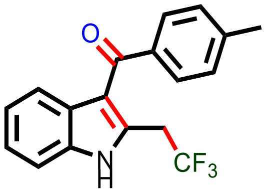

***p*-Tolyl(1-tosyl-2-(2,2,2-trifluoroethyl)-1H-indol-3-yl)methanone (6b):** Brown viscous liquid, yield (19.7 mg, 62%), ^{1}H-NMR (500 MHz, $CDCl_3$), δ 8.92 (brs, 1H), 7.75 (d, *J* = 8.1 Hz, 2H), 7.44 (d, *J* = 8.2 Hz, 1H), 7.30 (d, *J* = 9.0 Hz, 2H), 7.27-7.23 (m, 2H), 7.10 (t, *J* = 7.6 Hz, 1H), 4.09 (q, *J* = 10.7 Hz, 2H), 2.48 (s, 3H), ^{13}C NMR (125 MHz, $CDCl_3$), δ 192.6, 143.0, 137.4, 135.1, 129.5, 129.0, 126.5, 125.0 (q, *J* = 277.5 Hz, CF_3), 123.4, 121.6, 121.4, 116.9, 111.2, 31.8 (q, *J*.= 31.3 Hz, CH_2CF_3), 21.7, ^{19}F NMR (470.5MHz, $CDCl_3$), δ -63.9 (t, *J* = 10.4 Hz, 3F), HRMS (ESI), *m/z* calcd for $C_{18}H_{14}F_3NO$ $[M+Na]^+$ 340.0920, found 340.0932.

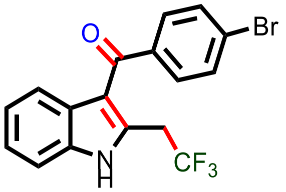

(4-Bromophenyl)(1-tosyl-2-(2,2,2-trifluoroethyl)-1H-indol-3-yl)methanone (6c): Yellow solid, yield (22.9 mg, 60%), ^{1}H-NMR (500 MHz, $CDCl_3$), δ 9.32 (brs, 1H), 7.71 (d, *J* = 8.4 Hz, 2H), 7.64 (d, *J* = 8.4 Hz, 2H), 7.45 (d, *J* = 8.3 Hz, 1H), 7.55 (d, *J* = 7.5 Hz, 1H), 7.16-7.10 (m, 2H), 4.09 (q, *J* = 10.5 Hz, 2H), ^{13}C NMR (125 MHz, $CDCl_3$), δ 191.6, 138.8, 135.1, 131.7, 130.9, 127.2, 126.2, 124.9 (q, *J* = 278.0 Hz, CF_3), 123.7, 121.9, 121.1, 116.2, 111.4, 31.9 (q, *J*.= 31.3 Hz, CH_2CF_3), ^{19}F NMR (470.5MHz, $CDCl_3$), δ -63.9 (t, *J* = 10.5 Hz, 3F), HRMS (ESI), *m/z* calcd for $C_{17}H_{11}BrF_3NO$ $[M+H]^+$ 382.0049, found 382.0041.

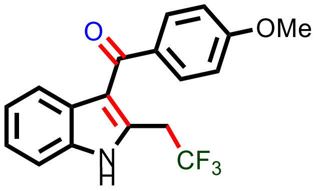

(4-methoxyphenyl)(2-(2,2,2-trifluoroethyl)-1H-indol-3-yl)methanone (6d): Yellow semi-solid, yield (21.0 mg, 63%), ^{1}H-NMR (500 MHz, $CDCl_3$), δ 8.94 (bs, 1H), 7.82 (d, *J* = 8.8 Hz, 2H), 7.40 (d, *J* = 8.0 Hz, 1H), 7.25-7.21 (m, 2H), 7.07 (t, *J* = 7.9 Hz, 1H), 6.94 (d, *J* = 8.8 Hz, 2H), 4.00 (q, *J* = 10.6 Hz, 2H), 3.88 (s, 3H), ^{13}C NMR (125 MHz, $CDCl_3$), δ 191.6, 163.1, 135.1, 132.6, 131.8, 126.5, 125.1(q, *J* = 277.9 Hz, CF_3), 123.4, 121.5, 121.3, 117.0, 113.6, 111.2, 55.4, 31.8 (q, *J* = 31.2 Hz, CH_2CF_3), ^{19}F NMR (375 MHz, $CDCl_3$), δ -63.9 (t, *J* = 10.7 Hz, 3F), HRMS (ESI), *m/z* calcd for $C_{18}H_{14}F_3NO_2$ $[M+H]^+$ 334.1049, found 334.1058.

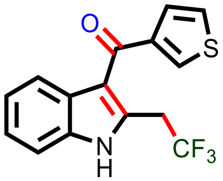

Thiophen-3-yl(1-tosyl-2-(2,2,2-trifluoroethyl)-1H-indol-3-yl)methanone (6e): Brown viscous liquid, yield (17.3 mg, 56%), ^{1}H-NMR (400 MHz, $CDCl_3$), δ 9.02 (brs, 1H), 7.95 (dd, *J* = 3.0, 1.0 Hz, 1H), 7.73-7.67 (m, 1H), 7.54 (dd, *J* = 5.0, 0.8 Hz, 1H), 7.41 (t, *J* = 7.5 Hz, 2H), 7.24-7.20 (m, 1H), 7.11 (t, *J* = 8.0 Hz, 1H), 4.03 (q, *J* = 10.6 Hz, 2H), ^{13}C NMR (100 MHz, $CDCl_3$), δ 186.1, 143.6, 136.1, 135.2, 132.9, 130.5, 129.6, 128.1, 126.0, 125.0 (q, *J* = 280.0 Hz, CF_3), 123.5, 121.7, 121.1, 111.3, 31.8 (q, *J*.= 31.4 Hz, CH_2CF_3), ^{19}F NMR (376.0 MHz, $CDCl_3$), δ -63.9 (t, *J* = 10.4 Hz, 3F), HRMS (ESI), *m/z* calcd for $C_{15}H_{10}F_3NOS$ $[M+H]^+$ 310.0508, found 310.0519.

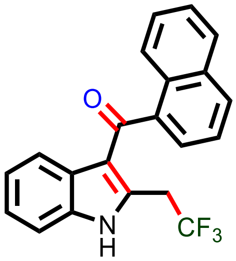

Naphthalen-1-yl(2-(2,2,2-trifluoroethyl)-1H-indol-3-yl)methanone (6f): Yellow -solid, yield (22.6 mg, 64%), ^{1}H-NMR (400 MHz, $CDCl_3$), δ 9.17 (brs, 1H), δ 8.12 (d, *J* = 8.3 Hz, 1H), 8.01 (d, *J* = 8.2 Hz, 1H), 7.93 (d, *J* =8.1 Hz, 1H), 7.61 (d, *J* = 7.11 Hz, 1H), 7.55-7.48 (m, 2H), 7.45 (t, *J* = 7.5 Hz, 1H), 7.32 (d, *J* = 8.2 Hz, 1H), 7.15 (t, *J* = 7.6 Hz, 1H), 6.91 (t, *J* = 7.6 Hz, 1H), 6.75 (d, *J* = 8.2 Hz, 1H), 4.07 (q, *J* = 10.6 Hz, 2H), ^{13}C NMR (100 MHz, $CDCl_3$), δ 194.0, 139.0, 136.1, 135.2, 134.4, 133.7, 130.9, 130.1, 128.3, 127.2, 126.5, 126.3, 125.3, 125.1 (q, *J* = 277.5 Hz, CF_3), 124.8, 123.5, 122.1, 121.2, 117.3, 111.3, 32.0 (q, *J* = 31.2, CH_2CF_3), ^{19}F NMR (376.0 MHz, $CDCl_3$), δ -63.7 (t, *J* = 10.5 Hz, 3F), HRMS (ESI), *m/z* calcd for $C_{21}H_{14}F_3NO$ $[M+H]^+$ 354.1100, found 354.1082.

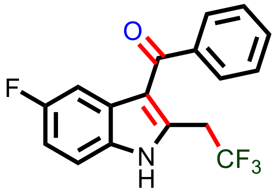

(5-fluoro-2-(2,2,2-trifluoroethyl)-1H-indol-3-yl)(phenyl)methanone (6g): Yellow -solid, yield (19.3 mg, 60%), ^{1}H-NMR (400 MHz, $CDCl_3$), δ 9.06 (s, 1H), 7.76 (d, *J* = 7.8 Hz, 2H), 7.60 (t, *J* = 7.4 Hz, 1H), 7.48 (t, *J* = 7.7 Hz, 2H), 7.33 (dd, *J* = 8.8, 4.4 Hz, 1H), 6.97 (td, *J* = 8.9, 2.5 Hz, 1H), 6.78 (dd, *J* = 9.9, 2.3 Hz, 1H), 4.02 (q, *J* = 10.6 Hz, 2H), ^{13}C NMR (100 MHz, $CDCl_3$), δ 192.6, 158.8 (d, *J* = 237.8 Hz), 139.8 Hz, 139.2, 132.4, 131.6, 129.0, 128.5, 127.1 (d, *J* = 10.6 Hz), 125.2 (q, *J* = 276.2 Hz, CF_3), 116.8 (d, *J* = 4.1 Hz), 114.0, 112.2 (d, *J* = 5.9 Hz), 112.0 (d, *J* = 10.9 Hz), 106.6 (d, *J* = 25.4 Hz), 32.0 (q, *J* = 31.4 Hz, CH_2CF_3), HRMS (ESI), *m/z* calcd for $C_{17}H_{11}F_4NO$ $[M+H]^+$ 322.0850, found 322.0850.

General Experimental Procedure for *tert*-Butyl 3-benzoyl-2-(2,2,2-trifluoroethyl)-1H-indole-1-carboxylate (6h)

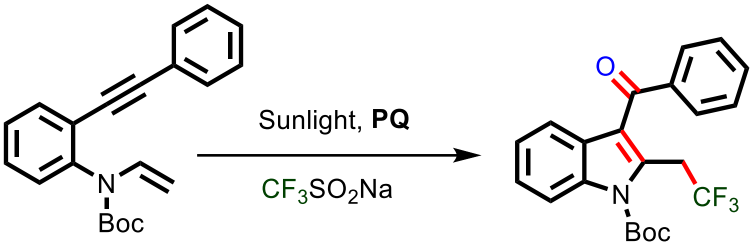

5a′ **6h**

tert-Butyl (2-(phenylethynyl)phenyl)(vinyl)carbamate (**5a′**) (22 mg, 0.1 mmol, 1.0 equiv.), CF_3SO_2Na (47 mg, 0.6 mmol), and photocatalyst (**PQ**) (2.1 mg, 0.01 mmol, 0.1 equiv.) were added to a 5 mL round bottom flask with magnetic stir bar. The flask was evacuated and backfilled with argon and then CH_3CN + H_2O (900 + 100 µL) was added to the mixture. The reaction mixture was stirred up to 4-6 h under sunlight. The progress of the reaction was monitored by TLC. After completion of the reaction, the solvent was removed on rotary evaporator under vacuum. The residue was washed with 1 N NaOH (2 mL) and extracted with diethyl ether (3 x 5 mL). The combined organic layers were washed with brine (10 mL), dried over Na_2SO_4, and the residue was purified by flash column chromatography on silica gel (n-Hexane: Dichloromethane = 9: 1) to afford the desired product **6h**.

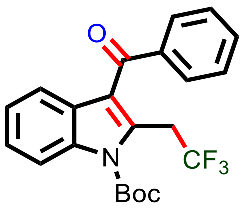

***tert*-Butyl 3-benzoyl-2-(2,2,2-trifluoroethyl)-1H-indole-1-carboxylate (6h):** Light yellow viscous liquid, yield (23.0 mg, 57%), ^{1}H-NMR (500 MHz, $CDCl_3$), δ. 8.19 (d, *J* = 8.5 Hz, 1H), 7.84 (d, *J* = 7.6 Hz, 2H), 7.64 (t, *J* = 7.5 Hz, 1H), 7.49 (t, *J* = 7.6 Hz, 2H), 7.37 (t, *J* = 8.1 Hz, 1H), 7.16 (t, *J* = 7.6 Hz, 1H), 7.08 (d, *J* = 8.1 Hz, 1H), 4.48 (d, *J* = 10.1 Hz, 2H), 1.76 (s, 9H), ^{13}C NMR (125 MHz, $CDCl_3$), δ 193.1, 149.7, 138.7, 136.0, 133.2, 132.1, 129.6, 128.5, 127.1, 125.3,124.9, (q, *J* = 278.1 Hz, CF_3), 123.4, 123.2, 121.0, 115.5, 86.0, 30.7 (q, *J* = 31.3 Hz, CH_2CF_3), 28.0, ^{19}F NMR (470 MHz, $CDCl_3$), δ -64.3 (t, *J* = 10.4 Hz, 3F), HRMS (ESI), *m/z* calcd for $C_{22}H_{20}F_3NO_3$ $[M+H]^+$ 426.1287, found 426.1269.

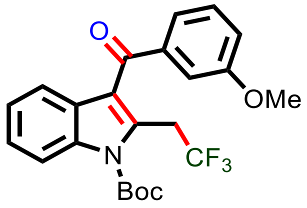

***tert*-Butyl 3-(3-methoxybenzoyl)-2-(2,2,2-trifluoroethyl)-1H-indole-1-carboxylate (6i):** White viscous liquid, yield (23.8 mg, 55%), ^{1}H-NMR (400 MHz, $CDCl_3$), δ. 8.13 (d, *J* = 8.5 Hz, 1H), 7.38 (s, 1H), 7.35-7.30 (m, 3H), 7.16-7.08 (m, 3H), 4.42 (q, *J* = 10.2 Hz, 2H), 3.81 (s, 3H), 1.71(s, 9H), ^{13}C NMR (100 MHz, $CDCl_3$), δ 192.9, 159.8, 149.7, 140.0, 135.9, 132.0, 129.5, 127.1, 125.3, 123.4, 123.2, 122.7, 122.5 (q, *J* = 278.3 Hz, CF_3), 121.0, 120.0, 115.5, 113.3, 86.1, 55.3, 30.7 (q, *J* = 31.8 Hz, CH_2CF_3), 28.0, ^{19}F NMR (470 MHz, $CDCl_3$), δ -64.3 (t, *J* = 10.3 Hz, 3F), HRMS (ESI), *m/z* calcd for $C_{23}H_{22}F_3NO_4$ $[M+H]^+$ 434.1574, found 434.1575.

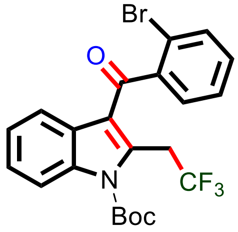

(2-Bromophenyl)(2-(2,2,2-trifluoroethyl)-1H-indol-3-yl)methanone (6j): Light yellow viscous liquid, yield (24.1 mg, 50%), ^{1}H-NMR (400 MHz, $CDCl_3$), δ. 8.09 (d, *J* = 8.6 Hz, 1H), 7.40-7.38 (m, 2H), 7.30-7.26 (m, 2H), 7.16 (d, *J* = 8.0 Hz, 1H), 7.13-7.10 (m, 1H), 7.07 (d, *J* = 7.5 Hz, 1H), 4.66 (q, *J* = 10.2 Hz, 2H), 1.71(s, 9H), ^{13}C NMR (100 MHz, $CDCl_3$), δ 191.9, 149.5, 141.9, 139.2, 136.2, 133.7, 131.9, 129.6, 128.1, 127.6, 126.4 (q, *J* = 277.7 Hz, CF_3), 125.3, 123.9, 120.4, 119.6, 115.3, 114.0, 86.5, 30.5 (q, *J* = 31.3 Hz, CH_2CF_3), 27.9, ^{19}F NMR (470 MHz, $CDCl_3$), δ -63.59 (t, *J* = 10.2 Hz, 3F), HRMS (ESI), *m/z* calcd for $C_{22}H_{19}BrF_3NO_3$ $[M+H]^+$ 482.0573, found 482.0572.

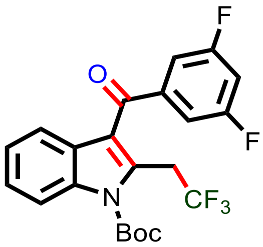

***tert*-Butyl 3-(3,5-difluorobenzoyl)-2-(2,2,2-trifluoroethyl)-1H-indole-1-carboxylate (6k)**: Light brown viscous liquid, trace, > 10%, ^{19}F NMR (470 MHz, $CDCl_3$), δ -64.4 (t, *J* = 10.3 Hz, 3F), -107.7 to -107.8 (m, 2F), HRMS (ESI), *m/z* calcd for $C_{22}H_{18}F_5NO_3$ $[M+H]^+$ 440.1280, found 440.1261.

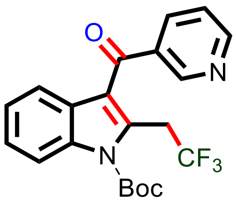

Pyridin-3-yl(2-(2,2,2-trifluoroethyl)-1H-indol-3-yl)methanone (6l): Light yellow viscous liquid, yield (>20%, crude), ^{19}F NMR (470 MHz, $CDCl_3$), δ -64.4 (t, *J* = 10.1 Hz, 3F), HRMS (ESI), *m/z* calcd for $C_{21}H_{19}F_3N_2O_3$ $[M+H]^+$ 405.1421, found 405.1433.

Further Functionalization

Scheme S13. Synthesis of (3,5-Dibromo-4-hydroxyphenyl)(2-(2,2,2-trifluoroethyl)benzofuran-3-yl)methanone (**7a**)

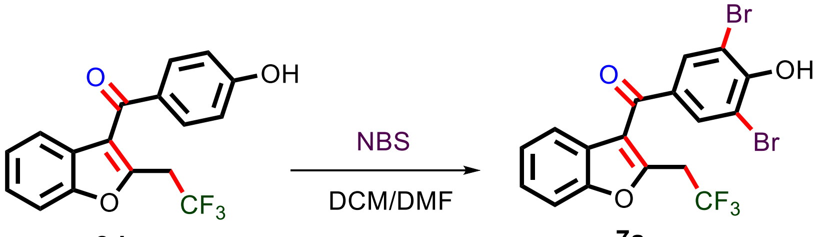

(3,5-Dibromo-4-hydroxyphenyl)(2-(2,2,2-trifluoroethyl)benzofuran-3-yl)methanone (**7a**) was prepared by the literature procedures.[13] The following representative procedures

To a stirred solution of NBS (0.40 mmol, 72 mg) in dichloromethane (9 mL) was diluted with DMF (0.33 mL) at –10 ^{0}C and stirred for 10 min, then (4-hydroxyphenyl)(2-(2,2,2-trifluoroethyl)benzofuran-3-yl)methanone **2d** (0.20 mmol, 62 mg) in dichloromethane (1 mL) was added. The reaction mixture was allowed to warm to room temperature and stirred for 17 h. After

completion, the reaction was quenched with H_2O (10 mL x 4) and extracted with dichloromethane. The organic layer was washed with brine (50 mL), dried over Na_2SO_4 and concentrated under reduced pressure. The crude product was purified by silica gel column chromatography hexane/ EtOAc (95/5) to afford desired product **7a**. White viscous liquid, yield (39.5 mg, 40%), ^{1}H-NMR (400 MHz, $CDCl_3$), δ. 7.99 (s, 2H), 7.58 (d, *J* = 8.4 Hz, 1H), 7.41-7.36 (m, 1H), 7.29-7.25 (m, 2H), 3.91 (q, *J* = 9.9 Hz, 2H), ^{13}C NMR (100 MHz, $CDCl_3$), δ 187.0, 154.2, 153.7, 139.2, 133.6, 132.7, 125.9, 125.4, 125.0 (q, *J* = 278.1 Hz, CF_3), 124.3, 121.2, 119.7, 114.0, 111.9, 110.2, 32.8 (q, *J* = 32.4 Hz, CH_2CF_3), ^{19}F NMR (376 MHz, $CDCl_3$), δ -63.8 (t, *J* = 10.0 Hz, 3F), HRMS (ESI), *m/z* calcd for $C_{17}H_9Br_2F_3O_3$ $[M+H]^+$ 476.8943, found 476.8945.

Scheme S14. Synthesis of Naphthalen-1-yl(1-propyl-2-(2,2,2-trifluoroethyl)-1H-indol-3-yl)methanone (**7b**)

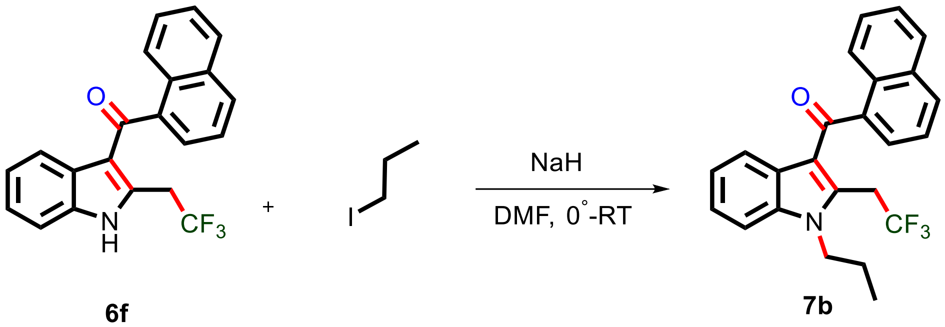

6f **7b**

Naphthalen-1-yl(1-propyl-2-(2,2,2-trifluoroethyl)-1H-indol-3-yl)methanone (**7b**) was prepared by the following literature procedures.[14]

To a stirred solution of naphthalen-1-yl(2-(2,2,2-trifluoroethyl)-1H-indol-3-yl)methanone **6f** (48.0 mg, 0. 12 mmol) in dry DMF (1.0 mL), NaH (3.84 mg, 60% suspension in mineral oil, 0.16 mmol) was added portionwise under nitrogen atmosphere at 0 °C. The reaction mixture was then warmed to room temperature and stirred for 2-3 min. After cooling to 0 °C, 1-iodopropane (30 µL, 0.30 mmol) was added dropwise to the reaction mixture. The reaction mixture was warmed to room temperature and stirred overnight. Water was added (5 mL) and the aqueous layer was extracted with ether (10 mL x 3). The combined organic layers were washed with brine, dried over anhydrous Na_2SO4 and concentrated under reduced pressure. The residue was purified by column chromatography on silica gel (*n*-hexane/ethyl acetate = 15/1) to give compound **7a** as a clear oil (29.0 mg, 54% yield). ^{1}H-NMR (500 MHz, $CDCl_3$), δ 8.17 (d, *J* = 8.6 Hz, 1H), 8.06 (d, *J* = 8.3

Hz, 1H), 7.97 (d, J =8.2 Hz, 1H), 7.69 (d, J = 7.9 Hz, 1H), 7.56-7.54 (m, 1H), 7.43 (d, J = 8.2 Hz, 1H), 7.22 (t, J = 7.5 Hz, 1H), 7.03 (dd, J = 7.9 Hz, 1H), 6.96 (t, J = 7.6 Hz, 1H), 6.80 (d, J = 8.0 Hz, 1H), 6.70 (t, J = 7.7 Hz, 1H), 4.17 (q, J = 10.7 Hz, 2H), 4.09 (t, J = 7.5 Hz, 2H), 1.82-1.78 (m, 2H), 0.91 (t, J = 7.2 Hz, 3H), ^{13}C NMR (125.7 MHz, $CDCl_3$), δ 193.7, 139.2, 136.6, 134.8, 133.0, 132.7, 130.9, 130.0, 128.3, 127.2, 126.6, 126.4, 125.3, 125.0 (q, J = 278.2 Hz, CF_3), 124.8, 123.6, 122.2, 121.3, 114.0, 111.1, 33.8, 31.7 (q, J = 31.2, CH_2CF_3), 24.8, 19.7 ^{19}F NMR (476.0 MHz, $CDCl_3$), δ -63.5 (t, J = 10.5 Hz, 3F), HRMS (ESI), m/z calcd for $C_{24}H_{20}F_3NO$ $[M-H]^+$ 394.1413, found 394.1419.

Computational Methods: All computations were performed with the GAUSSIAN 09 Revision A.02 program suite[15] with the DFT method of Becke's three parameter hybrid Hartree-Fock procedure with the Lee-Yang-Parr correlation function (B3LYP). The geometry optimization and energy calculations of the reactants, intermediates, and transition state in this study were fully optimized by the DFT/B3LYP method with the 6-31+G(d) basis set in solution phase using CPCM (Conductor-like Polarizable Continuum Model) model in acetonitrile (frequency calculations were done to validate the results).

Scheme S15. Proposed Mechanism

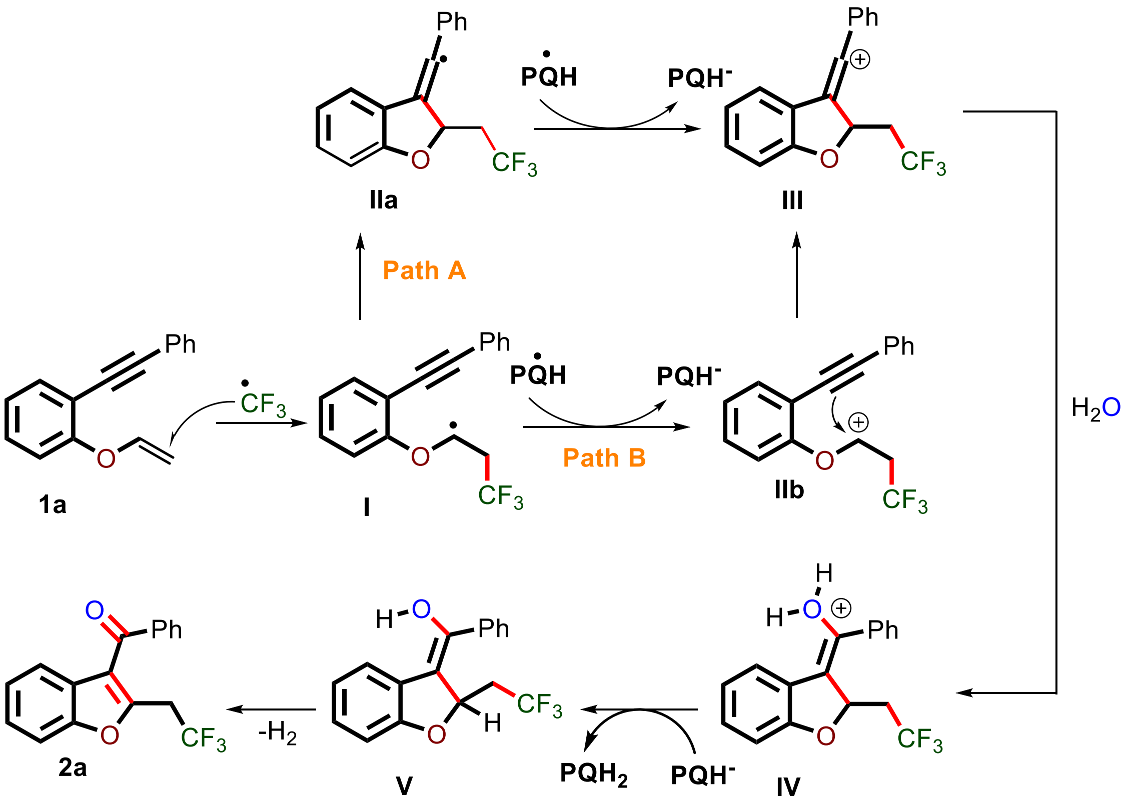

Figure, Gibbs Free Energy, and Cartesian Coordinate of Optimized Structures

1a

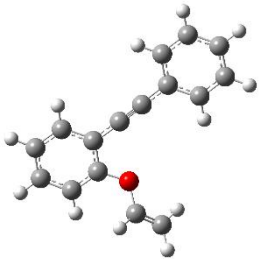

Gibbs free energy = -691.917416

Symbol	X	Y	Z
C	-4.6906640	-1.2372410	-0.3620370
C	-3.9898880	-2.2559760	0.3166900
C	-2.6199220	-2.1605330	0.5588770
C	-1.9470090	-1.0127620	0.1088850
C	-2.6748890	-0.0189510	-0.5619350
C	-4.0390970	-0.0900560	-0.8196930
H	-5.7579460	-1.3456310	-0.5326890
H	-4.5319790	-3.1329270	0.6594620
H	-2.0891300	-2.9460600	1.0865590
H	-4.5626620	0.7016020	-1.3459880
C	-0.5691300	-0.5482190	0.1209680
C	0.5513920	-1.2511430	0.7943040
C	1.9078000	-1.2888560	0.1716240
C	3.0329480	-1.4222720	1.0049430
C	2.0806080	-1.2687710	-1.2232290
C	4.3095500	-1.5109940	0.4527800
H	2.8942130	-1.4438890	2.0815570
C	3.3591740	-1.3805740	-1.7745000
H	1.2169240	-1.1916510	-1.8772010
C	4.4743180	-1.4939110	-0.9384180
H	5.1761060	-1.5956590	1.1026780
H	3.4832810	-1.3798010	-2.8537530
H	5.4694420	-1.5711040	-1.3680890
O	-1.8393750	1.0124110	-0.9217300
C	-0.5779720	0.6746760	-0.5047090
C	0.4603100	1.7216300	-0.7422840
H	1.4596980	1.2846700	-0.7094530

H	0.3204320	2.1737990	-1.7294510
C	0.4377150	2.8503880	0.2806550
F	0.6665470	2.4052950	1.5420760
F	1.4003550	3.7656060	0.0029450
F	-0.7429850	3.5155800	0.3079130
O	0.3362380	-1.8336950	1.8607850

$CF_3^{\bullet}$

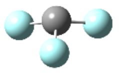

Gibbs free energy = -337.593646

**Symbol**	**X**	**Y**	**Z**
C	0.0000000	0.0000000	0.3320650
F	0.0000000	1.2666600	-0.0737920
F	-1.0969600	-0.6333300	-0.0737920
F	1.0969600	-0.6333300	-0.0737920

Intermediate I

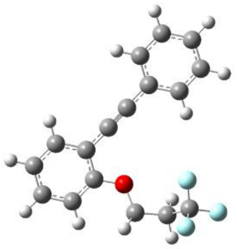

Gibbs free energy = -1029.546481

Symbol	X	Y	Z
C	3.4397220	-3.2924610	0.0374850
C	2.3302840	-4.1052840	0.2913080
C	1.0497920	-3.5547690	0.2684410
C	0.8506030	-2.1846320	0.0021220
C	1.9902070	-1.3812280	-0.2443980
C	3.2734380	-1.9313890	-0.2344290
H	4.4405950	-3.7147420	0.0399550
H	2.4609710	-5.1628610	0.5005660
H	0.1808670	-4.1769530	0.4605640
H	4.1339090	-1.3107150	-0.4591390

C	-0.4600440	-1.6280360	-0.0134650
C	-1.5911650	-1.1748600	-0.0213990
C	-2.9129980	-0.6360640	-0.0329820
C	-4.0302810	-1.4685870	0.1934560
C	-3.1224570	0.7394480	-0.2723050
C	-5.3193290	-0.9358100	0.1787640
H	-3.8769590	-2.5277610	0.3789090
C	-4.4153000	1.2625340	-0.2854610
H	-2.2672700	1.3860990	-0.4457480
C	-5.5173540	0.4291170	-0.0605990
H	-6.1711700	-1.5873350	0.3545420
H	-4.5630760	2.3230500	-0.4711990
H	-6.5230690	0.8403890	-0.0712950
O	1.7448720	-0.0558080	-0.5232280
C	2.7385880	0.8746400	-0.3895270
H	3.4847930	0.6971830	0.3791420
C	2.3770000	2.2463070	-0.8411840
H	3.2893730	2.8117220	-1.0598000
H	1.7757870	2.2084570	-1.7561670
C	1.5946010	3.0728930	0.1741880
F	2.2694220	3.2274610	1.3445020
F	1.3394980	4.3240270	-0.2960360
F	0.3937940	2.5228460	0.4888010

Intermediate IIa

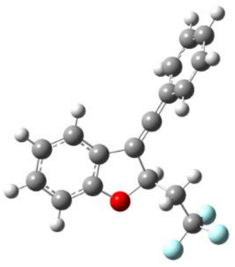

Gibbs free energy = -1029.578347

**Symbol**	**X**	**Y**	**Z**
C	2.4938200	3.9244670	-0.2257960
C	1.1225450	4.1716820	-0.3968720
C	0.1935740	3.1320660	-0.2738860
C	0.6576750	1.8491380	0.0231050
C	2.0291320	1.6238700	0.1883880

C	2.9711800	2.6406490	0.0704760
H	3.2027970	4.7421560	-0.3239820
H	0.7802830	5.1767790	-0.6262270
H	-0.8692290	3.3172120	-0.4055930
H	4.0303110	2.4421260	0.2031290
C	-0.0306680	0.5579150	0.2112980
C	-1.3062130	0.2711350	0.1415470
C	-2.6574590	0.0183340	0.0671420
C	-3.4971590	0.1230360	1.2277550
C	-3.2766330	-0.3619660	-1.1718060
C	-4.8574280	-0.1341300	1.1403990
H	-3.0489060	0.4081190	2.1749970
C	-4.6393080	-0.6141040	-1.2318710
H	-2.6593520	-0.4482510	-2.0612040
C	-5.4425260	-0.5038230	-0.0833300
H	-5.4751770	-0.0477120	2.0307220
H	-5.0878960	-0.8998970	-2.1799510
H	-6.5086720	-0.7034640	-0.1409760
O	2.3343880	0.3181170	0.4699470
C	1.1013460	-0.4495720	0.5290140
H	1.0071930	-0.8230830	1.5533720
C	1.1476190	-1.6099440	-0.4665450
H	0.1517900	-2.0620850	-0.5173940
H	1.4044070	-1.2440720	-1.4651570
C	2.1128870	-2.7200840	-0.1092130
F	1.8658860	-3.2439290	1.1226980
F	2.0175980	-3.7493410	-0.9948960
F	3.4124090	-2.3305950	-0.1100420

Intermediate IIb

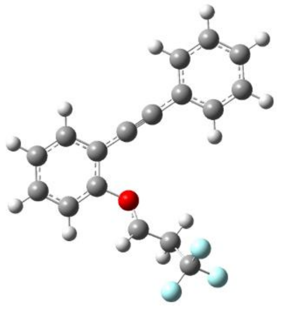

Gibbs free energy = -1029.365762

Symbol	X	Y	Z
C	-2.1236100	4.1153610	0.0228060
C	-0.8293970	4.5803340	0.2925070
C	0.2592650	3.7121880	0.2743090
C	0.0880110	2.3401950	-0.0101310
C	-1.2333760	1.9209950	-0.2552240
C	-2.3354880	2.7662970	-0.2595810
H	-2.9647990	4.8003400	0.0124990
H	-0.6683960	5.6323050	0.5063870
H	1.2597350	4.0776890	0.4793470
H	-3.3269780	2.4056650	-0.5135690
C	1.1809970	1.4368360	-0.0211190
C	2.1324640	0.6761810	-0.0184600
C	3.2483330	-0.2099690	-0.0143480
C	4.5517820	0.2880550	0.1997740
C	3.0622820	-1.5937830	-0.2218450
C	5.6403900	-0.5826600	0.2058970
H	4.6977630	1.3521510	0.3595930
C	4.1582870	-2.4551800	-0.2139640
H	2.0608560	-1.9797450	-0.3871920
C	5.4479490	-1.9539090	-0.0005020
H	6.6401930	-0.1912990	0.3719180
H	4.0066540	-3.5189660	-0.3742960
H	6.2993010	-2.6289110	0.0050900
O	-1.3676970	0.5402310	-0.5613190
C	-2.3754690	-0.1606520	-0.2717090
H	-3.1832680	0.2797090	0.3152250
C	-2.4310000	-1.5430400	-0.7902250
H	-3.0058550	-1.5036630	-1.7312490
H	-1.4308880	-1.9140370	-1.0291330
C	-3.1491470	-2.5084760	0.1484810
F	-4.4027600	-2.0875330	0.4340390
F	-3.2385090	-3.7273390	-0.4146720
F	-2.4916640	-2.6413200	1.3224040

Intermediate PQ•H

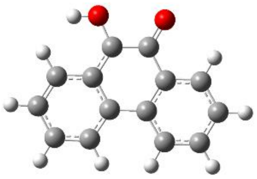

Gibbs free energy = -689.236424

Symbol	X	Y	Z
C	-3.5350290	-0.8773950	0.0000020
C	-2.8609950	0.3316860	0.0000020
C	-1.4416590	0.3853900	-0.0000030
C	-0.6947280	-0.8387210	-0.0000060
C	-1.4131980	-2.0480480	-0.0000030
C	-2.8046440	-2.0765700	0.0000000
C	-0.7397230	1.6323470	-0.0000030
C	0.7758860	-0.8032360	-0.0000030
C	1.4526410	0.4434690	-0.0000060
C	0.7086780	1.7169910	-0.0000170
C	2.8591030	0.4886930	0.0000000
H	3.3424220	1.4603090	-0.0000030
C	3.6084860	-0.6801280	0.0000050
C	2.9495380	-1.9193020	0.0000060
C	1.5600430	-1.9771400	0.0000020
H	-4.6207180	-0.8934650	0.0000060
H	-3.4565990	1.2409040	0.0000040
H	-0.8834880	-2.9933090	-0.0000030
H	-3.3224190	-3.0313440	0.0000020
H	4.6940030	-0.6358190	0.0000080
H	3.5230750	-2.8422140	0.0000110
H	1.0881130	-2.9528390	0.0000040
O	-1.3592170	2.8264090	0.0000070
O	1.2861030	2.8293560	0.0000050
H	-2.3258740	2.7334460	0.0000170

Intermediate PQH^{-}

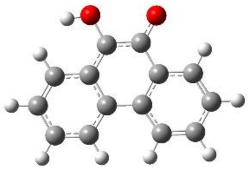

Gibbs free energy = -689.383174

Symbol	X	Y	Z
C	-3.5285790	-0.8583950	0.0000320
C	-2.8431310	0.3432890	0.0000280
C	-1.4141130	0.4003790	-0.0000210
C	-0.6963390	-0.8506140	-0.0000590
C	-1.4342440	-2.0556610	-0.0000360
C	-2.8223120	-2.0783300	0.0000010
C	-0.7008630	1.6329450	-0.0000460
C	0.7581390	-0.8179640	-0.0000440
C	1.4295000	0.4424420	-0.0000760
C	0.7033050	1.7239170	-0.0001870
C	2.8451340	0.4715300	-0.0000170
H	3.3262620	1.4448420	-0.0000420
C	3.5956040	-0.6921760	0.0000500
C	2.9386380	-1.9405760	0.0000620
C	1.5537840	-1.9941450	0.0000170
H	-4.6162160	-0.8560550	0.0000680
H	-3.4376520	1.2548260	0.0000580
H	-0.9062960	-3.0041610	-0.0000420
H	-3.3551730	-3.0254620	0.0000160
H	4.6823100	-0.6458790	0.0000870
H	3.5147350	-2.8629350	0.0001120
H	1.0775050	-2.9694650	0.0000390
O	-1.3740060	2.8557690	0.0000460
O	1.3411710	2.8440170	0.0001220
H	-2.3299320	2.7061610	0.0001370

Intermediate III

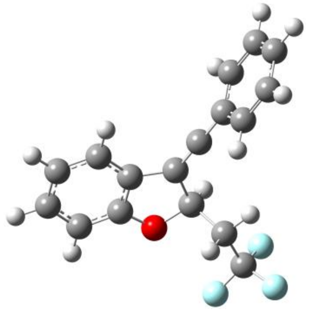

Gibbs free energy = -1029.394372

Symbol	X	Y	Z
C	2.5578570	3.8581350	-0.2117120
C	1.1973060	4.1437420	-0.4227990
C	0.2360990	3.1381160	-0.3158310
C	0.6679390	1.8476980	0.0061830
C	2.0282210	1.5776260	0.2109960
C	2.9977010	2.5702050	0.1089320
H	3.2893860	4.6563020	-0.2995620
H	0.8914180	5.1552330	-0.6710550
H	-0.8155730	3.3532210	-0.4779150
H	4.0467450	2.3461470	0.2712020
C	-0.0438280	0.5789580	0.1952750
C	-1.3155240	0.3270590	0.1236210
C	-2.6468950	0.0626000	0.0609030
C	-3.2485080	-0.3233250	-1.1886250
C	-3.4605270	0.1712910	1.2435920
C	-4.6023940	-0.5845000	-1.2392870
H	-2.6221480	-0.4009430	-2.0708150
C	-4.8118460	-0.0964070	1.1656230
H	-2.9929960	0.4628520	2.1779970
C	-5.3763890	-0.4712960	-0.0686120
H	-5.0717790	-0.8758370	-2.1727910
H	-5.4394080	-0.0194470	2.0469600
H	-6.4413640	-0.6793530	-0.1191970
O	2.2853420	0.2723650	0.5103800
C	1.0477850	-0.4703450	0.5308870
H	0.9018940	-0.8396360	1.5494110
C	1.0900470	-1.6205590	-0.4780540
H	0.0995170	-2.0819550	-0.5355910
H	1.3505940	-1.2476290	-1.4724740
C	2.0632930	-2.7266310	-0.1139900

F	1.8229970	-3.2316010	1.1250980
F	1.9562760	-3.7592710	-0.9888230
F	3.3583890	-2.3315800	-0.1309240

H_2O

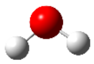

Gibbs free energy = -76.427803

Symbol	X	Y	Z
O	0.0000000	0.0000000	0.1185590
H	0.0000000	0.7684900	-0.4742380
H	0.0000000	-0.7684900	-0.4742380

Intermediate IV

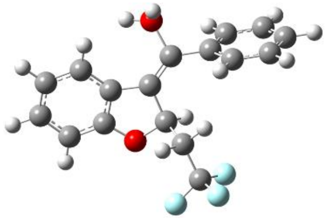

Gibbs free energy = -1105.807962

Symbol	X	Y	Z
C	-4.6923240	-1.1035830	0.1492470
C	-4.0603620	-2.1767170	-0.4995560
C	-2.6725270	-2.2008370	-0.6445360
C	-1.9159880	-1.1372200	-0.1261610
C	-2.5795170	-0.0630840	0.4922470
C	-3.9606490	-0.0231150	0.6498150
H	-5.7736310	-1.1033130	0.2517010
H	-4.6544930	-2.9902190	-0.9033920
H	-2.2237720	-3.0181280	-1.2019530
H	-4.4419670	0.8193070	1.1354320
C	-0.4858050	-0.8371870	-0.0471330
C	0.6021990	-1.6022990	-0.1868420
C	2.0406750	-1.3453710	-0.0370360
C	2.7723420	-0.7531260	-1.0816690
C	2.6979910	-1.7169320	1.1517900

C	4.1403130	-0.5159110	-0.9296570
H	2.2741230	-0.4926130	-2.0112380
C	4.0642740	-1.4791050	1.2955670
H	2.1345130	-2.1798220	1.9569600
C	4.7848020	-0.8762760	0.2572770
H	4.6999790	-0.0554450	-1.7385130
H	4.5660900	-1.7594490	2.2171250
H	5.8488670	-0.6905030	0.3738920
O	-1.7369220	0.9212530	0.9141350
C	-0.3998250	0.6159380	0.4407630
H	0.2722340	0.7070280	1.2964890
C	-0.0089910	1.6061280	-0.6660930
H	0.9626130	1.3190770	-1.0753450
H	-0.7405720	1.5843200	-1.4790810
C	0.1313960	3.0420090	-0.1968790
F	0.9769820	3.1564310	0.8621360
F	0.6399960	3.8179250	-1.1904490
F	-1.0404350	3.6091700	0.1766500
O	0.3695790	-3.0708800	-0.4257190
H	-0.5157470	-3.4102600	-0.1593650
H	0.5935900	-3.3845770	-1.3319260

Intermediate V

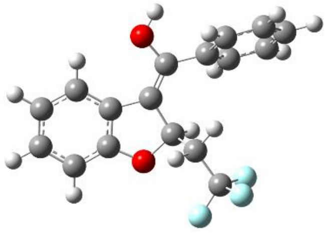

Gibbs free energy = -1105.449174

Symbol	X	Y	Z
C	-4.8105250	-0.8064100	0.1170360
C	-4.2196590	-2.0082520	-0.2956070
C	-2.8252940	-2.1401990	-0.3528900
C	-2.0237460	-1.0499360	0.0124310
C	-2.6454520	0.1431660	0.4190660
C	-4.0238700	0.2966710	0.4823490
H	-5.8933570	-0.7215280	0.1539850
H	-4.8483430	-2.8485980	-0.5771890

H	-2.3715980	-3.0701430	-0.6746990
H	-4.4685560	1.2337700	0.8035120
C	-0.5803690	-0.8351080	0.1031270
C	0.4150150	-1.7236630	-0.1221580
C	1.8686200	-1.4828210	0.0558160
C	2.7626710	-1.7814600	-0.9906520
C	2.3846840	-1.0063020	1.2737780
C	4.1357750	-1.5869880	-0.8271910
H	2.3789520	-2.1468180	-1.9399960
C	3.7584560	-0.8130140	1.4343700
H	1.7122360	-0.8098360	2.1038830
C	4.6372800	-1.0994940	0.3843560
H	4.8123150	-1.8114620	-1.6473730
H	4.1425690	-0.4497370	2.3837760
H	5.7059690	-0.9493250	0.5111950
O	-1.7488330	1.1221020	0.7591260
C	-0.4053200	0.6424310	0.4661730
H	0.1788290	0.8011130	1.3747680
C	0.1873960	1.4614410	-0.6930620
H	1.1378200	1.0115970	-0.9952130
H	-0.4866270	1.4355760	-1.5545320
C	0.4810350	2.9108860	-0.3693260
F	1.2982520	3.0446320	0.7118940
F	1.1135700	3.5219470	-1.4099390
F	-0.6265720	3.6492220	-0.1087420
O	0.0651060	-2.9683480	-0.6021010
H	0.8221730	-3.5725450	-0.5229210

Intermediate PQH_2

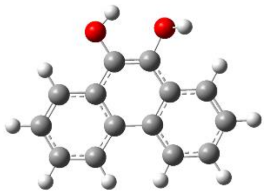

Gibbs free energy = -689.843186

Symbol	X	Y	Z
C	3.5560260	-0.8239010	-0.0285660
C	2.8479580	0.3659200	-0.0396500

C	1.4313270	0.3695610	-0.0089430
C	0.7206420	-0.8718500	0.0128260
C	1.4790880	-2.0666950	0.0266170
C	2.8653530	-2.0502140	0.0106280
C	0.6871850	1.5964650	0.0054700
C	-0.7388660	-0.8613940	0.0062160
C	-1.4348420	0.3850900	-0.0093780
C	-0.6803050	1.6073330	-0.0009530
C	-2.8502720	0.4098740	-0.0183700
H	-3.3582330	1.3684690	-0.0296390
C	-3.5764400	-0.7684560	-0.0123770
C	-2.9015500	-2.0051200	0.0022470
C	-1.5161170	-2.0455180	0.0115530
H	4.6422740	-0.8088020	-0.0546220
H	3.3887970	1.3064330	-0.0881050
H	0.9747290	-3.0265680	0.0488690
H	3.4164930	-2.9865390	0.0222600
H	-4.6627130	-0.7392610	-0.0196740
H	-3.4667070	-2.9333010	0.0059540
H	-1.0278970	-3.0137890	0.0213170
O	1.3065180	2.8370440	-0.0455910
H	2.0177390	2.8966580	0.6146090
O	-1.3740140	2.7856570	0.0072100
H	-0.7196340	3.5085300	0.0421460

H_2

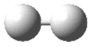

Gibbs free energy = -1.176923

**Symbol**	**X**	**Y**	**Z**

H	0.0000000	0.0000000	0.3715220
H	0.0000000	0.0000000	-0.3715220

Product 2a

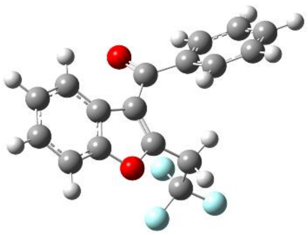

Gibbs free energy = -1104.283058

**Symbol**	**X**	**Y**	**Z**
C	-4.6906640	-1.2372410	-0.3620370
C	-3.9898880	-2.2559760	0.3166900
C	-2.6199220	-2.1605330	0.5588770
C	-1.9470090	-1.0127620	0.1088850
C	-2.6748890	-0.0189510	-0.5619350
C	-4.0390970	-0.0900560	-0.8196930
H	-5.7579460	-1.3456310	-0.5326890
H	-4.5319790	-3.1329270	0.6594620
H	-2.0891300	-2.9460600	1.0865590
H	-4.5626620	0.7016020	-1.3459880
C	-0.5691300	-0.5482190	0.1209680
C	0.5513920	-1.2511430	0.7943040
C	1.9078000	-1.2888560	0.1716240
C	3.0329480	-1.4222720	1.0049430
C	2.0806080	-1.2687710	-1.2232290
C	4.3095500	-1.5109940	0.4527800
H	2.8942130	-1.4438890	2.0815570
C	3.3591740	-1.3805740	-1.7745000
H	1.2169240	-1.1916510	-1.8772010
C	4.4743180	-1.4939110	-0.9384180
H	5.1761060	-1.5956590	1.1026780
H	3.4832810	-1.3798010	-2.8537530
H	5.4694420	-1.5711040	-1.3680890
O	-1.8393750	1.0124110	-0.9217300
C	-0.5779720	0.6746760	-0.5047090
C	0.4603100	1.7216300	-0.7422840
H	1.4596980	1.2846700	-0.7094530

H	0.3204320	2.1737990	-1.7294510
C	0.4377150	2.8503880	0.2806550
F	0.6665470	2.4052950	1.5420760
F	1.4003550	3.7656060	0.0029450
F	-0.7429850	3.5155800	0.3079130
O	0.3362380	-1.8336950	1.8607850

Transition state TS

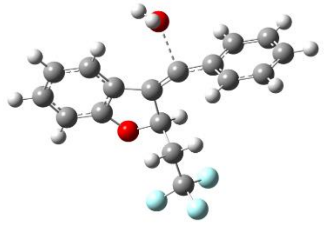

Gibbs free energy = -1105.795932

**Symbol**	**X**	**Y**	**Z**
C	-0.1776050	0.1433590	0.2325260
C	0.0076230	0.1012960	1.6242540
C	1.2869490	-0.0150070	2.1692410
C	2.3836780	-0.0819390	1.3007970
C	2.1691340	-0.0738040	-0.0873430
C	0.9030240	0.0485870	-0.6486780
H	-1.1816880	0.2368190	-0.1710420
H	-0.8520680	0.1530980	2.2849010
H	1.4123330	-0.0779040	3.2428230
H	0.7691420	0.0584050	-1.7252120
C	3.8294170	-0.1964180	1.4990030
C	4.6801320	-0.0622220	2.4828880
C	5.9223240	-0.1422120	3.1044970
C	6.3967140	-1.3965910	3.5858050
C	6.7301730	1.0231760	3.2430640
C	7.6564370	-1.4787910	4.1604020
H	5.7659790	-2.2752060	3.4964650
C	7.9873530	0.9211800	3.8159900
H	6.3508000	1.9756350	2.8881750
C	8.4471220	-0.3251370	4.2729150
H	8.0286680	-2.4315030	4.5229880
H	8.6149430	1.8010070	3.9142830
H	9.4328700	-0.3964420	4.7237000

One

O	3.3167030	-0.1950830	-0.8159310
C	4.4090230	-0.4885090	0.0780010
H	5.2212130	0.1987070	-0.1621340
C	4.8509650	-1.9488430	-0.0737570
H	5.5952850	-2.1792050	0.6936920
H	4.0021570	-2.6251040	0.0618960
C	5.4981510	-2.2624230	-1.4103450
F	6.5310530	-1.4233910	-1.6885750
F	6.0039820	-3.5229240	-1.4091160
F	4.6407580	-2.1867280	-2.4559410
O	3.6384400	0.8153820	4.1969680
H	2.9180910	1.4505350	4.0346390
H	3.2873030	0.1548700	4.8213020

imaginary frequency (negative Signs).

Crystal Data

ORTEP view of 2c with 50% ellipsoidal probability

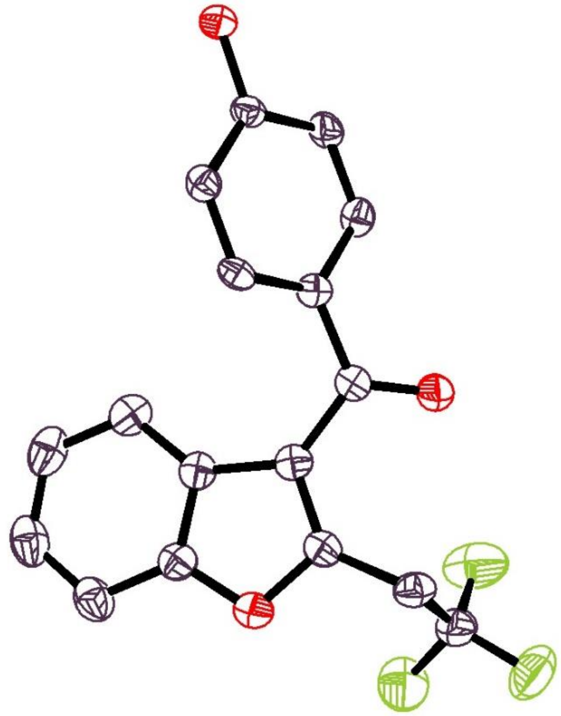

Packing diagram of 2c

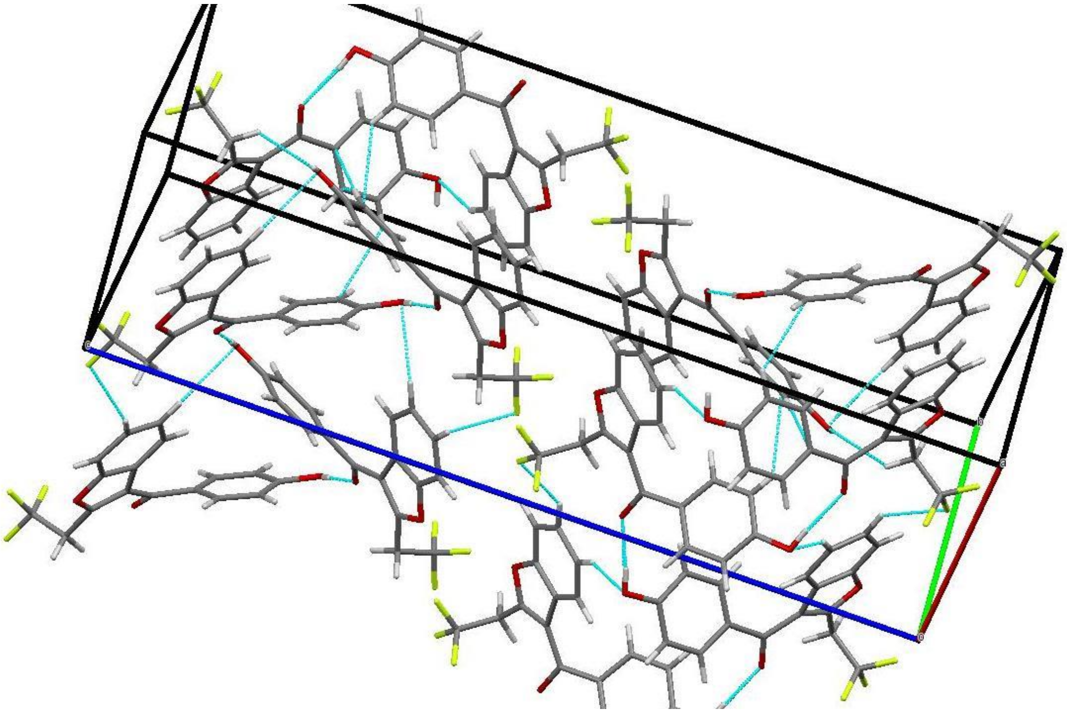

Table 1. Crystal data and structure refinement for 2c

Identification code	SJ-639	
Empirical formula	$C_{17} H_{11} F_3 O_3$	
Formula weight	320.26	
Temperature	140(2) K	
Wavelength	0.71073 Å	
Crystal system	Orthorhombic	
Space group	P b c a	
Unit cell dimensions	a = 10.159(4) Å	α= 90°.
	b = 9.480(4) Å	β= 90°.
	c = 29.456(10) Å	γ = 90°.
Volume	2836.9(18) $Å^3$	
Z	8	
Density (calculated)	1.500 g/cm^3	
Absorption coefficient	0.128 mm^{-1}	
F(000)	1312	
Crystal size	0.03 x 0.02 x 0.01 mm^3	
Theta range for data collection	2.435 to 25.856°.	
Index ranges	-12<=h<=12, -11<=k<=11, -35<=l<=32	
Reflections collected	18646	
Independent reflections	2709 [R(int) = 0.0992]	
Completeness to theta = 25.242°	100.0 %	
Absorption correction	None	
Refinement method	Full-matrix least-squares on F^2	
Data / restraints / parameters	2709 / 0 / 209	
Goodness-of-fit on F^2	1.081	
Final R indices [I>2sigma(I)]	R1 = 0.0635, wR2 = 0.1258	
R indices (all data)	R1 = 0.1075, wR2 = 0.1409	
Extinction coefficient	n/a	
Largest diff. peak and hole	0.229 and -0.250 e.Å^{-3}	

Table 2. Atomic coordinates (x 10^4) and equivalent isotropic displacement parameters (Å^{2}x 10^3) for 2c. U(eq) is defined as one third of the trace of the orthogonalized U^{ij} tensor

	x	y	z	U(eq)
O(3)	5869(2)	6605(2)	3582(1)	33(1)
O(2)	2934(2)	5137(2)	1757(1)	33(1)
O(1)	3445(2)	9351(2)	4325(1)	34(1)
F(3)	5318(2)	8350(2)	5003(1)	61(1)
F(2)	7328(2)	8645(3)	4821(1)	67(1)
F(1)	6287(3)	6828(2)	4592(1)	72(1)
C(11)	4729(3)	6908(3)	3474(1)	26(1)
C(13)	3294(3)	7259(3)	2785(1)	26(1)
C(15)	3336(3)	5612(3)	2168(1)	25(1)
C(12)	4201(3)	6453(3)	3026(1)	24(1)
C(6)	2476(3)	7873(3)	3817(1)	28(1)
C(7)	3909(3)	7757(3)	3778(1)	27(1)
C(1)	2265(3)	8864(3)	4155(1)	31(1)
C(8)	4411(3)	8651(3)	4097(1)	29(1)
C(17)	4686(3)	5229(3)	2824(1)	28(1)
C(14)	2876(3)	6858(3)	2356(1)	26(1)
C(16)	4259(3)	4813(3)	2403(1)	29(1)
C(9)	5775(3)	9004(3)	4248(1)	32(1)
C(5)	1372(3)	7219(3)	3626(1)	36(1)
C(4)	138(3)	7640(4)	3769(1)	43(1)
C(2)	1043(3)	9320(3)	4299(1)	40(1)
C(10)	6175(3)	8200(4)	4663(1)	41(1)
C(3)	-17(3)	8685(4)	4097(1)	44(1)

Table 3. Selected bond lengths [Å] for 2c

O3—C11	1.235 (3)	C6—C7	1.465 (4)
O2—C15	1.355 (3)	C7—C8	1.365 (4)
O2—H02	0.84	C1—C2	1.381 (4)

O1—C8	1.362 (3)	C8—C9	1.493 (4)
O1—C1	1.379 (4)	C17—C16	1.372 (4)
F3—C10	1.335 (4)	C17—H17	0.95
F2—C10	1.329 (4)	C14—H14	0.95
F1—C10	1.323 (4)	C16—H16	0.95
C11—C7	1.464 (4)	C9—C10	1.497 (4)
C11—C12	1.489 (4)	C9—H09B	0.99
C13—C14	1.386 (4)	C9—H09A	0.99
C13—C12	1.391 (4)	C5—C4	1.382 (4)
C13—H13	0.95	C5—H5	0.95
C15—C14	1.386 (4)	C4—C3	1.392 (5)
C15—C16	1.389 (4)	C4—H4	0.95
C12—C17	1.394 (4)	C2—C3	1.370 (5)
C6—C1	1.384 (4)	C2—H2	0.95
C6—C5	1.399 (4)	C3—H3	0.95

Table 3. Selected bond angles [°] for 2c

C15—O2—H02	109.5	C15—C14—H14	120.2
C8—O1—C1	106.5 (2)	C13—C14—H14	120.2
O3—C11—C7	120.2 (2)	C17—C16—C15	120.4 (3)
O3—C11—C12	120.0 (2)	C17—C16—H16	119.8
C7—C11—C12	119.7 (3)	C15—C16—H16	119.8
C14—C13—C12	121.2 (3)	C8—C9—C10	112.4 (3)

C14—C13—H13	119.4	C8—C9—H09B	109.1
C12—C13—H13	119.4	C10—C9—H09B	109.1
O2—C15—C14	122.6 (3)	C8—C9—H09A	109.1
O2—C15—C16	117.8 (2)	C10—C9—H09A	109.1
C14—C15—C16	119.6 (3)	H09B—C9—H09A	107.9
C13—C12—C17	118.3 (3)	C4—C5—C6	118.4 (3)
C13—C12—C11	122.1 (2)	C4—C5—H5	120.8
C17—C12—C11	119.4 (3)	C6—C5—H5	120.8
C1—C6—C5	117.8 (3)	C5—C4—C3	121.3 (3)
C1—C6—C7	105.2 (3)	C5—C4—H4	119.3
C5—C6—C7	137.0 (3)	C3—C4—H4	119.3
C8—C7—C11	123.4 (3)	C3—C2—C1	115.9 (3)
C8—C7—C6	105.6 (2)	C3—C2—H2	122.1
C11—C7—C6	131.0 (3)	C1—C2—H2	122.1
O1—C1—C2	124.4 (3)	F1—C10—F2	107.0 (3)
O1—C1—C6	110.7 (3)	F1—C10—F3	106.2 (3)
C2—C1—C6	124.9 (3)	F2—C10—F3	106.1 (3)
O1—C8—C7	111.9 (2)	F1—C10—C9	113.3 (3)
O1—C8—C9	114.4 (2)	F2—C10—C9	111.3 (3)
C7—C8—C9	133.7 (3)	F3—C10—C9	112.5 (3)
C16—C17—C12	120.9 (3)	C2—C3—C4	121.6 (3)
C16—C17—H17	119.6	C2—C3—H3	119.2

C12—C17—H17	119.6	C4—C3—H3	119.2
C15—C14—C13	119.6 (3)		

Table 5. Anisotropic displacement parameters ($Å^2 x 10^3$) for 2c The anisotropic displacement factor exponent takes the form: $-2\pi^2[h^2 a^{*2} U^{11} + ... + 2 h k a^* b^* U^{12}]$

	U^{11}	U^{22}	U^{33}	U^{23}	U^{13}	U^{12}
O(3)	32(1)	39(1)	29(1)	-2(1)	0(1)	3(1)
O(2)	32(1)	34(1)	32(1)	-12(1)	-3(1)	5(1)
O(1)	36(1)	35(1)	30(1)	-4(1)	2(1)	1(1)
F(3)	60(1)	87(2)	37(1)	13(1)	7(1)	2(1)
F(2)	46(1)	105(2)	49(1)	-2(1)	-14(1)	-12(1)
F(1)	113(2)	50(1)	54(1)	-2(1)	-26(1)	18(1)
C(11)	30(2)	22(2)	26(2)	4(1)	2(1)	-1(1)
C(13)	31(2)	19(1)	28(2)	-3(1)	5(1)	2(1)
C(15)	25(2)	24(2)	26(2)	-5(1)	4(1)	-3(1)
C(12)	27(2)	22(1)	24(2)	1(1)	2(1)	-4(1)
C(6)	31(2)	30(2)	25(2)	7(1)	2(1)	-1(1)
C(7)	33(2)	25(2)	23(2)	5(1)	0(1)	-1(1)
C(1)	37(2)	33(2)	23(2)	6(1)	-1(1)	2(2)
C(8)	33(2)	28(2)	26(2)	3(1)	4(1)	1(1)
C(17)	28(2)	22(2)	35(2)	4(1)	-3(1)	2(1)
C(14)	28(2)	24(2)	28(2)	3(1)	0(1)	3(1)
C(16)	27(2)	18(1)	41(2)	-9(1)	1(1)	2(1)
C(9)	38(2)	33(2)	26(2)	-2(1)	2(1)	-6(2)
C(5)	39(2)	38(2)	32(2)	6(1)	1(1)	-8(2)
C(4)	33(2)	56(2)	40(2)	16(2)	-3(2)	-9(2)
C(2)	39(2)	46(2)	34(2)	6(2)	7(2)	8(2)
C(10)	38(2)	51(2)	34(2)	-7(2)	2(2)	-3(2)
C(3)	35(2)	57(2)	40(2)	16(2)	10(2)	8(2)

Table 6. Hydrogen coordinates (x 10^4) and isotropic displacement parameters (Å^{2}x 10^3) for 2c

	x	y	z	U(eq)
H(02)	2283	5611	1669	49
H(13)	2956	8098	2917	31
H(17)	5321	4676	2980	34
H(14)	2278	7435	2192	32
H(16)	4596	3973	2271	34
H(09B)	5829	10028	4312	39
H(09A)	6399	8791	3999	39
H(5)	1469	6502	3403	44
H(4)	-620	7207	3641	51
H(2)	944	10031	4523	48
H(3)	-880	8964	4183	53

***ORTEP* View of 4k with 50% ellipsoidal probability**

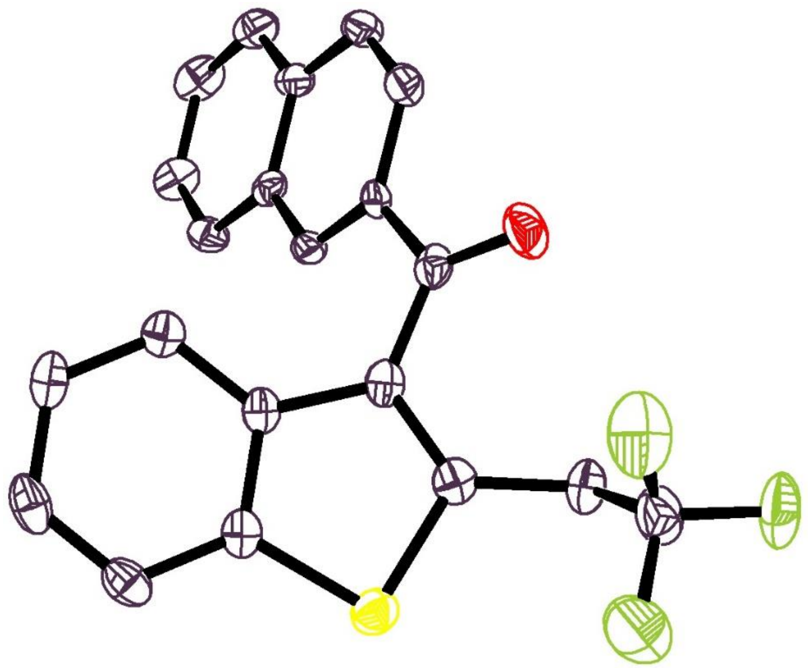

Packing diagram of **4k**

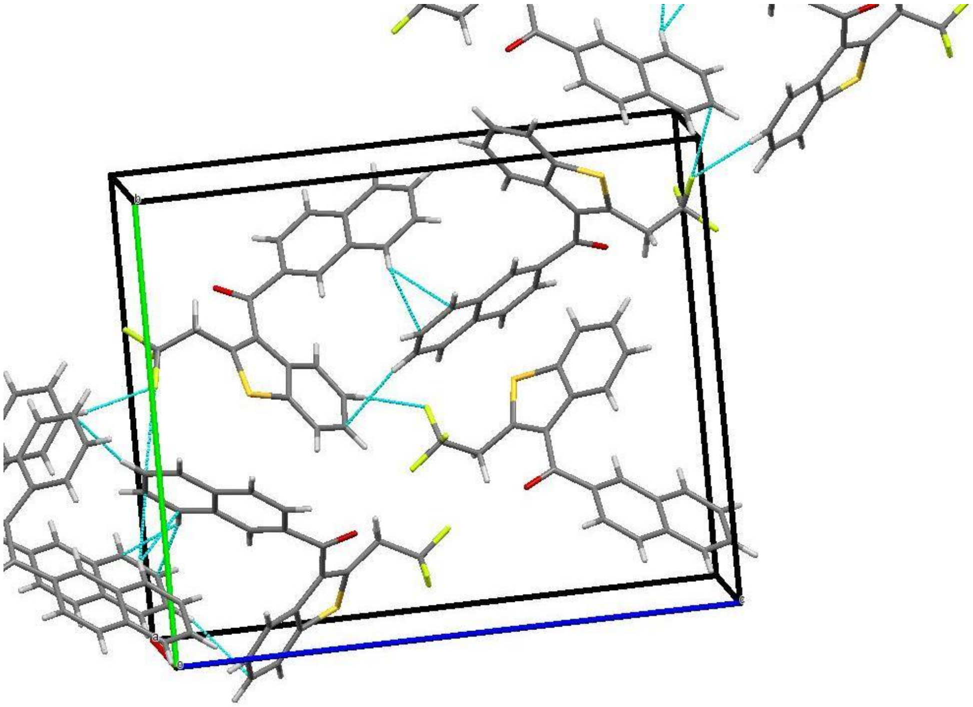

Table 1. Crystal data and structure refinement for 4k

Identification code	SJ_694	
Empirical formula	$C_{21} H_{13} F_3 O S$	
Formula weight	370.37	
Temperature	139(2) K	
Wavelength	0.71073 Å	
Crystal system	Orthorhombic	
Space group	$P2_12_12_1$	
Unit cell dimensions	a = 5.4622(3) Å	α= 90°.
	b = 15.8611(10) Å	β= 90°.
	c = 19.1121(10) Å	γ = 90°.
Volume	1655.81(16) $Å^3$	
Z	4	
Density (calculated)	1.486 g/cm^3	
Absorption coefficient	0.234 mm^{-1}	
F(000)	760	
Crystal size	0.05 x 0.04 x 0.02 mm^3	
Theta range for data collection	2.488 to 25.026°.	
Index ranges	-5<=h<=6, -18<=k<=18, -22<=l<=22	
Reflections collected	11543	
Independent reflections	2917 [R(int) = 0.0690]	
Completeness to theta = 25.026°	99.8 %	
Absorption correction	None	
Refinement method	Full-matrix least-squares on F^2	
Data / restraints / parameters	2917 / 0 / 235	
Goodness-of-fit on F^2	1.051	
Final R indices [I>2sigma(I)]	R1 = 0.0418, wR2 = 0.0755	
R indices (all data)	R1 = 0.0640, wR2 = 0.0826	
Absolute structure parameter	0.00(6)	
Extinction coefficient	n/a	
Largest diff. peak and hole	0.184 and -0.198 e.$Å^{-3}$	

Table 2. Atomic coordinates (x 10^4) and equivalent isotropic displacement parameters ($Å^2$x 10^3) for 4k U(eq) is defined as one third of the trace of the orthogonalized U^{ij} tensor

	x	y	z	U(eq)
S(1)	3058(2)	5450(1)	1825(1)	29(1)
F(1)	5056(5)	6984(2)	-168(1)	52(1)
F(2)	3981(6)	5773(2)	215(1)	72(1)
F(3)	7640(5)	6203(2)	369(1)	53(1)
O(1)	9382(5)	7376(2)	1642(1)	36(1)
C(17)	8305(8)	9089(3)	5174(2)	37(1)
C(18)	9947(8)	9120(2)	4640(2)	33(1)
C(19)	9628(7)	8618(2)	4031(2)	24(1)
C(20)	11273(8)	8641(3)	3458(2)	31(1)
C(21)	10888(7)	8150(3)	2881(2)	29(1)
C(12)	8853(7)	7601(2)	2846(2)	21(1)
C(11)	8439(7)	7125(2)	2179(2)	23(1)
C(2)	6733(7)	6392(2)	2174(2)	22(1)
C(3)	6645(7)	5724(2)	2690(2)	20(1)
C(8)	4689(7)	5170(2)	2570(2)	23(1)
C(7)	4248(7)	4478(2)	3002(2)	28(1)
C(6)	5830(8)	4336(2)	3552(2)	31(1)
C(16)	6261(8)	8563(2)	5131(2)	31(1)
C(15)	5883(7)	8066(2)	4558(2)	25(1)
C(14)	7563(7)	8082(2)	3995(2)	22(1)
C(13)	7229(7)	7570(2)	3390(2)	21(1)
C(5)	7849(8)	4853(2)	3661(2)	29(1)
C(4)	8269(7)	5543(2)	3241(2)	24(1)
C(1)	4979(7)	6299(2)	1671(2)	23(1)
C(9)	4541(8)	6853(2)	1044(2)	29(1)
C(10)	5302(9)	6453(3)	371(2)	38(1)

Table 3. Selected bond lengths [Å] for 4k

S1—C1	1.733 (4)	C2—C3	1.449 (5)
S1—C8	1.737 (4)	C3—C8	1.402 (5)
F1—C10	1.338 (5)	C3—C4	1.406 (5)
F2—C10	1.331 (5)	C8—C7	1.394 (5)
F3—C10	1.338 (5)	C7—C6	1.379 (5)
O1—C11	1.216 (4)	C7—H7	0.95
C17—C18	1.358 (6)	C6—C5	1.390 (5)
C17—C16	1.397 (6)	C6—H6	0.95
C17—H17	0.95	C16—C15	1.366 (5)
C18—C19	1.422 (5)	C16—H16	0.95
C18—H18	0.95	C15—C14	1.414 (5)
C19—C14	1.413 (5)	C15—H15	0.95
C19—C20	1.416 (5)	C14—C13	1.425 (4)
C20—C21	1.366 (5)	C13—H13	0.95
C20—H20	0.95	C5—C4	1.378 (5)
C21—C12	1.414 (5)	C5—H5	0.95
C21—H21	0.95	C4—H4	0.95
C12—C13	1.368 (5)	C1—C9	1.504 (5)
C12—C11	1.498 (5)	C9—C10	1.492 (5)
C11—C2	1.491 (5)	C9—H9B	0.99
C2—C1	1.365 (5)	C9—H9A	0.99

Table 4. Selected bond angles [°] for 4k

C1—S1—C8	91.62 (18)	C7—C6—H6	119.5
C18—C17—C16	120.3 (4)	C5—C6—H6	119.5
C18—C17—H17	119.8	C15—C16—C17	120.9 (4)
C16—C17—H17	119.8	C15—C16—H16	119.6
C17—C18—C19	120.9 (4)	C17—C16—H16	119.6
C17—C18—H18	119.5	C16—C15—C14	120.1 (4)
C19—C18—H18	119.5	C16—C15—H15	120
C14—C19—C20	119.0 (3)	C14—C15—H15	120
C14—C19—C18	118.3 (4)	C19—C14—C15	119.5 (3)
C20—C19—C18	122.7 (4)	C19—C14—C13	119.0 (3)
C21—C20—C19	120.7 (4)	C15—C14—C13	121.5 (3)
C21—C20—H20	119.6	C12—C13—C14	120.9 (3)
C19—C20—H20	119.6	C12—C13—H13	119.6
C20—C21—C12	120.7 (4)	C14—C13—H13	119.6
C20—C21—H21	119.6	C4—C5—C6	120.9 (4)
C12—C21—H21	119.6	C4—C5—H5	119.6
C13—C12—C21	119.7 (3)	C6—C5—H5	119.6
C13—C12—C11	122.0 (3)	C5—C4—C3	119.5 (4)
C21—C12—C11	118.0 (3)	C5—C4—H4	120.2
O1—C11—C2	120.9 (3)	C3—C4—H4	120.2
O1—C11—C12	119.3 (3)	C2—C1—C9	127.6 (4)
C2—C11—C12	119.6 (3)	C2—C1—S1	112.8 (3)

C1—C2—C3	112.2 (3)	C9—C1—S1	119.6 (3)
C1—C2—C11	121.8 (3)	C10—C9—C1	113.2 (3)
C3—C2—C11	125.9 (3)	C10—C9—H9B	108.9
C8—C3—C4	118.4 (3)	C1—C9—H9B	108.9
C8—C3—C2	111.9 (3)	C10—C9—H9A	108.9
C4—C3—C2	129.6 (4)	C1—C9—H9A	108.9
C7—C8—C3	121.9 (3)	H9B—C9—H9A	107.8
C7—C8—S1	126.7 (3)	F2—C10—F1	106.4 (3)
C3—C8—S1	111.4 (3)	F2—C10—F3	106.1 (4)
C6—C7—C8	118.1 (4)	F1—C10—F3	106.2 (3)
C6—C7—H7	121	F2—C10—C9	112.7 (3)
C8—C7—H7	121	F1—C10—C9	111.6 (4)
C7—C6—C5	121.1 (4)	F3—C10—C9	113.3 (4)

Table 5. Anisotropic displacement parameters ($Å^2$x 10^3) for 4k The anisotropic displacement factor exponent takes the form: $-2\pi^2[h^2a^{*2}U^{11} + ... + 2 h k a^* b^* U^{12}]$

	U^{11}	U^{22}	U^{33}	U^{23}	U^{13}	U^{12}
S(1)	31(1)	29(1)	26(1)	-2(1)	-4(1)	-1(1)
F(1)	74(2)	61(2)	20(1)	11(1)	3(1)	2(2)
F(2)	119(3)	67(2)	32(1)	-15(1)	8(2)	-49(2)
F(3)	64(2)	55(2)	40(1)	-3(1)	19(1)	23(2)
O(1)	42(2)	41(2)	25(1)	5(1)	10(1)	-6(2)
C(17)	42(3)	29(2)	40(2)	-15(2)	-10(2)	6(2)
C(18)	32(2)	21(2)	45(2)	-1(2)	-11(2)	0(2)
C(19)	20(2)	17(2)	35(2)	3(2)	-6(2)	0(2)
C(20)	24(2)	26(2)	41(2)	10(2)	-5(2)	-6(2)

C(21)	24(2)	33(3)	30(2)	10(2)	2(2)	-1(2)
C(12)	22(2)	18(2)	21(2)	7(2)	-1(2)	0(2)
C(11)	19(2)	28(2)	23(2)	5(2)	1(2)	8(2)
C(2)	26(2)	24(2)	15(2)	-3(2)	5(2)	5(2)
C(3)	25(2)	20(2)	15(2)	-2(2)	4(2)	4(2)
C(8)	29(2)	19(2)	19(2)	-2(2)	3(2)	6(2)
C(7)	34(2)	20(2)	31(2)	-4(2)	7(2)	-3(2)
C(6)	48(3)	17(2)	27(2)	2(2)	10(2)	4(2)
C(16)	32(3)	31(3)	29(2)	-10(2)	1(2)	5(2)
C(15)	20(2)	25(2)	31(2)	-1(2)	3(2)	-1(2)
C(14)	21(2)	17(2)	26(2)	2(2)	-6(2)	5(2)
C(13)	20(2)	16(2)	27(2)	3(2)	-4(2)	-2(2)
C(5)	40(3)	27(2)	20(2)	4(2)	-1(2)	9(2)
C(4)	28(2)	22(2)	21(2)	-4(2)	0(2)	2(2)
C(1)	28(2)	22(2)	20(2)	-6(2)	4(2)	3(2)
C(9)	36(3)	31(2)	20(2)	2(2)	1(2)	6(2)
C(10)	54(3)	37(3)	23(2)	-1(2)	5(2)	-8(3)

Table 6. Hydrogen coordinates (x 10^4) and isotropic displacement parameters (Å^{2}x 10^3) for 4k

	x	y	z	U(eq)
H(17)	8549	9428	5578	44
H(18)	11328	9481	4675	39
H(20)	12659	9002	3475	37
H(21)	12000	8178	2500	35
H(7)	2897	4114	2919	34
H(6)	5534	3879	3861	37
H(16)	5119	8550	5506	37
H(15)	4489	7708	4537	30
H(13)	5860	7202	3364	25
H(5)	8955	4728	4030	35
H(4)	9647	5896	3322	28

H(9B)	2777	6993	1019	35
H(9A)	5456	7386	1105	35

***ORTEP* View of 6c with 70% ellipsoidal probability**

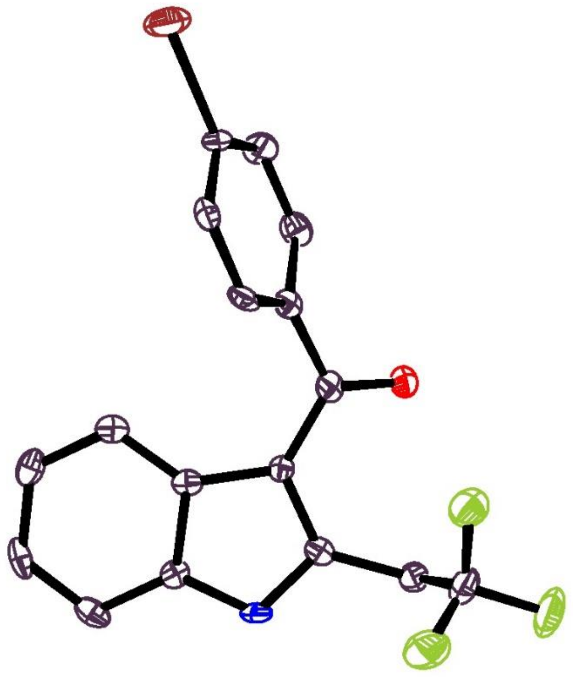

Packing diagram of **6c**

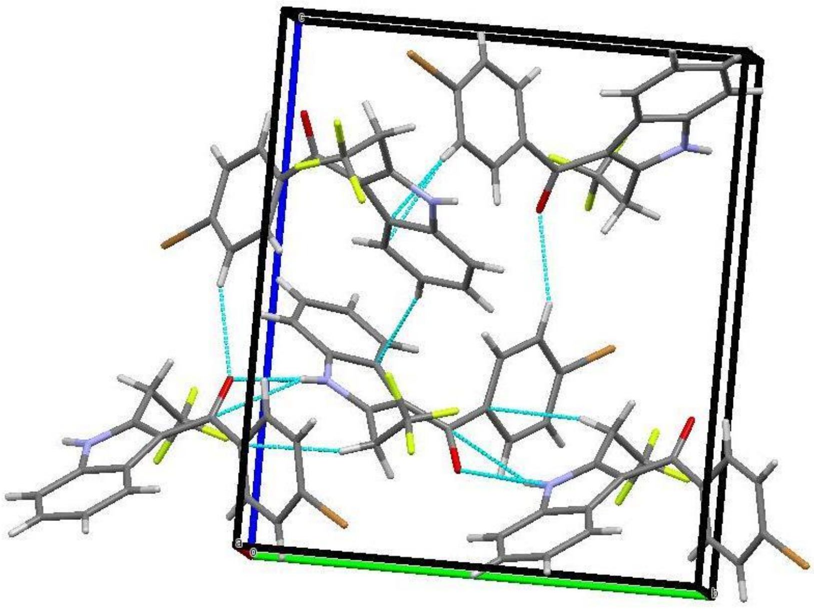

Table 1. Crystal data and structure refinement for 6c

Identification code	SJ_741	
Empirical formula	$C_{17} H_{11} Br F_3 N O$	
Formula weight	382.18	
Temperature	100(2) K	
Wavelength	0.71073 Å	
Crystal system	Orthorhombic	
Space group	$P2_12_12_1$	
Unit cell dimensions	a = 8.8354(4) Å	α= 90°.
	b = 12.2152(5) Å	β= 90°.
	c = 14.1439(7) Å	γ = 90°.
Volume	1526.50(12) $Å^3$	
Z	4	
Density (calculated)	1.663 g/cm^3	
Absorption coefficient	2.728 mm^{-1}	
F(000)	760	
Crystal size	0.4 x 0.3 x 0.1 mm^3	
Theta range for data collection	2.718 to 25.717°.	
Index ranges	-10<=h<=10, -14<=k<=13, -17<=l<=16	
Reflections collected	12899	
Independent reflections	2889 [R(int) = 0.0601]	
Completeness to theta = 25.242°	99.9 %	
Absorption correction	Multi scan	
Max. and min. transmission	0.745 and 0.571	
Refinement method	Full-matrix least-squares on F^2	
Data / restraints / parameters	2889 / 0 / 208	
Goodness-of-fit on F^2	1.044	
Final R indices [I>2sigma(I)]	R1 = 0.0317, wR2 = 0.0566	
R indices (all data)	R1 = 0.0409, wR2 = 0.0590	
Absolute structure parameter	0.020(7)	
Extinction coefficient	n/a	
Largest diff. peak and hole	0.482 and -0.413 e.$Å^{-3}$	

Table 2. Atomic coordinates (x 10^4) and equivalent isotropic displacement parameters ($Å^2$x 10^3) for 6c U(eq) is defined as one third of the trace of the orthogonalized U^{ij} tensor

	x	y	z	U(eq)
C(1)	4787(4)	2419(3)	2811(3)	11(1)
C(2)	3522(4)	3052(3)	3041(3)	9(1)
C(3)	2628(4)	2415(3)	3698(2)	10(1)
C(4)	1219(4)	2524(3)	4142(2)	12(1)
C(5)	668(5)	1669(3)	4675(3)	15(1)
C(6)	1468(5)	688(3)	4778(3)	16(1)
C(7)	2851(4)	550(3)	4354(3)	14(1)
C(8)	3412(4)	1414(3)	3816(3)	10(1)
C(9)	6105(4)	2665(3)	2180(3)	14(1)
C(10)	7291(5)	3359(3)	2651(3)	20(1)
C(11)	3184(4)	4110(3)	2623(3)	12(1)
C(12)	2076(4)	4847(3)	3091(3)	11(1)
C(13)	2152(5)	5066(3)	4059(3)	13(1)
C(14)	1168(4)	5811(3)	4480(3)	12(1)
C(15)	91(5)	6306(3)	3905(3)	15(1)
C(16)	-27(5)	6100(3)	2952(3)	18(1)
C(17)	995(4)	5380(3)	2542(3)	15(1)
N(1)	4721(4)	1462(3)	3288(2)	13(1)
F(1)	8465(3)	3529(2)	2065(2)	29(1)
F(2)	7846(3)	2878(2)	3428(2)	27(1)
F(3)	6778(2)	4346(2)	2909(2)	22(1)
BR1	-1287(1)	7324(1)	4457(1)	31(1)
O(1)	3766(3)	4421(2)	1869(2)	13(1)

Table 3. Selected bond lengths [Å] for 6c

C1—N1	1.350 (5)	C9—H9B	0.97
C1—C2	1.398 (5)	C10—F3	1.339 (5)
C1—C9	1.497 (5)	C10—F2	1.339 (5)
C2—C3	1.447 (5)	C10—F1	1.344 (5)

C2—C11	1.452 (5)	C11—O1	1.243 (5)
C3—C4	1.401 (5)	C11—C12	1.486 (5)
C3—C8	1.414 (5)	C12—C17	1.393 (5)
C4—C5	1.377 (5)	C12—C13	1.396 (5)
C4—H4	0.93	C13—C14	1.393 (5)
C5—C6	1.399 (6)	C13—H13	0.93
C5—H5	0.93	C14—C15	1.389 (5)
C6—C7	1.372 (6)	C14—H14	0.93
C6—H6	0.93	C15—C16	1.376 (6)
C7—C8	1.392 (5)	C15—BR1	1.908 (4)
C7—H7	0.93	C16—C17	1.388 (6)
C8—N1	1.378 (5)	C16—H16	0.93
C9—C10	1.503 (6)	C17—H17	0.93
C9—H9A	0.97	N1—H1	0.86

Table 4. Selected bond angles [°] for 6c

N1—C1—C2	109.1 (3)	F3—C10—F2	107.2 (4)
N1—C1—C9	120.4 (3)	F3—C10—F1	106.9 (3)
C2—C1—C9	130.5 (3)	F2—C10—F1	106.9 (3)
C1—C2—C3	106.8 (3)	F3—C10—C9	113.1 (3)
C1—C2—C11	124.2 (4)	F2—C10—C9	111.8 (3)
C3—C2—C11	128.9 (3)	F1—C10—C9	110.6 (4)
C4—C3—C8	117.7 (3)	O1—C11—C2	122.4 (4)
C4—C3—C2	136.2 (4)	O1—C11—C12	118.0 (3)
C8—C3—C2	105.9 (3)	C2—C11—C12	119.6 (4)
C5—C4—C3	119.2 (4)	C17—C12—C13	119.4 (4)
C5—C4—H4	120.4	C17—C12—C11	119.1 (4)

C3—C4—H4	120.4	C13—C12—C11	121.4 (4)
C4—C5—C6	121.8 (4)	C14—C13—C12	120.9 (4)
C4—C5—H5	119.1	C14—C13—H13	119.5
C6—C5—H5	119.1	C12—C13—H13	119.5
C7—C6—C5	120.7 (4)	C15—C14—C13	117.5 (4)
C7—C6—H6	119.6	C15—C14—H14	121.2
C5—C6—H6	119.6	C13—C14—H14	121.2
C6—C7—C8	117.5 (4)	C16—C15—C14	123.1 (4)
C6—C7—H7	121.2	C16—C15—BR1	118.2 (3)
C8—C7—H7	121.2	C14—C15—BR1	118.8 (3)
N1—C8—C7	128.8 (4)	C15—C16—C17	118.4 (4)
N1—C8—C3	108.1 (3)	C15—C16—H16	120.8
C7—C8—C3	123.1 (3)	C17—C16—H16	120.8
C1—C9—C10	113.1 (3)	C16—C17—C12	120.6 (4)
C1—C9—H9A	109	C16—C17—H17	119.7
C10—C9—H9A	109	C12—C17—H17	119.7
C1—C9—H9B	109	C1—N1—C8	110.1 (3)
C10—C9—H9B	109	C1—N1—H1	124.9
H9A—C9—H9B	107.8	C8—N1—H1	124.9

Table 5. Anisotropic displacement parameters ($Å^2$x 10^3) for 6c The anisotropic displacement factor exponent takes the form: $-2\pi^2$[$h^2a^{*2}U^{11}$ + ... + 2 h k a* b* U^{12}]

	U^{11}	U^{22}	U^{33}	U^{23}	U^{13}	U^{12}
C(1)	13(2)	9(2)	11(2)	-1(2)	-2(2)	-1(2)

C(2)	10(2)	9(2)	8(2)	-2(1)	0(2)	-1(2)
C(3)	10(2)	12(2)	7(2)	-1(2)	-4(2)	1(2)
C(4)	13(2)	11(2)	13(2)	-2(2)	-3(2)	0(2)
C(5)	11(2)	20(2)	13(2)	-3(2)	1(2)	-4(2)
C(6)	22(2)	14(2)	13(2)	3(2)	2(2)	-9(2)
C(7)	21(2)	9(2)	13(2)	0(2)	-4(2)	0(2)
C(8)	9(2)	9(2)	12(2)	-4(2)	-2(2)	-3(2)
C(9)	16(2)	12(2)	16(2)	0(2)	5(2)	2(2)
C(10)	12(2)	21(3)	27(3)	1(2)	6(2)	-1(2)
C(11)	9(2)	12(2)	15(2)	0(2)	-3(2)	-4(2)
C(12)	10(2)	10(2)	13(2)	0(2)	-3(2)	-3(2)
C(13)	14(2)	9(2)	15(2)	5(2)	-3(2)	2(2)
C(14)	16(2)	10(2)	10(2)	1(2)	2(2)	-3(2)
C(15)	16(2)	9(2)	20(3)	2(2)	7(2)	6(2)
C(16)	15(2)	24(3)	15(3)	8(2)	-3(2)	7(2)
C(17)	18(2)	17(2)	12(2)	3(2)	-3(2)	0(2)
N(1)	12(2)	9(2)	17(2)	1(2)	0(2)	5(1)
F(1)	17(1)	33(2)	39(2)	-1(1)	13(1)	-7(1)
F(2)	16(1)	31(2)	33(2)	3(1)	-7(1)	2(1)
F(3)	19(1)	16(1)	31(2)	-6(1)	3(1)	-1(1)
BR1	38(1)	28(1)	27(1)	7(1)	13(1)	22(1)
O(1)	15(1)	13(1)	13(2)	1(1)	1(1)	-1(1)

Table 6. Hydrogen coordinates (x 10^4) and isotropic displacement parameters (Å^{2}x 10^3) for 6c

	x	y	z	U(eq)
H(4)	662	3166	4077	15
H(5)	-263	1746	4975	18
H(6)	1058	124	5137	20
H(7)	3394	-98	4422	17
H(9A)	5742	3041	1620	17
H(9B)	6562	1982	1979	17

H(13)	2870	4709	4428	15
H(14)	1229	5971	5121	15
H(16)	-774	6436	2590	22
H(17)	958	5251	1894	18
H(1)	5398	957	3265	15

***ORTEP* View of 6d with 50% ellipsoidal probability**

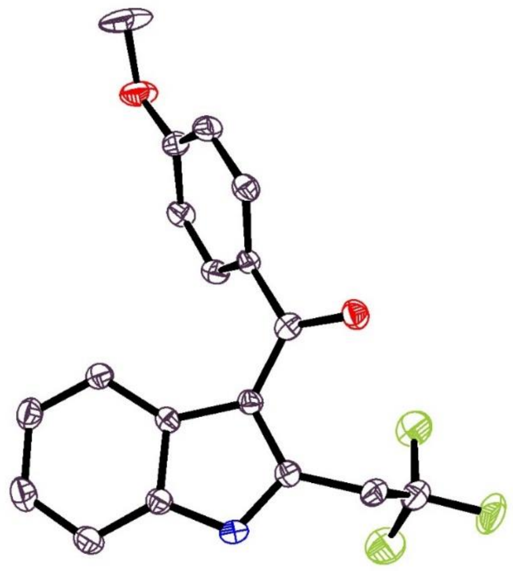

Packing diagram of **6d**

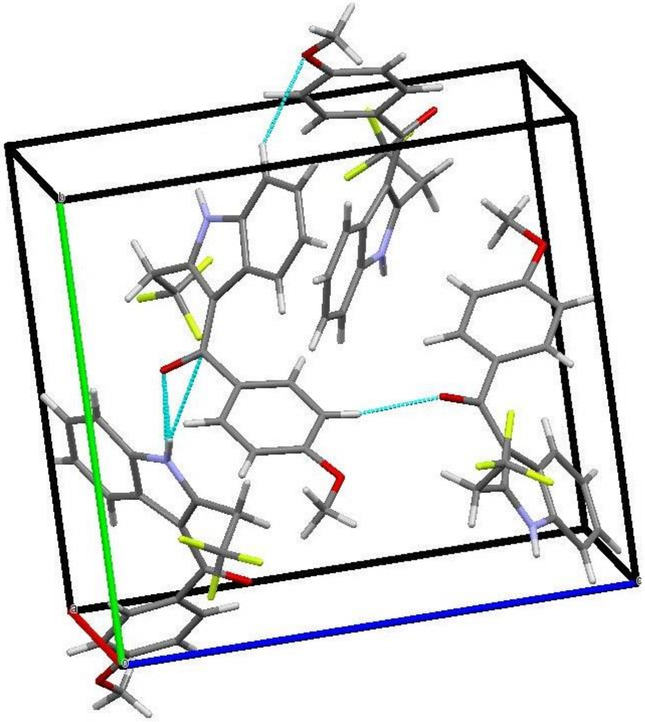

Table 1. Crystal data and structure refinement for 6d

Identification code	SJ-768	
Empirical formula	$C_{18} H_{14} F_3 N O_2$	
Formula weight	333.30	
Temperature	293(2) K	
Wavelength	0.71073 Å	
Crystal system	Orthorhombic	
Space group	$P 2_1 2_1 2_1$	
Unit cell dimensions	a = 8.8605(8) Å	α= 90°.
	b = 12.7157(14) Å	β= 90°.
	c = 13.9809(14) Å	γ = 90°.
Volume	1575.2(3) $Å^3$	
Z	4	
Density (calculated)	1.405 g/cm^3	
Absorption coefficient	0.115 mm^{-1}	
F(000)	688	
Crystal size	0.05 x 0.04 x 0.02 mm^3	
Theta range for data collection	2.722 to 25.114°.	
Index ranges	-10<=h<=10, -12<=k<=15, -16<=l<=16	
Reflections collected	12288	
Independent reflections	2788 [R(int) = 0.0700]	
Completeness to theta = 25.114°	99.1 %	
Absorption correction	None	
Refinement method	Full-matrix least-squares on F^2	
Data / restraints / parameters	2788 / 0 / 218	
Goodness-of-fit on F^2	1.044	
Final R indices [I>2sigma(I)]	R1 = 0.0445, wR2 = 0.0826	
R indices (all data)	R1 = 0.0722, wR2 = 0.0914	
Absolute structure parameter	0.3(5)	
Extinction coefficient	n/a	
Largest diff. peak and hole	0.181 and -0.261 e.$Å^{-3}$	

Table 2. Atomic coordinates (x 10^4) and equivalent isotropic displacement parameters (Å^{2}x 10^3) for 6d. U(eq) is defined as one third of the trace of the orthogonalized U^{ij} tensor

	x	y	z	U(eq)
F(2)	6960(2)	5749(2)	2883(2)	40(1)
F(3)	7961(2)	7193(2)	3367(2)	46(1)
O(1)	3976(2)	5552(2)	1880(2)	24(1)
F(1)	8601(2)	6534(2)	2006(2)	48(1)
O(2)	-791(3)	3334(2)	4587(2)	37(1)
N(1)	4793(3)	8491(2)	3183(2)	24(1)
C(1)	4903(4)	7554(3)	2740(2)	19(1)
C(8)	3480(4)	8544(3)	3726(2)	21(1)
C(14)	1317(4)	4421(3)	4581(2)	23(1)
C(13)	2337(4)	5072(3)	4118(2)	23(1)
C(11)	3368(3)	5890(3)	2624(2)	19(1)
C(2)	3670(3)	6932(3)	2994(2)	19(1)
C(12)	2283(3)	5201(3)	3133(2)	18(1)
C(3)	2745(3)	7571(3)	3623(2)	17(1)
C(5)	772(4)	8272(3)	4605(2)	27(1)
C(4)	1335(4)	7452(3)	4081(2)	23(1)
C(15)	199(4)	3919(3)	4055(3)	24(1)
C(16)	130(4)	4036(3)	3069(2)	26(1)
C(9)	6220(4)	7322(3)	2102(2)	24(1)
C(17)	1191(3)	4659(3)	2615(2)	22(1)
C(7)	2923(4)	9374(3)	4246(2)	26(1)
C(10)	7430(4)	6704(3)	2592(3)	32(1)
C(6)	1544(4)	9216(3)	4692(3)	30(1)
C(18)	-2033(5)	2870(4)	4087(3)	58(1)

Table 3. Selected bond lengths [Å] for 6d

F2—C10	1.347 (4)	C2—C3	1.451 (4)
F3—C10	1.335 (4)	C12—C17	1.392 (4)

O1—C11	1.248 (4)	C3—C4	1.412 (4)
F1—C10	1.340 (4)	C5—C4	1.370 (5)
O2—C15	1.369 (4)	C5—C6	1.387 (5)
O2—C18	1.431 (4)	C5—H5	0.93
N1—C1	1.346 (4)	C4—H4	0.93
N1—C8	1.391 (4)	C15—C16	1.387 (5)
N1—H1	0.86	C16—C17	1.384 (5)
C1—C2	1.395 (5)	C16—H16	0.93
C1—C9	1.499 (5)	C9—C10	1.496 (5)
C8—C7	1.373 (5)	C9—H9A	0.97
C8—C3	1.407 (5)	C9—H9B	0.97
C14—C13	1.385 (5)	C17—H17	0.93
C14—C15	1.390 (5)	C7—C6	1.386 (5)
C14—H14	0.93	C7—H7	0.93
C13—C12	1.388 (5)	C6—H6	0.93
C13—H13	0.93	C18—H18A	0.96
C11—C2	1.447 (5)	C18—H18B	0.96
C11—C12	1.481 (4)	C18—H18C	0.96

Table 4. Selected bond angles [°] for 6d

C15—O2—C18	116.9 (3)	O2—C15—C16	124.7 (3)
C1—N1—C8	110.8 (3)	O2—C15—C14	114.8 (3)
C1—N1—H1	124.6	C16—C15—C14	120.5 (3)

C8—N1—H1	124.6	C17—C16—C15	119.2 (3)
N1—C1—C2	109.2 (3)	C17—C16—H16	120.4
N1—C1—C9	120.3 (3)	C15—C16—H16	120.4
C2—C1—C9	130.6 (3)	C10—C9—C1	112.9 (3)
C7—C8—N1	128.9 (3)	C10—C9—H9A	109
C7—C8—C3	124.3 (3)	C1—C9—H9A	109
N1—C8—C3	106.8 (3)	C10—C9—H9B	109
C13—C14—C15	119.4 (3)	C1—C9—H9B	109
C13—C14—H14	120.3	H9A—C9—H9B	107.8
C15—C14—H14	120.3	C16—C17—C12	121.2 (3)
C14—C13—C12	120.9 (3)	C16—C17—H17	119.4
C14—C13—H13	119.6	C12—C17—H17	119.4
C12—C13—H13	119.6	C8—C7—C6	116.4 (3)
O1—C11—C2	122.2 (3)	C8—C7—H7	121.8
O1—C11—C12	118.5 (3)	C6—C7—H7	121.8
C2—C11—C12	119.3 (3)	F3—C10—F1	107.4 (3)
C1—C2—C11	125.0 (3)	F3—C10—F2	106.5 (3)
C1—C2—C3	106.2 (3)	F1—C10—F2	106.2 (3)
C11—C2—C3	128.6 (3)	F3—C10—C9	112.3 (3)
C13—C12—C17	118.7 (3)	F1—C10—C9	111.1 (3)
C13—C12—C11	121.6 (3)	F2—C10—C9	113.0 (3)
C17—C12—C11	119.7 (3)	C7—C6—C5	121.4 (3)

C8—C3—C4	117.2 (3)	C7—C6—H6	119.3
C8—C3—C2	107.0 (3)	C5—C6—H6	119.3
C4—C3—C2	135.6 (3)	O2—C18—H18A	109.5
C4—C5—C6	121.8 (3)	O2—C18—H18B	109.5
C4—C5—H5	119.1	H18A—C18—H18B	109.5
C6—C5—H5	119.1	O2—C18—H18C	109.5
C5—C4—C3	118.9 (3)	H18A—C18—H18C	109.5
C5—C4—H4	120.5	H18B—C18—H18C	109.5
C3—C4—H4	120.5		

Table 5. Anisotropic displacement parameters ($Å^2$x 10^3) for 6d The anisotropic displacement factor exponent takes the form: $-2\pi^2$[$h^2a^{*2}U^{11}$ + ... + 2 h k a* b* U^{12}]

	U^{11}	U^{22}	U^{33}	U^{23}	U^{13}	U^{12}
F(2)	32(1)	31(2)	56(1)	12(1)	1(1)	6(1)
F(3)	31(1)	54(2)	53(1)	-5(1)	-13(1)	-2(1)
O(1)	28(1)	23(2)	22(1)	-2(1)	3(1)	1(1)
F(1)	24(1)	48(2)	71(2)	2(1)	18(1)	8(1)
O(2)	41(2)	37(2)	34(2)	-2(1)	5(1)	-20(1)
N(1)	21(2)	20(2)	30(2)	-2(2)	2(1)	-5(1)
C(1)	19(2)	18(2)	20(2)	2(2)	-1(2)	2(2)
C(8)	20(2)	20(2)	23(2)	-1(2)	-2(2)	0(2)
C(14)	30(2)	20(2)	20(2)	1(2)	1(2)	-1(2)
C(13)	23(2)	19(2)	25(2)	-1(2)	-5(2)	0(2)
C(11)	16(2)	23(2)	18(2)	4(2)	-6(2)	2(2)
C(2)	18(2)	17(2)	22(2)	0(2)	-1(2)	1(2)
C(12)	18(2)	16(2)	22(2)	-2(2)	0(2)	1(2)
C(3)	18(2)	16(2)	17(2)	1(2)	-4(2)	3(2)
C(5)	24(2)	30(3)	28(2)	0(2)	7(2)	2(2)

C(4)	22(2)	23(2)	24(2)	-2(2)	2(2)	-3(2)
C(15)	24(2)	19(2)	28(2)	1(2)	6(2)	-4(2)
C(16)	23(2)	26(2)	30(2)	-6(2)	-5(2)	-2(2)
C(9)	22(2)	23(2)	27(2)	2(2)	4(2)	-2(2)
C(17)	25(2)	23(2)	19(2)	-3(2)	-1(2)	1(2)
C(7)	30(2)	20(2)	30(2)	-3(2)	-1(2)	-1(2)
C(10)	24(2)	31(3)	41(2)	0(2)	7(2)	-2(2)
C(6)	34(2)	28(3)	29(2)	-4(2)	6(2)	7(2)
C(18)	57(3)	63(4)	53(3)	4(3)	-5(3)	-46(3)

Table 6. Hydrogen coordinates (x 10^4) and isotropic displacement parameters (Å^{2}x 10^3) for 6d

	x	y	z	U(eq)
H(1)	5446	8989	3136	28
H(14)	1380	4322	5239	28
H(13)	3067	5425	4471	27
H(5)	-154	8195	4911	33
H(4)	798	6826	4027	28
H(16)	-619	3700	2718	32
H(9A)	5870	6932	1549	29
H(9B)	6644	7980	1875	29
H(17)	1174	4716	1952	27
H(7)	3442	10007	4297	32
H(6)	1128	9755	5057	36
H(18A)	-2560	3404	3734	87
H(18B)	-2709	2551	4539	87
H(18C)	-1664	2344	3654	87

References

1) (a) L. J. Hounjet, C. Bannwarth, C. N. Garon, C. B. Caputo, S. Grimme and D. W. Stephan, *Angew. Chem. Inter. Ed*. 2013, **52**, 7492 (b) K. Urakawa, M. Sumimoto, M. Arisawa, M. Matsuda and H Ishikawa, *Angew. Chem. Int. Ed.*, 2016, **55**, 7432.
2) (a) Z. Li, Y. Zhang, L. Zhang and Z.-Q. Liu, *Org. Lett*., 2014, **16**, 382. (b) Z. Hang, Z. Li and Z.-Q. Liu, *Org. Lett.*, 2014, **16**, 3648. (c) S. Chen, D.-Y. Li, L.-L. Jiang, K. Liu and P.-N. Liu, *Org. Lett.*, 2017, **19**, 2014.
3) M. A. Cismesia and T. P. Yoon, *Chem. Sci.*, 2015, **6**, 5426.
4) N. Sakiyama, K. Noguchi and K. Tanaka, *Angew. Chem. Int. Ed.* **2012**, *51*, 5976.
5) M. Hu, R. -J. Song and J. -H. Li, *Angew. Chem. Int. Ed.* **2015**, *54*, 608.
6) M. Hu, R. -J. Song, X.-H. Ouyang, F. –L. Tan, W. –T. Wei and J. -H. Li, *Chem. Commun*. **2016**, *52*, 3328.
7) M. Hu, B. Liu, X.-H. Ouyang, R.-J. Song and J.-H. Li, *Adv. Synth. & Catal*. **2015**, 357, 3332.
8) Q.-H. Chen, P. N. Praveen Rao and E. E. Knaus, *Bioorg. Med. Chem*. **2005**, *13*, 6425.
9) Z. Pan, S. M. Pound, N. R. Rondla and C. J. Douglas, *Angew. Chem. Int. Ed.* **2014**, *53*, 5170.
10) M. Paraja and C. Valdes, *Chem. Commun.* **2016**, *52*, 6312.
11) J. Zhu, Y.-J. Cheng, X.-K. Kuang, L. Wang, Z.-B. Zheng and Y. Tang, *Angew. Chem. Int. Ed.* **2016**, *55*, 9224.
12) W.-I. Lee, J.-W. Jung, J. Sim, H. An and Y.-G. Suh, *Tetrahedron* **2013**, *69*, 7211.
13) M. F. Wempe, et al. *J. Med. Chem.* **2011**, *54*, 2701.
14) X.-H. Xu, G.-K. Liu, A. Azuma, E. Tokunaga and N. Shibata, *Org. Lett.* **2011**, *13*, 4854.
15) Gaussian 09, Revision A.02, M. J. Frisch, G. W. Trucks, H. B. Schlegel et al. Gaussian, Inc., Wallingford CT, 2009
