# Supplementary material for: Visible-light-induced oxidant and metal-free dehydrogenative cascade trifluoromethylation and oxidation of 1,6-enynes with water
Source: Chem Sci. 2017 Jul 10;8(9):6633–44. doi: 10.1039/c7sc02556d (PMC5625288; doi:10.1039/c7sc02556d)

Electronic Supplementary Material (ESI) for Chemical Science.
This journal is © The Royal Society of Chemistry 2017

Supporting Information - II

Visible-Light-Induced Oxidant and Metal-Free Dehydrogenative Cascade Trifluoromethylation and Oxidation of 1,6-Enynes with Water

Sadhan Jana, Ajay Verma, Rahul Kadu and Sangit Kumar*

Department of Chemistry, Indian Institute of Science Education and Research (IISER) Bhopal,

Bhopal By-pass Road, Bhauri, Bhopal, Madhya Pradesh, India, 462 066

E-mail: sangitkumar@iiserb.ac.in

	Page
Table of Contents	S1
^{1}H, ^{13}C, NMR and HRMS Spectra of Starting Materials	S2-S86
^{1}H, ^{13}C, ^{19}F NMR and HRMS Spectra of Products	S87-S263

^{1}H NMR of 1-(2-bromoethoxy)-2-iodobenzene

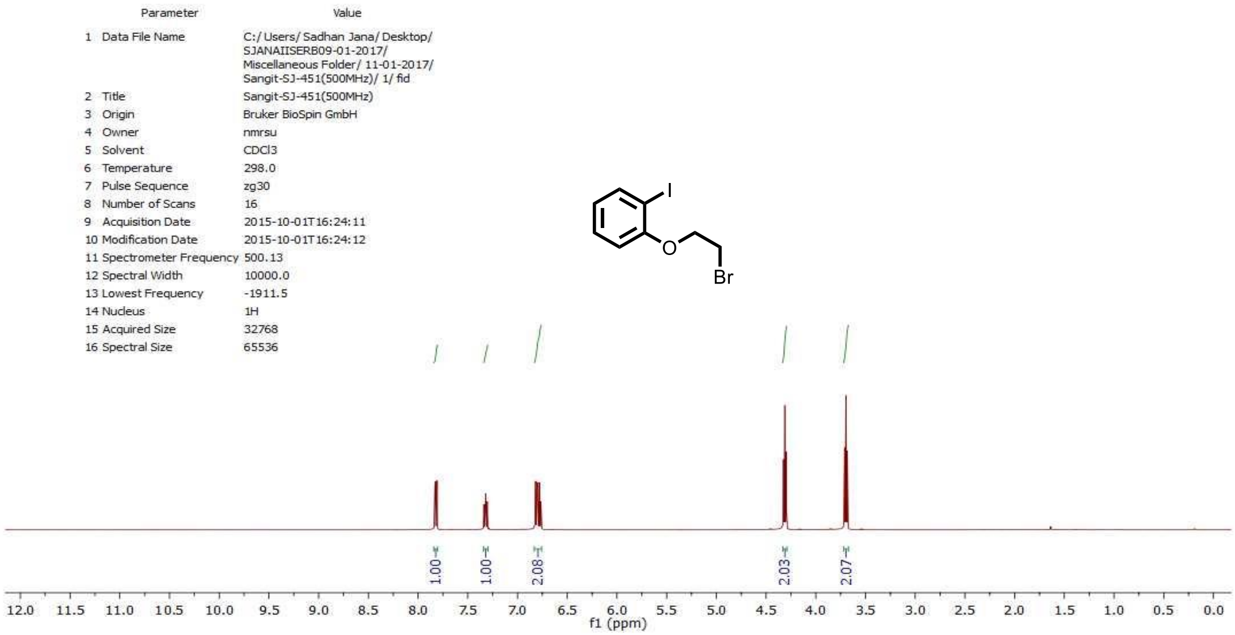

^{13}C NMR of 1-(2-bromoethoxy)-2-iodobenzene

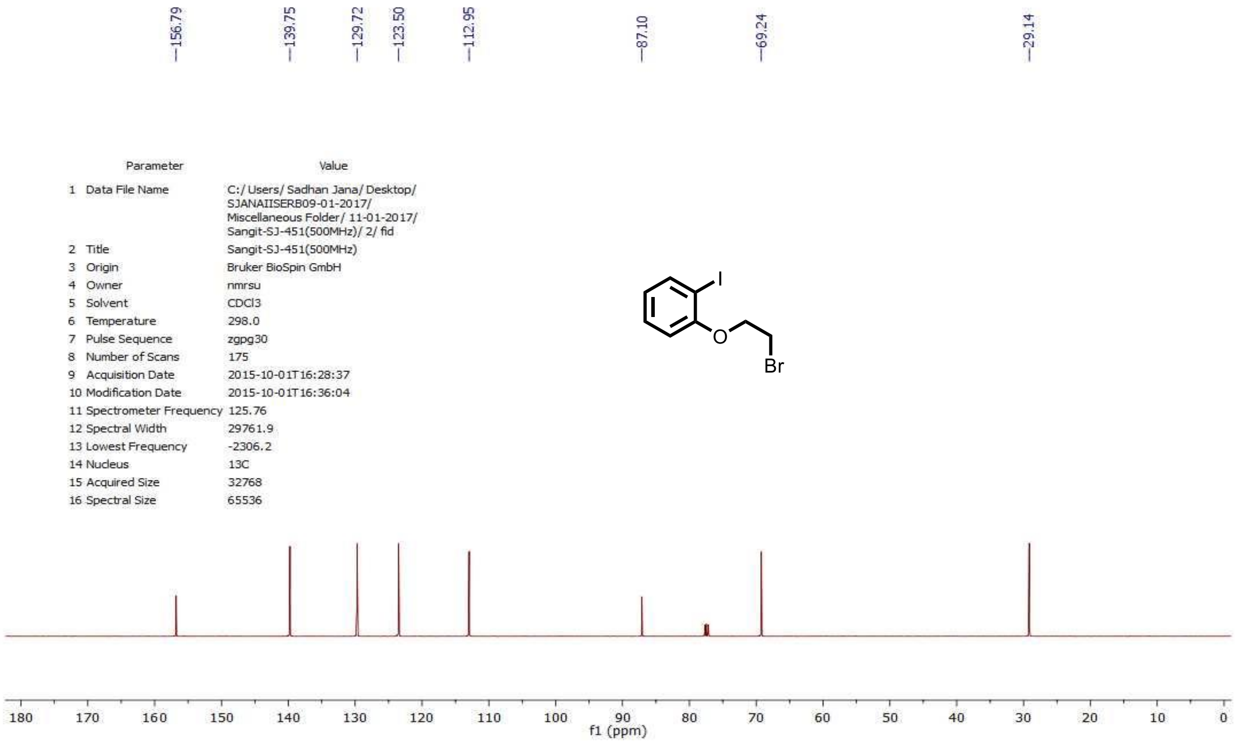

^{1}H NMR of 1-iodo-2-(vinyloxy)benzene

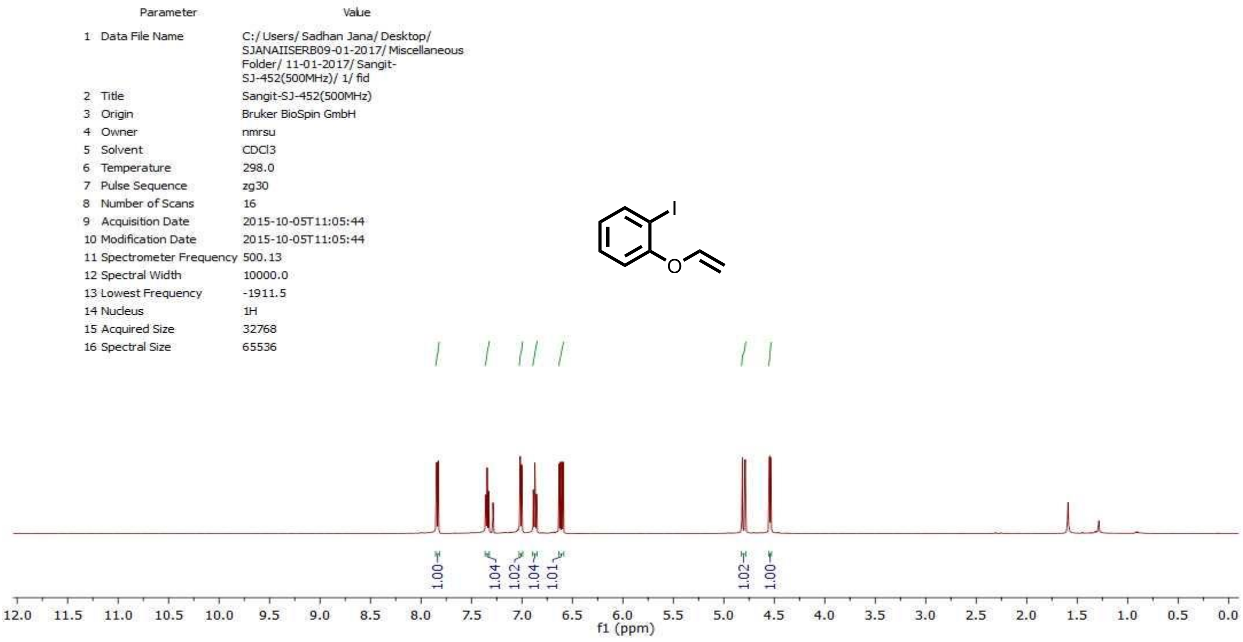

^{13}C NMR of 1-iodo-2-(vinyloxy)benzene

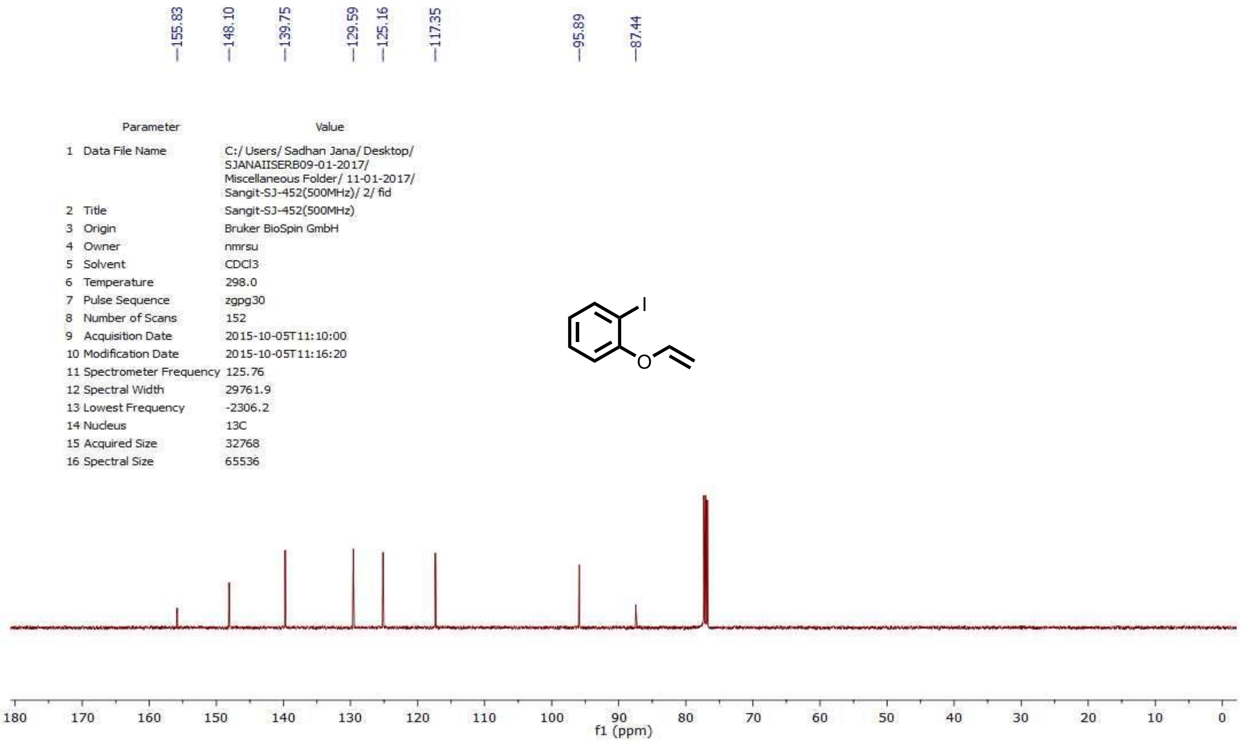

^{1}H NMR of 1-(phenylethynyl)-2-(vinyloxy) benzene

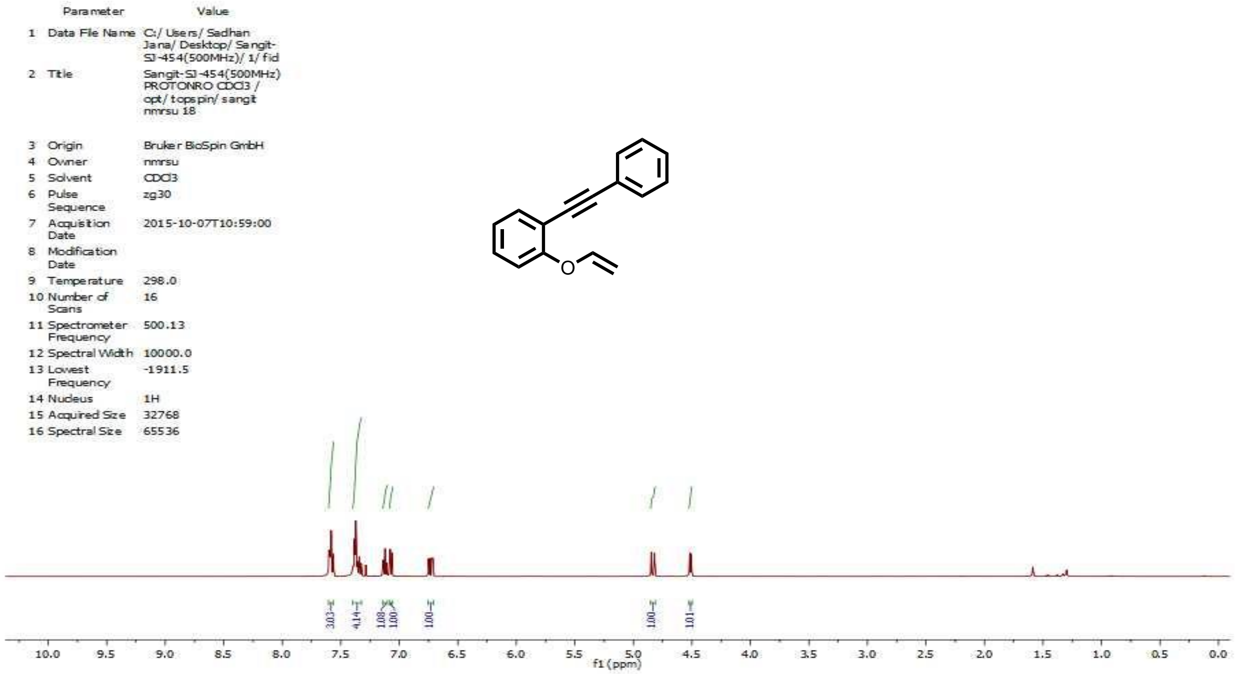

^{13}C NMR of 1-(phenylethynyl)-2-(vinyloxy) benzene

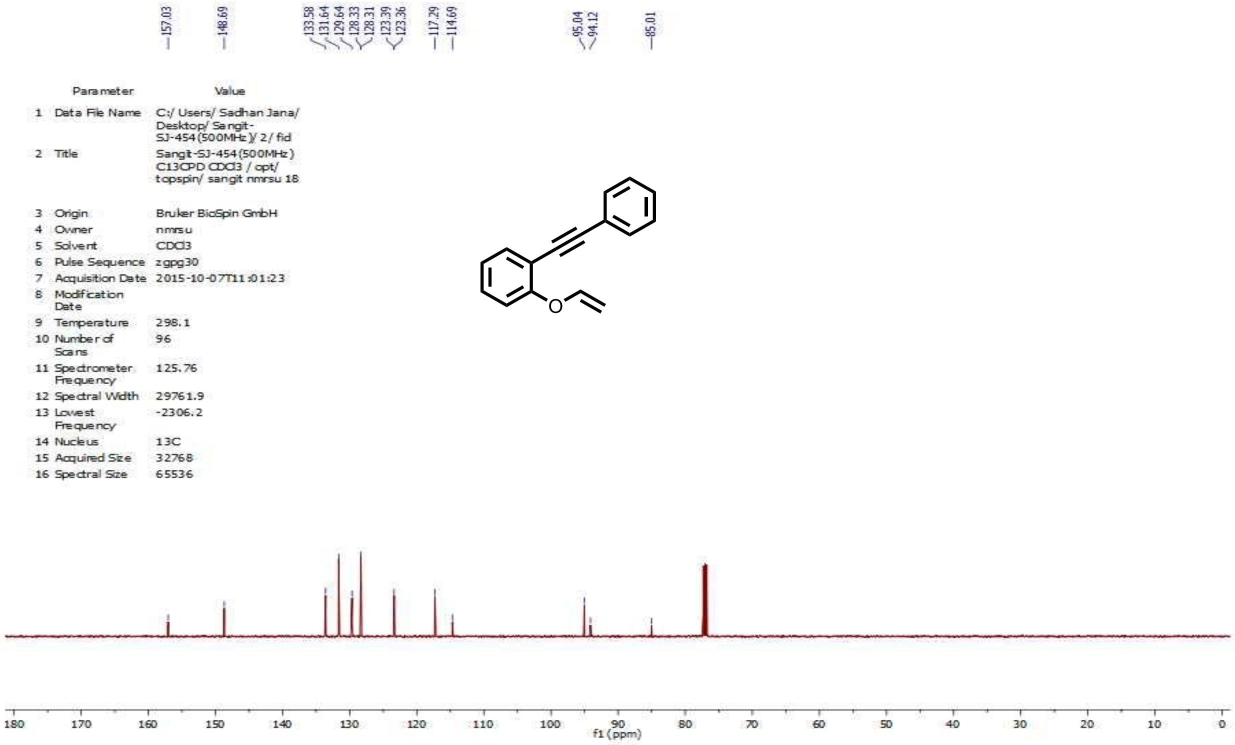

^{1}H NMR of 1-((4-fluorophenyl)ethynyl)-2-(vinyloxy)benzene

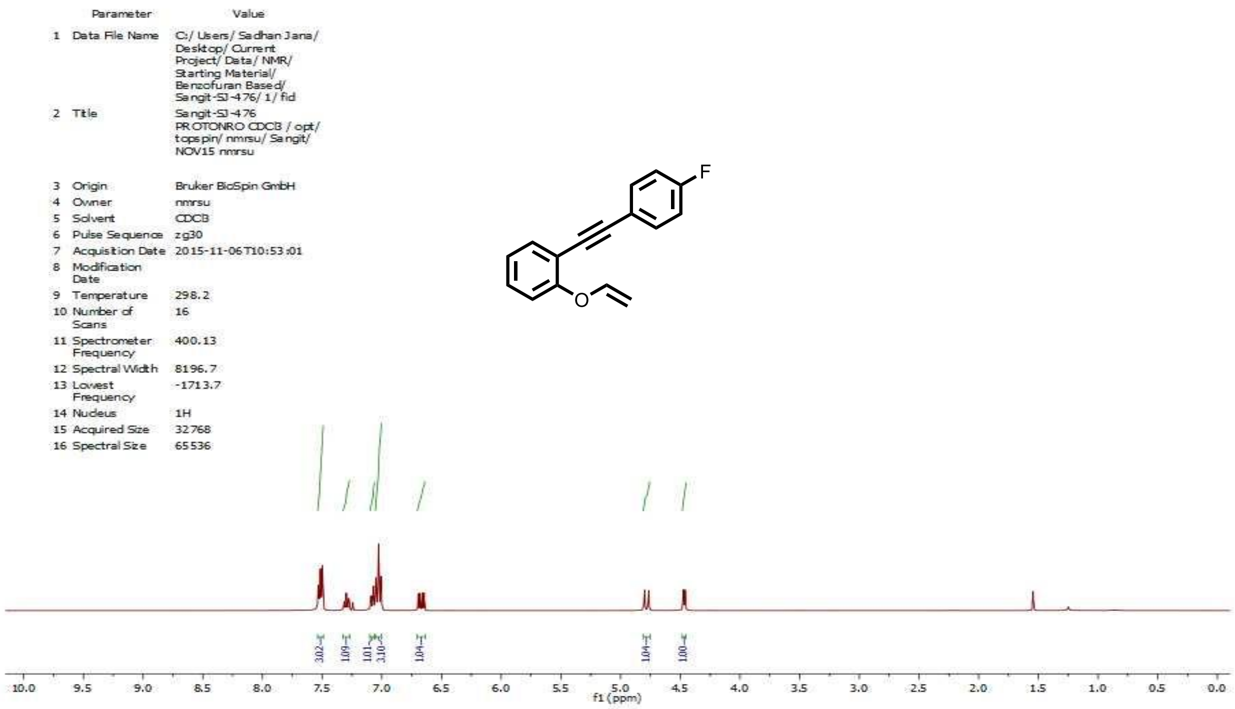

^{13}C NMR of 1-((4-fluorophenyl)ethynyl)-2-(vinyloxy)benzene

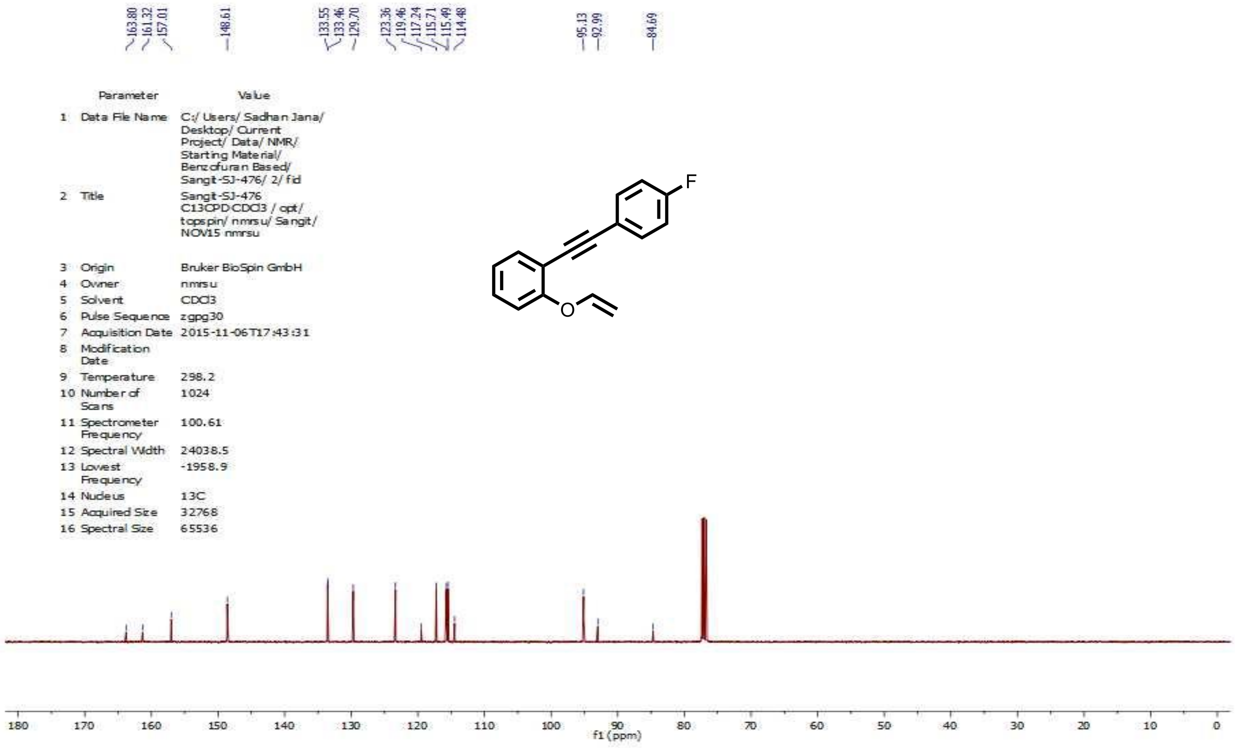

^{1}H NMR of 1-((4-methoxyphenyl)ethynyl)-2-(vinyloxy)benzene

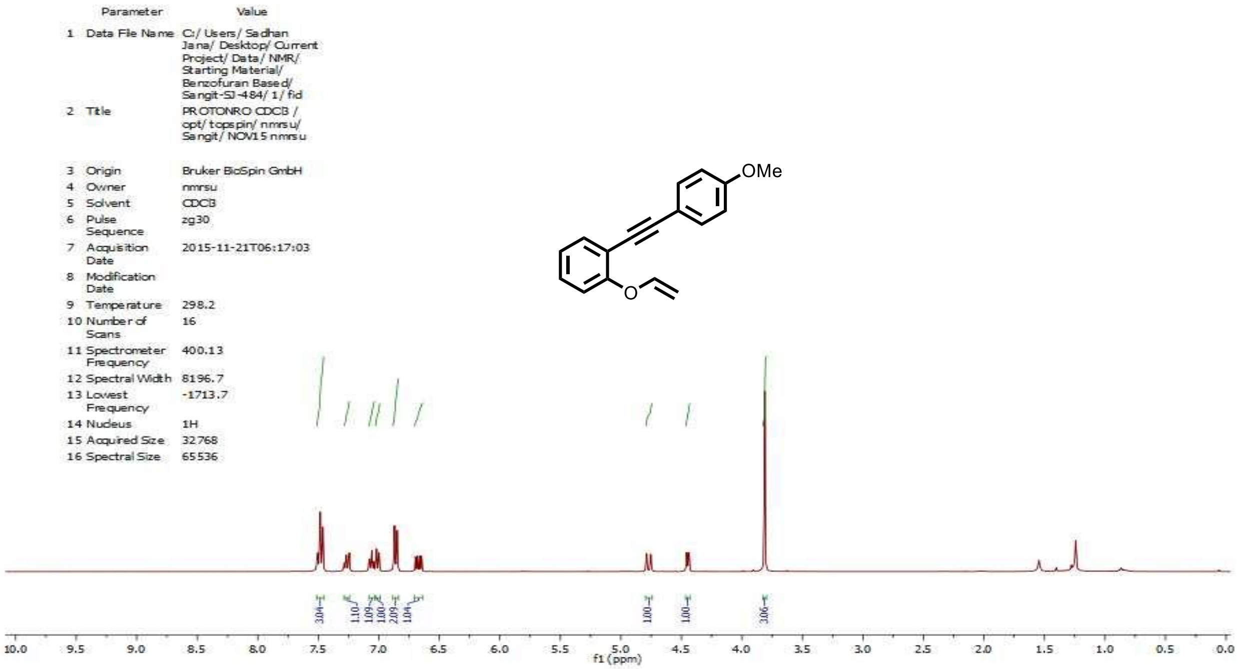

^{13}C NMR of 1-((4-methoxyphenyl)ethynyl)-2-(vinyloxy)benzene

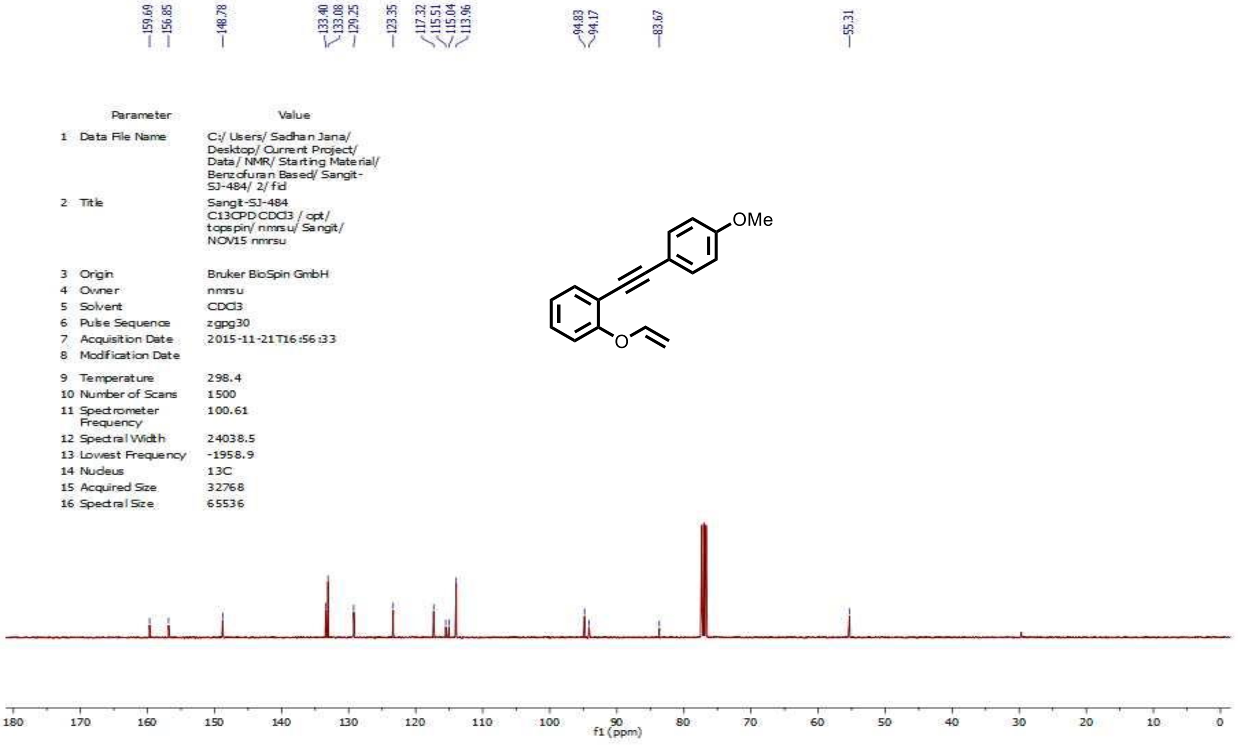

^{1}H NMR of 4-((2-(vinyloxy)phenyl)ethynyl)phenol

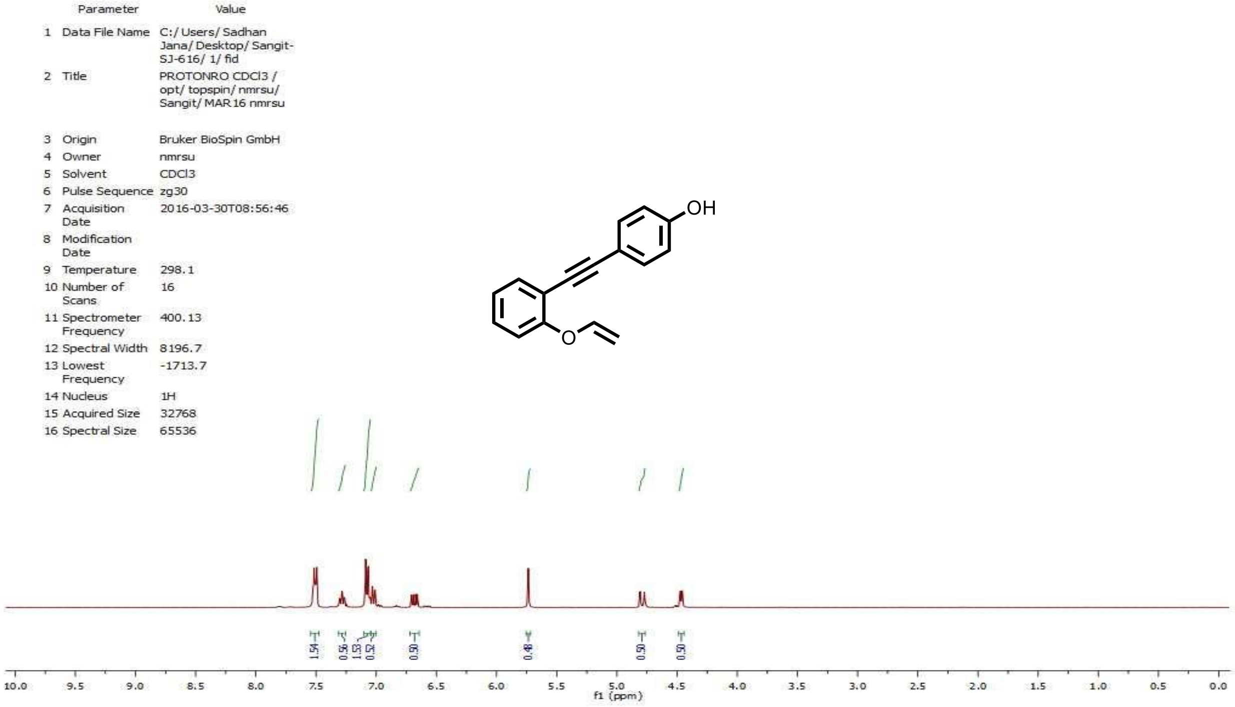

^{13}C NMR of 4-((2-(vinyloxy)phenyl)ethynyl)phenol

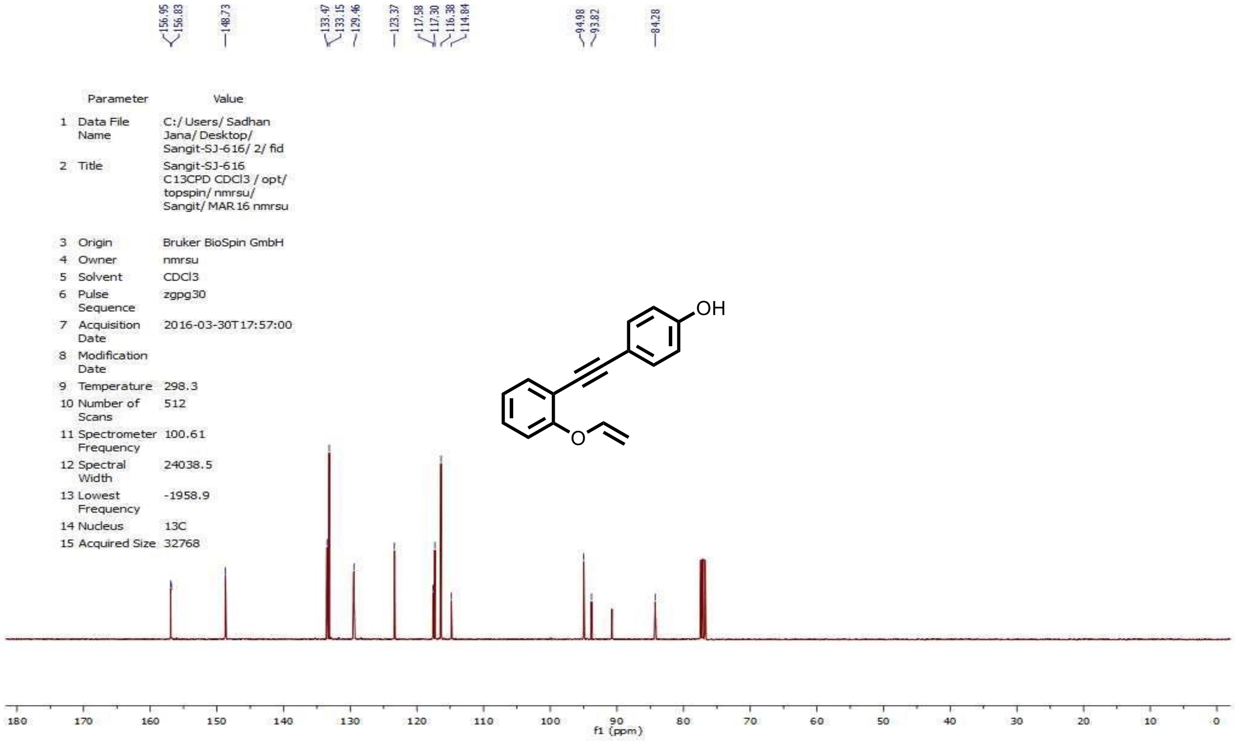

HRMS of 4-((2-(vinyloxy)phenyl)ethynyl)phenol

Display Report

Analysis Info

Analysis Name	D:\Data\user data\2016\SEPT-2016\12-09-2016\Dr.S.Kumar-SJ-616-VE.d	Acquisition Date	9/12/2016 3:57:16 PM
Method	tune_low_APCI.m	Operator	DIMPLE
Sample Name	SJ-616-VE	Instrument	micrOTOF-Q II 10330
Comment			

Acquisition Parameter

Source Type	APCI	Ion Polarity	Negative	Set Nebulizer	3.1 Bar
Focus	Not active	Set Capillary	3500 V	Set Dry Heater	200 °C
Scan Begin	50 m/z	Set End Plate Offset	-500 V	Set Dry Gas	8.0 l/min
Scan End	3000 m/z	Set Collision Cell RF	130.0 Vpp	Set Divert Valve	Waste

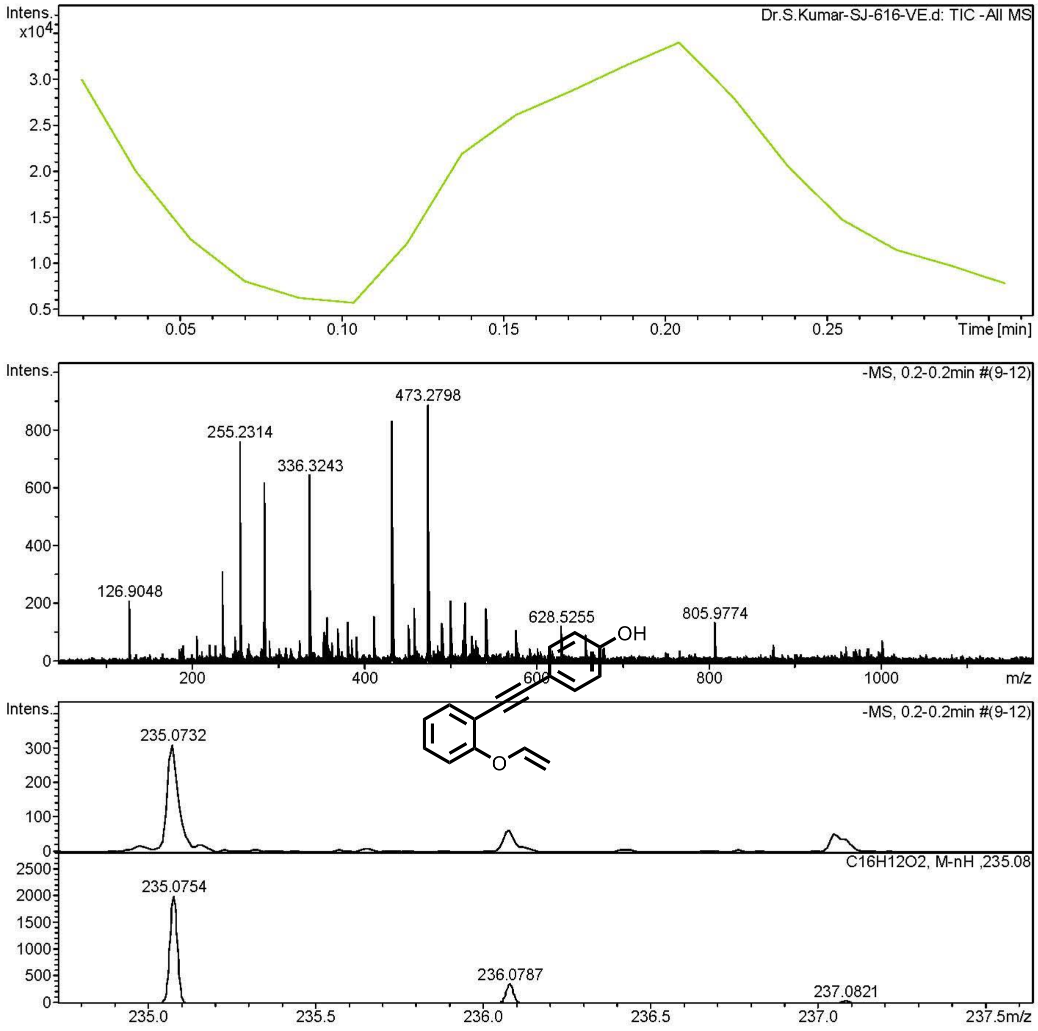

^{1}H NMR of 1-((3-chlorophenyl)ethynyl)-2-(vinyloxy)benzene

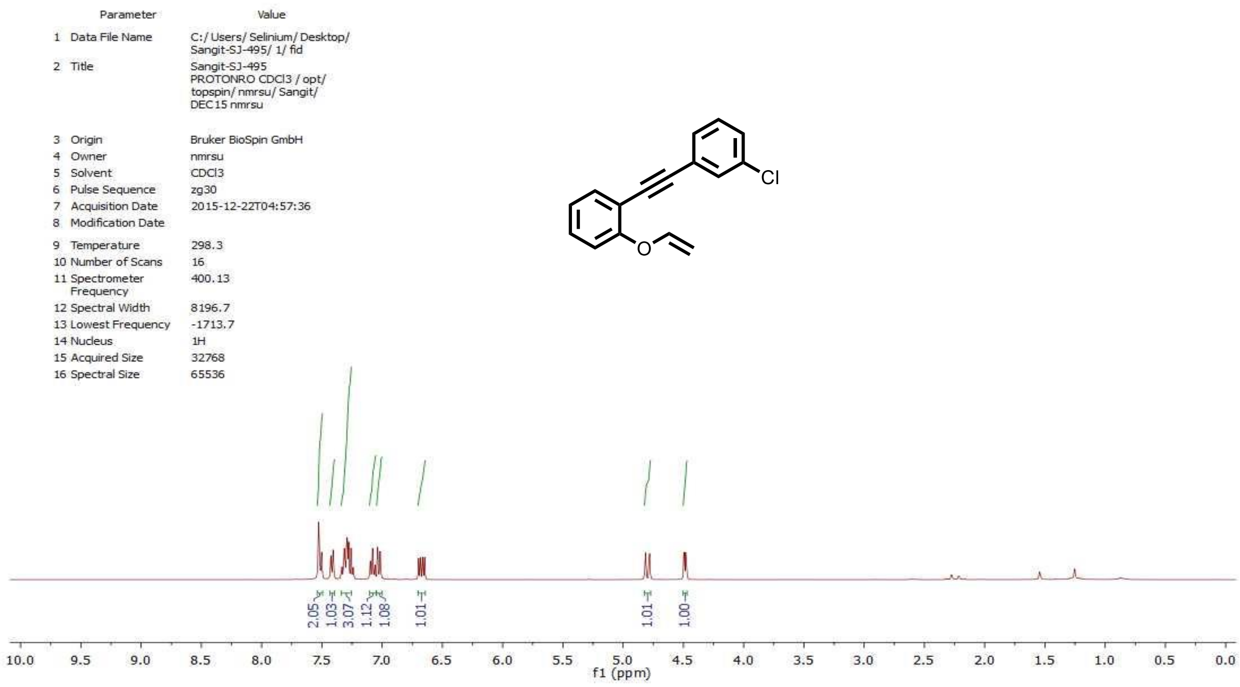

^{13}C NMR of 1-((3-chlorophenyl)ethynyl)-2-(vinyloxy)benzene

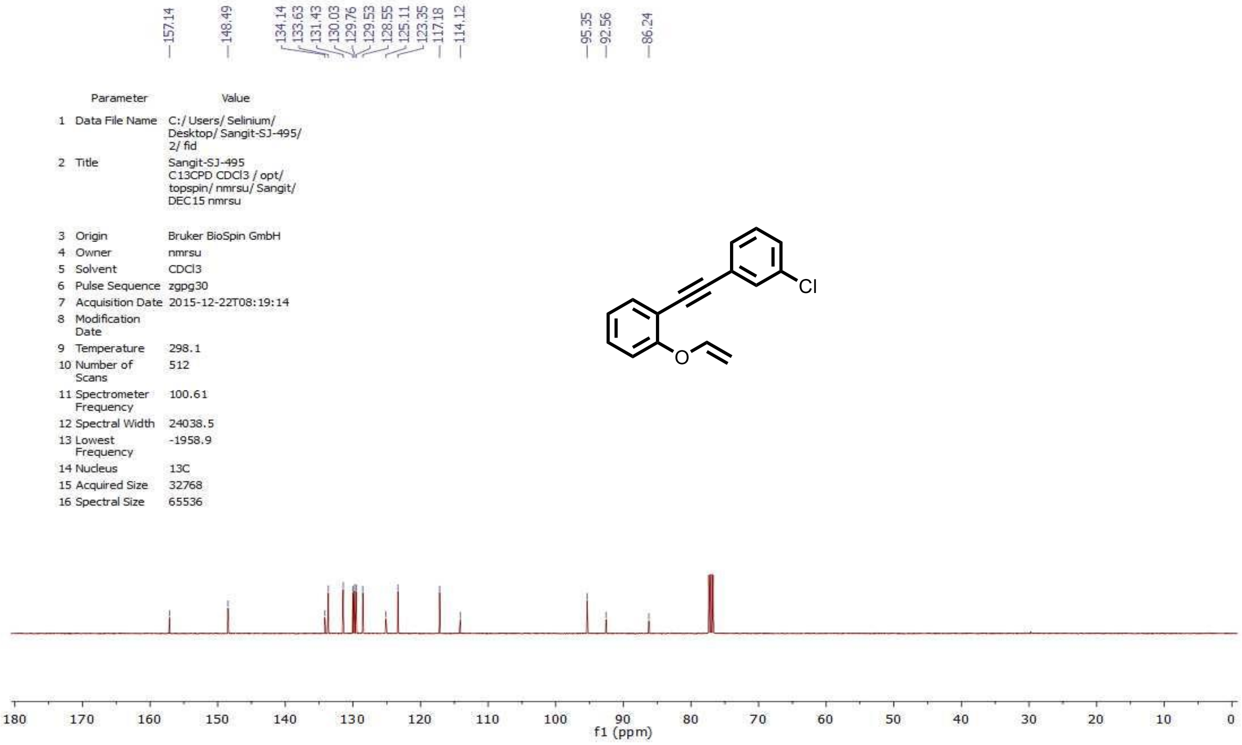

HRMS of 1-((3-chlorophenyl)ethynyl)-2-(vinyloxy)benzene

Display Report

Analysis Info

Analysis Name	D:\Data\user data\2016\SEPT-2016\06-sep-2016\Dr.S.Kumar-SJ-495_1-A,8_01_7318.d	Acquisition Date	9/6/2016 2:22:26 PM
Method	HRLCMS-20 Sept.m	Operator	DIMPLE
Sample Name	Dr.S.Kumar-SJ-495	Instrument	micrOTOF-Q II 10330
Comment			

Acquisition Parameter

Source Type	ESI	Ion Polarity	Positive	Set Nebulizer	1.2 Bar
Focus	Active	Set Capillary	4500 V	Set Dry Heater	200 °C
Scan Begin	50 m/z	Set End Plate Offset	-500 V	Set Dry Gas	7.0 l/min
Scan End	3000 m/z	Set Collision Cell RF	130.0 Vpp	Set Divert Valve	Waste

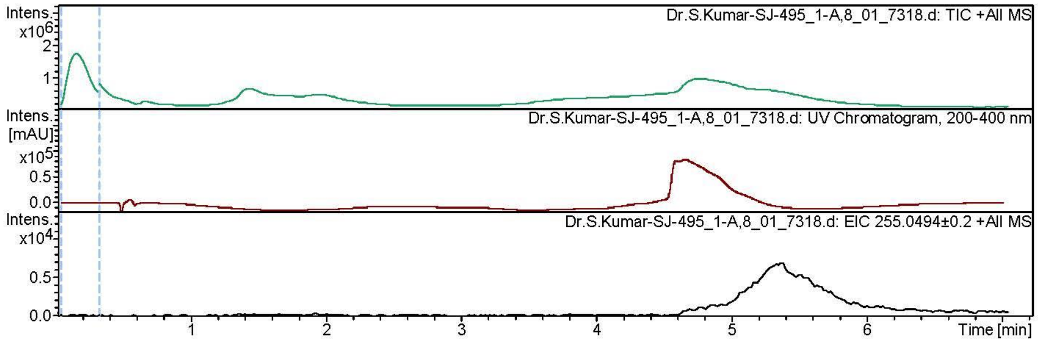

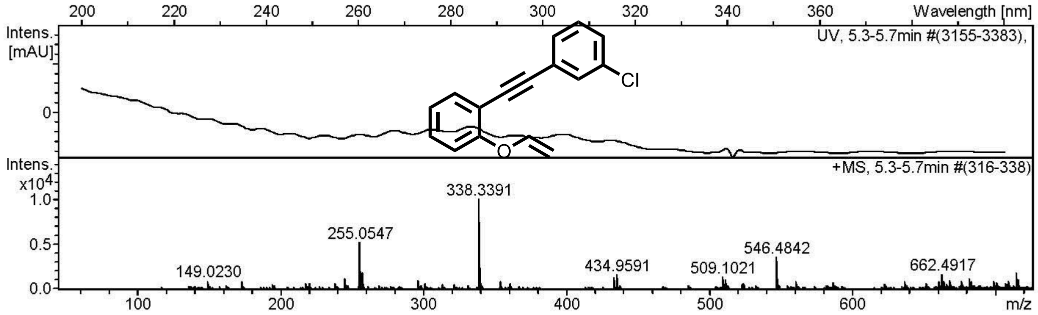

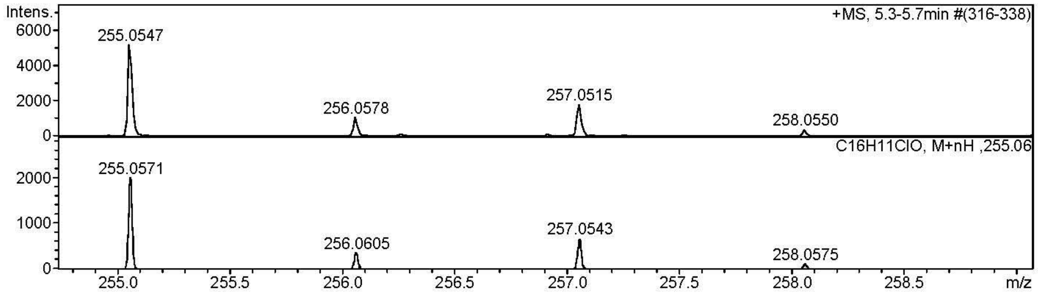

Bruker Compass DataAnalysis 4.0 printed: 9/6/2016 3:30:21 PM Page 1 of 1

^{1}H NMR of 1-(m-tolylethynyl)-2-(vinyloxy)benzene

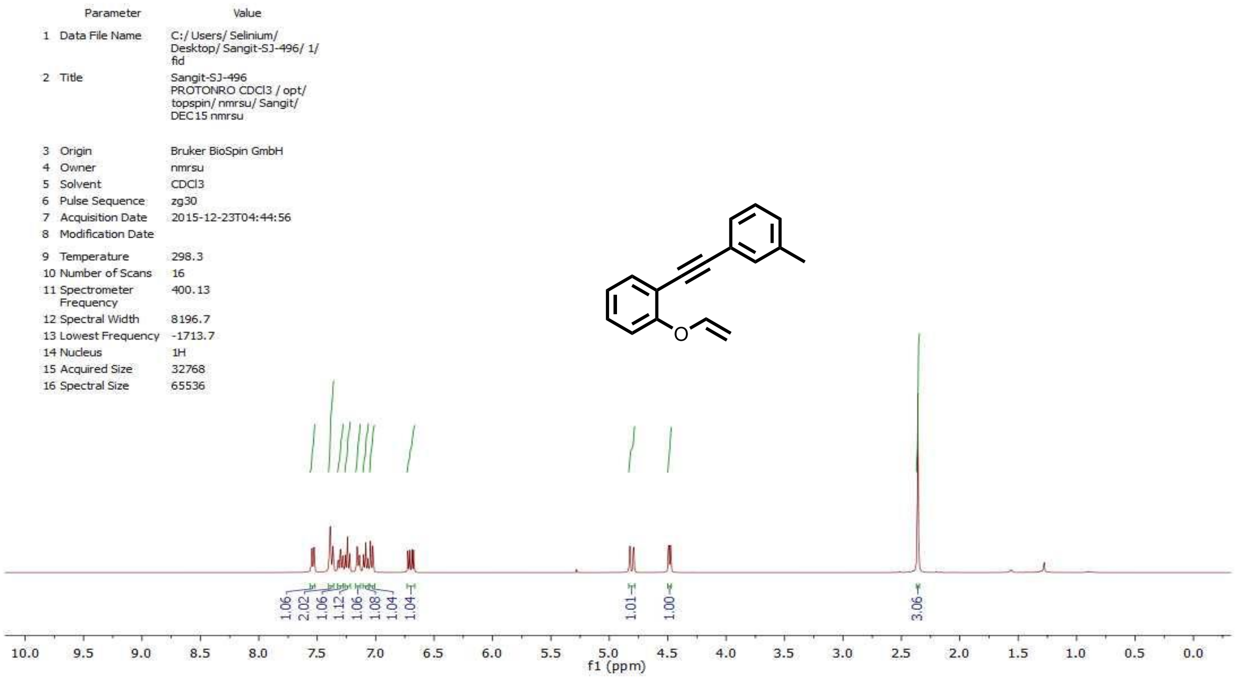

^{13}C NMR of 1-(m-tolylethynyl)-2-(vinyloxy)benzene

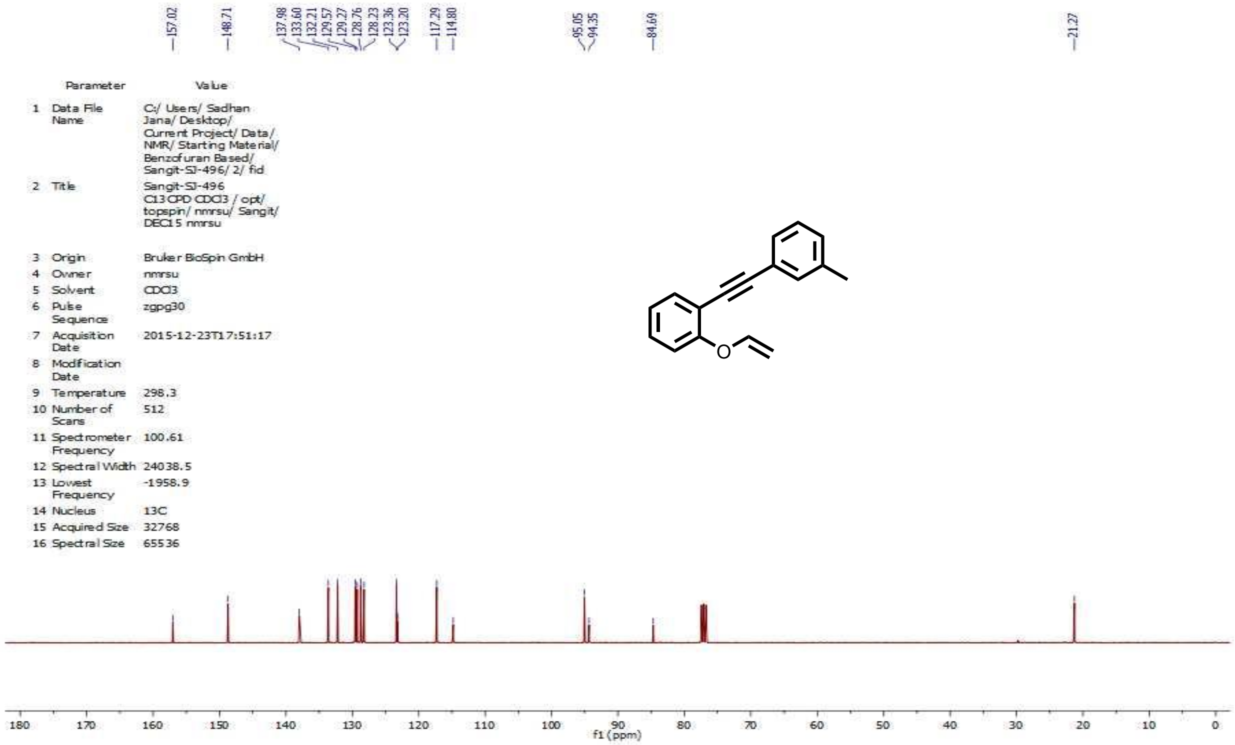

^{1}H NMR of 1-methoxy-2-((2-(vinyloxy)phenyl)ethynyl)benzene

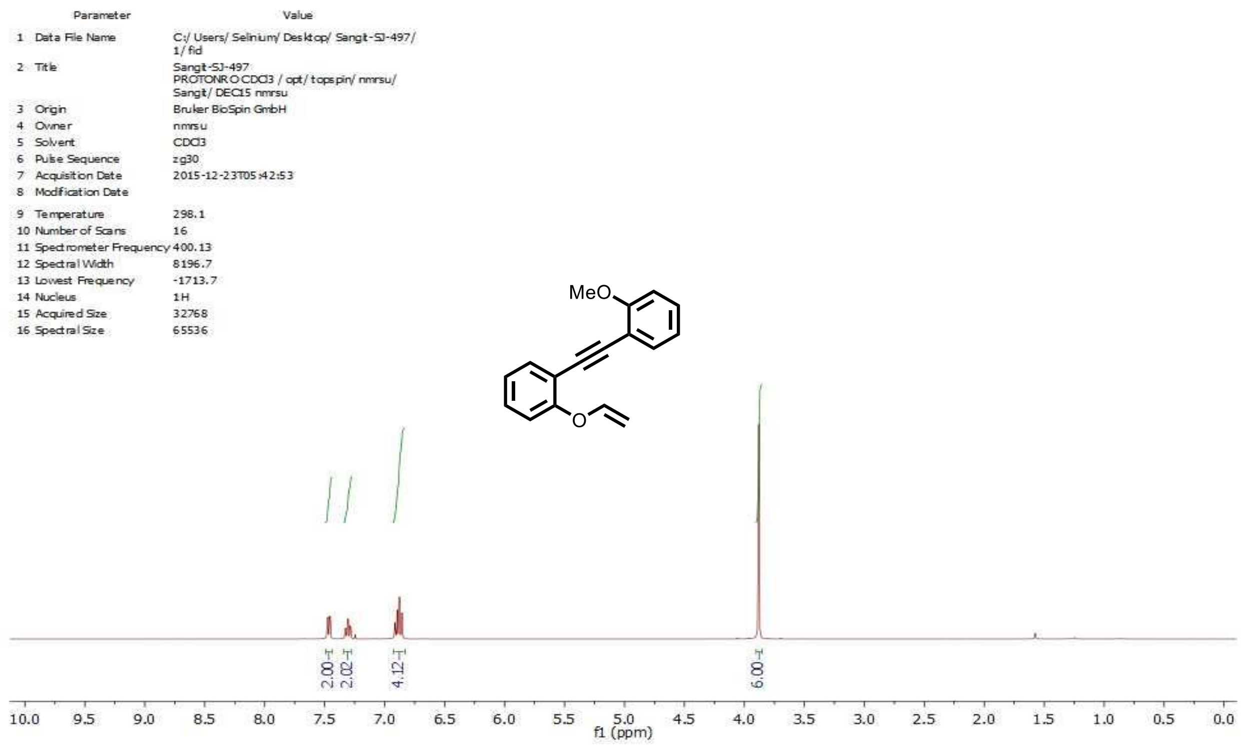

^{13}C NMR of 1-methoxy-2-((2-(vinyloxy)phenyl)ethynyl)benzene

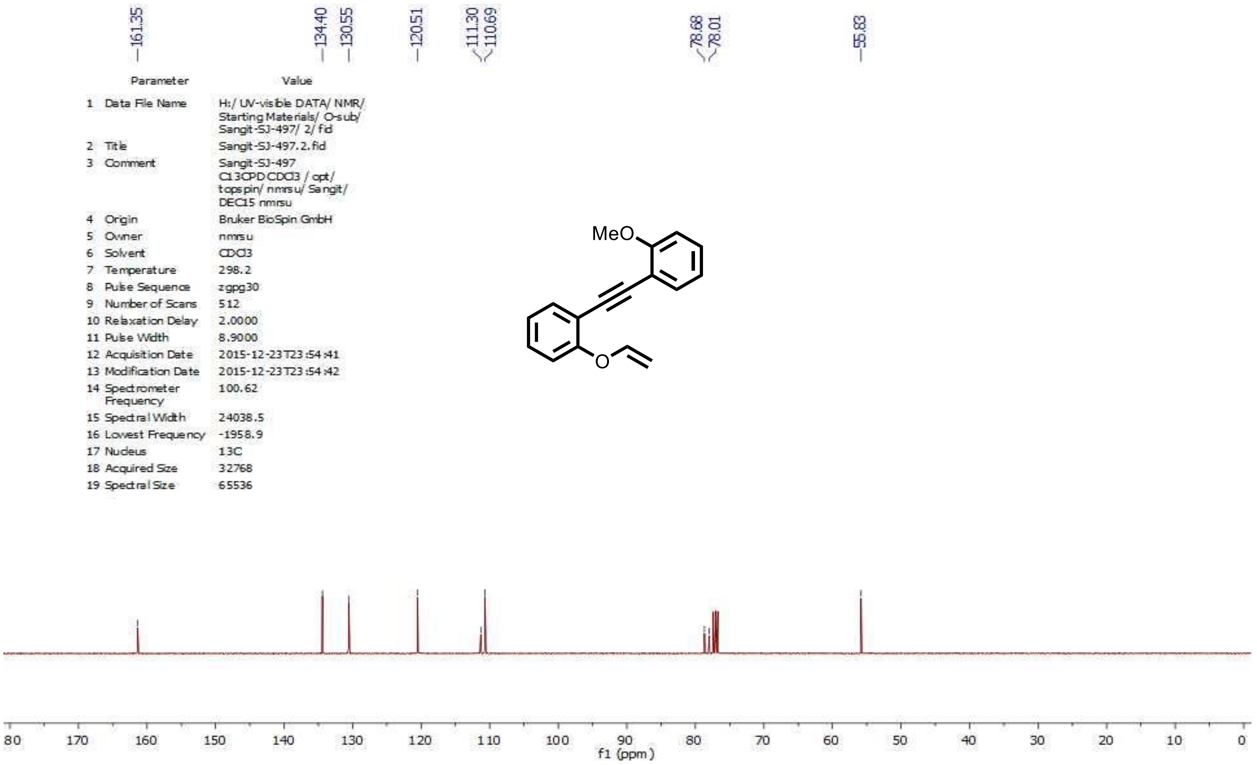

^{1}H NMR of 1,2-dimethoxy-4-((2-(vinyloxy)phenyl)ethynyl)benzene

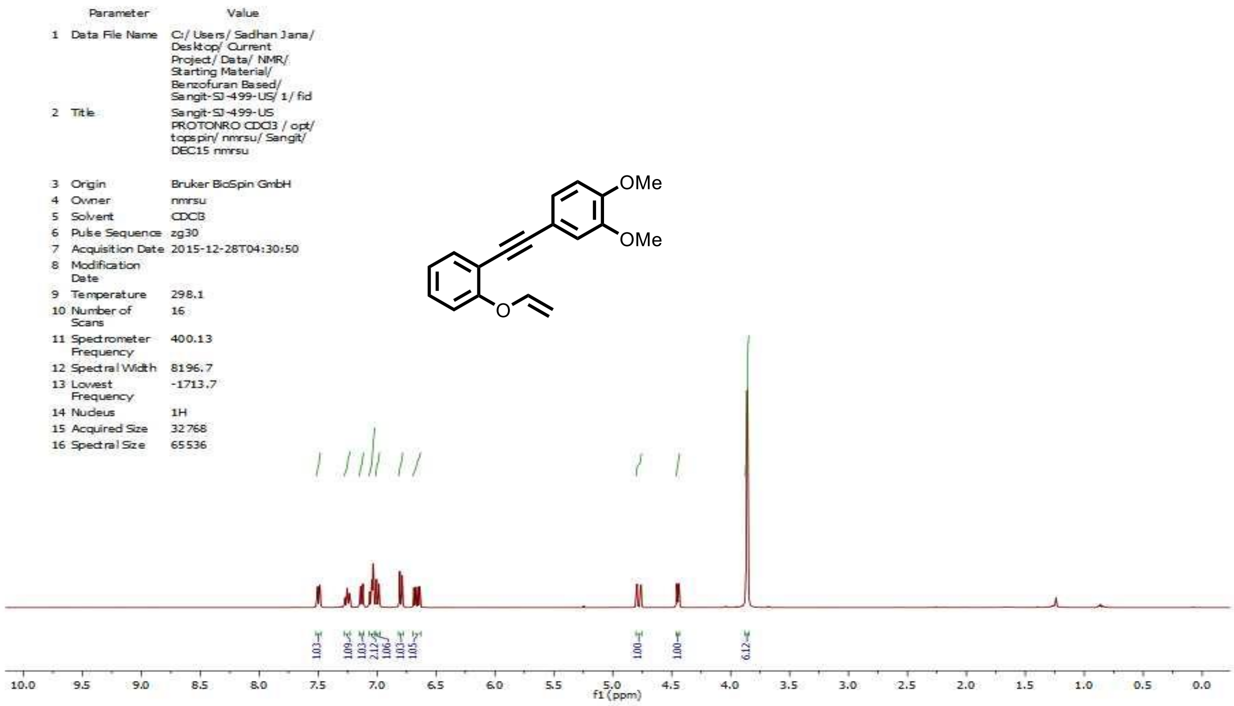

^{13}C NMR of 1,2-dimethoxy-4-((2-(vinyloxy)phenyl)ethynyl)benzene

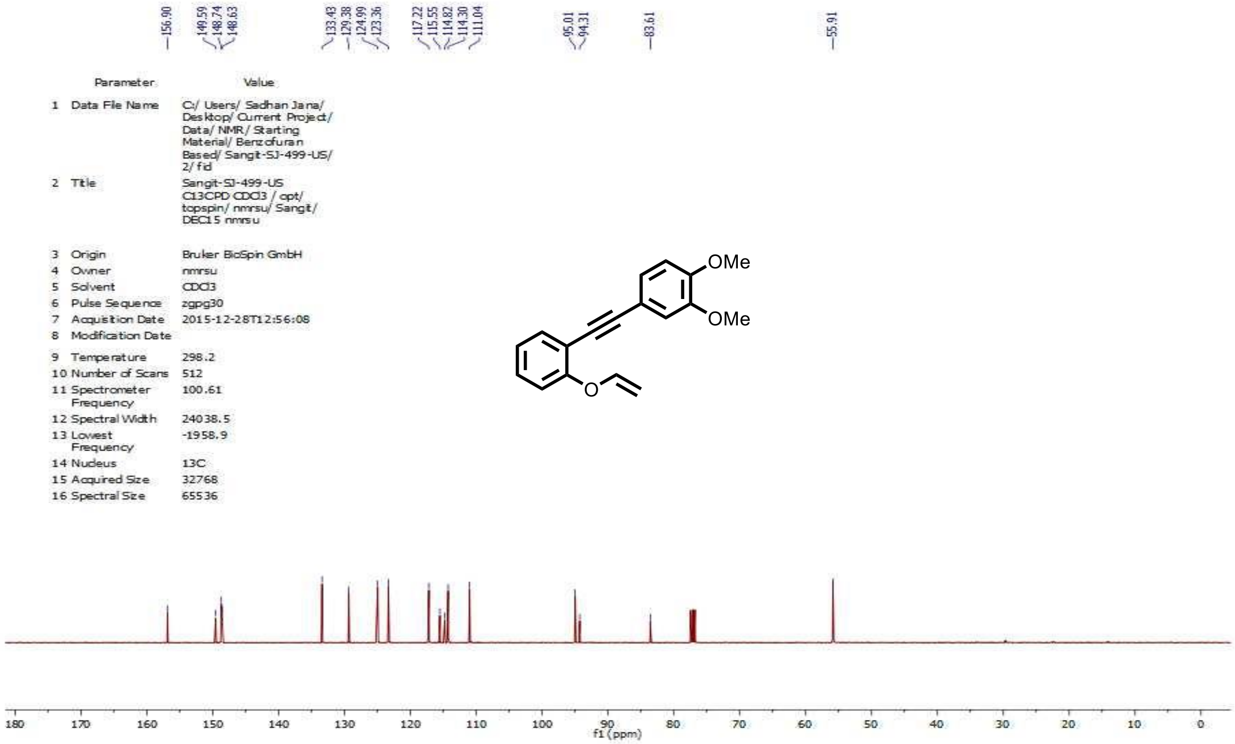

HRMS of 1,2-dimethoxy-4-((2-(vinyloxy)phenyl)ethynyl)benzene

Display Report

Analysis Info

Analysis Name	D:\Data\user data\2016\SEPT-2016\06-sep-2016\Dr.S.Kumar-SJ-499_1-B,1_01_7319.d	Acquisition Date	9/6/2016 2:30:35 PM
Method	HRLCMS-20 Sept.m	Operator	DIMPLE
Sample Name	Dr.S.Kumar-SJ-499	Instrument	micrOTOF-Q II 10330
Comment			

Acquisition Parameter

Source Type	ESI	Ion Polarity	Positive	Set Nebulizer	1.2 Bar
Focus	Active	Set Capillary	4500 V	Set Dry Heater	200 °C
Scan Begin	50 m/z	Set End Plate Offset	-500 V	Set Dry Gas	7.0 l/min
Scan End	3000 m/z	Set Collision Cell RF	130.0 Vpp	Set Divert Valve	Waste

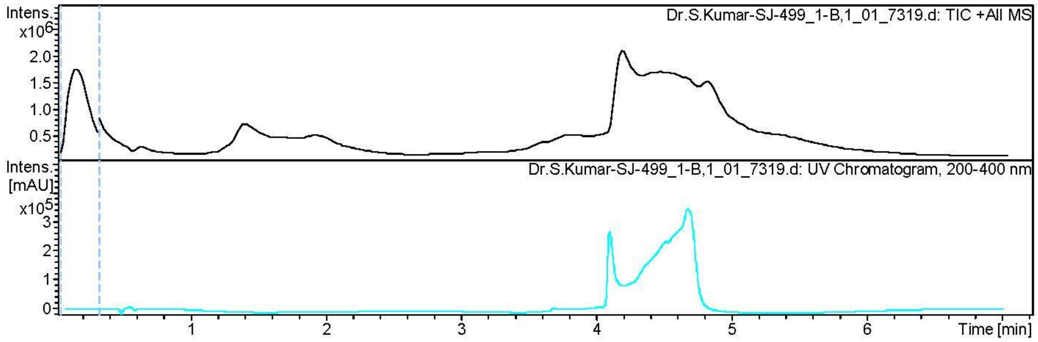

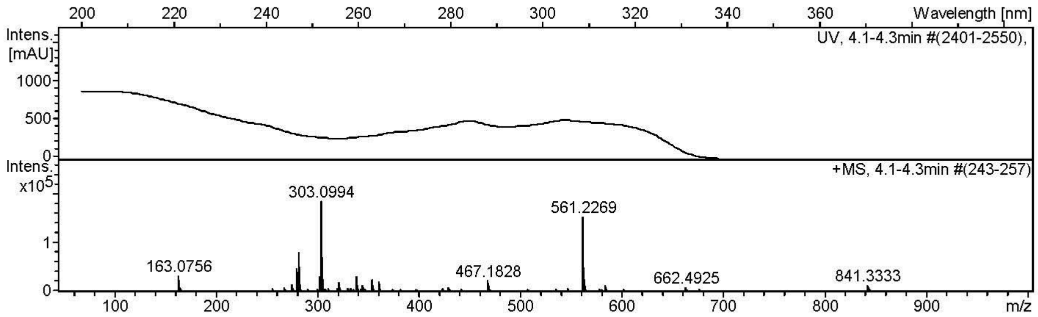

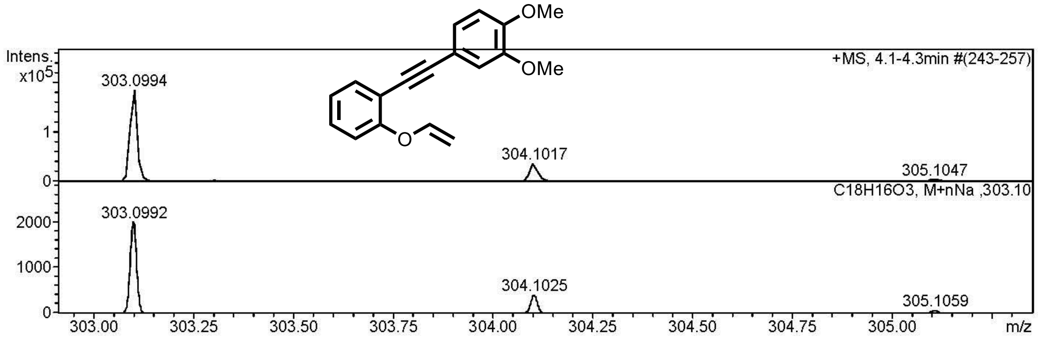

Bruker Compass DataAnalysis 4.0 printed: 9/6/2016 3:33:15 PM Page 1 of 1

^{1}H NMR of 1,3-difluoro-5-((2-(vinyloxy)phenyl)ethynyl)benzene

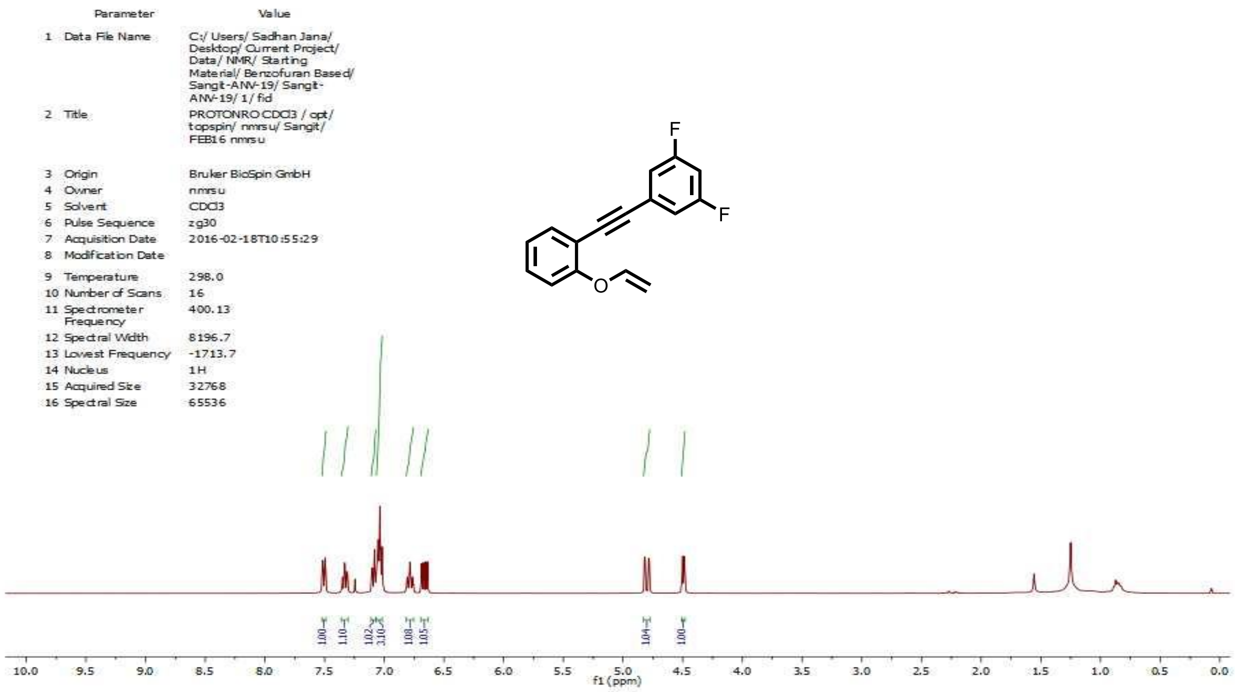

^{13}C NMR of 1,3-difluoro-5-((2-(vinyloxy)phenyl)ethynyl)benzene

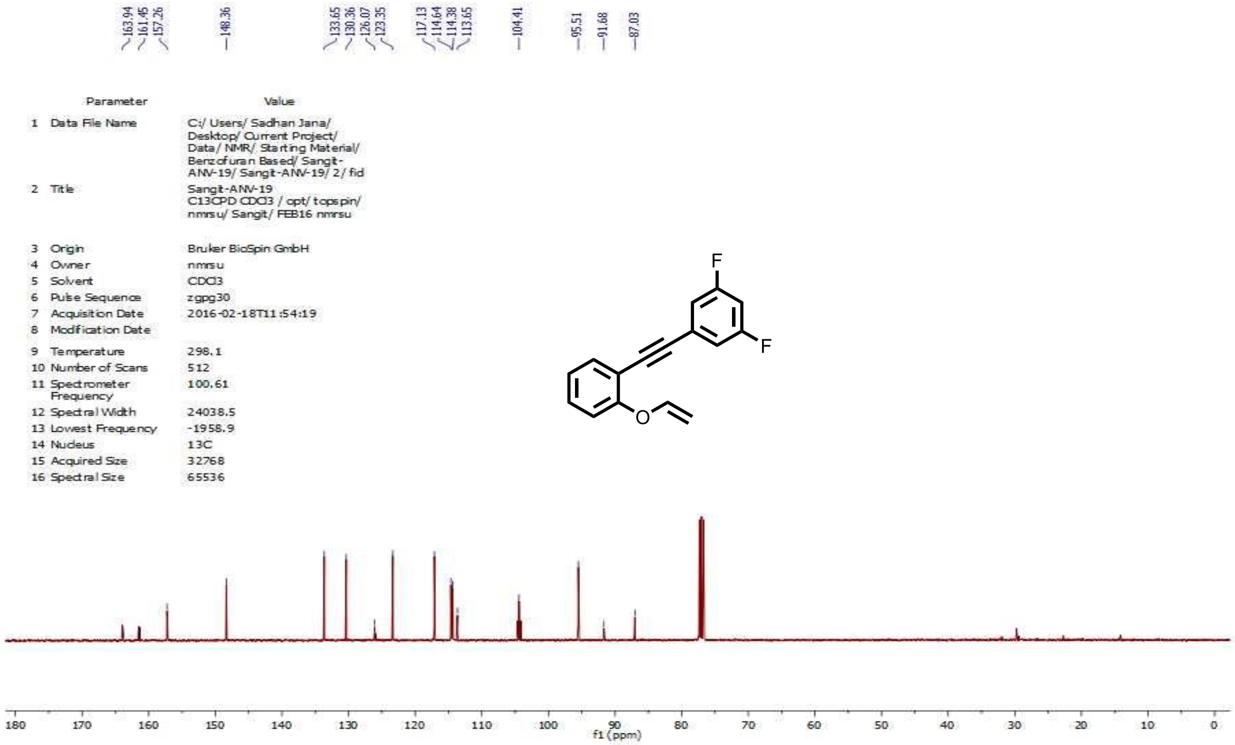

HRMS of 1,3-difluoro-5-((2-(vinyloxy)phenyl)ethynyl)benzene

Display Report

Analysis Info

		Acquisition Date	9/2/2016 12:20:54 PM
Analysis Name	D:\Data\user data\2016\SEPT-2016\02-SEPT-2016\Dr.S.Kumar-ANV-19-A.d		
Method	tune_low_APCI.m	Operator	DIMPLE
Sample Name	ANV-19-A	Instrument	micrOTOF-Q II 10330
Comment			

Acquisition Parameter

Source Type	APCI	Ion Polarity	Positive	Set Nebulizer	45.0 psi
Focus	Not active	Set Capillary	4500 V	Set Dry Heater	200 °C
Scan Begin	50 m/z	Set End Plate Offset	-500 V	Set Dry Gas	8.0 l/min
Scan End	3000 m/z	Set Collision Cell RF	130.0 Vpp	Set Divert Valve	Waste

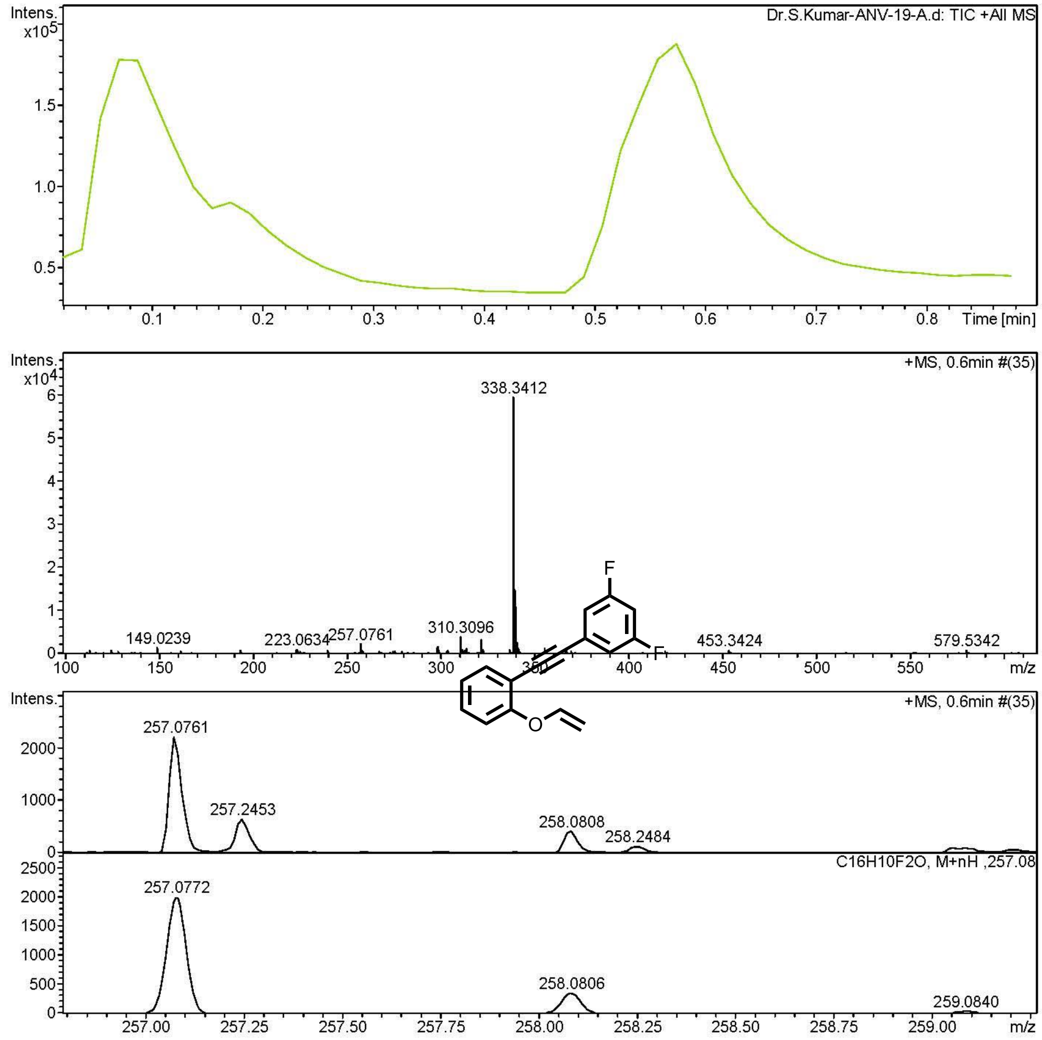

Bruker Compass DataAnalysis 4.0 printed: 9/2/2016 12:25:39 PM Page 1 of 1

^{1}H NMR of 2-((2-(vinyloxy)phenyl)ethynyl)pyridine

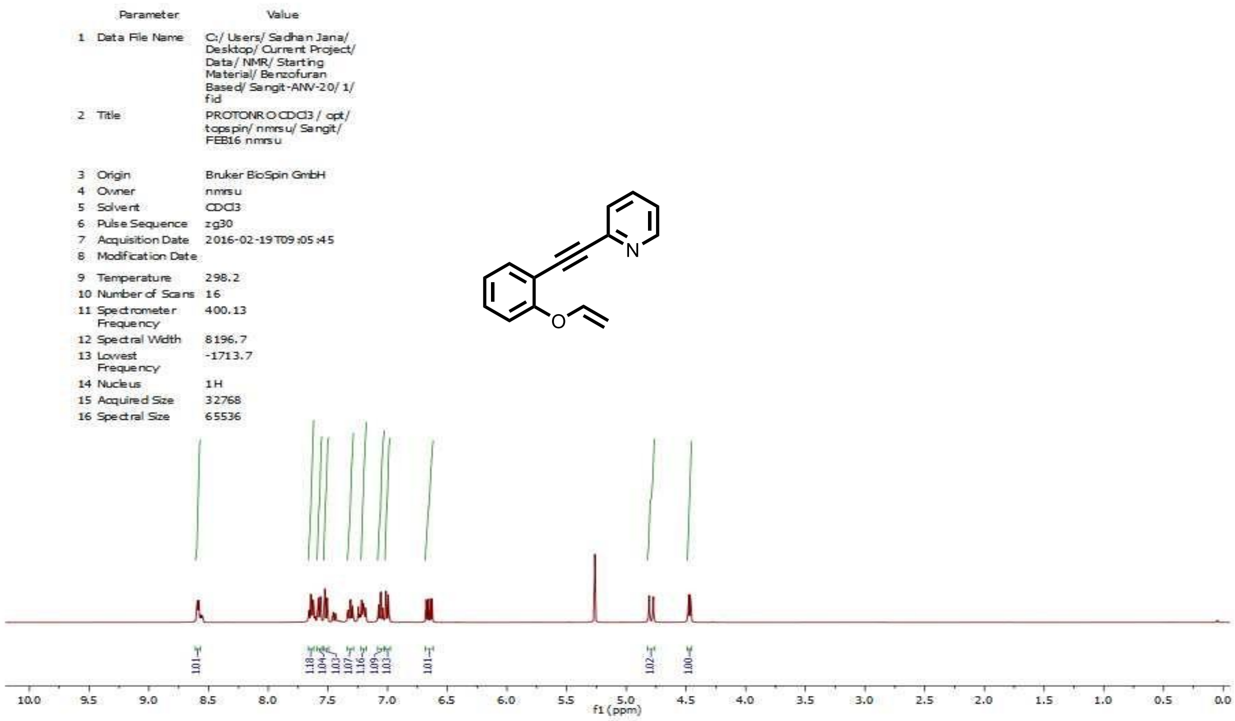

^{13}C NMR of 2-((2-(vinyloxy)phenyl)ethynyl)pyridine

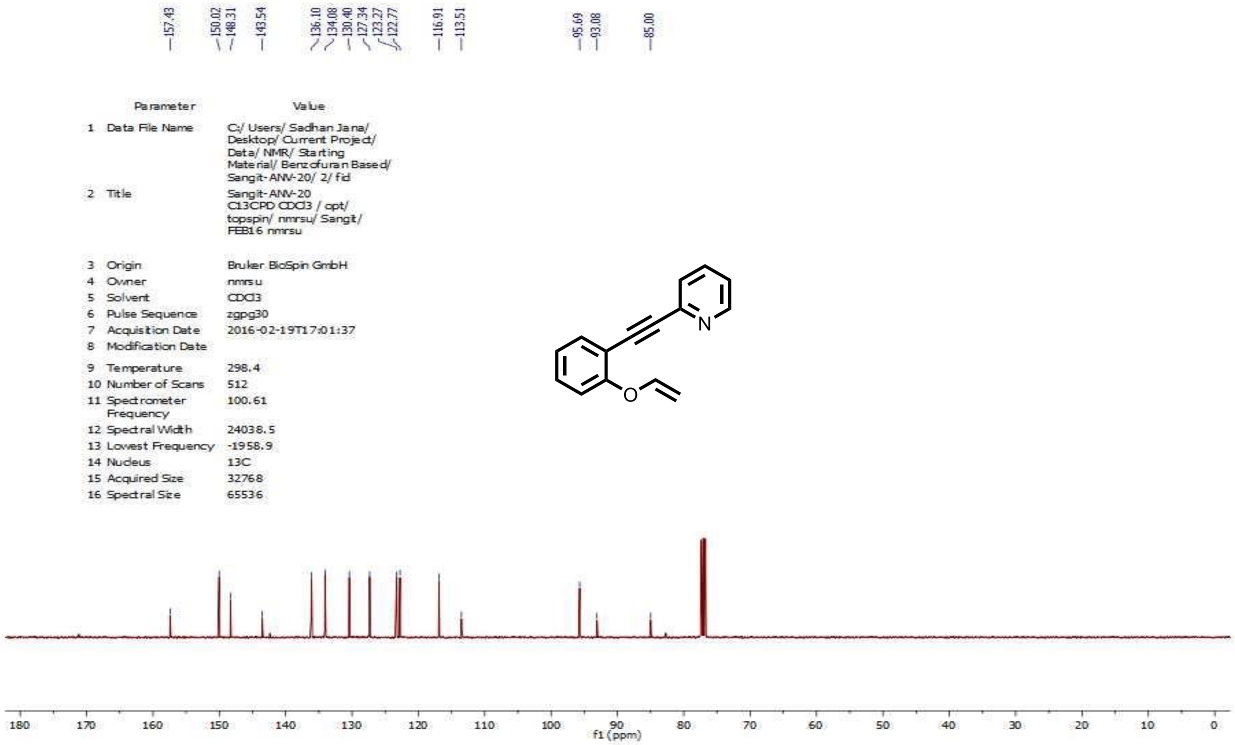

HRMS of 2-((2-(vinyloxy)phenyl)ethynyl)pyridine

Display Report

Analysis Info

		Acquisition Date	8/31/2016 1:46:57 PM
Analysis Name	D:\Data\user data\2016\August 2016\31-08-2016\Dr.S.Kumar-ANV-20_1-A,8_01_7294.d		
Method	hrlcms_pos_low_tunemix.m	Operator	DIMPLE
Sample Name	Dr.S.Kumar-ANV-20	Instrument	micrOTOF-Q II 10330
Comment			

Acquisition Parameter

Source Type	ESI	Ion Polarity	Positive	Set Nebulizer	1.0 Bar
Focus	Active	Set Capillary	4500 V	Set Dry Heater	250 °C
Scan Begin	50 m/z	Set End Plate Offset	-500 V	Set Dry Gas	7.0 l/min
Scan End	3000 m/z	Set Collision Cell RF	130.0 Vpp	Set Divert Valve	Waste

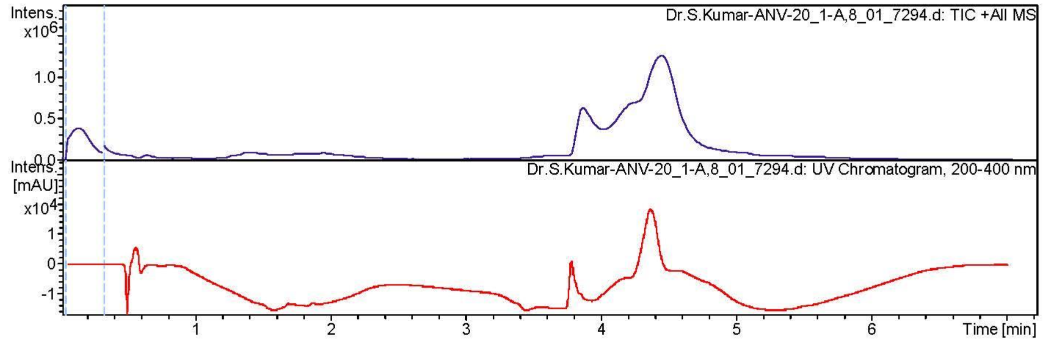

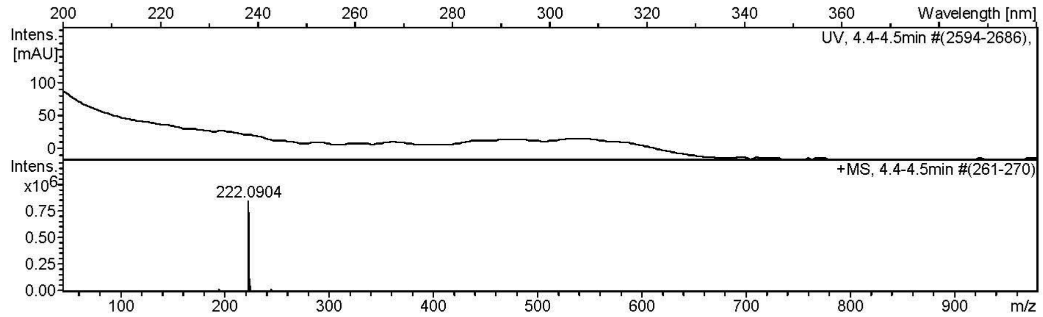

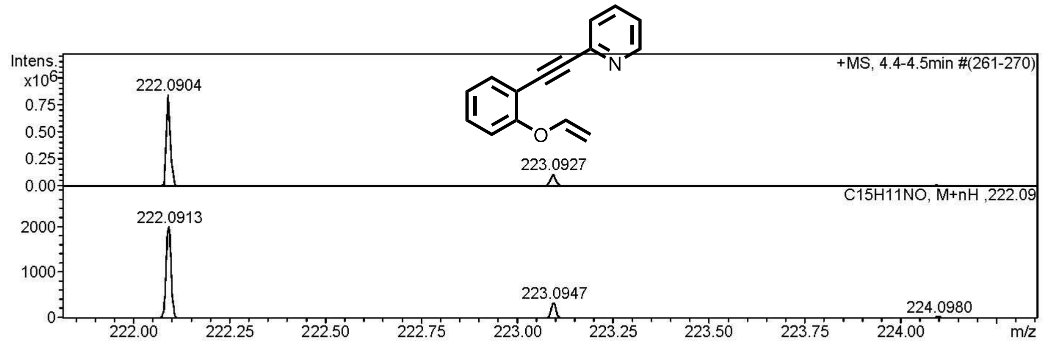

Bruker Compass DataAnalysis 4.0 printed: 8/31/2016 5:12:04 PM Page 1 of 1

^{1}H NMR of 3-((2-(vinyloxy)phenyl)ethynyl)thiophene

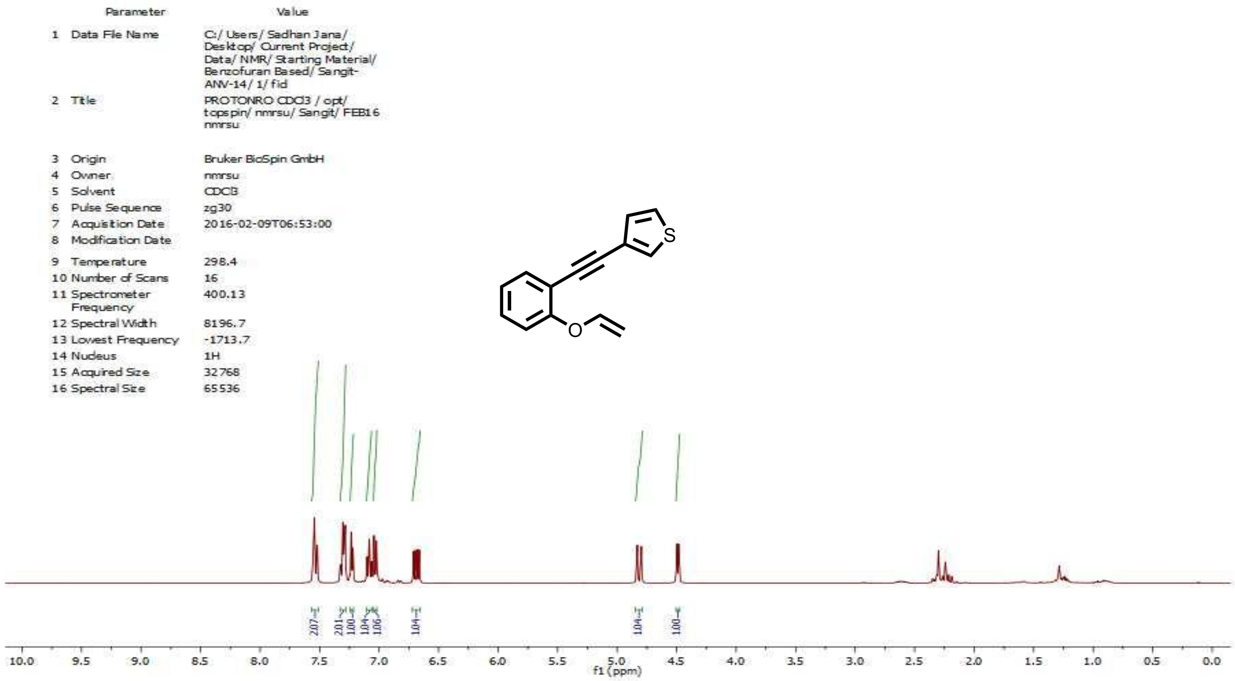

^{13}C NMR of 3-((2-(vinyloxy)phenyl)ethynyl)thiophene

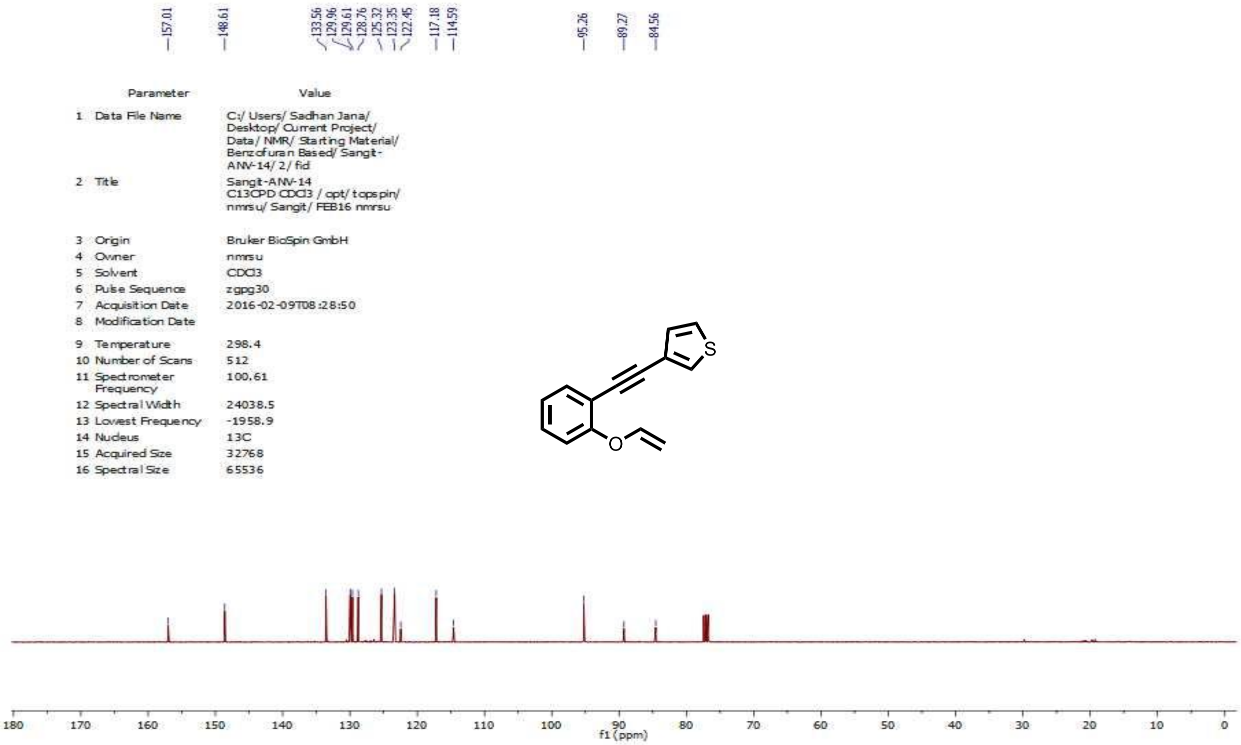

^{1}H of 2-((2-(vinyloxy)phenyl)ethynyl)naphthalene

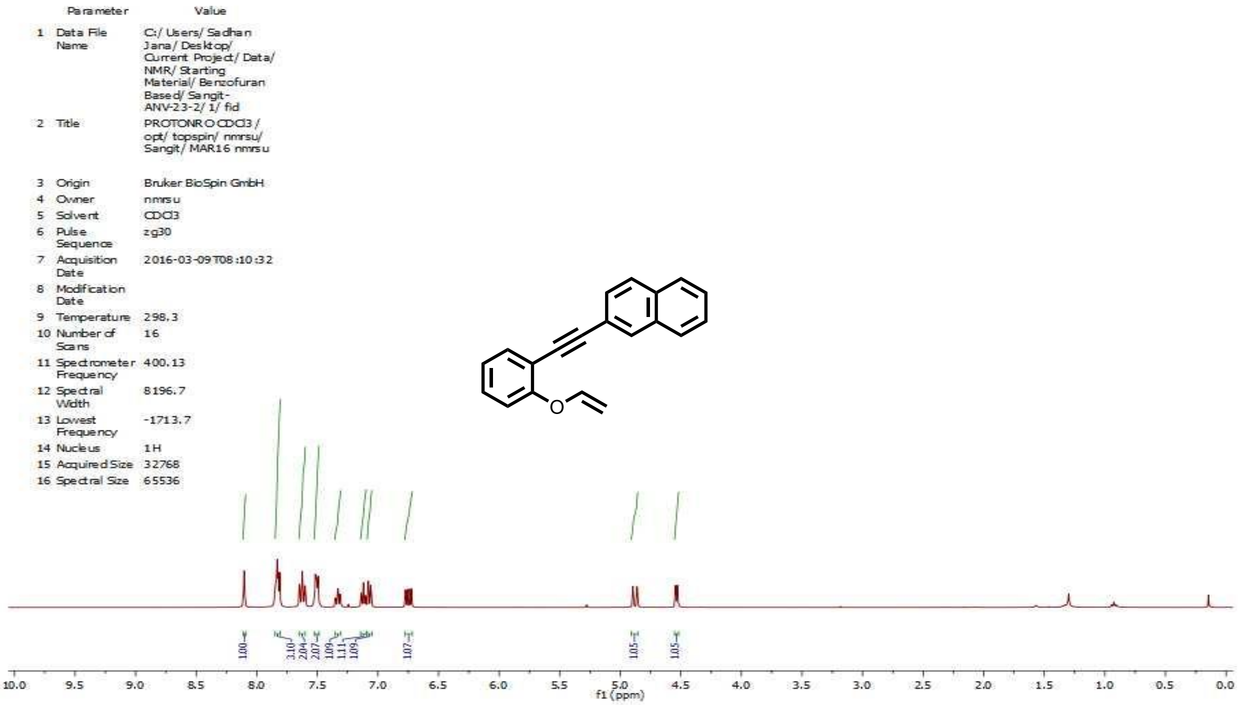

^{13}C of 2-((2-(vinyloxy)phenyl)ethynyl)naphthalene

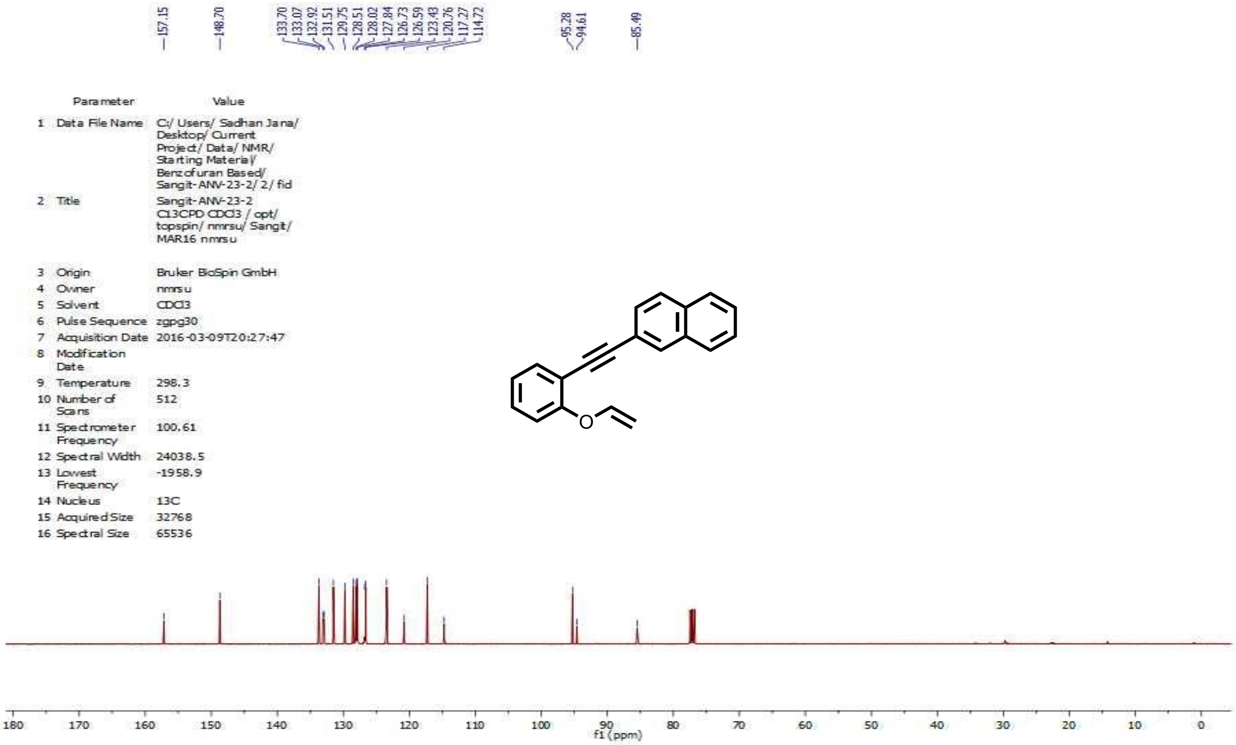

HRMS of 2-((2-(vinyloxy)phenyl)ethynyl)naphthalene

Display Report

Analysis Info

		Acquisition Date	9/2/2016 12:11:09 PM
Analysis Name	D:\Data\user data\2016\SEPT-2016\02-SEPT-2016\Dr.S.Kumar-ANV-23A.d		
Method	tune_low_APCI.m	Operator	DIMPLE
Sample Name	ANV-23	Instrument	micrOTOF-Q II 10330
Comment			

Acquisition Parameter

Source Type	APCI	Ion Polarity	Positive	Set Nebulizer	45.0 psi
Focus	Not active	Set Capillary	4500 V	Set Dry Heater	200 °C
Scan Begin	50 m/z	Set End Plate Offset	-500 V	Set Dry Gas	8.0 l/min
Scan End	3000 m/z	Set Collision Cell RF	130.0 Vpp	Set Divert Valve	Waste

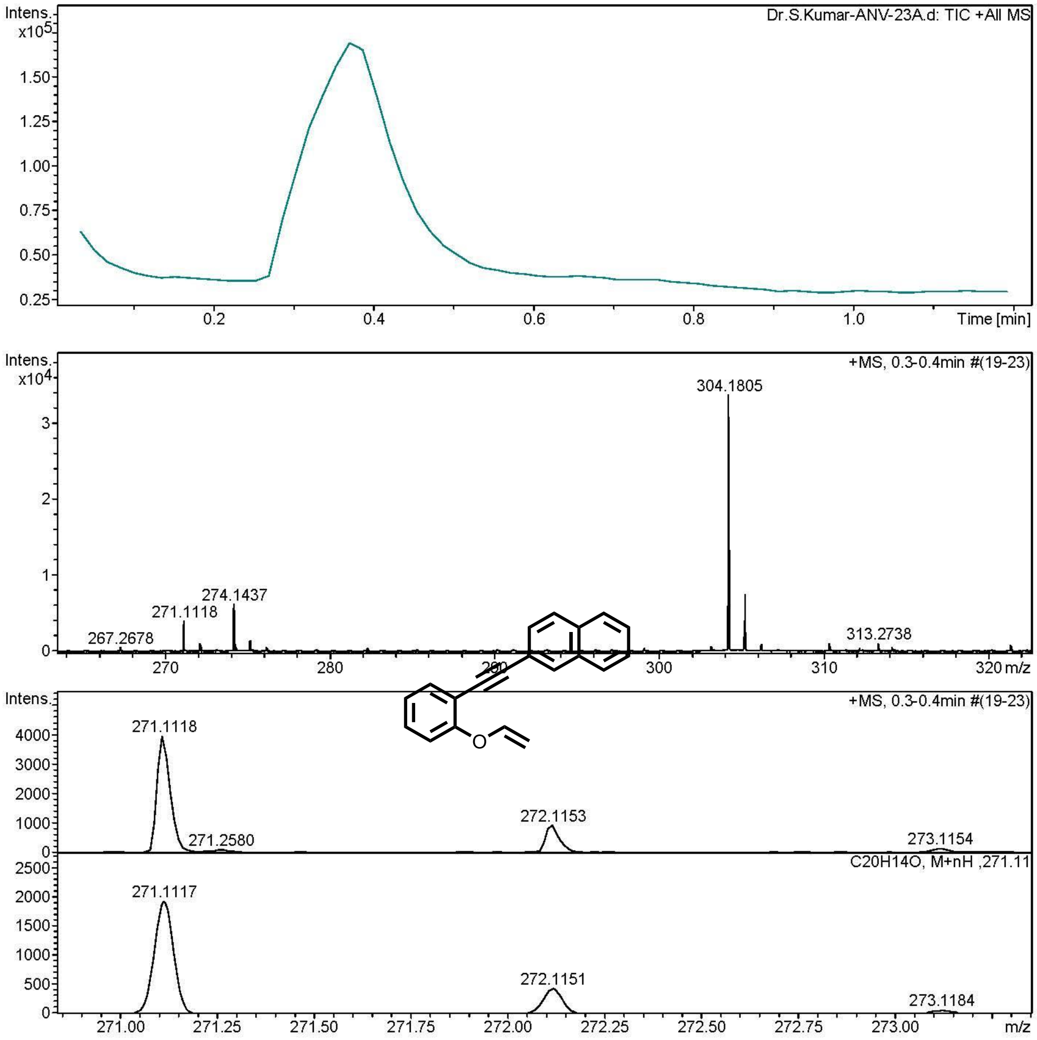

Bruker Compass DataAnalysis 4.0   printed: 9/2/2016 12:15:31 PM   Page 1 of 1

^{1}H NMR of 1-(cyclopentylethynyl)-2-(vinyloxy)benzene

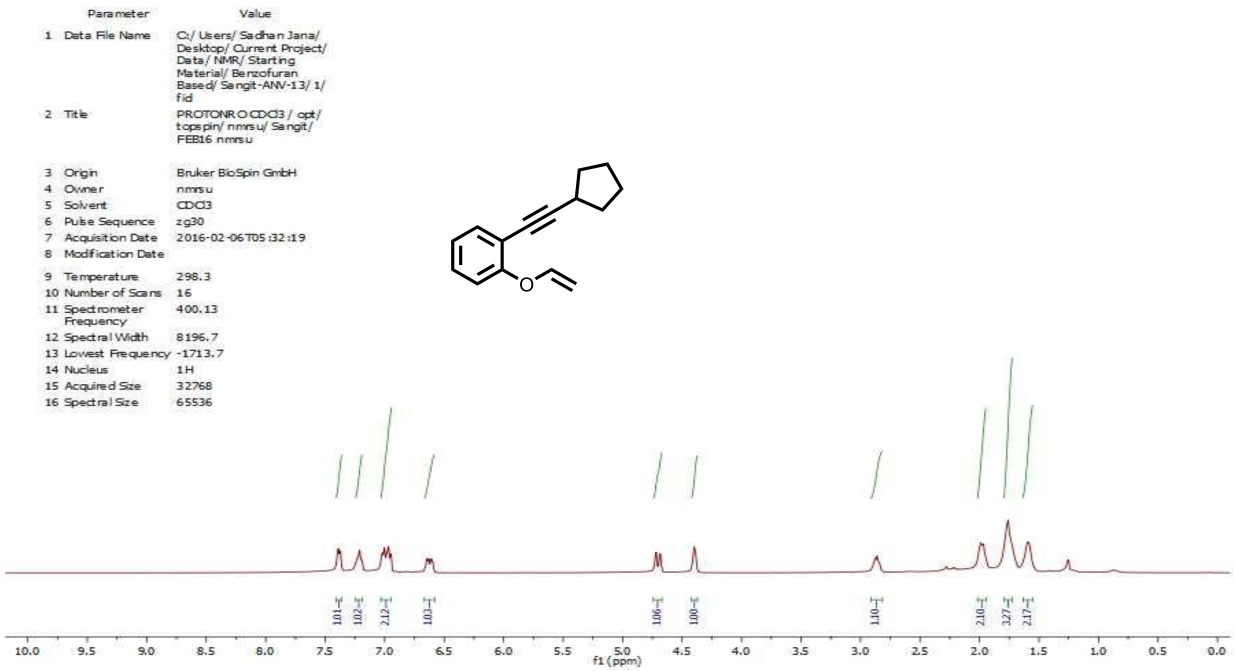

^{13}C NMR of 1-(cyclopentylethynyl)-2-(vinyloxy)benzene

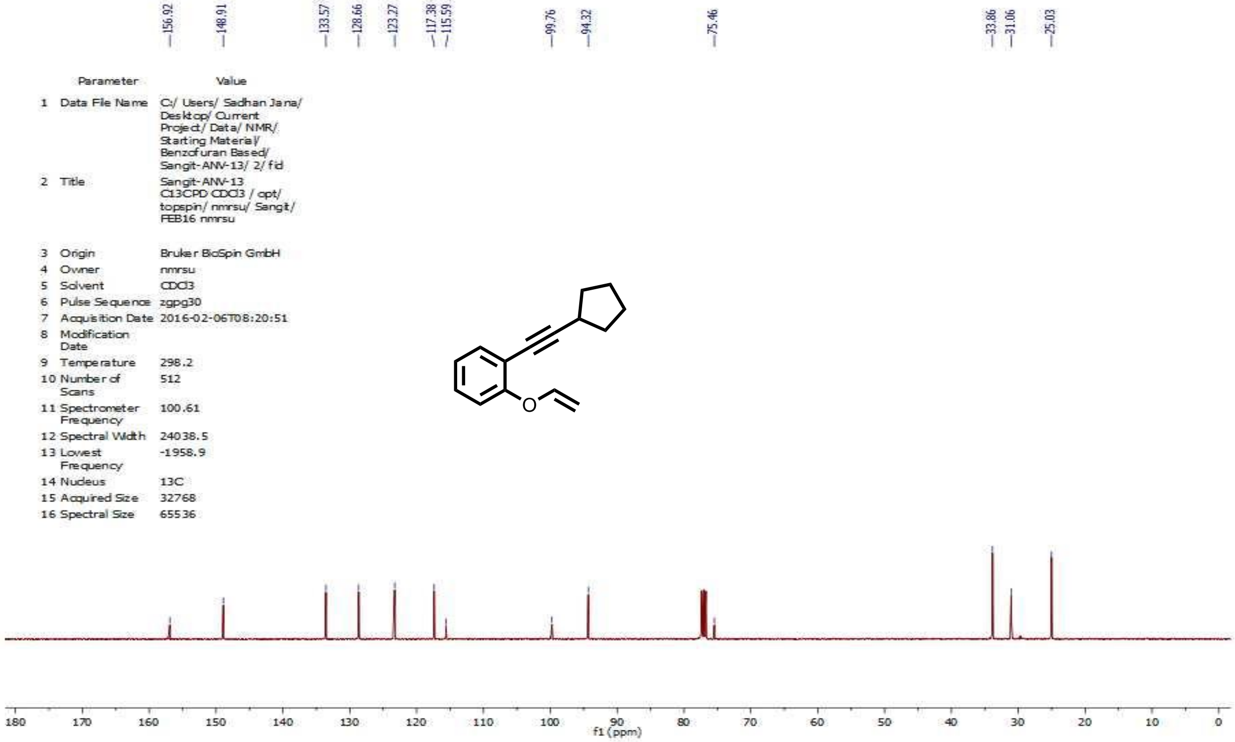

HRMS of 1-(cyclopentylethynyl)-2-(vinyloxy)benzene

Display Report

Analysis Info

		Acquisition Date	8/30/2016 2:07:13 PM
Analysis Name	D:\Data\user data\2016\August 2016\30-08-2016\Dr.S.Kumar-ANV-13_1-B,4_01_7273.d		
Method	hrlcms_pos_low_tunemix.m	Operator	DIMPLE
Sample Name	Dr.S.Kumar-ANV-13	Instrument	micrOTOF-Q II 10330
Comment			

Acquisition Parameter

Source Type	ESI	Ion Polarity	Positive	Set Nebulizer	1.0 Bar
Focus	Active	Set Capillary	4500 V	Set Dry Heater	250 °C
Scan Begin	50 m/z	Set End Plate Offset	-500 V	Set Dry Gas	7.0 l/min
Scan End	3000 m/z	Set Collision Cell RF	130.0 Vpp	Set Divert Valve	Waste

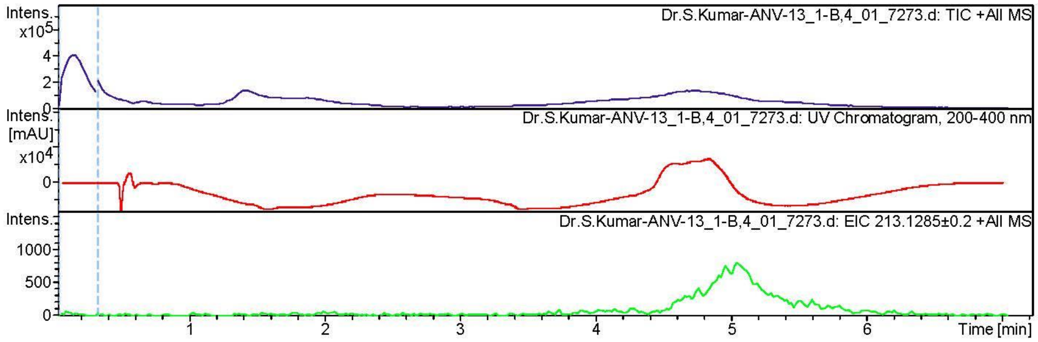

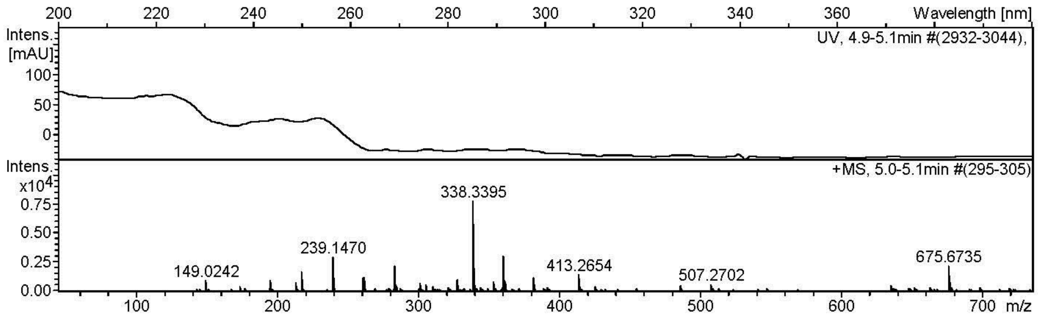

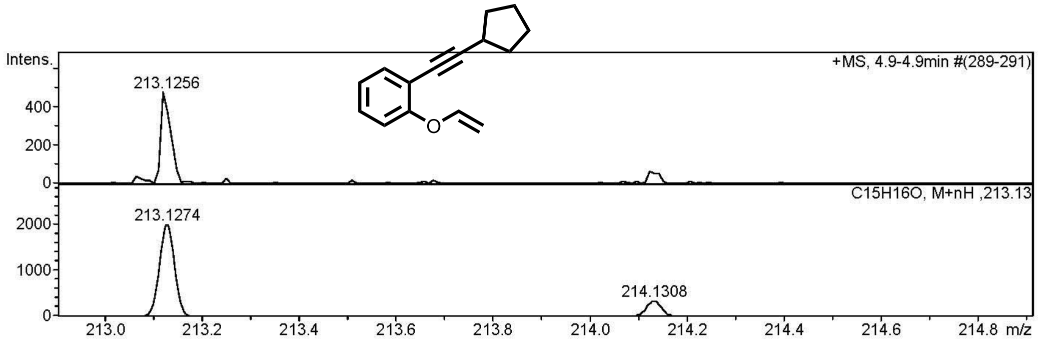

^{1}H NMR of 1-(hept-1-yn-1-yl)-2-(vinyloxy)benzene

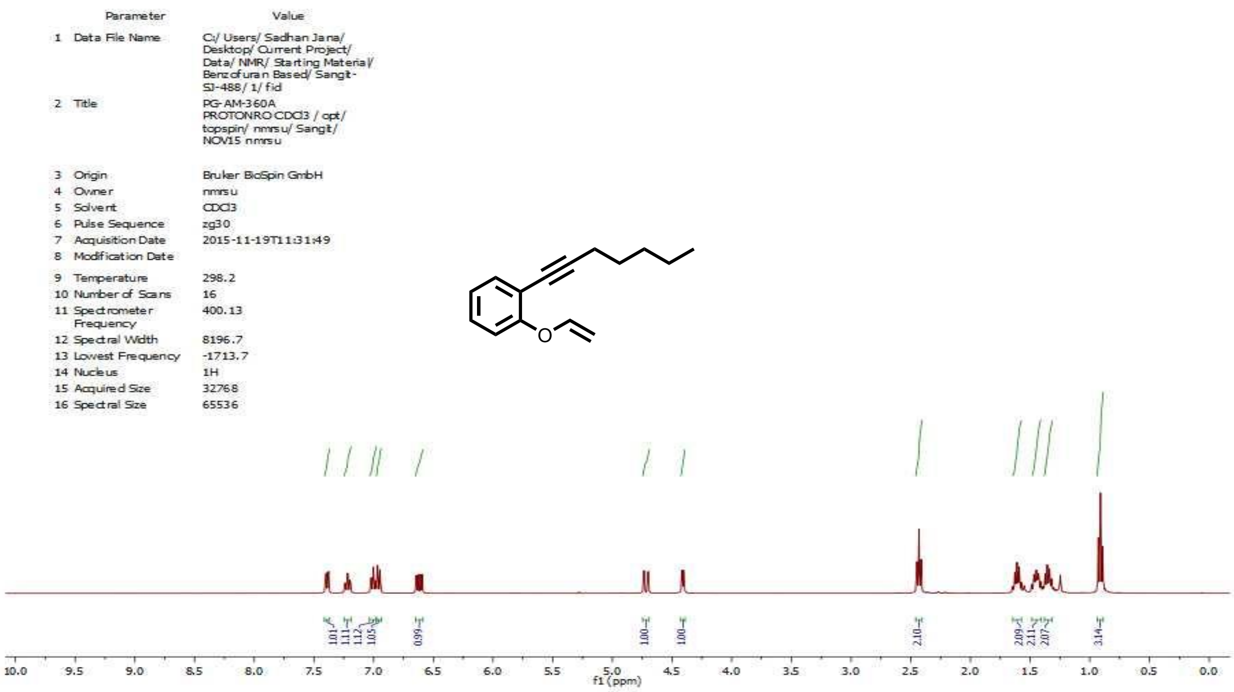

	Parameter	Value
1	Data File Name	C:/ Users/ Sadhan Jana/ Desktop/ Current Project/ Data/ NMR/ Starting Material/ Benzofuran Based/ Sangit-SJ-488/ 1/ fid
2	Title	PG-AM-360A PROTONRO CDCl3 / opt/ topspin/ nmrsu/ Sangit/ NOV15 nmrsu
3	Origin	Bruker BioSpin GmbH
4	Owner	nmrsu
5	Solvent	CDCl3
6	Pulse Sequence	zg30
7	Acquisition Date	2015-11-19T11:31:49
8	Modification Date	
9	Temperature	298.2
10	Number of Scans	16
11	Spectrometer Frequency	400.13
12	Spectral Width	8196.7
13	Lowest Frequency	-1713.7
14	Nucleus	1H
15	Acquired Size	32768
16	Spectral Size	65536

^{13}C NMR of 1-(hept-1-yn-1-yl)-2-(vinyloxy)benzene

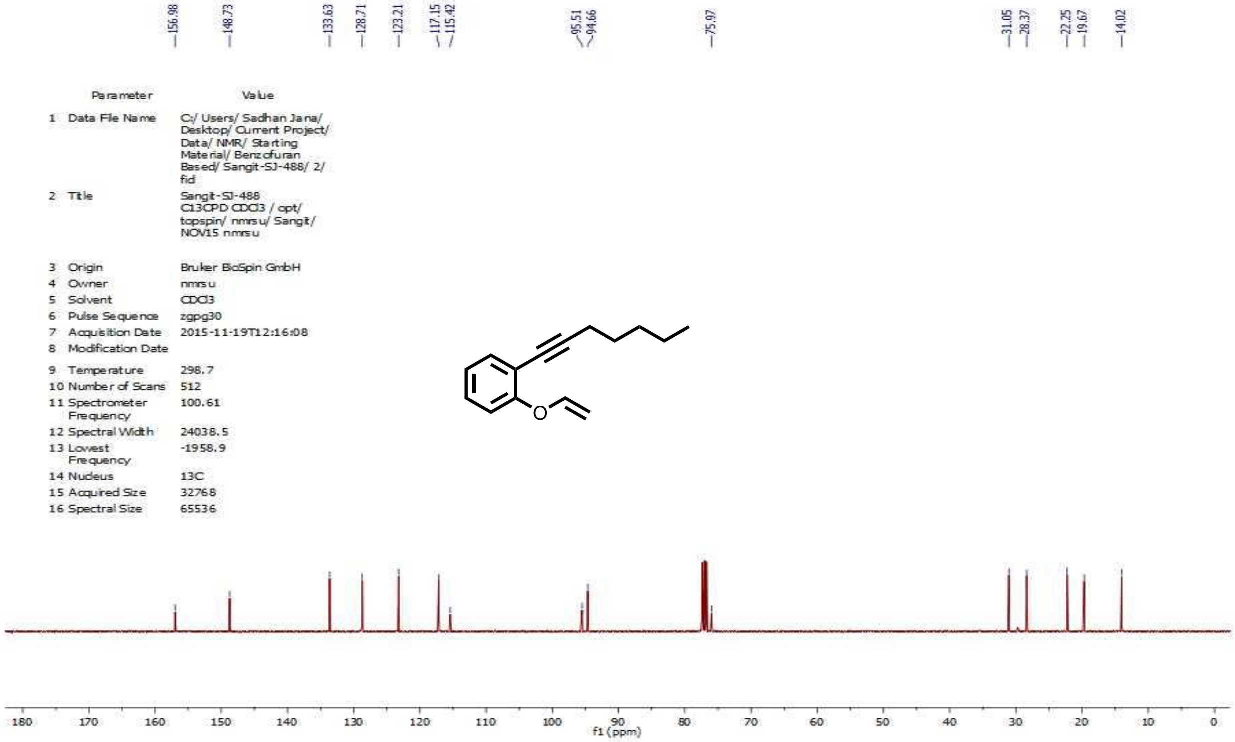

	Parameter	Value
1	Data File Name	C:/ Users/ Sadhan Jana/ Desktop/ Current Project/ Data/ NMR/ Starting Material/ Benzofuran Based/ Sangit-SJ-488/ 2/ fid
2	Title	Sangit-SJ-488 C13CPD CDCl3 / opt/ topspin/ nmrsu/ Sangit/ NOV15 nmrsu
3	Origin	Bruker BioSpin GmbH
4	Owner	nmrsu
5	Solvent	CDCl3
6	Pulse Sequence	zgpg30
7	Acquisition Date	2015-11-19T12:16:08
8	Modification Date	
9	Temperature	298.7
10	Number of Scans	512
11	Spectrometer Frequency	100.61
12	Spectral Width	24038.5
13	Lowest Frequency	-1958.9
14	Nucleus	13C
15	Acquired Size	32768
16	Spectral Size	65536

^{1}H NMR of 1-(phenylethynyl)-2-(vinyloxy)naphthalene

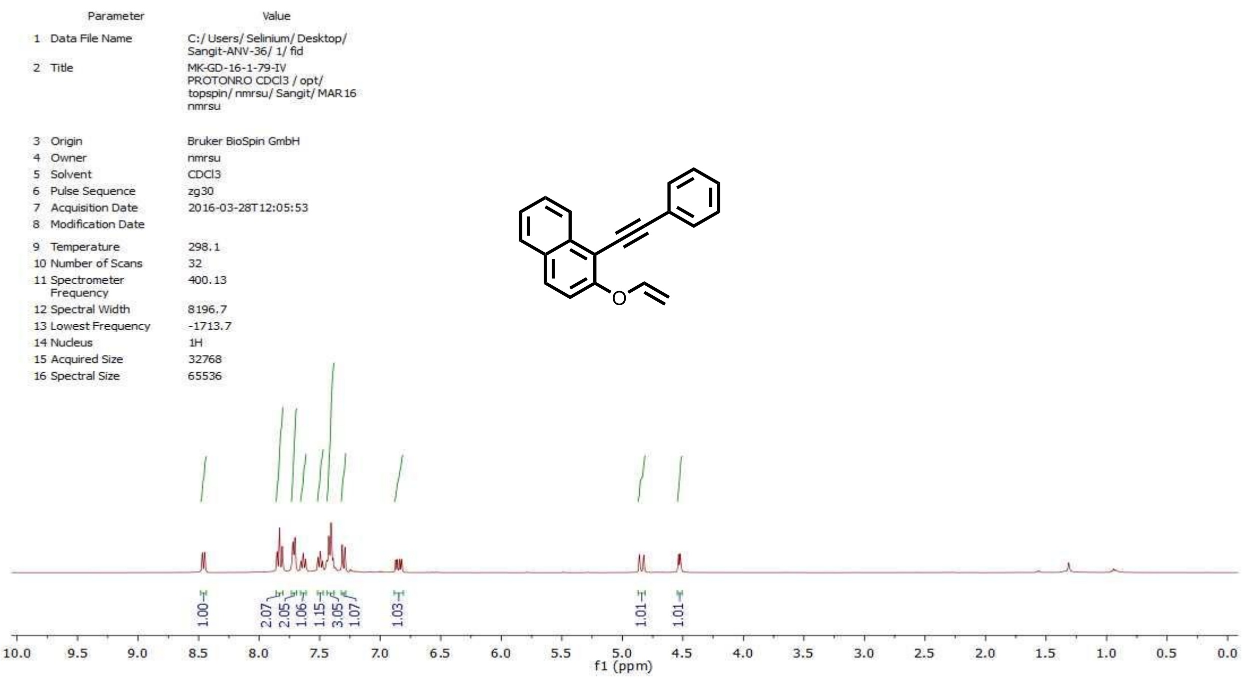

^{13}C NMR of 1-(phenylethynyl)-2-(vinyloxy)naphthalene

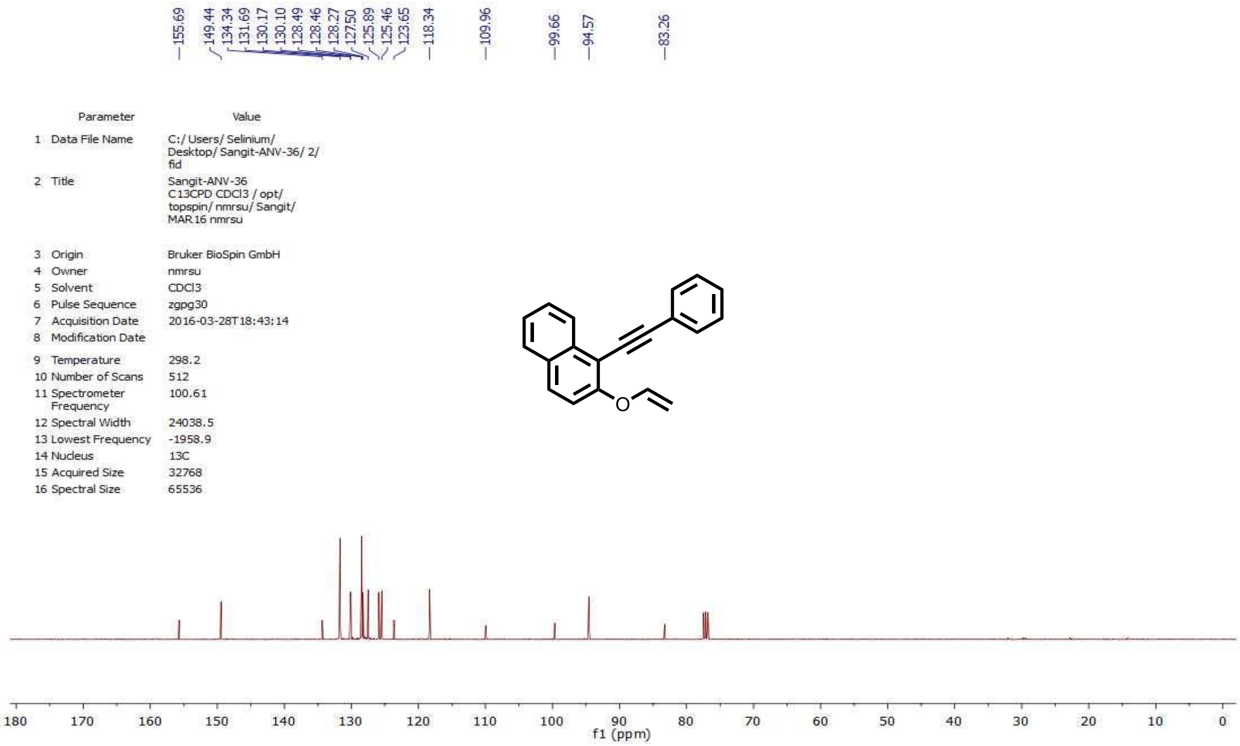

^{1}H NMR of 4-methyl-2-(phenylethynyl)-1-(vinyloxy)benzene

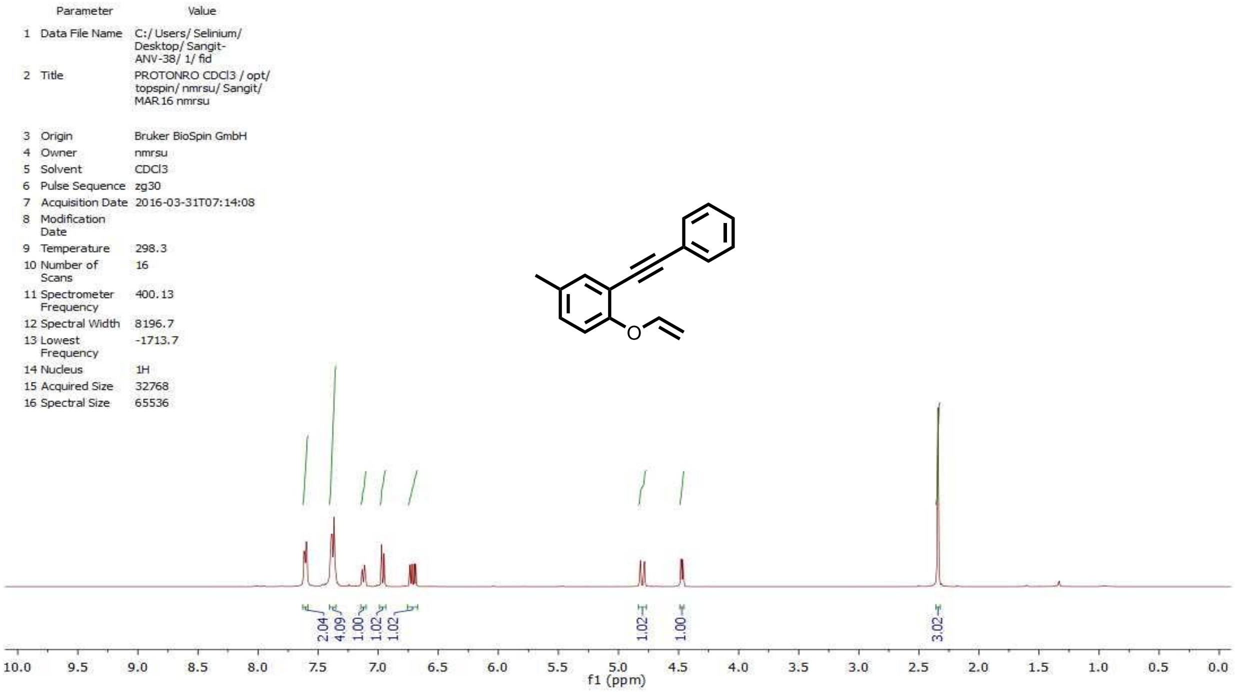

^{13}C NMR of 4-methyl-2-(phenylethynyl)-1-(vinyloxy)benzene

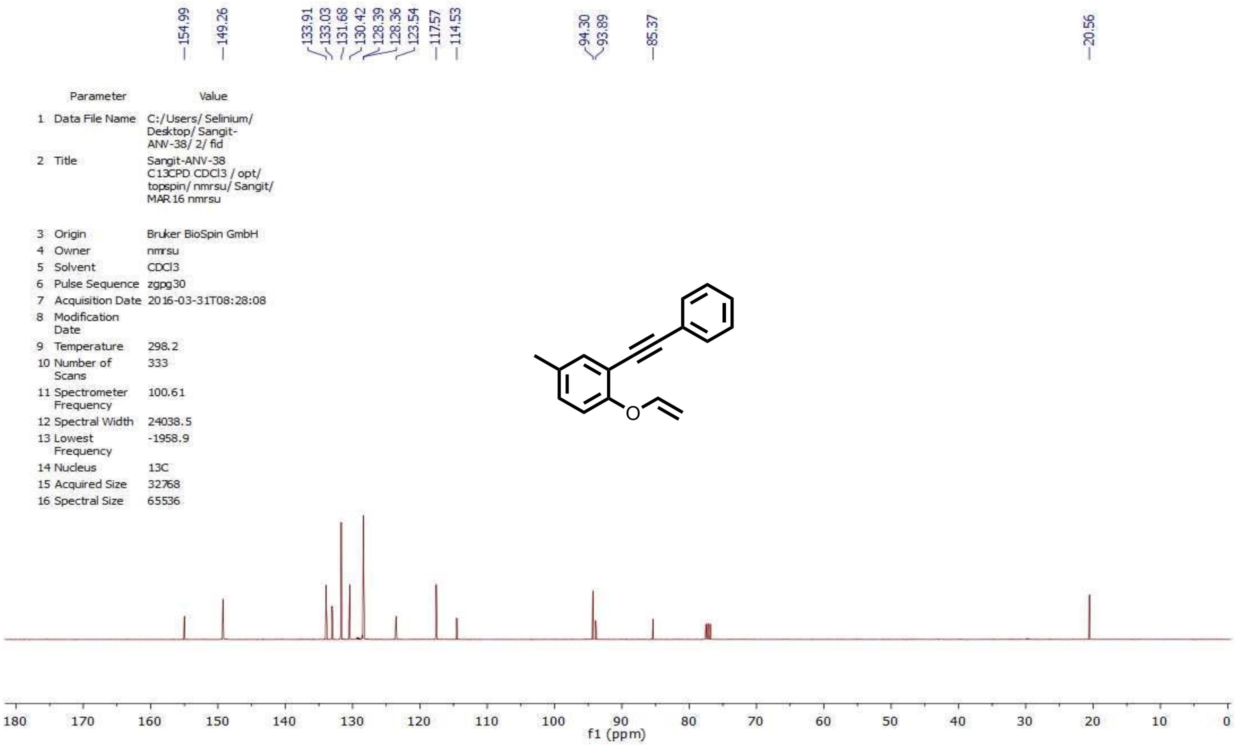

^{1}H NMR of (2-Bromoethyl)(2-bromophenyl)sulfane

	Parameter	Value
1	Data File Name	C:/ Users/ Selenium-A/ Desktop/ Sangit-SJ-458-P1(500MHz)/ 1/ fid
2	Title	Sangit-SJ-458-P1(500MHz). 1. fid
3	Origin	Bruker BioSpin GmbH
4	Owner	nmrsu
5	Solvent	CDCl3
6	Temperature	298.0
7	Pulse Sequence	zg30
8	Number of Scans	16
9	Acquisition Date	2016-11-25T12:56:36
10	Modification Date	2016-11-25T12:56:36
11	Spectrometer Frequency	500.13
12	Spectral Width	10000.0
13	Lowest Frequency	-1911.5
14	Nucleus	1H
15	Acquired Size	32768
16	Spectral Size	65536

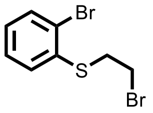

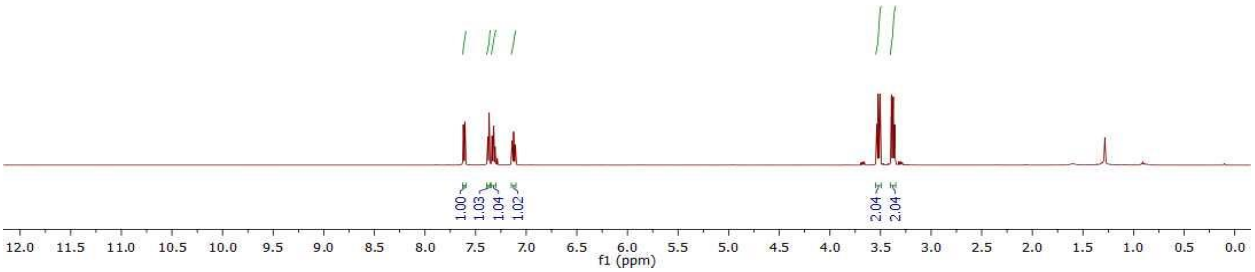

^{13}C NMR of (2-Bromoethyl)(2-bromophenyl)sulfane

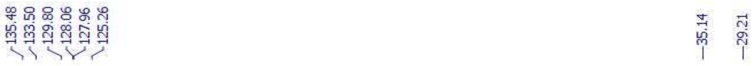

	Parameter	Value
1	Data File Name	C:/ Users/ Selenium-A/ Desktop/ Sangit-SJ-458_P1(500MHz)/ 1/ fid
2	Title	Sangit-SJ-458_P1(500MHz). 1. fid
3	Origin	Bruker BioSpin GmbH
4	Owner	nmrsu
5	Solvent	CDCl3
6	Temperature	298.0
7	Pulse Sequence	zgpg30
8	Number of Scans	512
9	Acquisition Date	2015-10-12T14:32:24
10	Modification Date	2015-10-12T14:32:24
11	Spectrometer Frequency	125.77
12	Spectral Width	29761.9
13	Lowest Frequency	-2306.2
14	Nucleus	13C
15	Acquired Size	32768
16	Spectral Size	65536

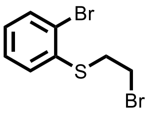

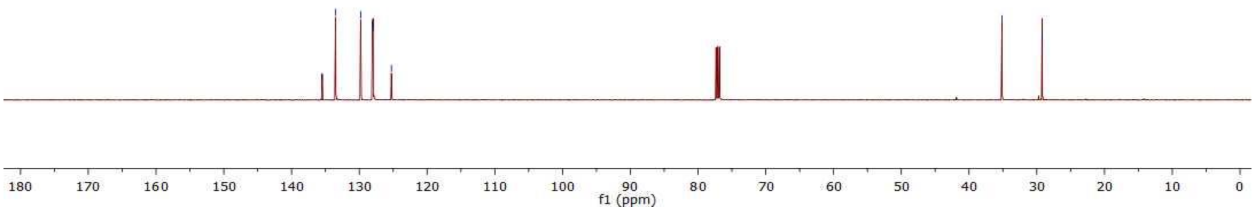

^{1}H NMR of 1,2-Bis ((2-bromophenyl)thio)ethane

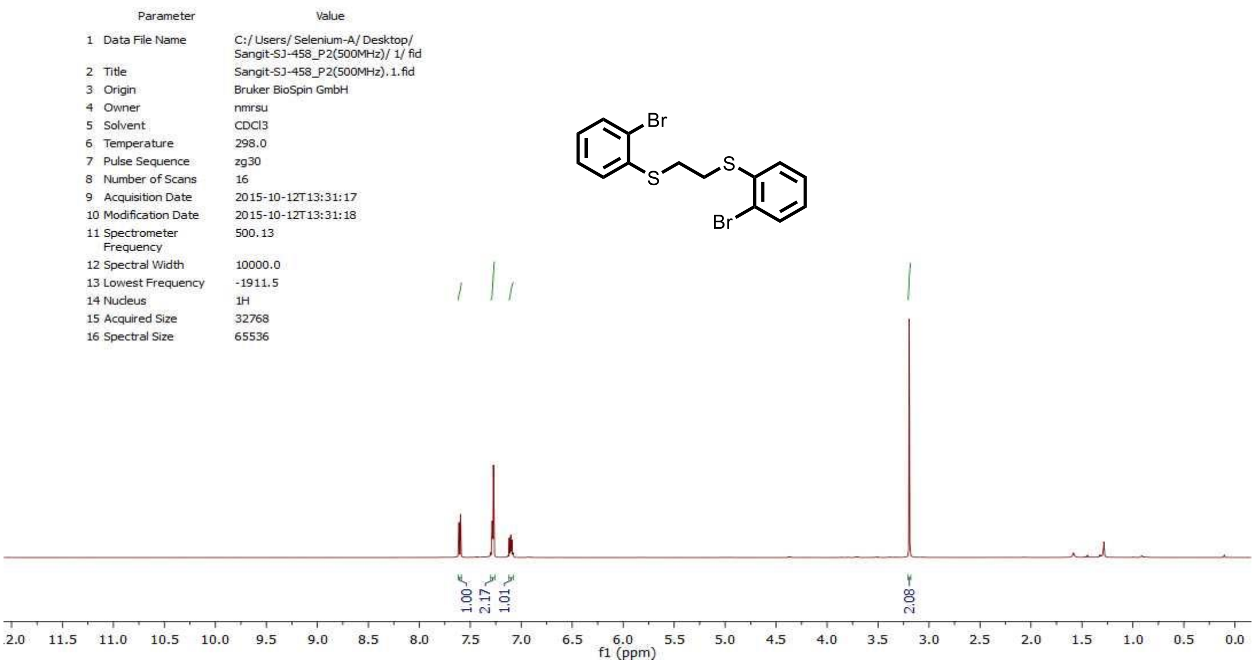

^{13}C NMR of 1,2-Bis ((2-bromophenyl)thio)ethane

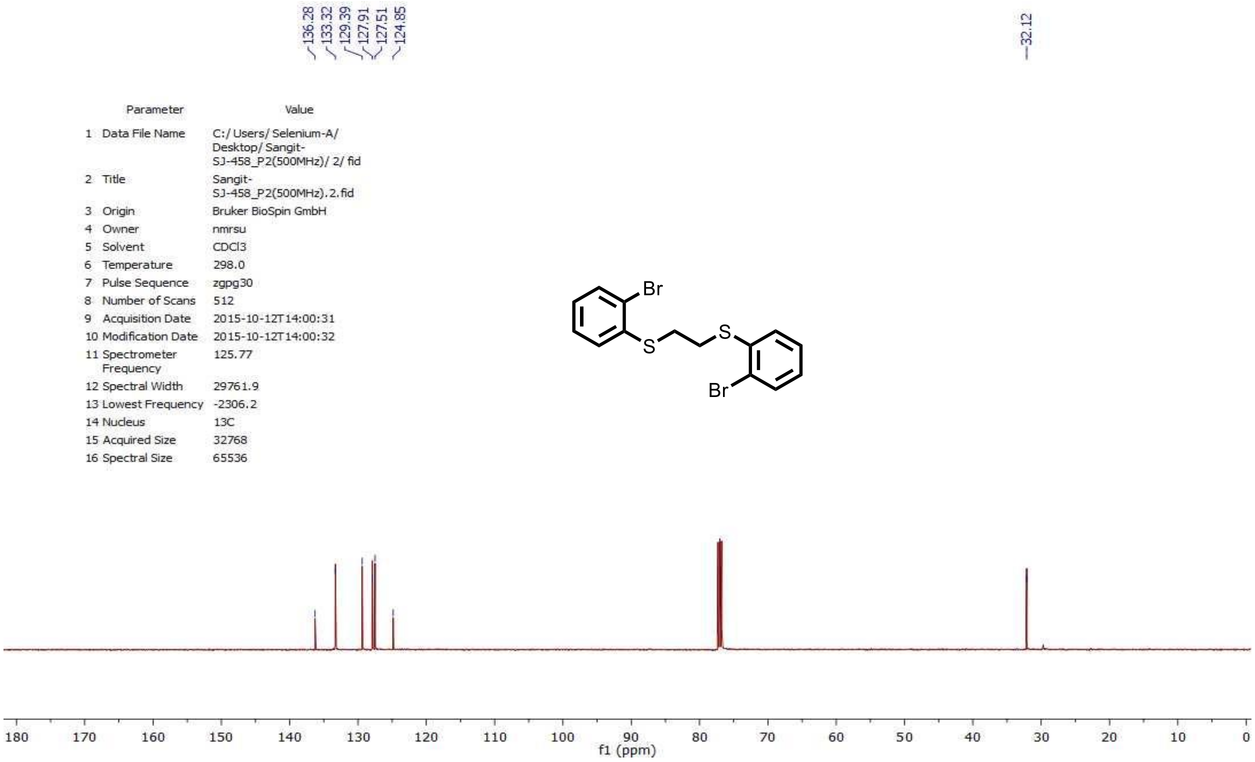

^{1}H NMR of (2-Bromophenyl)(vinyl)sulfane

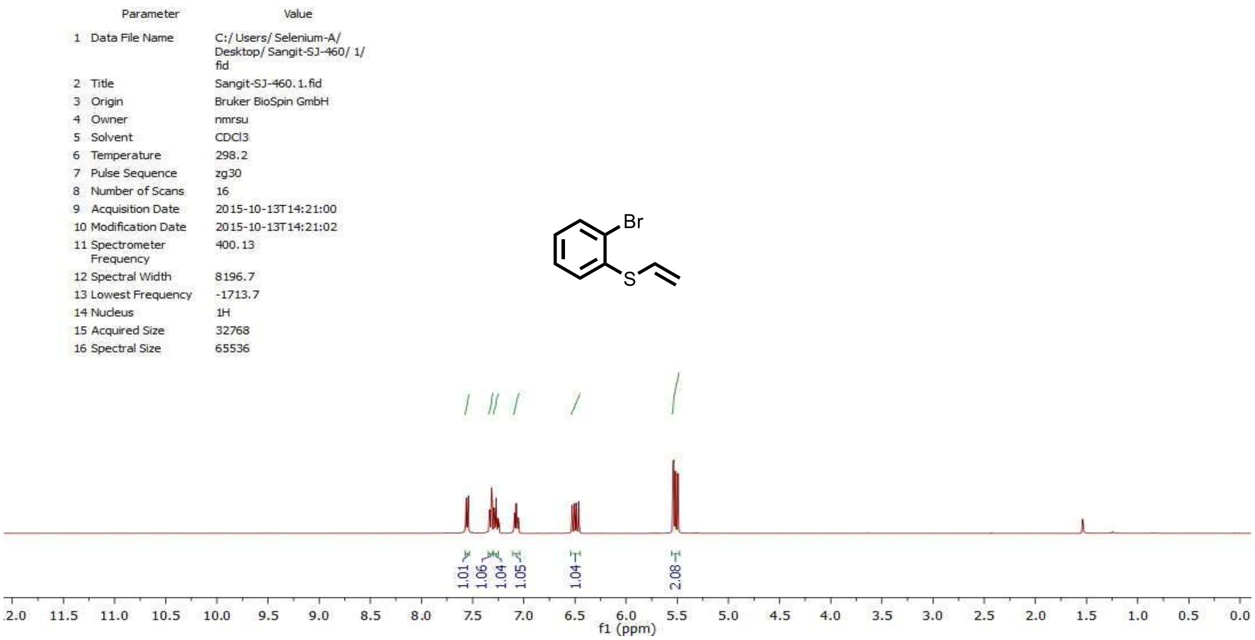

^{13}C NMR of (2-Bromophenyl)(vinyl)sulfane

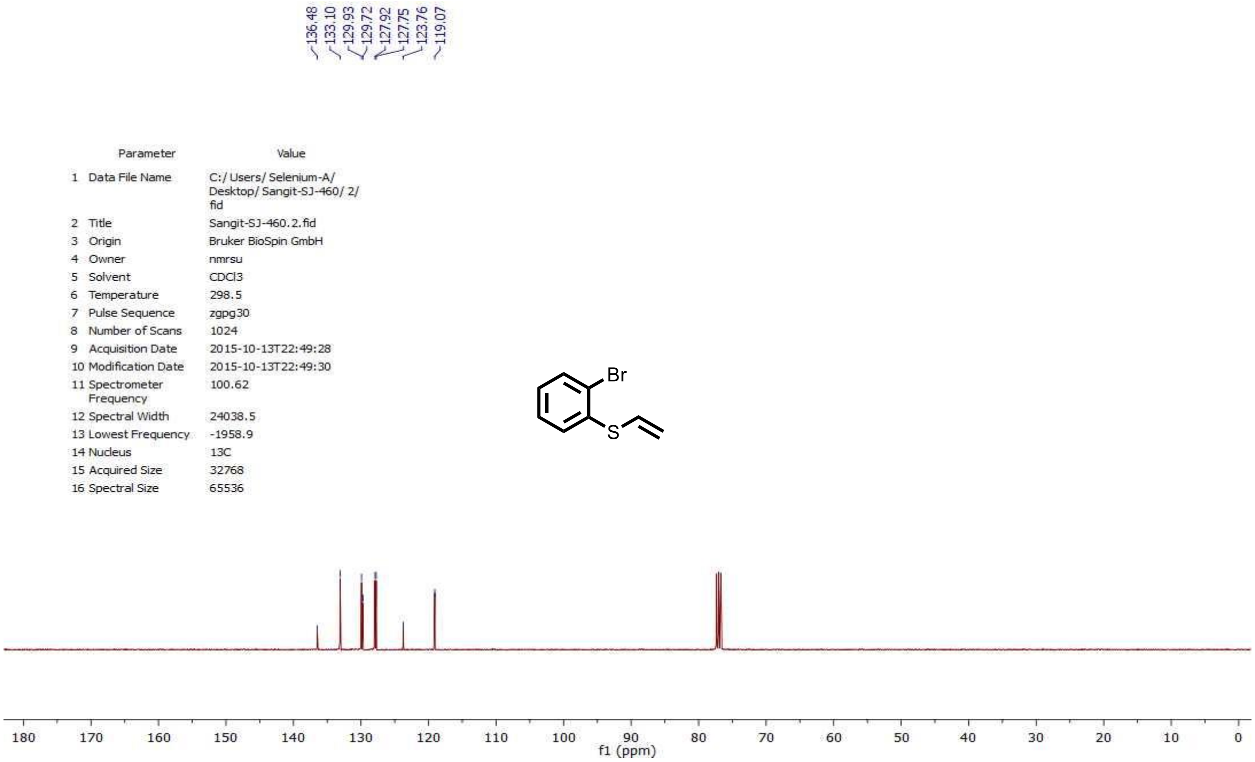

HRMS of (2-Bromophenyl)(vinyl)sulfane

Display Report

Analysis Info

Analysis Name	D:\Data\user data\2017\JAN 2017\19 jan\Dr.S.Kumar-SJ-460.d	Acquisition Date	1/19/2017 3:48:25 PM
Method	tune_low_APCI.m	Operator	RUCHI SHRIVASTAVA
Sample Name	SJ-460	Instrument	micrOTOF-Q II 10330
Comment			

Acquisition Parameter

Source Type	APCI	Ion Polarity	Positive	Set Nebulizer	3.1 Bar
Focus	Not active	Set Capillary	4500 V	Set Dry Heater	200 °C
Scan Begin	50 m/z	Set End Plate Offset	-500 V	Set Dry Gas	8.0 l/min
Scan End	3000 m/z	Set Collision Cell RF	130.0 Vpp	Set Divert Valve	Waste

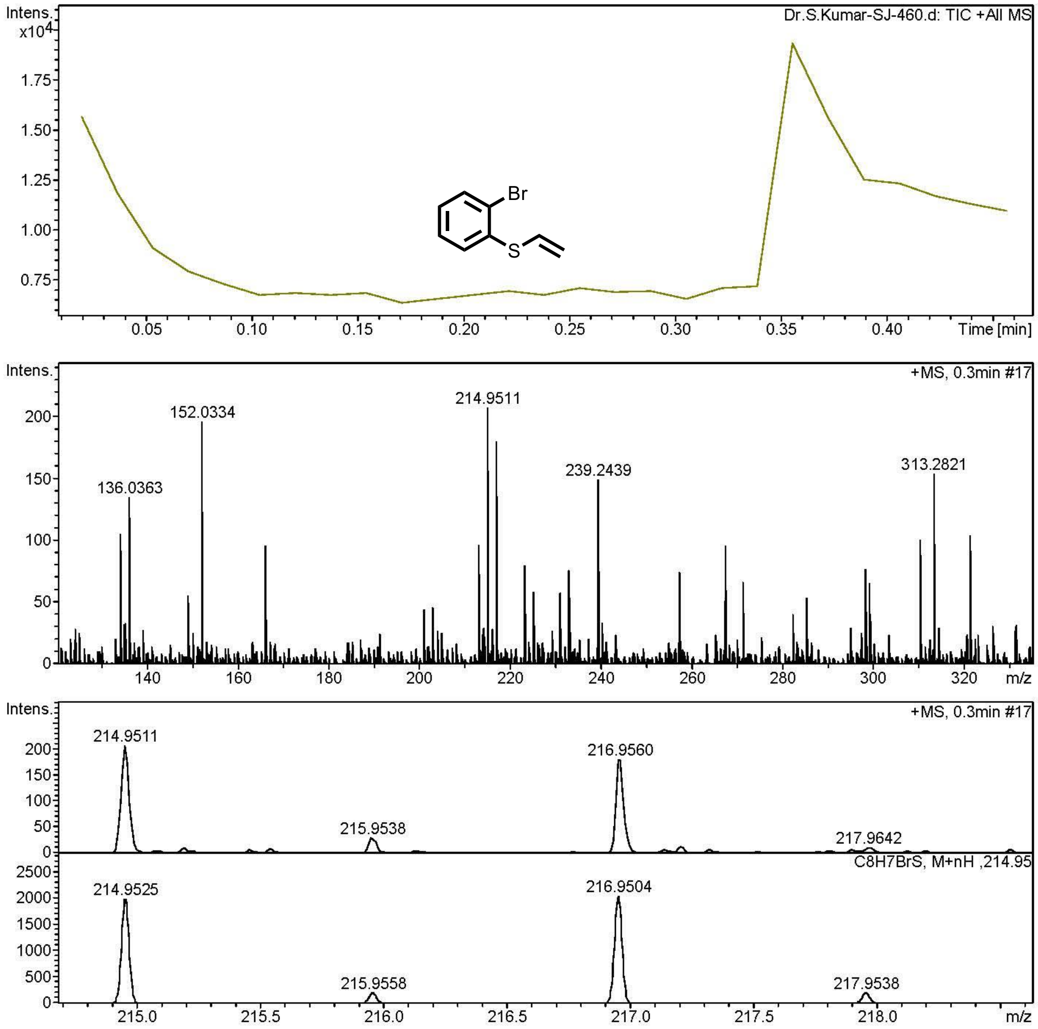

^{1}H NMR of (2-(phenylethynyl)phenyl)(vinyl)sulfane

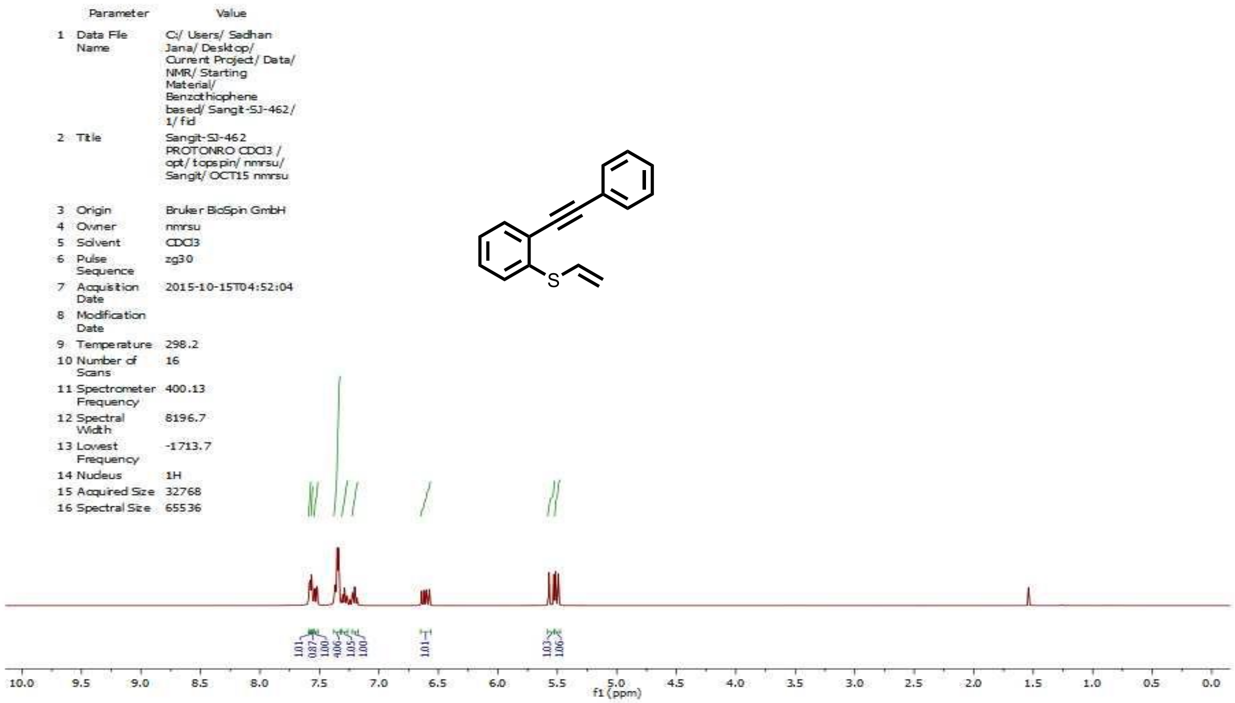

^{13}C NMR of (2-(phenylethynyl)phenyl)(vinyl)sulfane

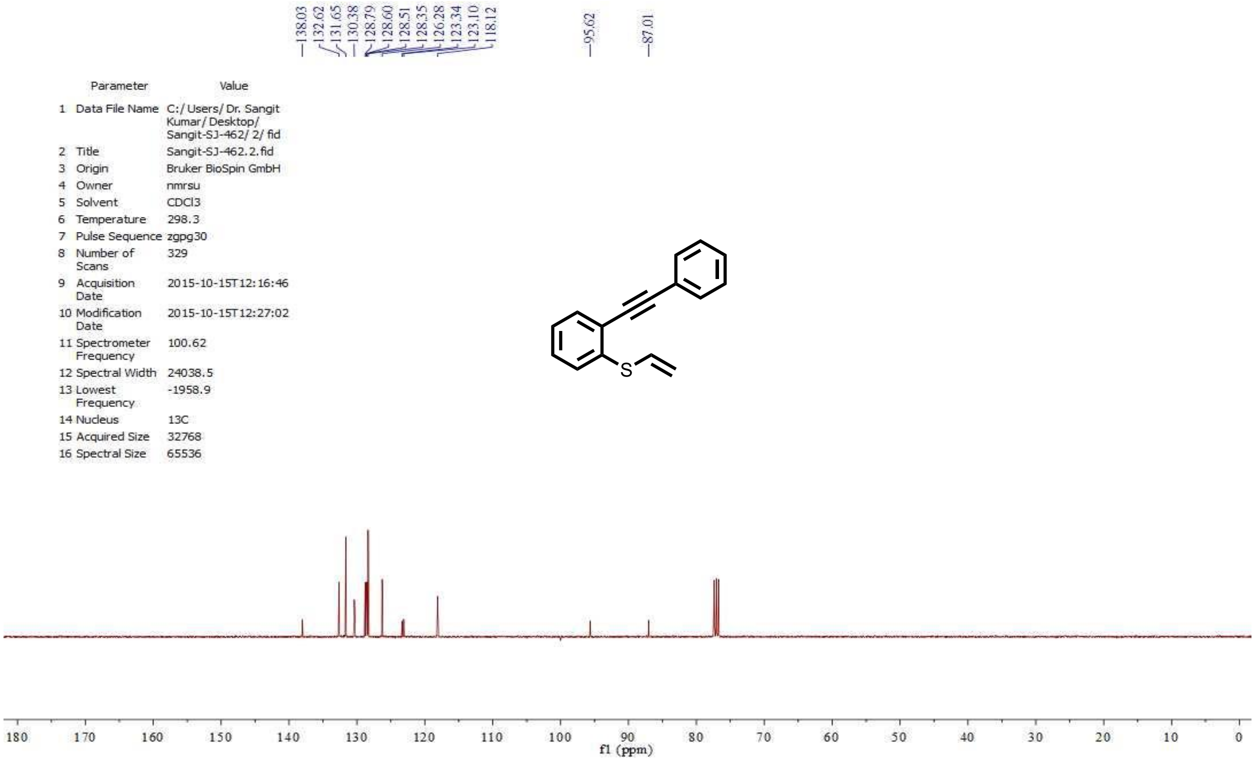

HRMS of (2-(phenylethynyl)phenyl)(vinyl)sulfane

Display Report

Analysis Info

		Acquisition Date	8/19/2016 3:01:49 PM
Analysis Name	D:\Data\user data\2016\August 2016\19-08-2016\Dr.S.Kumar-SJ-462_1-A,2_01_7222.d		
Method	hrlcms_pos_low_tunemix.m	Operator	DIMPLE
Sample Name	Dr.S.Kumar-SJ-462	Instrument	micrOTOF-Q II 10330
Comment			

Acquisition Parameter

Source Type	ESI	Ion Polarity	Positive	Set Nebulizer	1.0 Bar
Focus	Active	Set Capillary	4500 V	Set Dry Heater	250 °C
Scan Begin	50 m/z	Set End Plate Offset	-500 V	Set Dry Gas	7.0 l/min
Scan End	3000 m/z	Set Collision Cell RF	130.0 Vpp	Set Divert Valve	Waste

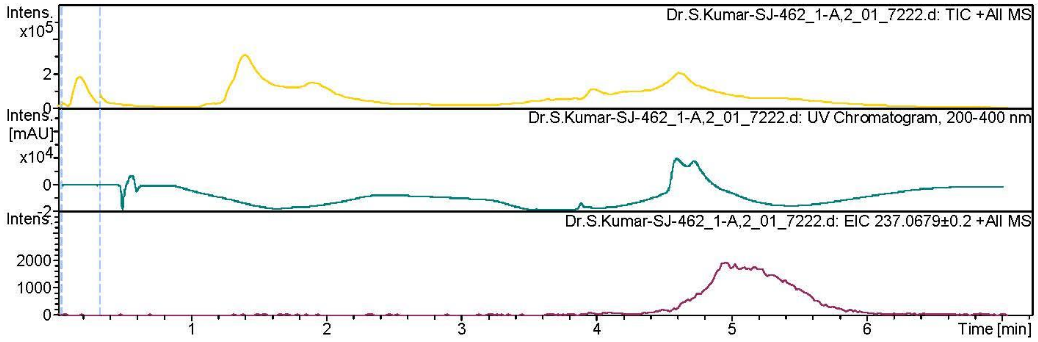

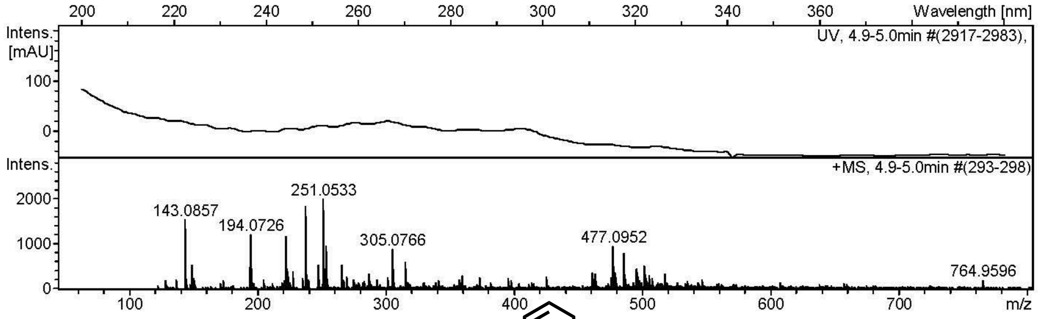

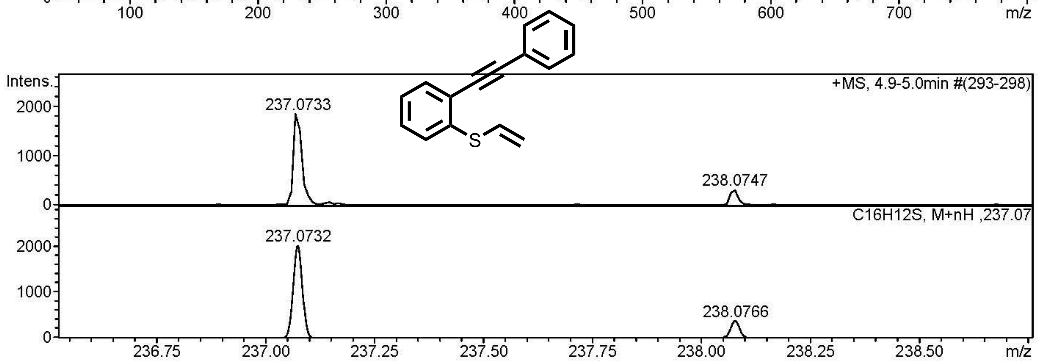

^{1}H NMR of (2-((4-chlorophenyl)ethynyl)phenyl)(vinyl)sulfane

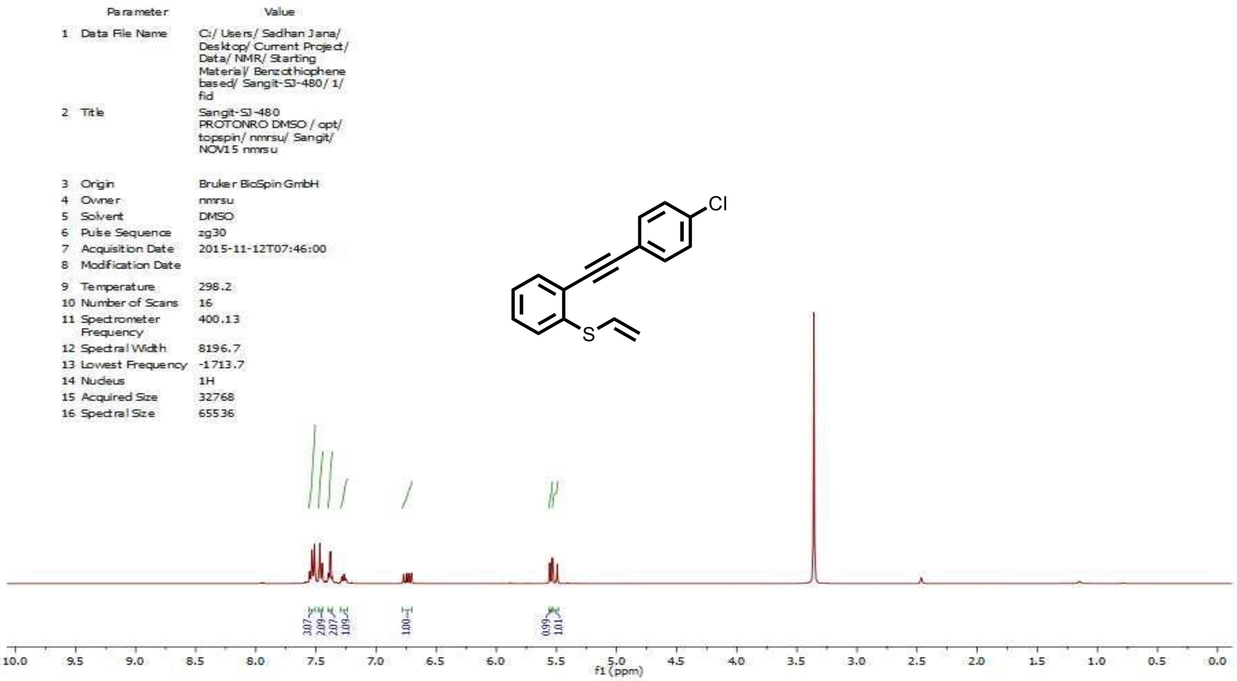

^{13}C NMR of (2-((4-chlorophenyl)ethynyl)phenyl)(vinyl)sulfane

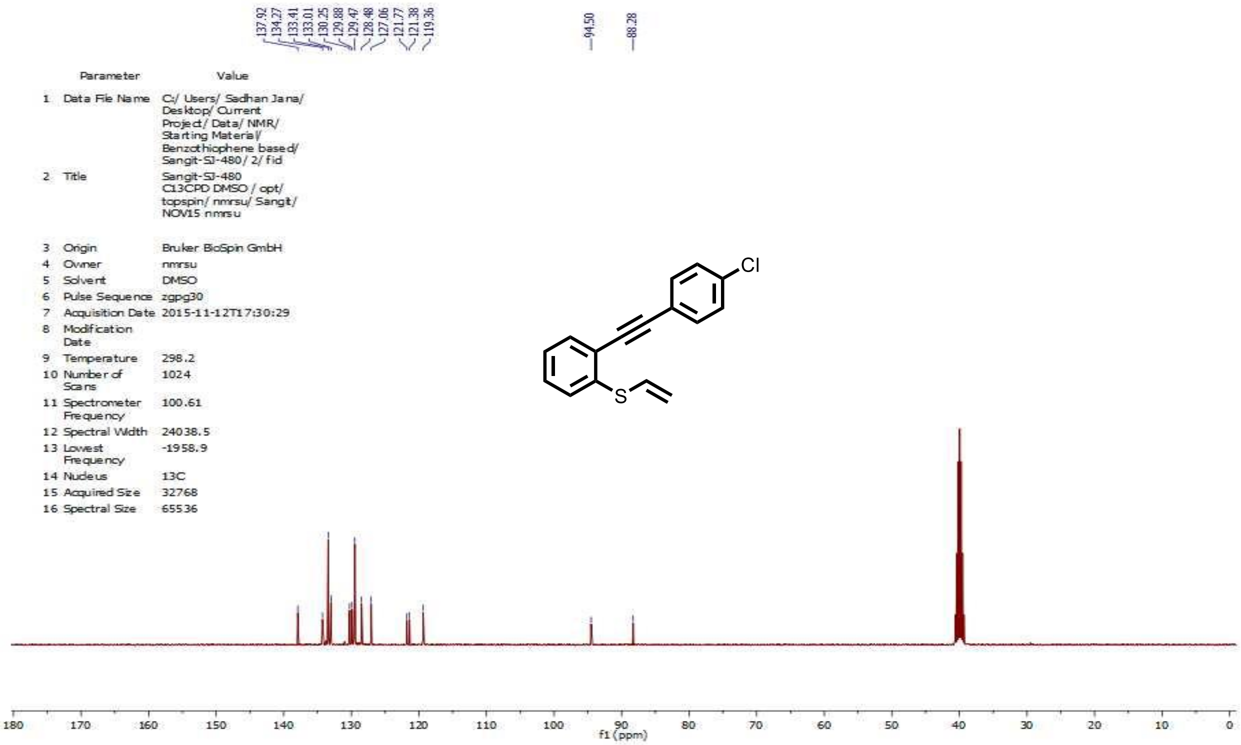

HRMS of (2-((4-chlorophenyl)ethynyl)phenyl)(vinyl)sulfane

Display Report

Analysis Info

Analysis Name	D:\Data\user data\2016\August 2016\29-08-2016\Dr.S.Kumar-SJ-480-APCI.d	Acquisition Date	8/29/2016 3:25:10 PM
Method	tune_low_APCI.m	Operator	DIMPLE
Sample Name	SJ-480-APCI	Instrument	micrOTOF-Q II 10330
Comment			

Acquisition Parameter

Source Type	APCI	Ion Polarity	Positive	Set Nebulizer	2.5 Bar
Focus	Not active	Set Capillary	4500 V	Set Dry Heater	200 °C
Scan Begin	50 m/z	Set End Plate Offset	-500 V	Set Dry Gas	4.0 l/min
Scan End	3000 m/z	Set Collision Cell RF	130.0 Vpp	Set Divert Valve	Waste

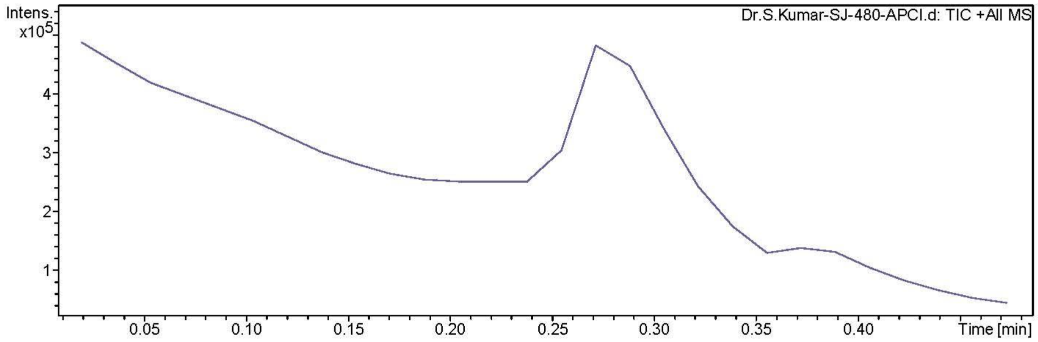

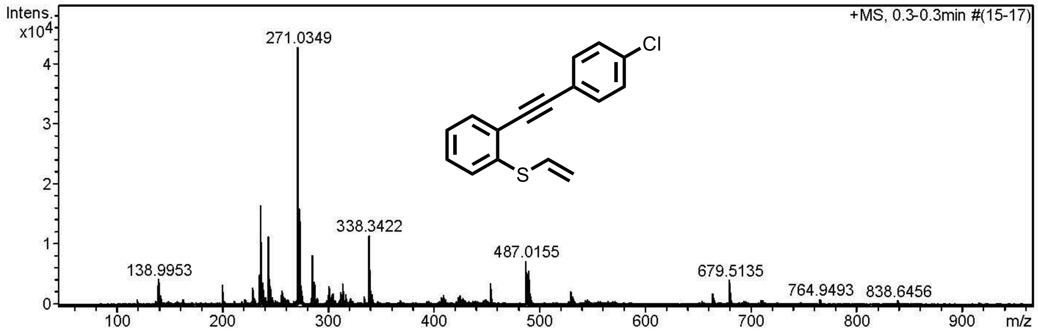

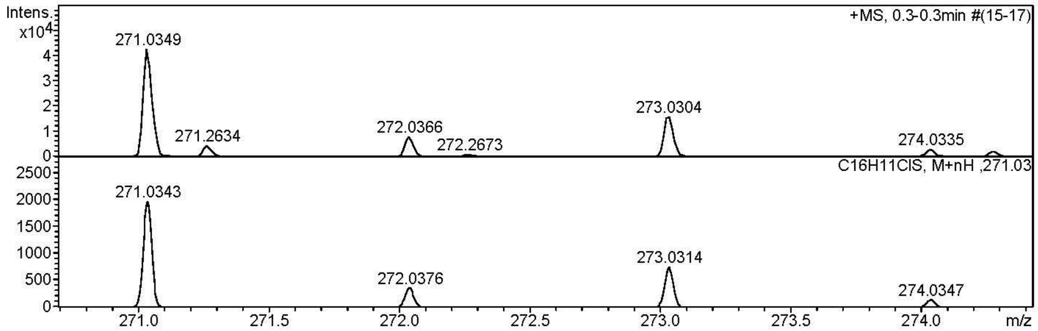

Bruker Compass DataAnalysis 4.0 printed: 8/29/2016 3:48:23 PM Page 1 of 1

^{1}H NMR of (2-(p-tolylethynyl)phenyl)(vinyl)sulfane

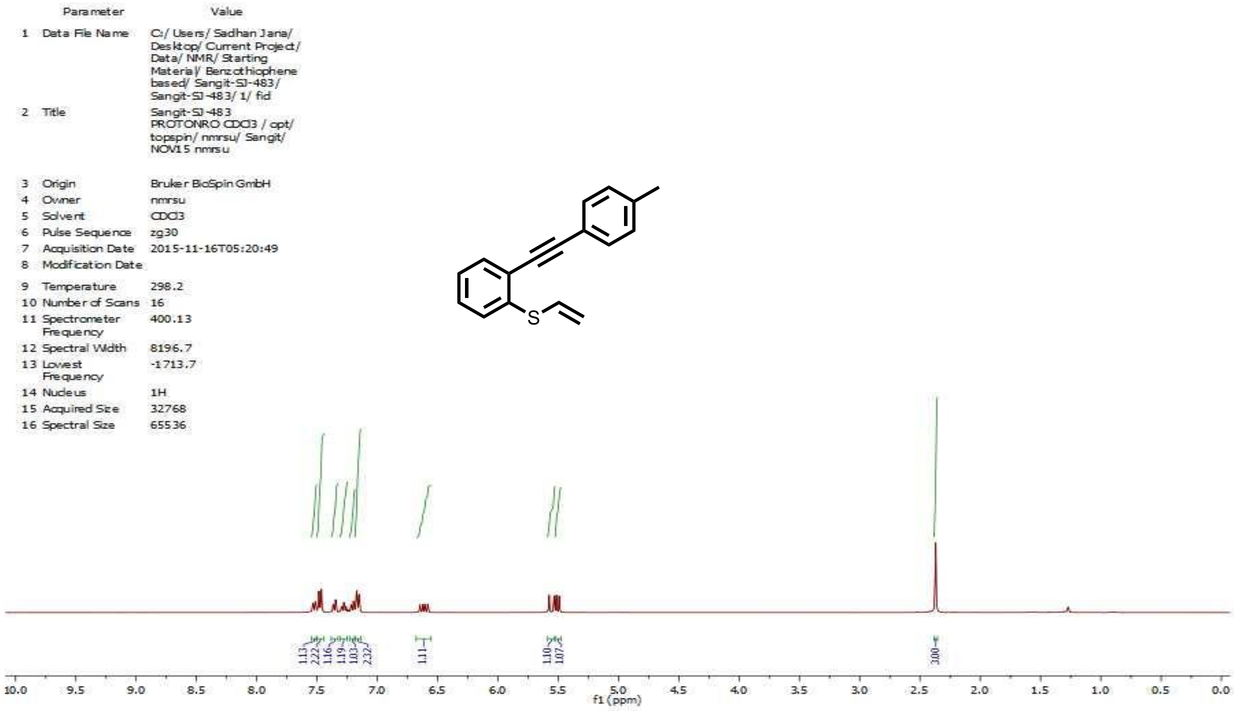

^{13}C NMR of (2-(p-tolylethynyl)phenyl)(vinyl)sulfane

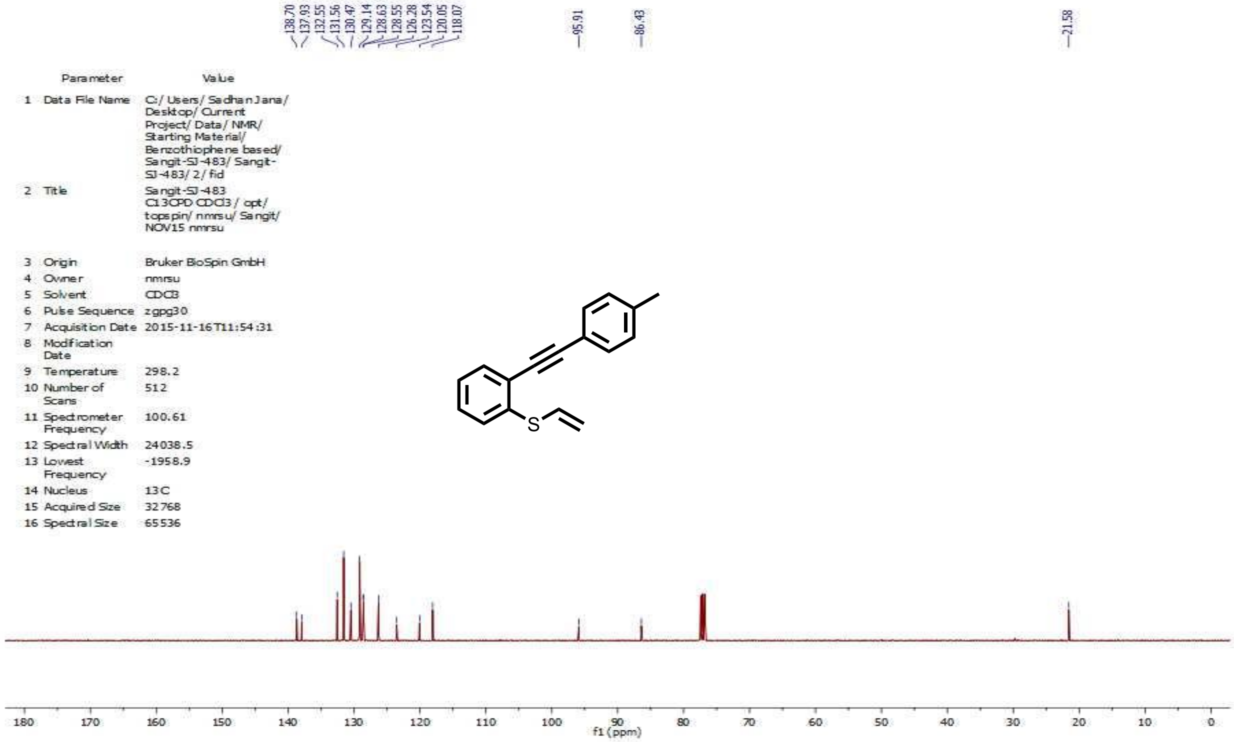

HRMS of (2-(p-tolylethynyl)phenyl)(vinyl)sulfane

Display Report

Analysis Info

		Acquisition Date	8/29/2016 3:23:18 PM
Analysis Name	D:\Data\user data\2016\August 2016\29-08-2016\Dr.S.Kumar-SJ-483-APCI.d		
Method	tune_low_APCI.m	Operator	DIMPLE
Sample Name	SJ-483-APCI	Instrument	micrOTOF-Q II 10330
Comment			

Acquisition Parameter

Source Type	APCI	Ion Polarity	Positive	Set Nebulizer	2.5 Bar
Focus	Not active	Set Capillary	4500 V	Set Dry Heater	200 °C
Scan Begin	50 m/z	Set End Plate Offset	-500 V	Set Dry Gas	4.0 l/min
Scan End	3000 m/z	Set Collision Cell RF	130.0 Vpp	Set Divert Valve	Waste

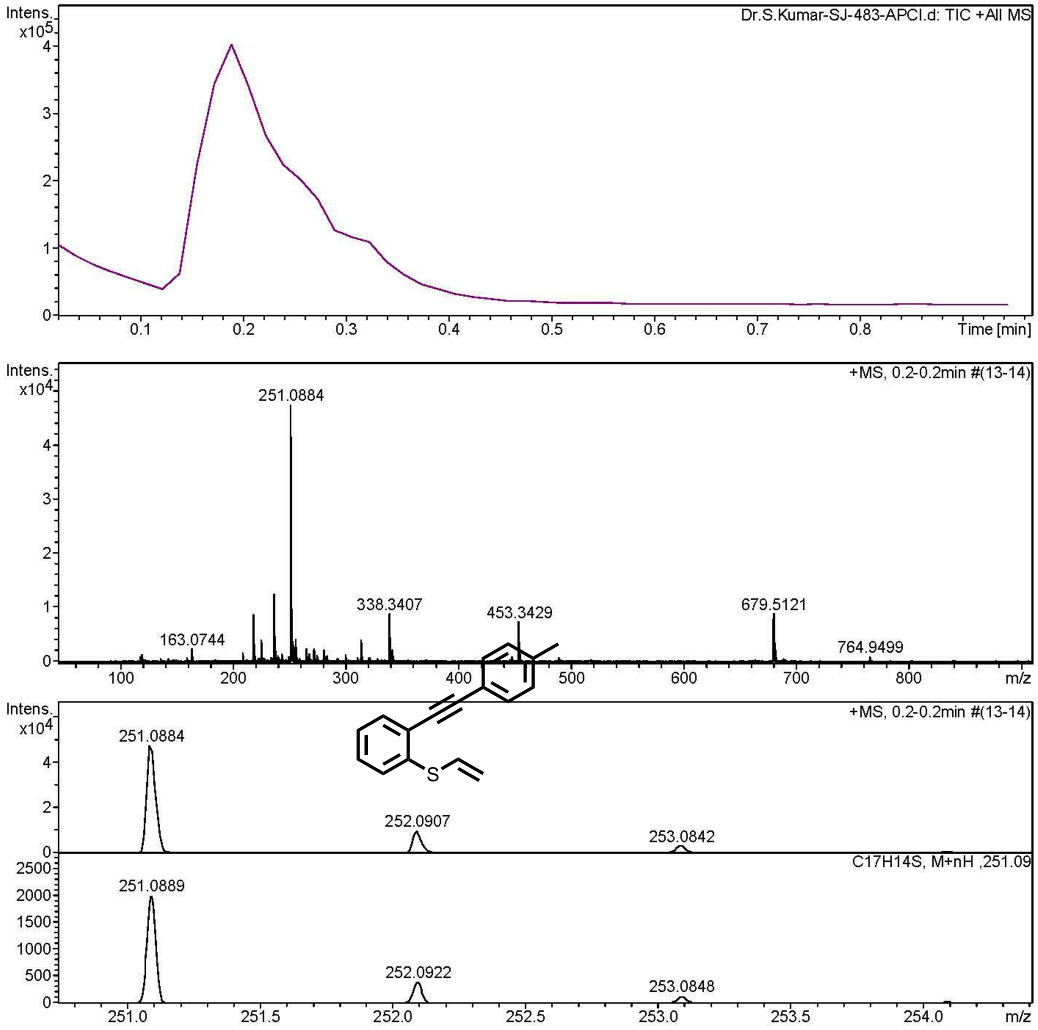

Bruker Compass DataAnalysis 4.0 printed: 8/29/2016 4:01:03 PM Page 1 of 1

^{1}H NMR of (2-((3-fluorophenyl)ethynyl)phenyl)(vinyl)sulfane

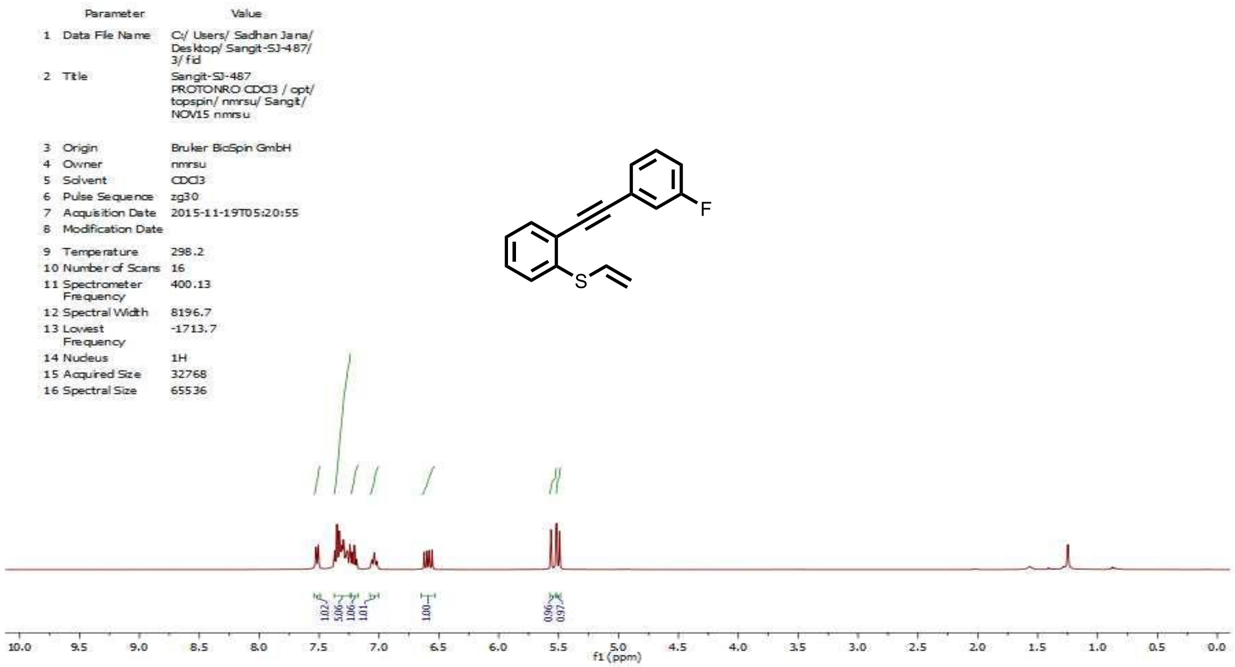

^{13}C NMR of (2-((3-fluorophenyl)ethynyl)phenyl)(vinyl)sulfane

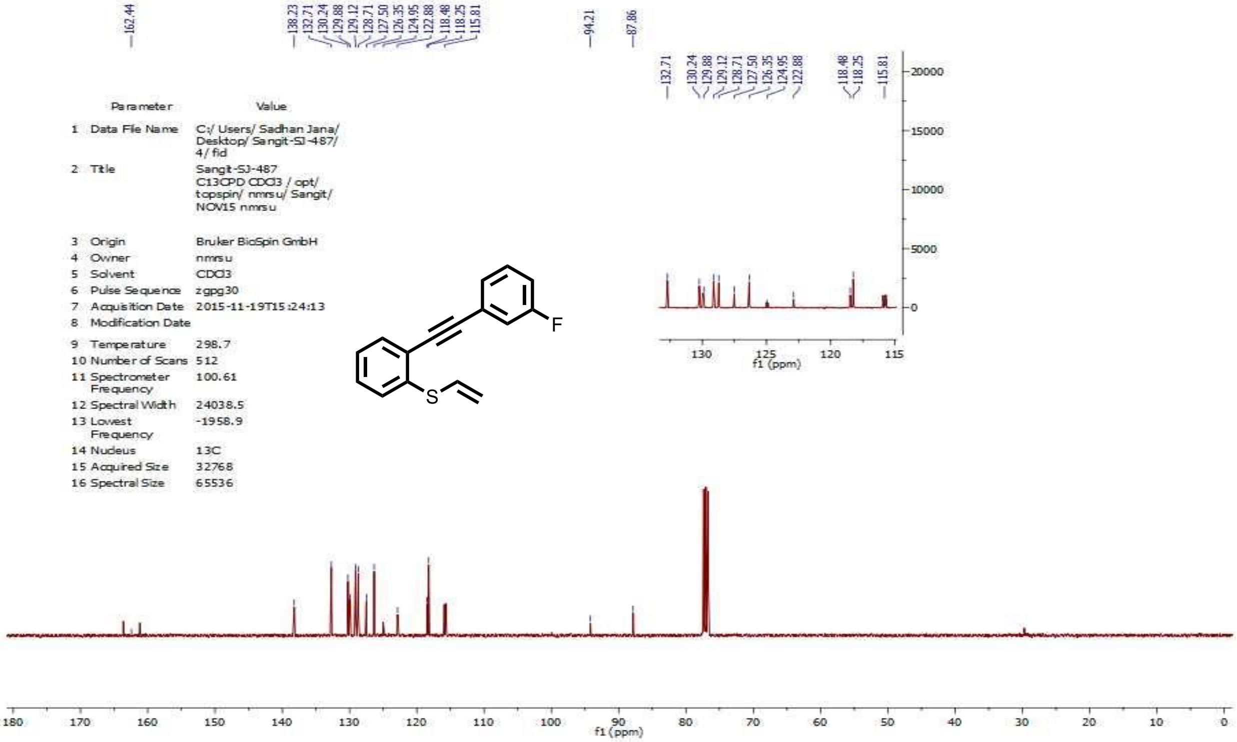

HRMS of (2-((3-fluorophenyl)ethynyl)phenyl)(vinyl)sulfane

Display Report

Analysis Info

		Acquisition Date	8/29/2016 3:21:04 PM
Analysis Name	D:\Data\user data\2016\August 2016\29-08-2016\Dr.S.Kumar-SJ-487-APCI.d		
Method	tune_low_APCI.m	Operator	DIMPLE
Sample Name	SJ-487-APCI	Instrument	micrOTOF-Q II 10330
Comment			

Acquisition Parameter

Source Type	APCI	Ion Polarity	Positive	Set Nebulizer	2.5 Bar
Focus	Not active	Set Capillary	4500 V	Set Dry Heater	200 °C
Scan Begin	50 m/z	Set End Plate Offset	-500 V	Set Dry Gas	4.0 l/min
Scan End	3000 m/z	Set Collision Cell RF	130.0 Vpp	Set Divert Valve	Waste

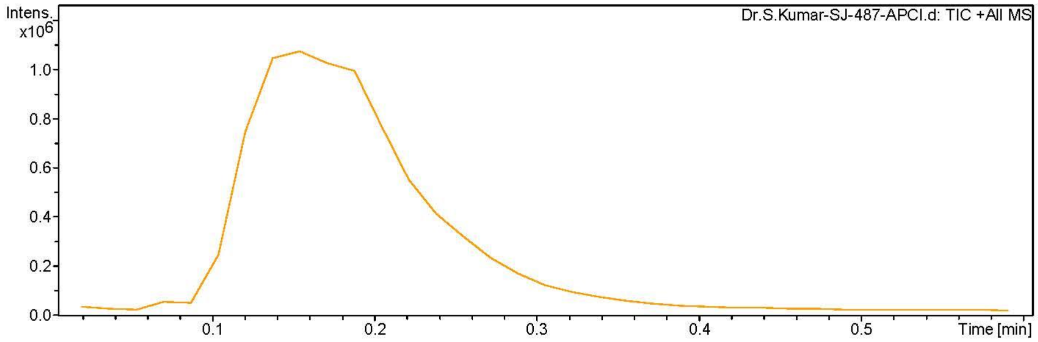

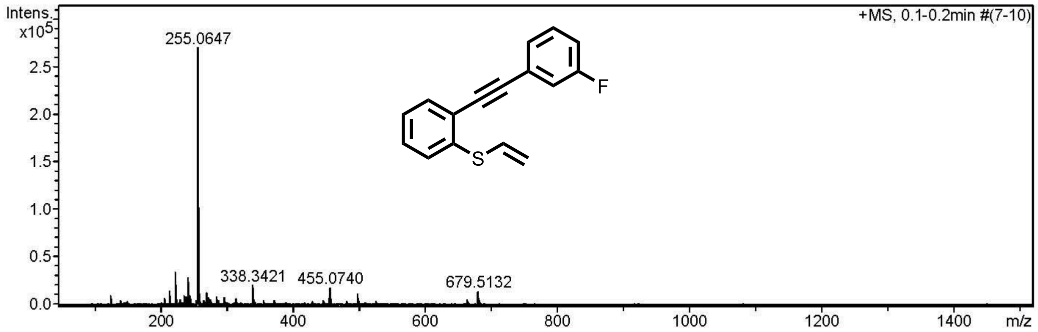

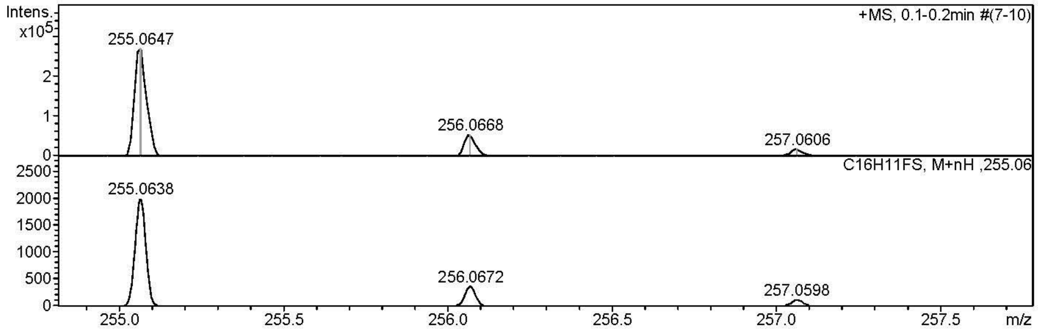

^{1}H NMR of (2-((3-Methoxyphenyl)ethynyl)phenyl)(vinyl)sulfane

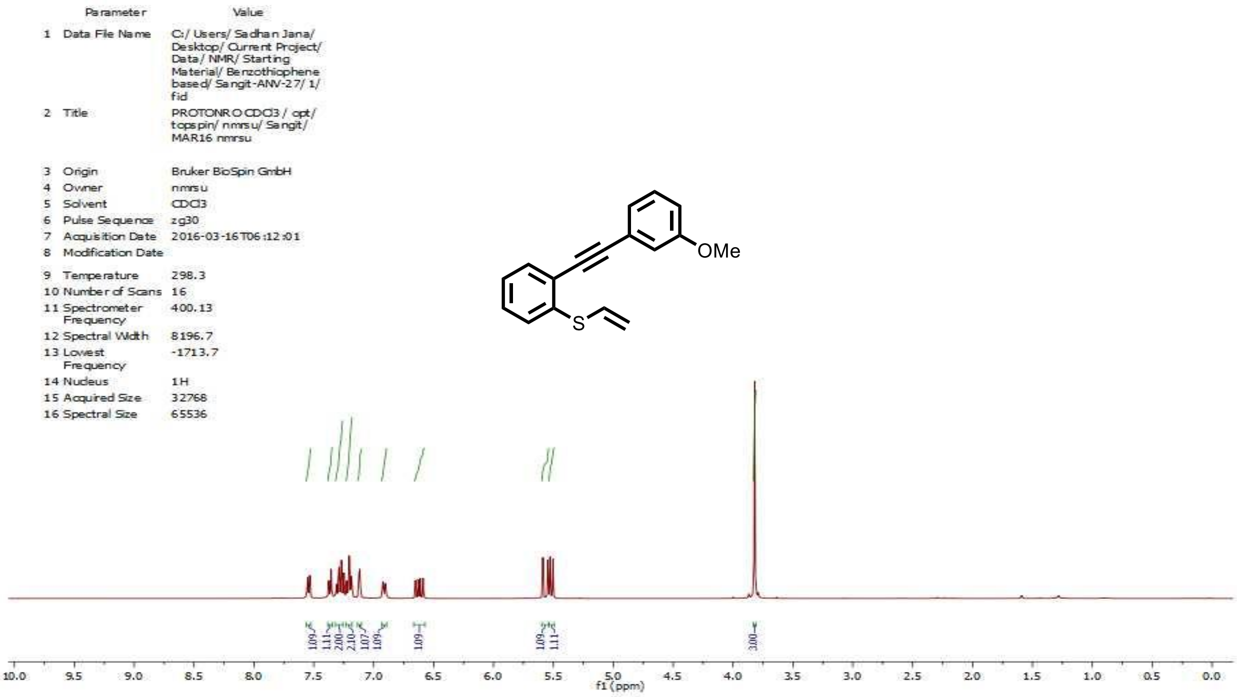

^{13}C NMR of (2-((3-Methoxyphenyl)ethynyl)phenyl)(vinyl)sulfane

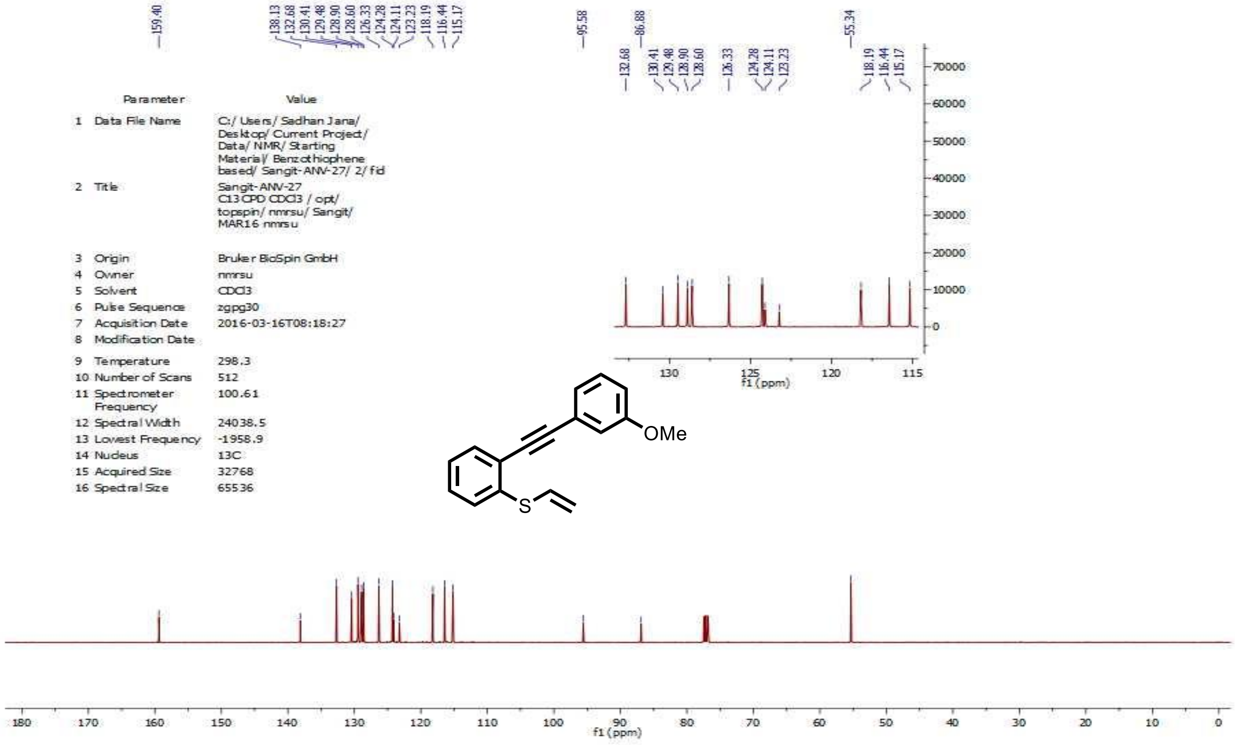

HRMS of (2-((3-Methoxyphenyl)ethynyl)phenyl)(vinyl)sulfane

Display Report

Analysis Info

		Acquisition Date	8/30/2016 1:50:52 PM
Analysis Name	D:\Data\user data\2016\August 2016\30-08-2016\Dr.S.Kumar-ANV-27_1-B,2_01_7271.d		
Method	hrlcms_pos_low_tunemix.m	Operator	DIMPLE
Sample Name	Dr.S.Kumar-ANV-27	Instrument	micrOTOF-Q II 10330
Comment			

Acquisition Parameter

Source Type	ESI	Ion Polarity	Positive	Set Nebulizer	1.0 Bar
Focus	Active	Set Capillary	4500 V	Set Dry Heater	250 °C
Scan Begin	50 m/z	Set End Plate Offset	-500 V	Set Dry Gas	7.0 l/min
Scan End	3000 m/z	Set Collision Cell RF	130.0 Vpp	Set Divert Valve	Waste

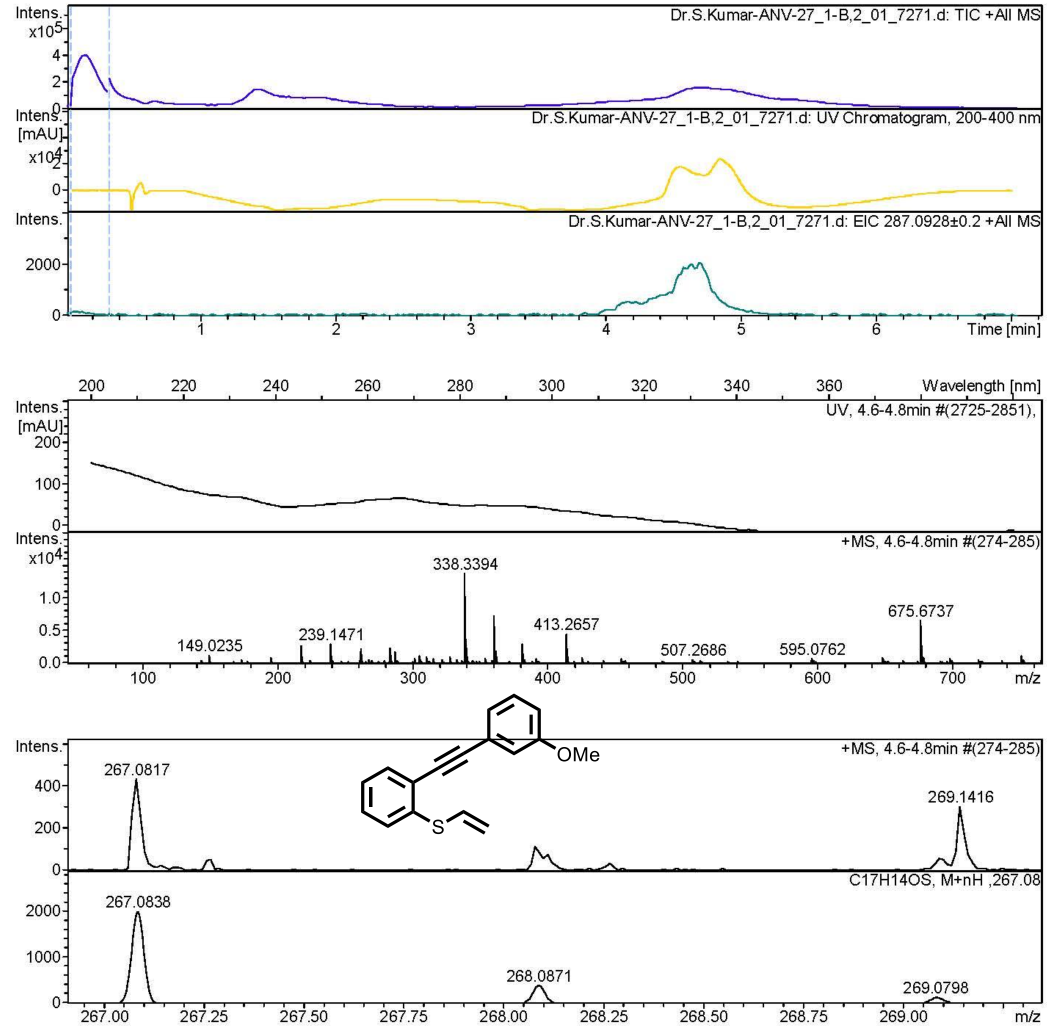

^{1}H NMR of (2-((2-(Trifluoromethyl)phenyl)ethynyl)phenyl)(vinyl)sulfane

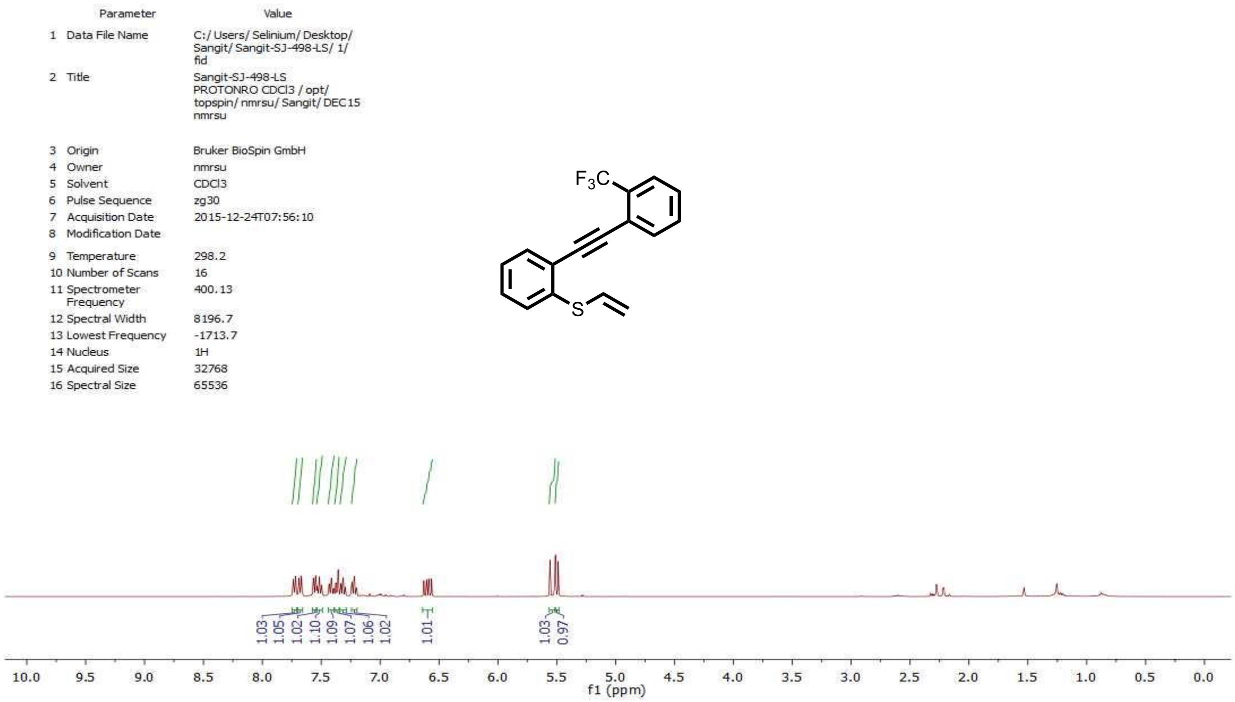

^{13}C NMR of (2-((2-(Trifluoromethyl)phenyl)ethynyl)phenyl)(vinyl)sulfane

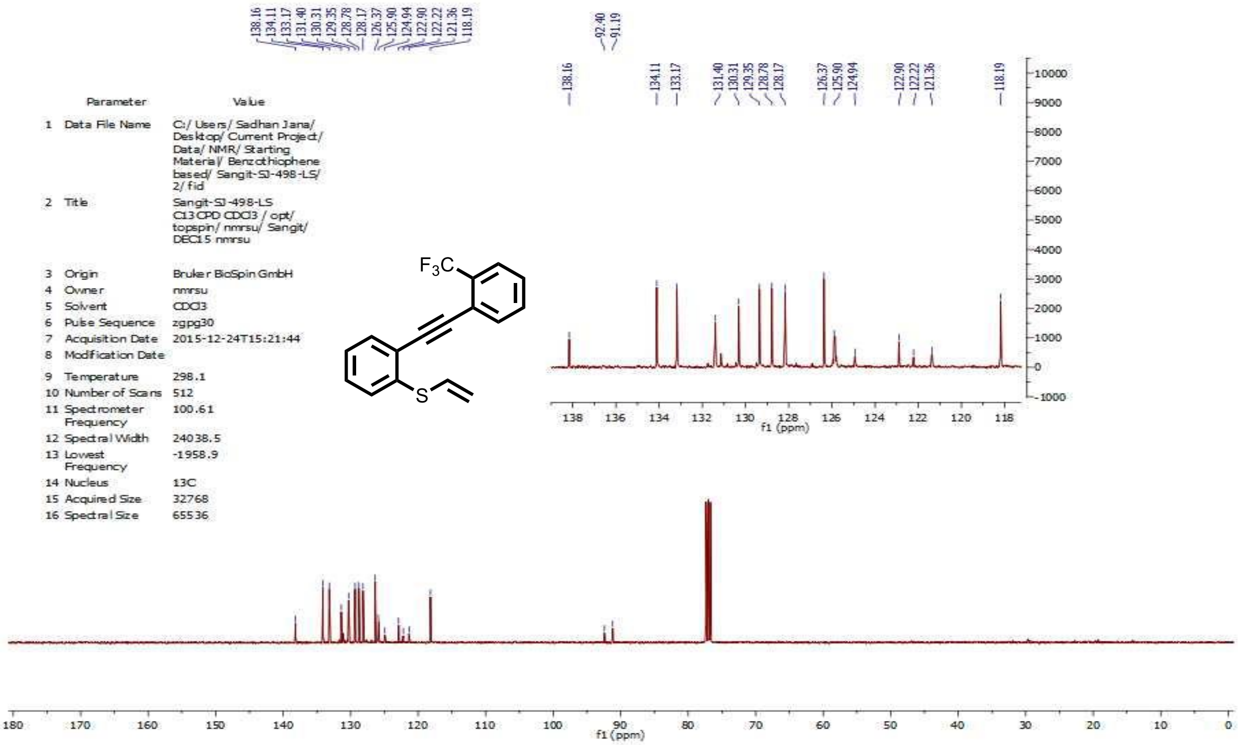

HRMS of (2-((2-(Trifluoromethyl)phenyl)ethynyl)phenyl)(vinyl)sulfane

Display Report

Analysis Info

		Acquisition Date	8/10/2016 12:57:07 PM
Analysis Name	D:\Data\user data\2016\August 2016\10-08-2016\Dr.S.Kumar-SJ-498_1-A,2_01_7145.d		
Method	hrlcms_pos_low_tunemix.m	Operator	DIMPLE
Sample Name	Dr.S.Kumar-SJ-498	Instrument	micrOTOF-Q II 10330
Comment			

Acquisition Parameter

Source Type	ESI	Ion Polarity	Positive	Set Nebulizer	1.0 Bar
Focus	Active	Set Capillary	4500 V	Set Dry Heater	250 °C
Scan Begin	50 m/z	Set End Plate Offset	-500 V	Set Dry Gas	7.0 l/min
Scan End	3000 m/z	Set Collision Cell RF	130.0 Vpp	Set Divert Valve	Waste

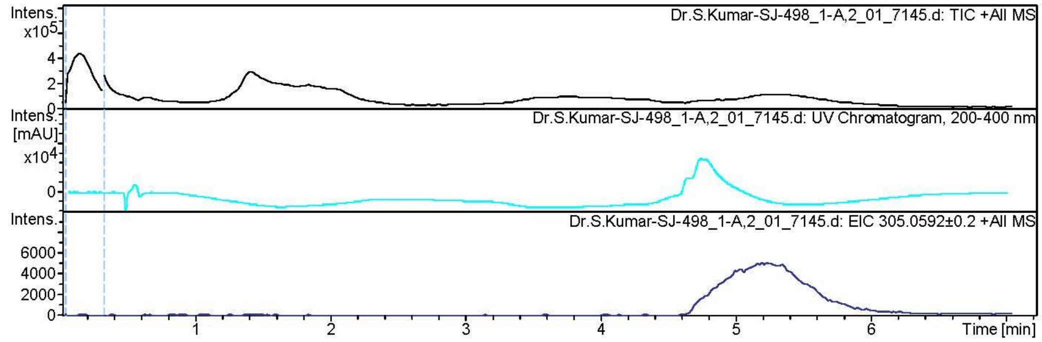

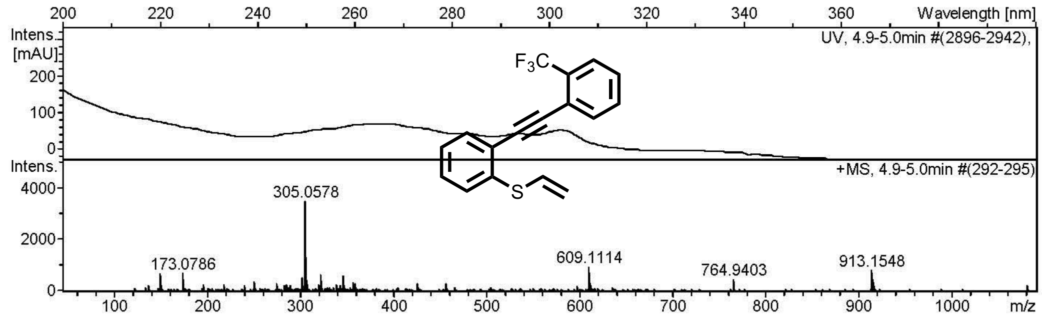

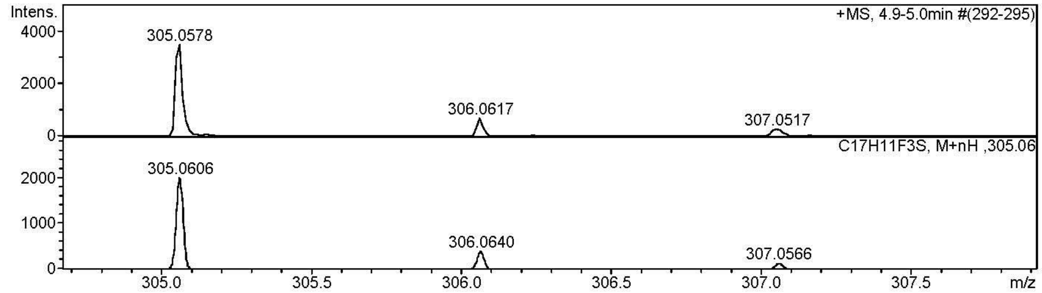

Bruker Compass DataAnalysis 4.0 printed: 8/10/2016 4:06:49 PM Page 1 of 1

^{1}H NMR of (2-(*o*-Tolylethynyl)phenyl)(vinyl)sulfane

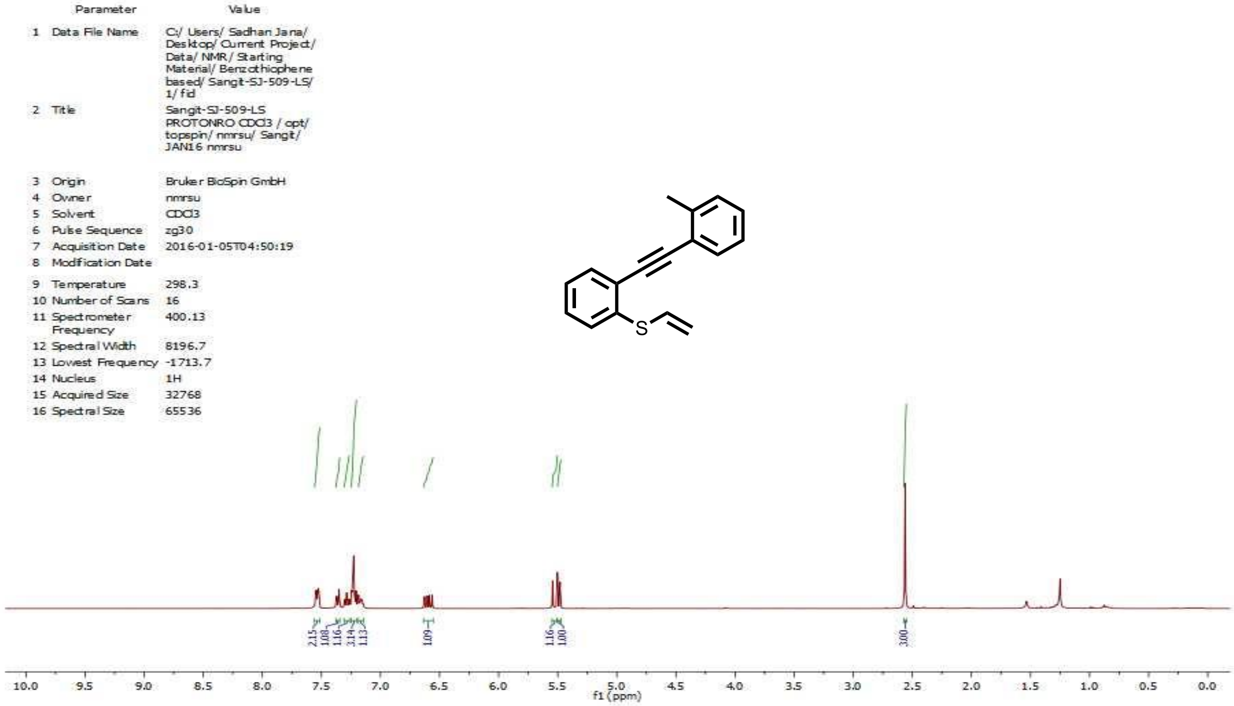

^{13}C NMR of (2-(*o*-Tolylethynyl)phenyl)(vinyl)sulfane

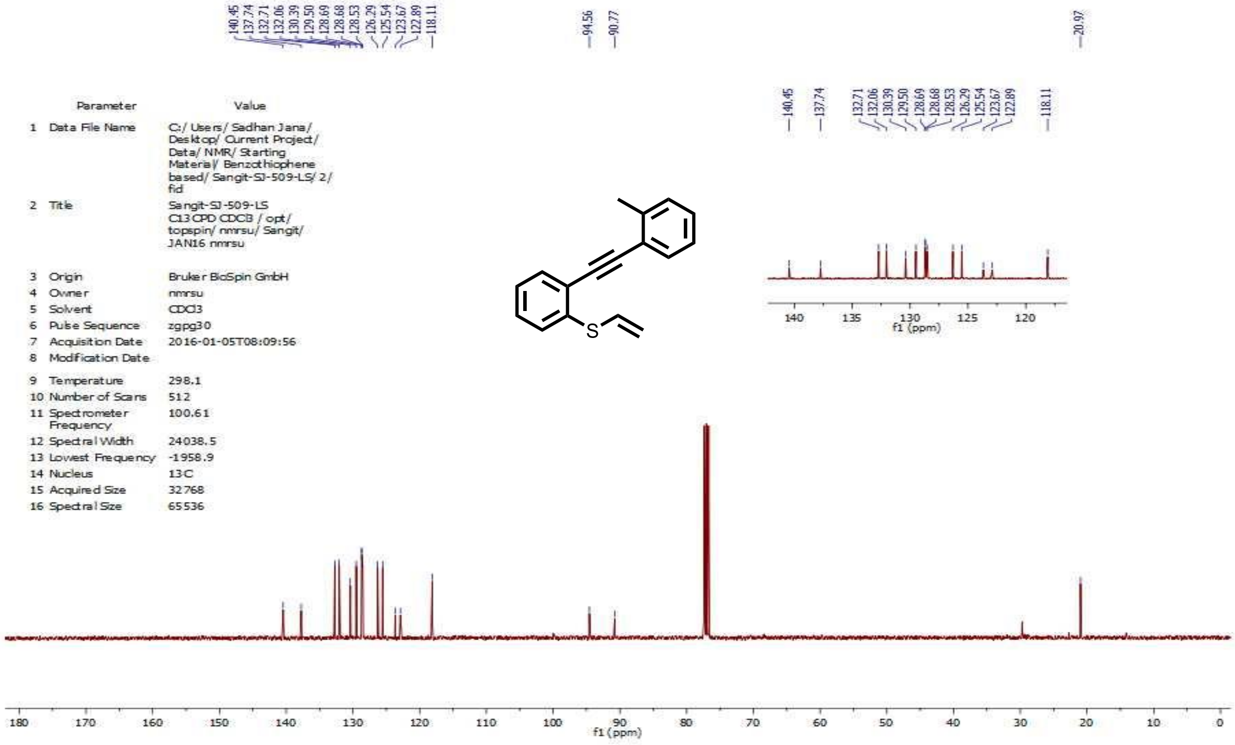

HRMS NMR of (2-(*o*-Tolylethynyl)phenyl)(vinyl)sulfane

Display Report

Analysis Info

Analysis Name	D:\Data\user data\2016\August 2016\11-08-2016\Dr.S.Kumar-SJ-509.d	Acquisition Date	8/11/2016 2:51:45 PM
Method	tune_low_APCI.m	Operator	DIMPLE
Sample Name	SJ-509	Instrument	micrOTOF-Q II 10330
Comment			

Acquisition Parameter

Source Type	APCI	Ion Polarity	Positive	Set Nebulizer	2.5 Bar
Focus	Not active	Set Capillary	4500 V	Set Dry Heater	200 °C
Scan Begin	50 m/z	Set End Plate Offset	-500 V	Set Dry Gas	4.0 l/min
Scan End	3000 m/z	Set Collision Cell RF	130.0 Vpp	Set Divert Valve	Waste

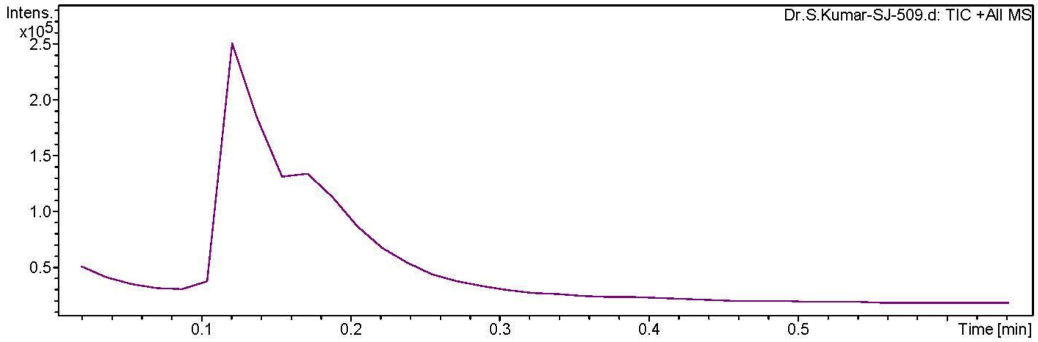

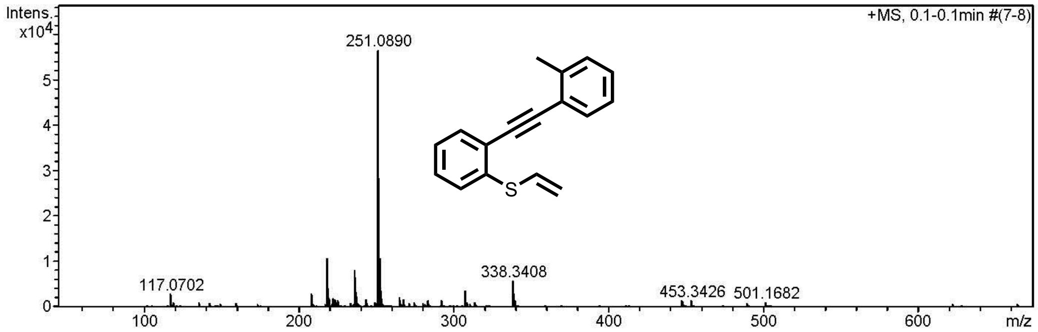

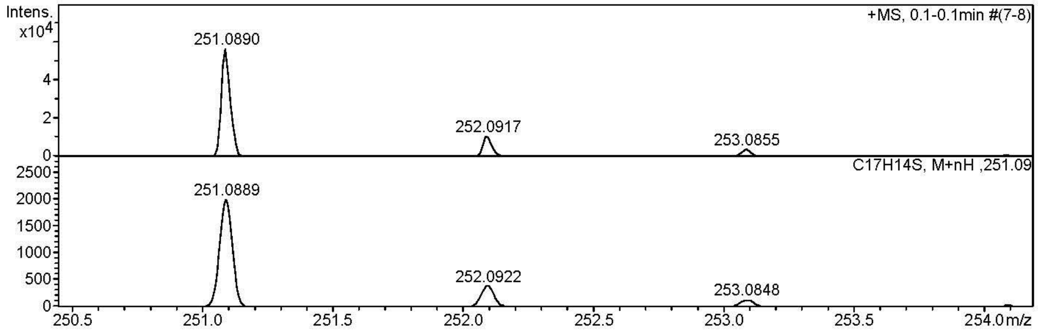

Bruker Compass DataAnalysis 4.0 printed: 8/11/2016 3:41:12 PM Page 1 of 1

^{1}H NMR of 5-((2-(Vinylthio)phenyl)ethynyl)benzo[*d*][1,3]dioxole

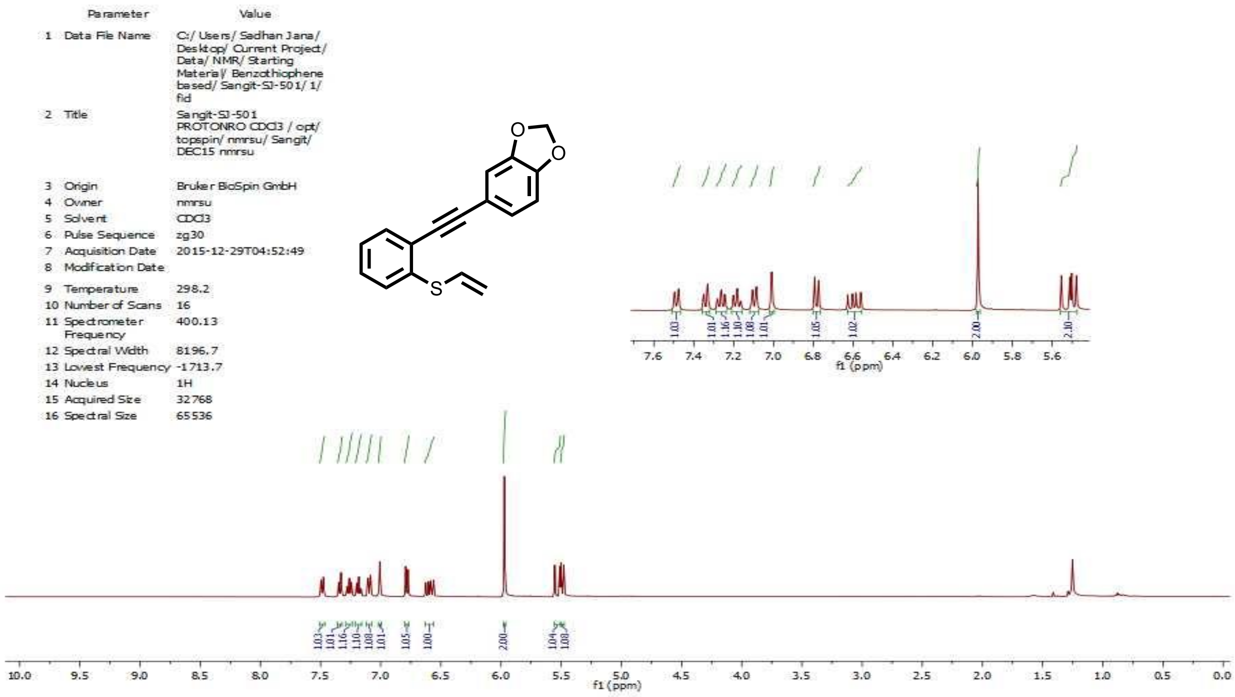

^{13}C NMR of 5-((2-(Vinylthio)phenyl)ethynyl)benzo[*d*][1,3]dioxole

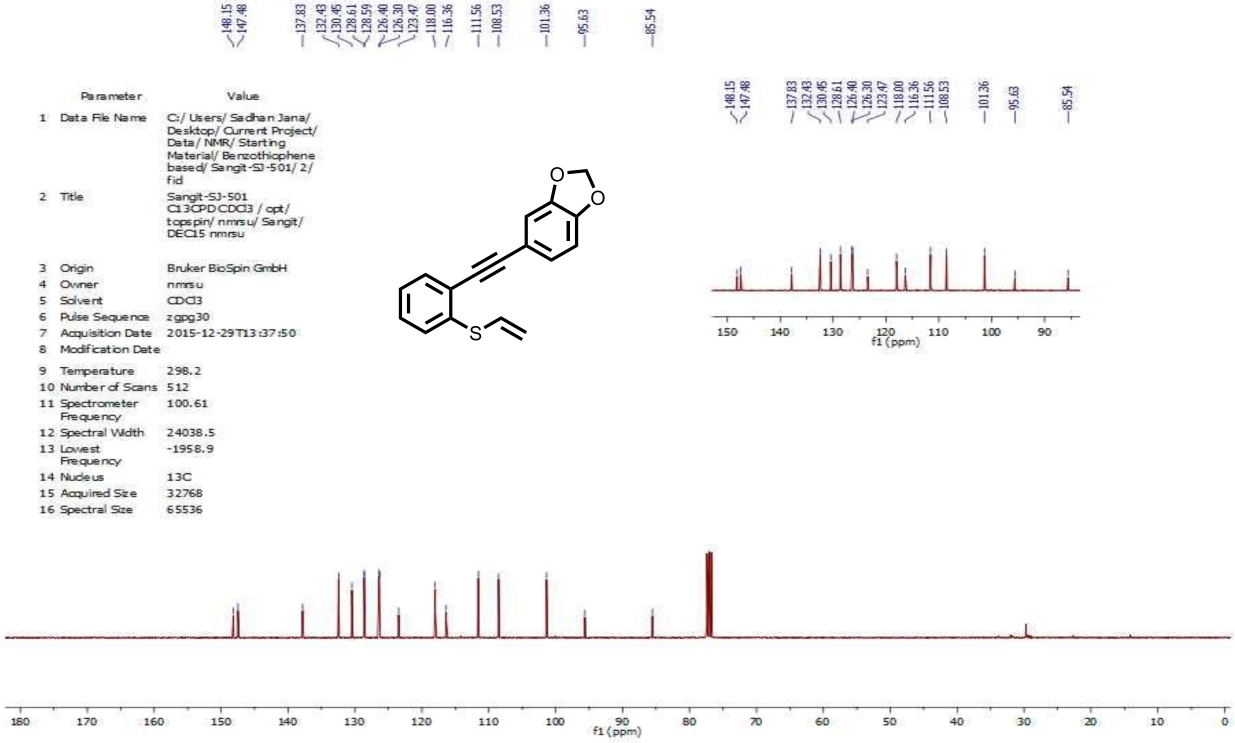

HRMS of 5-((2-(Vinylthio)phenyl)ethynyl)benzo[*d*][1,3]dioxole

Display Report

Analysis Info

Analysis Name	D:\Data\user data\2016\August 2016\05-08-2016\Dr.S.Kumar-SJ-501_1-A,4_01_7066.d	Acquisition Date	8/5/2016 12:35:25 PM
Method	hrlcms_pos_low_tunemix.m	Operator	DIMPLE
Sample Name	Dr.S.Kumar-SJ-501	Instrument	micrOTOF-Q II 10330
Comment			

Acquisition Parameter

Source Type	ESI	Ion Polarity	Positive	Set Nebulizer	1.0 Bar
Focus	Active	Set Capillary	4500 V	Set Dry Heater	250 °C
Scan Begin	50 m/z	Set End Plate Offset	-500 V	Set Dry Gas	7.0 l/min
Scan End	3000 m/z	Set Collision Cell RF	130.0 Vpp	Set Divert Valve	Waste

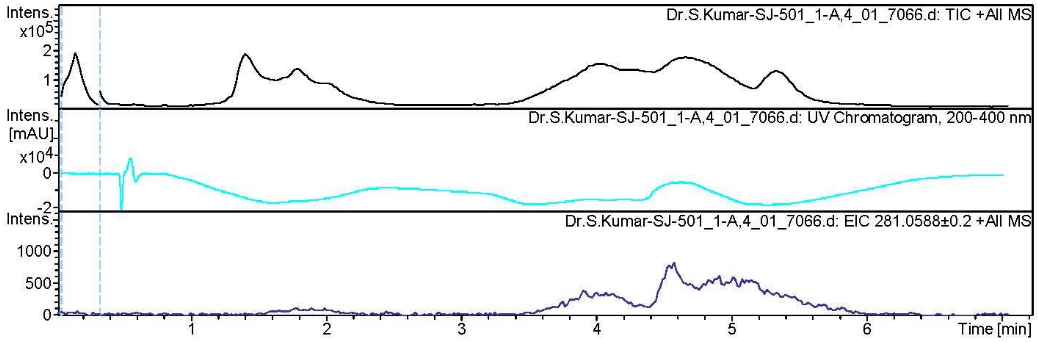

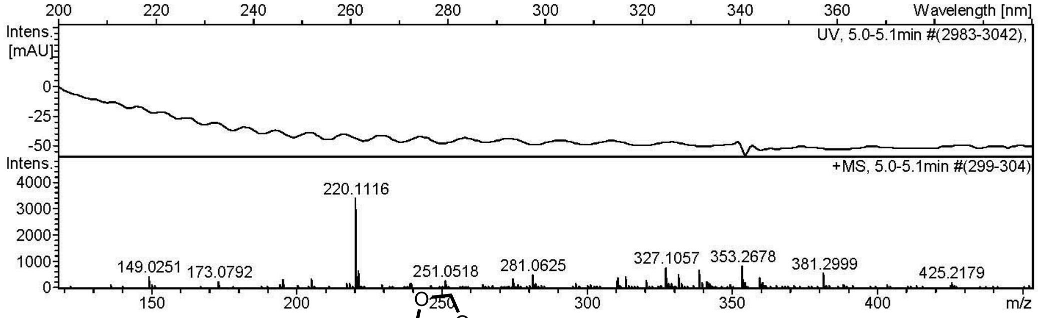

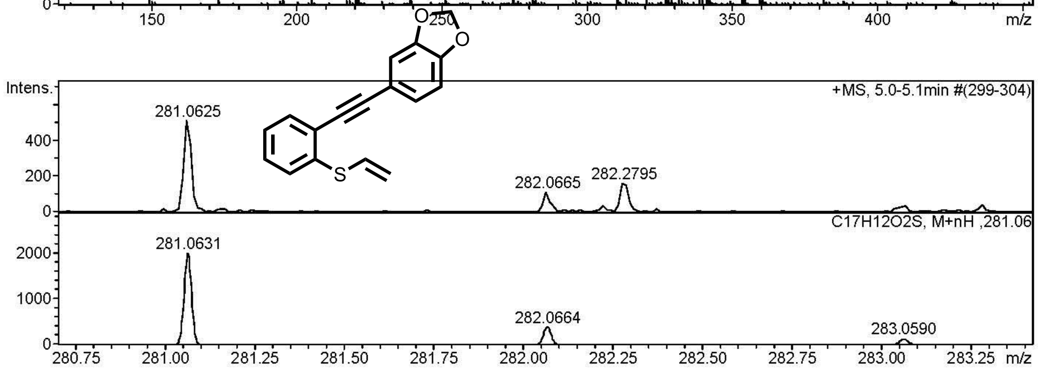

^{1}H NMR of (2-((3,4,5-Trimethoxyphenyl)ethynyl)phenyl)(vinyl)sulfane

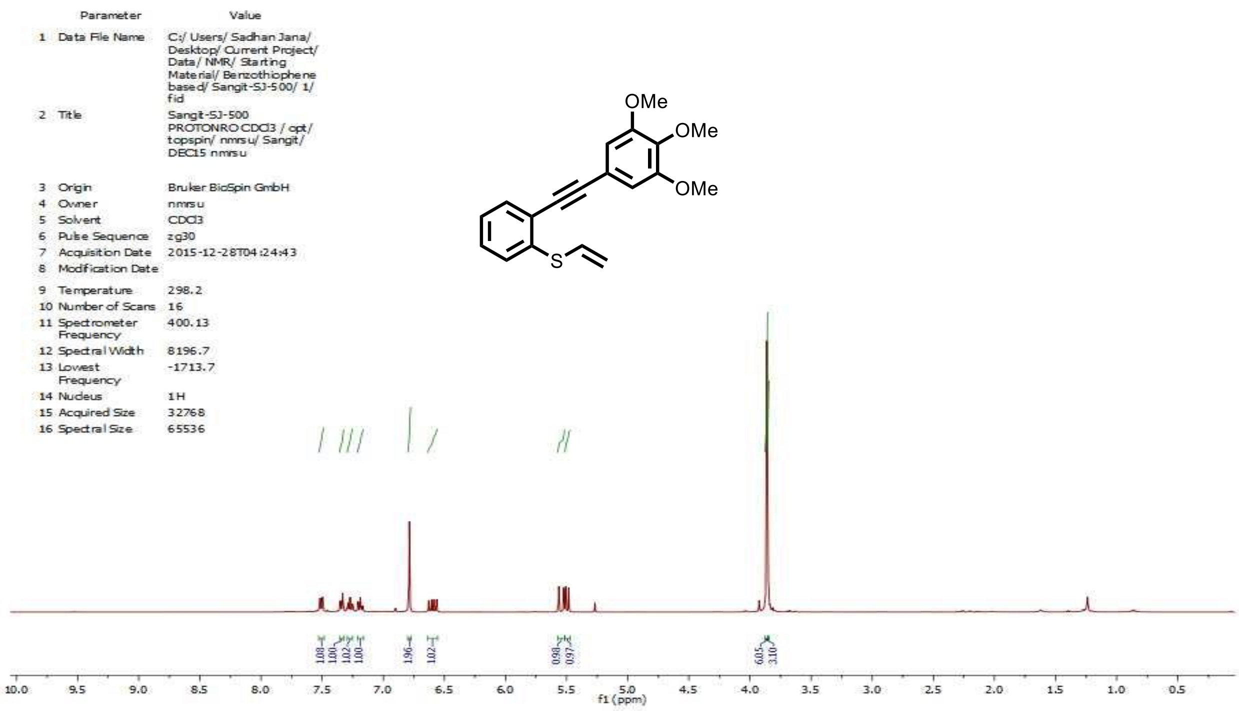

^{13}C NMR of (2-((3,4,5-Trimethoxyphenyl)ethynyl)phenyl)(vinyl)sulfane

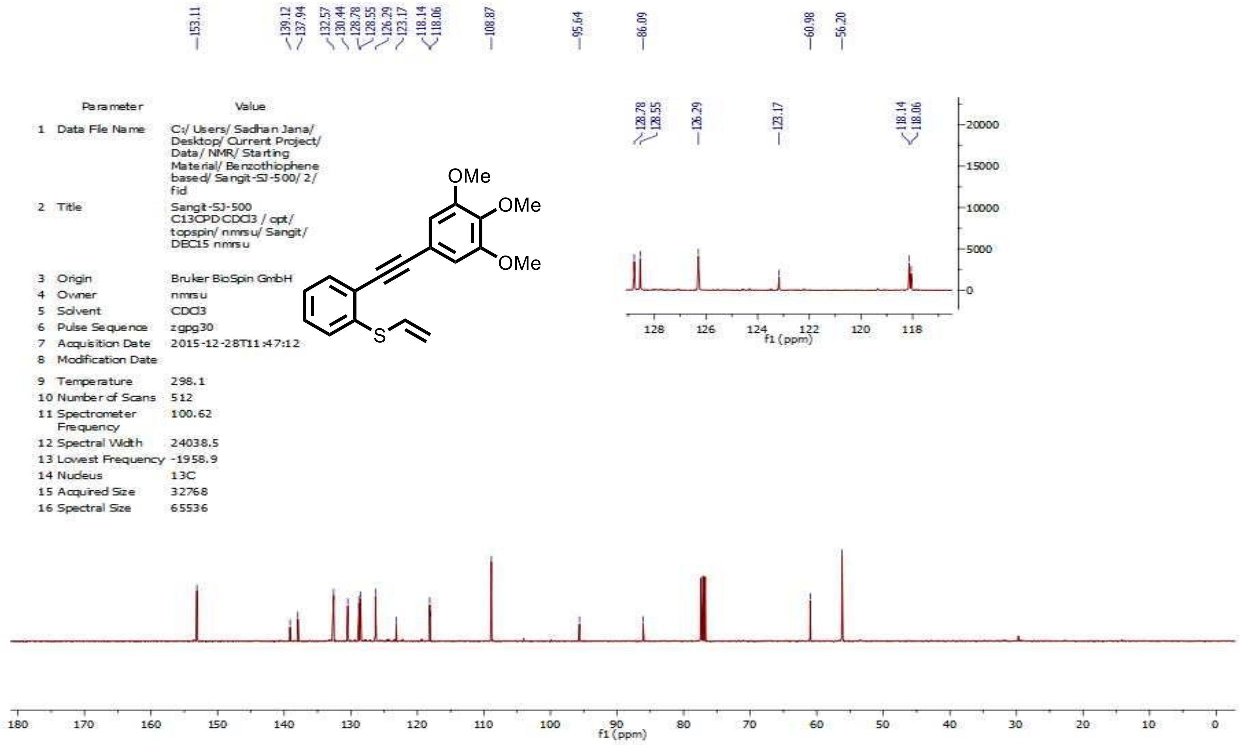

HRMS of (2-((3,4,5-Trimethoxyphenyl)ethynyl)phenyl)(vinyl)sulfane

Display Report

Analysis Info

Analysis Name	D:\Data\user data\2016\August 2016\10-08-2016\Dr.S.Kumar-SJ-556_1-A,5_01_7148.d	Acquisition Date	8/10/2016 1:21:34 PM
Method	hrlcms_pos_low_tunemix.m	Operator	DIMPLE
Sample Name	Dr.S.Kumar-SJ-556	Instrument	micrOTOF-Q II 10330
Comment			

Acquisition Parameter

Source Type	ESI	Ion Polarity	Positive	Set Nebulizer	1.0 Bar
Focus	Active	Set Capillary	4500 V	Set Dry Heater	250 °C
Scan Begin	50 m/z	Set End Plate Offset	-500 V	Set Dry Gas	7.0 l/min
Scan End	3000 m/z	Set Collision Cell RF	130.0 Vpp	Set Divert Valve	Waste

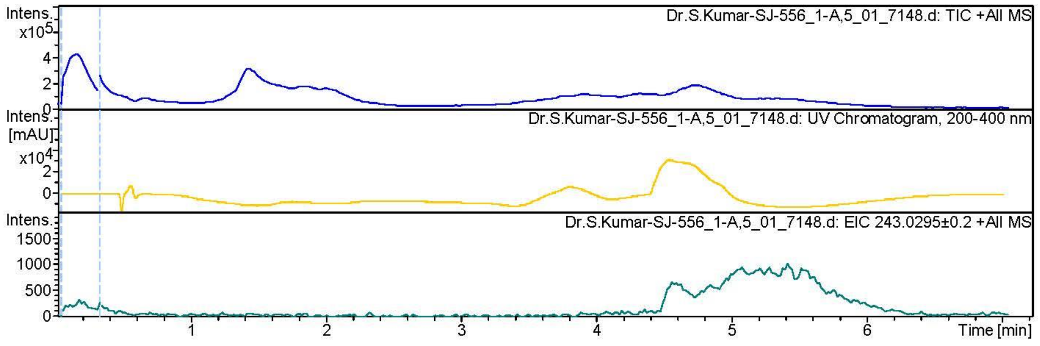

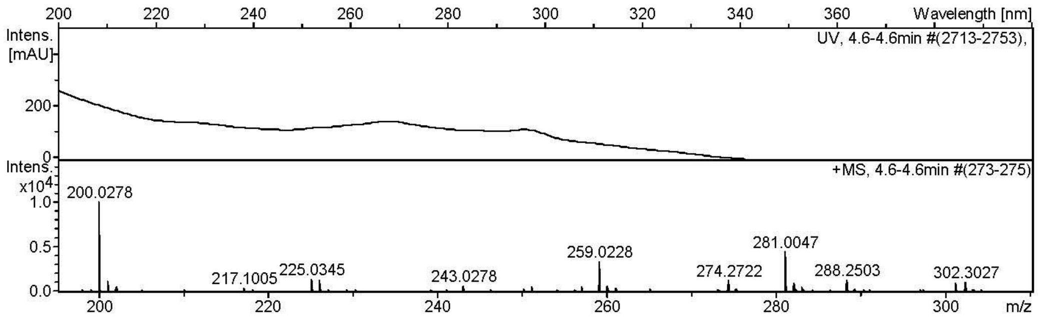

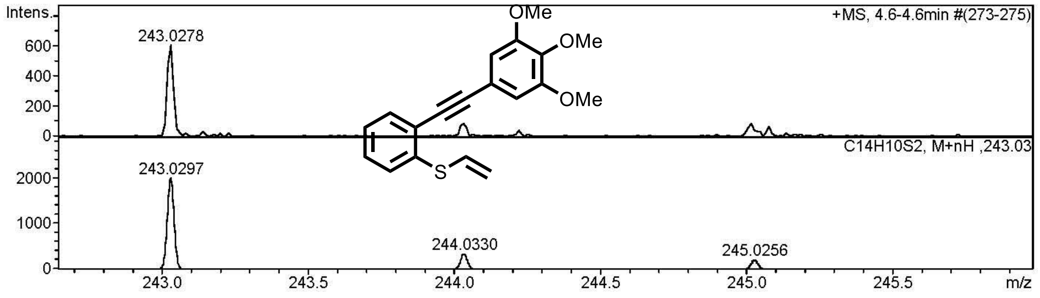

^{1}H NMR of 3-((2-(Vinylthio)phenyl)ethynyl)thiophene

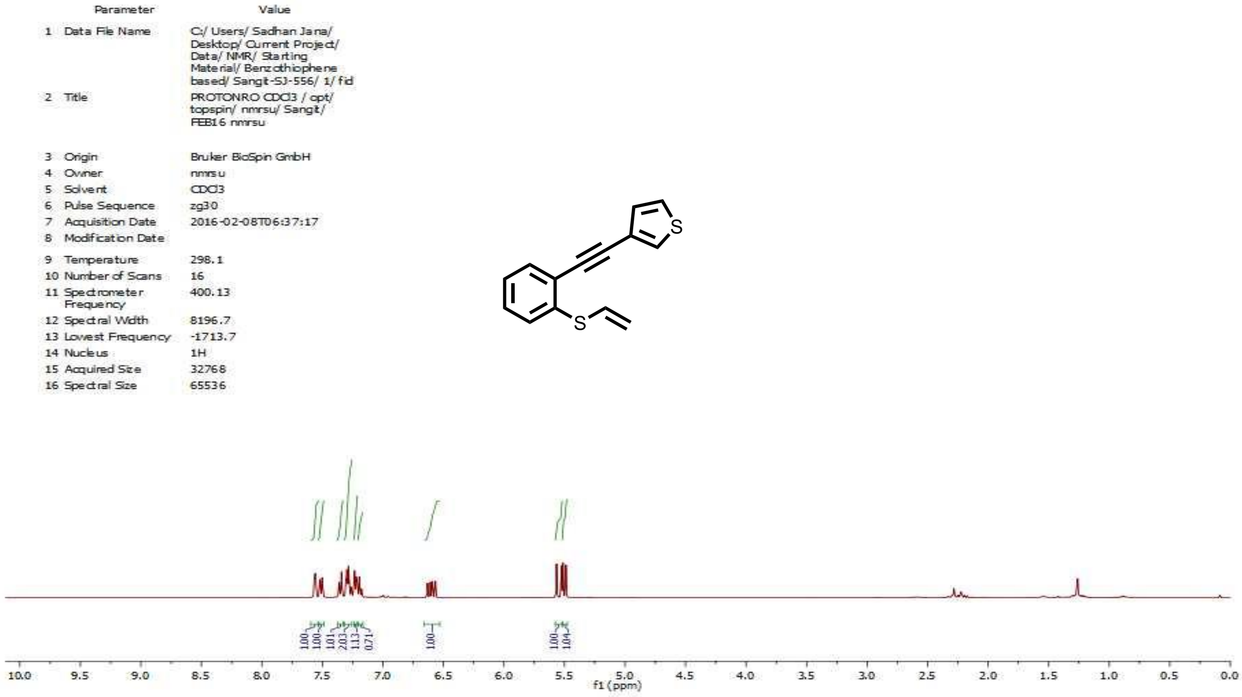

^{13}C NMR of 3-((2-(Vinylthio)phenyl)ethynyl)thiophene

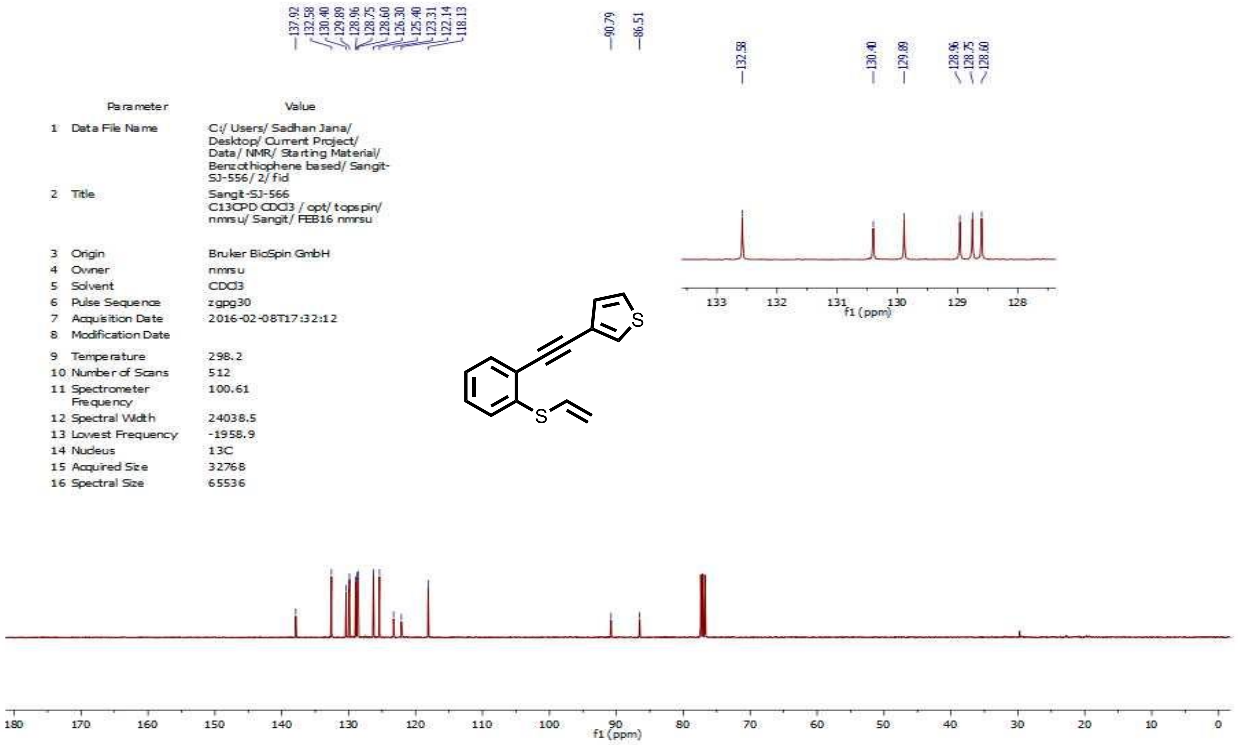

HRMS of 3-((2-(Vinylthio)phenyl)ethynyl)thiophene

Display Report

Analysis Info

Analysis Name	D:\Data\user data\2016\August 2016\10-08-2016\Dr.S.Kumar-SJ-556_1-A,5_01_7148.d	Acquisition Date	8/10/2016 1:21:34 PM
Method	hrlcms_pos_low_tunemix.m	Operator	DIMPLE
Sample Name	Dr.S.Kumar-SJ-556	Instrument	micrOTOF-Q II 10330
Comment			

Acquisition Parameter

Source Type	ESI	Ion Polarity	Positive	Set Nebulizer	1.0 Bar
Focus	Active	Set Capillary	4500 V	Set Dry Heater	250 °C
Scan Begin	50 m/z	Set End Plate Offset	-500 V	Set Dry Gas	7.0 l/min
Scan End	3000 m/z	Set Collision Cell RF	130.0 Vpp	Set Divert Valve	Waste

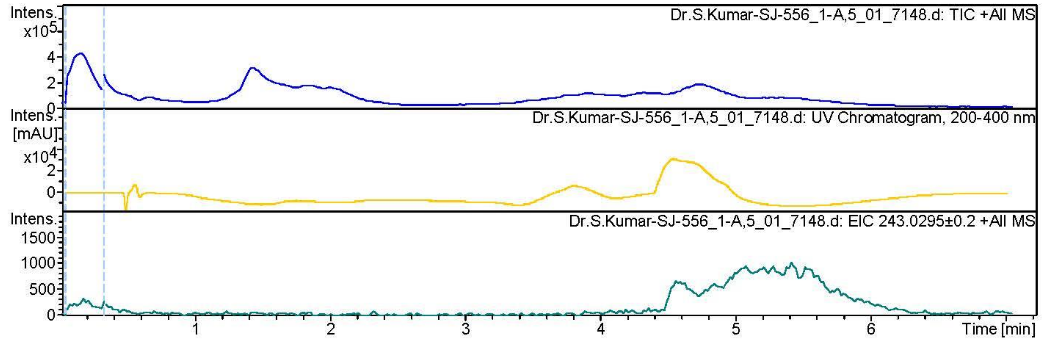

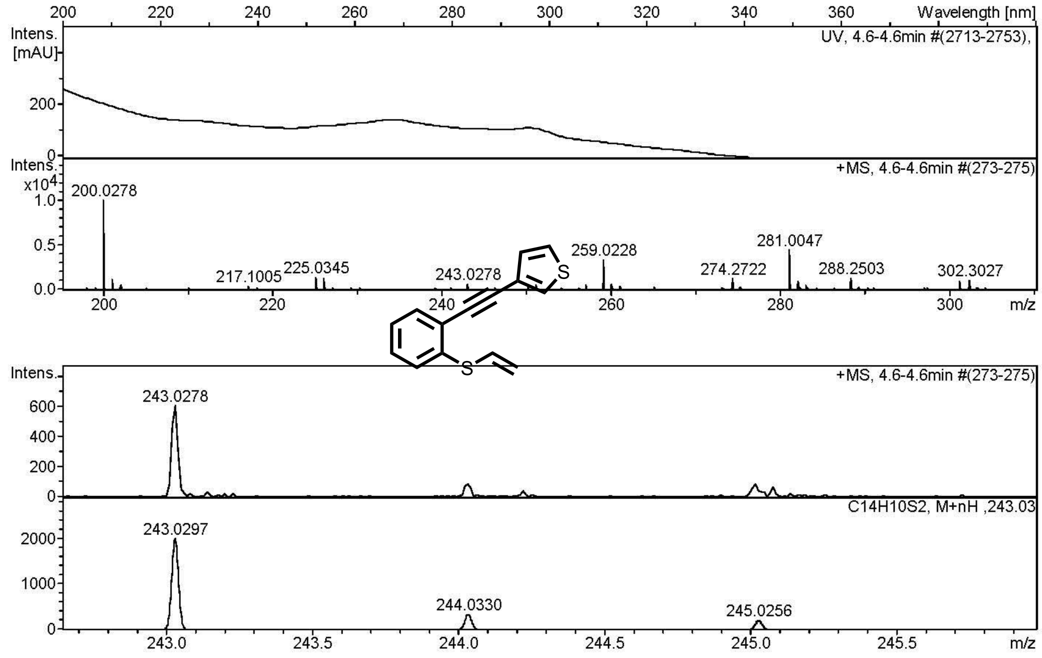

^{1}H NMR of (2-(Naphthalen-2-ylethynyl)phenyl)(vinyl)sulfane

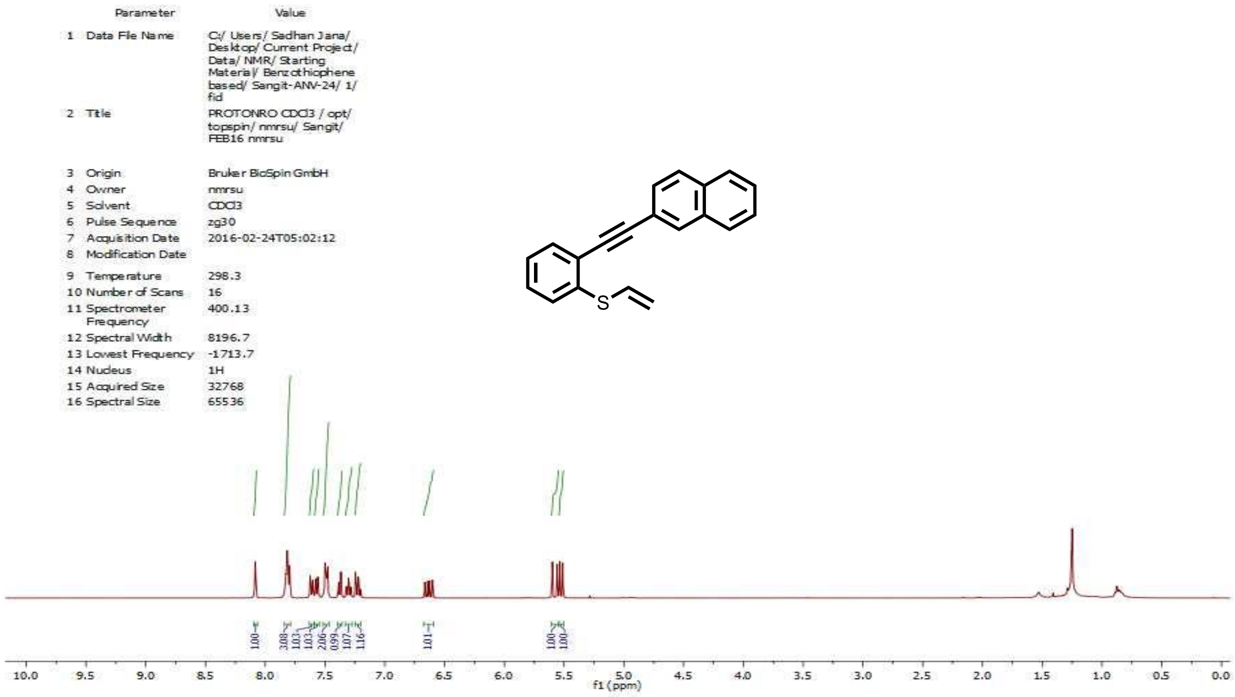

^{13}C NMR of (2-(Naphthalen-2-ylethynyl)phenyl)(vinyl)sulfane

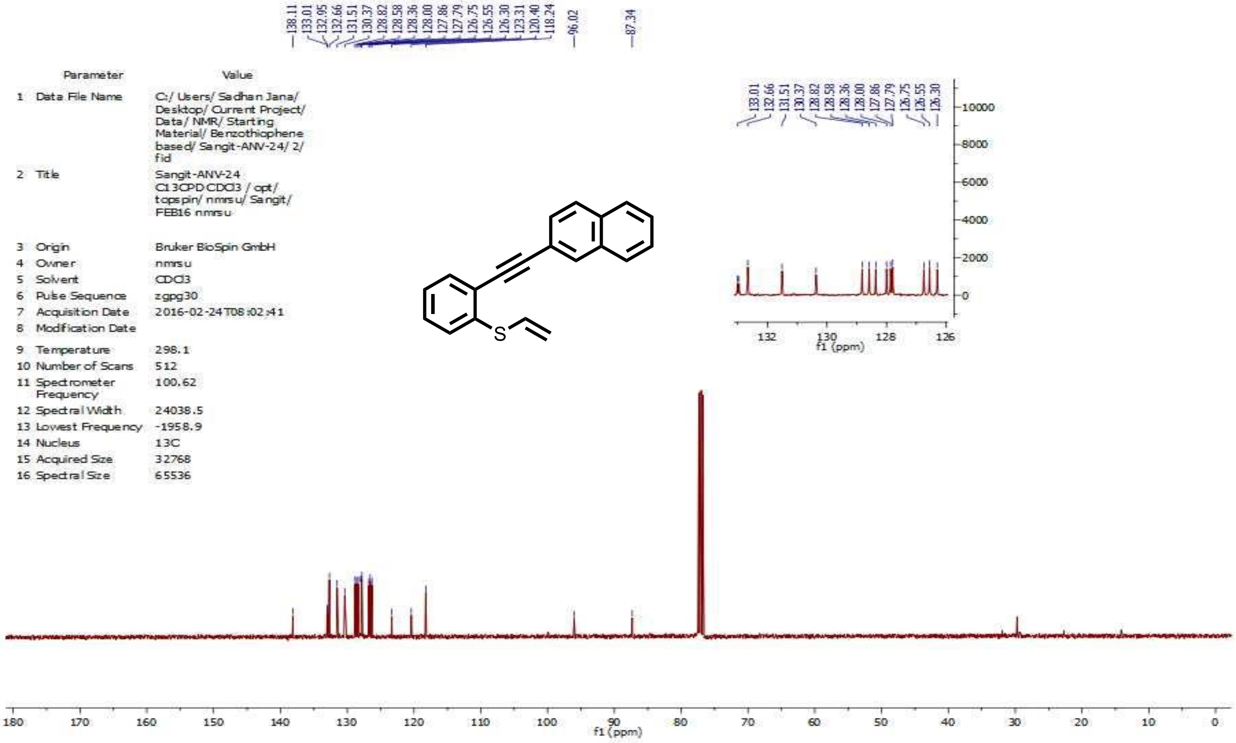

HRMS of (2-(Naphthalen-2-ylethynyl)phenyl)(vinyl)sulfane

Display Report

Analysis Info

Analysis Name	D:\Data\user data\2016\SEPT-2016\02-SEPT-2016\Dr.S.Kumar-ANV-24.d	Acquisition Date	9/2/2016 11:53:54 AM
Method	tune_low_APCI.m	Operator	DIMPLE
Sample Name	ANV-24	Instrument	micrOTOF-Q II 10330
Comment			

Acquisition Parameter

Source Type	APCI	Ion Polarity	Positive	Set Nebulizer	36.3 psi
Focus	Not active	Set Capillary	4500 V	Set Dry Heater	200 °C
Scan Begin	50 m/z	Set End Plate Offset	-500 V	Set Dry Gas	4.0 l/min
Scan End	3000 m/z	Set Collision Cell RF	130.0 Vpp	Set Divert Valve	Waste

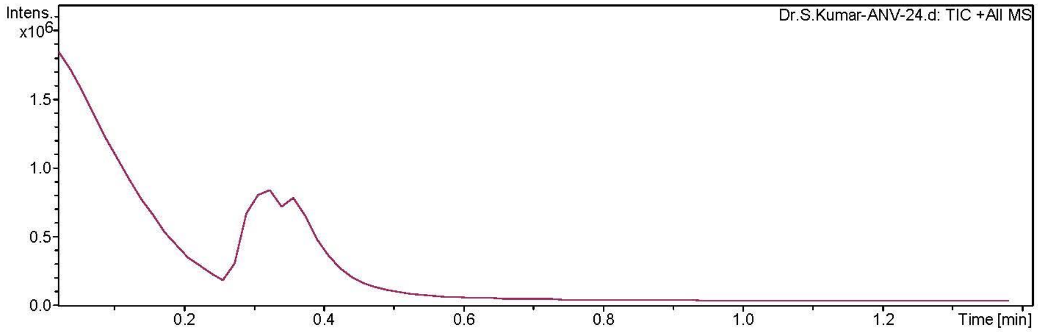

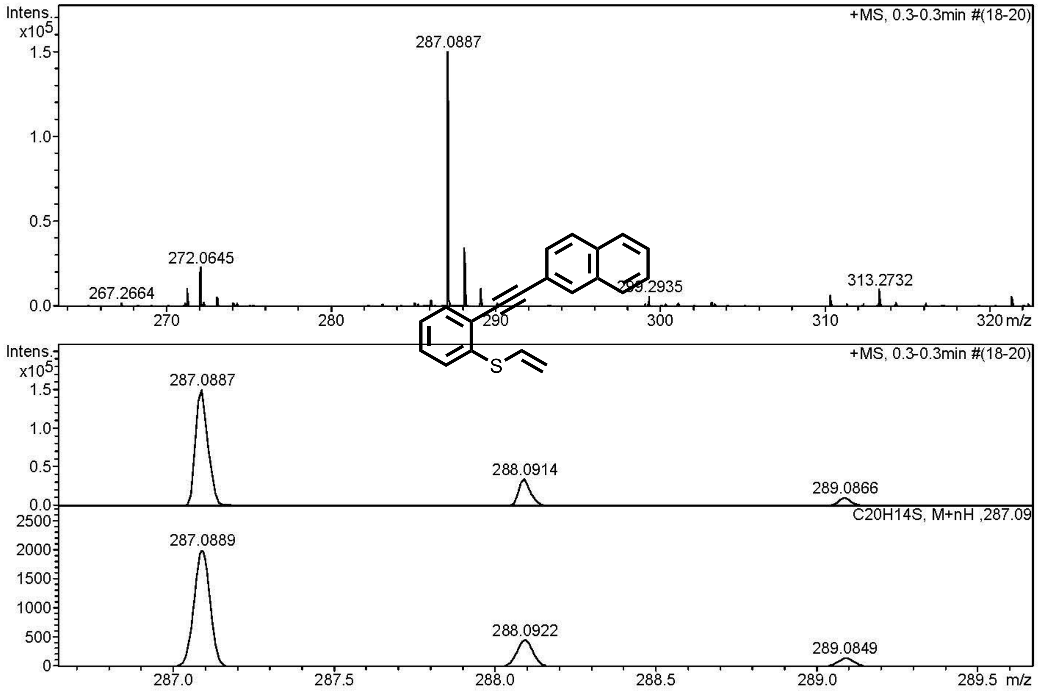

^{1}H NMR of (2-(Cyclopentylethynyl)phenyl)(vinyl)sulfane

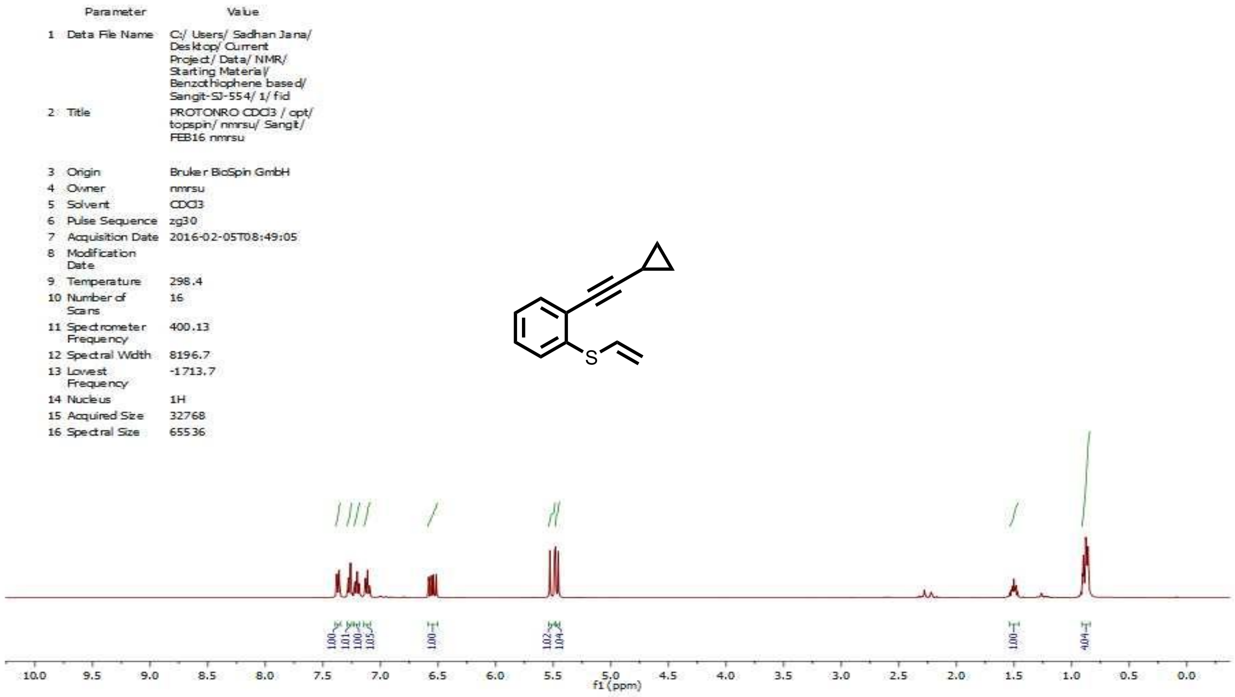

^{13}C NMR of (2-(Cyclopentylethynyl)phenyl)(vinyl)sulfane

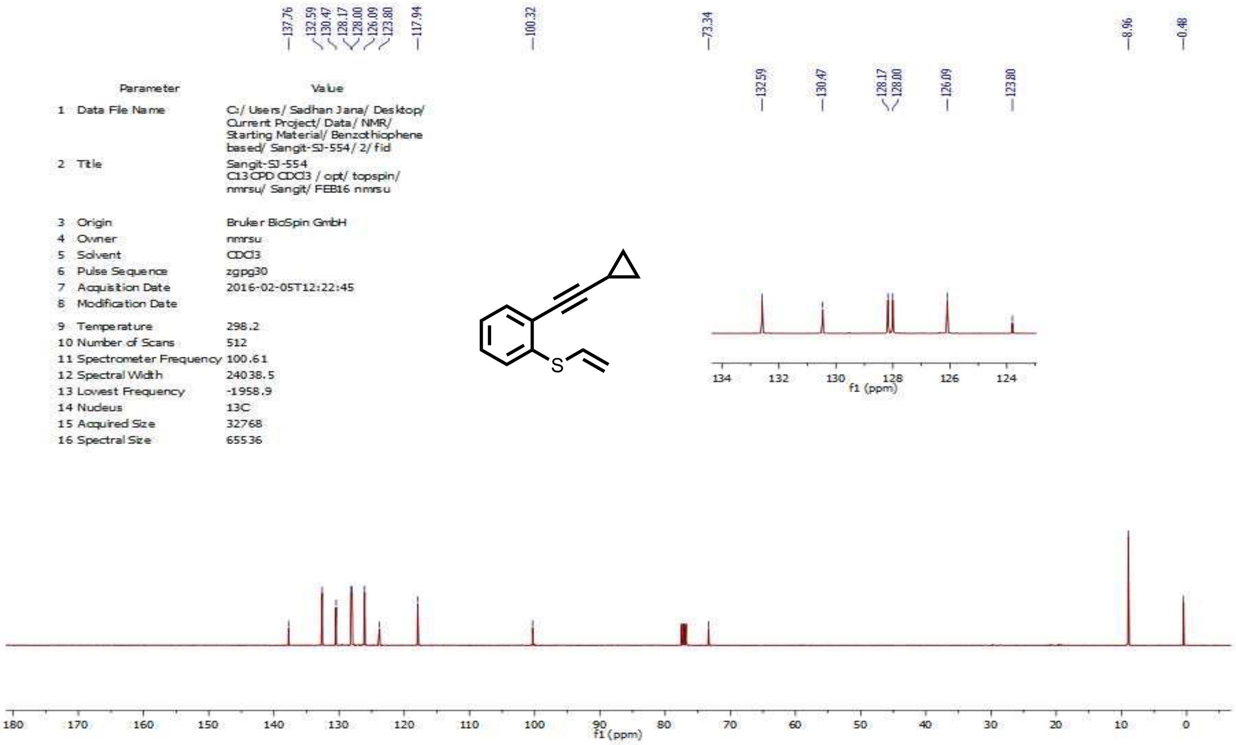

HRMS of (2-(Cyclopentylethynyl)phenyl)(vinyl)sulfane

Display Report

Analysis Info

Analysis Name	D:\Data\user data\2016\August 2016\10-08-2016\Dr.S.Kumar-SJ-554_1-A,4_01_7147.d	Acquisition Date	8/10/2016 1:13:24 PM
Method	hrlcms_pos_low_tunemix.m	Operator	DIMPLE
Sample Name	Dr.S.Kumar-SJ-554	Instrument	micrOTOF-Q II 10330
Comment			

Acquisition Parameter

Source Type	ESI	Ion Polarity	Positive	Set Nebulizer	1.0 Bar
Focus	Active	Set Capillary	4500 V	Set Dry Heater	250 °C
Scan Begin	50 m/z	Set End Plate Offset	-500 V	Set Dry Gas	7.0 l/min
Scan End	3000 m/z	Set Collision Cell RF	130.0 Vpp	Set Divert Valve	Waste

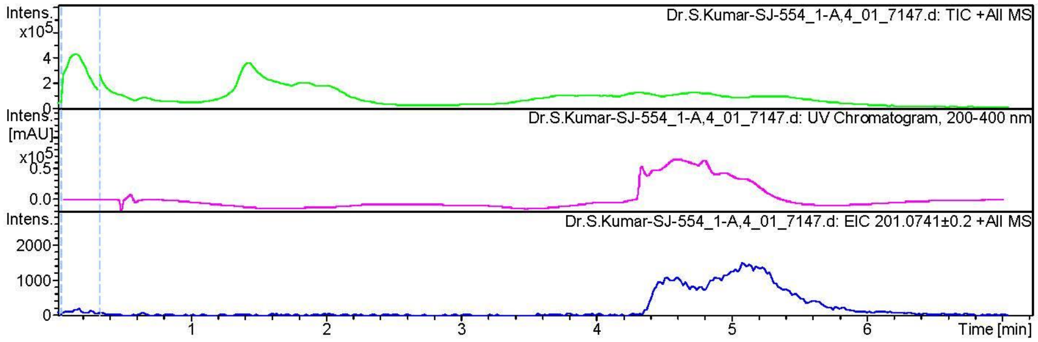

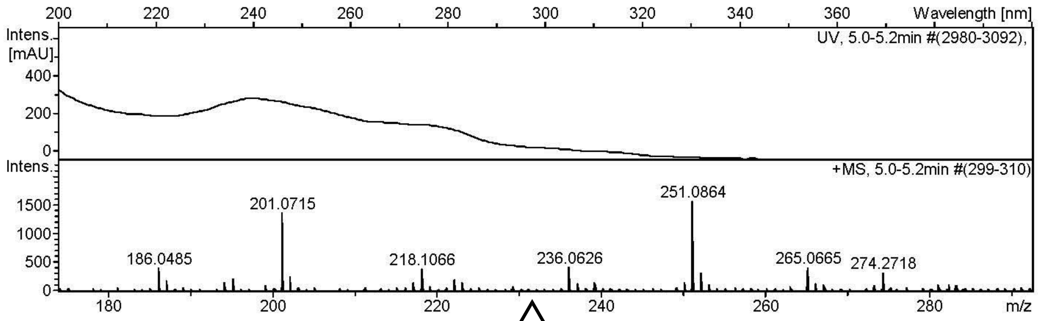

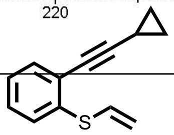

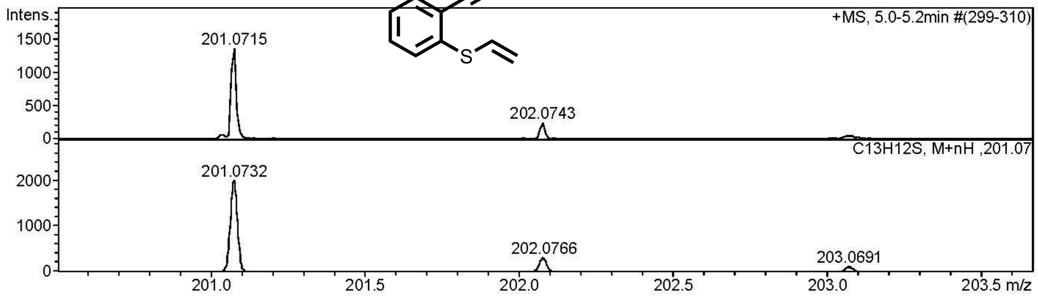

^{1}H NMR of (2-(Hex-1-yn-1-yl)phenyl)(vinyl)sulfane

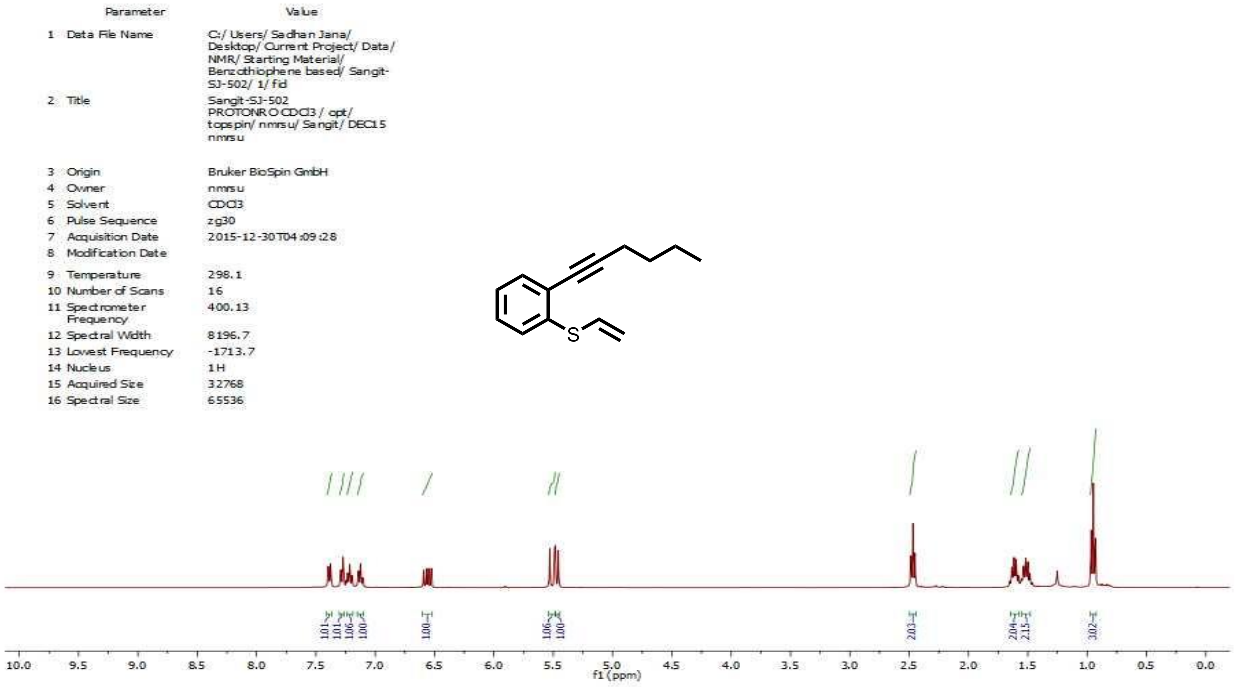

^{13}C NMR of (2-(Hex-1-yn-1-yl)phenyl)(vinyl)sulfane

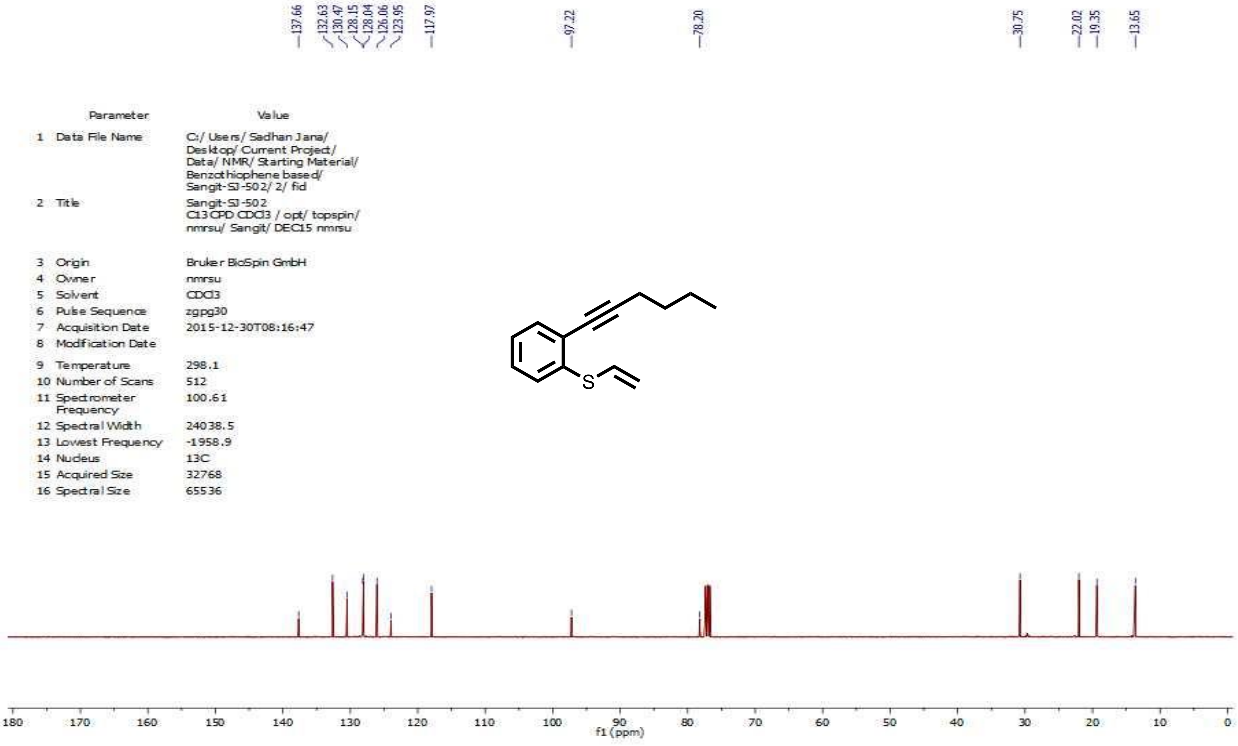

HRMS of (2-(Hex-1-yn-1-yl)phenyl)(vinyl)sulfane

Display Report

Analysis Info

Analysis Name	D:\Data\user data\2016\August 2016\05-08-2016\Dr.S.Kumar-SJ-502_1-A,5_01_7067.d	Acquisition Date	8/5/2016 12:43:34 PM
Method	hrlcms_pos_low_tunemix.m	Operator	DIMPLE
Sample Name	Dr.S.Kumar-SJ-502	Instrument	micrOTOF-Q II 10330
Comment			

Acquisition Parameter

Source Type	ESI	Ion Polarity	Positive	Set Nebulizer	1.0 Bar
Focus	Active	Set Capillary	4500 V	Set Dry Heater	250 °C
Scan Begin	50 m/z	Set End Plate Offset	-500 V	Set Dry Gas	7.0 l/min
Scan End	3000 m/z	Set Collision Cell RF	130.0 Vpp	Set Divert Valve	Waste

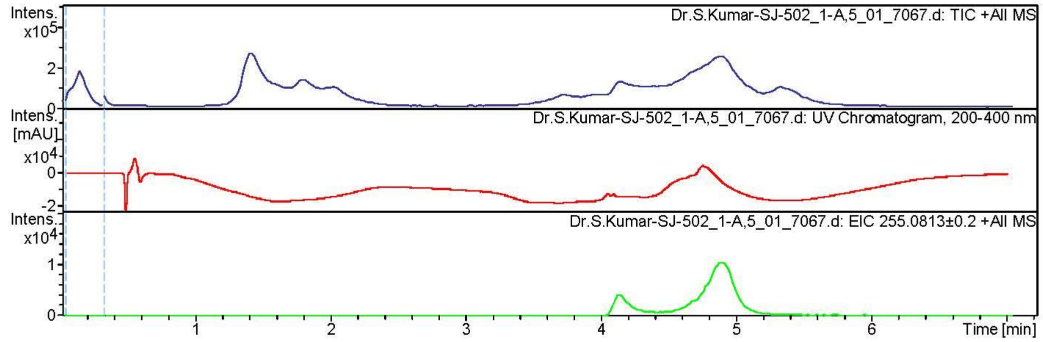

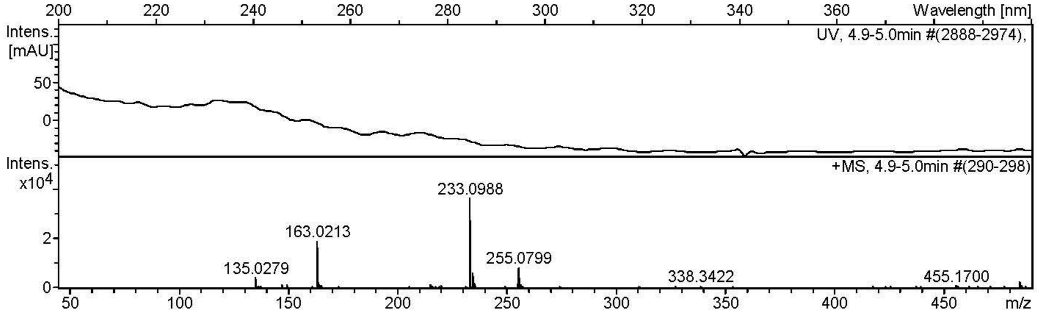

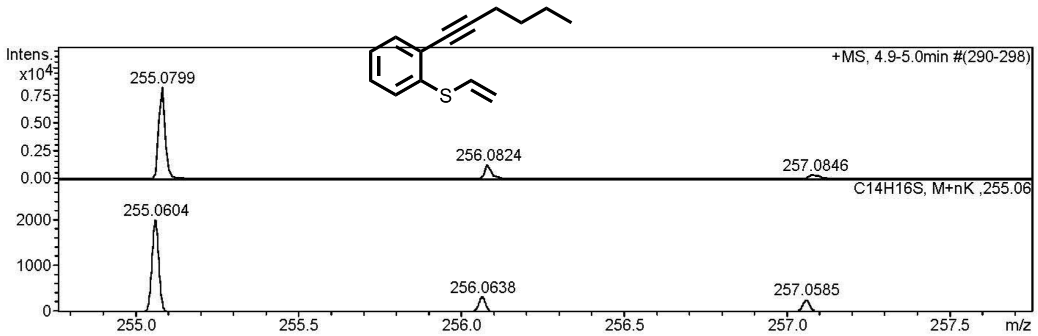

^{1}H NMR of *N*-(2-Iodophenyl)-4-methylbenzenesulfonamide

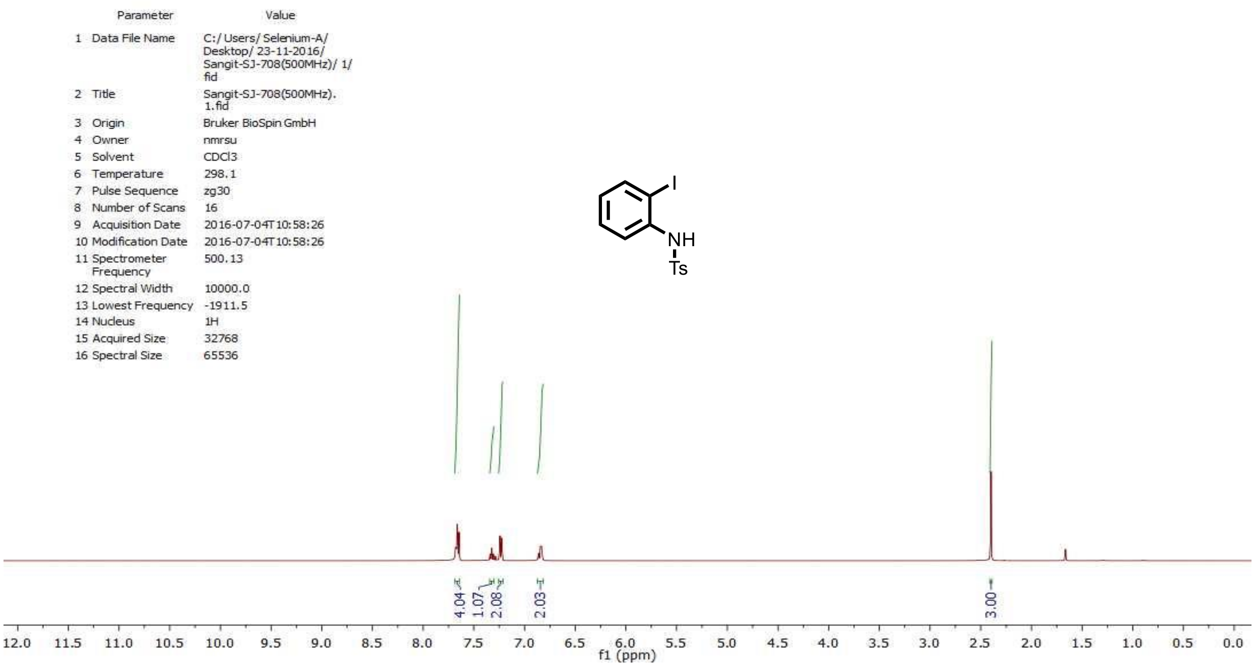

^{13}C NMR of *N*-(2-Iodophenyl)-4-methylbenzenesulfonamide

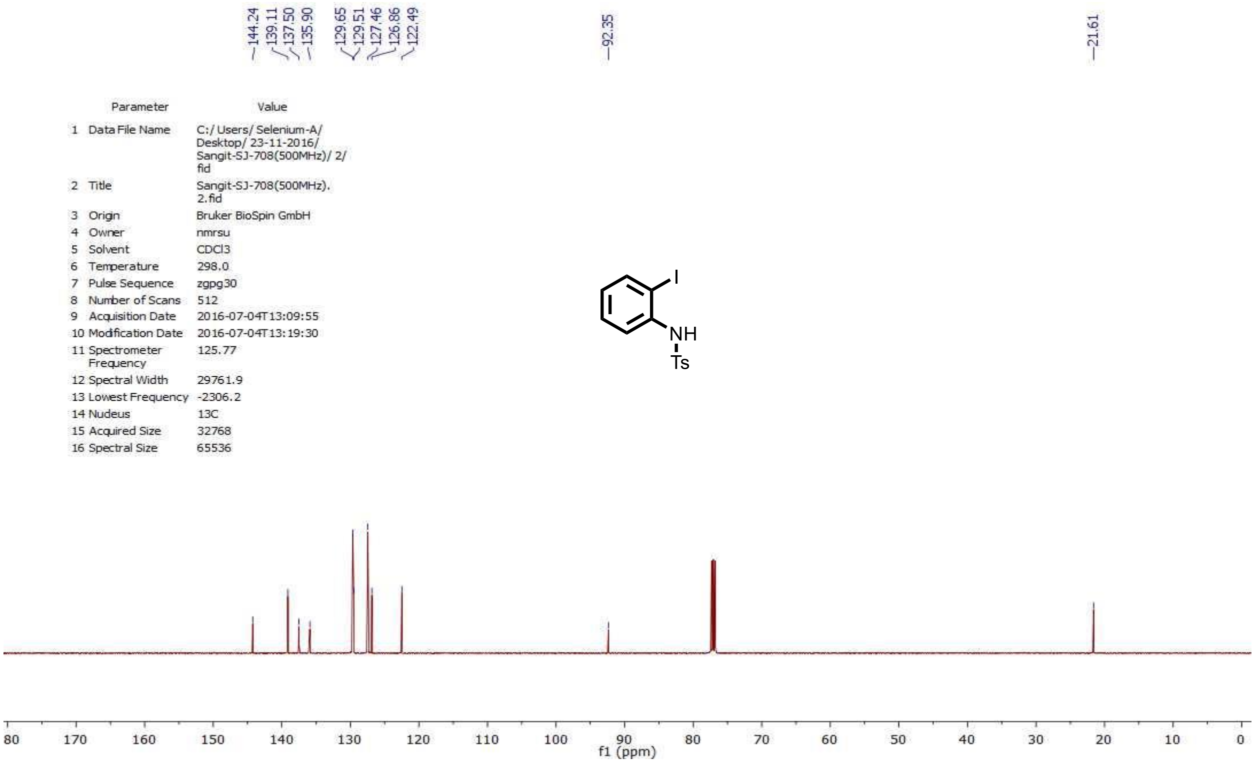

^{1}H NMR of *N*-(2-Bromoethyl)-N-(2-iodophenyl)-4-methylbenzenesulfonamide

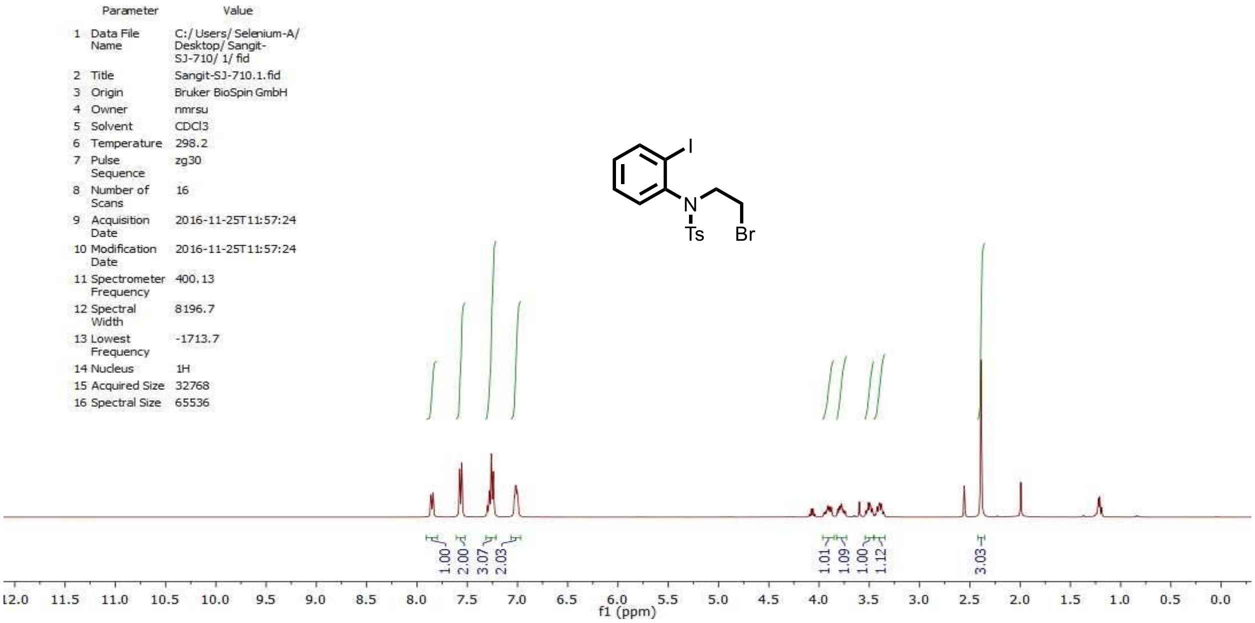

^{1}H NMR of *N*-(2-Bromoethyl)-N-(2-iodophenyl)-4-methylbenzenesulfonamide

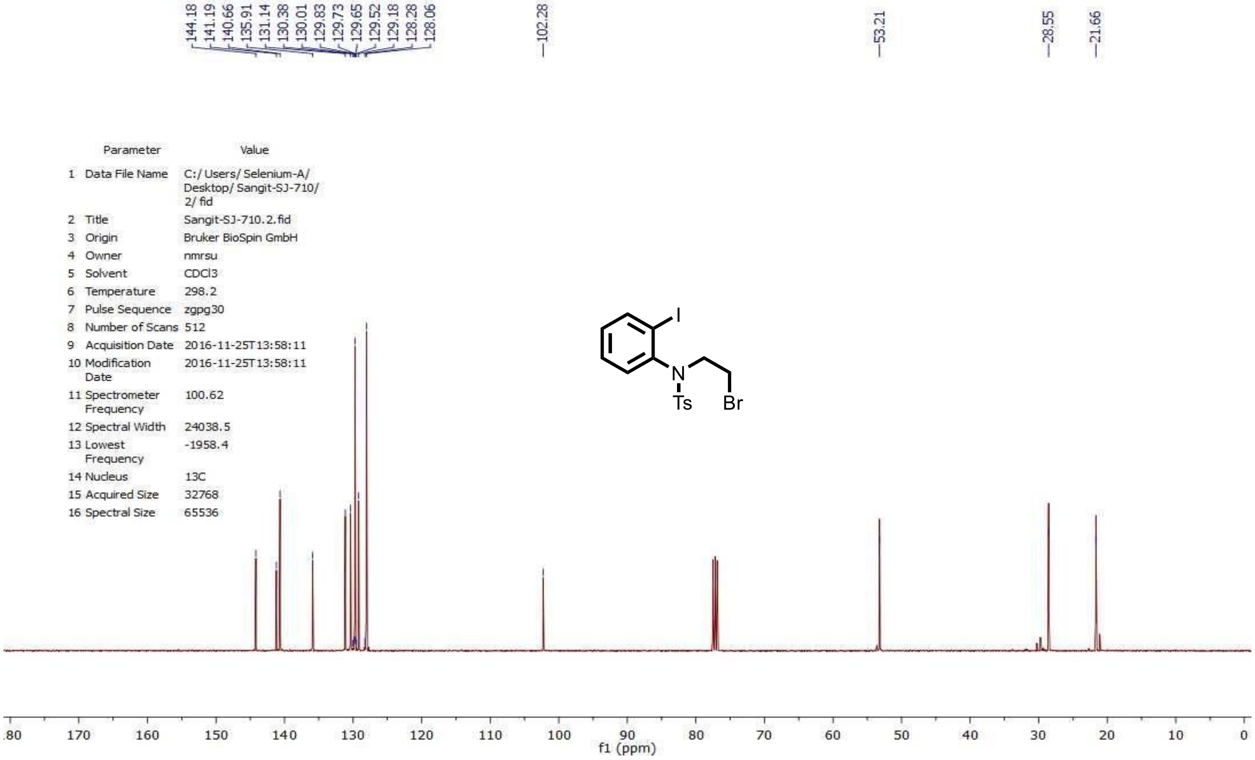

^{1}H NMR of *N*-(2-Iodophenyl)-4-methyl-N-vinylbenzenesulfonamide

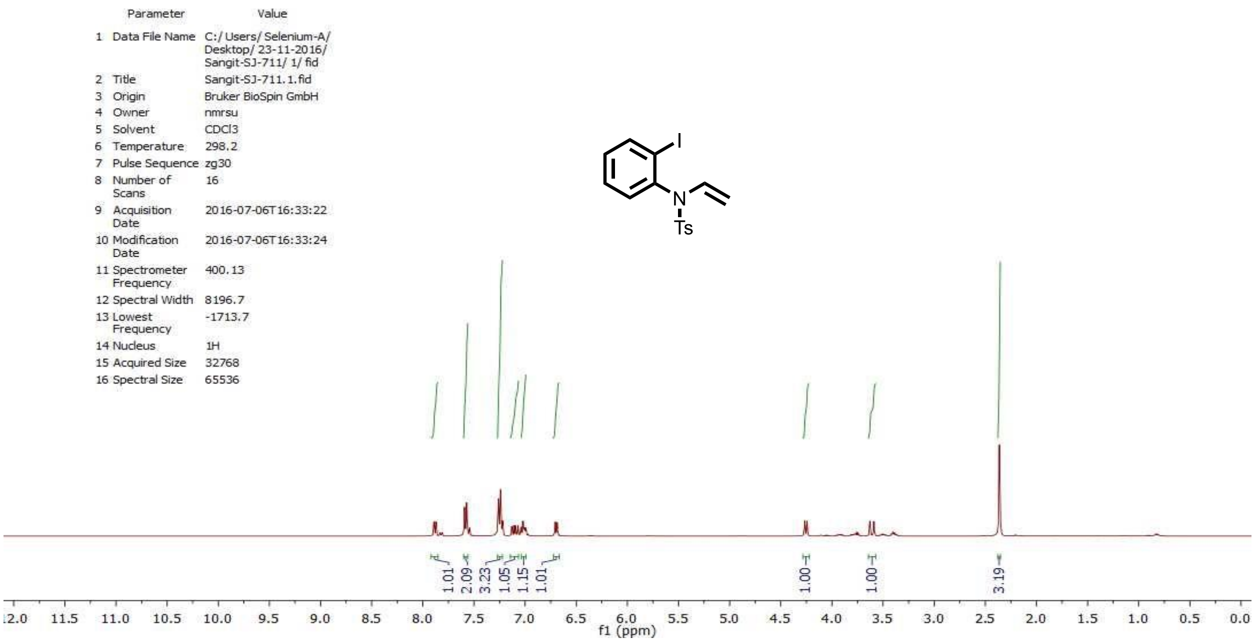

^{1}H NMR of *N*-(2-Iodophenyl)-4-methyl-*N*-vinylbenzenesulfonamide

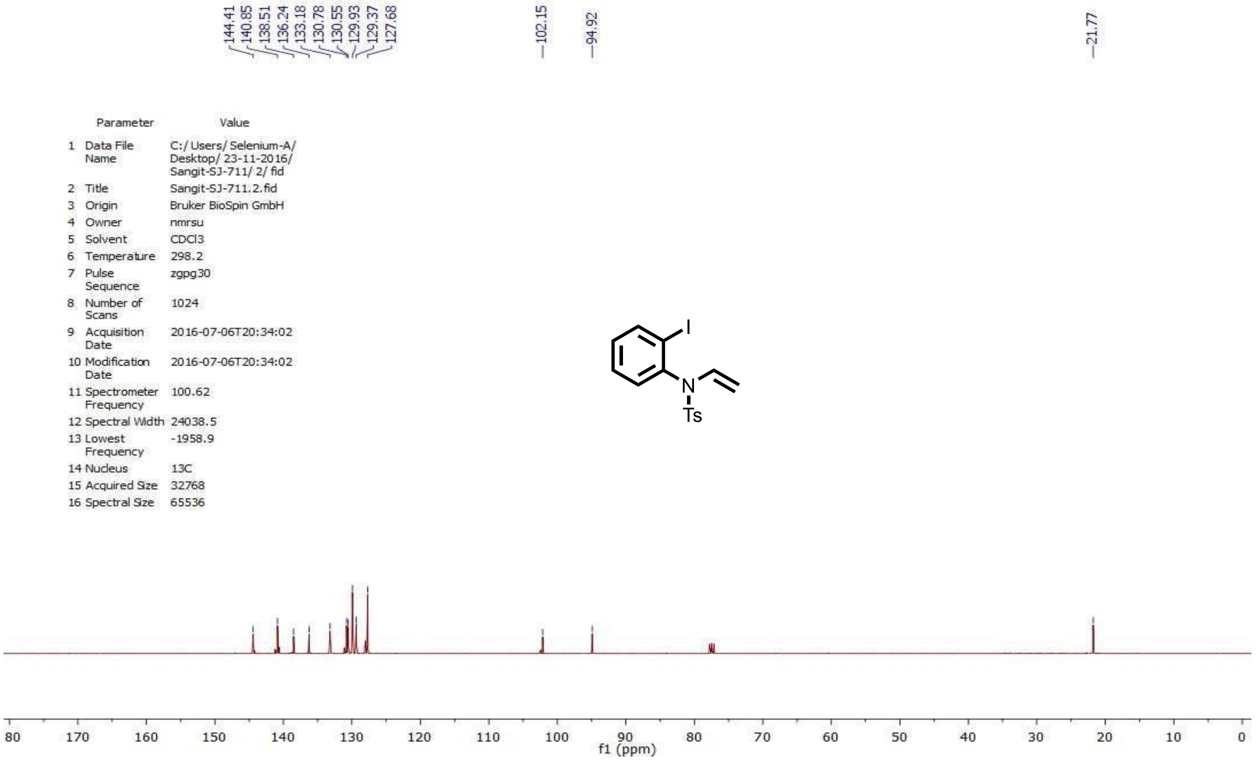

^{1}H NMR of 4-Methyl-*N*-(2-(phenylethynyl)phenyl)-*N*-vinylbenzenesulfonamide

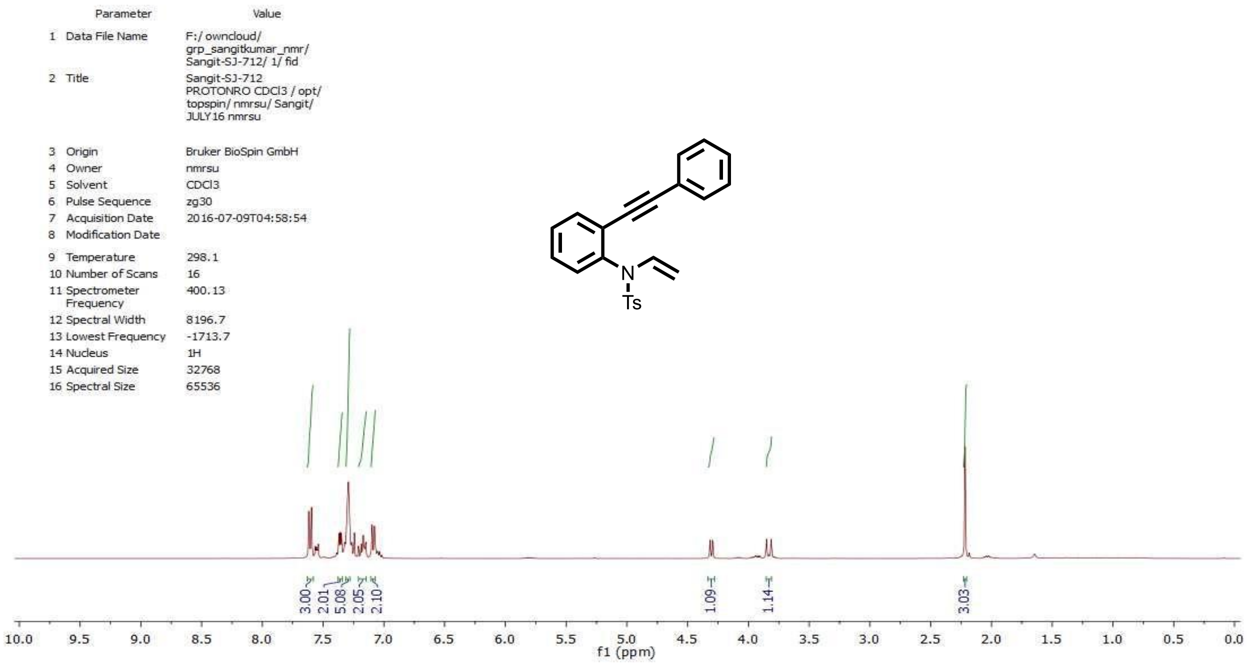

^{13}C NMR of 4-Methyl-*N*-(2-(phenylethynyl)phenyl)-*N*-vinylbenzenesulfonamide

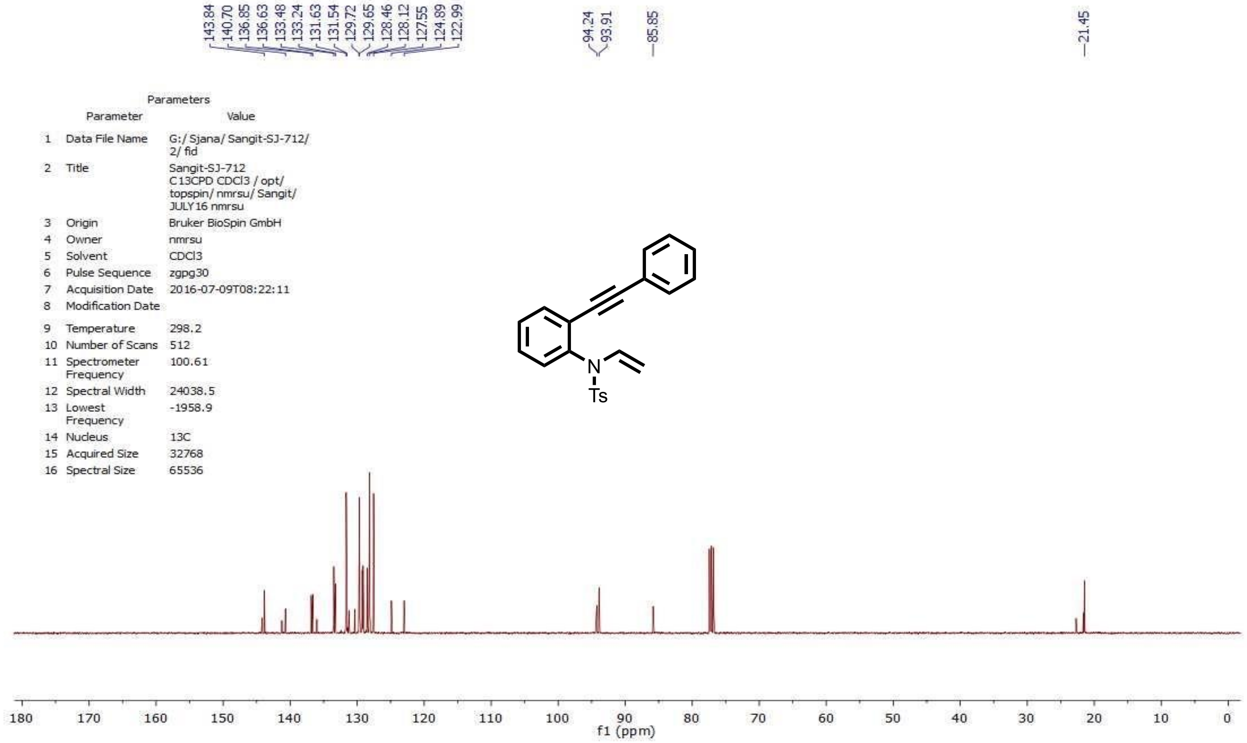

HRMS of 4-methyl-N-(2-(phenylethynyl)phenyl)-N-vinylbenzenesulfonamide

Display Report

Analysis Info

Analysis Name	D:\Data\user data\2016\August 2016\02-08-2016\Dr.S.Kumar-SJ-712_1-A,7_01_7023.d	Acquisition Date	8/2/2016 12:12:59 PM
Method	hrlcms_pos_low_tunemix.m	Operator	DIMPLE
Sample Name	Dr.S.Kumar-SJ-712	Instrument	micrOTOF-Q II 10330
Comment			

Acquisition Parameter

Source Type	ESI	Ion Polarity	Positive	Set Nebulizer	1.0 Bar
Focus	Active	Set Capillary	4500 V	Set Dry Heater	250 °C
Scan Begin	50 m/z	Set End Plate Offset	-500 V	Set Dry Gas	7.0 l/min
Scan End	3000 m/z	Set Collision Cell RF	130.0 Vpp	Set Divert Valve	Waste

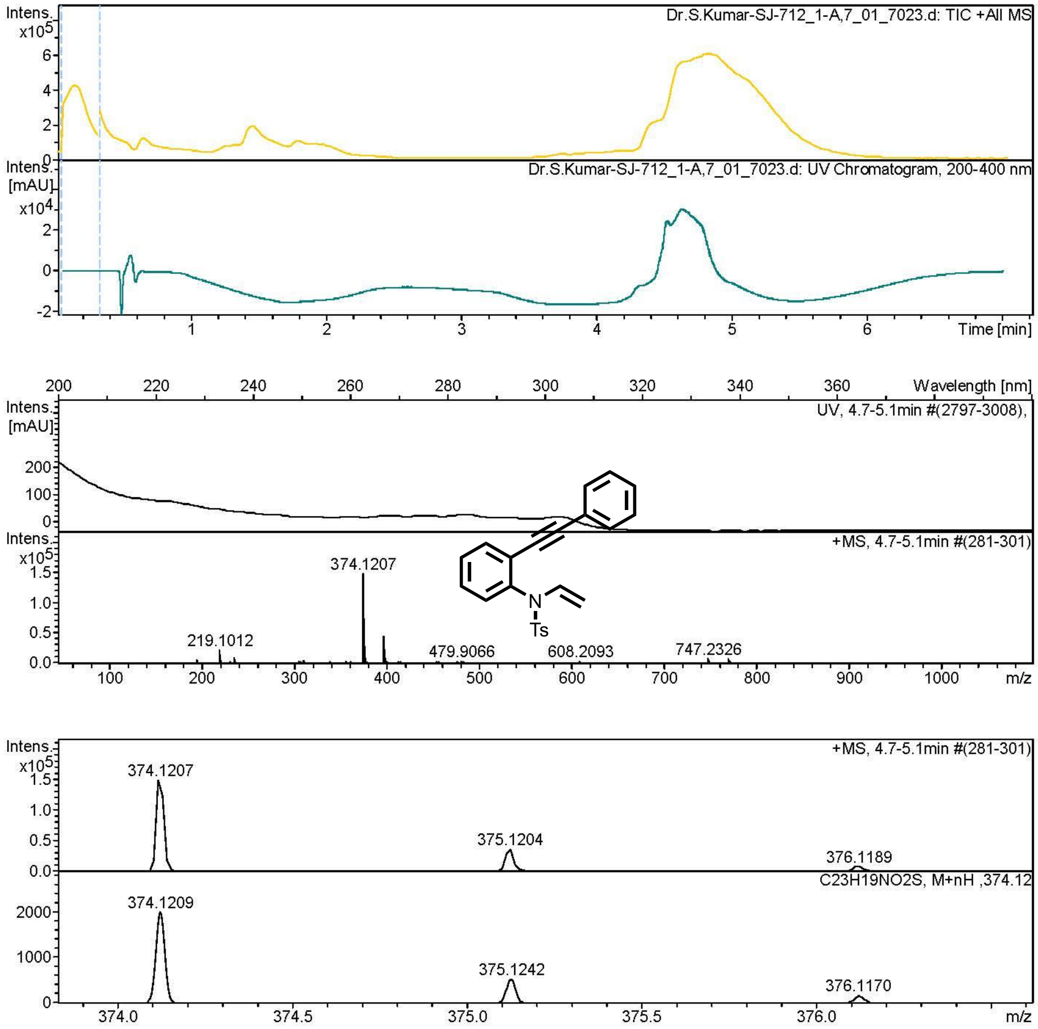

^{1}H NMR of 4-Methyl-*N*-(2-(p-tolylethynyl)phenyl)-*N*-vinylbenzenesulfonamide

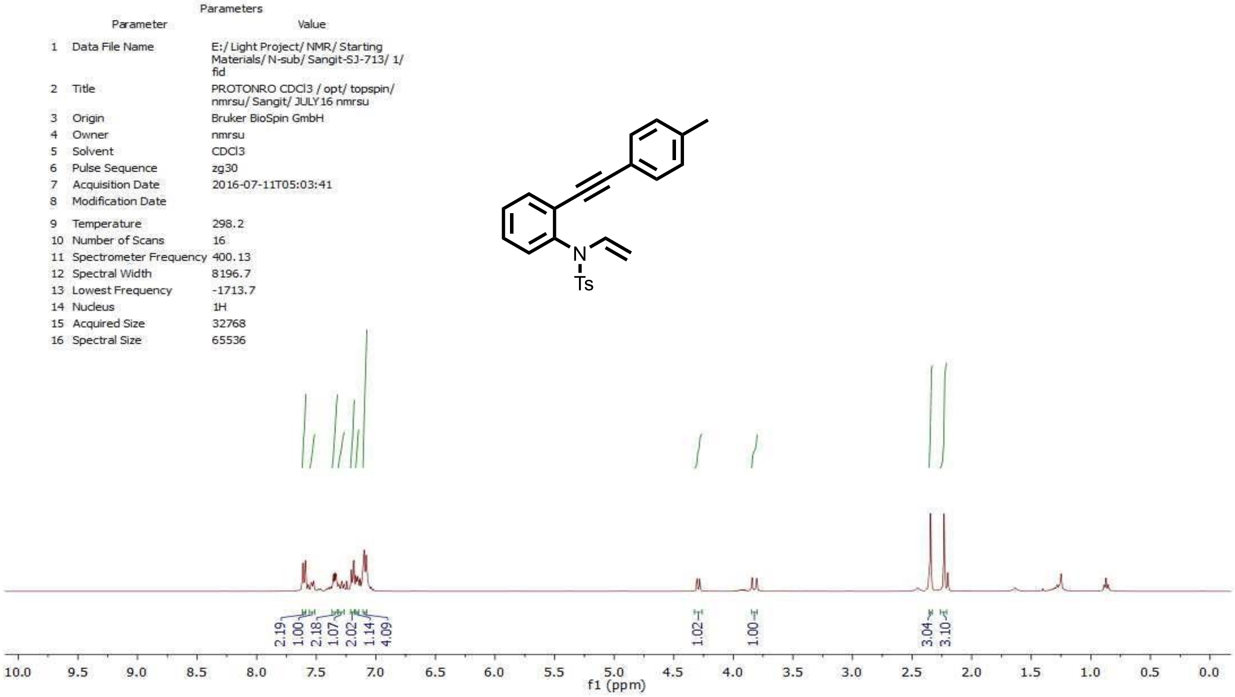

^{13}C NMR of 4-Methyl-*N*-(2-(p-tolylethynyl)phenyl)-N-vinylbenzenesulfonamide

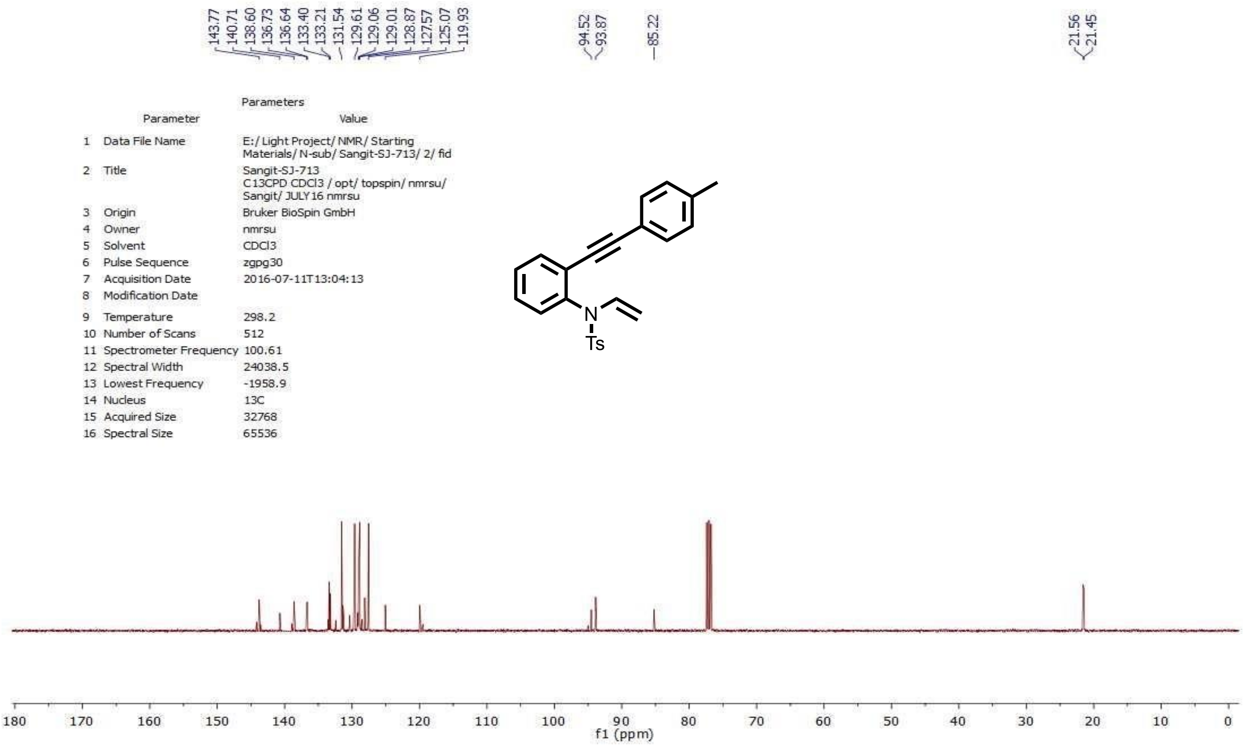

HRMS of 4-Methyl-*N*-(2-(p-tolylethynyl)phenyl)-N-vinylbenzenesulfonamide

Display Report

Analysis Info

		Acquisition Date	8/2/2016 12:33:25 PM
Analysis Name	D:\Data\user data\2016\August 2016\02-08-2016\Dr.S.Kumar-SJ-713_1-A,8_01_7025.d		
Method	hrlcms_pos_low_tunemix.m	Operator	DIMPLE
Sample Name	Dr.S.Kumar-SJ-713	Instrument	micrOTOF-Q II 10330
Comment			

Acquisition Parameter

Source Type	ESI	Ion Polarity	Positive	Set Nebulizer	1.0 Bar
Focus	Active	Set Capillary	4500 V	Set Dry Heater	250 °C
Scan Begin	50 m/z	Set End Plate Offset	-500 V	Set Dry Gas	7.0 l/min
Scan End	3000 m/z	Set Collision Cell RF	130.0 Vpp	Set Divert Valve	Waste

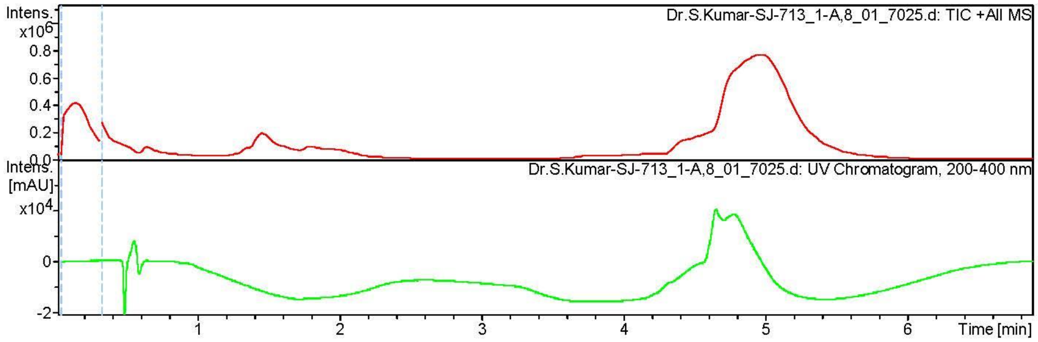

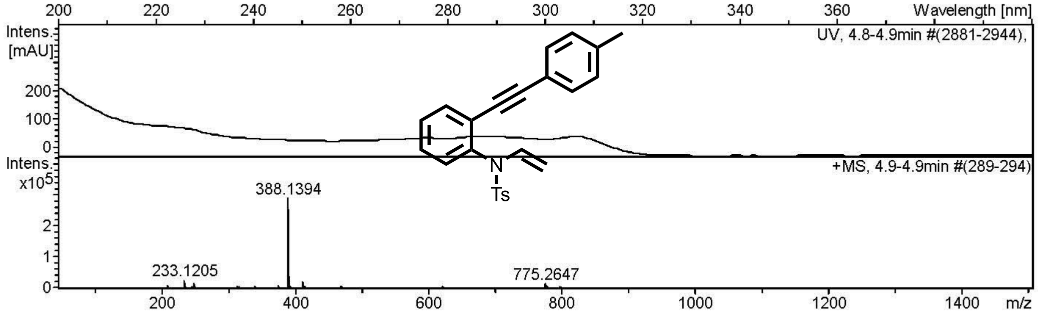

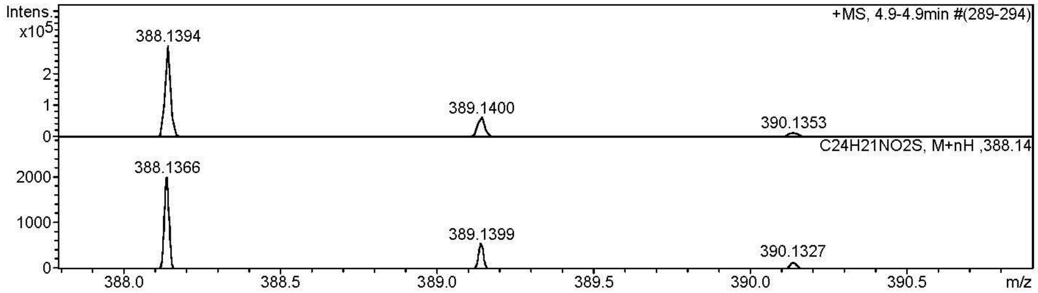

^{1}H NMR of *N*-(2-((4-Methoxyphenyl)ethynyl)phenyl)-4-methyl-*N*-vinylbenzenesulfonamide

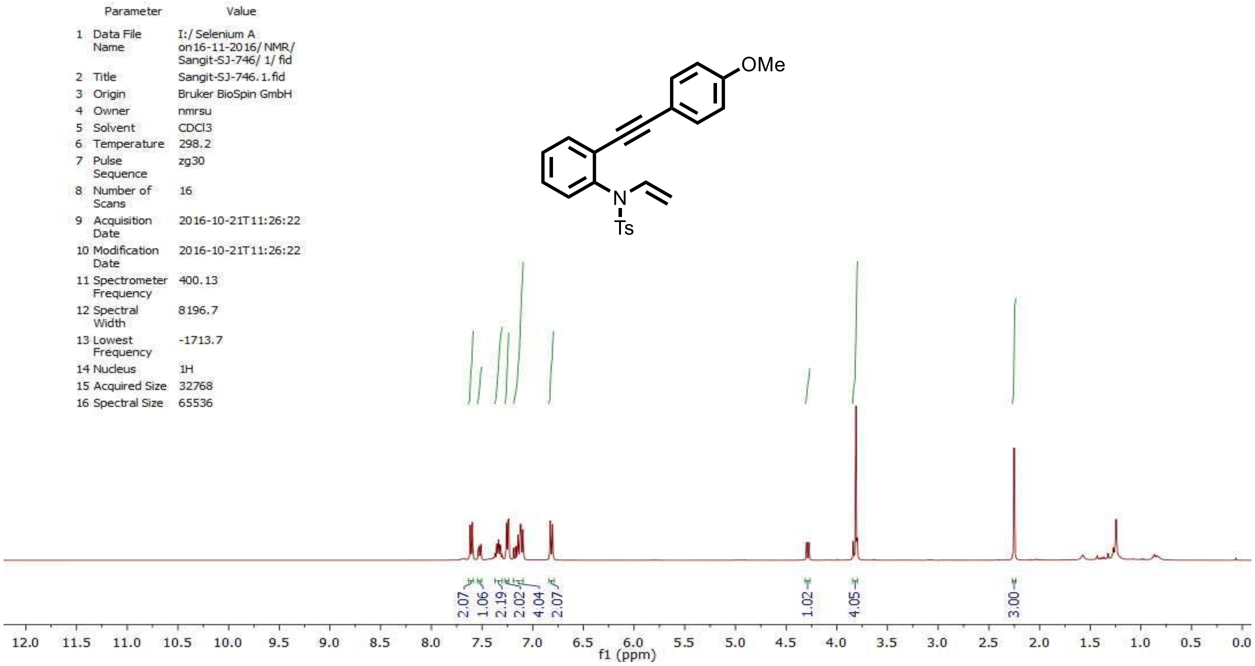

^{13}C NMR of *N*-(2-((4-Methoxyphenyl)ethynyl)phenyl)-4-methyl-*N*-vinylbenzenesulfonamide

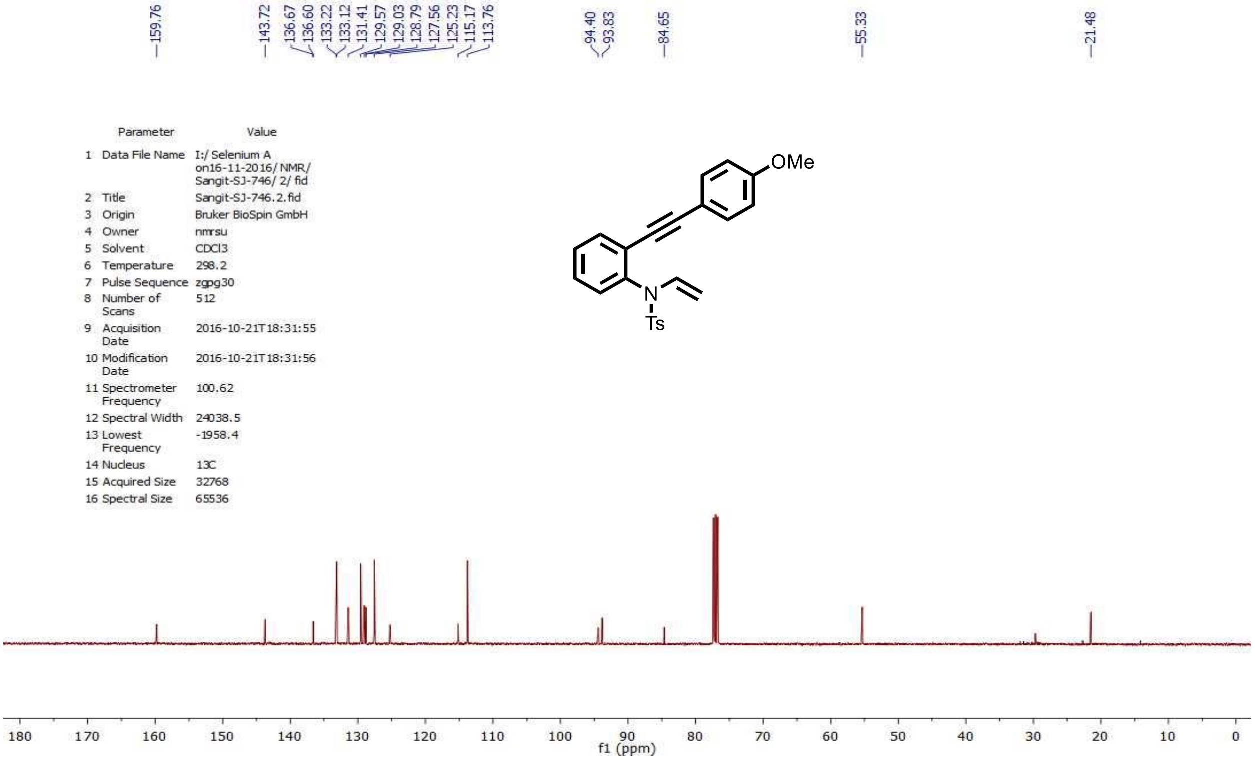

HRMS of *N*-(2-((4-Methoxyphenyl)ethynyl)phenyl)-4-methyl-*N*-vinylbenzenesulfonamide

Display Report

Analysis Info

Analysis Name	D:\Data\user data\2016\DEC-2016\07 DEC 2016\Dr.S.Kumar-SJ-746_1-A,7_01_85.d	Acquisition Date	12/7/2016 12:32:41 PM
Method	hrlcms_pos_mid_tunemix.m	Operator	RUCHI SHRIVASTAVA
Sample Name	Dr.S.Kumar-SJ-746	Instrument	micrOTOF-Q II 10330
Comment			

Acquisition Parameter

Source Type	ESI	Ion Polarity	Positive	Set Nebulizer	0.3 Bar
Focus	Active	Set Capillary	4500 V	Set Dry Heater	200 °C
Scan Begin	50 m/z	Set End Plate Offset	-500 V	Set Dry Gas	4.0 l/min
Scan End	3000 m/z	Set Collision Cell RF	450.0 Vpp	Set Divert Valve	Waste

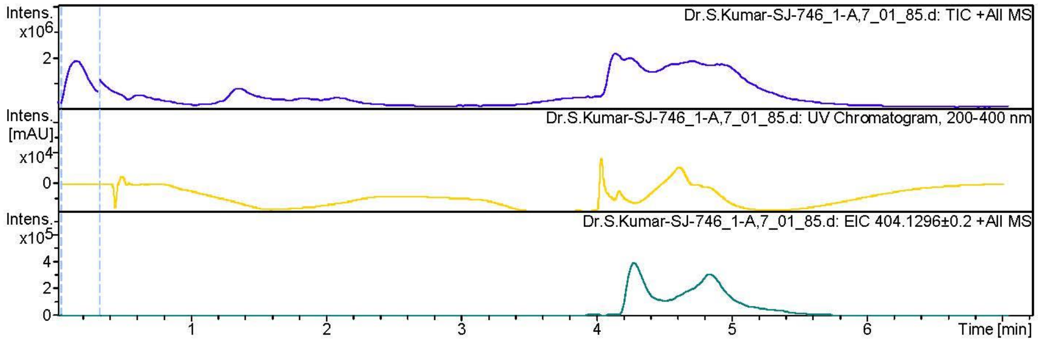

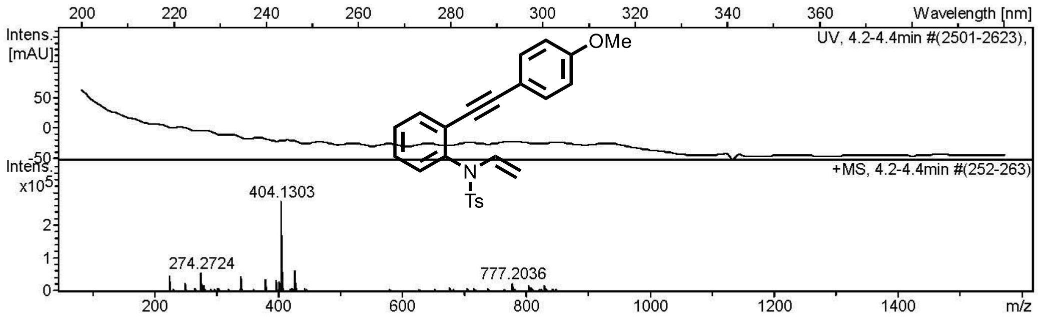

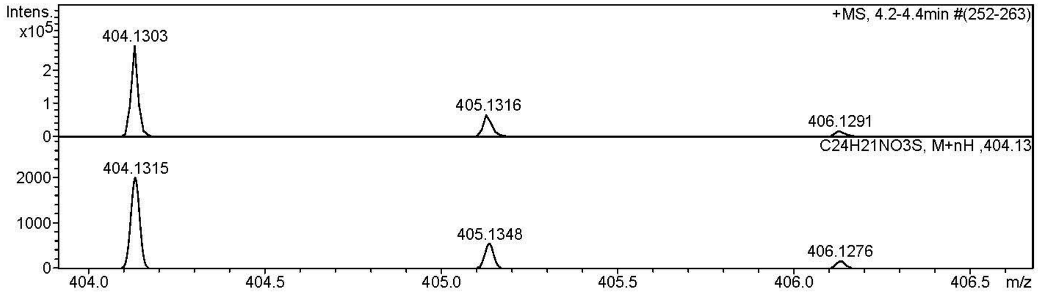

Bruker Compass DataAnalysis 4.0 printed: 12/7/2016 3:51:36 PM Page 1 of 1

^{1}H NMR of *N*-(2-((4-Bromophenyl)ethynyl)phenyl)-4-methyl-*N*-vinylbenzenesulfonamide

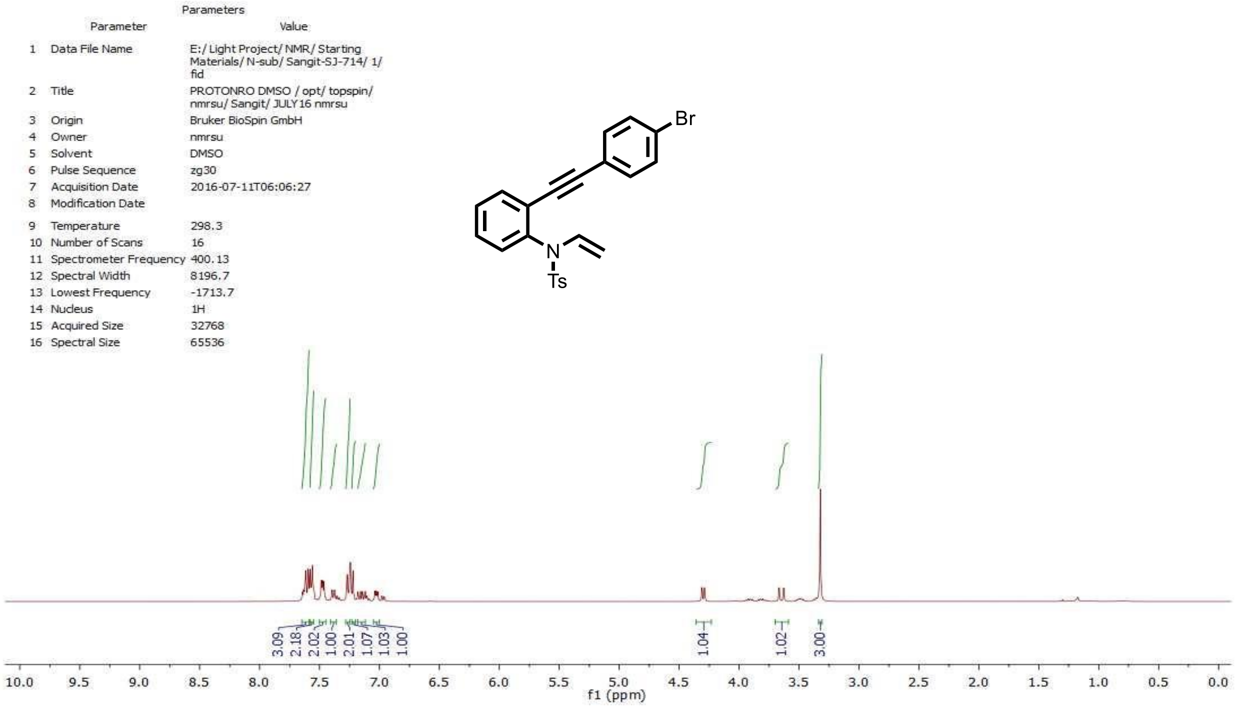

^{13}C NMR of *N*-(2-((4-Bromophenyl)ethynyl)phenyl)-4-methyl-*N*-vinylbenzenesulfonamide

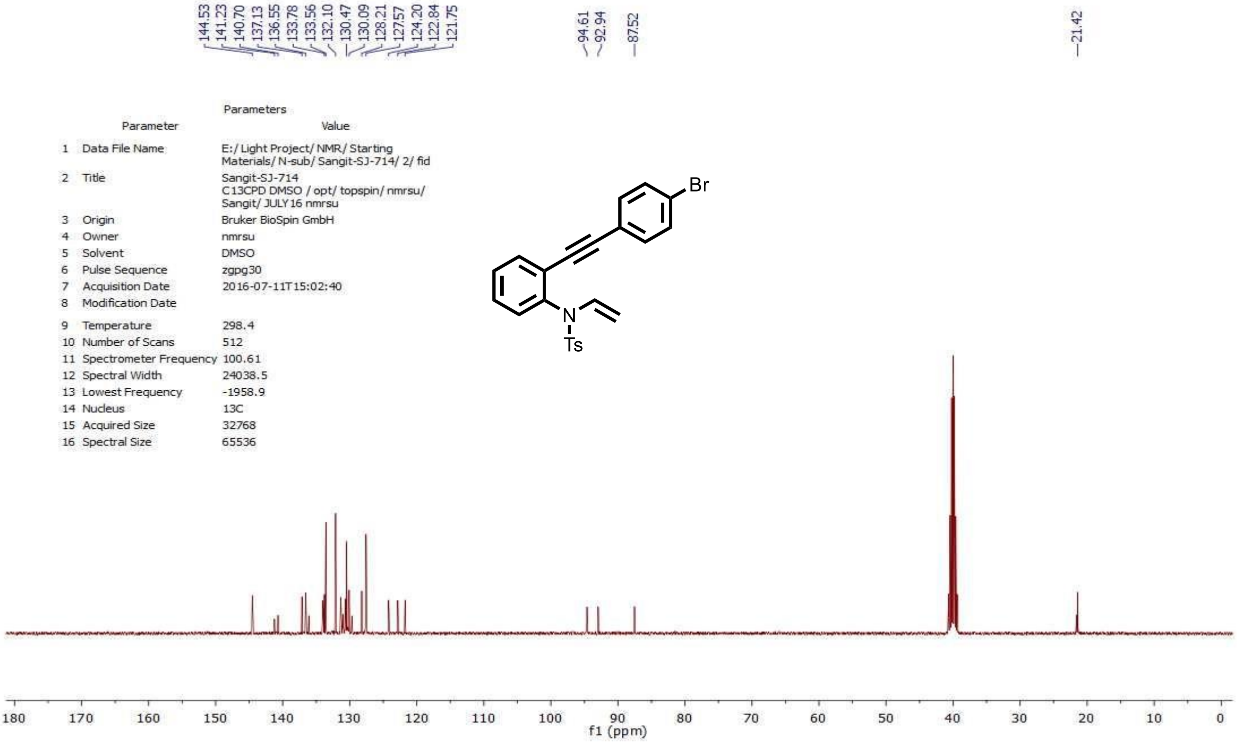

HRMS of *N*-(2-((4-Bromophenyl)ethynyl)phenyl)-4-methyl-*N*-vinylbenzenesulfonamide

Display Report

Analysis Info

Analysis Name	D:\Data\user data\2016\August 2016\02-08-2016\Dr.S.Kumar-SJ-714_1-B,1_01_7026.d	Acquisition Date	8/2/2016 12:41:47 PM
Method	hrlcms-pos_mid_tune wide.m	Operator	DIMPLE
Sample Name	Dr.S.Kumar-SJ-714	Instrument	micrOTOF-Q II 10330
Comment			

Acquisition Parameter

Source Type	ESI	Ion Polarity	Positive	Set Nebulizer	0.3 Bar
Focus	Active	Set Capillary	4500 V	Set Dry Heater	200 °C
Scan Begin	50 m/z	Set End Plate Offset	-500 V	Set Dry Gas	4.0 l/min
Scan End	3000 m/z	Set Collision Cell RF	450.0 Vpp	Set Divert Valve	Waste

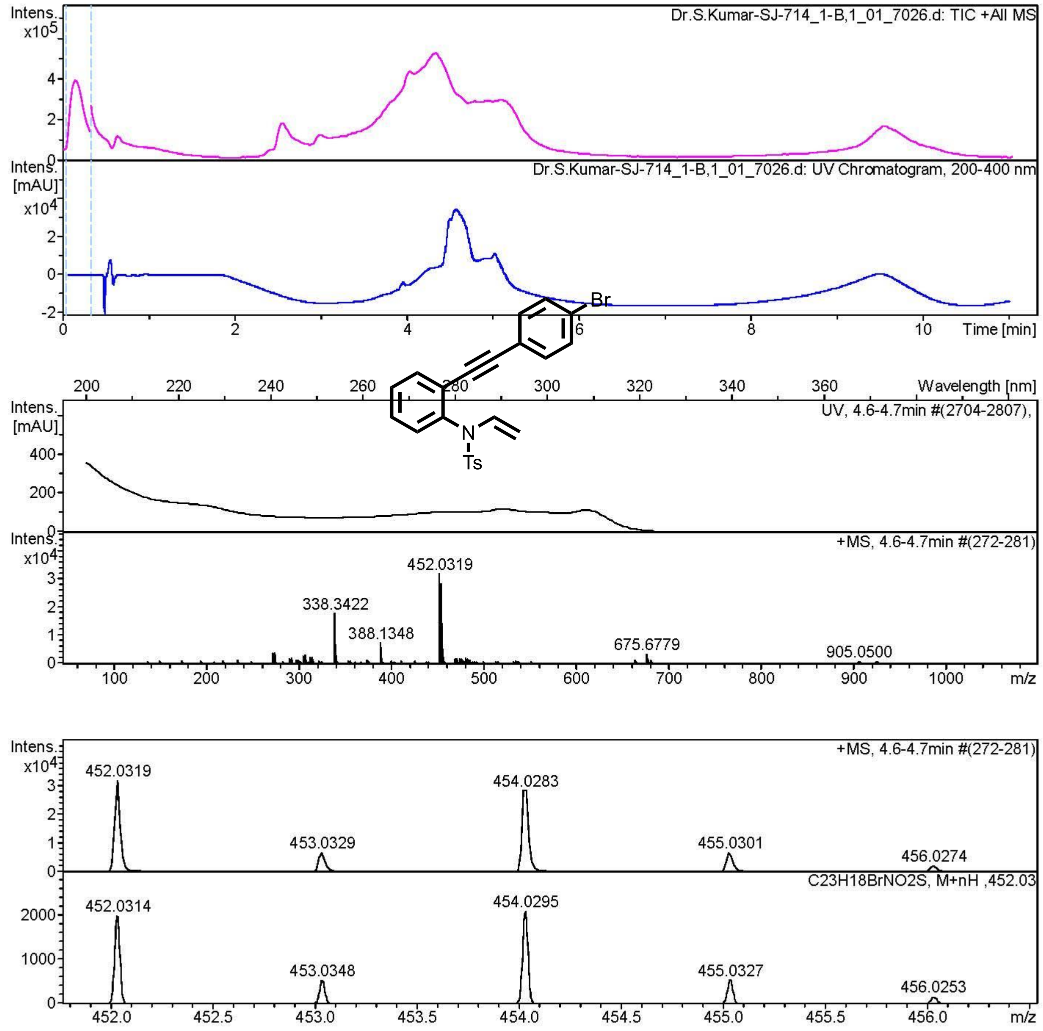

Bruker Compass DataAnalysis 4.0 printed: 8/3/2016 10:53:20 AM Page 1 of 1

^{1}H NMR of 4-Methyl-*N*-(2-(thiophen-3-ylethynyl)phenyl)-*N*-vinylbenzenesulfonamide

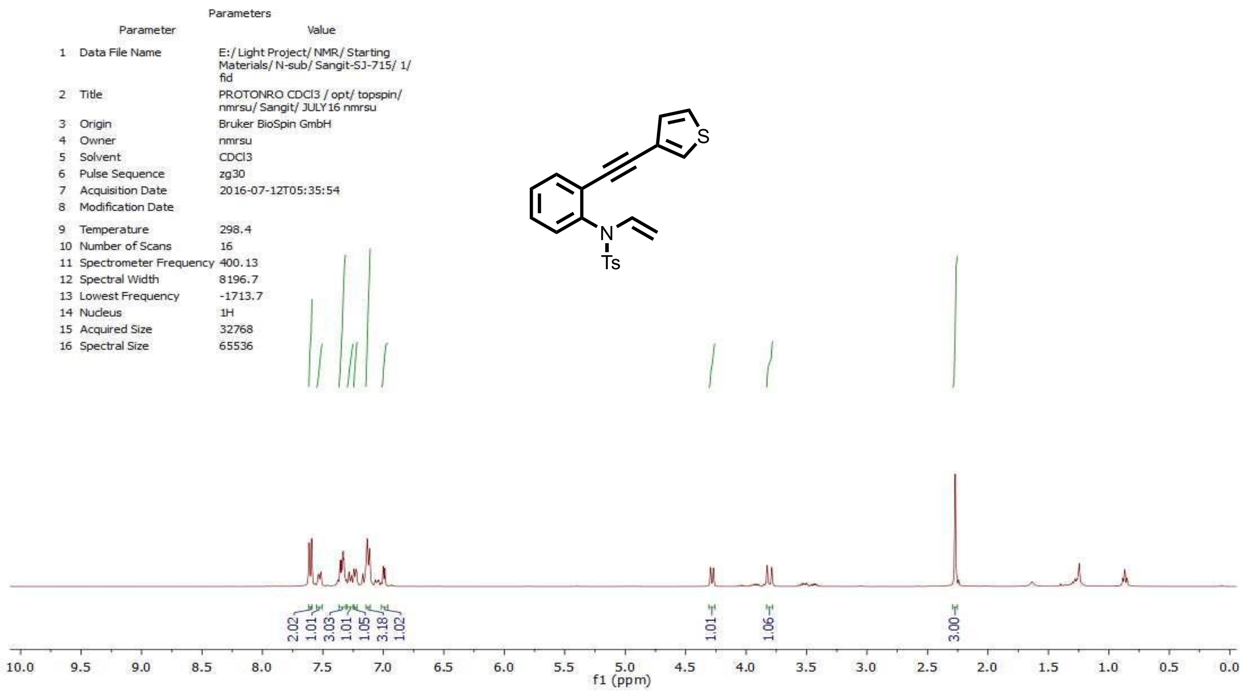

^{1}H NMR of 4-Methyl-*N*-(2-(thiophen-3-ylethynyl)phenyl)-*N*-vinylbenzenesulfonamide

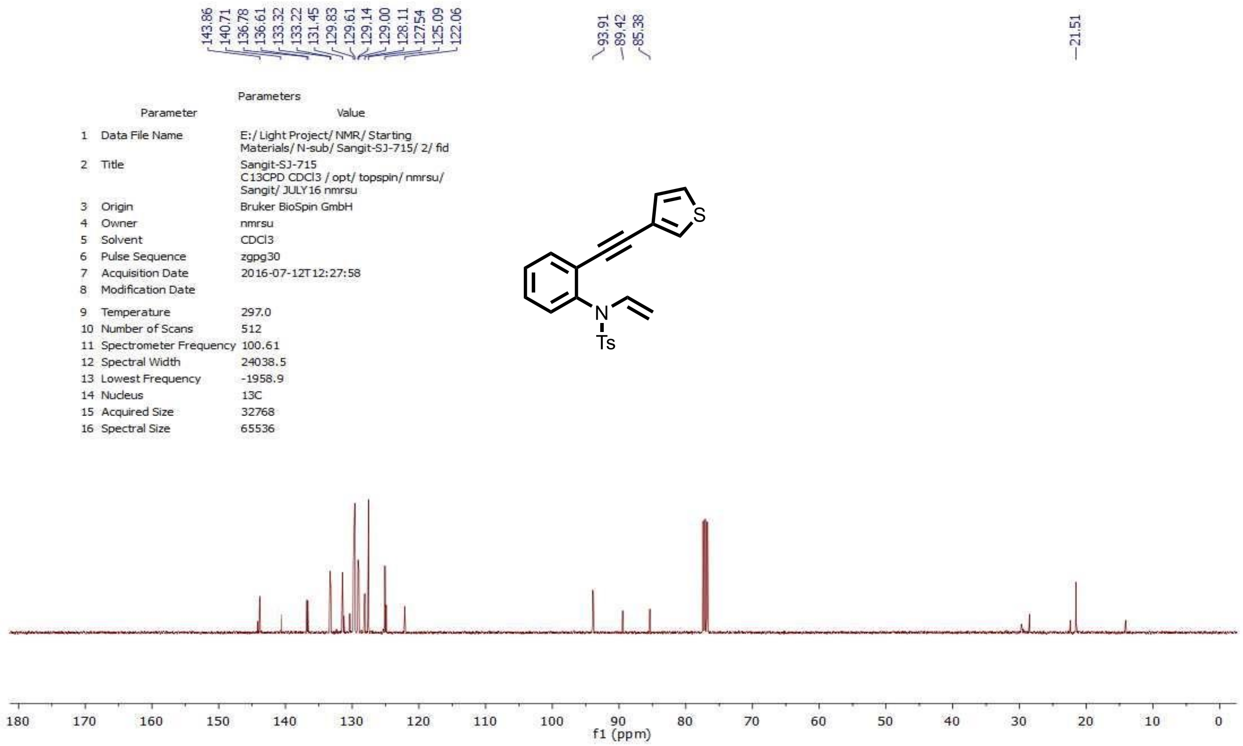

HRMS of 4-Methyl-*N*-(2-(thiophen-3-ylethynyl)phenyl)-*N*-vinylbenzenesulfonamide

Display Report

Analysis Info

		Acquisition Date	8/3/2016 1:12:30 PM
Analysis Name	D:\Data\user data\2016\August 2016\03-08-2016\Dr.S.Kumar-SJ-715_1-A,2_01_7036.d		
Method	hrlcms-pos_mid_tune wide.m	Operator	DIMPLE
Sample Name	Dr.S.Kumar-SJ-715	Instrument	micrOTOF-Q II 10330
Comment			

Acquisition Parameter

Source Type	ESI	Ion Polarity	Positive	Set Nebulizer	0.3 Bar
Focus	Active	Set Capillary	4500 V	Set Dry Heater	200 °C
Scan Begin	50 m/z	Set End Plate Offset	-500 V	Set Dry Gas	4.0 l/min
Scan End	3000 m/z	Set Collision Cell RF	450.0 Vpp	Set Divert Valve	Waste

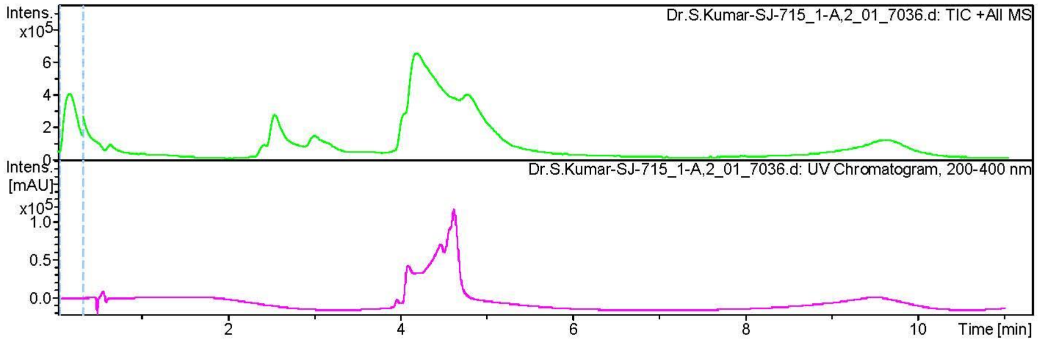

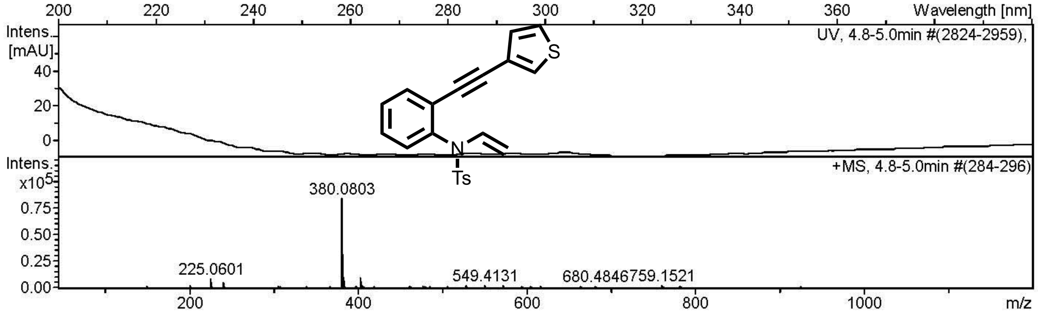

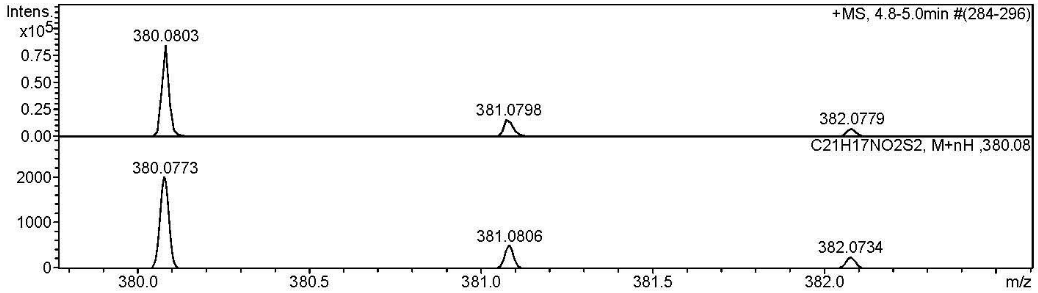

^{1}H NMR of 4-Methyl-*N*-(2-(naphthalen-1-ylethynyl)phenyl)-*N*-vinylbenzenesulfonamide

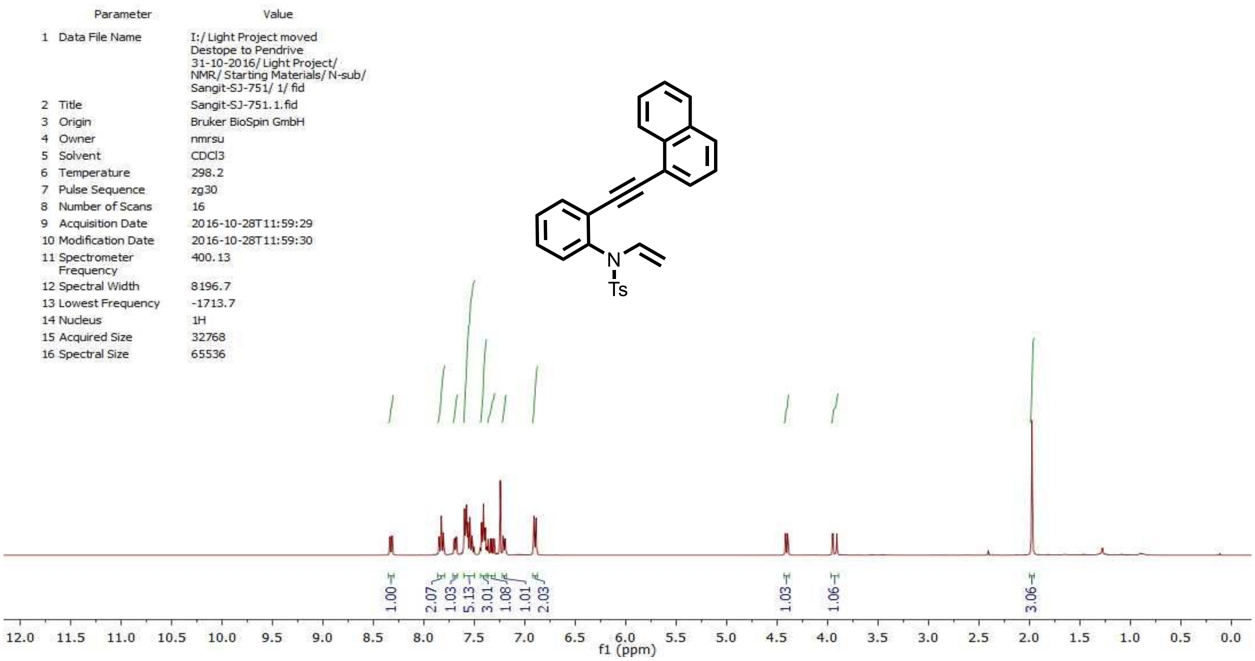

^{13}C NMR of 4-Methyl-N-(2-(naphthalen-1-ylethynyl)phenyl)-N-vinylbenzenesulfonamide

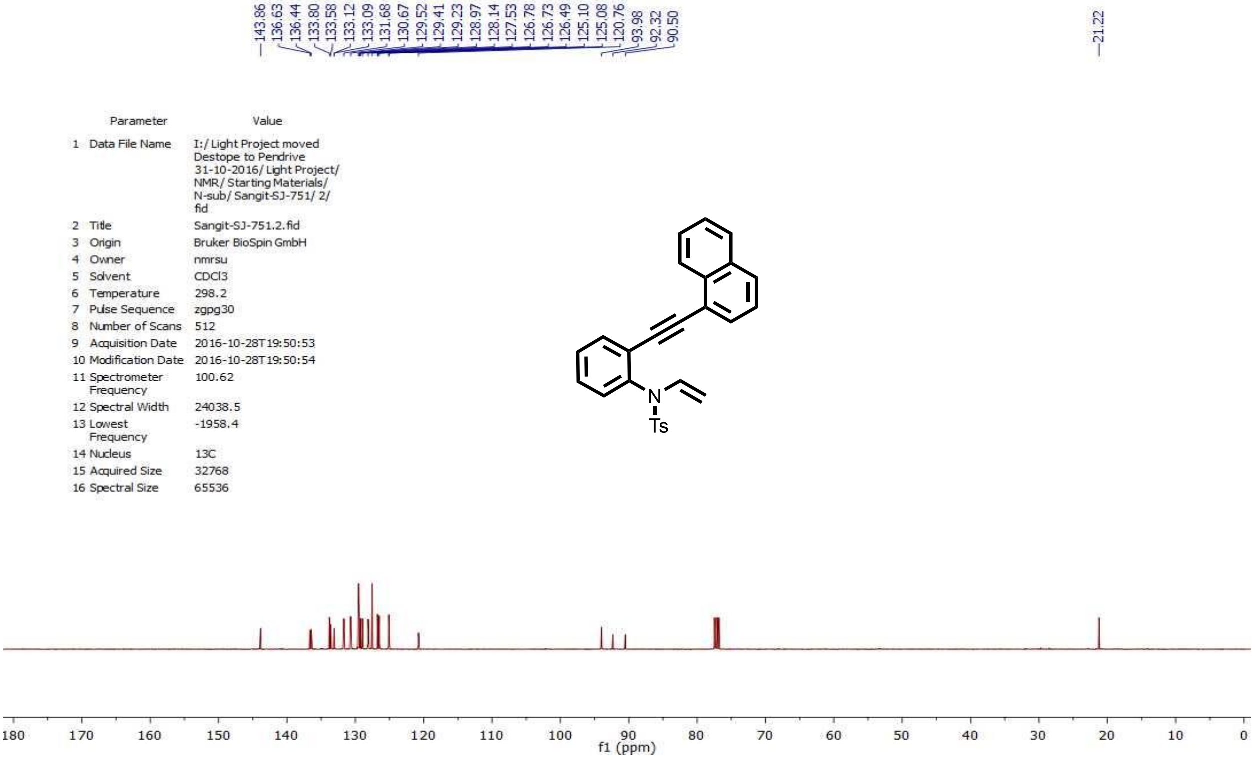

HRMS of 4-Methyl-*N*-(2-(naphthalen-1-ylethynyl)phenyl)-*N*-vinylbenzenesulfonamide

Display Report

Analysis Info

Analysis Name	D:\Data\user data\2016\DEC-2016\07 DEC 2016\Dr.S.Kumar-SJ-751_1-A,8_01_86.d	Acquisition Date	12/7/2016 12:40:51 PM
Method	hrlcms_pos_mid_tunemix.m	Operator	RUCHI SHRIVASTAVA
Sample Name	Dr.S.Kumar-SJ-751	Instrument	micrOTOF-Q II 10330
Comment			

Acquisition Parameter

Source Type	ESI	Ion Polarity	Positive	Set Nebulizer	0.3 Bar
Focus	Active	Set Capillary	4500 V	Set Dry Heater	200 °C
Scan Begin	50 m/z	Set End Plate Offset	-500 V	Set Dry Gas	4.0 l/min
Scan End	3000 m/z	Set Collision Cell RF	450.0 Vpp	Set Divert Valve	Waste

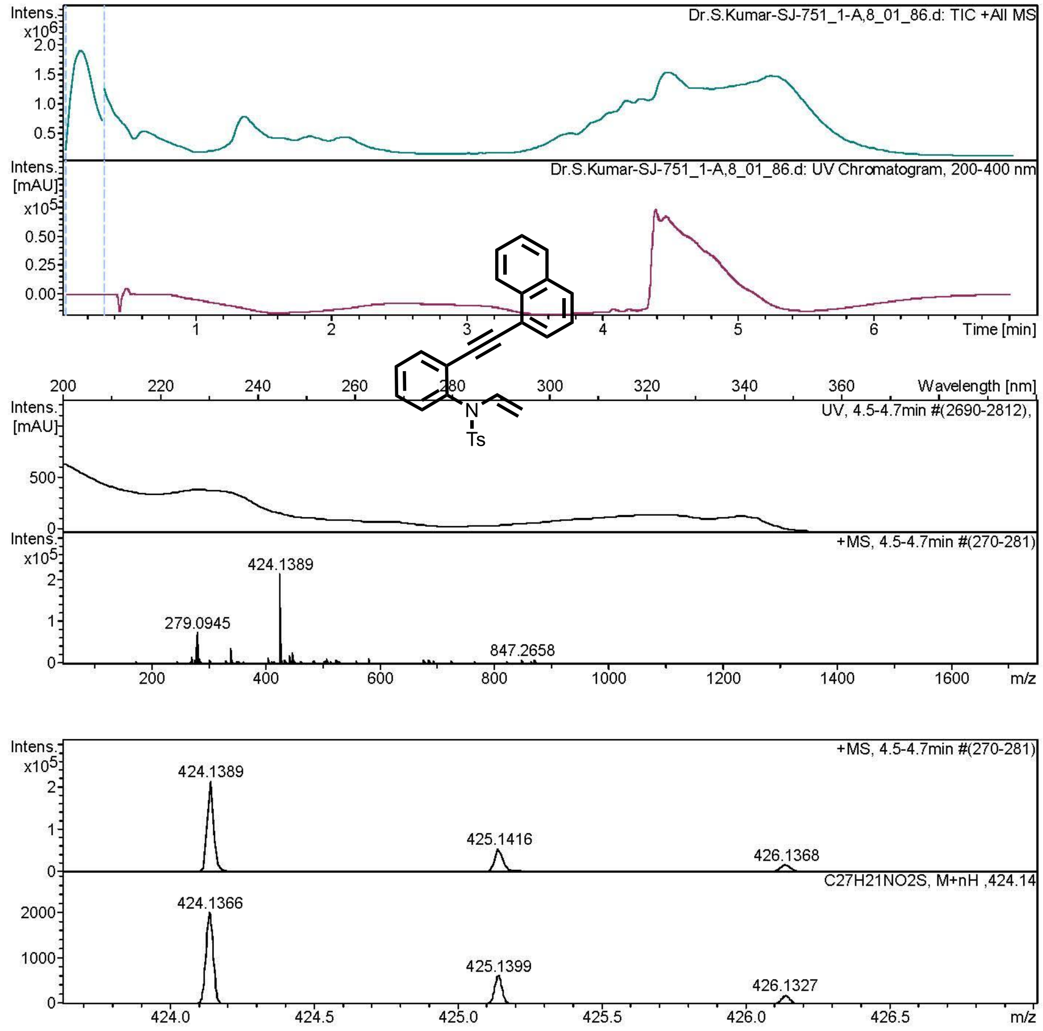

Bruker Compass DataAnalysis 4.0 printed: 12/7/2016 3:52:47 PM Page 1 of 1

^{1}H NMR of *N*-(4-Fluoro-2-(phenylethynyl)phenyl)-4-methyl-*N*-vinylbenzenesulfonamid

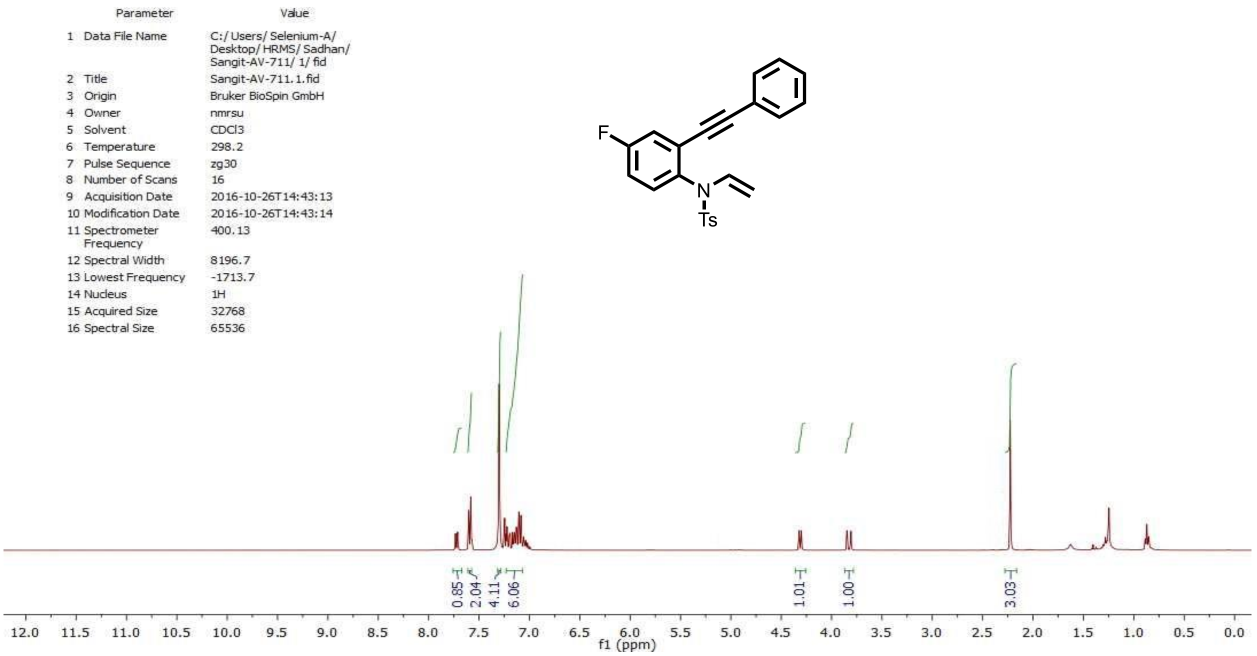

^{13}C NMR of *N*-(4-Fluoro-2-(phenylethynyl)phenyl)-4-methyl-N-vinylbenzenesulfonamid

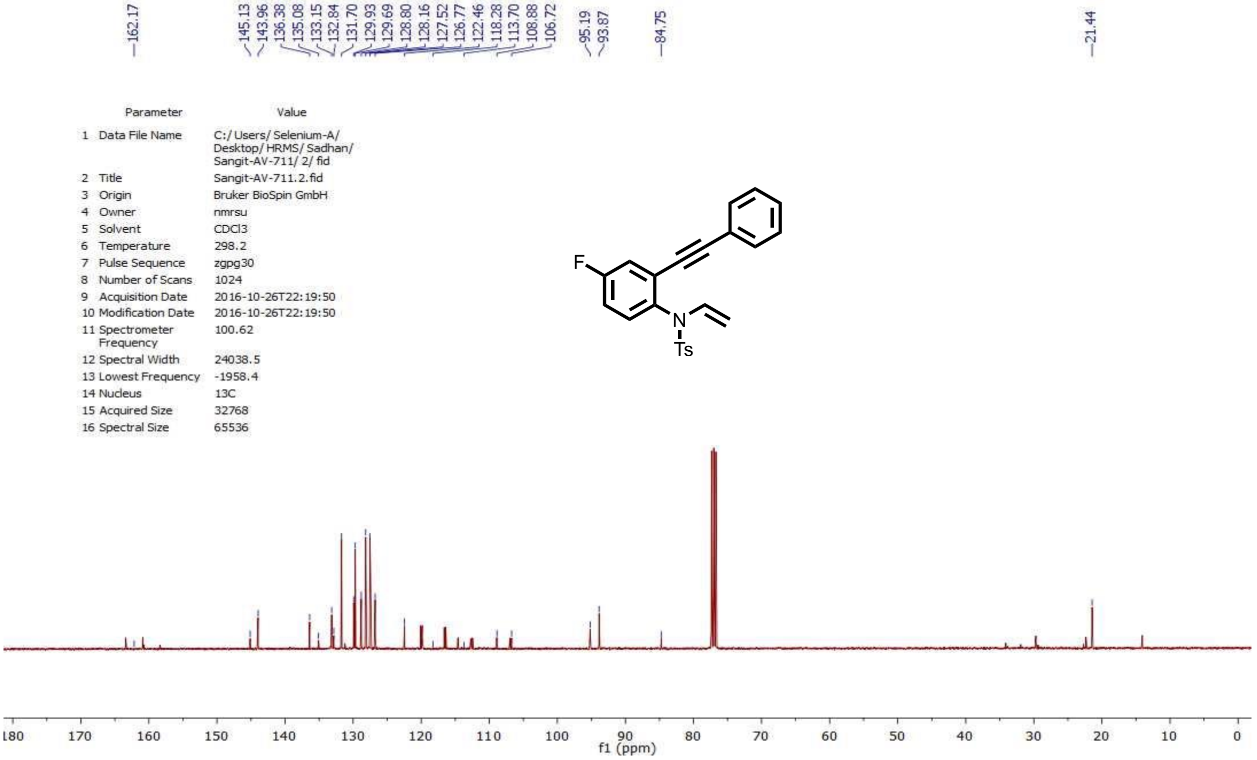

HRMS of *N*-(4-Fluoro-2-(phenylethynyl)phenyl)-4-methyl-N-vinylbenzenesulfonamid

Display Report

Analysis Info

Analysis Name	D:\Data\user data\2016\DEC-2016\5 DEC 2016\Dr.S.Kumar-AV-711_1-A,3_01_31.d	Acquisition Date	12/5/2016 12:08:55 PM
Method	hrlcms_pos_mid_tunemix.m	Operator	RUCHI SHRIVASTAVA
Sample Name	Dr.S.Kumar-AV-711	Instrument	micrOTOF-Q II 10330
Comment			

Acquisition Parameter

Source Type	ESI	Ion Polarity	Positive	Set Nebulizer	0.3 Bar
Focus	Active	Set Capillary	4500 V	Set Dry Heater	200 °C
Scan Begin	50 m/z	Set End Plate Offset	-500 V	Set Dry Gas	4.0 l/min
Scan End	3000 m/z	Set Collision Cell RF	450.0 Vpp	Set Divert Valve	Waste

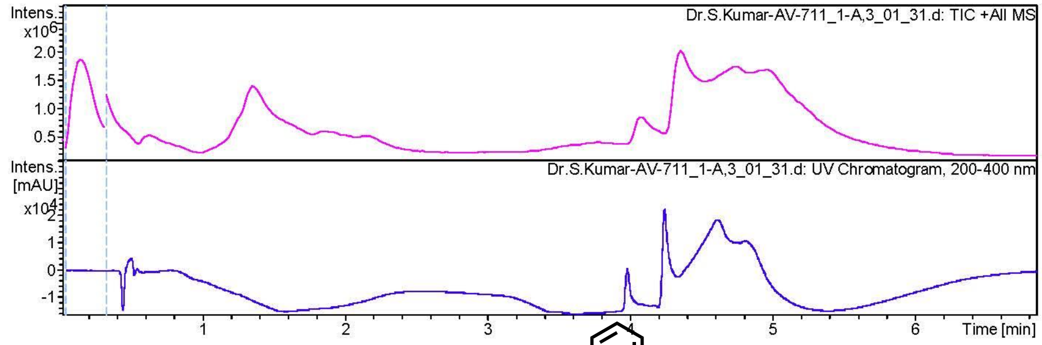

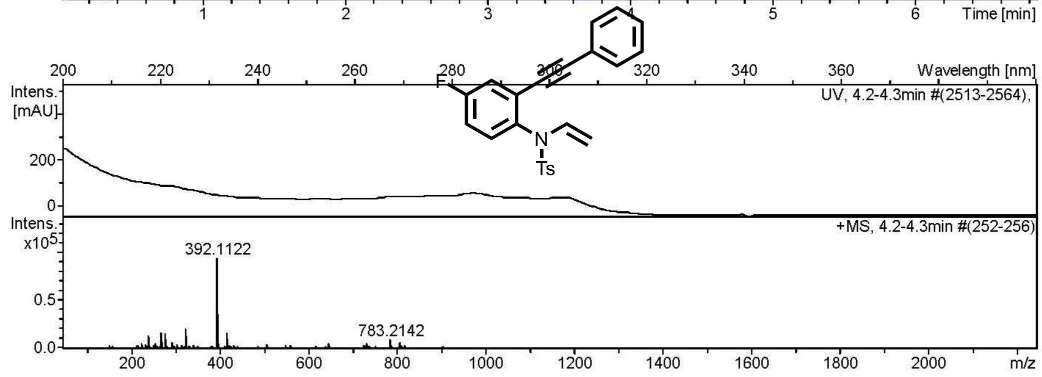

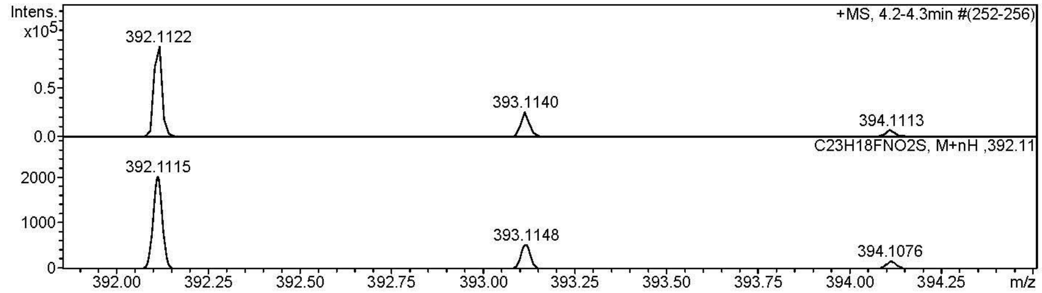

Bruker Compass DataAnalysis 4.0 printed: 12/5/2016 1:01:09 PM Page 1 of 1

^{1}H NMR of *tert*-Butyl (2-(phenylethynyl)phenyl)(vinyl)carbamate

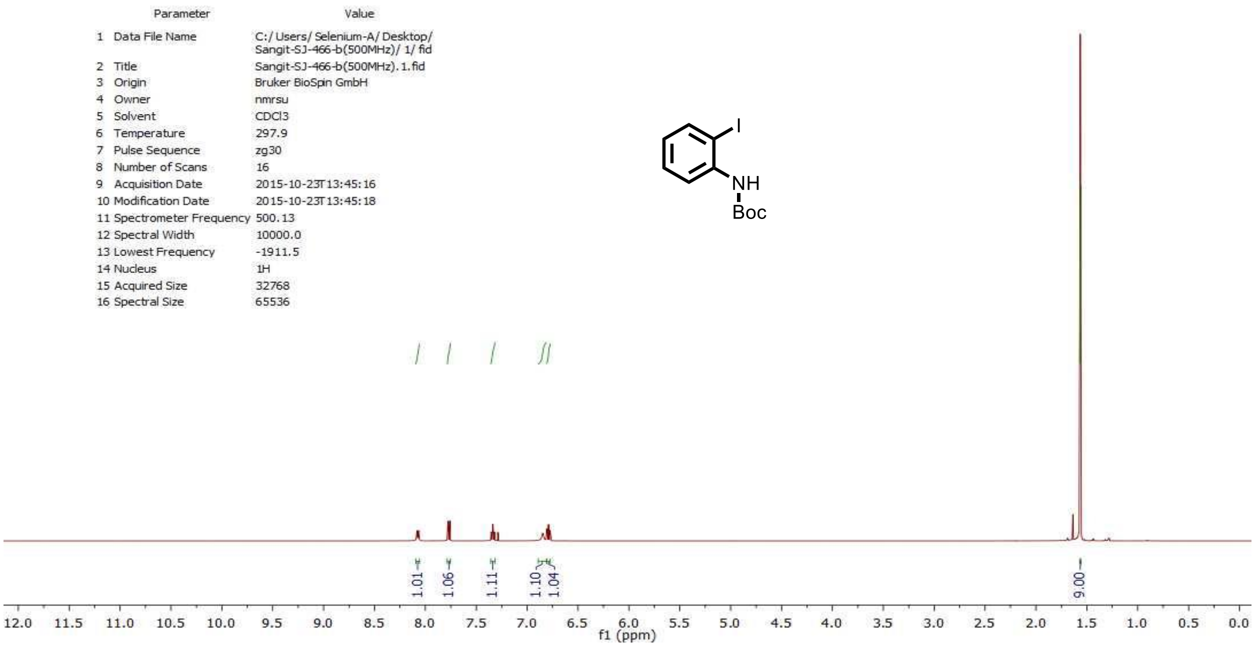

^{1}H NMR of *tert*-Butyl (2-(phenylethynyl)phenyl)(vinyl)carbamate

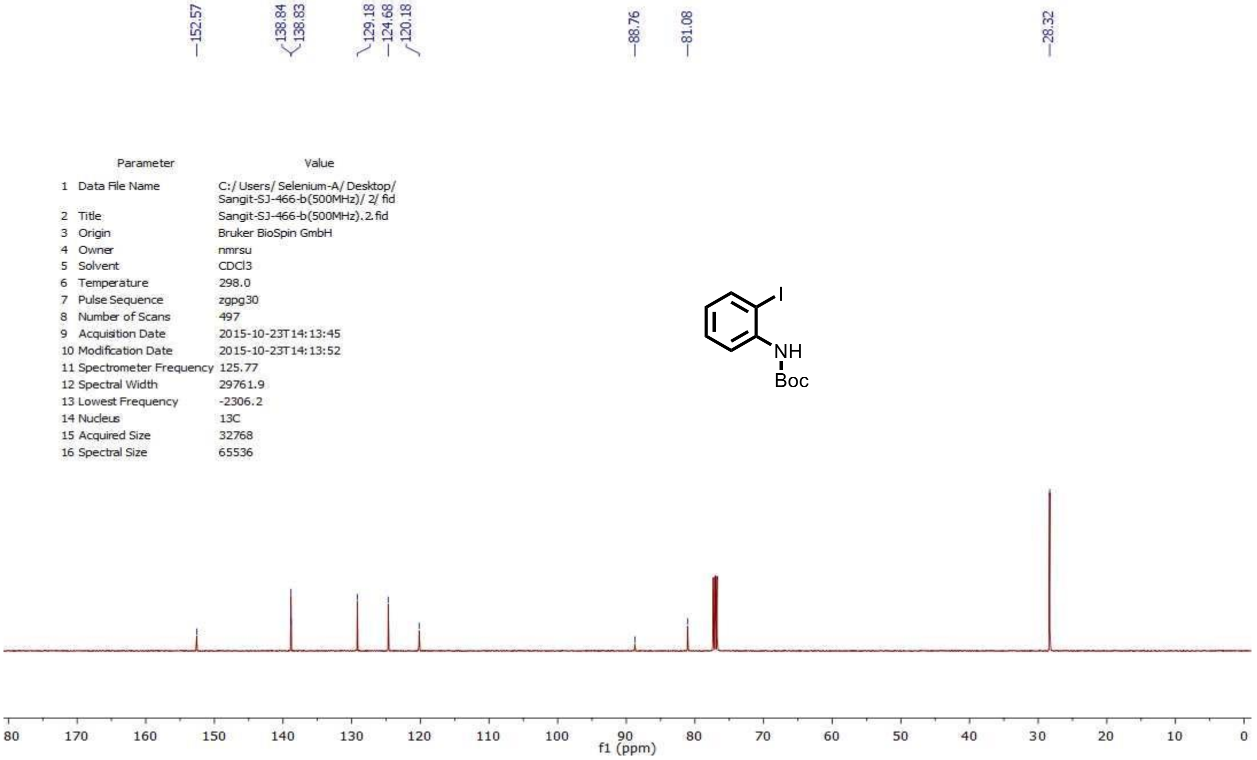

^{1}H NMR of *tert*-Butyl acetyl(2-iodophenyl)carbamate

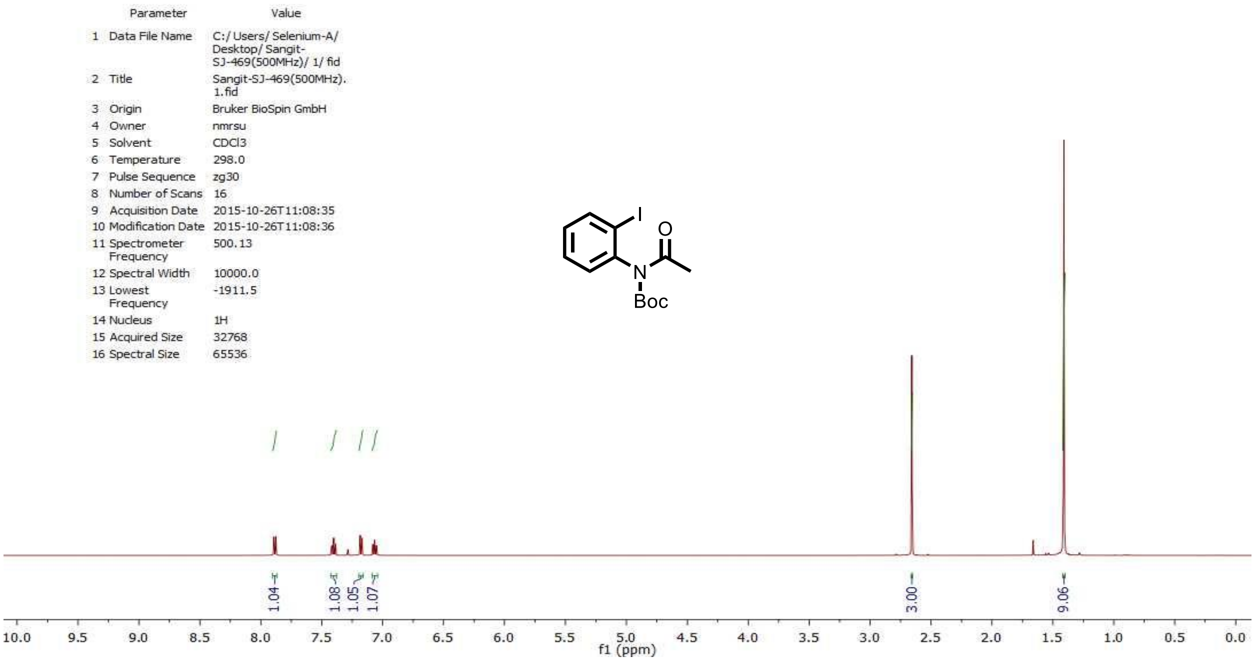

^{13}C NMR of *tert*-Butyl acetyl(2-iodophenyl)carbamate

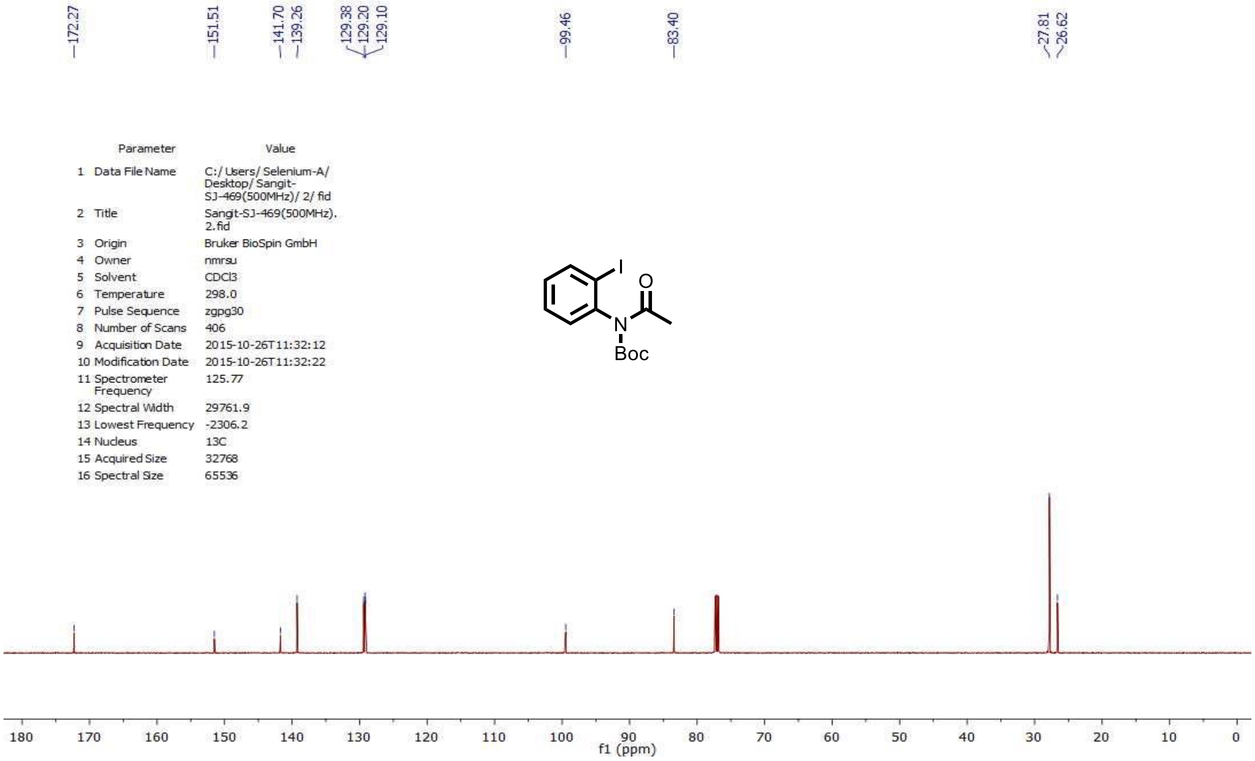

^{1}H NMR of *tert*-Butyl (2-iodophenyl)(vinyl)carbamate

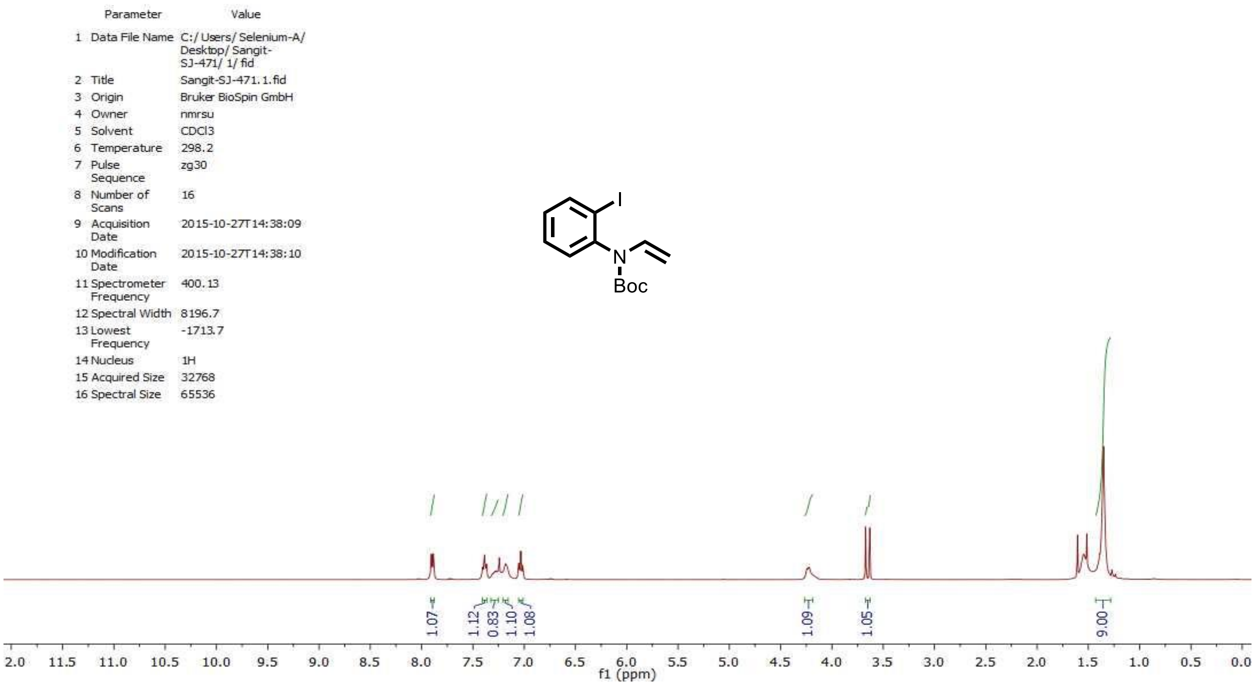

^{13}C NMR of *tert*-Butyl (2-iodophenyl)(vinyl)carbamate

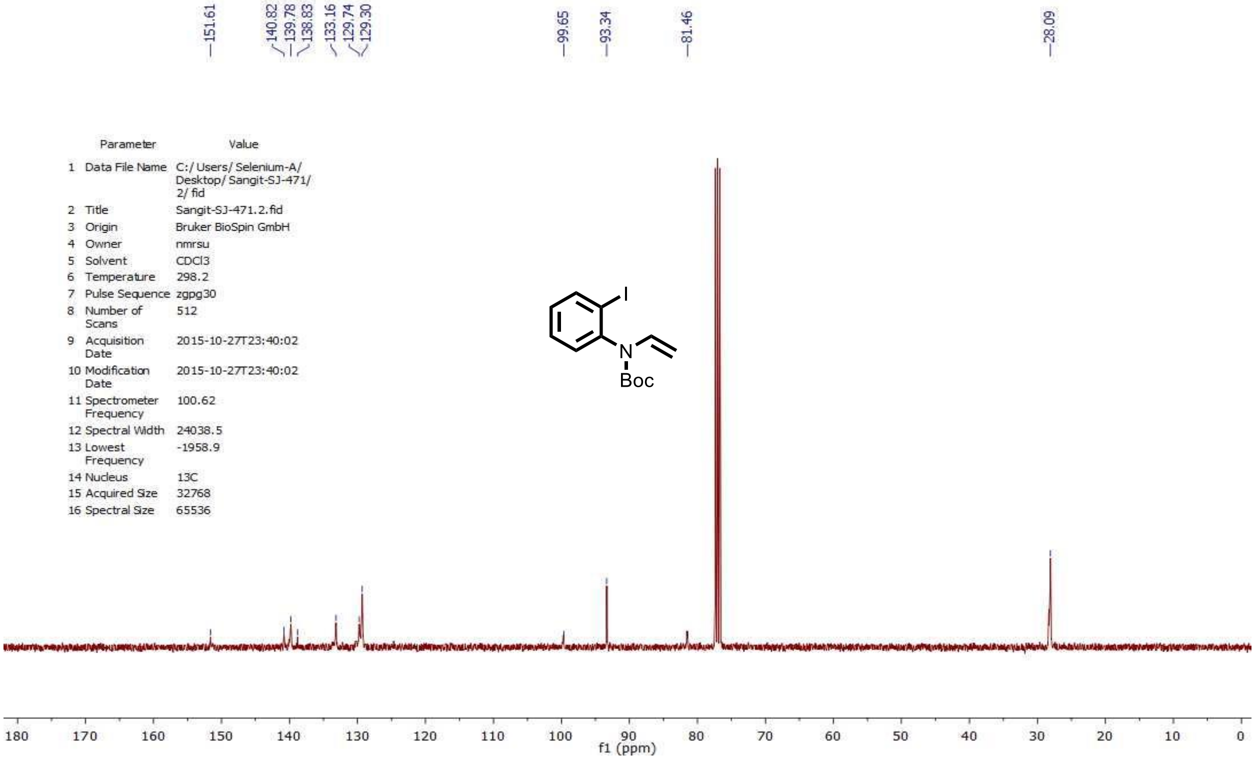

^{1}H NMR of *tert*-Butyl (2-iodophenyl)(vinyl)carbamate

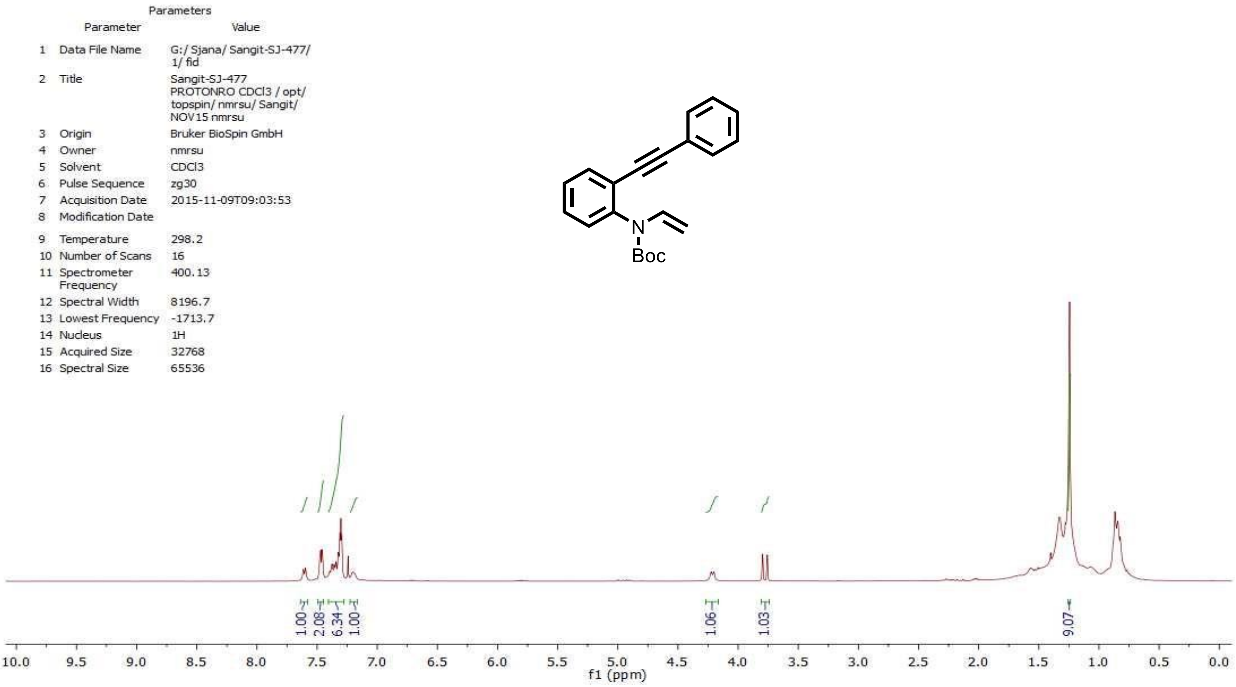

^{13}C NMR of *tert*-Butyl (2-iodophenyl)(vinyl)carbamate

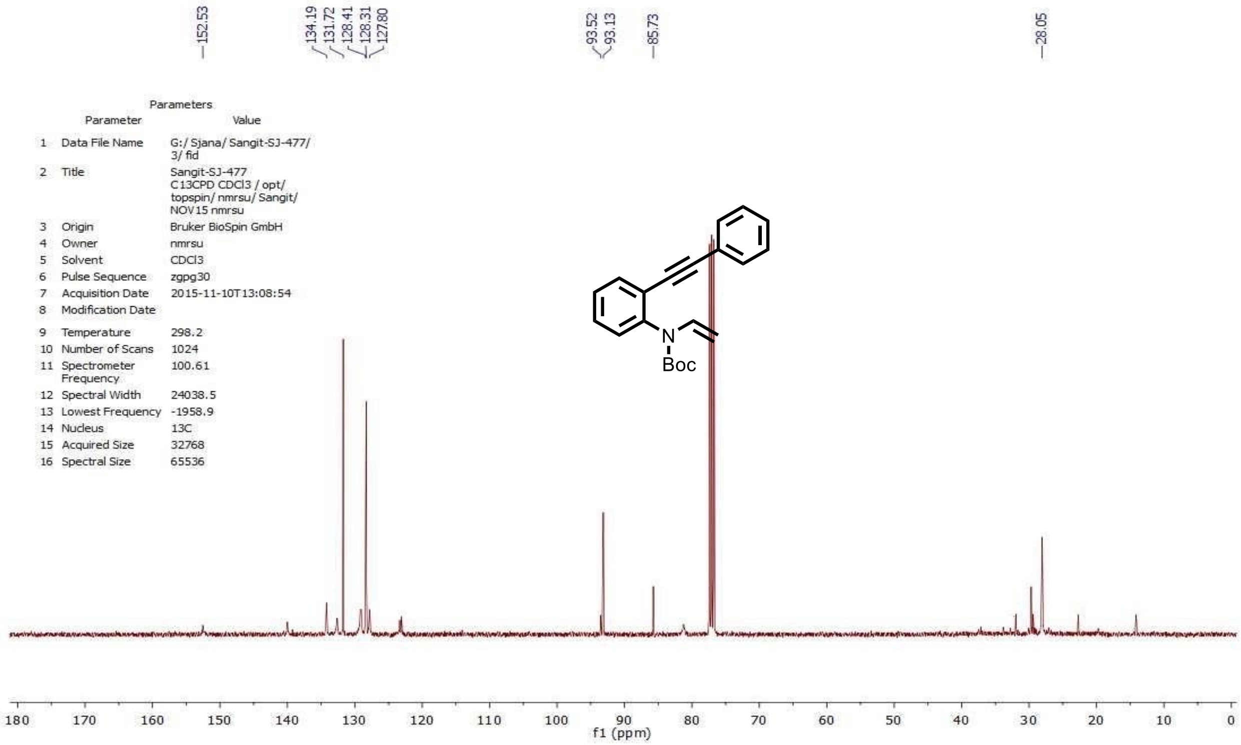

HRMS of tert-butyl (2-(phenylethynyl)phenyl)(vinyl)carbamate

Display Report

Analysis Info

Analysis Name	D:\Data\user data\2016\August 2016\05-08-2016\Dr.S.Kumar-SJ-477_1-A,2_01_7064.d	Acquisition Date	8/5/2016 12:19:07 PM
Method	hrlcms_pos_low_tunemix.m	Operator	DIMPLE
Sample Name	Dr.S.Kumar-SJ-477	Instrument	micrOTOF-Q II 10330
Comment			

Acquisition Parameter

Source Type	ESI	Ion Polarity	Positive	Set Nebulizer	1.0 Bar
Focus	Active	Set Capillary	4500 V	Set Dry Heater	250 °C
Scan Begin	50 m/z	Set End Plate Offset	-500 V	Set Dry Gas	7.0 l/min
Scan End	3000 m/z	Set Collision Cell RF	130.0 Vpp	Set Divert Valve	Waste

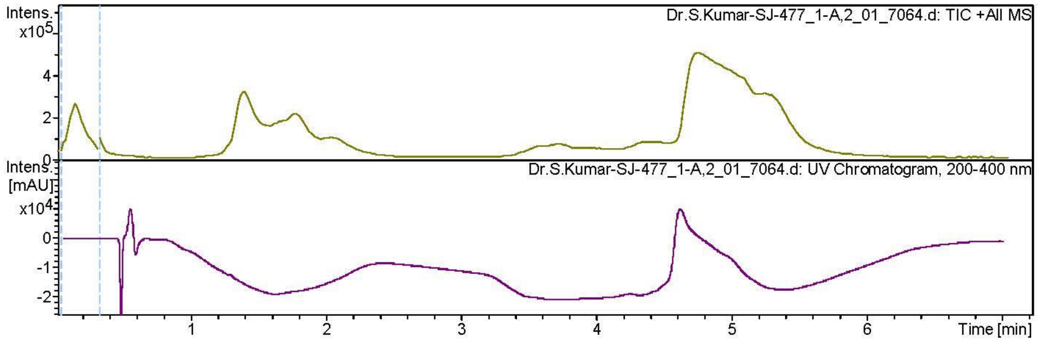

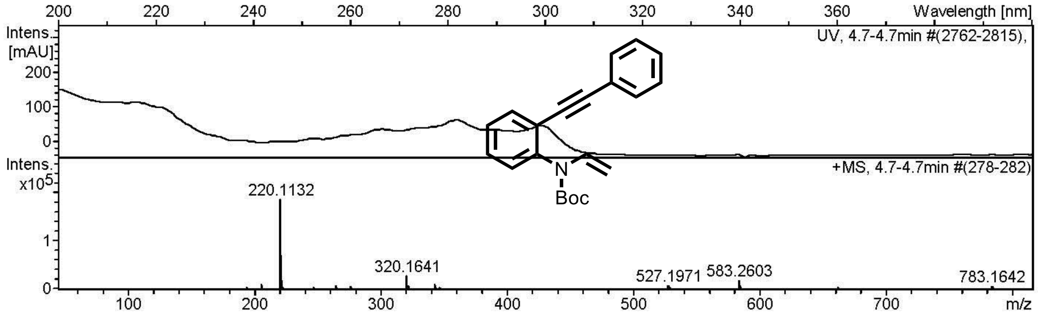

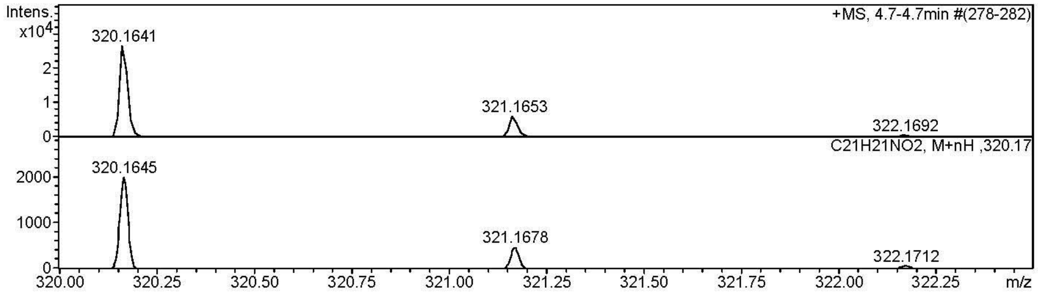

Bruker Compass DataAnalysis 4.0 printed: 8/5/2016 2:37:06 PM Page 1 of 1

^{1}H NMR of *tert*-butyl (2-((3-methoxyphenyl)ethynyl)phenyl)(vinyl)carbamate

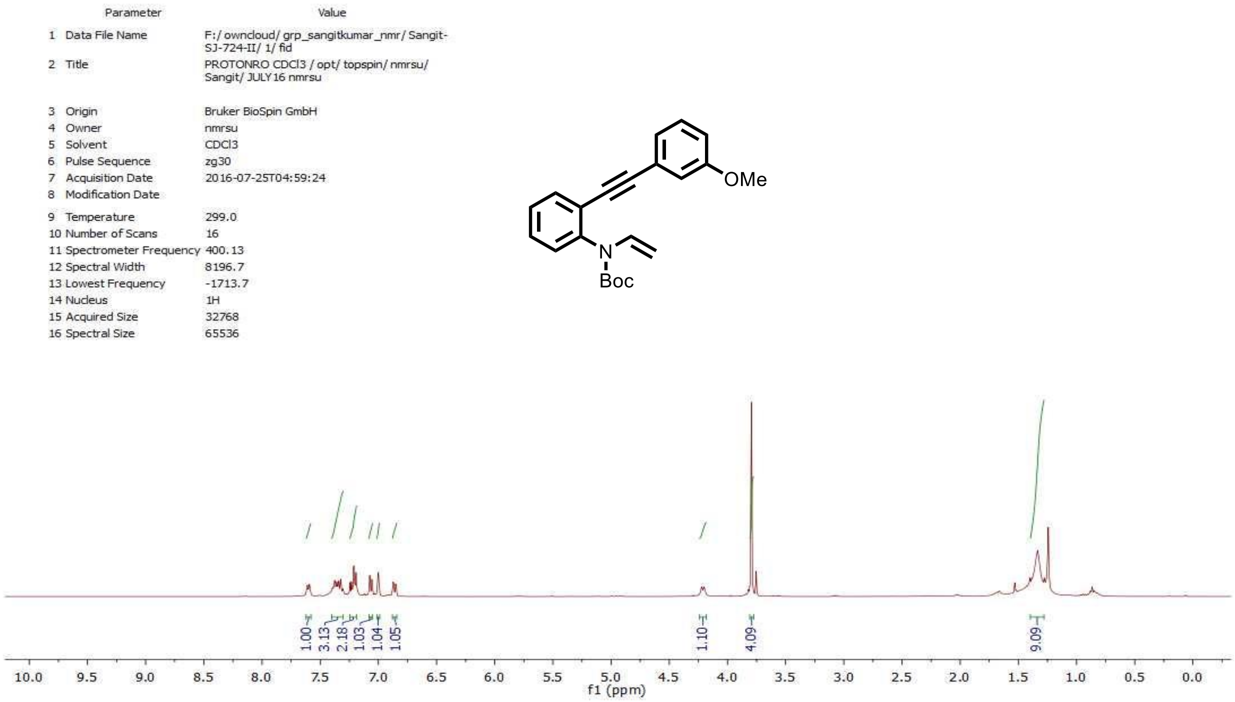

^{13}C NMR of *tert*-butyl (2-((3-methoxyphenyl)ethynyl)phenyl)(vinyl)carbamate

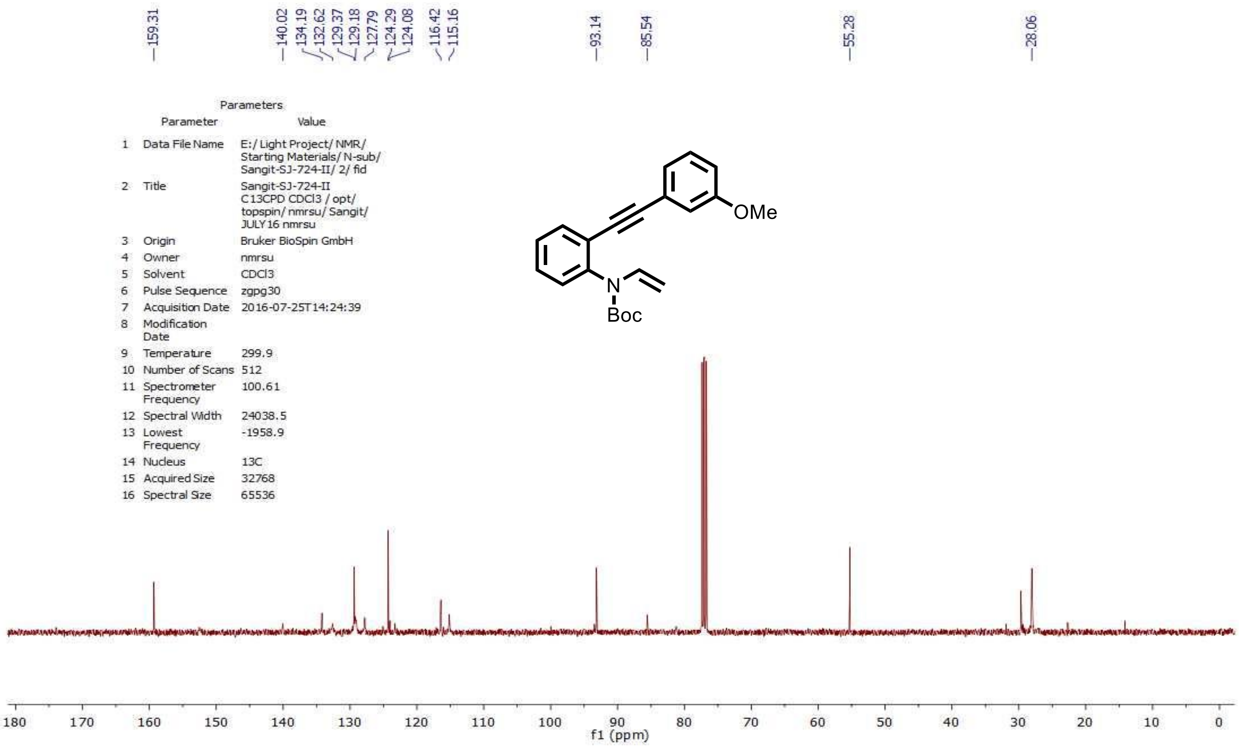

HRMS of *tert*-butyl (2-((3-methoxyphenyl)ethynyl)phenyl)(vinyl)carbamate

Display Report

Analysis Info

		Acquisition Date	8/3/2016 1:36:50 PM
Analysis Name	D:\Data\user data\2016\August 2016\03-08-2016\Dr.S.Kumar-SJ-724_1-A,4_01_7038.d		
Method	hrlcms-pos_mid_tune wide.m	Operator	DIMPLE
Sample Name	Dr.S.Kumar-SJ-724	Instrument	micrOTOF-Q II 10330
Comment			

Acquisition Parameter

Source Type	ESI	Ion Polarity	Positive	Set Nebulizer	0.3 Bar
Focus	Active	Set Capillary	4500 V	Set Dry Heater	200 °C
Scan Begin	50 m/z	Set End Plate Offset	-500 V	Set Dry Gas	4.0 l/min
Scan End	3000 m/z	Set Collision Cell RF	450.0 Vpp	Set Divert Valve	Waste

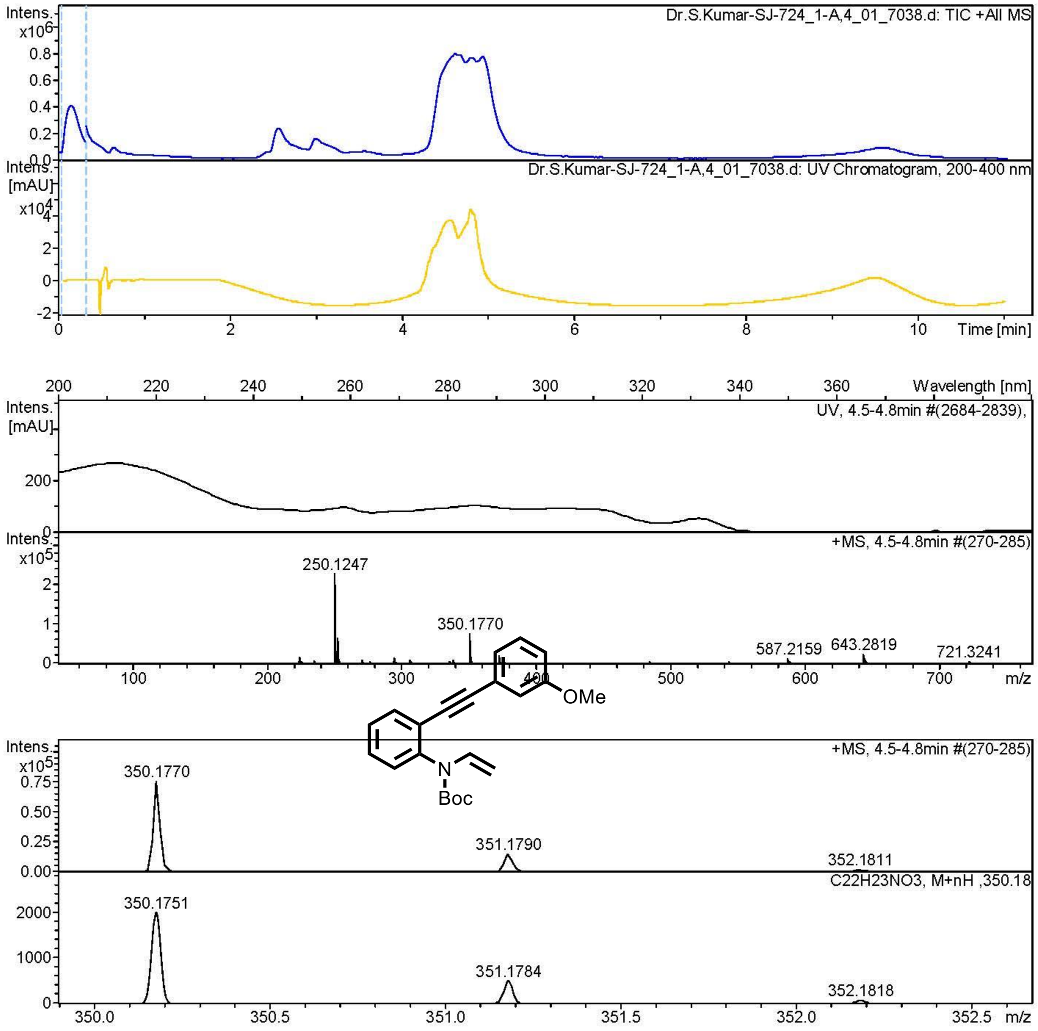

^{1}H NMR of *tert*-Butyl (2-((2-bromophenyl)ethynyl)phenyl)(vinyl)carbamate

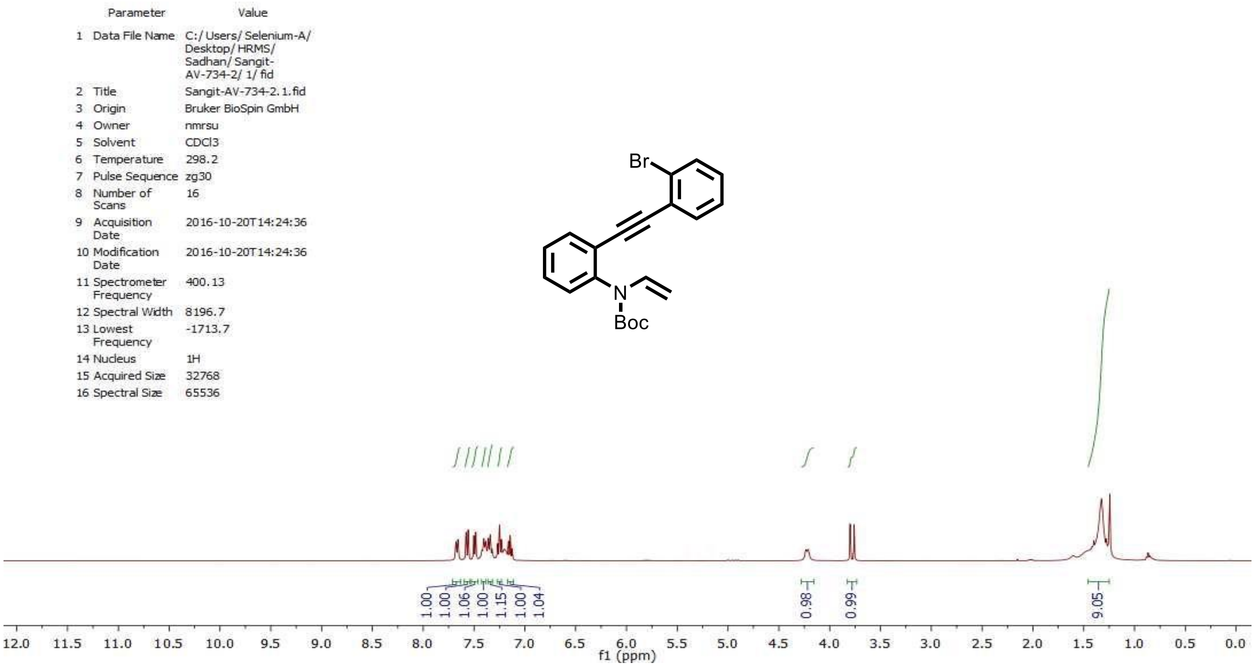

^{13}C NMR of *tert*-Butyl (2-((2-bromophenyl)ethynyl)phenyl)(vinyl)carbamate

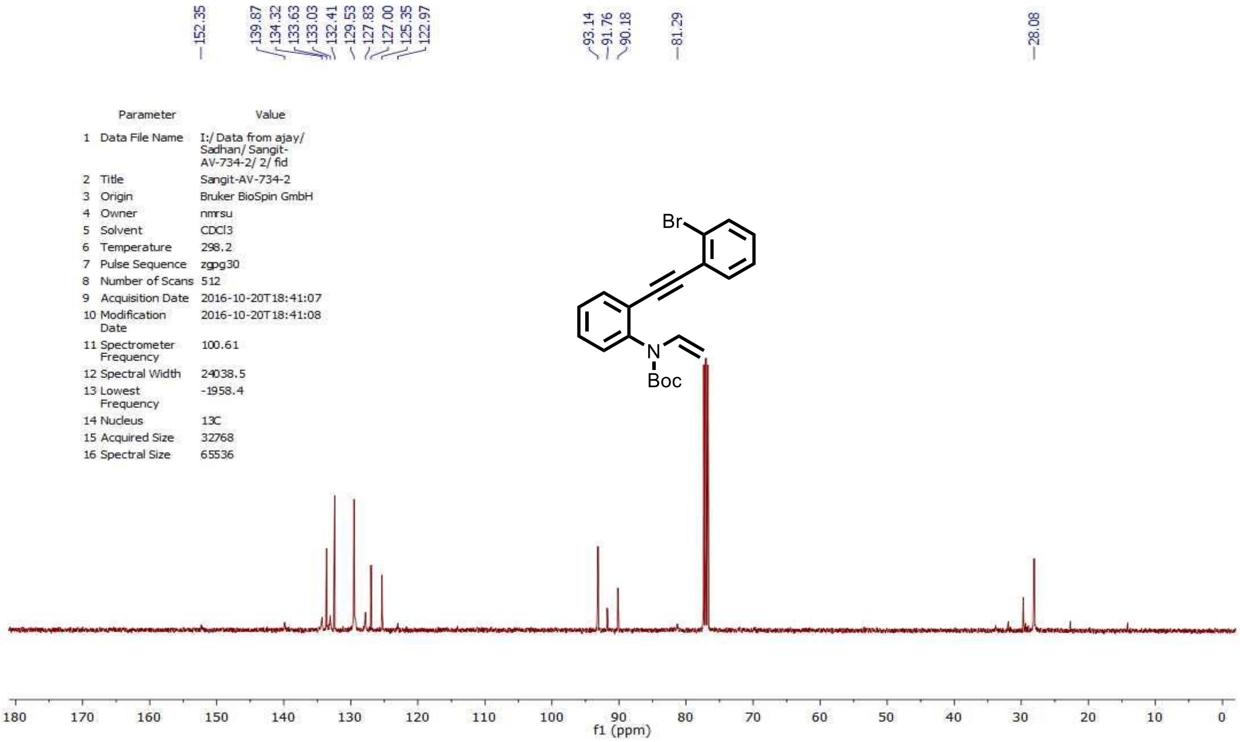

HRMS of *tert*-Butyl (2-((2-bromophenyl)ethynyl)phenyl)(vinyl)carbamate

Display Report

Analysis Info

Analysis Name	D:\Data\user data\2016\DEC-2016\5 DEC 2016\Dr.S.Kumar-AV-734-2_1-A,5_01_33.d	Acquisition Date	12/5/2016 12:25:14 PM
Method	hrlcms_pos_mid_tunemix.m	Operator	RUCHI SHRIVASTAVA
Sample Name	Dr.S.Kumar-AV-734-2	Instrument	micrOTOF-Q II 10330
Comment			

Acquisition Parameter

Source Type	ESI	Ion Polarity	Positive	Set Nebulizer	0.3 Bar
Focus	Active	Set Capillary	4500 V	Set Dry Heater	200 °C
Scan Begin	50 m/z	Set End Plate Offset	-500 V	Set Dry Gas	4.0 l/min
Scan End	3000 m/z	Set Collision Cell RF	450.0 Vpp	Set Divert Valve	Waste

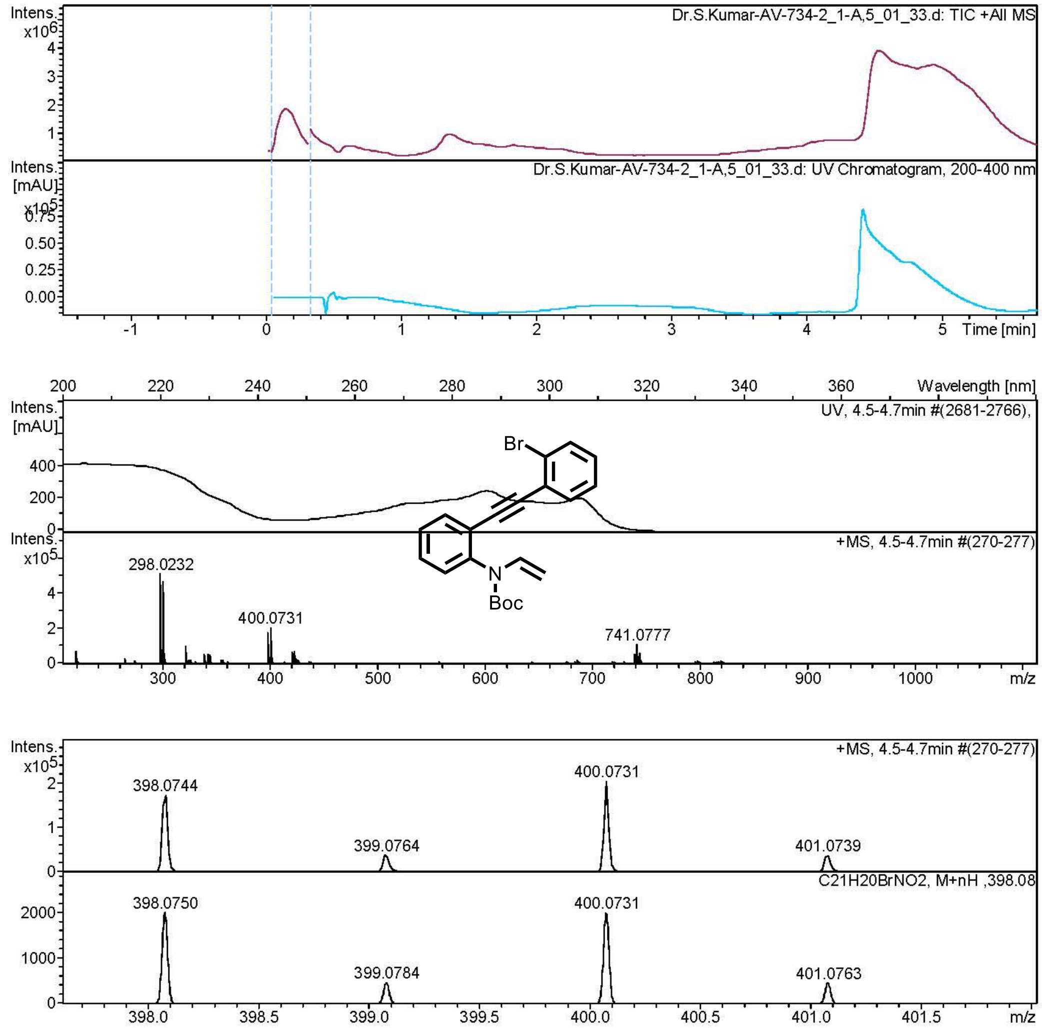

^{1}H NMR of *tert*-Butyl (2-((3,5-difluorophenyl)ethynyl)phenyl)(vinyl)carbamate

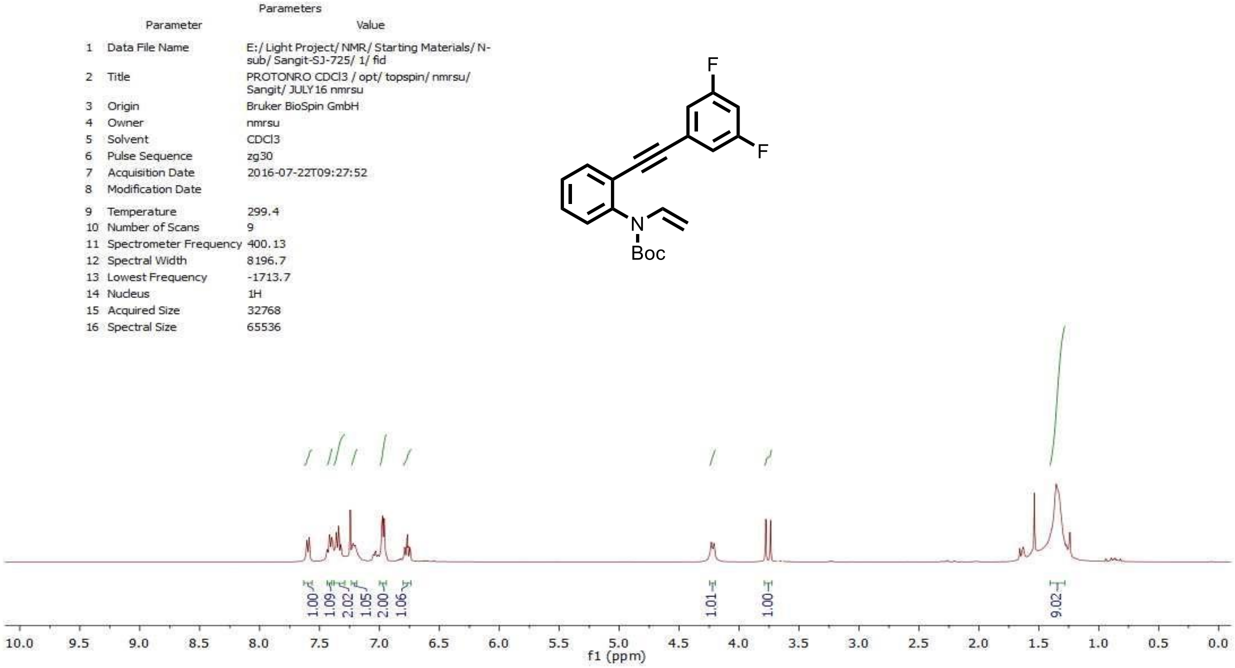

^{13}C NMR of *tert*-Butyl (2-((3,5-difluorophenyl)ethynyl)phenyl)(vinyl)carbamate

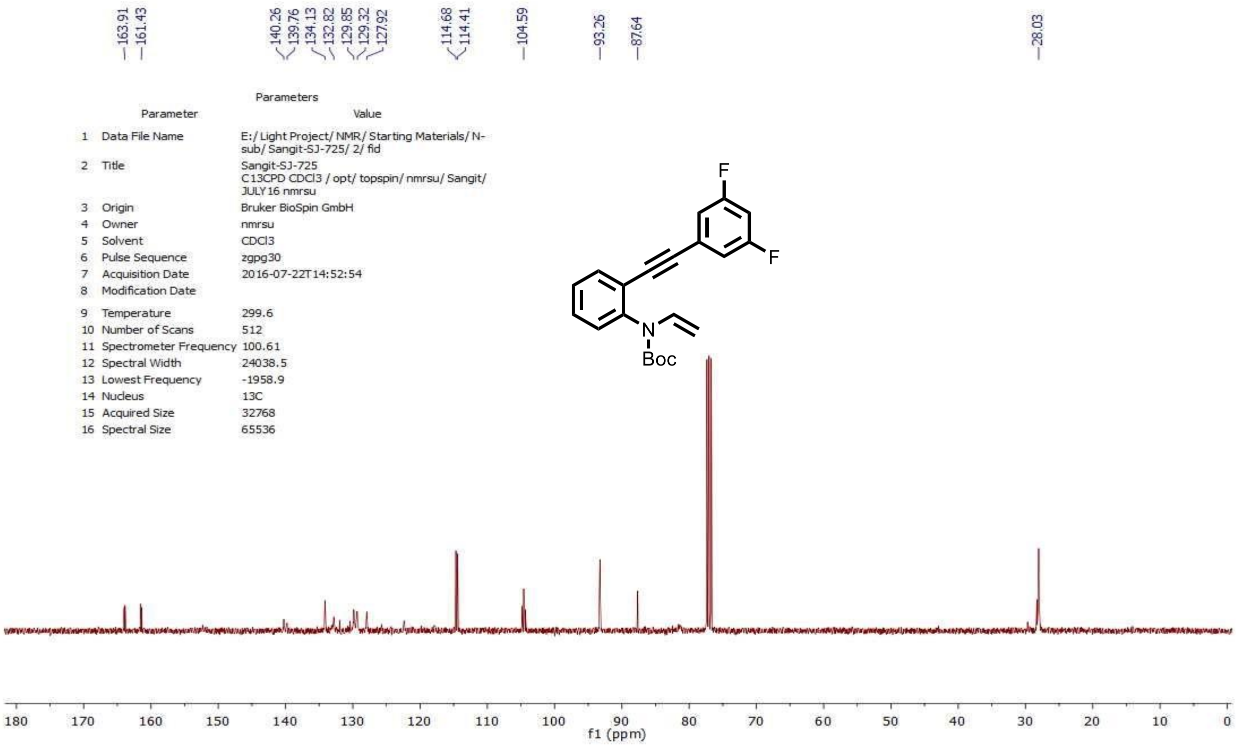

HRMS of *tert*-Butyl (2-((3,5-difluorophenyl)ethynyl)phenyl)(vinyl)carbamate

Display Report

Analysis Info

Analysis Name	D:\Data\user data\2016\August 2016\03-08-2016\Dr.S.Kumar-SJ-725_1-A,3_01_7037.d	Acquisition Date	8/3/2016 1:24:40 PM
Method	hrlcms-pos_mid_tune wide.m	Operator	DIMPLE
Sample Name	Dr.S.Kumar-SJ-725	Instrument	micrOTOF-Q II 10330
Comment			

Acquisition Parameter

Source Type	ESI	Ion Polarity	Positive	Set Nebulizer	0.3 Bar
Focus	Active	Set Capillary	4500 V	Set Dry Heater	200 °C
Scan Begin	50 m/z	Set End Plate Offset	-500 V	Set Dry Gas	4.0 l/min
Scan End	3000 m/z	Set Collision Cell RF	450.0 Vpp	Set Divert Valve	Waste

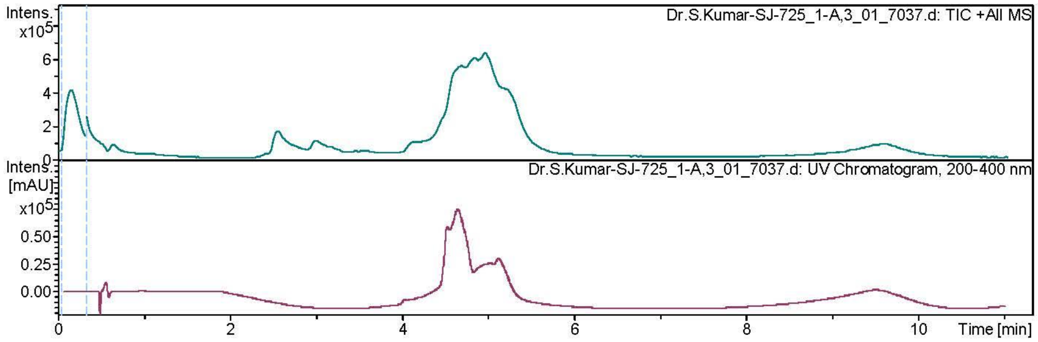

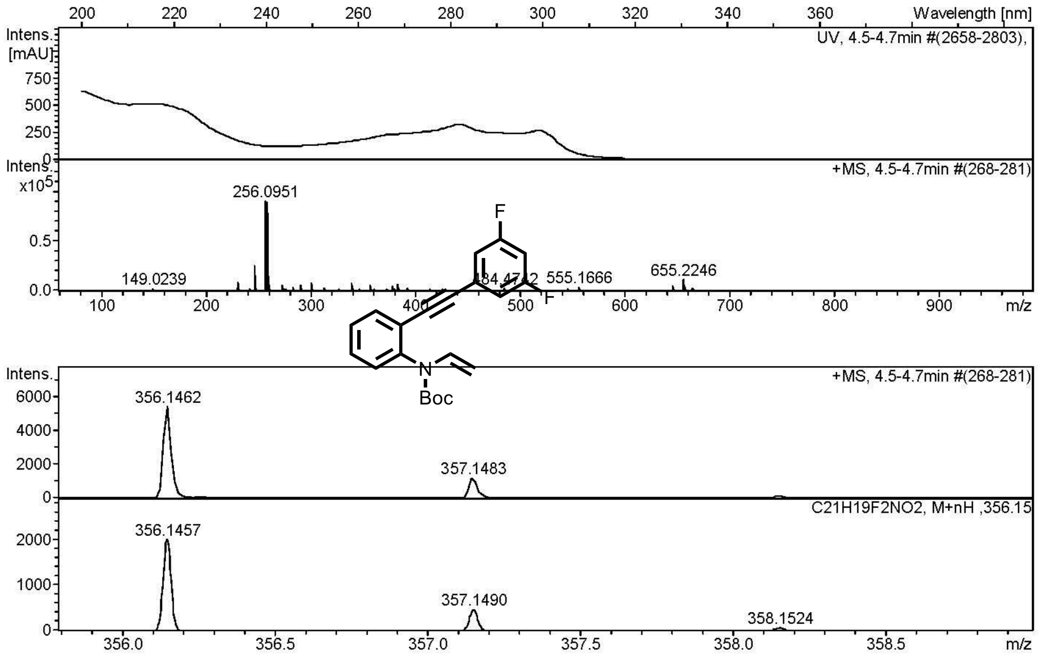

^{1}H NMR of *tert*-Butyl (2-(pyridin-3-ylethynyl)phenyl)(vinyl)carbamate

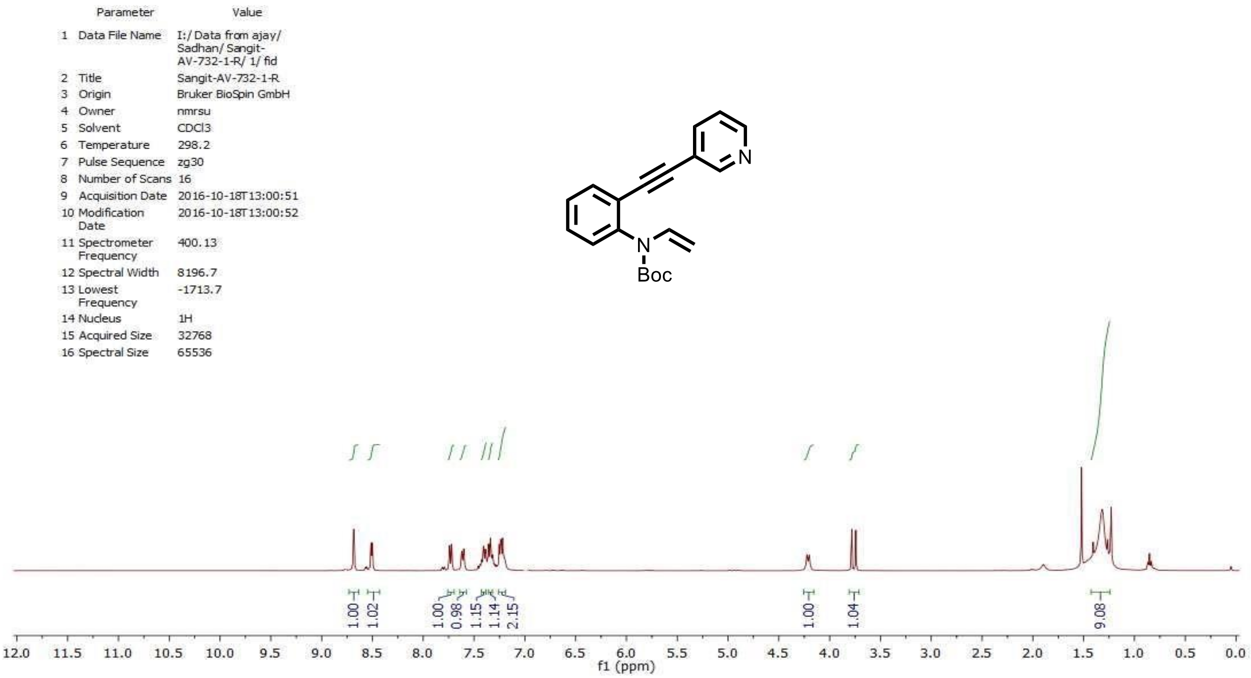

^{13}C NMR of *tert*-Butyl (2-(pyridin-3-ylethynyl)phenyl)(vinyl)carbamate

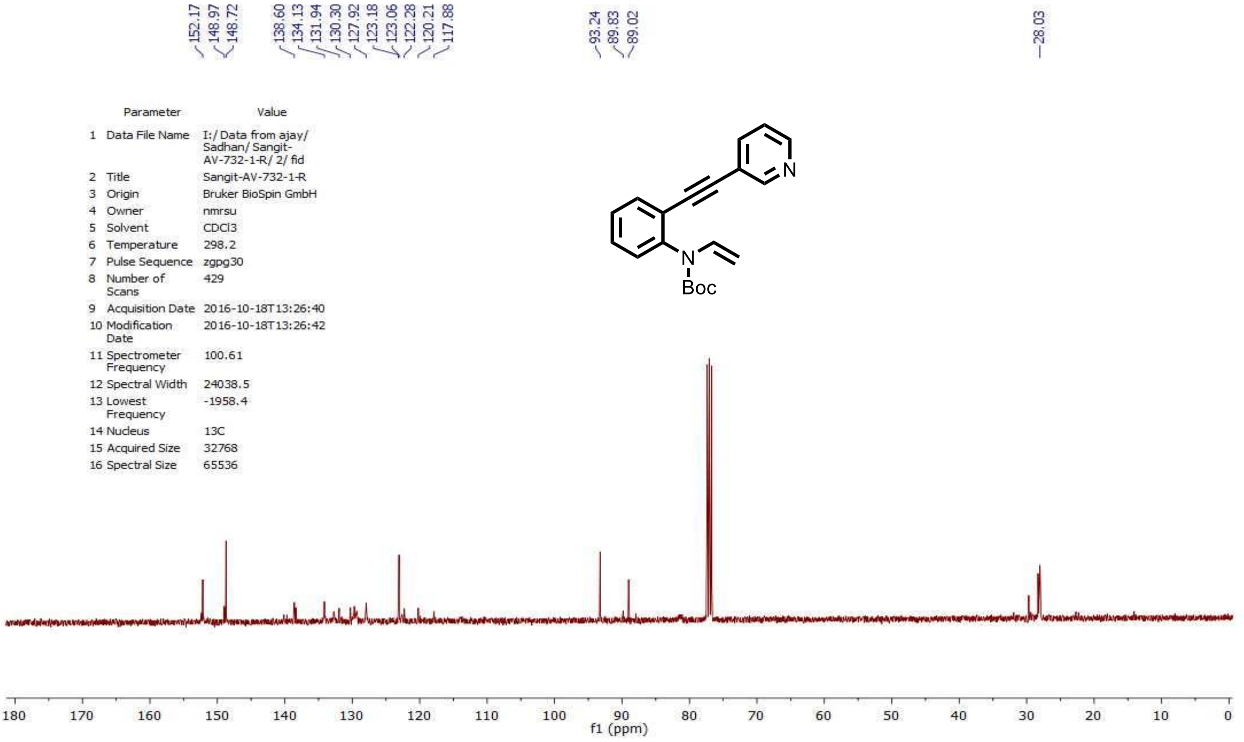

HRMS of *tert*-Butyl (2-(pyridin-3-ylethynyl)phenyl)(vinyl)carbamate

Display Report

Analysis Info

Analysis Name D:\Data\user data\2016\DEC-2016\5 DEC 2016\Dr.S.Kumar-AV-732-1_1-A,4_01_32.d
Method hrlcms_pos_mid_tunemix.m
Sample Name Dr.S.Kumar-AV-732-1
Comment

Acquisition Date 12/5/2016 12:17:04 PM
Operator RUCHI SHRIVASTAVA
Instrument micrOTOF-Q II 10330

Acquisition Parameter

Source Type	ESI	Ion Polarity	Positive	Set Nebulizer	0.3 Bar
Focus	Active	Set Capillary	4500 V	Set Dry Heater	200 °C
Scan Begin	50 m/z	Set End Plate Offset	-500 V	Set Dry Gas	4.0 l/min
Scan End	3000 m/z	Set Collision Cell RF	450.0 Vpp	Set Divert Valve	Waste

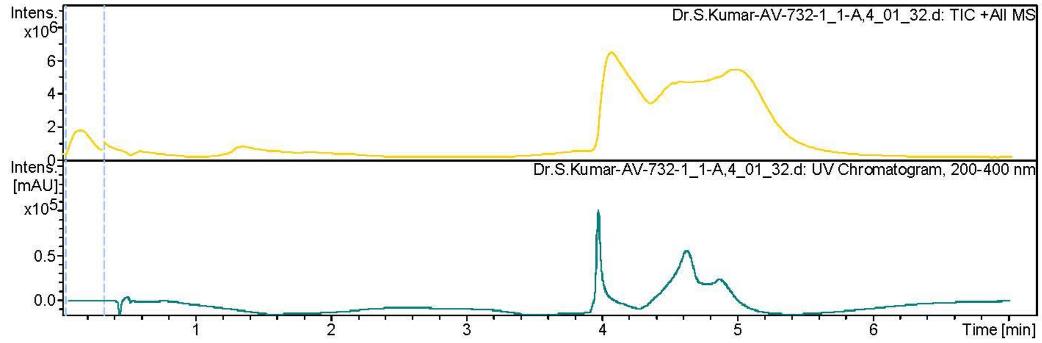

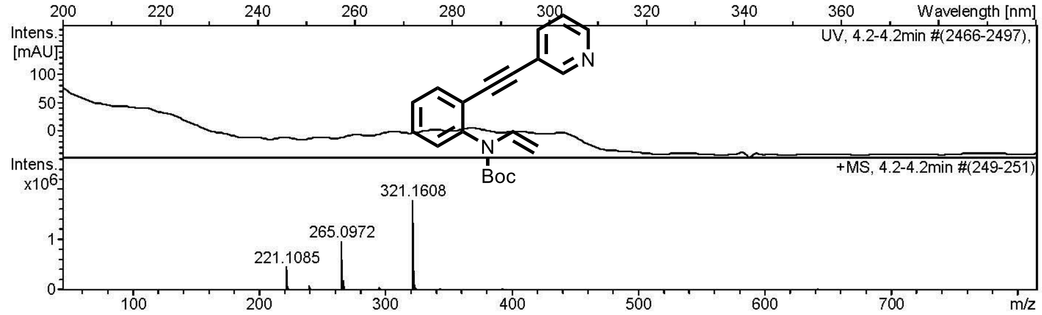

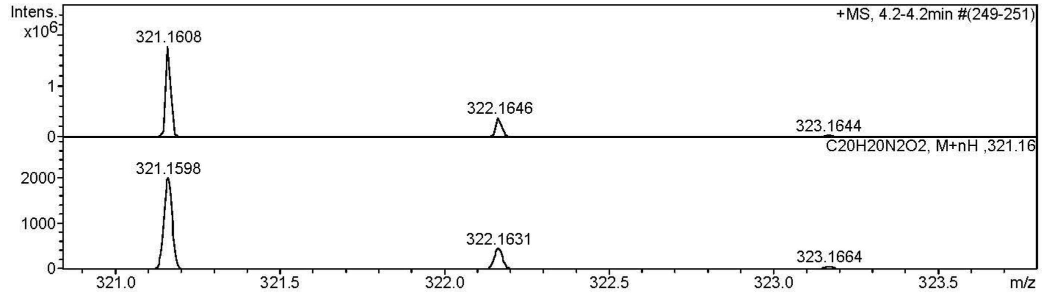

^1H NMR of phenyl(2-(2,2,2-trifluoroethyl)benzofuran-3-yl)methanone (**2a**)

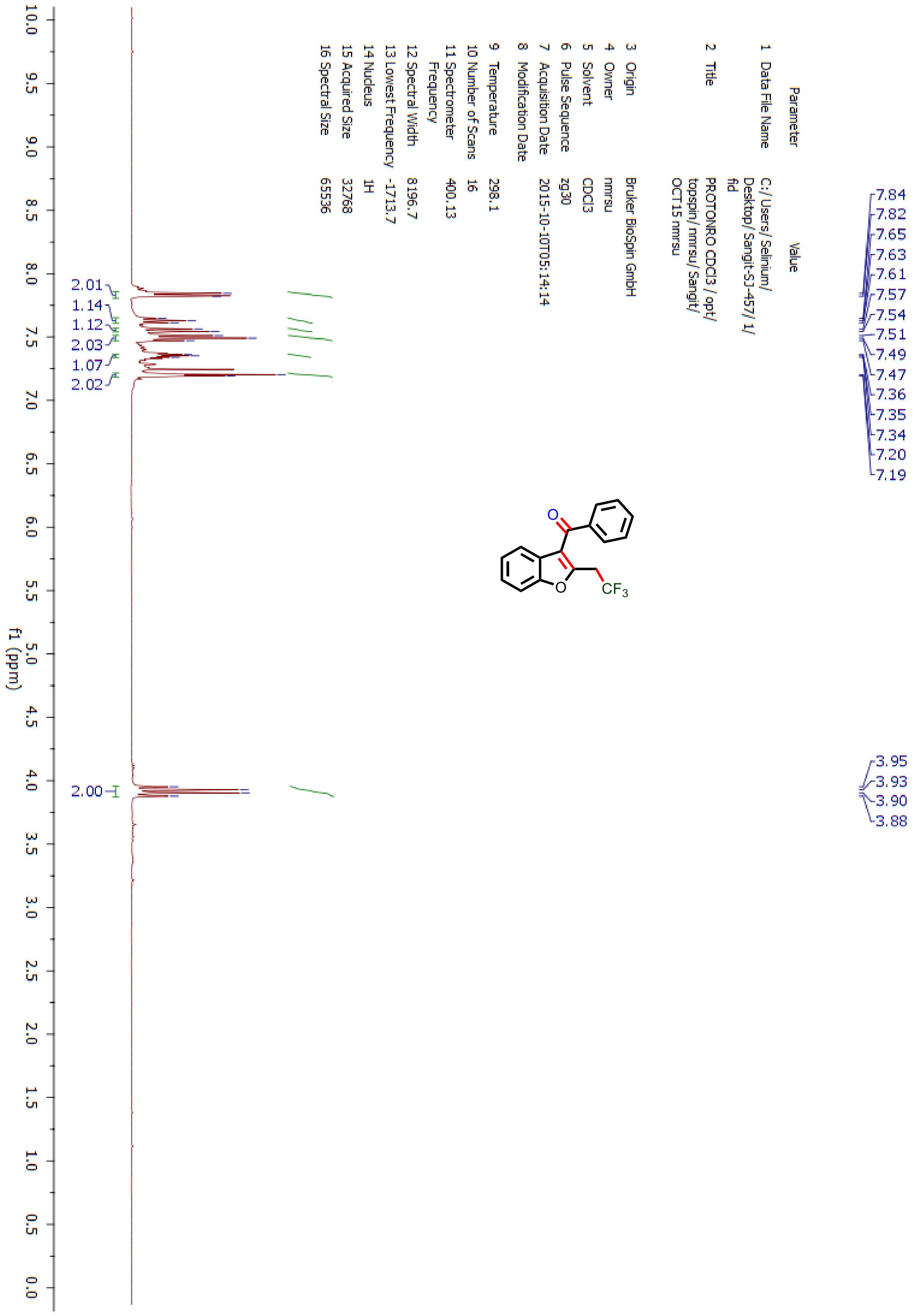

^{13}C NMR of phenyl(2-(2,2,2-trifluoroethyl)benzofuran-3-yl)methanone (**2a**)

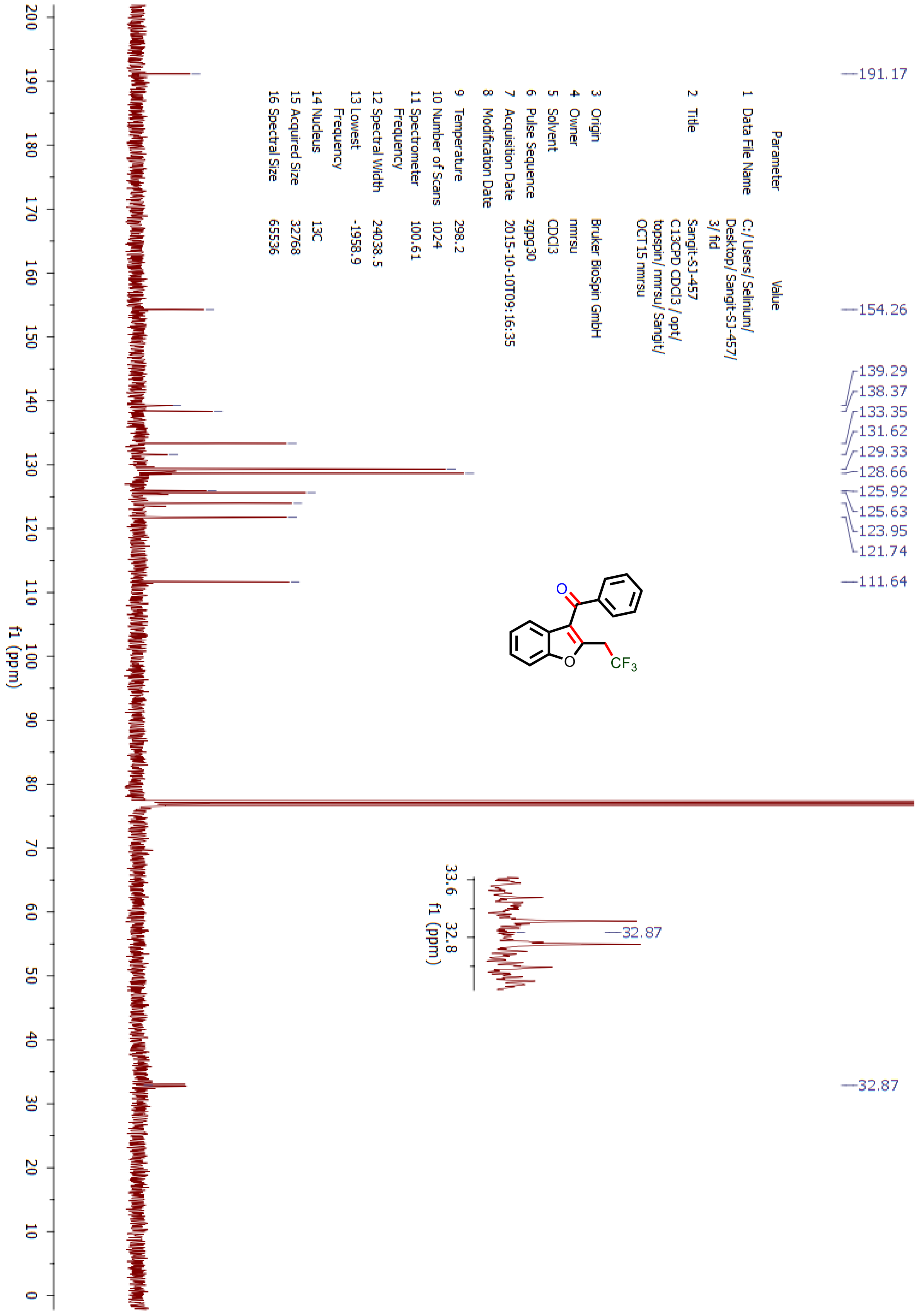

^{19}F NMR of phenyl(2-(2,2,2-trifluoroethyl)benzofuran-3-yl)methanone (**2a**)

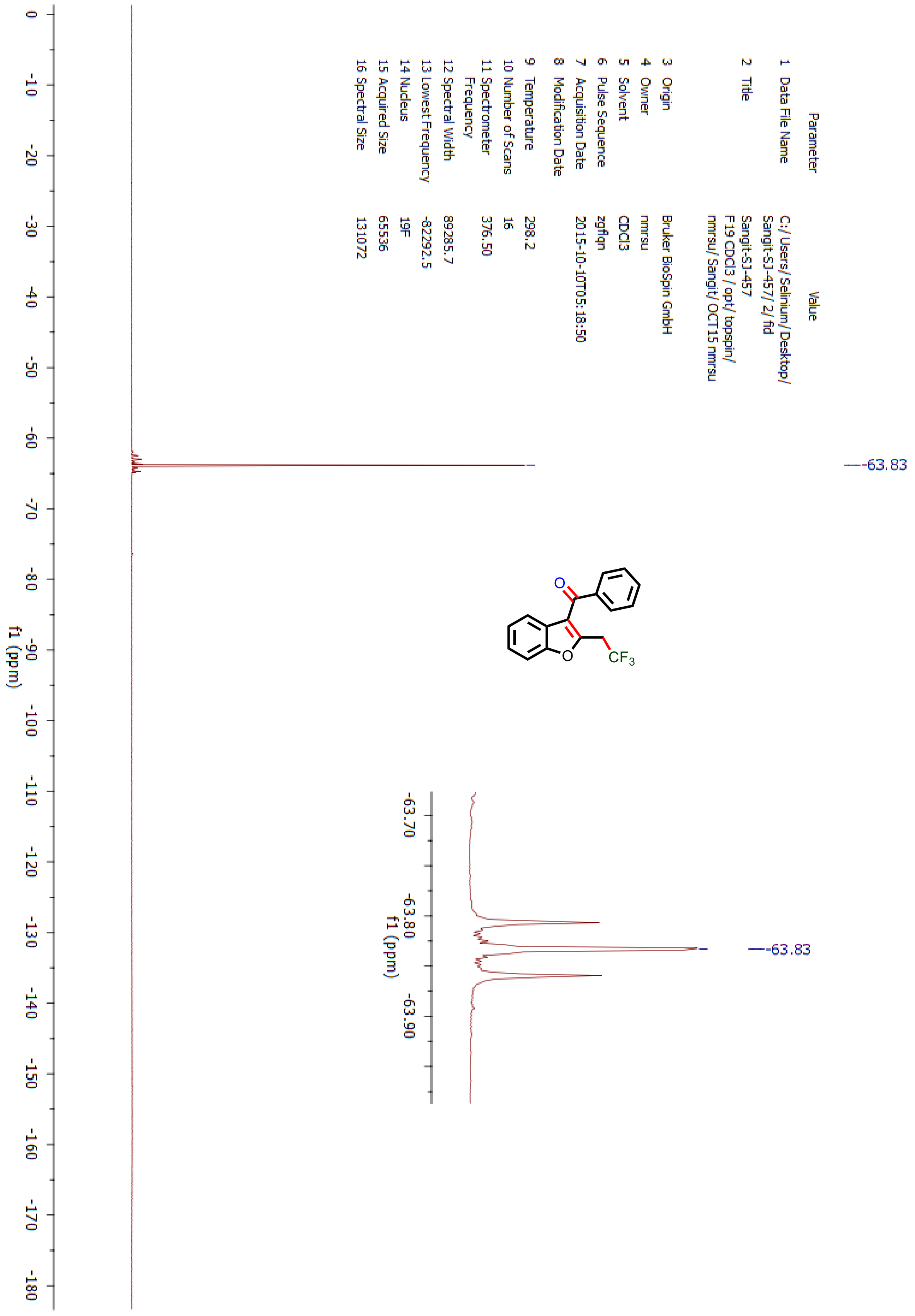

HRMS of phenyl(2-(2,2,2-trifluoroethyl)benzofuran-3-yl)methanone

Display Report

Analysis Info

Analysis Name	D:\Data\user data\2015\Oct-2015\12 Oct-2015\Dr.S.Kumar-SJ-457_1-A,6_01_3930.d	Acquisition Date	10/12/2015 4:13:10 PM
Method	HRLCMS-20 Sept.m	Operator	RUCHI
Sample Name	Dr.S.Kumar-SJ-457	Instrument	micrOTOF-Q II 10330
Comment			

Acquisition Parameter

Source Type	ESI	Ion Polarity	Positive	Set Nebulizer	1.2 Bar
Focus	Active	Set Capillary	4500 V	Set Dry Heater	200 °C
Scan Begin	50 m/z	Set End Plate Offset	-500 V	Set Dry Gas	7.0 l/min
Scan End	3000 m/z	Set Collision Cell RF	130.0 Vpp	Set Divert Valve	Waste

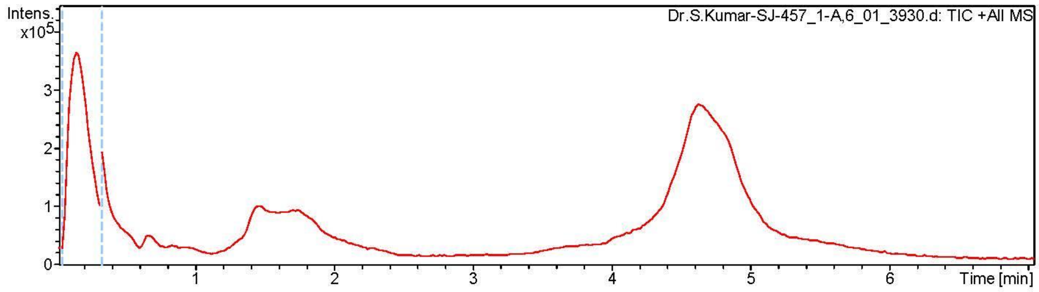

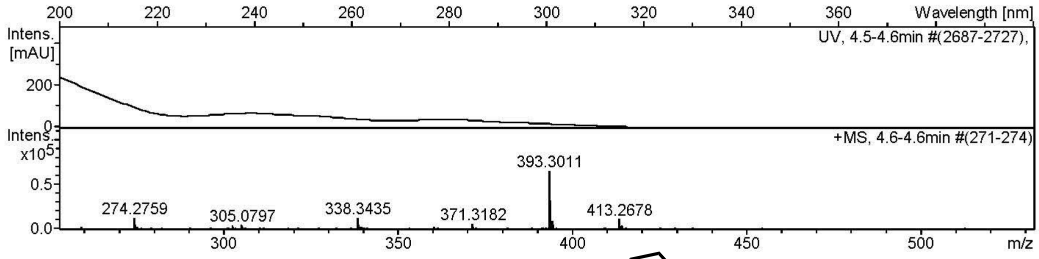

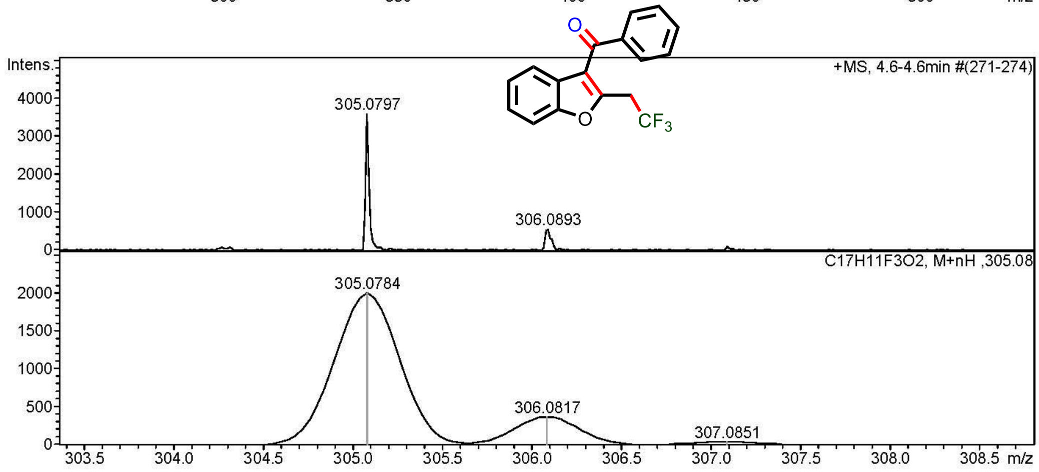

Bruker Compass DataAnalysis 4.0 printed: 10/12/2015 5:24:54 PM Page 1 of 1

^{1}H NMR of (4-fluorophenyl)(2-(2,2,2-trifluoroethyl)benzofuran-3-yl)methanone (**2b**)

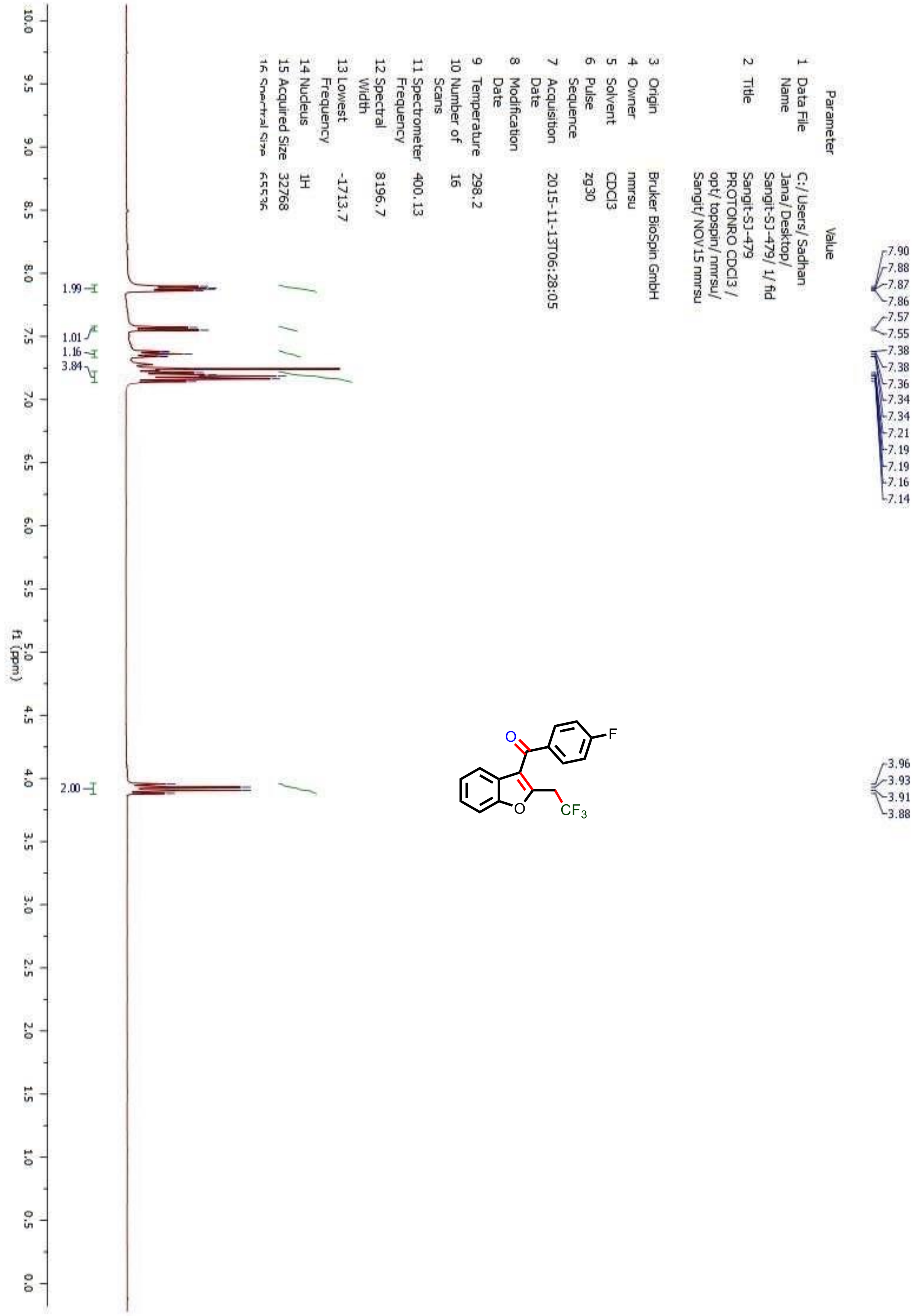

^{13}C NMR of (4-fluorophenyl)(2-(2,2,2-trifluoroethyl)benzofuran-3-yl)methanone (**2b**)

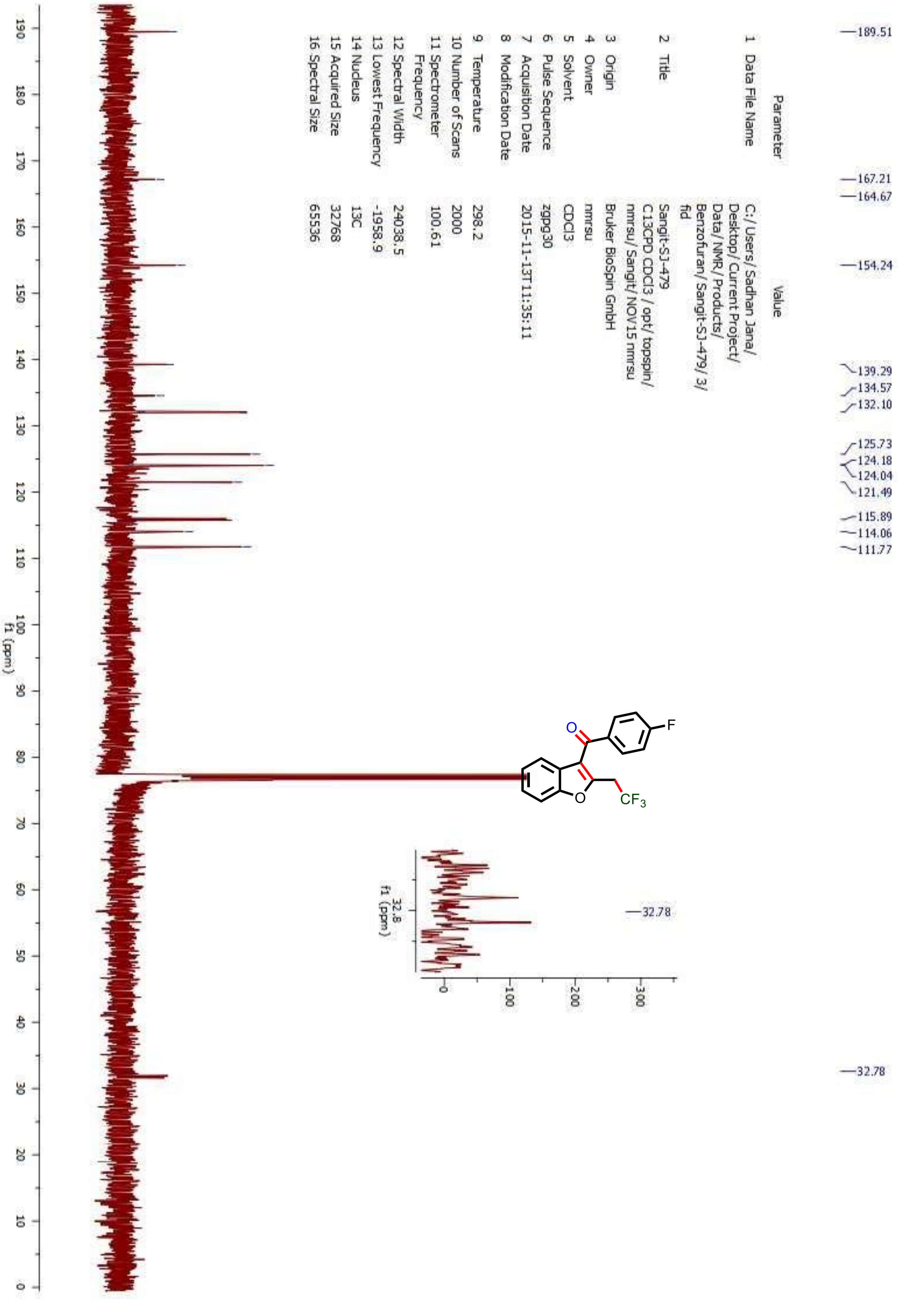

^{19}F NMR of (4-fluorophenyl)(2-(2,2,2-trifluoroethyl)benzofuran-3-yl)methanone (**2b**)

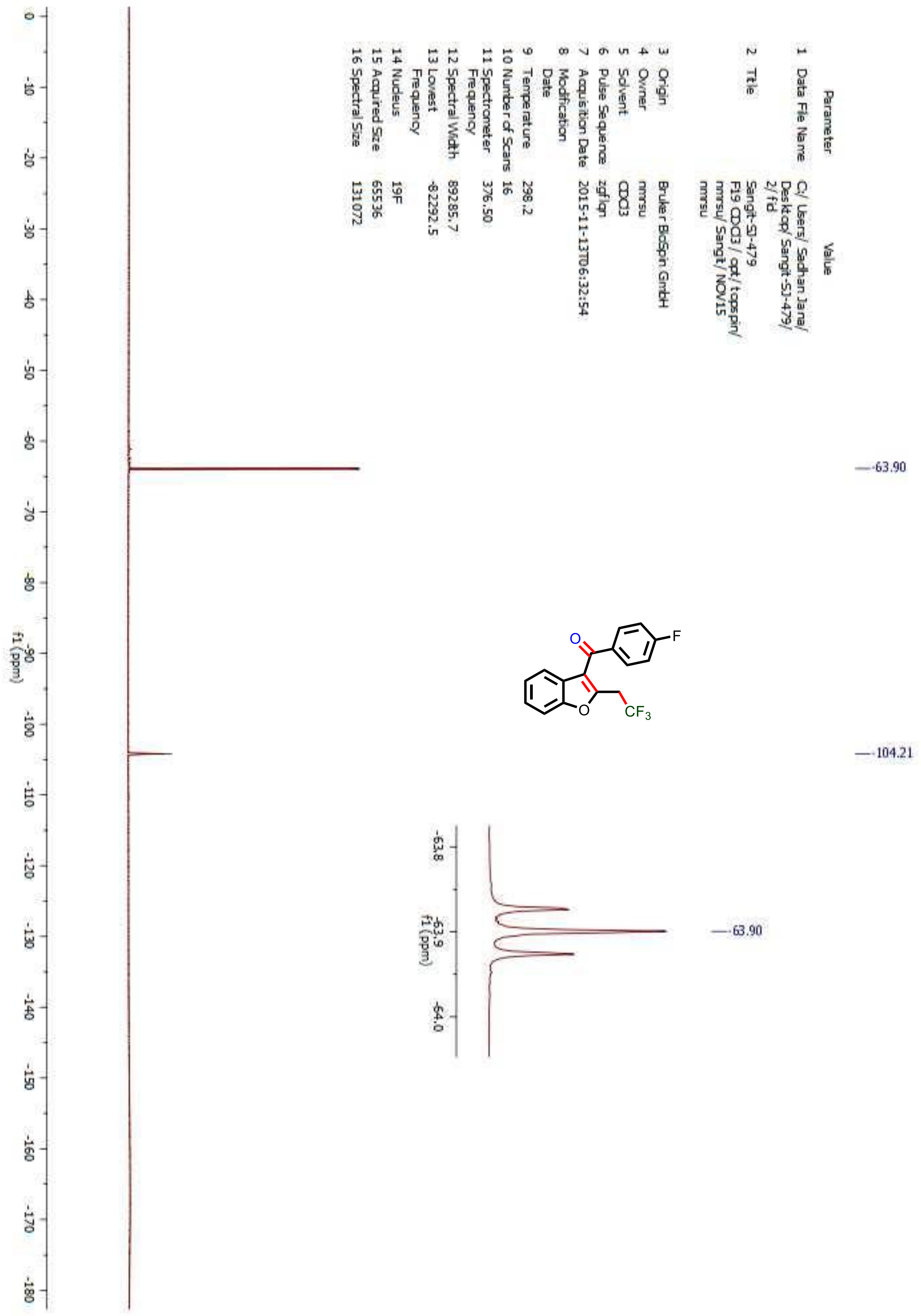

HRMS of (4-fluorophenyl)(2-(2,2,2-trifluoroethyl)benzofuran-3-yl)methanone (**2b**)

Display Report

Analysis Info

Analysis Name	D:\Data\user data\2015\NOV-2015\27-NOV-2015\Dr.S.Kumar-SJ-479_1-B,2_01_4356.d	Acquisition Date	11/27/2015 4:29:50 PM
Method	HRLCMS-20 Sept.m	Operator	RUCHI
Sample Name	Dr.S.Kumar-SJ-479	Instrument	micrOTOF-Q II 10330
Comment			

Acquisition Parameter

Source Type	ESI	Ion Polarity	Positive	Set Nebulizer	1.2 Bar
Focus	Active	Set Capillary	4500 V	Set Dry Heater	200 °C
Scan Begin	50 m/z	Set End Plate Offset	-500 V	Set Dry Gas	7.0 l/min
Scan End	3000 m/z	Set Collision Cell RF	130.0 Vpp	Set Divert Valve	Waste

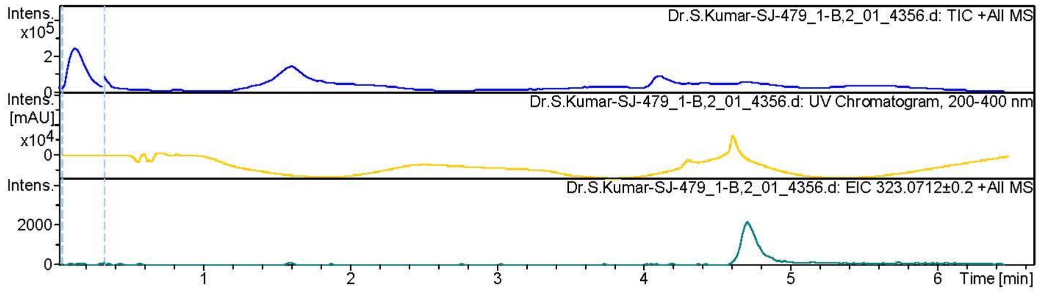

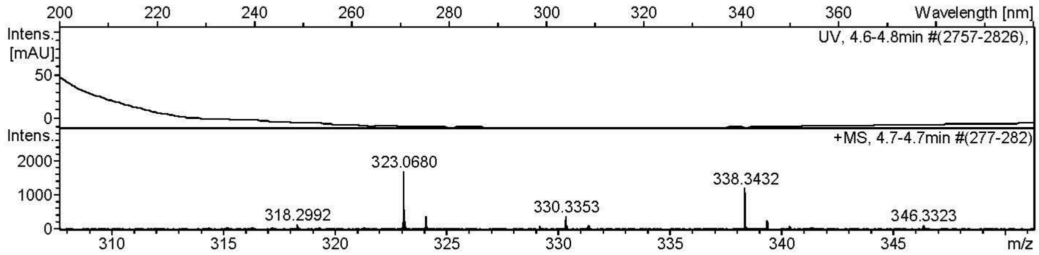

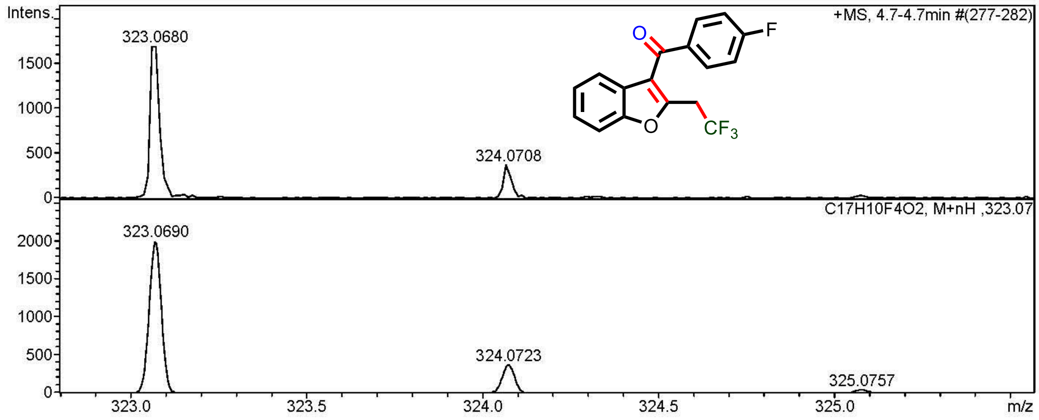

^1H of (4-hydroxyphenyl)(2-(2,2,2-trifluoroethyl)benzofuran-3-yl)methanone (**2c**)

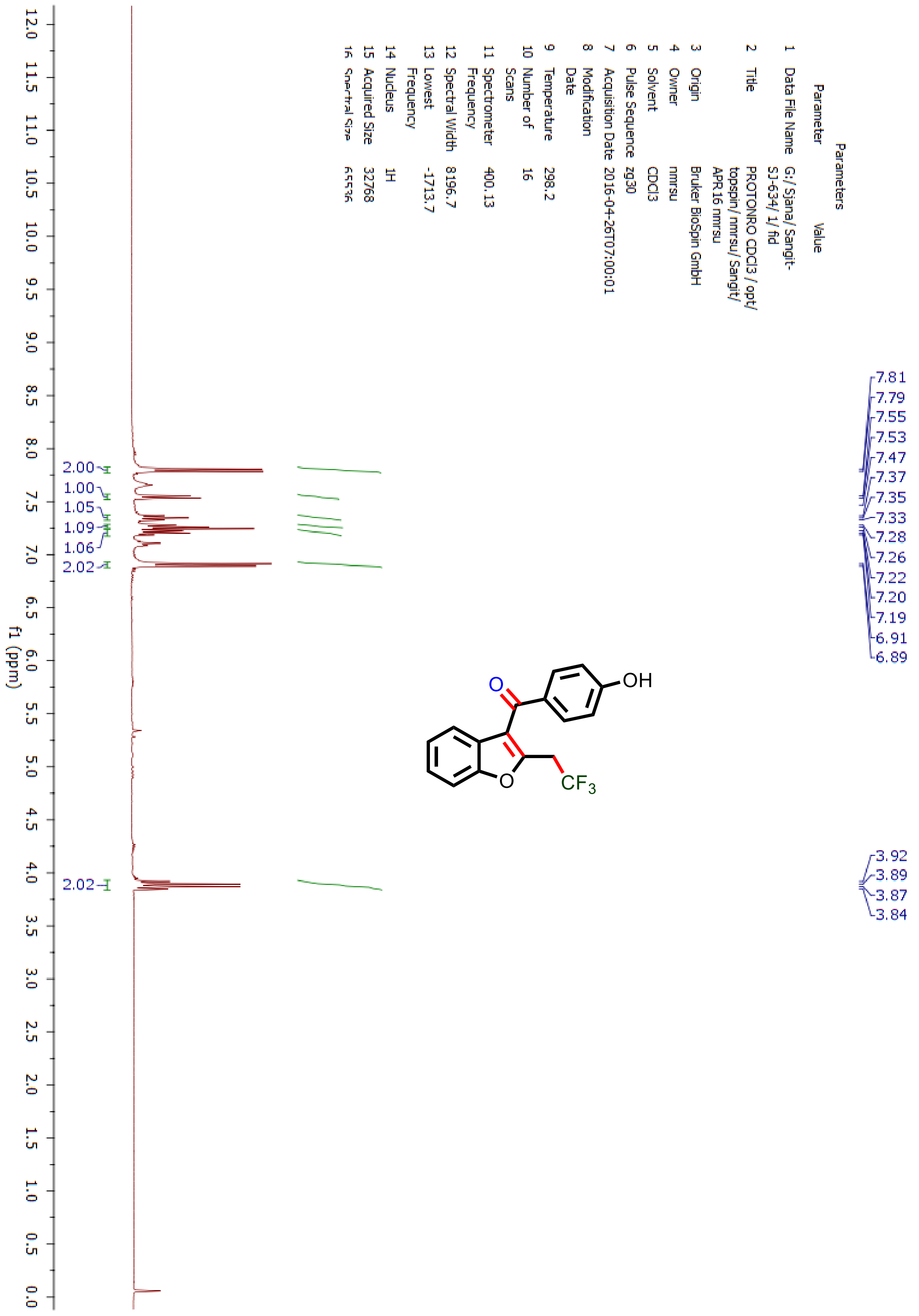

^{13}C of (4-hydroxyphenyl)(2-(2,2,2-trifluoroethyl)benzofuran-3-yl)methanone (**2c**)

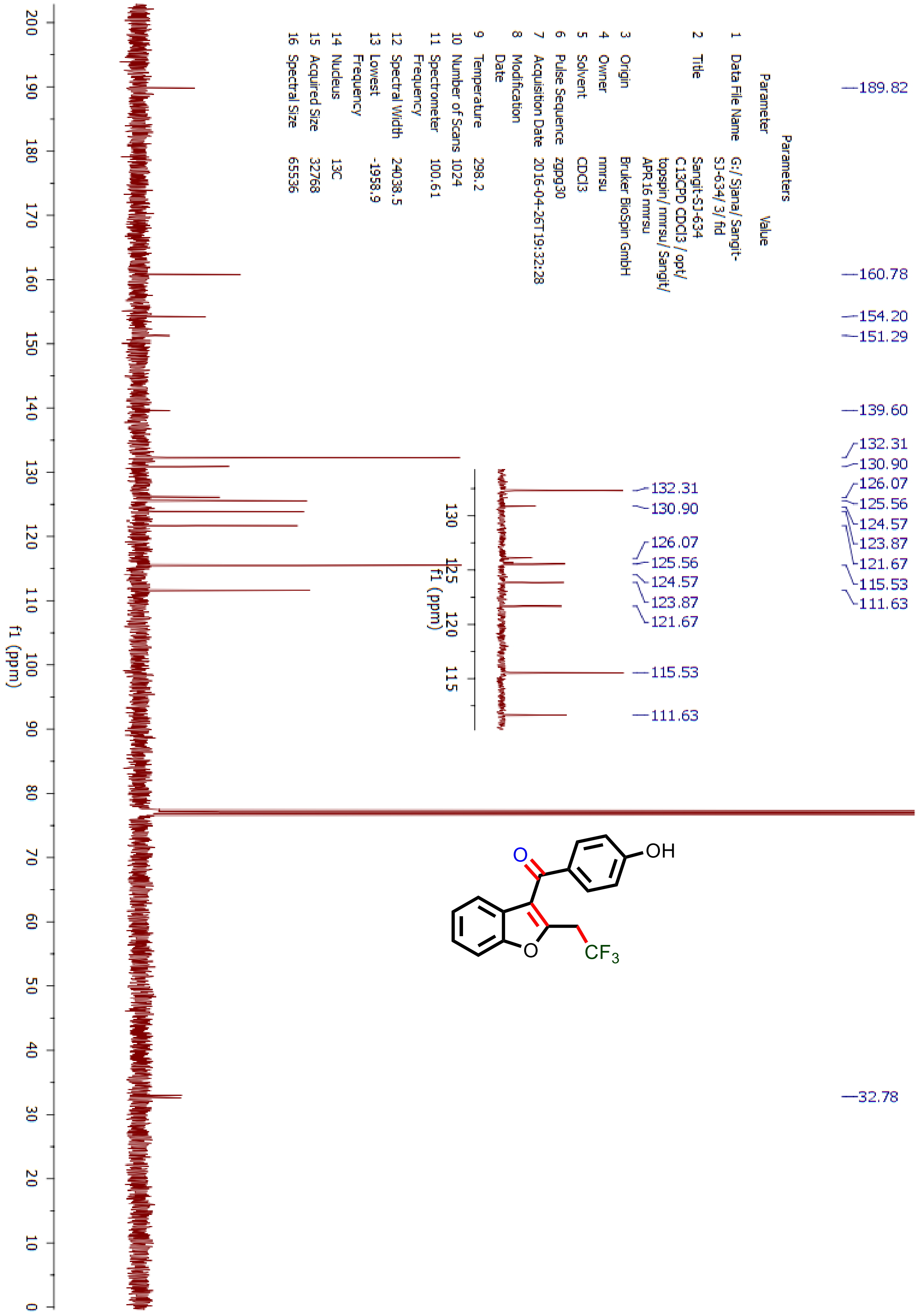

^{19}F of (4-hydroxyphenyl)(2-(2,2,2-trifluoroethyl)benzofuran-3-yl)methanone (**2c**)

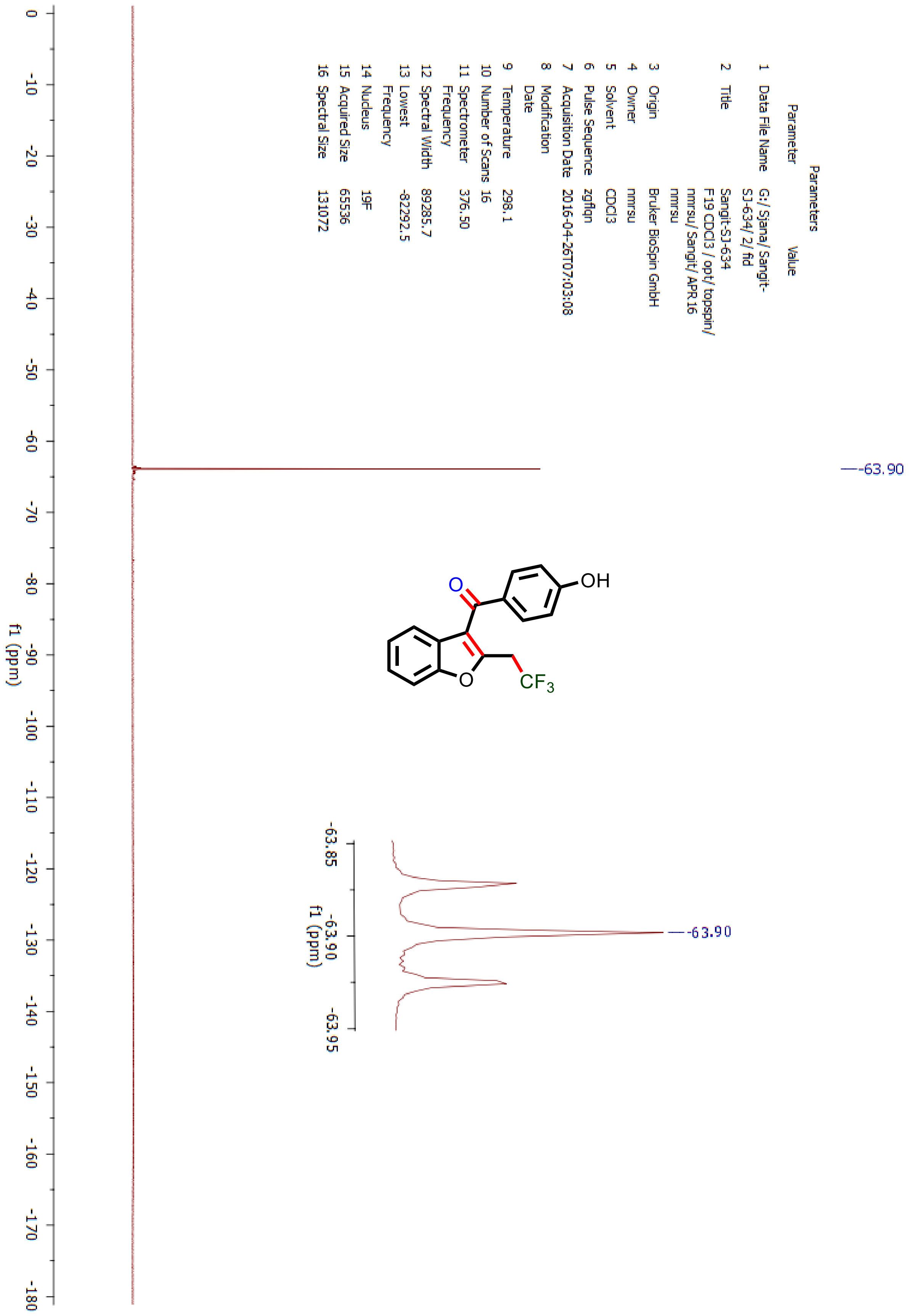

HRMS of (4-hydroxyphenyl)(2-(2,2,2-trifluoroethyl)benzofuran-3-yl)methanone (**2c**)

Display Report

Analysis Info

Analysis Name	D:\Data\user data\2016\SEPT-2016\16-09-2016\Dr S Kumar-SJ-634_1-A,4_01_7410.d	Acquisition Date	9/16/2016 12:58:12 PM
Method	hrlcms_pos_mid_tunemix.m	Operator	DIMPLE
Sample Name	Dr S Kumar-SJ-634	Instrument	micrOTOF-Q II 10330
Comment			

Acquisition Parameter

Source Type	ESI	Ion Polarity	Positive	Set Nebulizer	0.3 Bar
Focus	Active	Set Capillary	4500 V	Set Dry Heater	200 °C
Scan Begin	50 m/z	Set End Plate Offset	-500 V	Set Dry Gas	4.0 l/min
Scan End	3000 m/z	Set Collision Cell RF	450.0 Vpp	Set Divert Valve	Waste

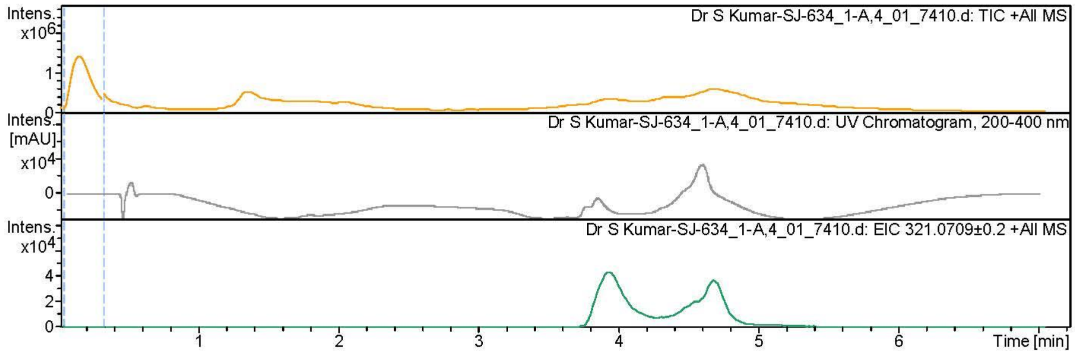

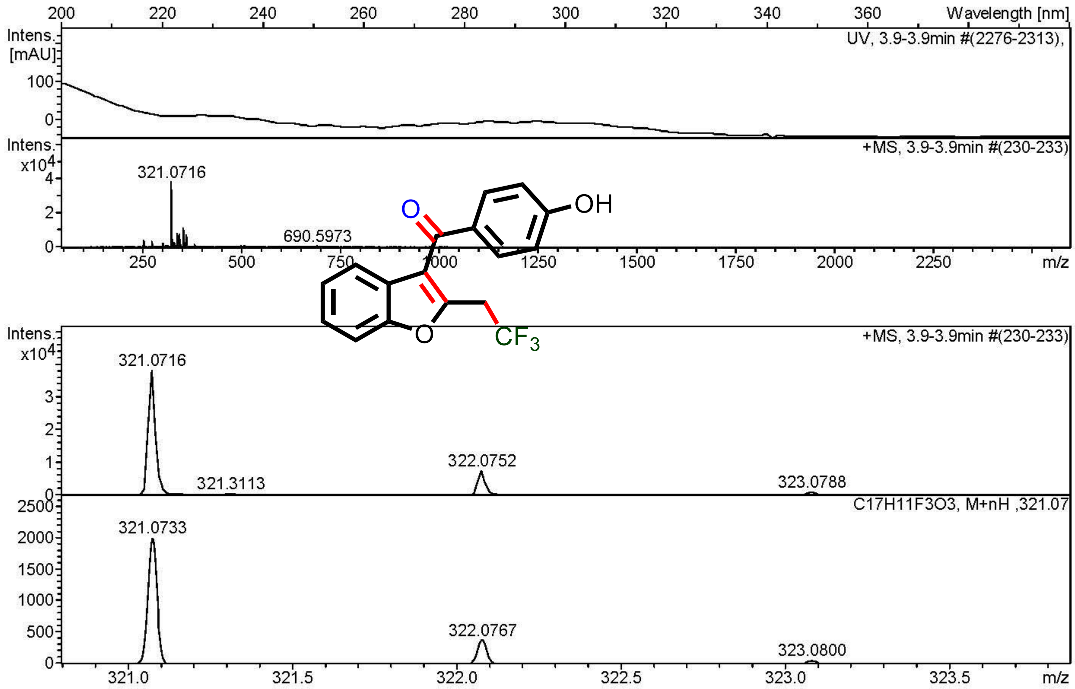

^{1}H NMR of (4-methoxyphenyl)(2-(2,2,2-trifluoroethyl)benzofuran-3-yl)methanone (**2d**)

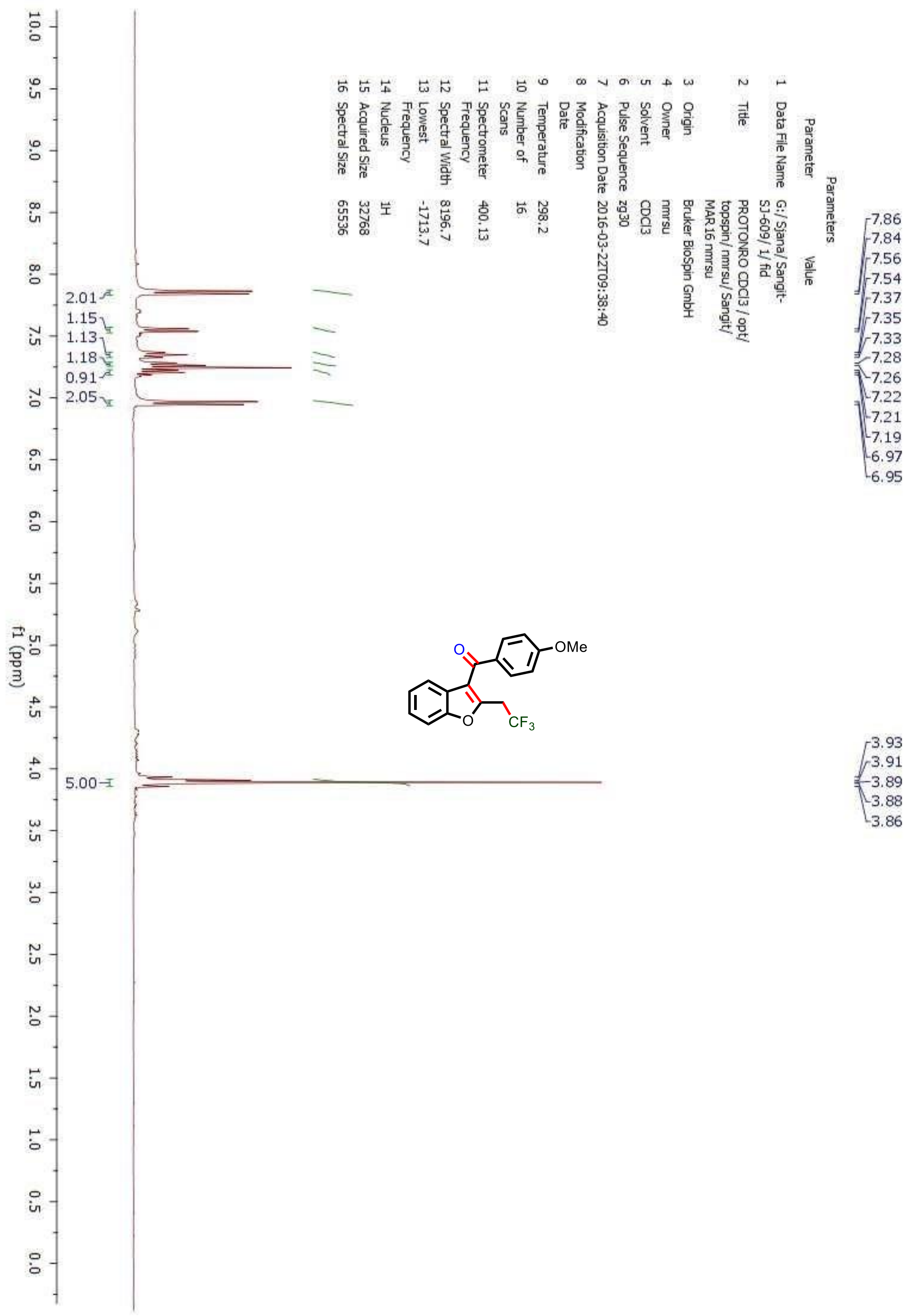

^{13}C NMR of (4-methoxyphenyl)(2-(2,2,2-trifluoroethyl)benzofuran-3-yl)methanone (**2d**)

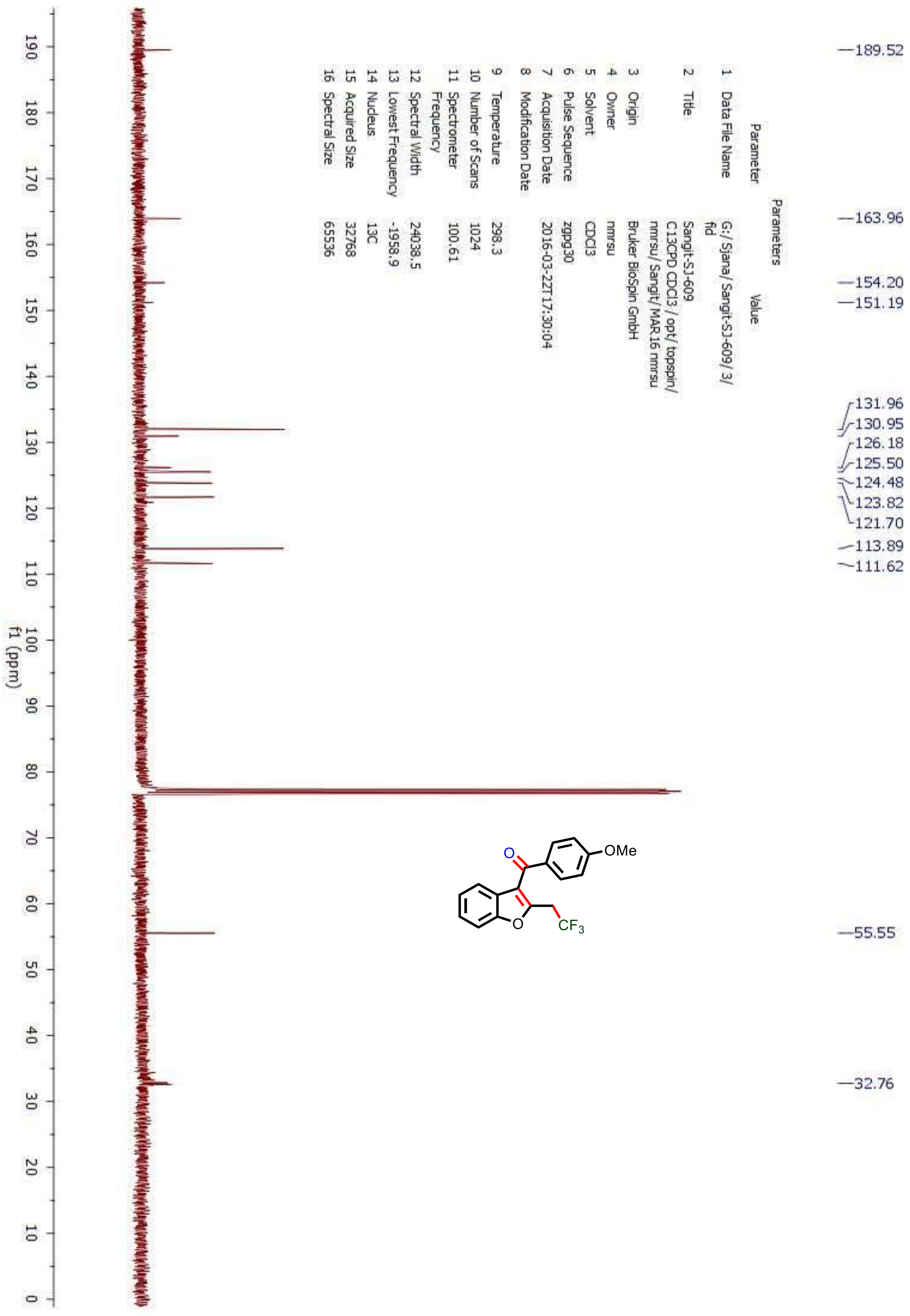

^{19}F NMR of (4-methoxyphenyl)(2-(2,2,2-trifluoroethyl)benzofuran-3-yl)methanone (**2d**)

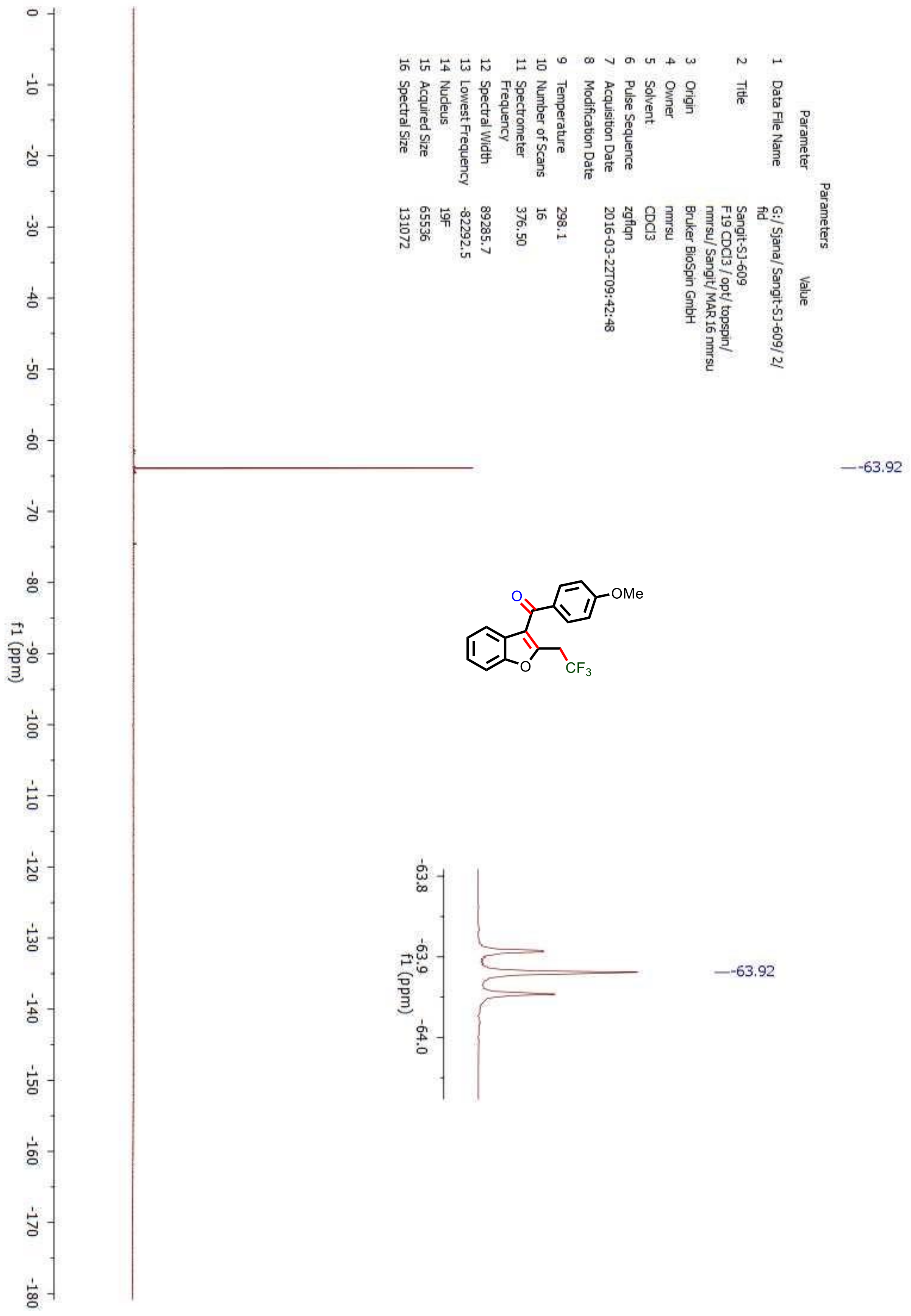

HRMS of (4-methoxyphenyl)(2-(2,2,2-trifluoroethyl)benzofuran-3-yl)methanone (**2d**)

Display Report

Analysis Info

Analysis Name	D:\Data\user data\2016\SEPT-2016\16-09-2016\Dr S Kumar-SJ-609_1-A,2_01_7408.d	Acquisition Date	9/16/2016 12:41:54 PM
Method	hrlcms_pos_mid_tunemix.m	Operator	DIMPLE
Sample Name	Dr S Kumar-SJ-609	Instrument	micrOTOF-Q II 10330
Comment			

Acquisition Parameter

Source Type	ESI	Ion Polarity	Positive	Set Nebulizer	0.3 Bar
Focus	Active	Set Capillary	4500 V	Set Dry Heater	200 °C
Scan Begin	50 m/z	Set End Plate Offset	-500 V	Set Dry Gas	4.0 l/min
Scan End	3000 m/z	Set Collision Cell RF	450.0 Vpp	Set Divert Valve	Waste

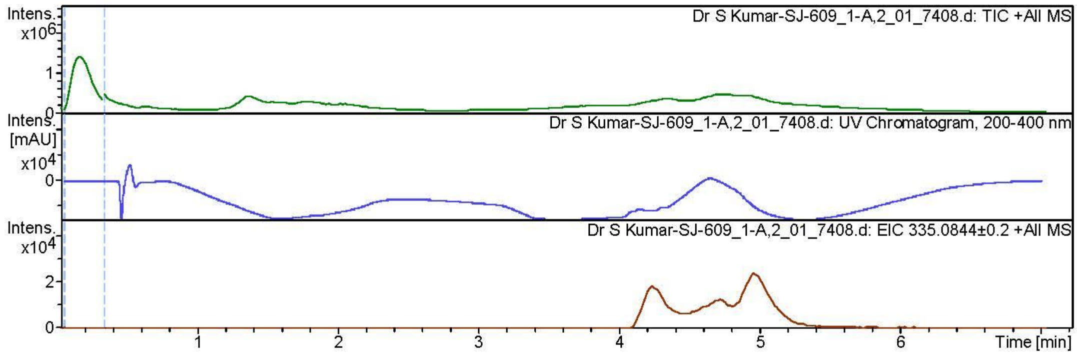

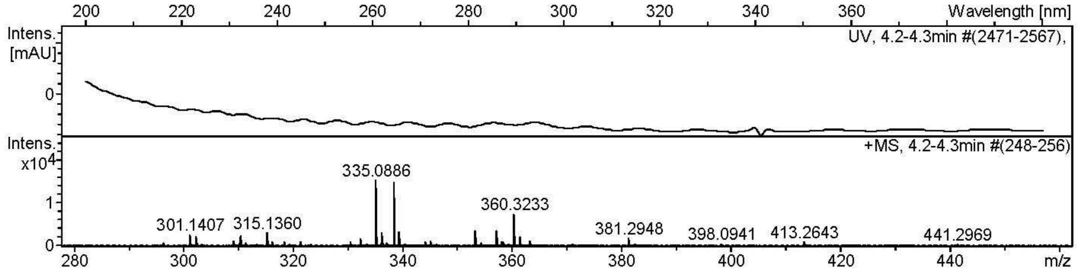

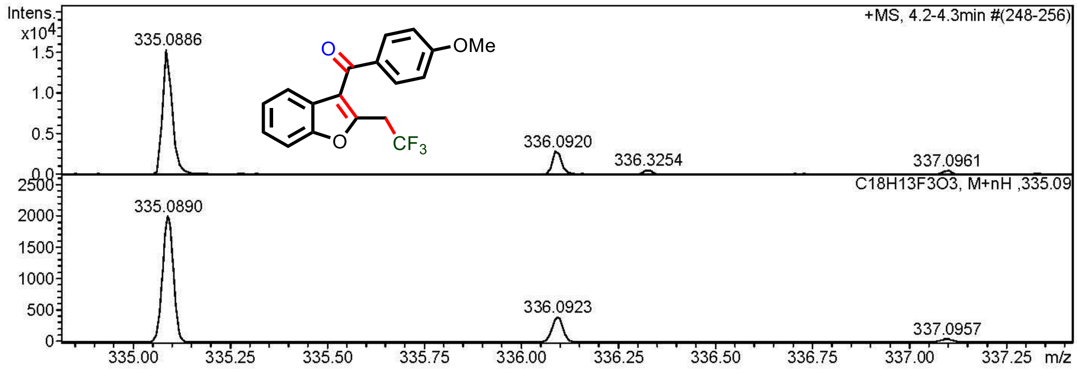

^{1}H NMR of (3-chlorophenyl)(2-(2,2,2-trifluoroethyl)benzofuran-3-yl)methanone (**2e**)

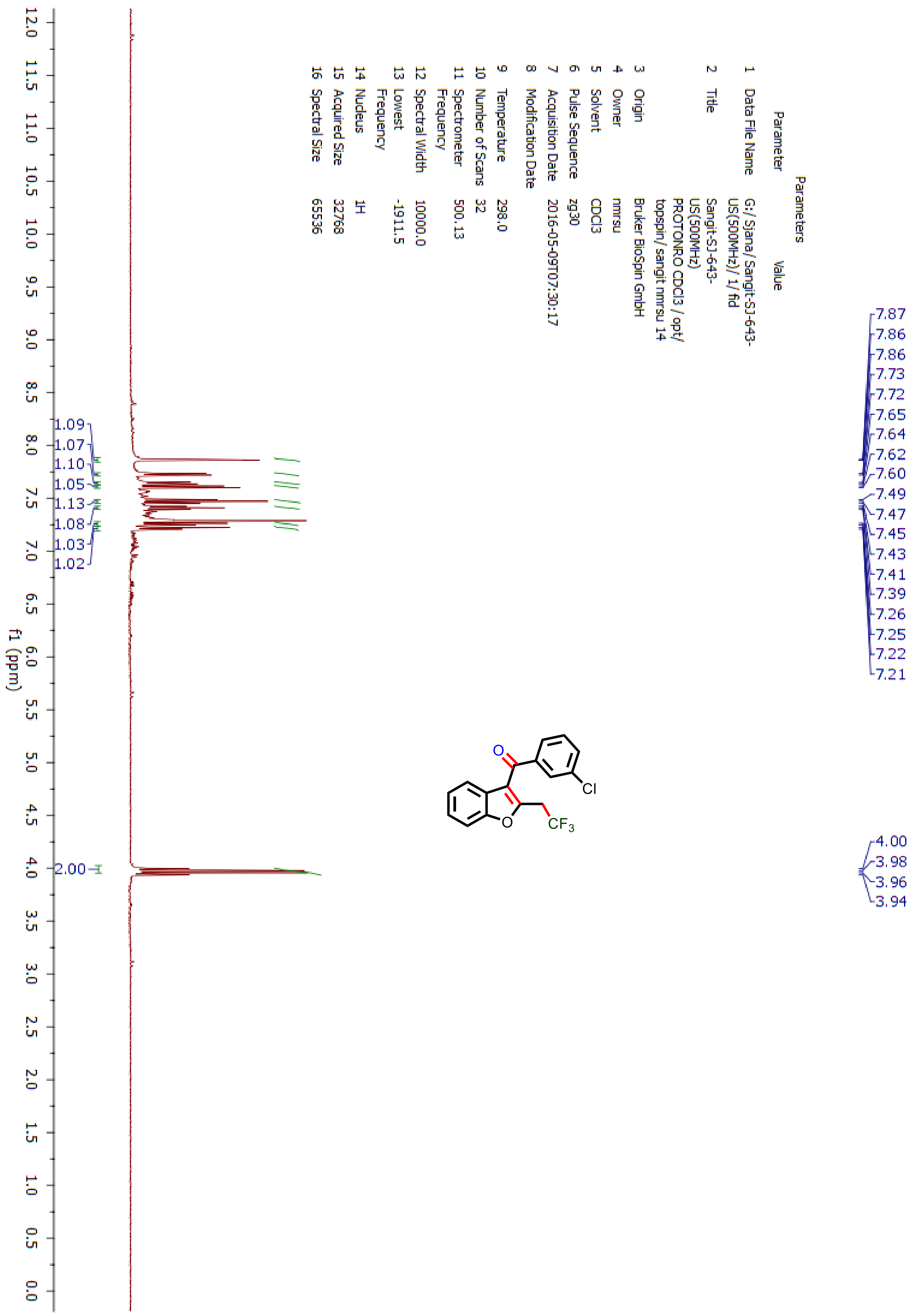

^{13}C NMR of (3-chlorophenyl)(2-(2,2,2-trifluoroethyl)benzofuran-3-yl)methanone (**2e**)

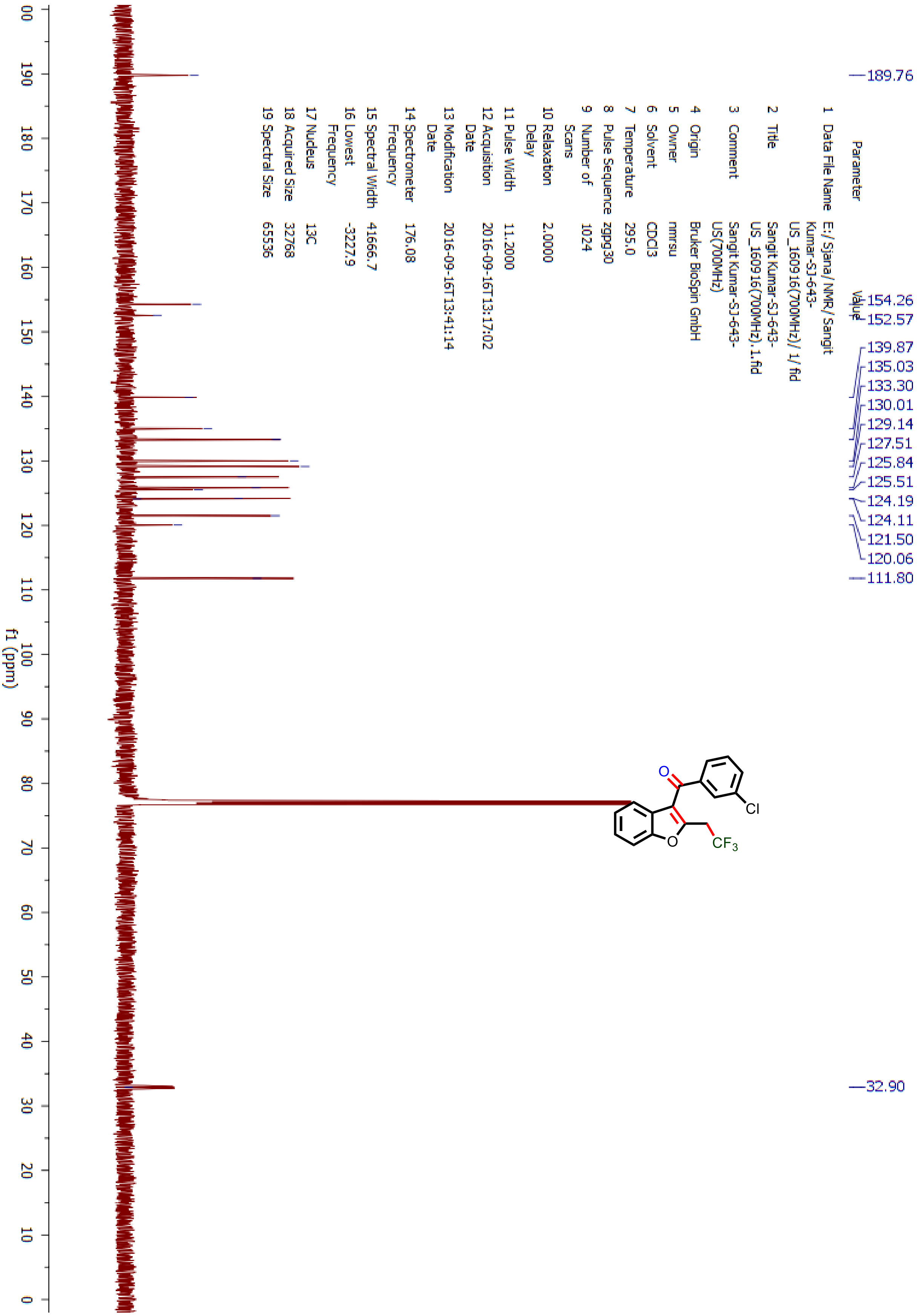

^{19}F NMR of (3-chlorophenyl)(2-(2,2,2-trifluoroethyl)benzofuran-3-yl)methanone (**2e**)

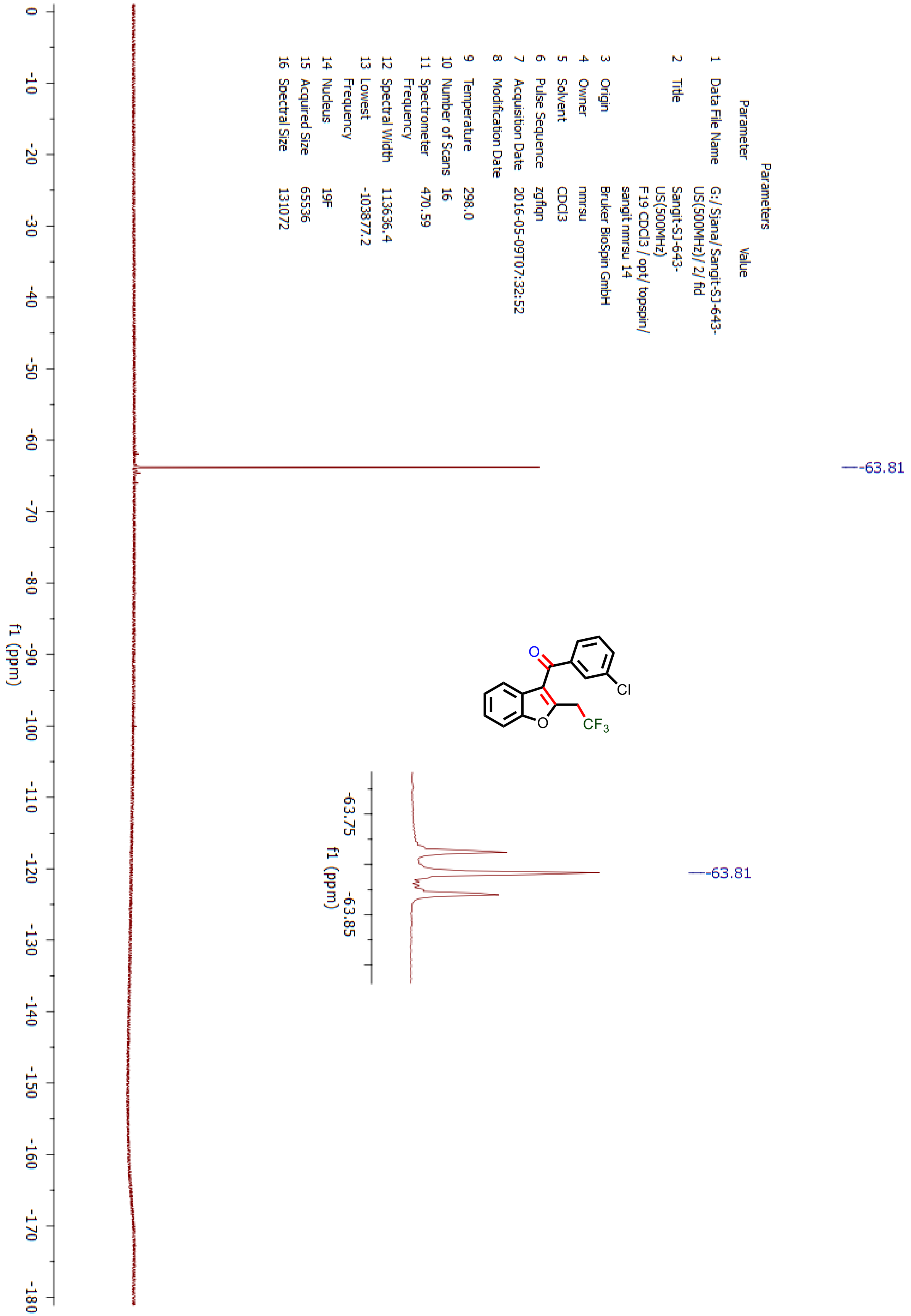

HRMS of (3-chlorophenyl)(2-(2,2,2-trifluoroethyl)benzofuran-3-yl)methanone (**2e**)

Display Report

Analysis Info

		Acquisition Date	9/22/2016 3:44:24 PM
Analysis Name	D:\Data\user data\2016\SEPT-2016\22-sep-2016\Dr S.Kumar-SJ-643-US.d		
Method	tune_low_APCI.m	Operator	DIMPLE
Sample Name	SJ-643-US	Instrument	micrOTOF-Q II 10330
Comment			

Acquisition Parameter

Source Type	APCI	Ion Polarity	Positive	Set Nebulizer	3.1 Bar
Focus	Not active	Set Capillary	4500 V	Set Dry Heater	200 °C
Scan Begin	50 m/z	Set End Plate Offset	-500 V	Set Dry Gas	8.0 l/min
Scan End	3000 m/z	Set Collision Cell RF	130.0 Vpp	Set Divert Valve	Waste

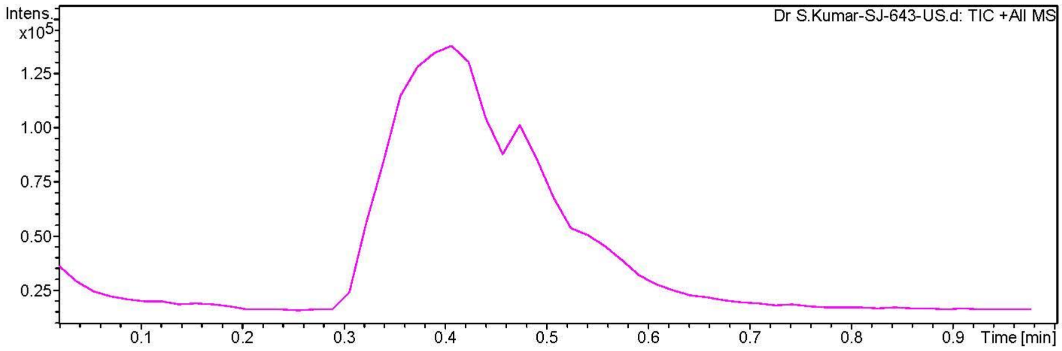

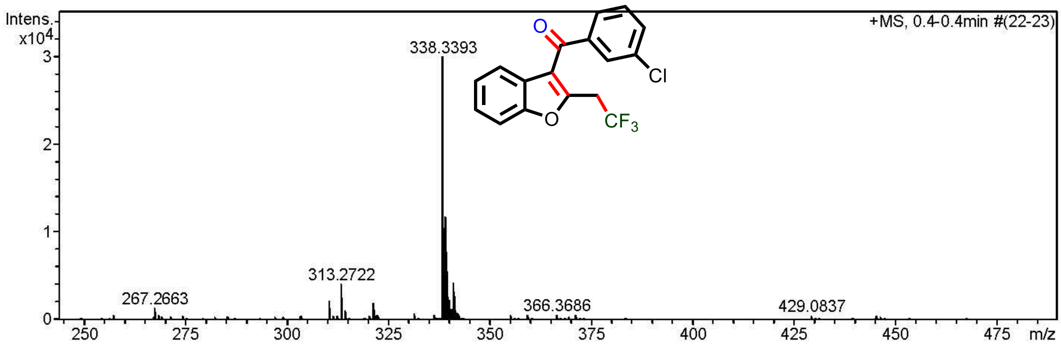

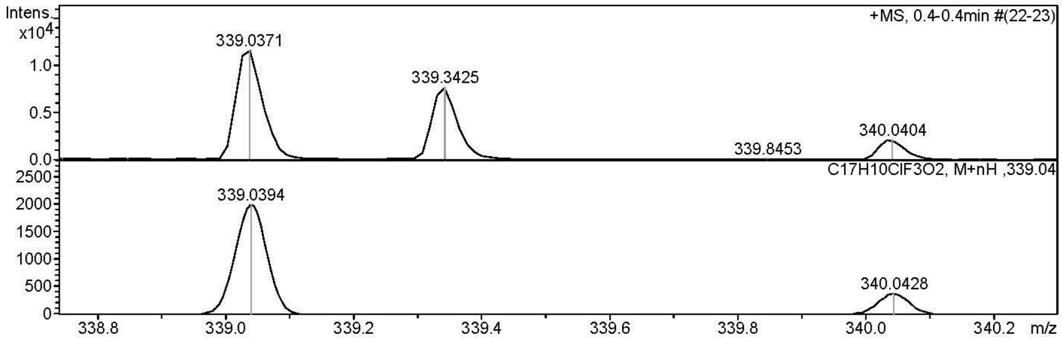

Bruker Compass DataAnalysis 4.0	printed:	9/22/2016 3:46:37 PM	Page 1 of 1

^{1}H NMR of m-tolyl(2-(2,2,2-trifluoroethyl)benzofuran-3-yl)methanone (**2f**)

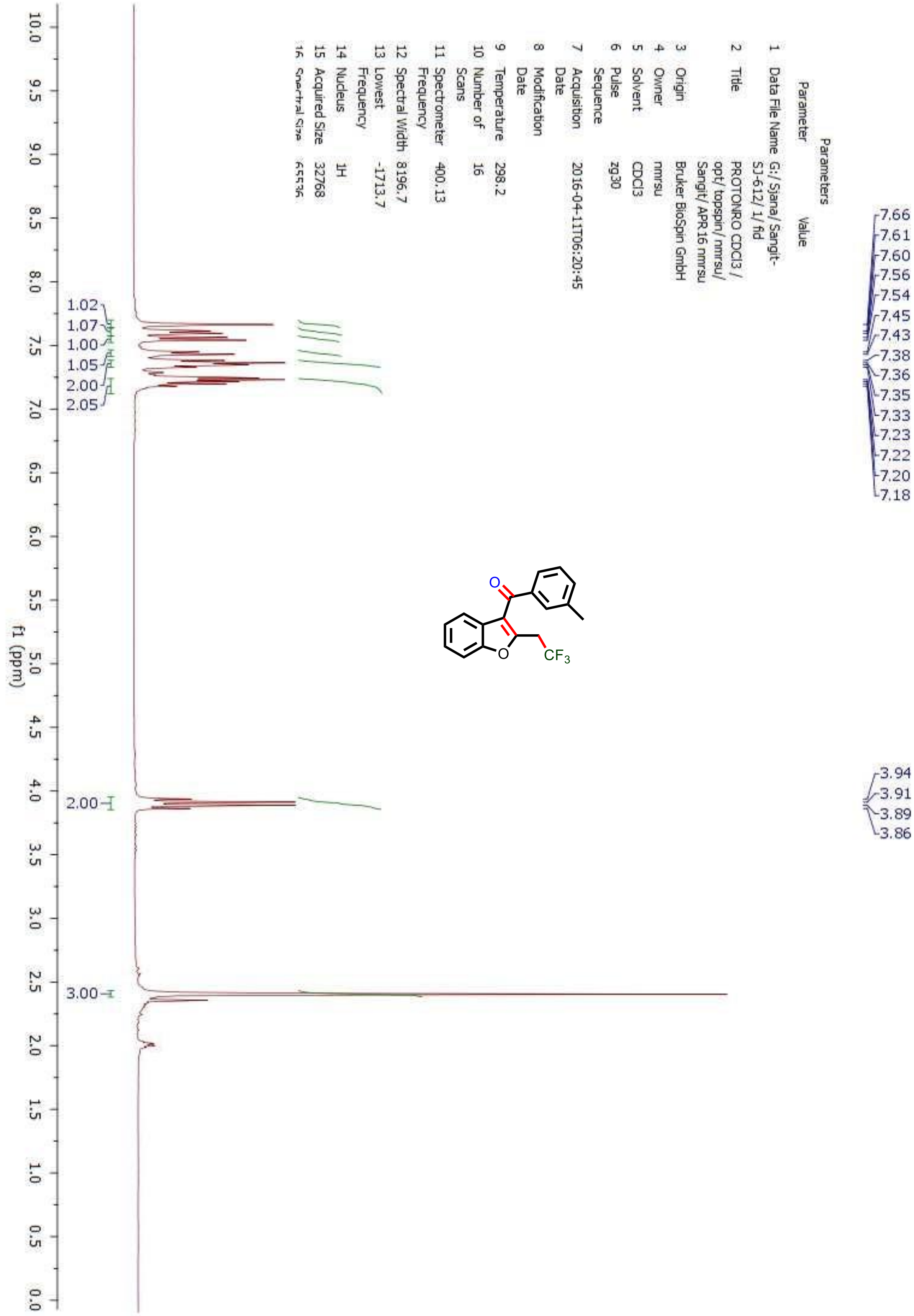

^{13}C NMR of m-tolyl(2-(2,2,2-trifluoroethyl)benzofuran-3-yl)methanone (**2f**)

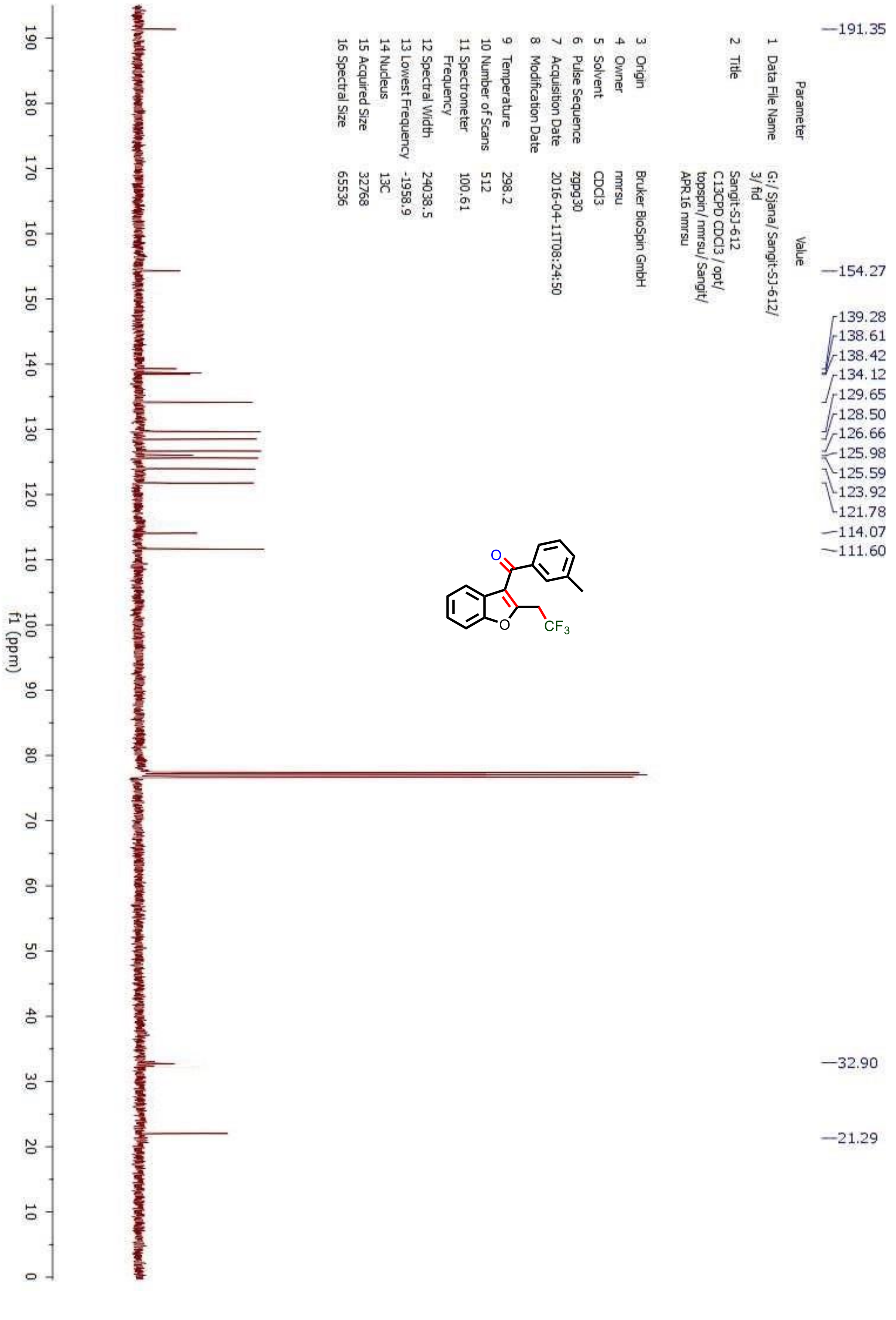

^{19}F NMR of m-tolyl(2-(2,2,2-trifluoroethyl)benzofuran-3-yl)methanone (**2f**)

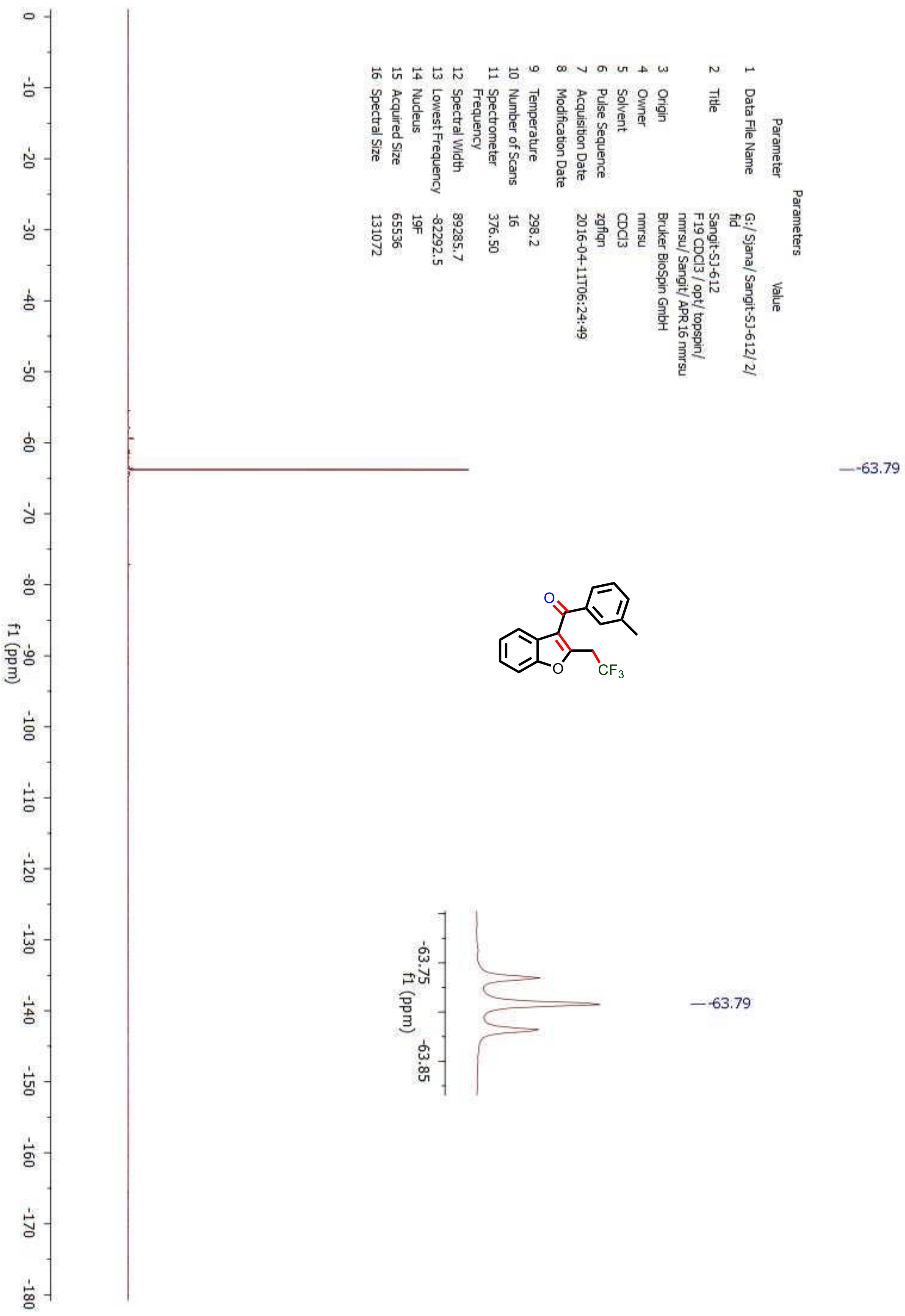

HRMS of m-tolyl(2-(2,2,2-trifluoroethyl)benzofuran-3-yl)methanone (**2f**)

Display Report

Analysis Info

		Acquisition Date	9/16/2016 1:06:22 PM
Analysis Name	D:\Data\user data\2016\SEPT-2016\16-09-2016\Dr S Kumar-SJ-634_1-A,5_01_7411.d		
Method	hrlcms_pos_mid_tunemix.m	Operator	DIMPLE
Sample Name	Dr S Kumar-SJ-634	Instrument	micrOTOF-Q II 10330
Comment			

Acquisition Parameter

Source Type	ESI	Ion Polarity	Positive	Set Nebulizer	0.3 Bar		
Focus	Active	Set Capillary	4500 V	Set Dry Heater	200 °C		
Scan Begin	50 m/z	Set End Plate Offset	-500 V	Set Dry Gas	4.0 l/min		
Scan End	3000 m/z	Set Collision Cell RF	450.0 Vpp	Set Divert Valve	Waste		

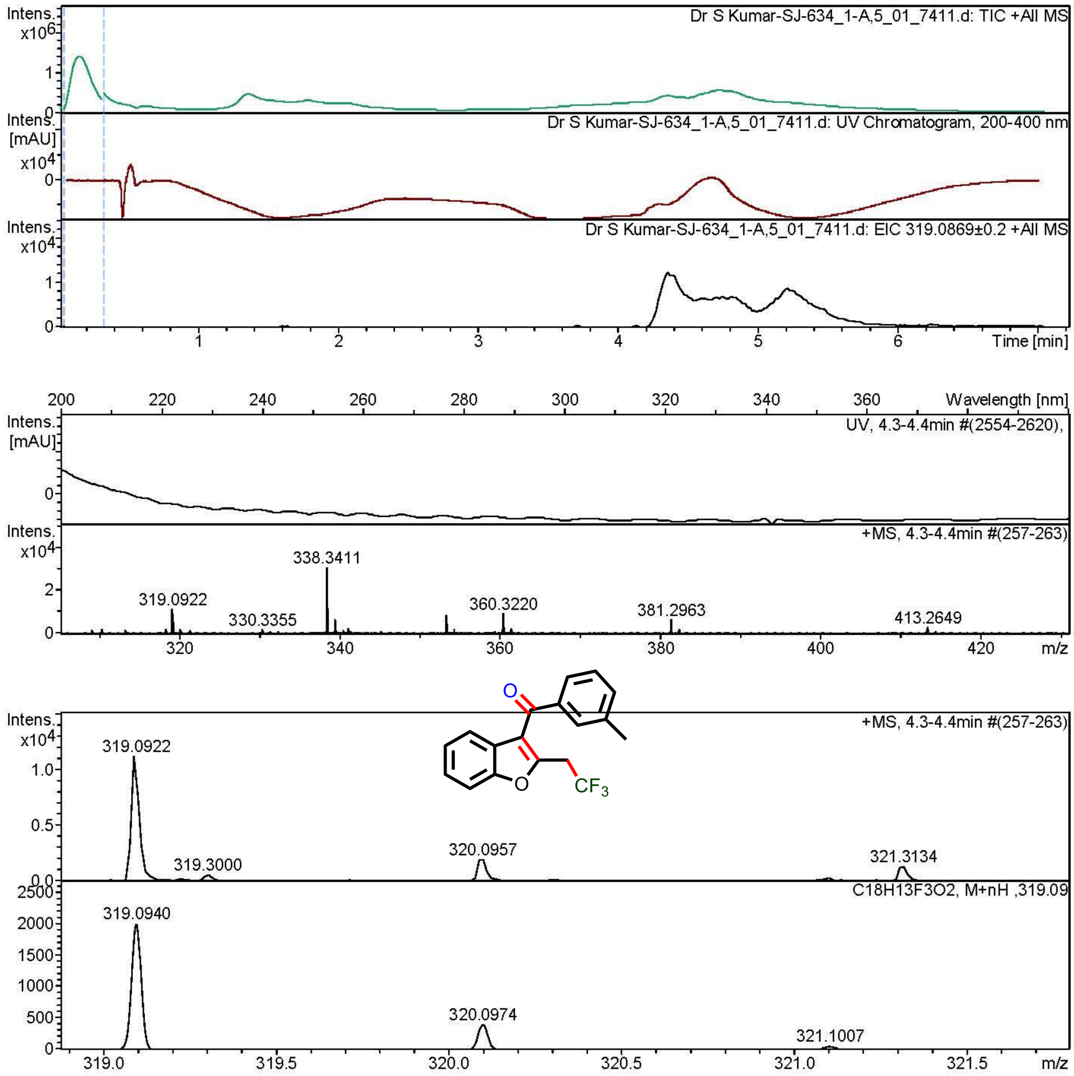

^{1}H NMR of (2-methoxyphenyl)(2-(2,2,2-trifluoroethyl)benzofuran-3-yl)methanone (**2g**)

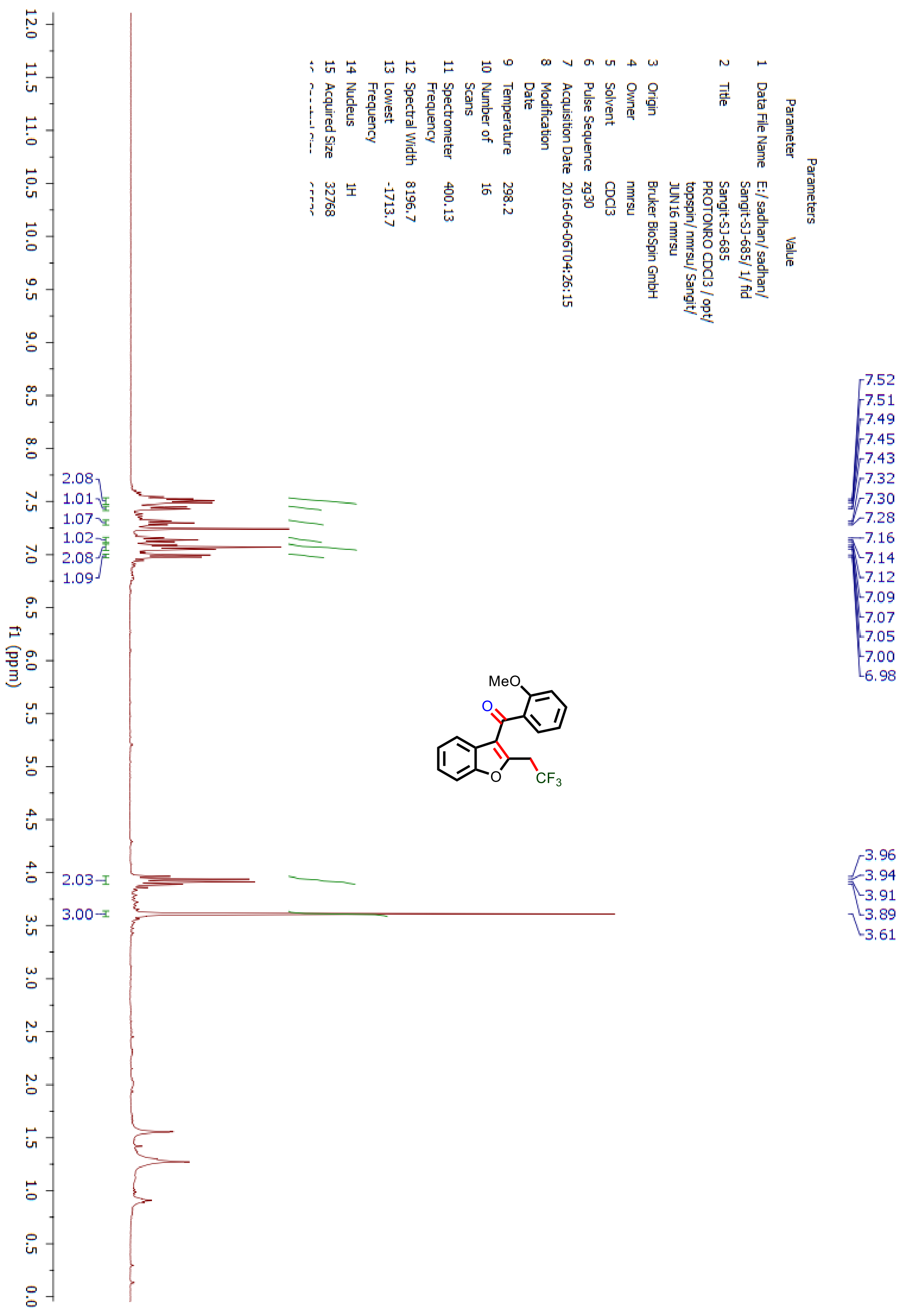

^{13}C NMR of (2-methoxyphenyl)(2-(2,2,2-trifluoroethyl)benzofuran-3-yl)methanone (**2g**)

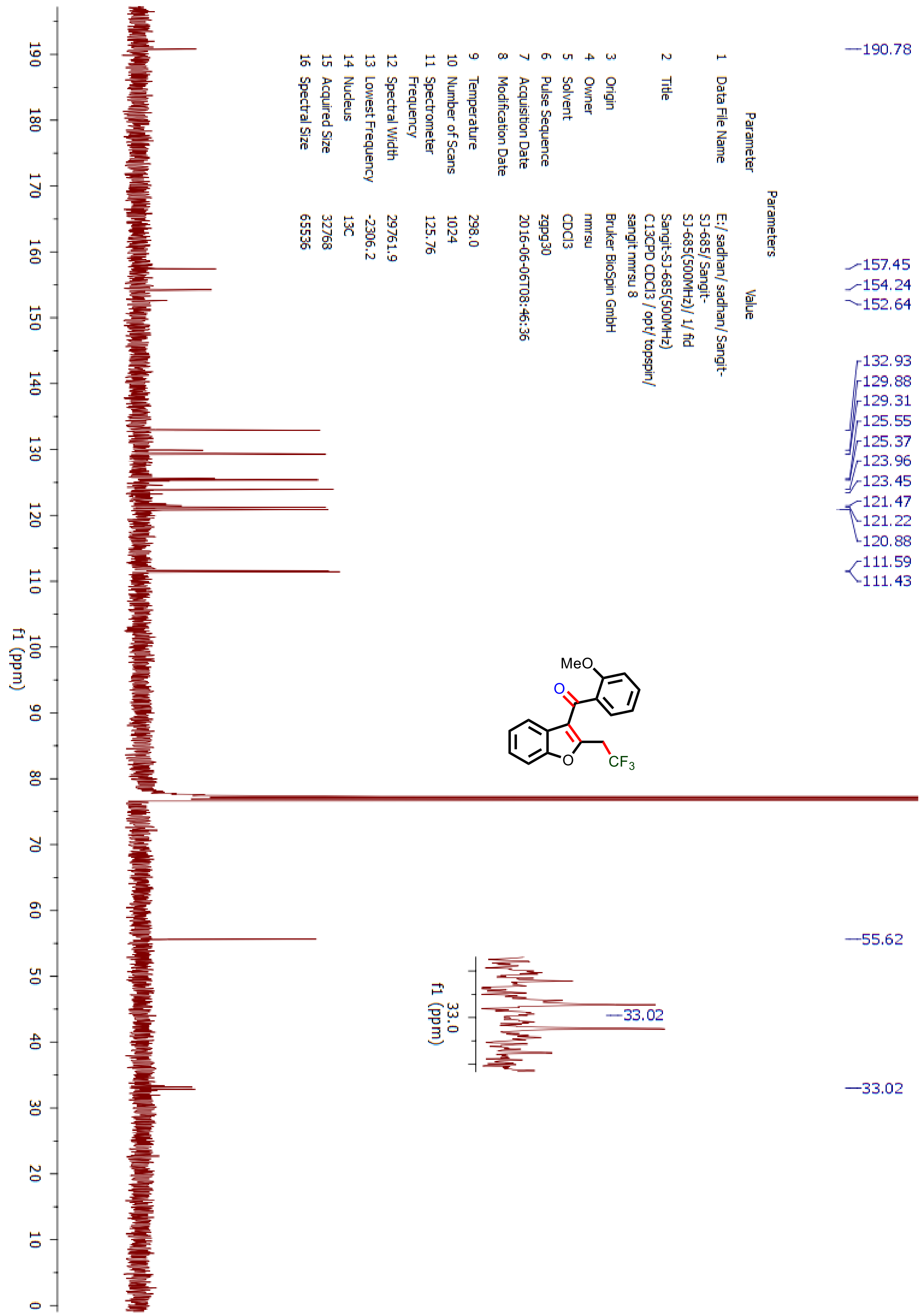

^{19}F NMR of (2-methoxyphenyl)(2-(2,2,2-trifluoroethyl)benzofuran-3-yl)methanone (**2g**)

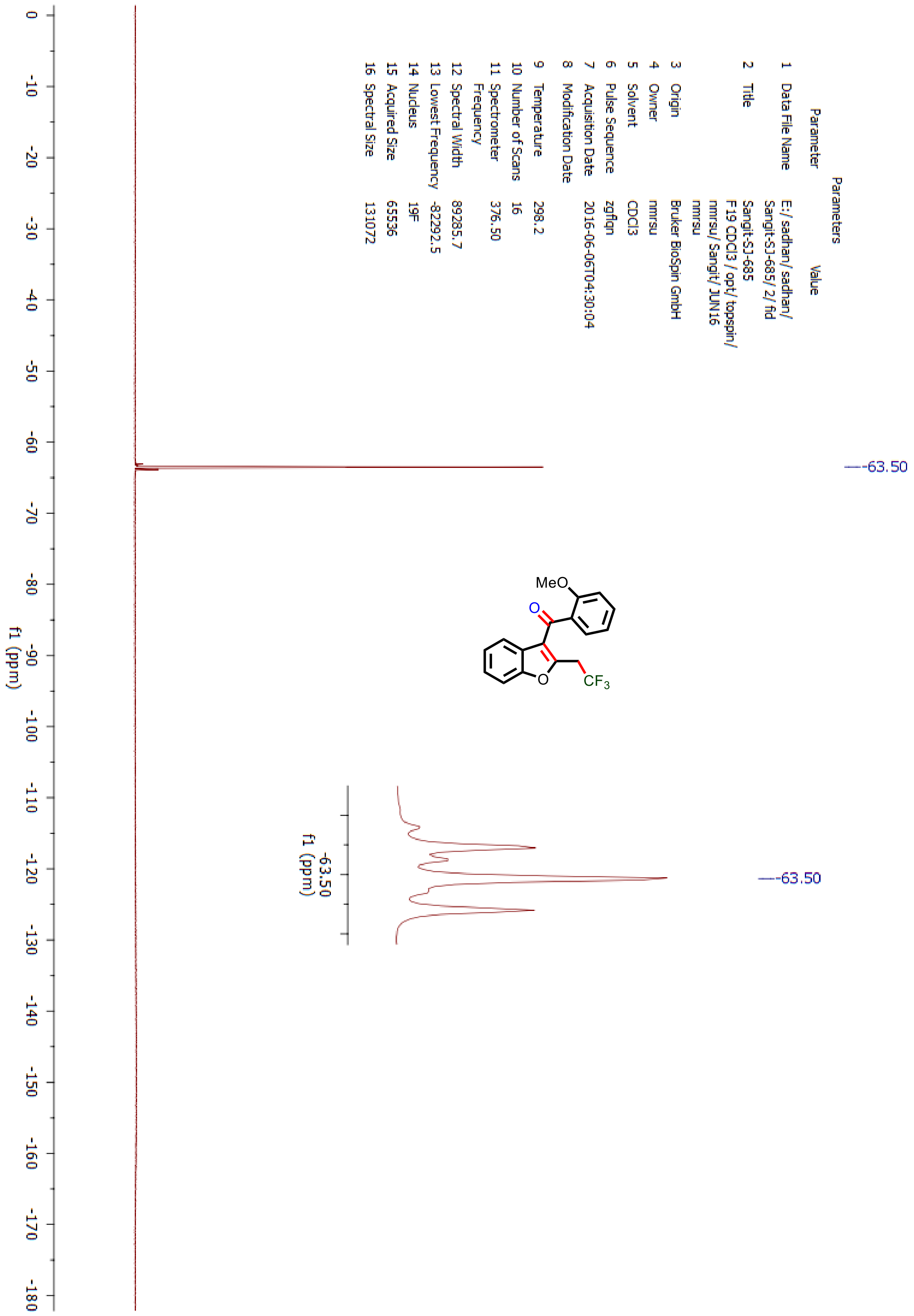

HRMS of (2-methoxyphenyl)(2-(2,2,2-trifluoroethyl)benzofuran-3-yl)methanone (**2g**)

Display Report

Analysis Info

Analysis Name	D:\Data\user data\2016\SEPT-2016\15-09-2016\Dr S Kumar-SJ-685_1-B,8_01_7394.d	Acquisition Date	9/15/2016 3:04:40 PM
Method	hrlcms_pos_mid_tunemix.m	Operator	DIMPLE
Sample Name	Dr S Kumar-SJ-685	Instrument	micrOTOF-Q II 10330
Comment			

Acquisition Parameter

Source Type	ESI	Ion Polarity	Positive	Set Nebulizer	0.3 Bar
Focus	Active	Set Capillary	4500 V	Set Dry Heater	200 °C
Scan Begin	50 m/z	Set End Plate Offset	-500 V	Set Dry Gas	4.0 l/min
Scan End	3000 m/z	Set Collision Cell RF	450.0 Vpp	Set Divert Valve	Waste

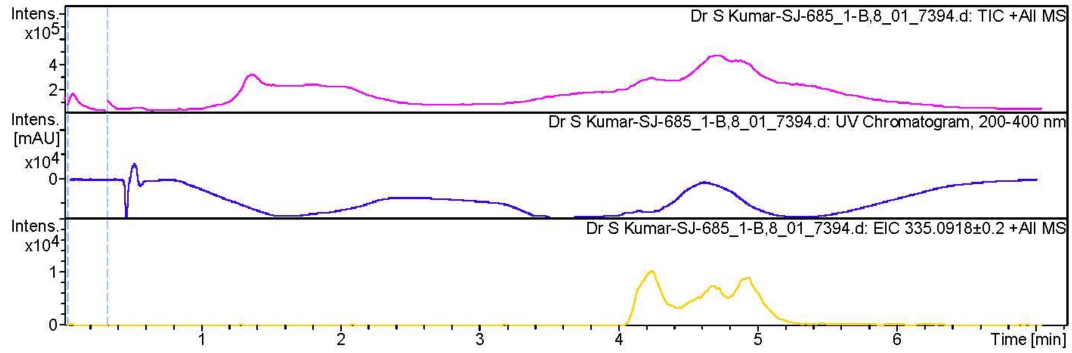

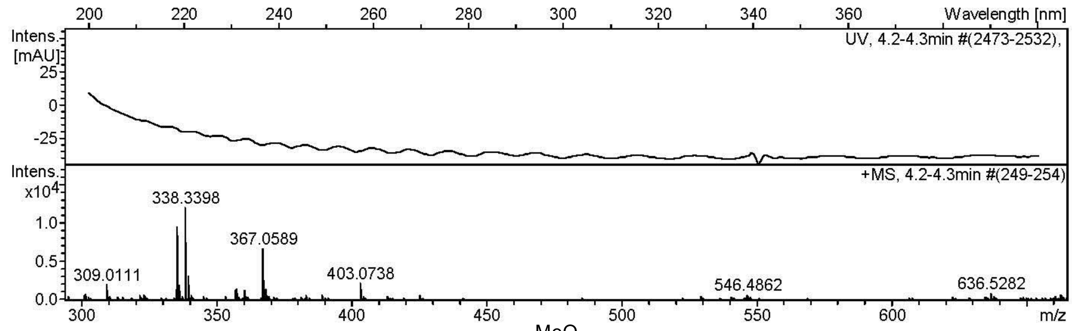

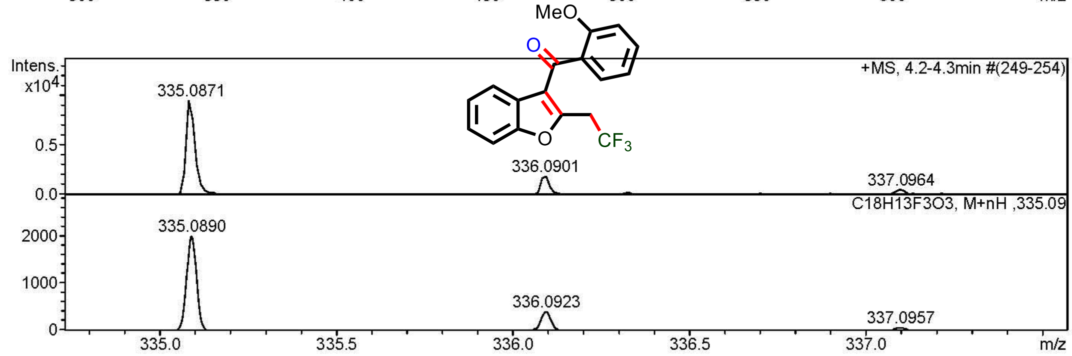

^{1}H NMR of (2-bromophenyl)(2-(2,2,2-trifluoroethyl)benzofuran-3-yl)methanone (**2h**)

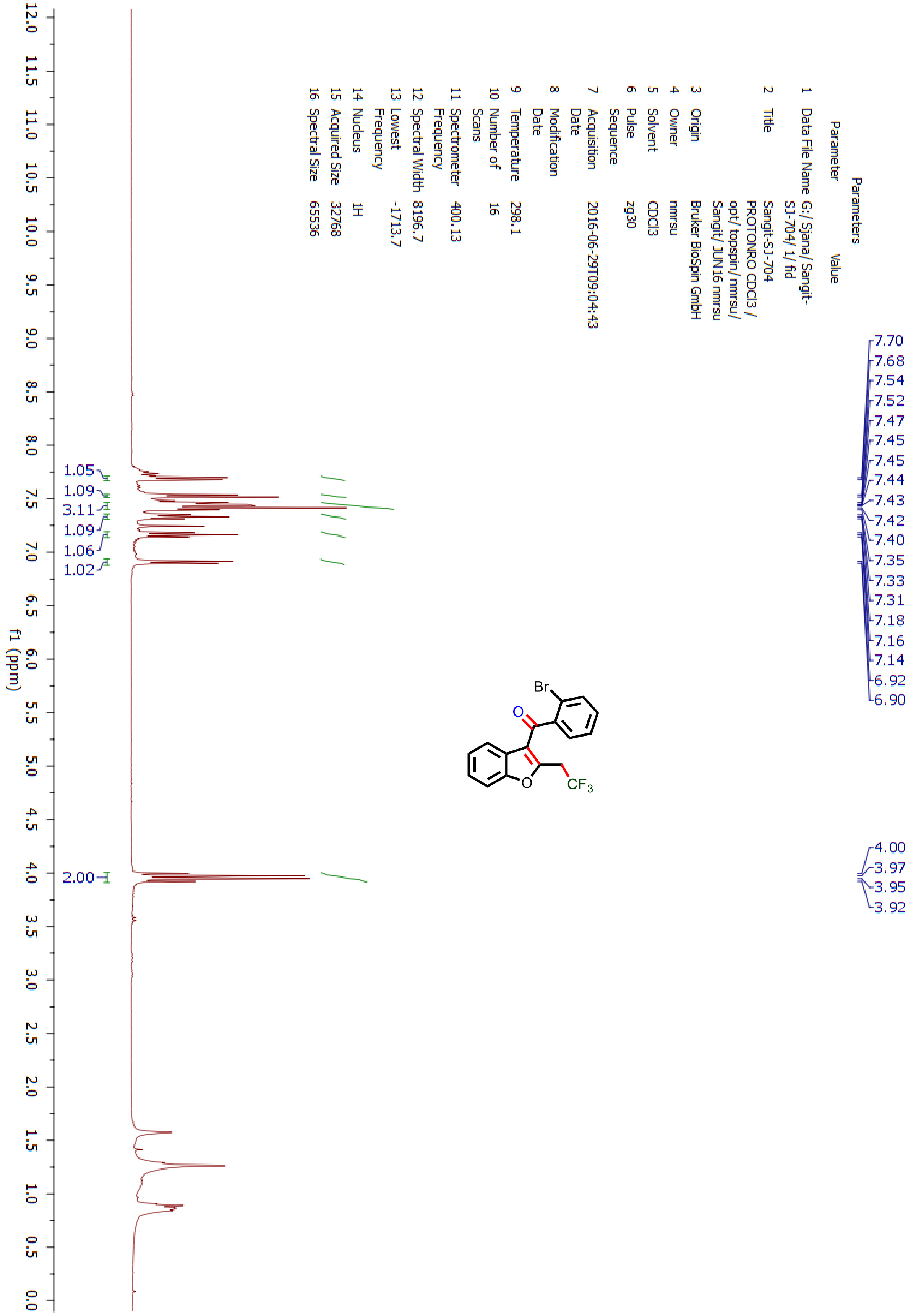

^{13}C NMR of (2-bromophenyl)(2-(2,2,2-trifluoroethyl)benzofuran-3-yl)methanone (**2h**)

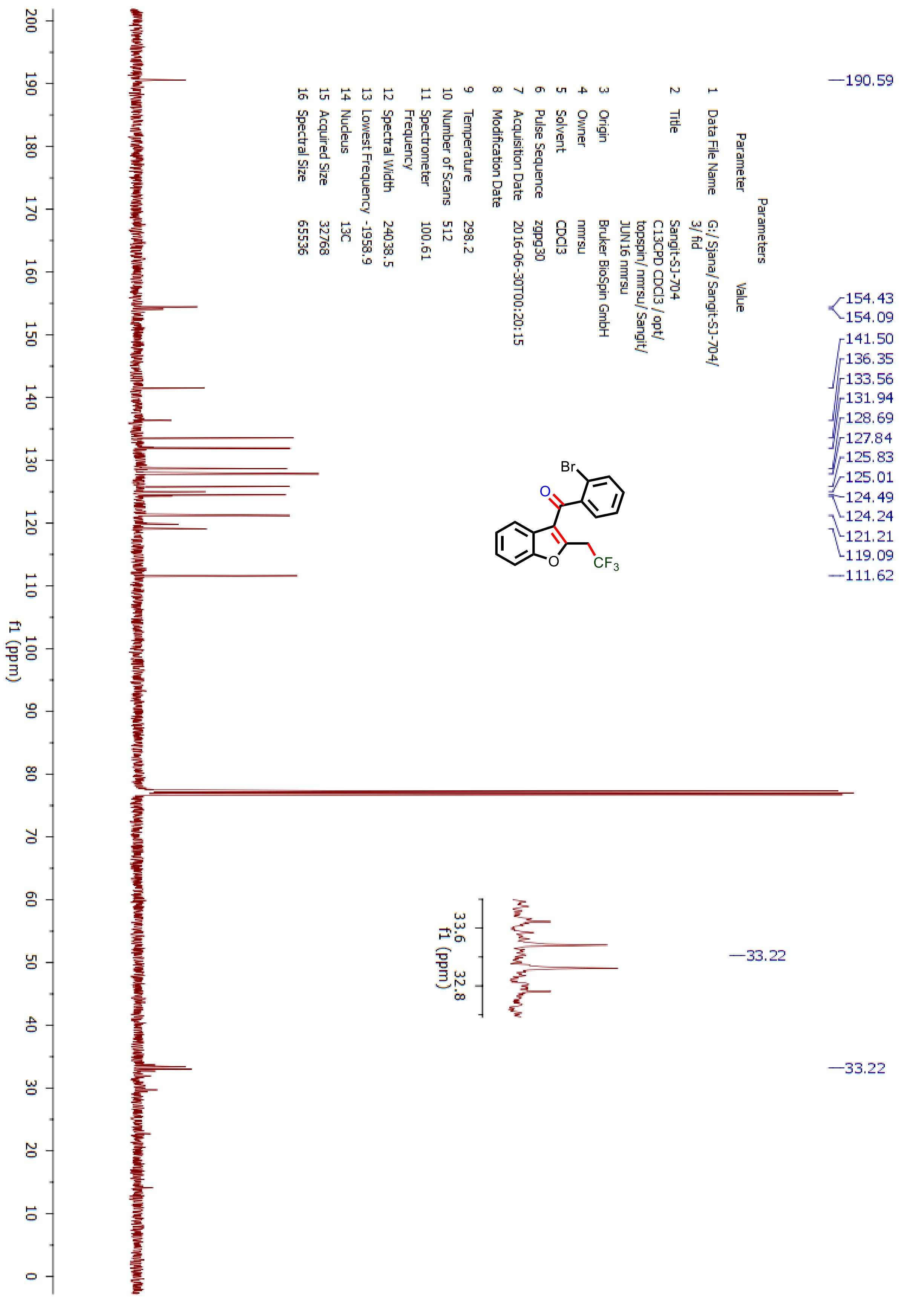

^{19}F NMR of (2-bromophenyl)(2-(2,2,2-trifluoroethyl)benzofuran-3-yl)methanone (**2h**)

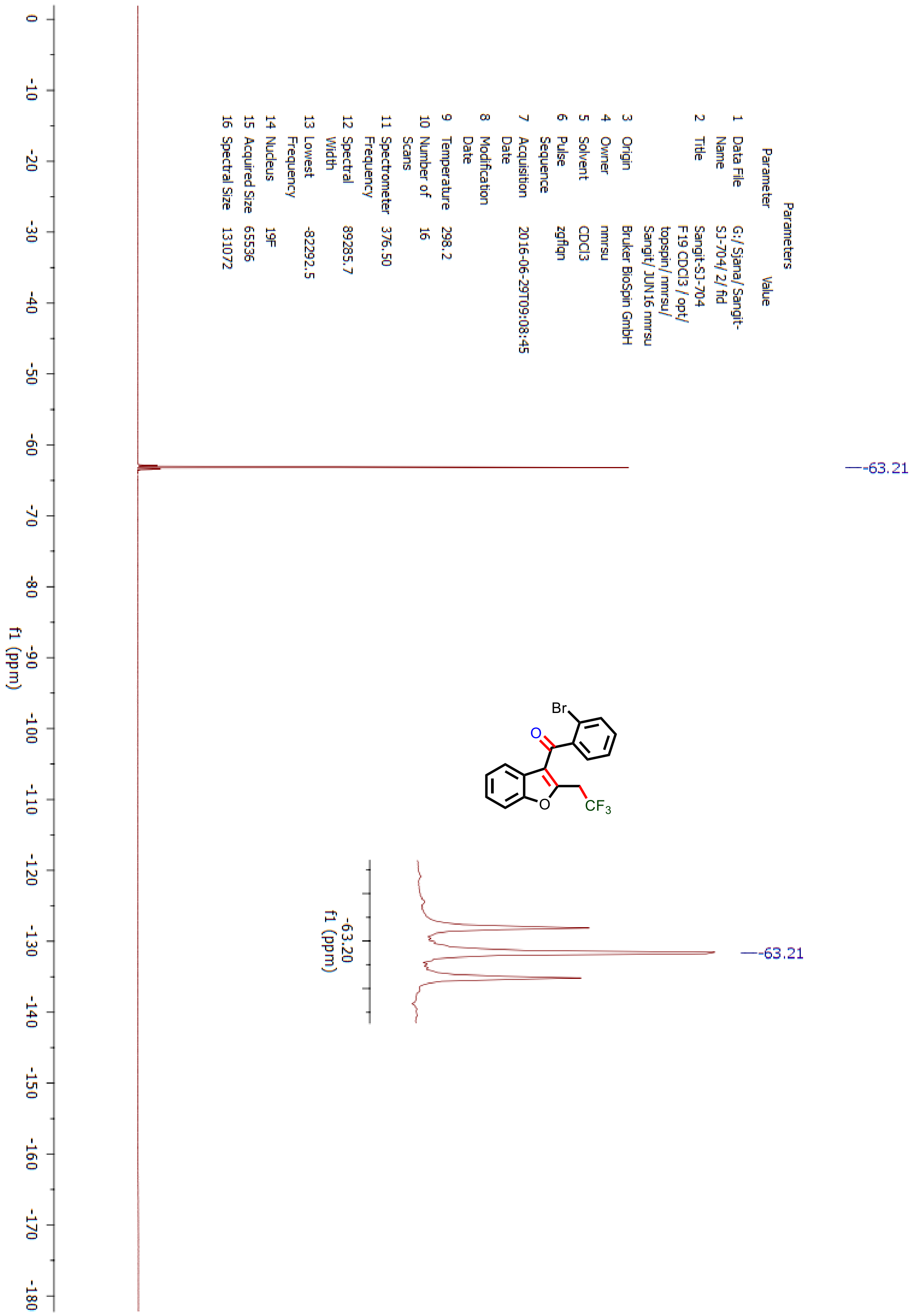

HRMS of (2-bromophenyl)(2-(2,2,2-trifluoroethyl)benzofuran-3-yl)methanone (**2h**)

Display Report

Analysis Info

Analysis Name	D:\Data\user data\2016\SEPT-2016\15-09-2016\Dr S Kumar-SJ-704_1-C,2_01_7397.d	Acquisition Date	9/15/2016 3:29:14 PM
Method	hrlcms_pos_mid_tunemix.m	Operator	DIMPLE
Sample Name	Dr S Kumar-SJ-704	Instrument	micrOTOF-Q II 10330
Comment			

Acquisition Parameter

Source Type	ESI	Ion Polarity	Positive	Set Nebulizer	0.3 Bar
Focus	Active	Set Capillary	4500 V	Set Dry Heater	200 °C
Scan Begin	50 m/z	Set End Plate Offset	-500 V	Set Dry Gas	4.0 l/min
Scan End	3000 m/z	Set Collision Cell RF	450.0 Vpp	Set Divert Valve	Waste

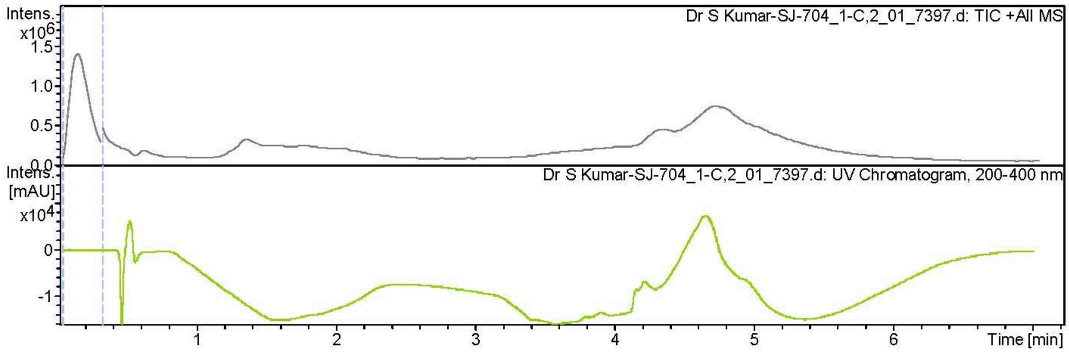

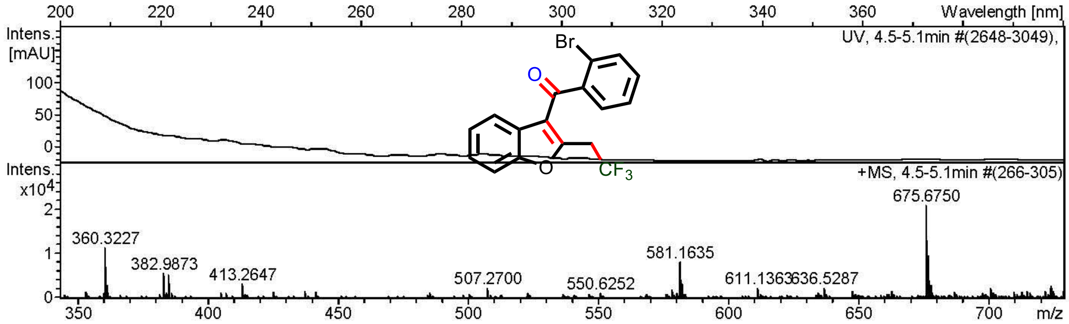

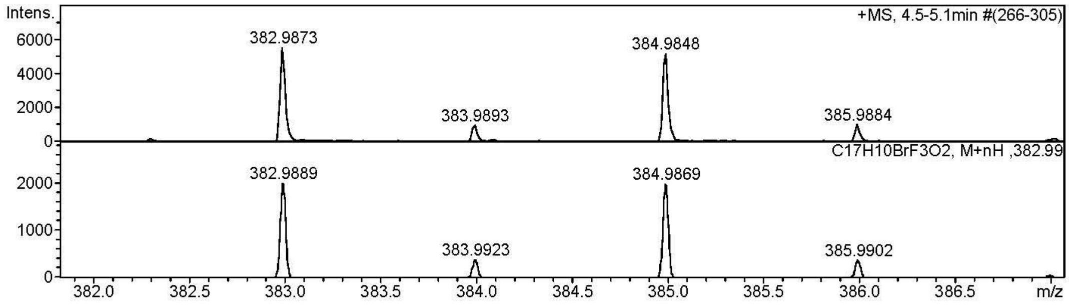

^{1}H NMR of (3,4-dimethoxyphenyl)(2-(2,2,2-trifluoroethyl)benzofuran-3-yl)methanone (**2i**)

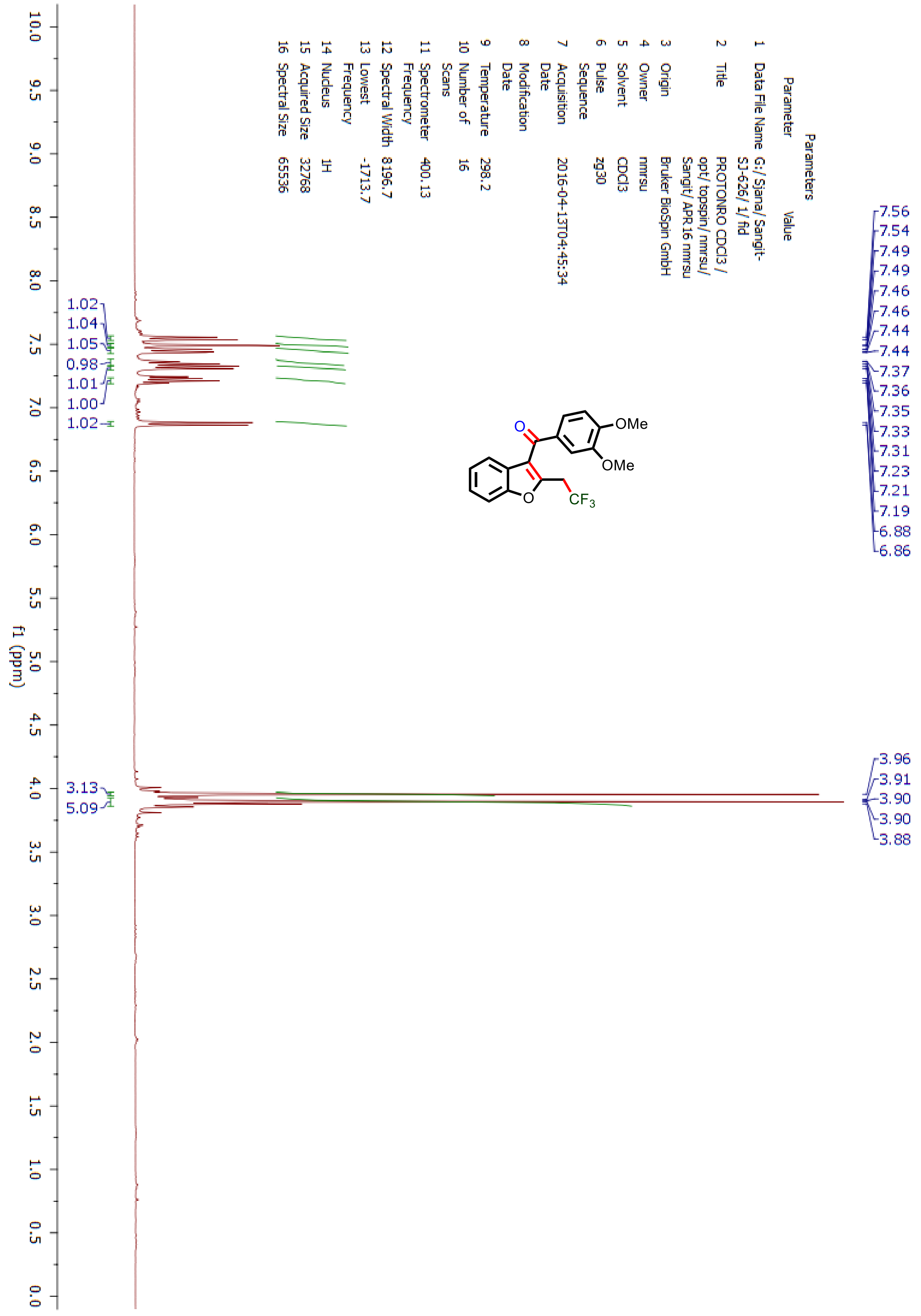

^{13}C NMR of (3,4-dimethoxyphenyl)(2-(2,2,2-trifluoroethyl)benzofuran-3-yl)methanone (**2i**)

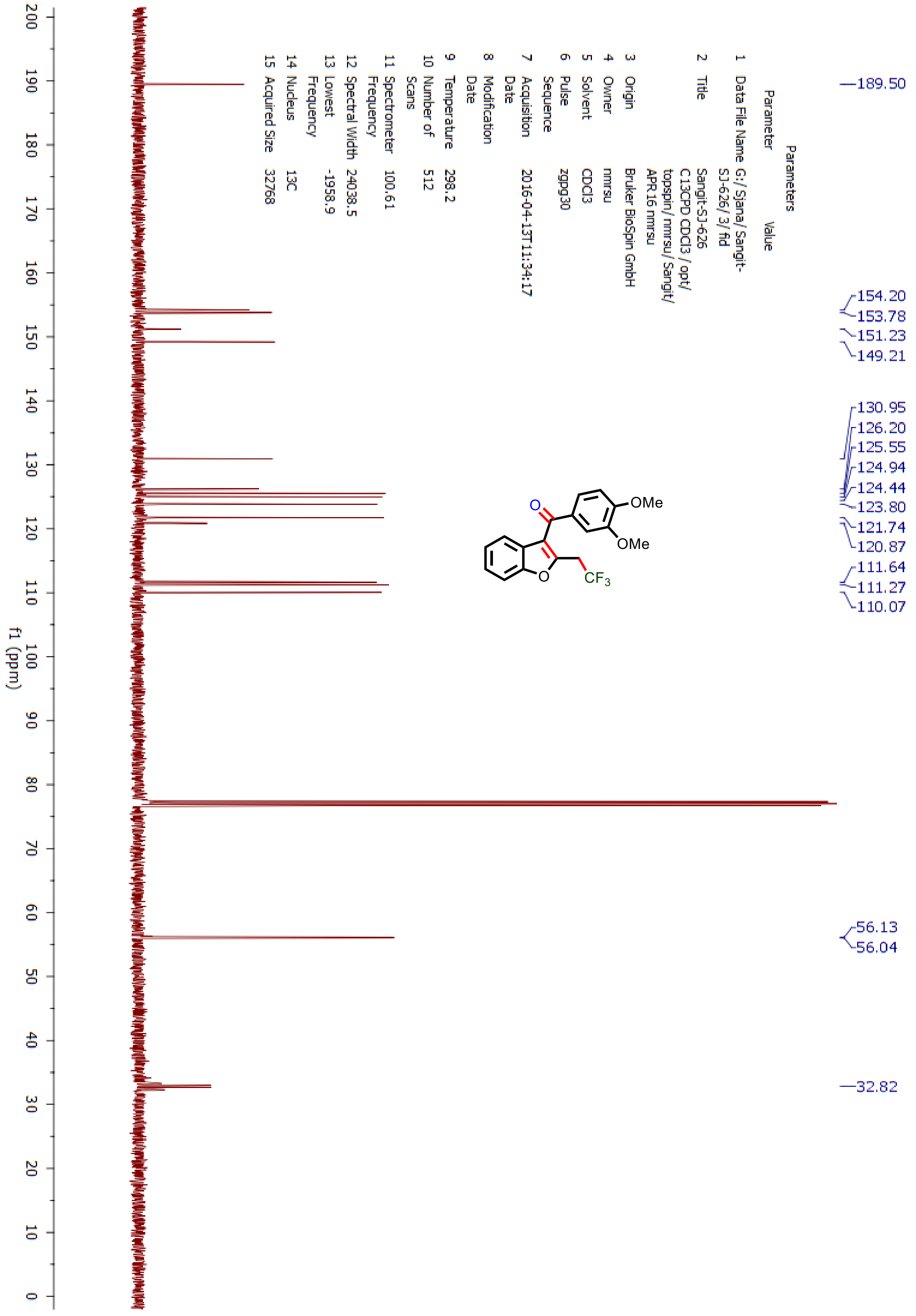

^{19}F NMR of (3,4-dimethoxyphenyl)(2-(2,2,2-trifluoroethyl)benzofuran-3-yl)methanone (**2i**)

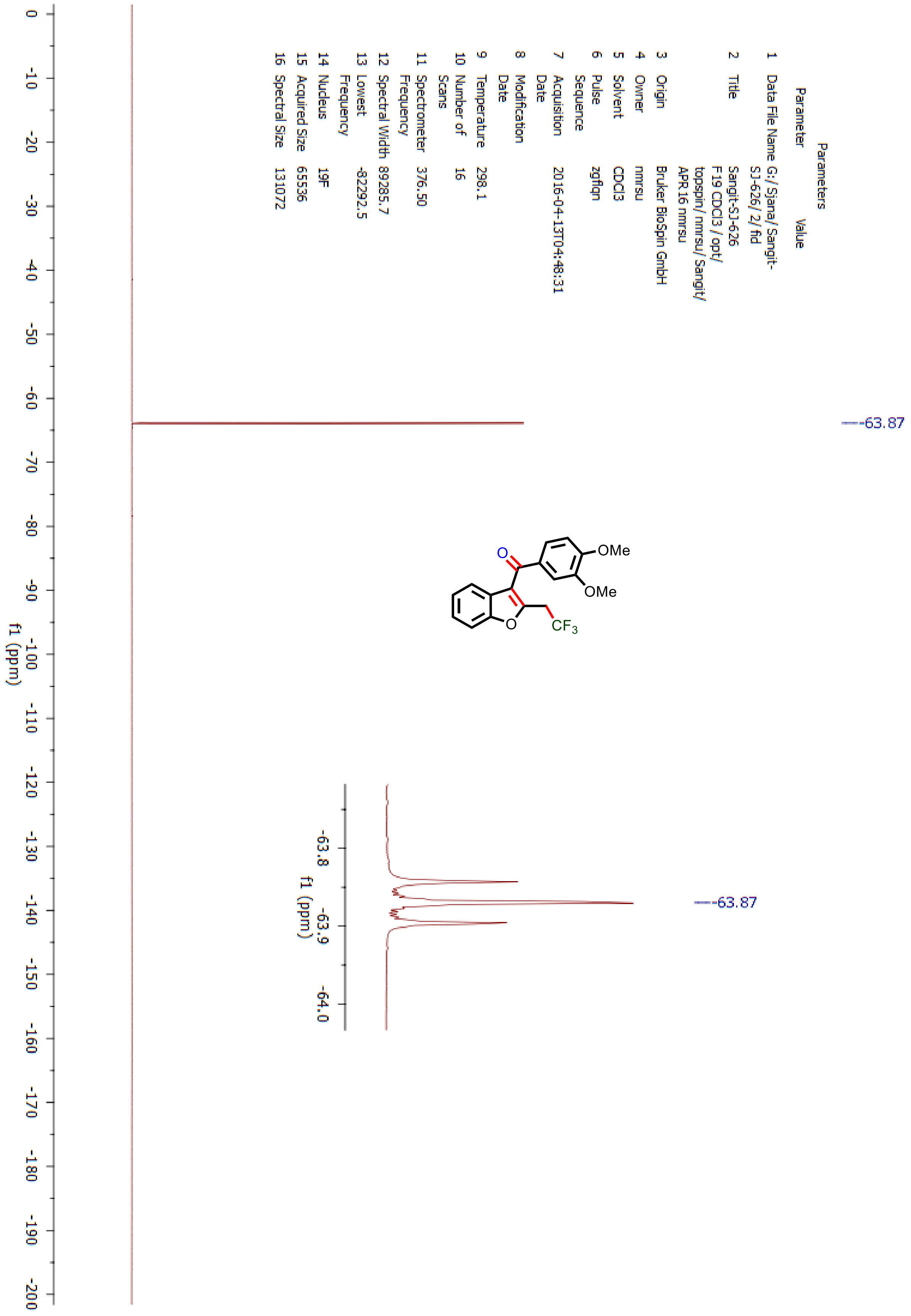

HRMS of (3,4-dimethoxyphenyl)(2-(2,2,2-trifluoroethyl)benzofuran-3-yl)methanone (**2i**)

Display Report

Analysis Info

Analysis Name	D:\Data\user data\2016\SEPT-2016\16-09-2016\Dr S Kumar-SJ-626_1-A,3_01_7409.d	Acquisition Date	9/16/2016 12:50:02 PM
Method	hrlcms_pos_mid_tunemix.m	Operator	DIMPLE
Sample Name	Dr S Kumar-SJ-626	Instrument	micrOTOF-Q II 10330
Comment			

Acquisition Parameter

Source Type	ESI	Ion Polarity	Positive	Set Nebulizer	0.3 Bar
Focus	Active	Set Capillary	4500 V	Set Dry Heater	200 °C
Scan Begin	50 m/z	Set End Plate Offset	-500 V	Set Dry Gas	4.0 l/min
Scan End	3000 m/z	Set Collision Cell RF	450.0 Vpp	Set Divert Valve	Waste

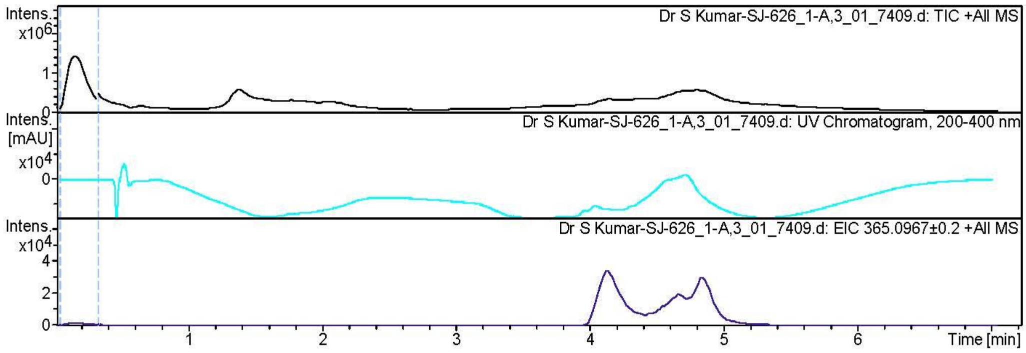

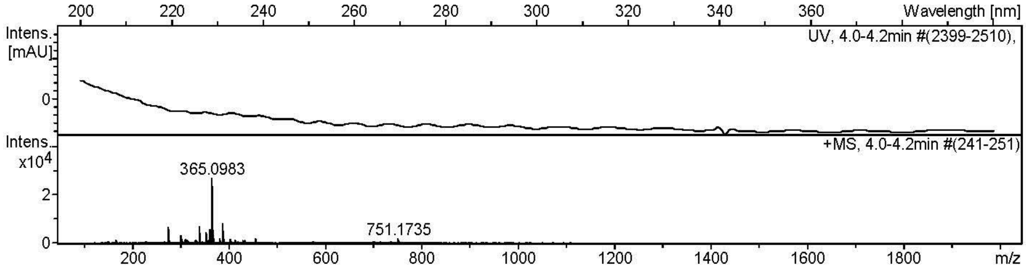

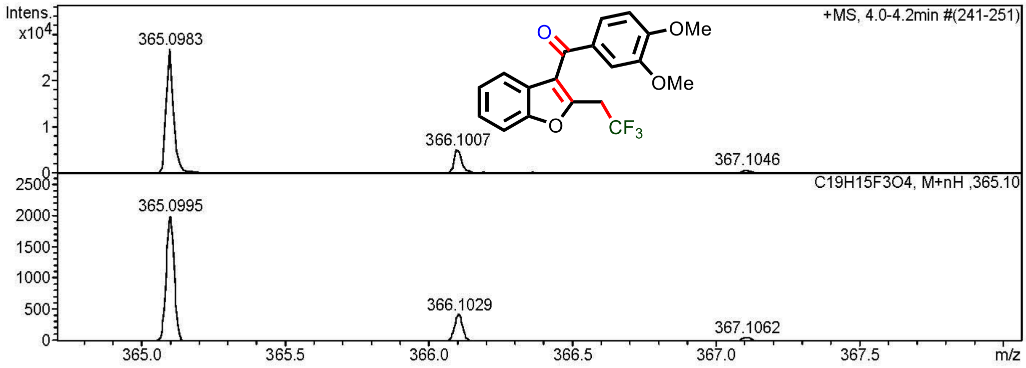

Bruker Compass DataAnalysis 4.0 printed: 9/16/2016 4:19:55 PM Page 1 of 1

^{1}H NMR of (3,5-difluorophenyl)(2-(2,2,2-trifluoroethyl)benzofuran-3-yl)methanone (**2j**)

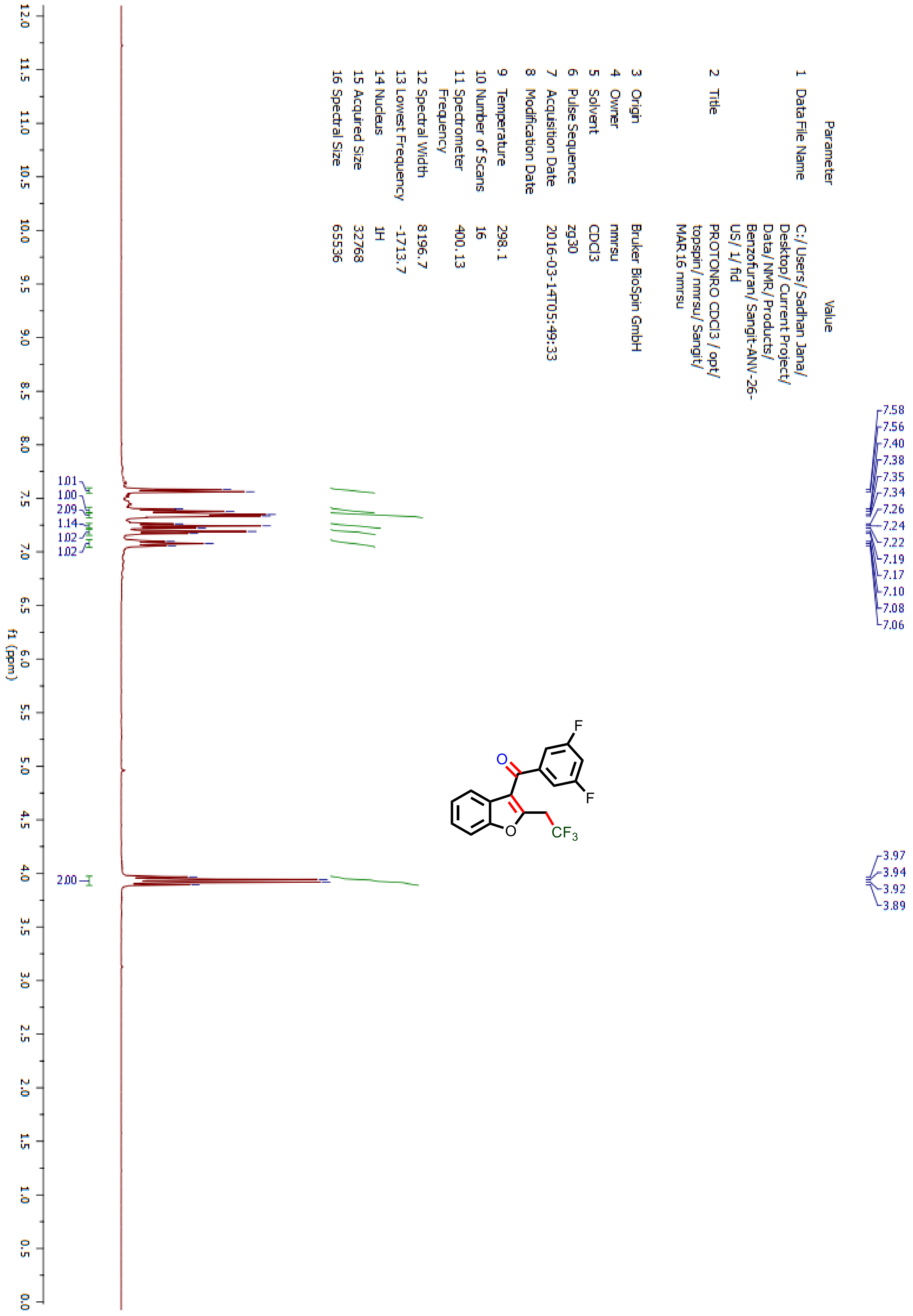

^{13}C NMR of (3,5-difluorophenyl)(2-(2,2,2-trifluoroethyl)benzofuran-3-yl)methanone (**2j**)

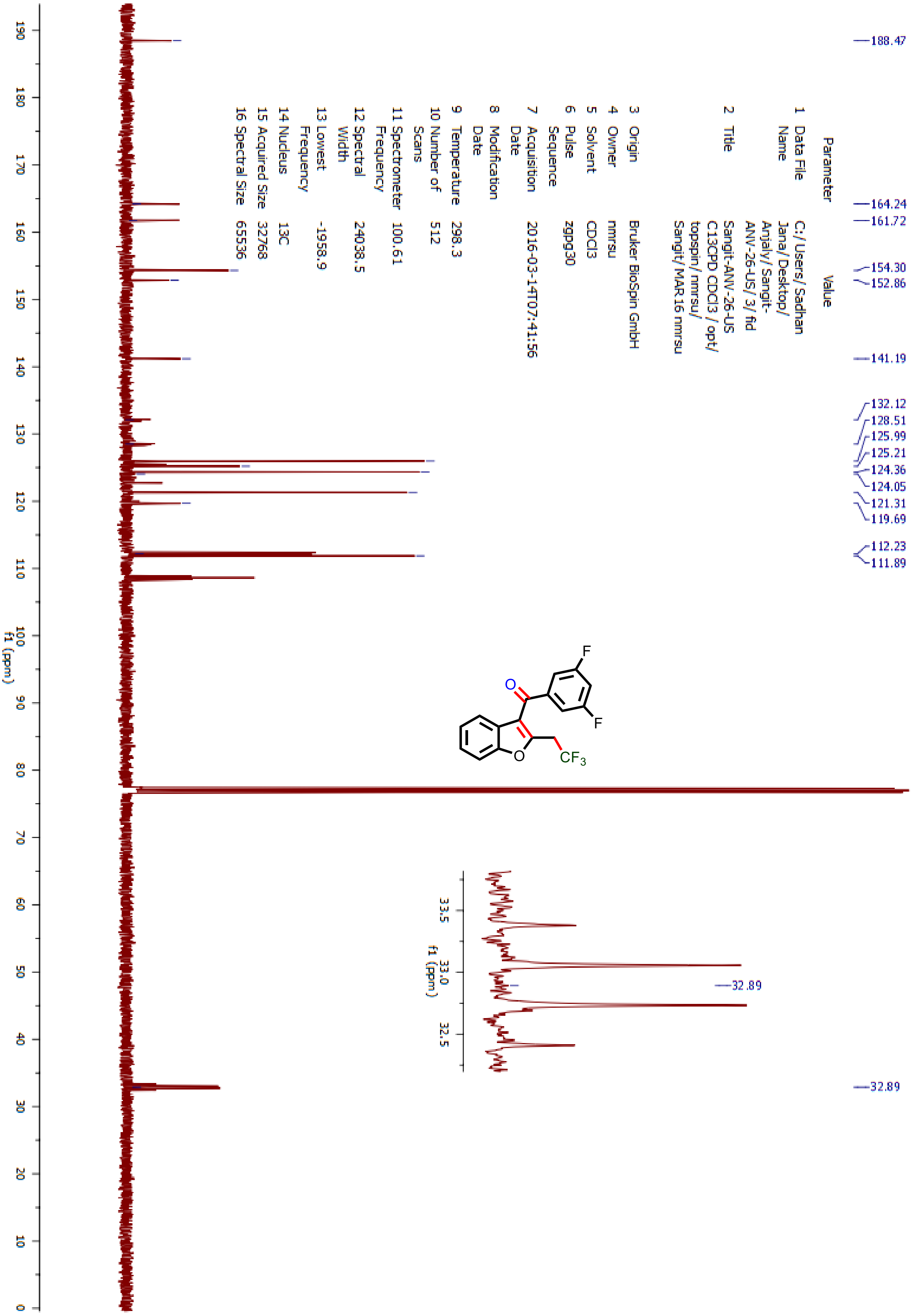

^{19}F NMR of (3,5-difluorophenyl)(2-(2,2,2-trifluoroethyl)benzofuran-3-yl)methanone (**2j**)

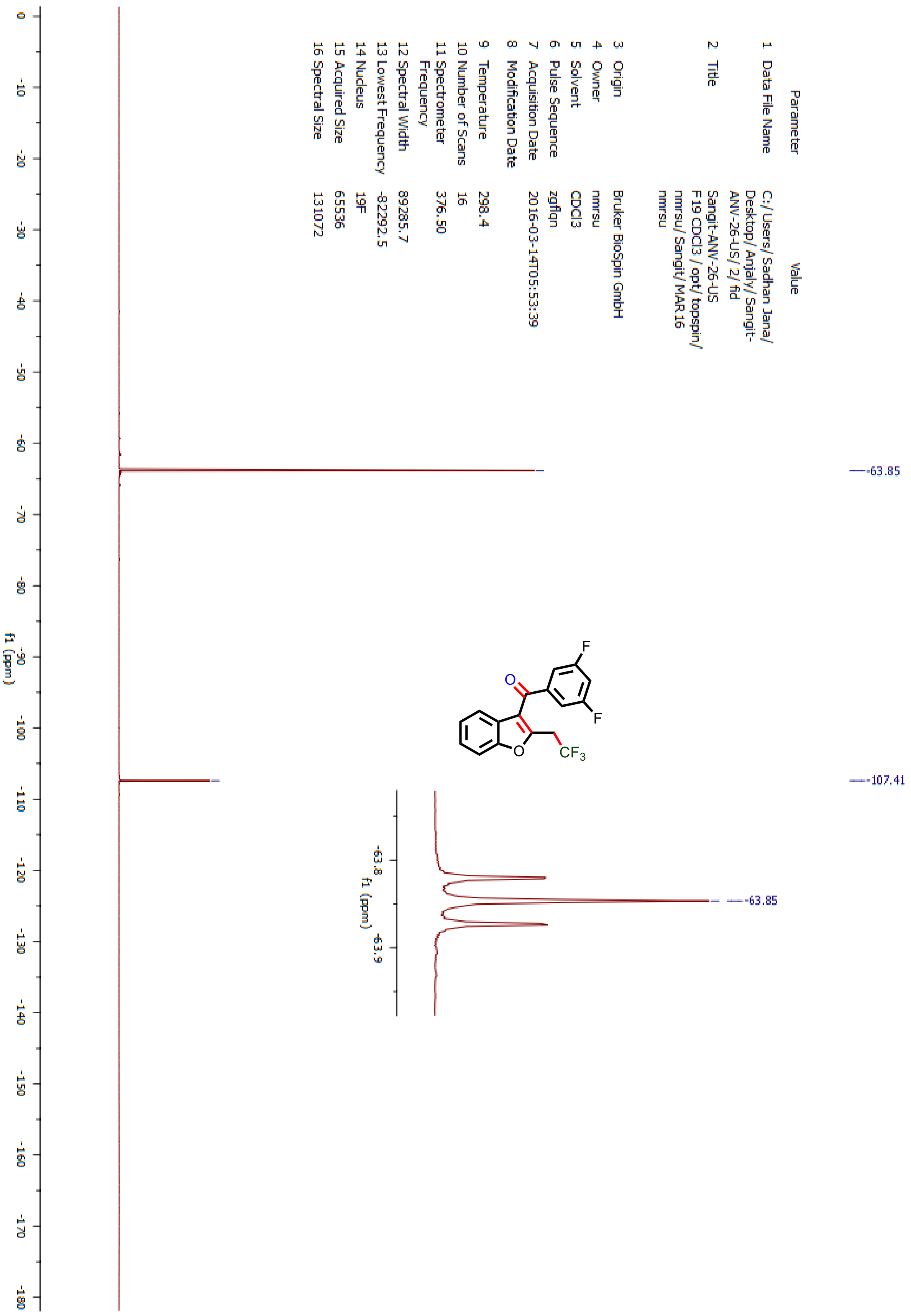

HRMS NMR of (3,5-difluorophenyl)(2-(2,2,2-trifluoroethyl)benzofuran-3-yl)methanone (**2j**)

Display Report

Analysis Info

Analysis Name	D:\Data\user data\2016\SEPT-2016\19-sep-2016\Dr S Kumar-ANV-26-US_1-D,6_01_7439.d	Acquisition Date	9/19/2016 1:16:36 PM
Method	hrlcms_pos_mid_tunemix.m	Operator	DIMPLE
Sample Name	Dr S Kumar-ANV-26-US	Instrument	micrOTOF-Q II 10330
Comment			

Acquisition Parameter

Source Type	ESI	Ion Polarity	Positive	Set Nebulizer	0.3 Bar
Focus	Active	Set Capillary	4500 V	Set Dry Heater	200 °C
Scan Begin	50 m/z	Set End Plate Offset	-500 V	Set Dry Gas	4.0 l/min
Scan End	3000 m/z	Set Collision Cell RF	450.0 Vpp	Set Divert Valve	Waste

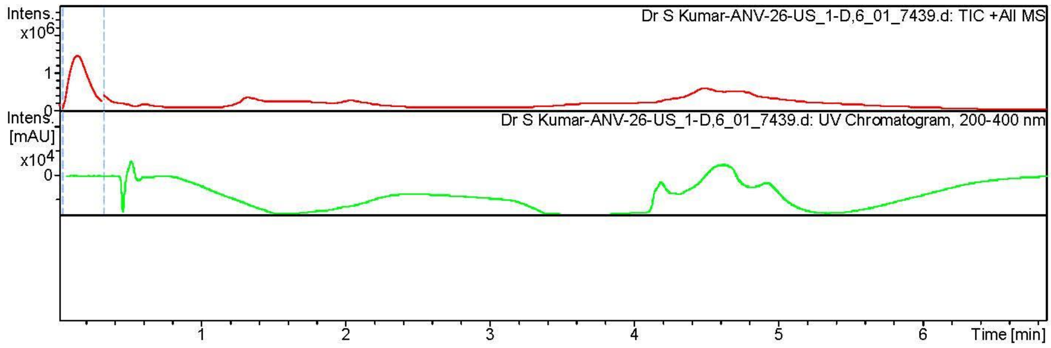

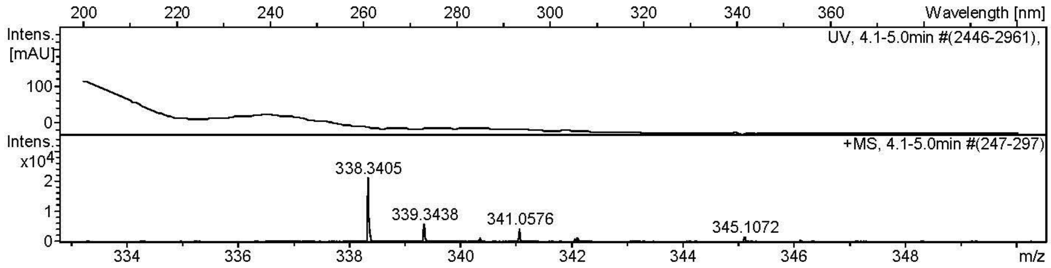

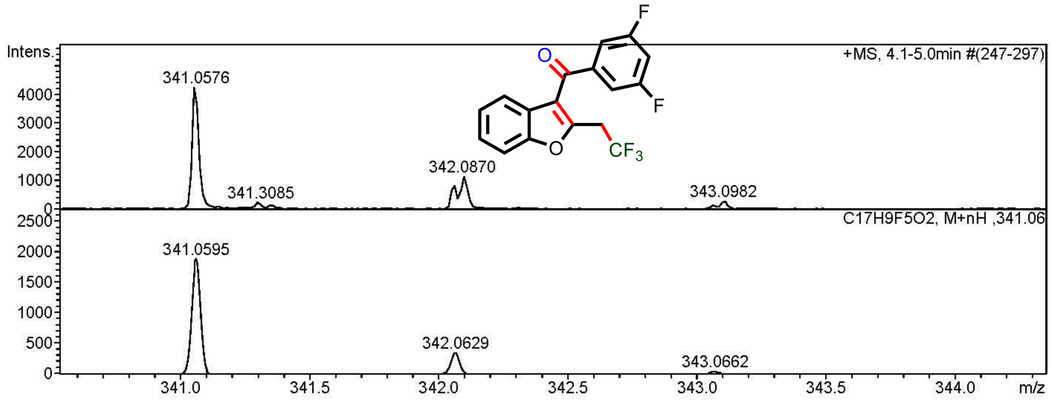

Bruker Compass DataAnalysis 4.0 printed: 9/19/2016 2:39:01 PM Page 1 of 1

^1H NMR of pyridin-2-yl(2-(2,2,2-trifluoroethyl)benzofuran-3-yl)methanone (**2k**)

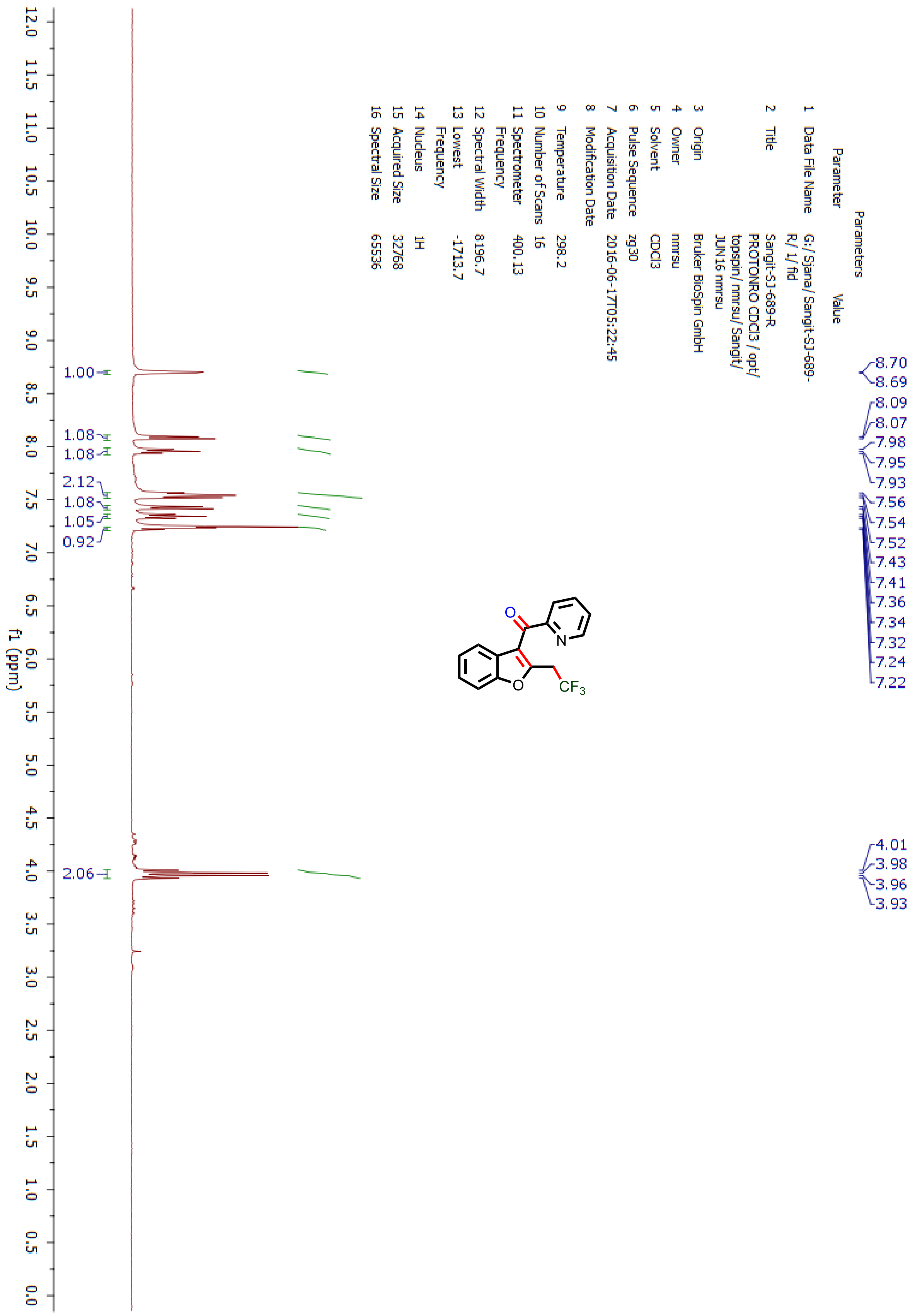

^{13}C NMR of pyridin-2-yl(2-(2,2,2-trifluoroethyl)benzofuran-3-yl)methanone (**2k**)

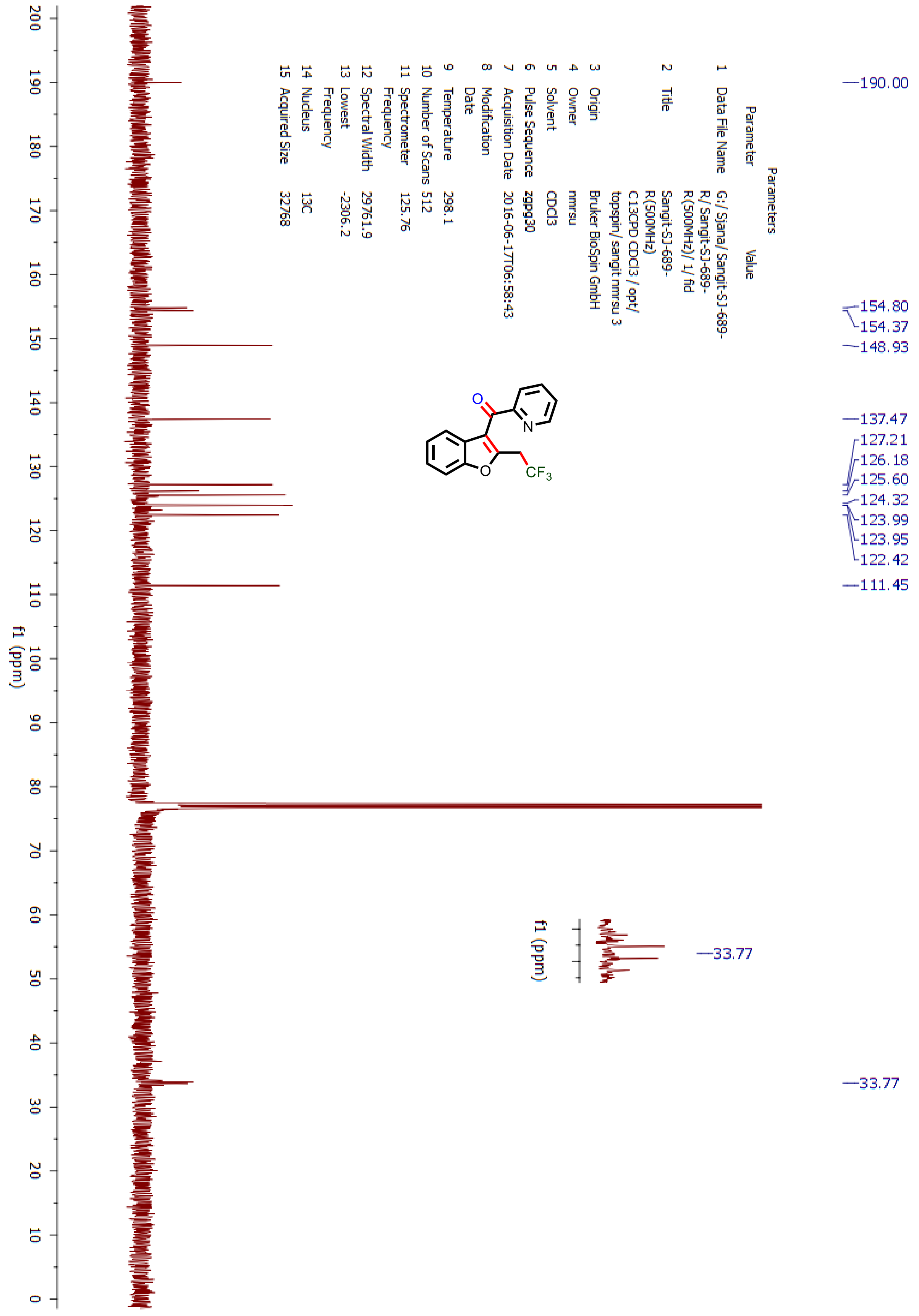

^{19}F NMR of pyridin-2-yl(2-(2,2,2-trifluoroethyl)benzofuran-3-yl)methanone (**2k**)

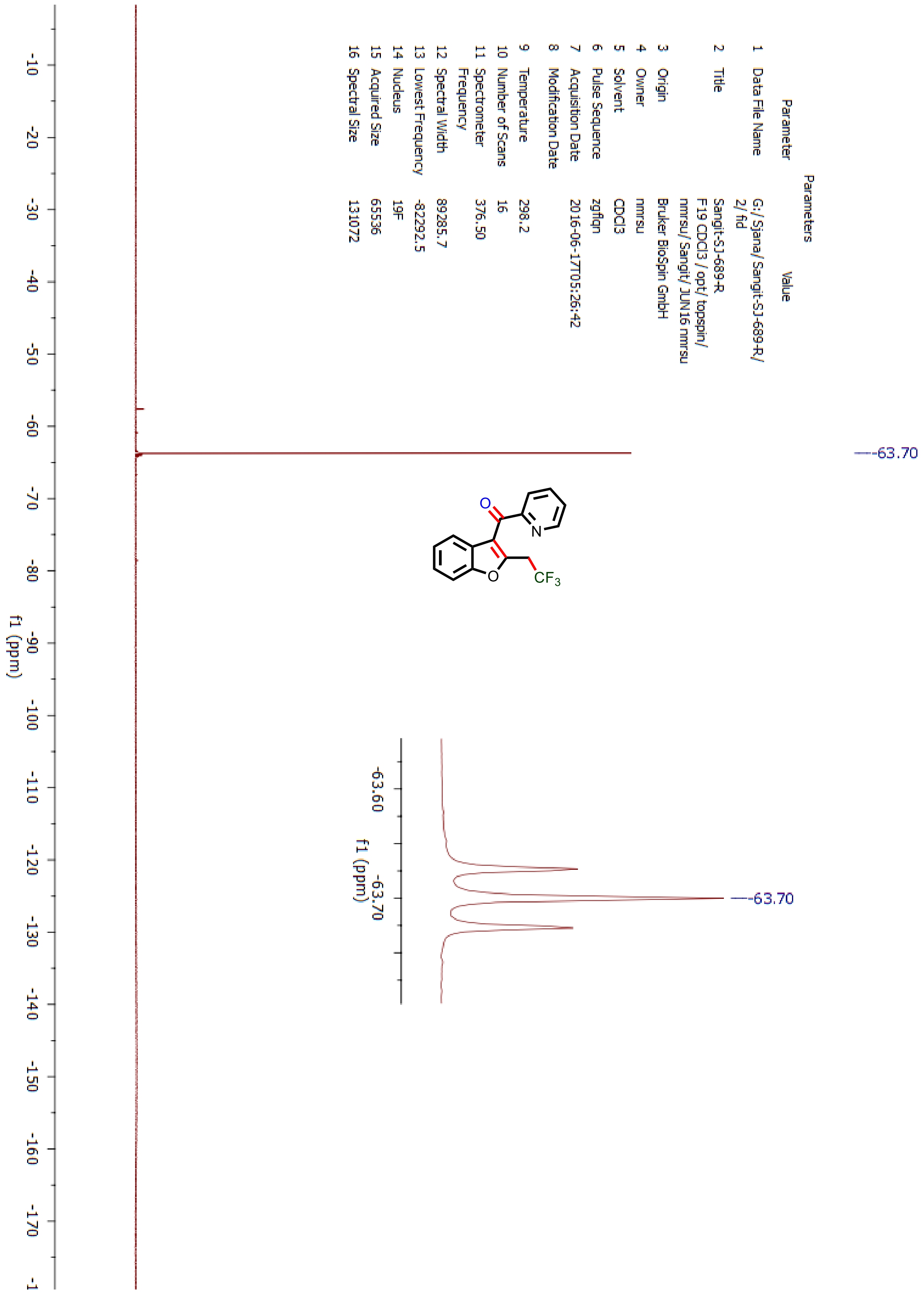

HRMS of pyridin-2-yl(2-(2,2,2-trifluoroethyl)benzofuran-3-yl)methanone (**2k**)

Display Report

Analysis Info

Analysis Name	D:\Data\user data\2016\SEPT-2016\15-09-2016\Dr S Kumar-SJ-689_1-C,1_01_7396.d	Acquisition Date	9/15/2016 3:21:04 PM
Method	hrlcms_pos_mid_tunemix.m	Operator	DIMPLE
Sample Name	Dr S Kumar-SJ-689	Instrument	micrOTOF-Q II 10330
Comment			

Acquisition Parameter

Source Type	ESI	Ion Polarity	Positive	Set Nebulizer	0.3 Bar
Focus	Active	Set Capillary	4500 V	Set Dry Heater	200 °C
Scan Begin	50 m/z	Set End Plate Offset	-500 V	Set Dry Gas	4.0 l/min
Scan End	3000 m/z	Set Collision Cell RF	450.0 Vpp	Set Divert Valve	Waste

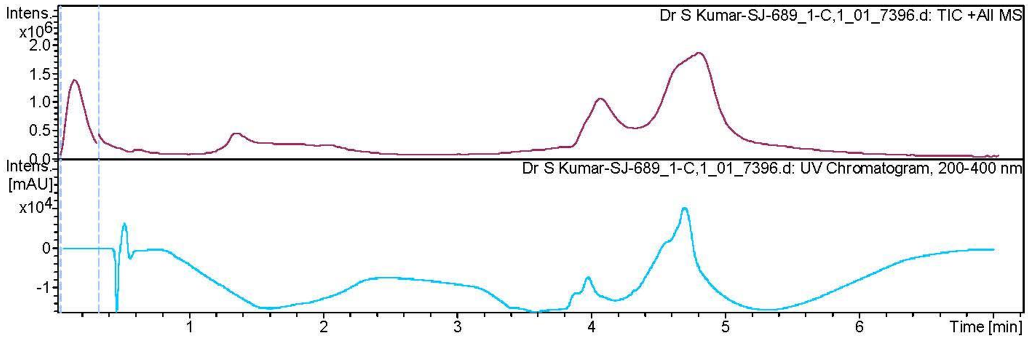

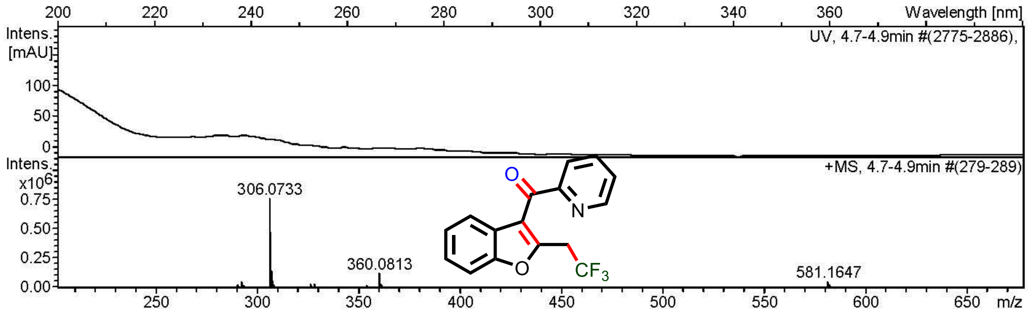

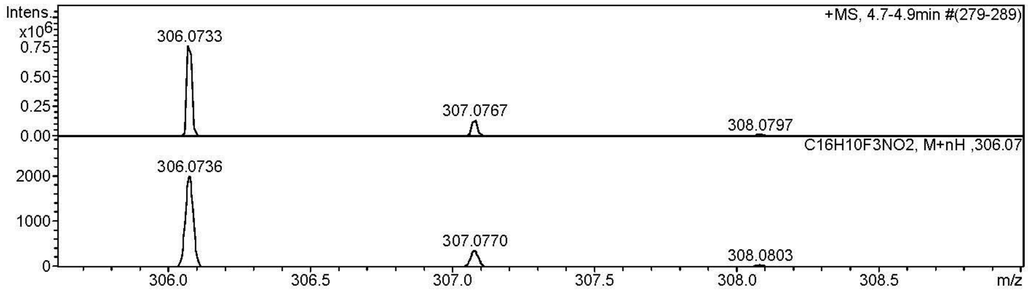

Bruker Compass DataAnalysis 4.0 printed: 9/15/2016 3:49:04 PM Page 1 of 1

^{1}H NMR of thiophen-3-yl(2-(2,2,2-trifluoroethyl)benzofuran-3-yl)methanone (**2l**)

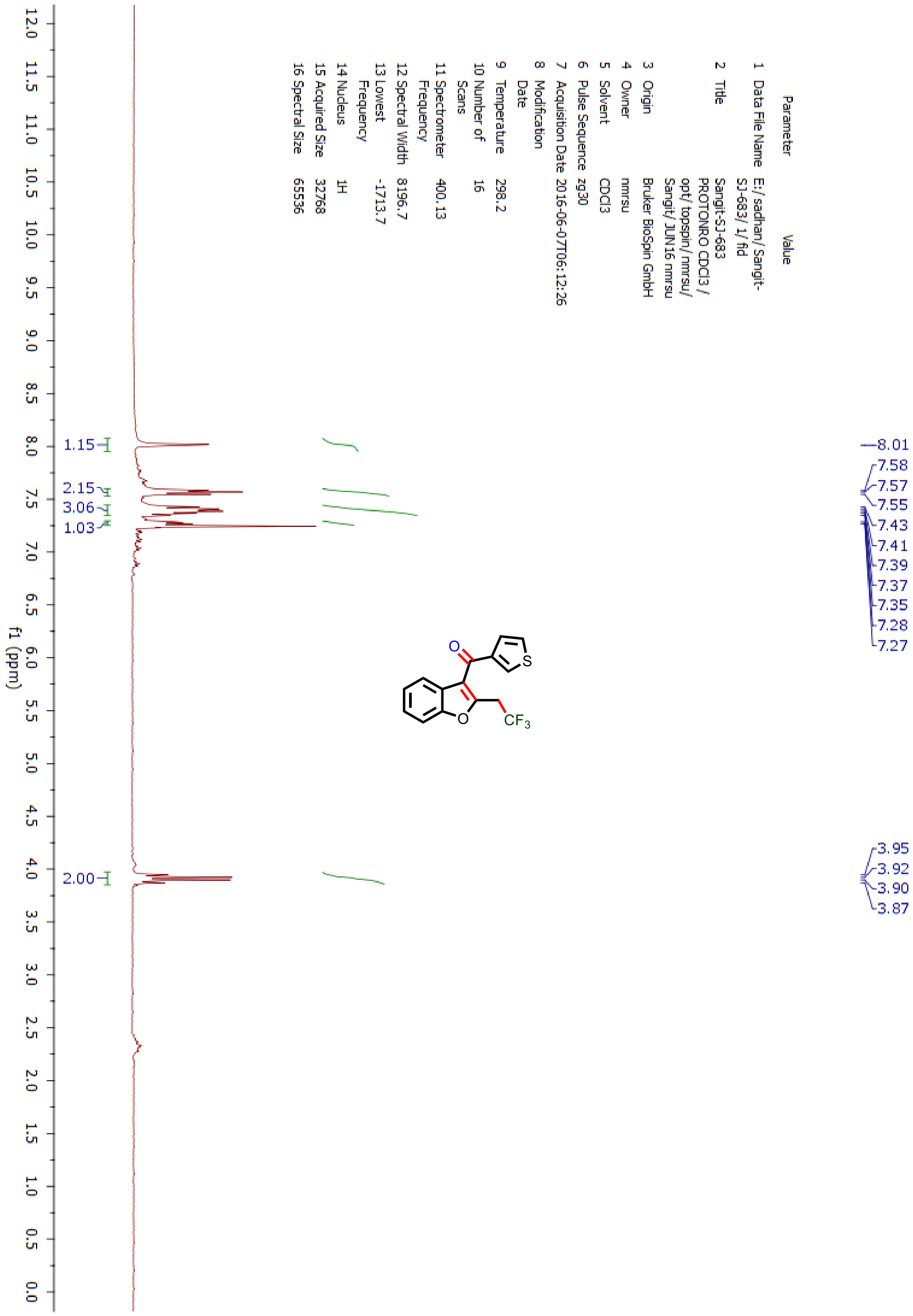

^{13}C NMR of thiophen-3-yl(2-(2,2,2-trifluoroethyl)benzofuran-3-yl)methanone (**2l**)

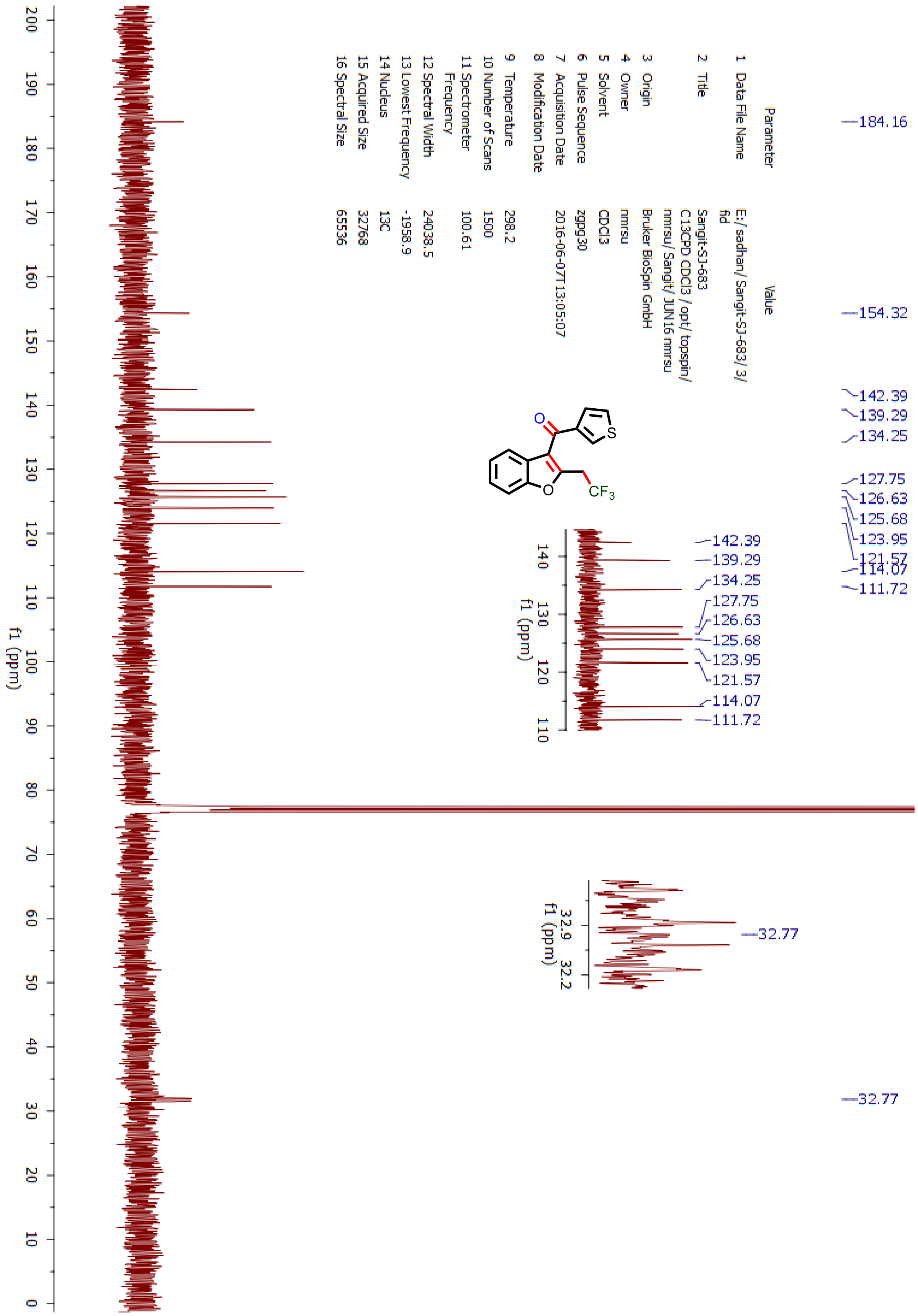

^{19}F NMR of thiophen-3-yl(2-(2,2,2-trifluoroethyl)benzofuran-3-yl)methanone (**2l**)

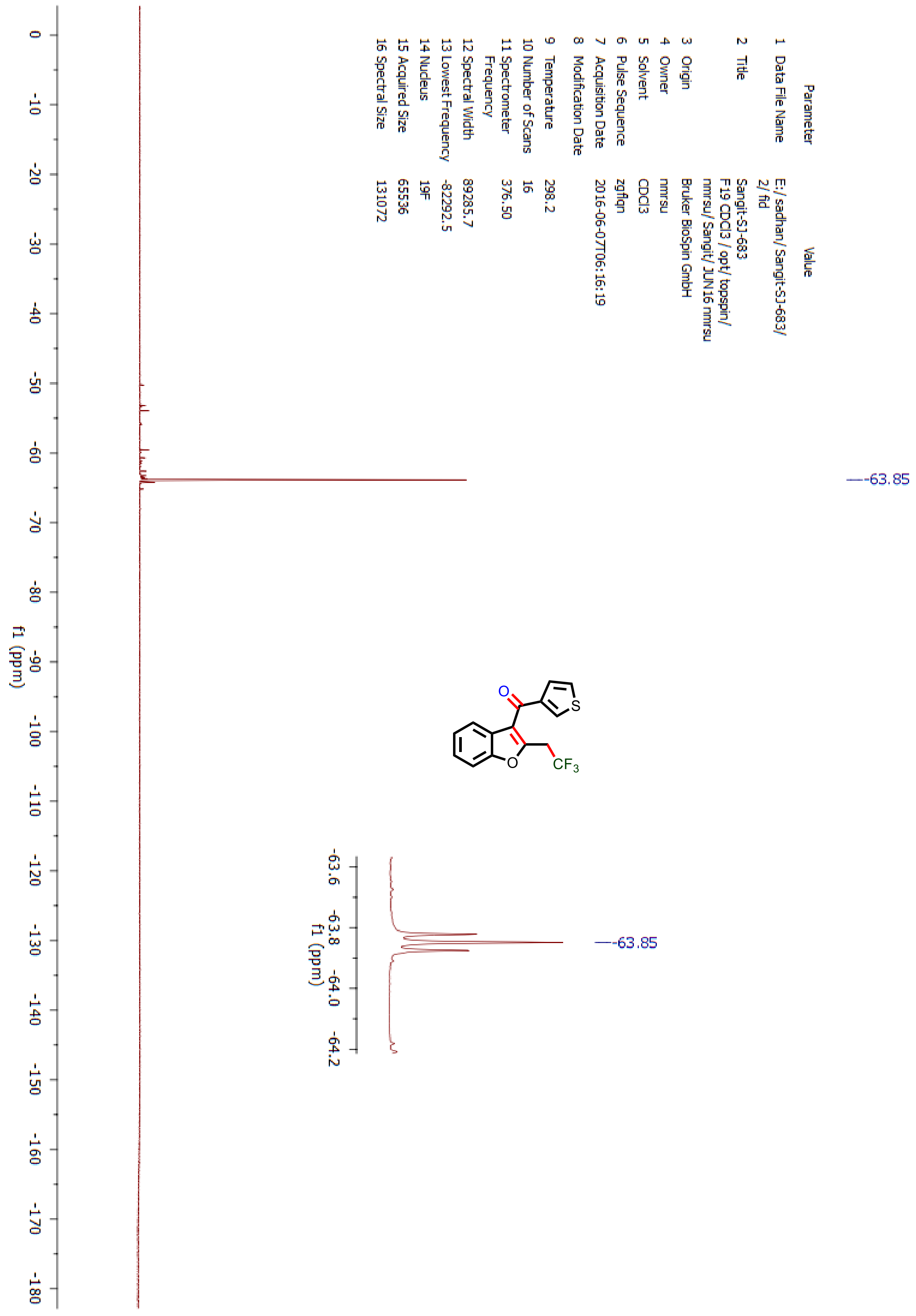

HRMS of thiophen-3-yl(2-(2,2,2-trifluoroethyl)benzofuran-3-yl)methanone (**2l**)

Display Report

Analysis Info

Analysis Name	D:\Data\user data\2016\SEPT-2016\15-09-2016\Dr S Kumar-SJ-683_1-B,7_01_7393.d	Acquisition Date	9/15/2016 2:56:29 PM
Method	hrlcms_pos_mid_tunemix.m	Operator	DIMPLE
Sample Name	Dr S Kumar-SJ-683	Instrument	micrOTOF-Q II 10330
Comment			

Acquisition Parameter

Source Type	ESI	Ion Polarity	Positive	Set Nebulizer	0.3 Bar
Focus	Active	Set Capillary	4500 V	Set Dry Heater	200 °C
Scan Begin	50 m/z	Set End Plate Offset	-500 V	Set Dry Gas	4.0 l/min
Scan End	3000 m/z	Set Collision Cell RF	450.0 Vpp	Set Divert Valve	Waste

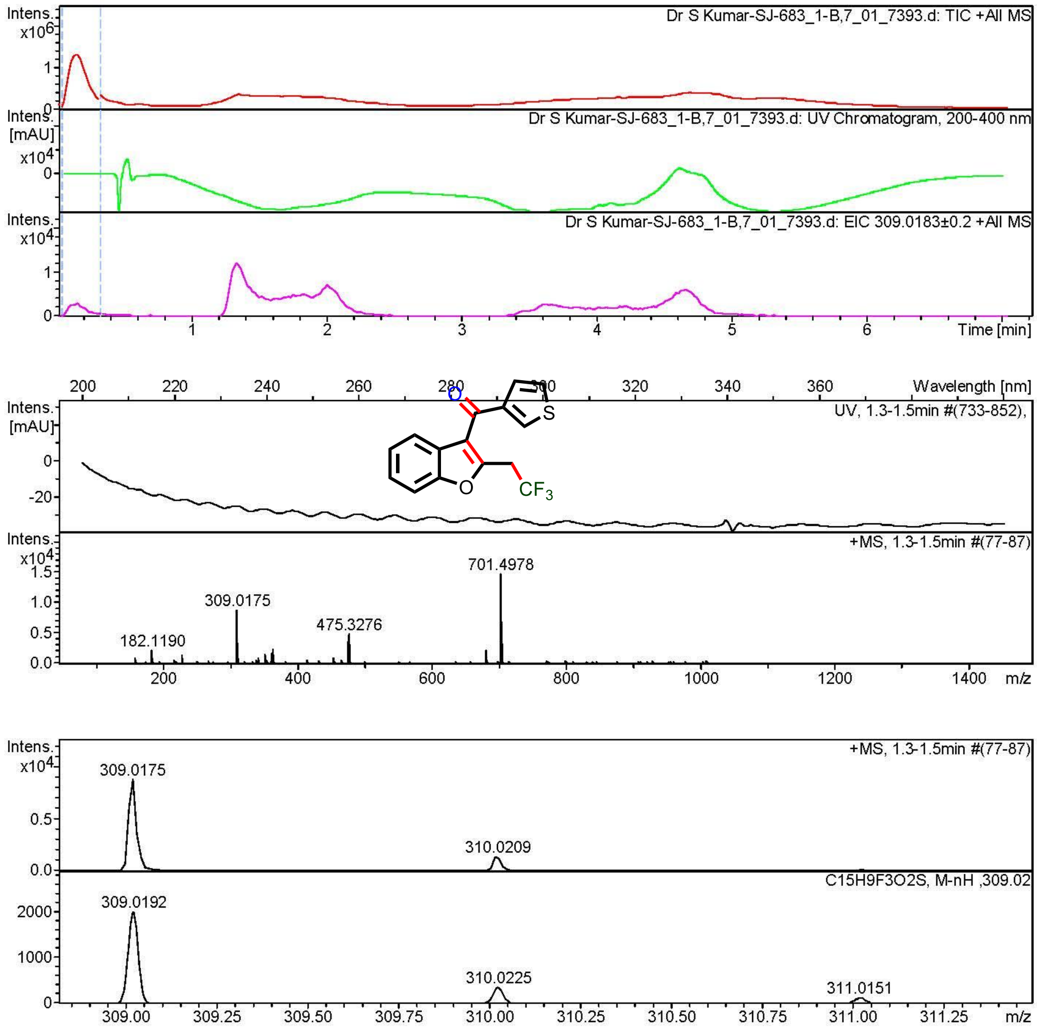

Bruker Compass DataAnalysis 4.0 printed: 9/15/2016 3:18:31 PM Page 1 of 1

^{1}H of naphthalen-2-yl(2-(2,2,2-trifluoroethyl)benzofuran-3-yl)methanone (**2m**)

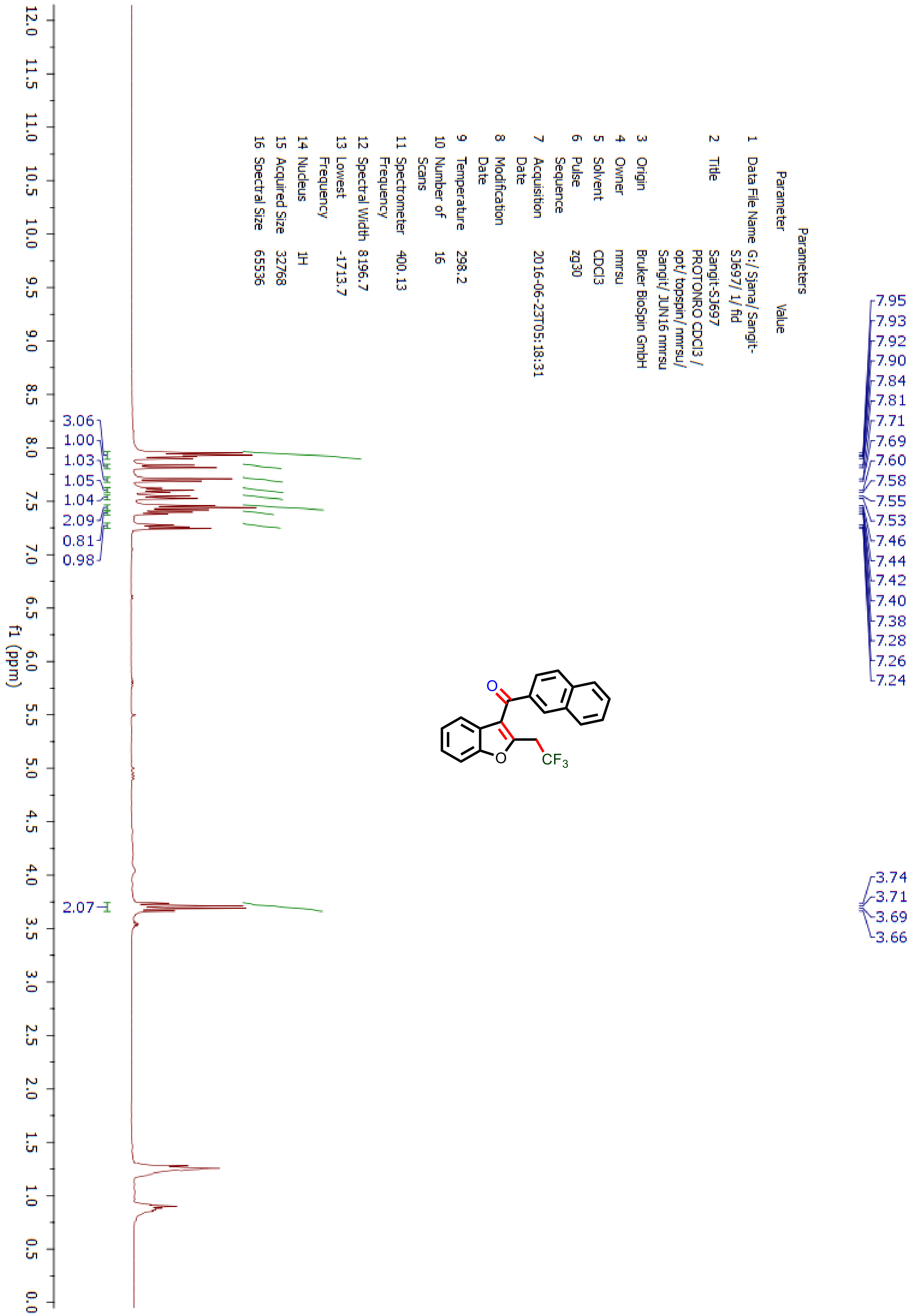

^{13}C of naphthalen-2-yl(2-(2,2,2-trifluoroethyl)benzofuran-3-yl)methanone (**2m**)

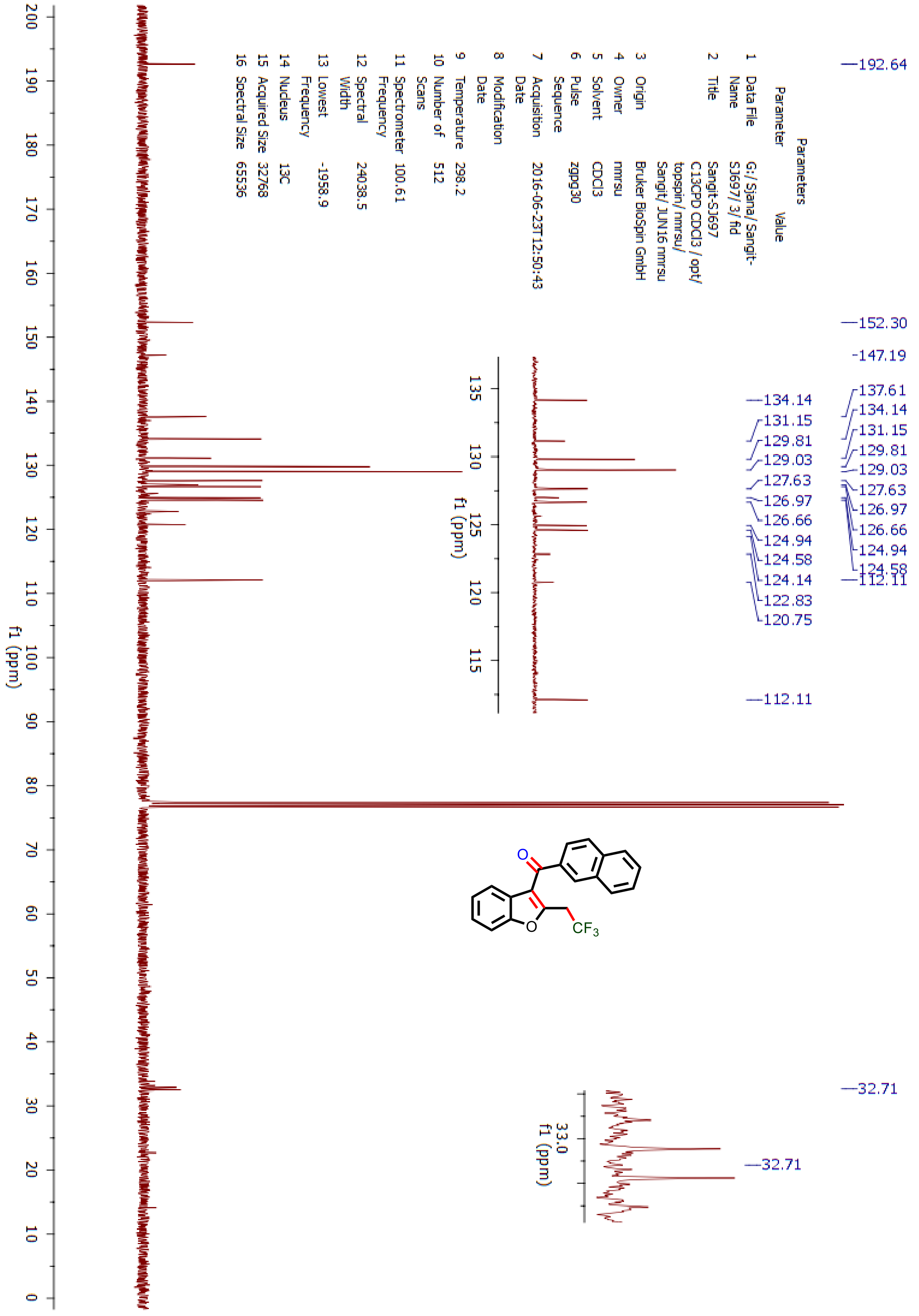

^{19}F of naphthalen-2-yl(2-(2,2,2-trifluoroethyl)benzofuran-3-yl)methanone (**2m**)

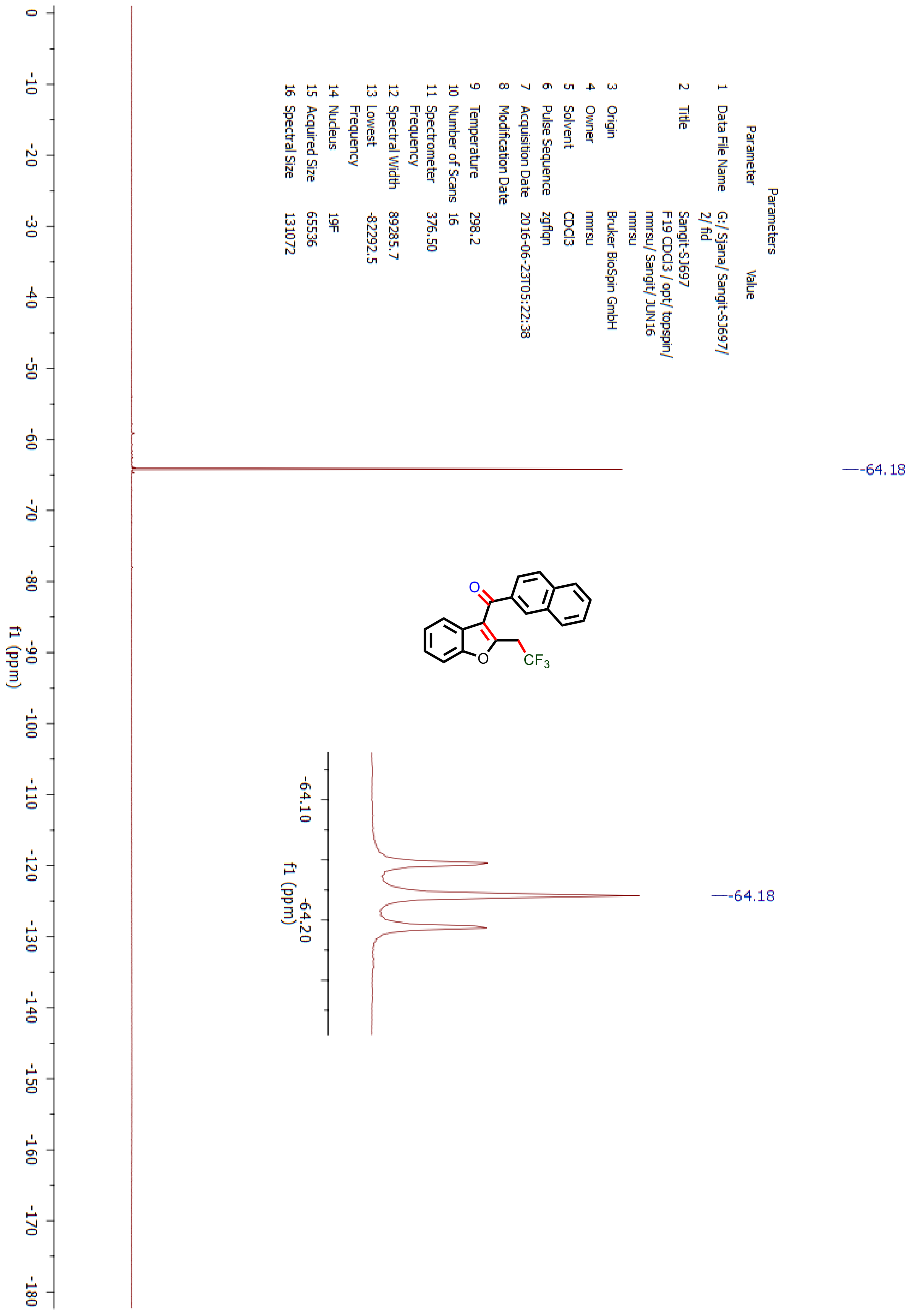

HRMS of naphthalen-2-yl(2-(2,2,2-trifluoroethyl)benzofuran-3-yl)methanone (**2m**)

Display Report

Analysis Info

		Acquisition Date	9/14/2016 2:22:40 PM
Analysis Name	D:\Data\user data\2016\SEPT-2016\14-09-2016\Dr S.Kumar-SJ-696_1-A,2_01_7373.d		
Method	hrlcms_pos_mid_tunemix.m	Operator	DIMPLE
Sample Name	Dr S.Kumar-SJ-696	Instrument	micrOTOF-Q II 10330
Comment			

Acquisition Parameter

Source Type	ESI	Ion Polarity	Positive	Set Nebulizer	0.3 Bar
Focus	Active	Set Capillary	4500 V	Set Dry Heater	200 °C
Scan Begin	50 m/z	Set End Plate Offset	-500 V	Set Dry Gas	4.0 l/min
Scan End	3000 m/z	Set Collision Cell RF	450.0 Vpp	Set Divert Valve	Waste

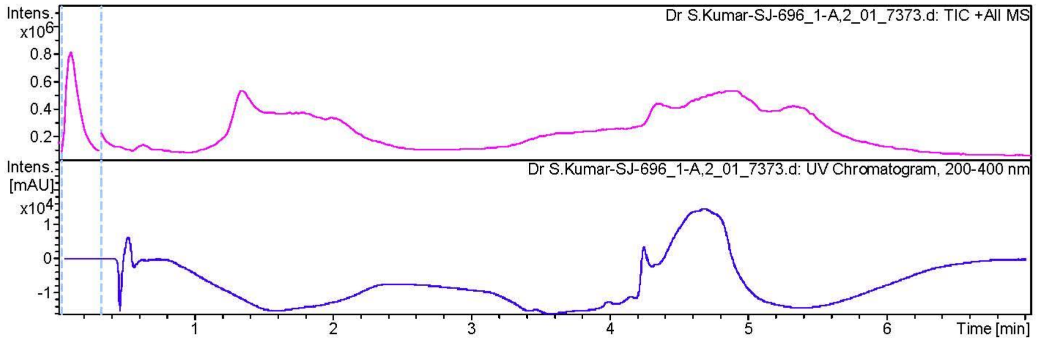

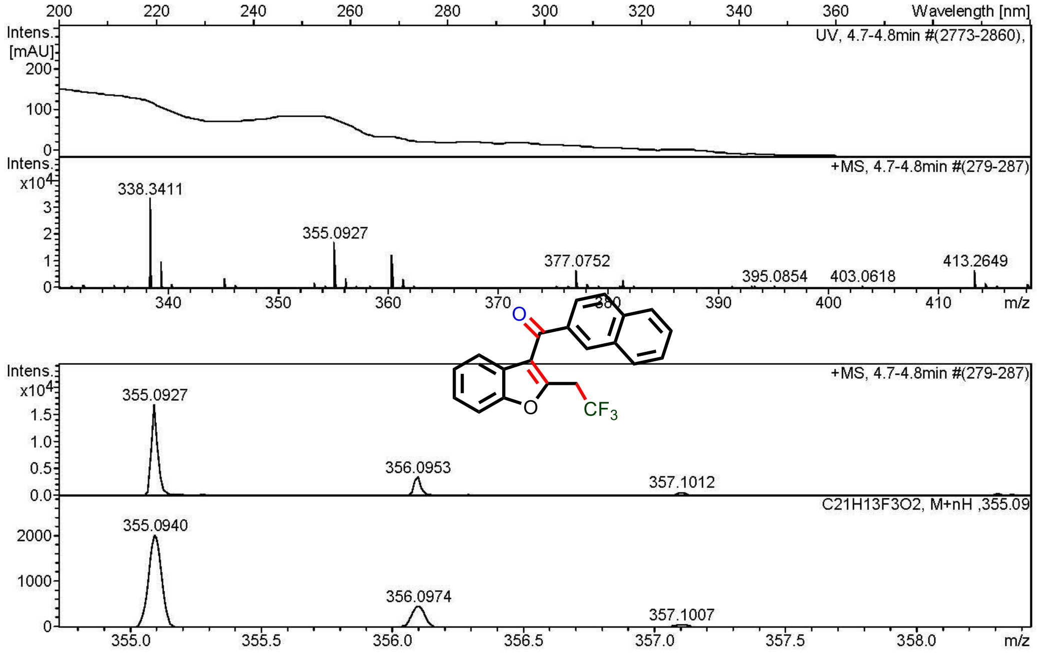

^1H of cyclopentyl(2-(2,2,2-trifluoroethyl)benzofuran-3-yl)methanone (**2n**)

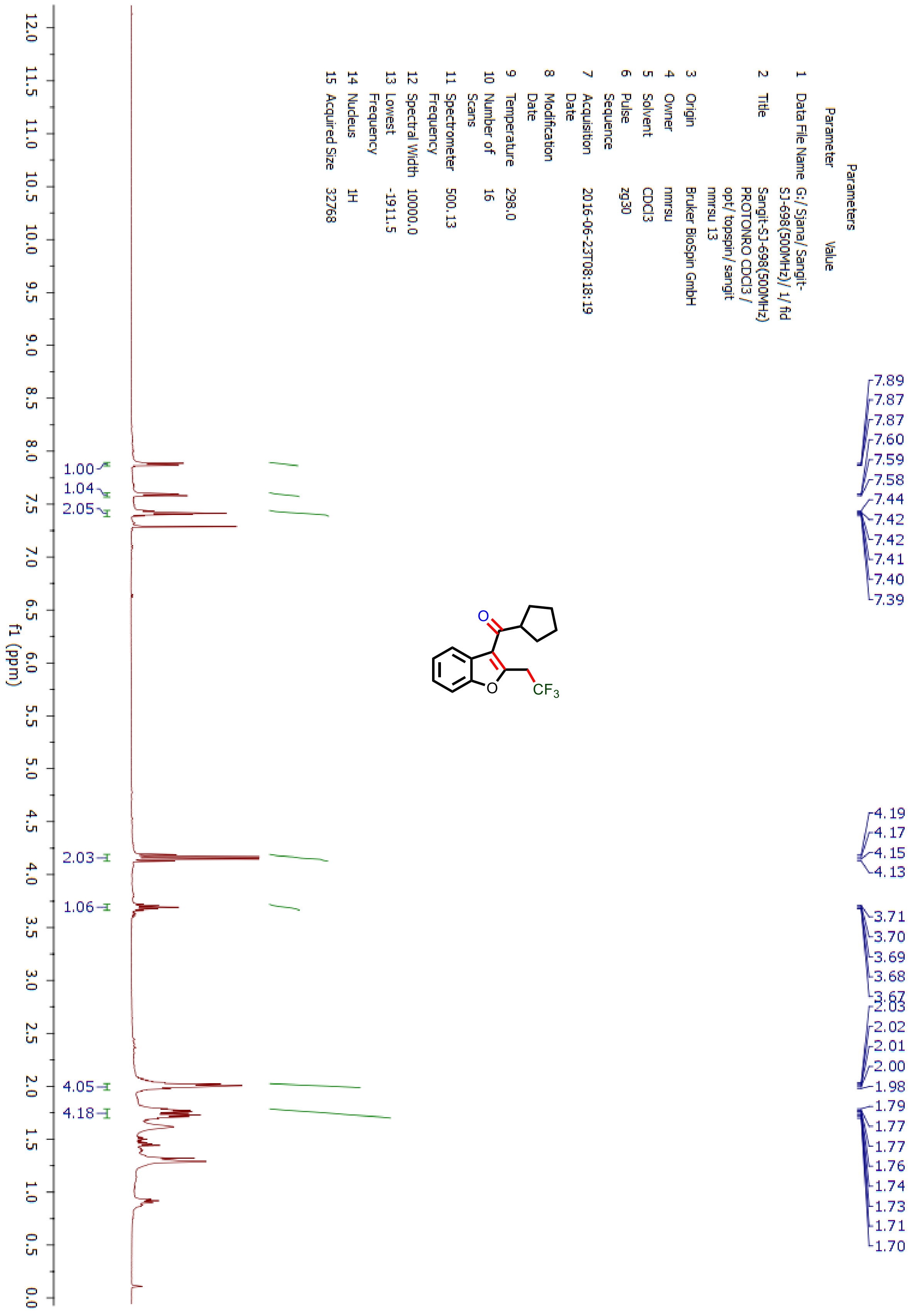

^{13}C of cyclopentyl(2-(2,2,2-trifluoroethyl)benzofuran-3-yl)methanone (**2n**)

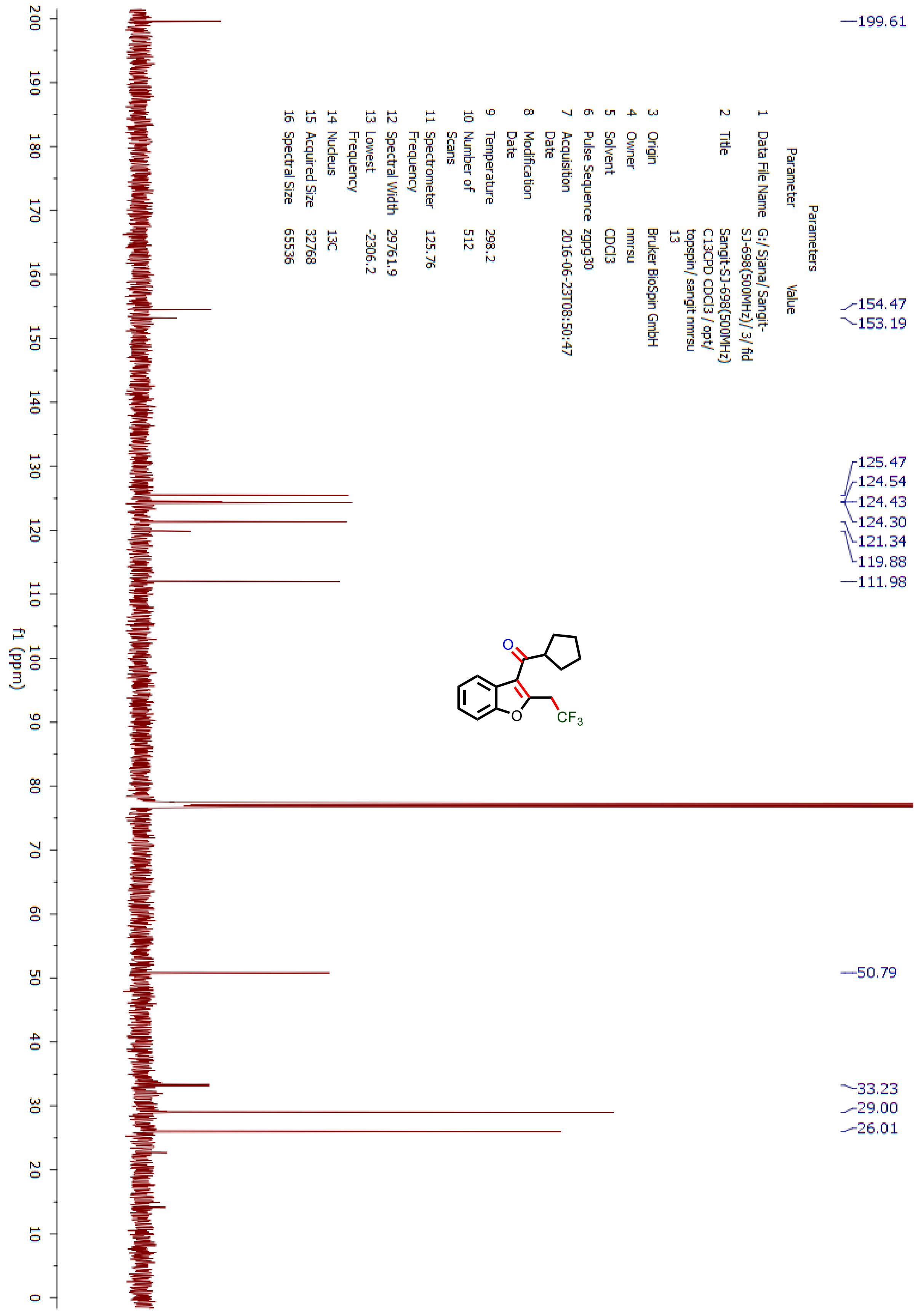

^{19}F of cyclopentyl(2-(2,2,2-trifluoroethyl)benzofuran-3-yl)methanone (**2n**)

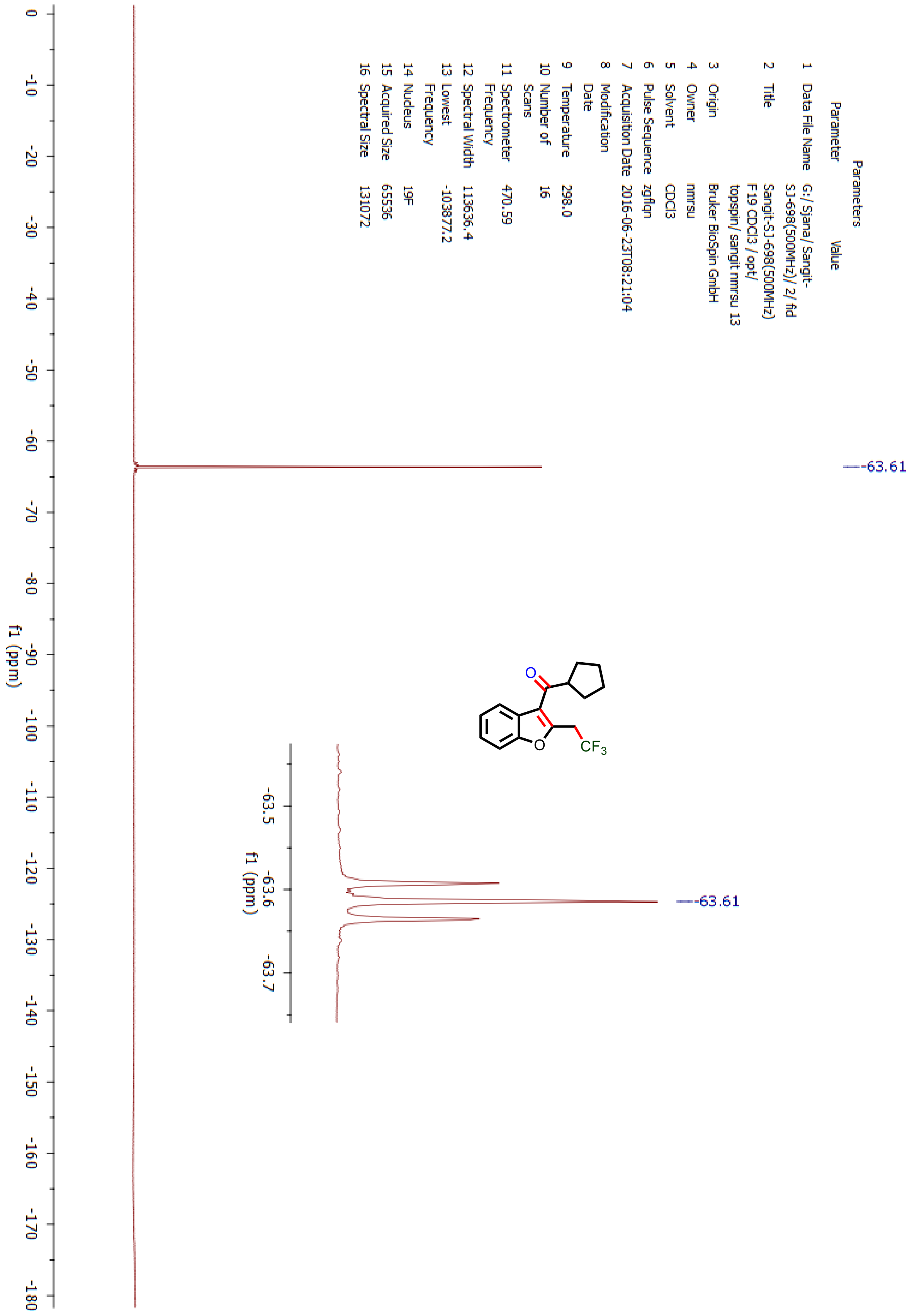

HRMS of cyclopentyl(2-(2,2,2-trifluoroethyl)benzofuran-3-yl)methanone (**2n**)

Display Report

Analysis Info

		Acquisition Date	9/14/2016 2:38:59 PM
Analysis Name	D:\Data\user data\2016\SEPT-2016\14-09-2016\Dr S.Kumar-SJ-698_1-A,4_01_7375.d		
Method	hrlcms_pos_mid_tunemix.m	Operator	DIMPLE
Sample Name	Dr S.Kumar-SJ-698	Instrument	micrOTOF-Q II 10330
Comment			

Acquisition Parameter

Source Type	ESI	Ion Polarity	Positive	Set Nebulizer	0.3 Bar
Focus	Active	Set Capillary	4500 V	Set Dry Heater	200 °C
Scan Begin	50 m/z	Set End Plate Offset	-500 V	Set Dry Gas	4.0 l/min
Scan End	3000 m/z	Set Collision Cell RF	450.0 Vpp	Set Divert Valve	Waste

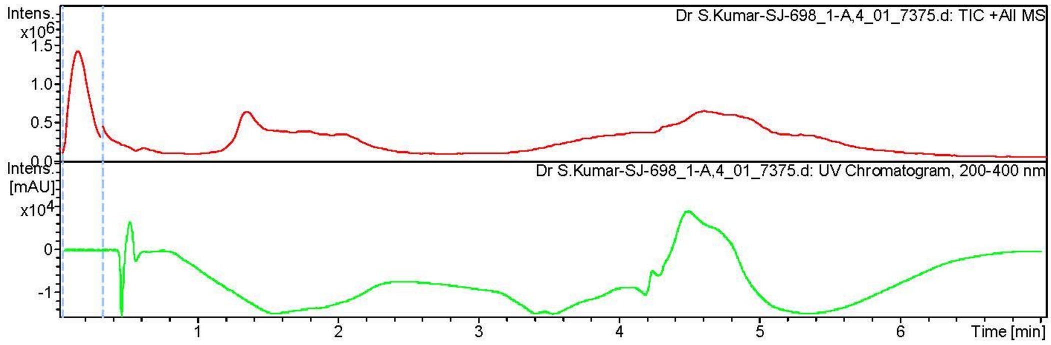

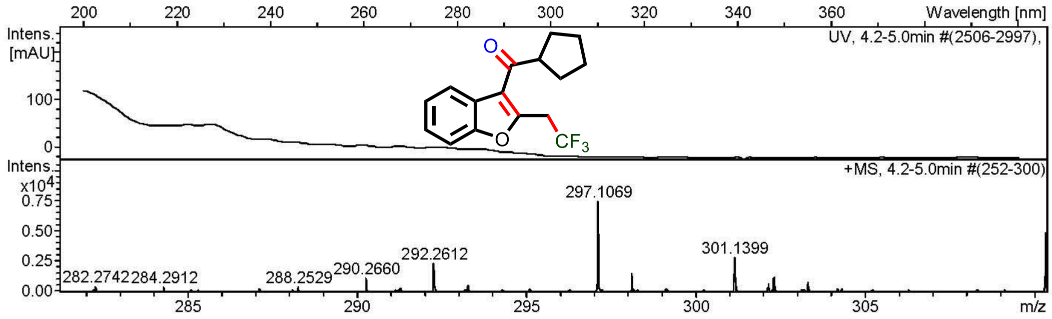

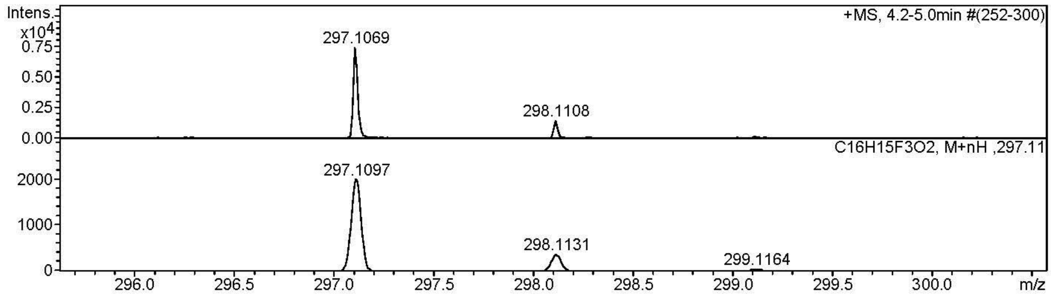

^{1}H NMR of 1-(2-(2,2,2-trifluoroethyl)benzofuran-3-yl)hexan-1-one (**2o**)

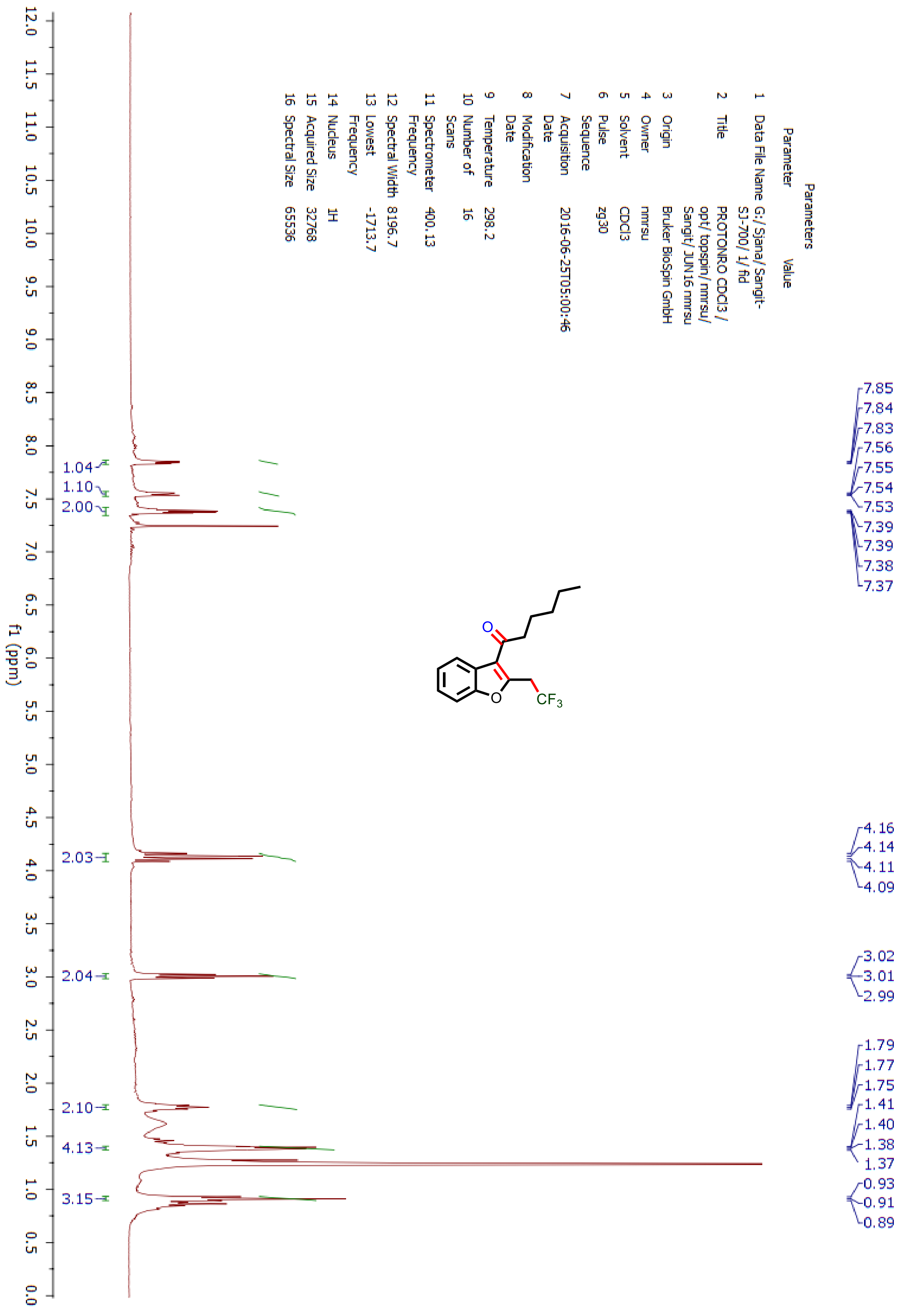

^{13}C NMR of 1-(2-(2,2,2-trifluoroethyl)benzofuran-3-yl)hexan-1-one (**2o**)

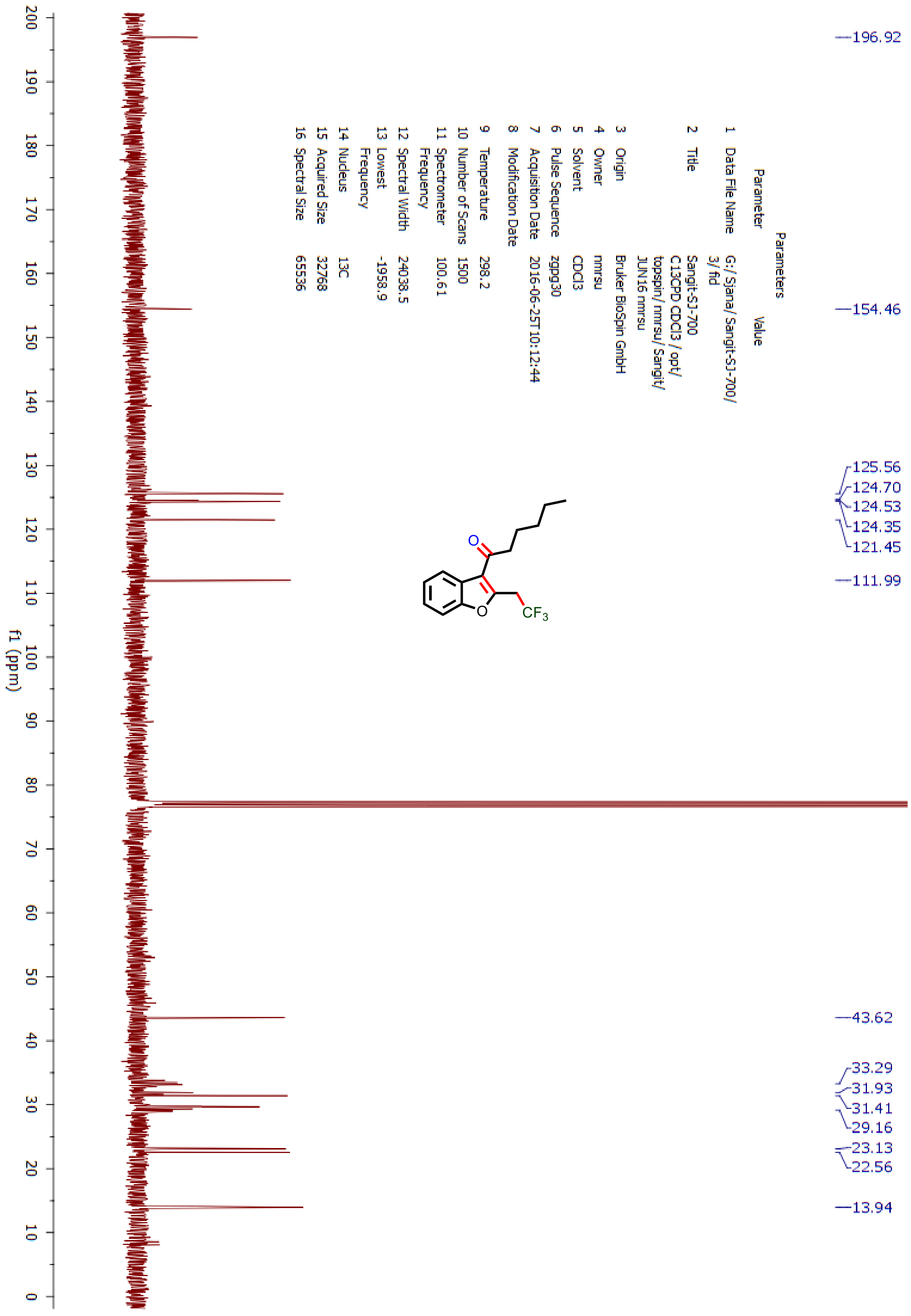

^{19}F NMR of 1-(2-(2,2,2-trifluoroethyl)benzofuran-3-yl)hexan-1-one (**2o**)

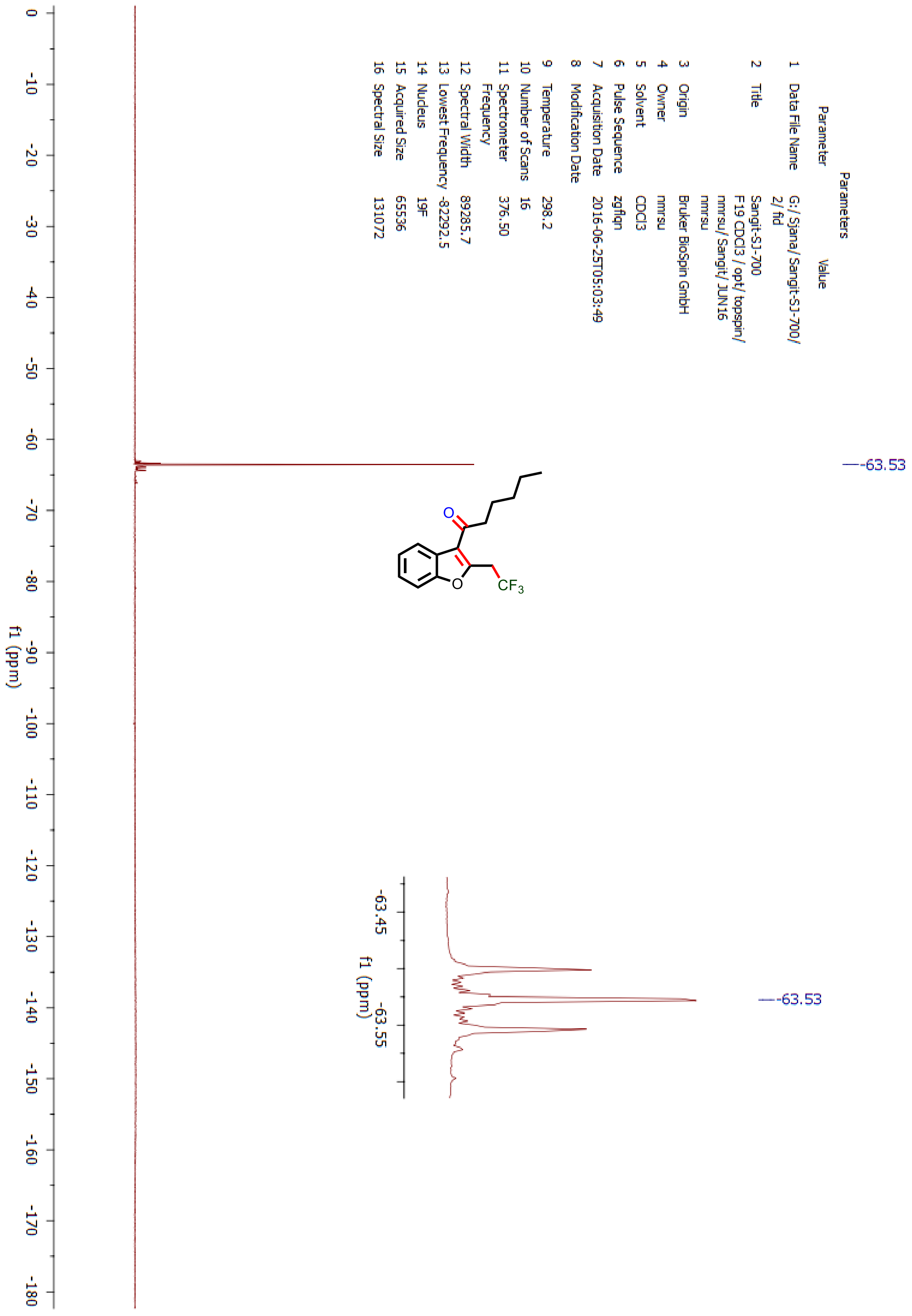

HRMS of 1-(2-(2,2,2-trifluoroethyl)benzofuran-3-yl)hexan-1-one (**2o**)

Display Report

Analysis Info

Analysis Name	D:\Data\user data\2016\SEPT-2016\14-09-2016\Dr S.Kumar-SJ-700_1-A,5_01_7376.d	Acquisition Date	9/14/2016 2:47:08 PM
Method	hrlcms_pos_mid_tunemix.m	Operator	DIMPLE
Sample Name	Dr S.Kumar-SJ-700	Instrument	micrOTOF-Q II 10330
Comment			

Acquisition Parameter

Source Type	ESI	Ion Polarity	Positive	Set Nebulizer	0.3 Bar
Focus	Active	Set Capillary	4500 V	Set Dry Heater	200 °C
Scan Begin	50 m/z	Set End Plate Offset	-500 V	Set Dry Gas	4.0 l/min
Scan End	3000 m/z	Set Collision Cell RF	450.0 Vpp	Set Divert Valve	Waste

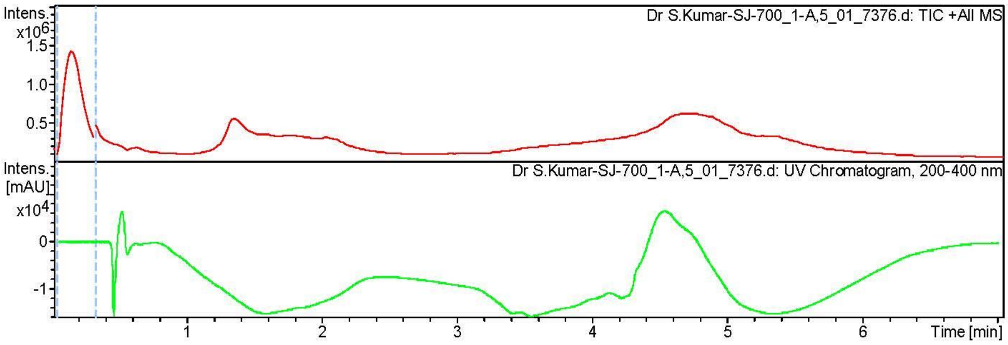

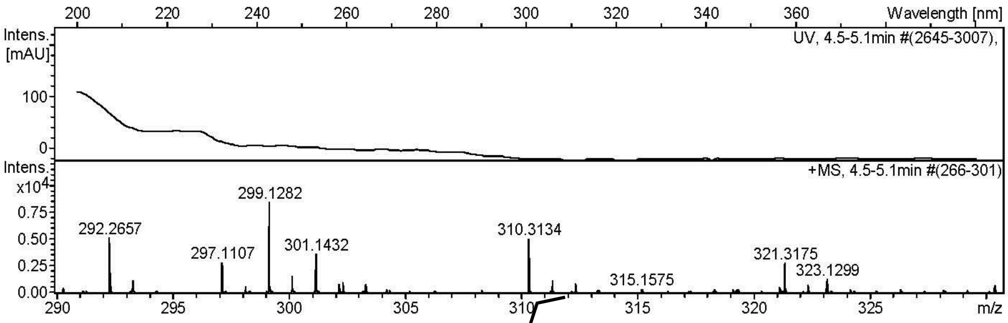

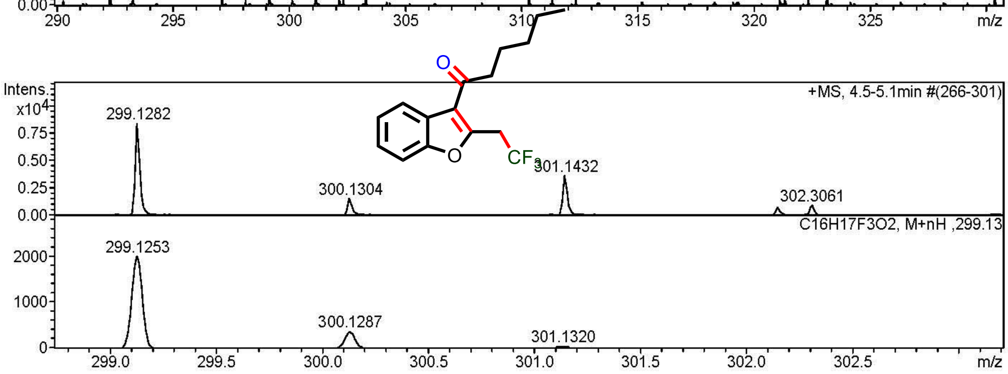

^{1}H NMR of phenyl(2-(2,2,2-trifluoroethyl)naphtho[2,1-b]furan-1-yl)methanone (**2p**)

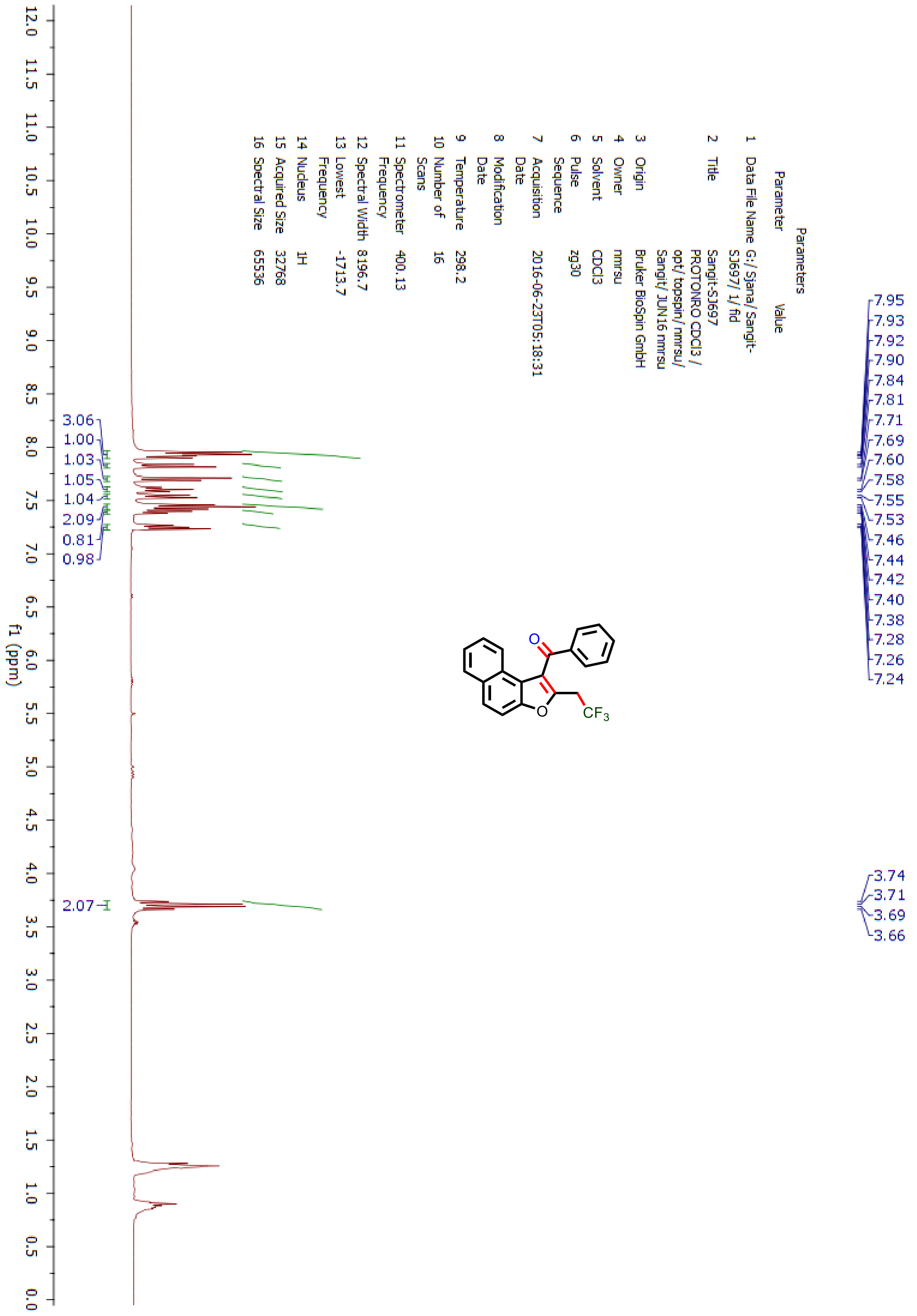

^{13}C NMR of phenyl(2-(2,2,2-trifluoroethyl)naphtho[2,1-b]furan-1-yl)methanone (**2p**)

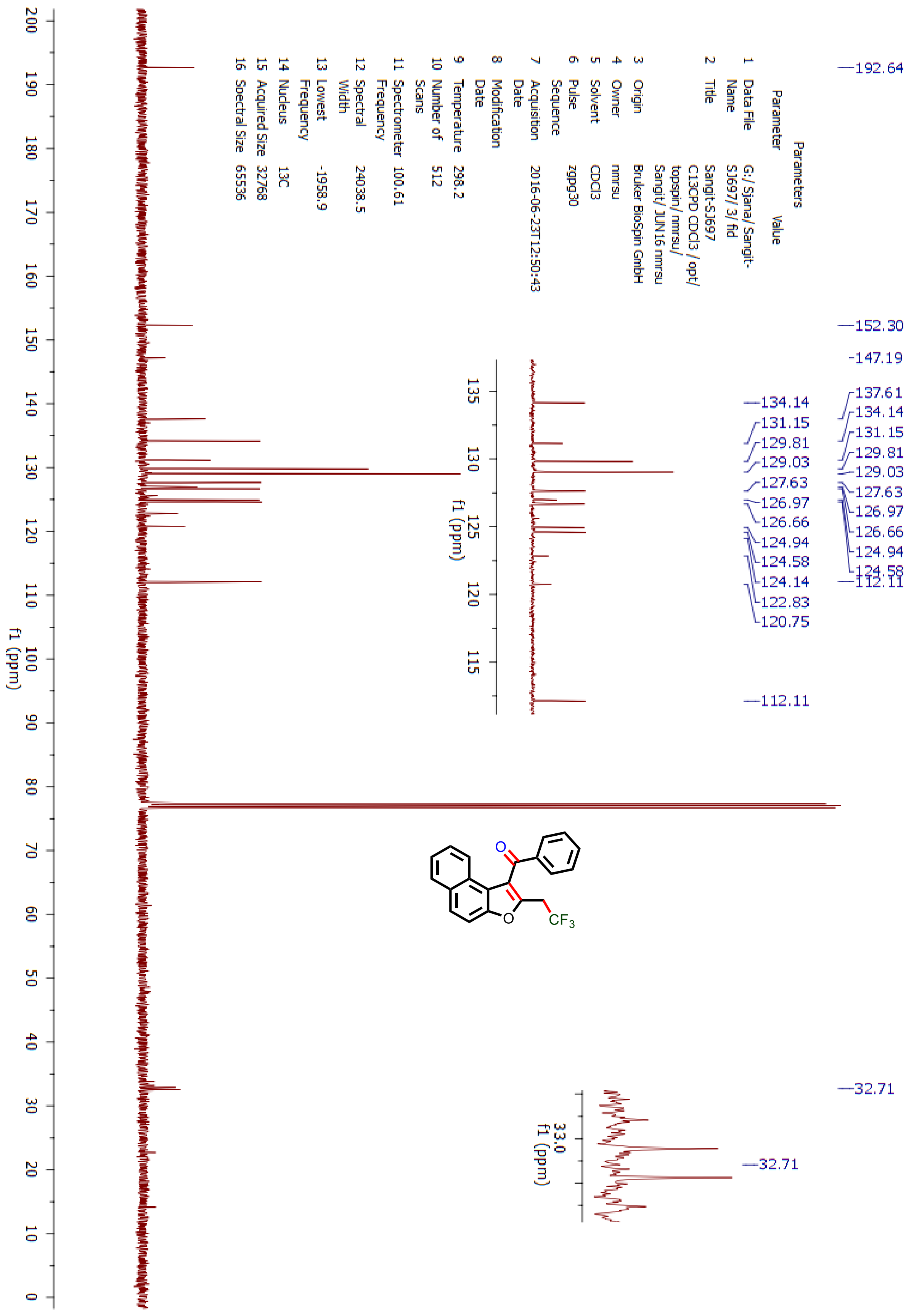

^{19}F NMR of phenyl(2-(2,2,2-trifluoroethyl)naphtho[2,1-b]furan-1-yl)methanone (**2p**)

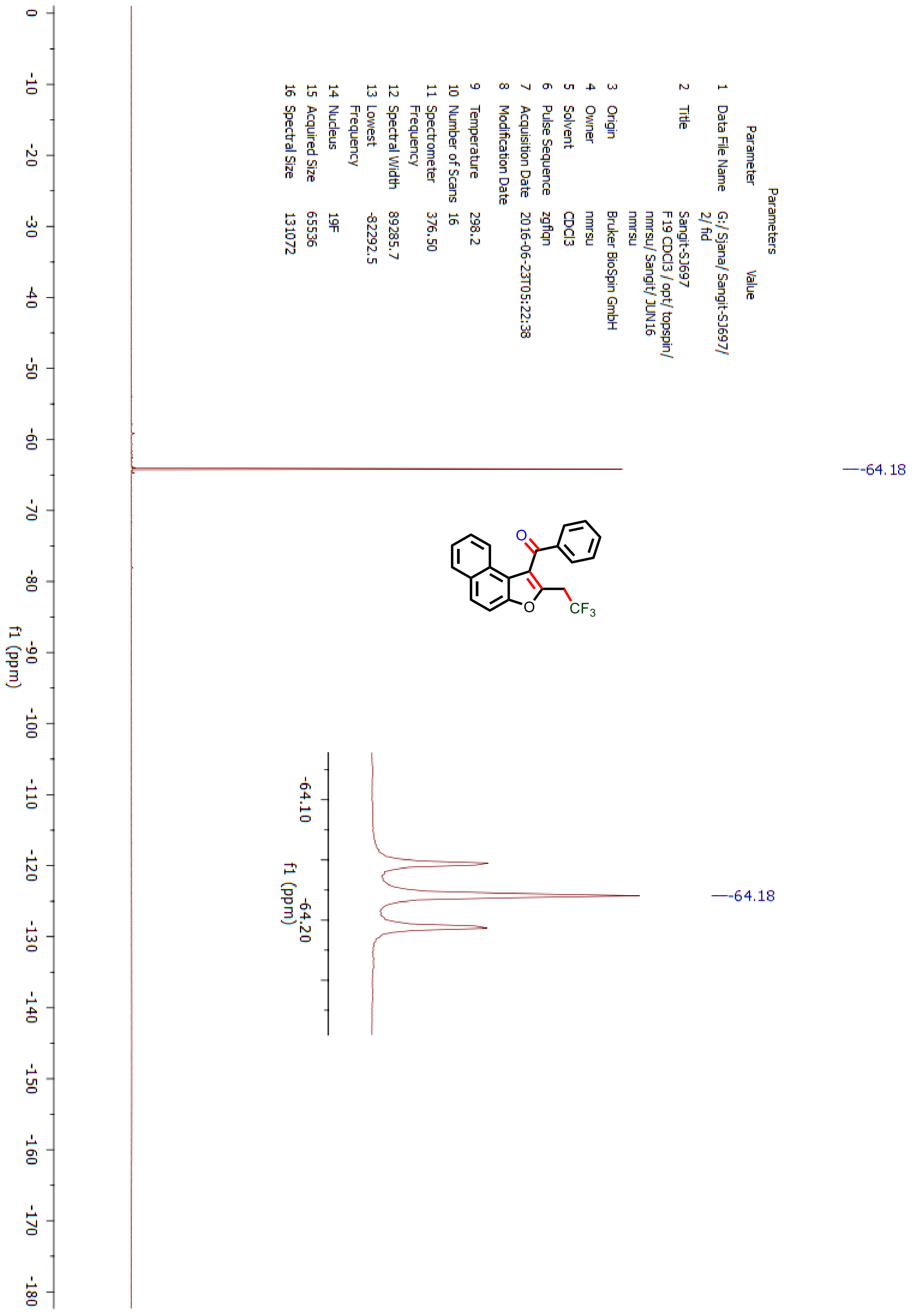

HRMS of phenyl(2-(2,2,2-trifluoroethyl)naphtho[2,1-b]furan-1-yl)methanone (**2p**)

Display Report

Analysis Info

		Acquisition Date	9/14/2016 4:08:50 PM
Analysis Name	D:\Data\user data\2016\SEPT-2016\14-09-2016\Dr S.Kumar-SJ-697_1-A,3_01_7386.d		
Method	hrlcms_pos_mid_tunemix.m	Operator	DIMPLE
Sample Name	Dr S.Kumar-SJ-697	Instrument	micrOTOF-Q II 10330
Comment			

Acquisition Parameter

Source Type	ESI	Ion Polarity	Positive	Set Nebulizer	0.3 Bar
Focus	Active	Set Capillary	4500 V	Set Dry Heater	200 °C
Scan Begin	50 m/z	Set End Plate Offset	-500 V	Set Dry Gas	4.0 l/min
Scan End	3000 m/z	Set Collision Cell RF	450.0 Vpp	Set Divert Valve	Waste

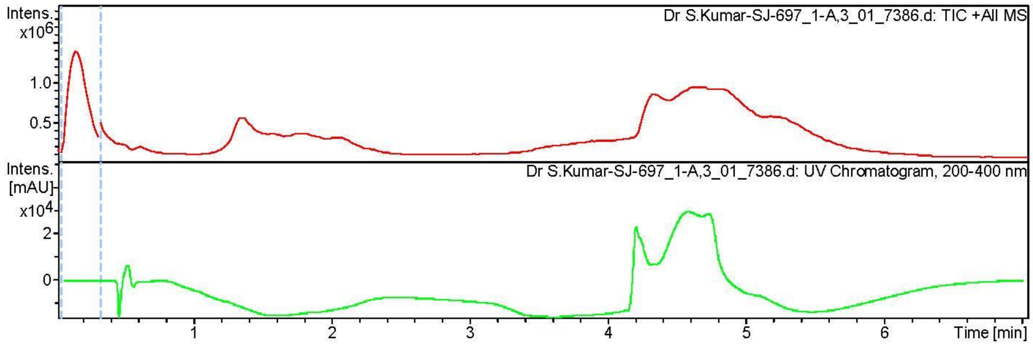

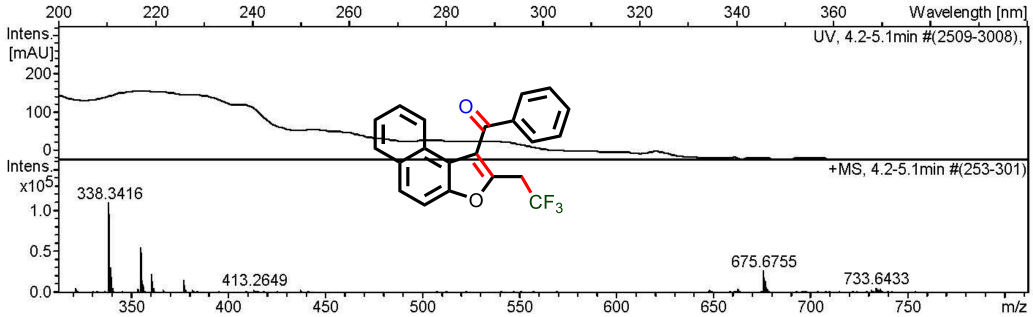

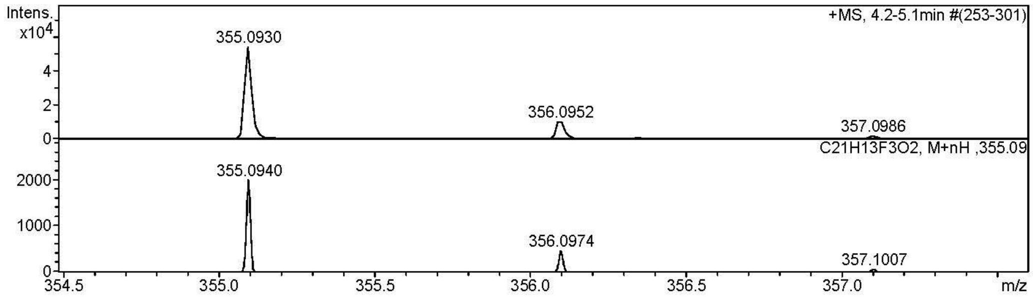

^{1}H NMR of (5-methyl-2-(2,2,2-trifluoroethyl)benzofuran-3-yl)(phenyl)methanone (**2q**)

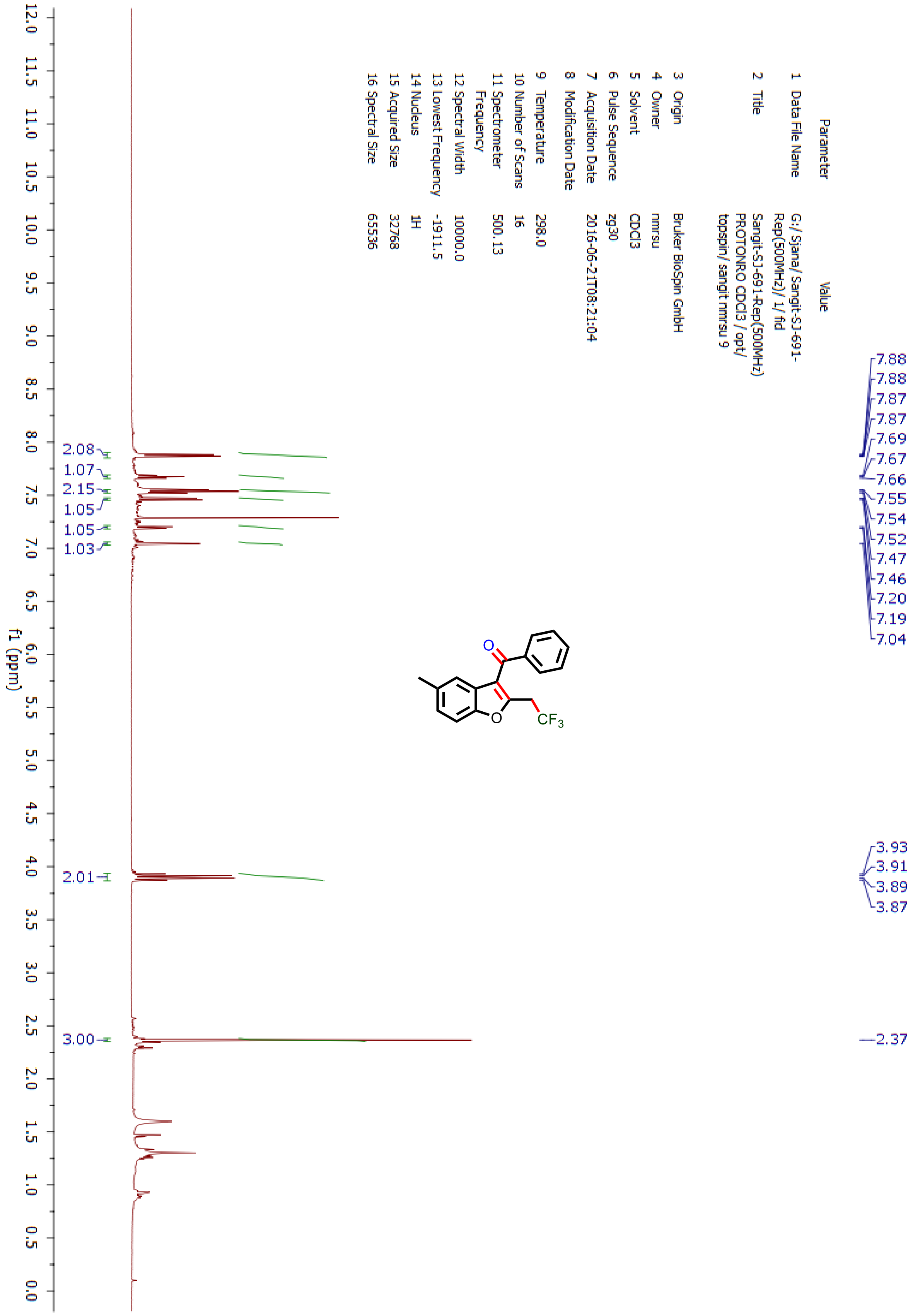

^{13}C NMR of (5-methyl-2-(2,2,2-trifluoroethyl)benzofuran-3-yl)(phenyl)methanone (**2q**)

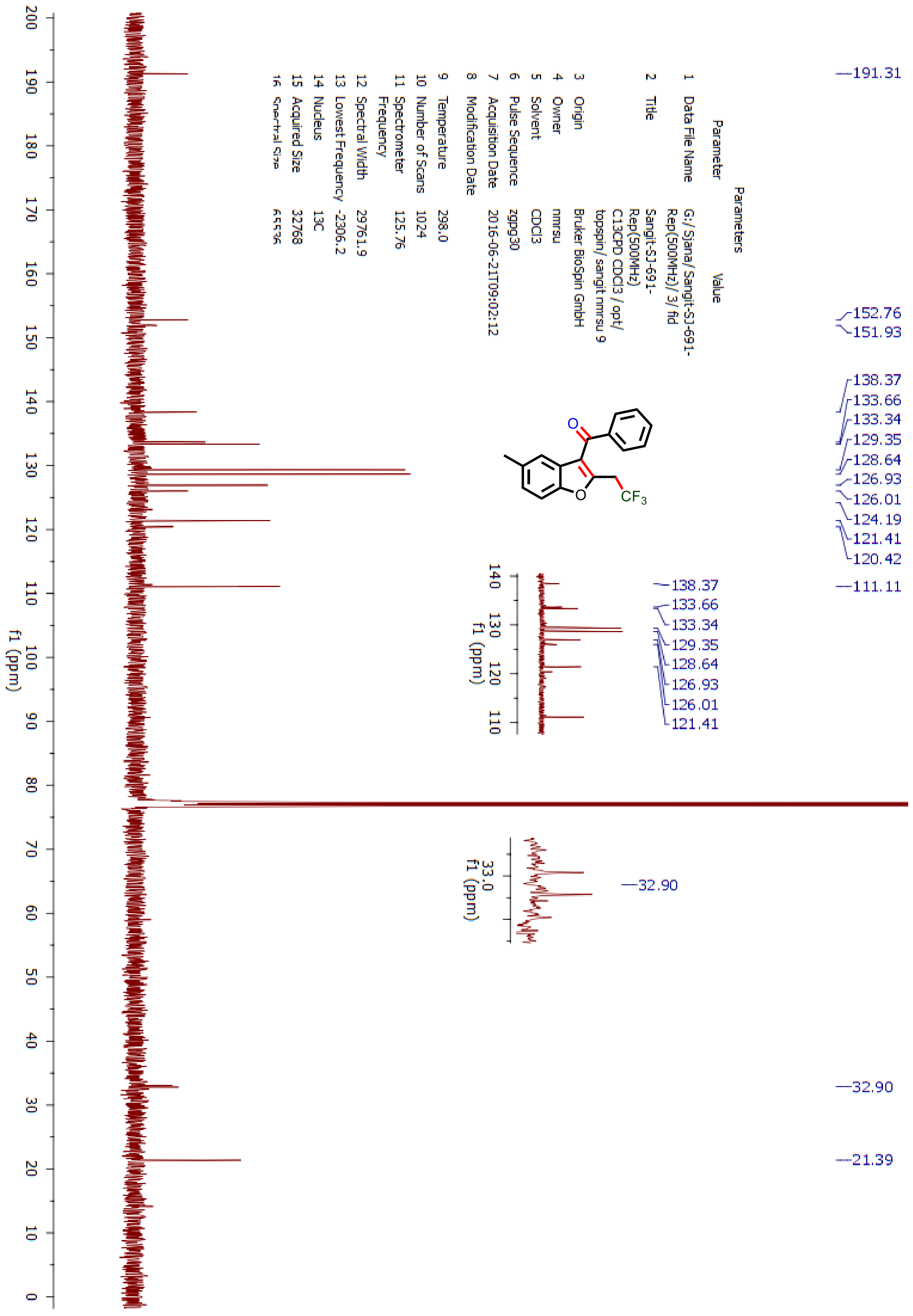

^{19}F NMR of (5-methyl-2-(2,2,2-trifluoroethyl)benzofuran-3-yl)(phenyl)methanone (**2q**)

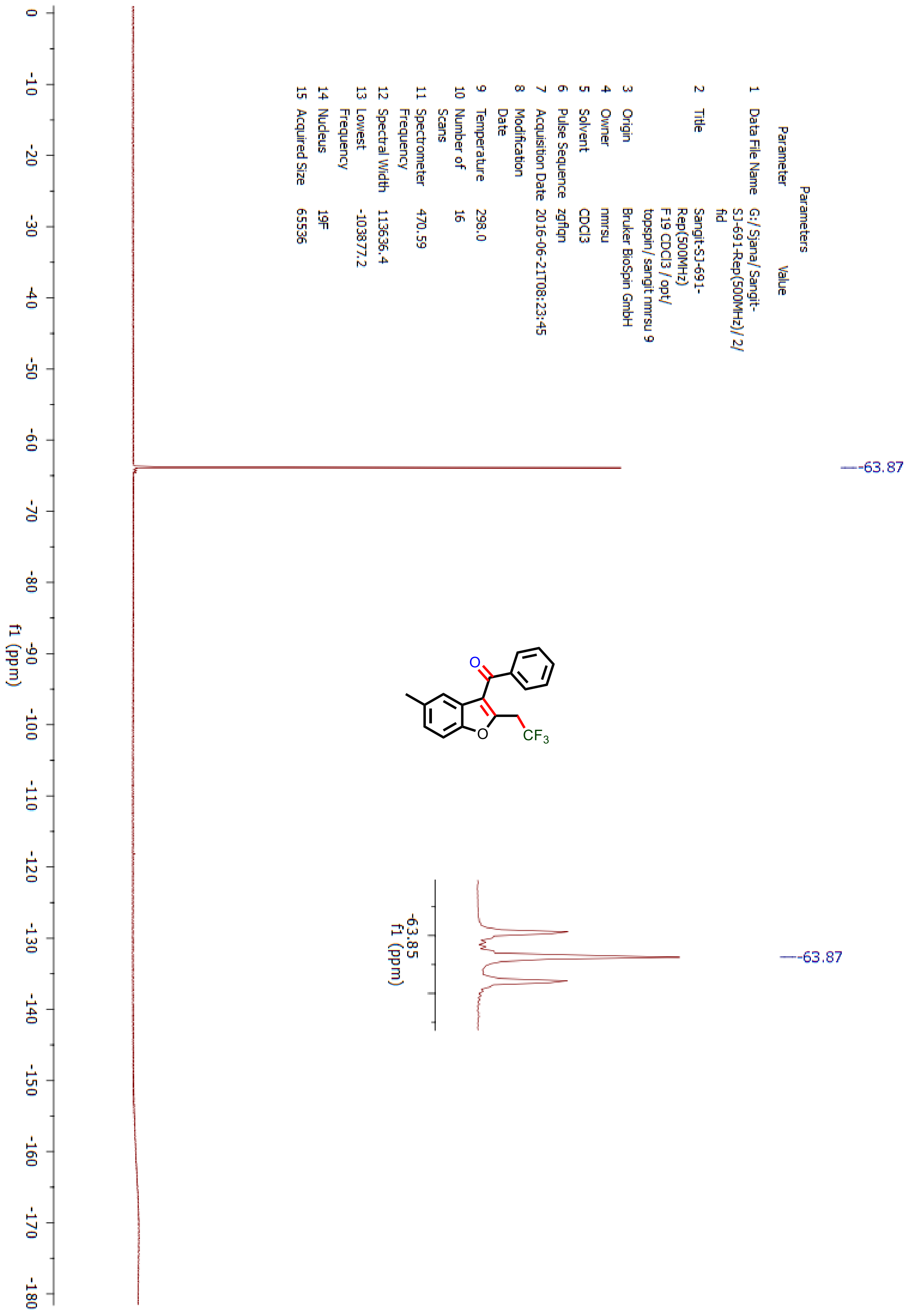

HRMS of (5-methyl-2-(2,2,2-trifluoroethyl)benzofuran-3-yl)(phenyl)methanone (**2q**)

Display Report

Analysis Info

Analysis Name	D:\Data\user data\2016\SEPT-2016\12-09-2016\Dr.S.Kumar-SJ-691_1-A,5_01_7366.d	Acquisition Date	9/12/2016 11:28:45 AM
Method	hrlcms_pos_mid_tunemix.m	Operator	DIMPLE
Sample Name	Dr.S.Kumar-SJ-691	Instrument	micrOTOF-Q II 10330
Comment			

Acquisition Parameter

Source Type	ESI	Ion Polarity	Positive	Set Nebulizer	0.3 Bar
Focus	Active	Set Capillary	4500 V	Set Dry Heater	200 °C
Scan Begin	50 m/z	Set End Plate Offset	-500 V	Set Dry Gas	4.0 l/min
Scan End	3000 m/z	Set Collision Cell RF	450.0 Vpp	Set Divert Valve	Waste

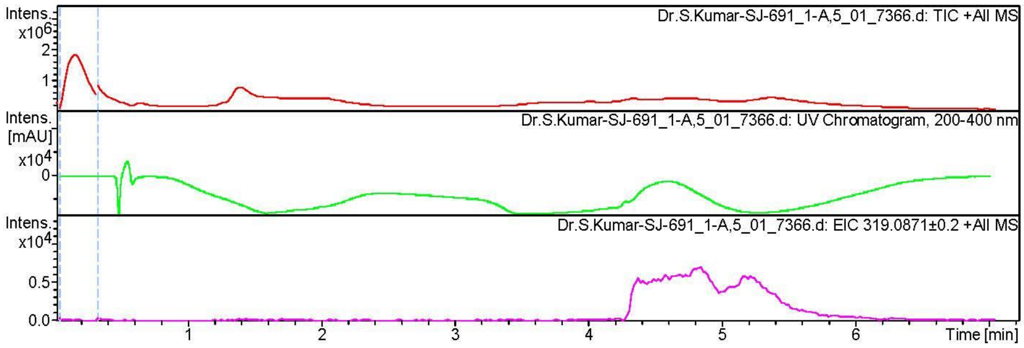

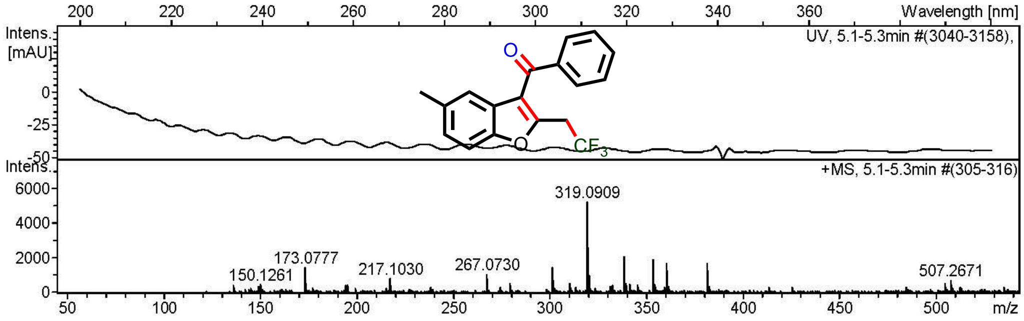

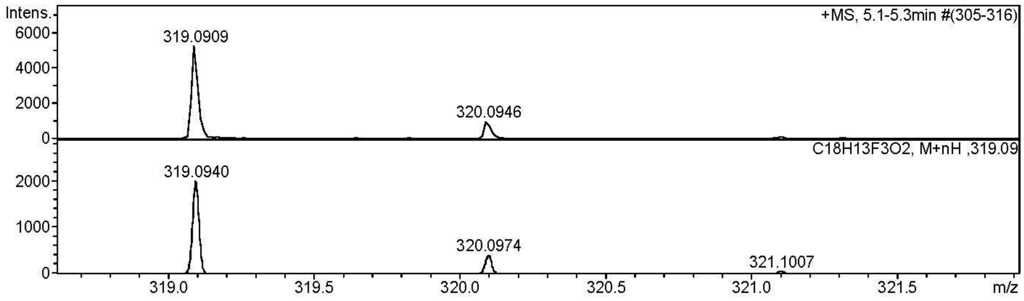

Bruker Compass DataAnalysis 4.0 printed: 9/12/2016 12:52:27 PM Page 1 of 1

^{1}H of (5-fluoro-2-(2,2,2-trifluoroethyl)benzofuran-3-yl)(phenyl)methanone (**2r**)

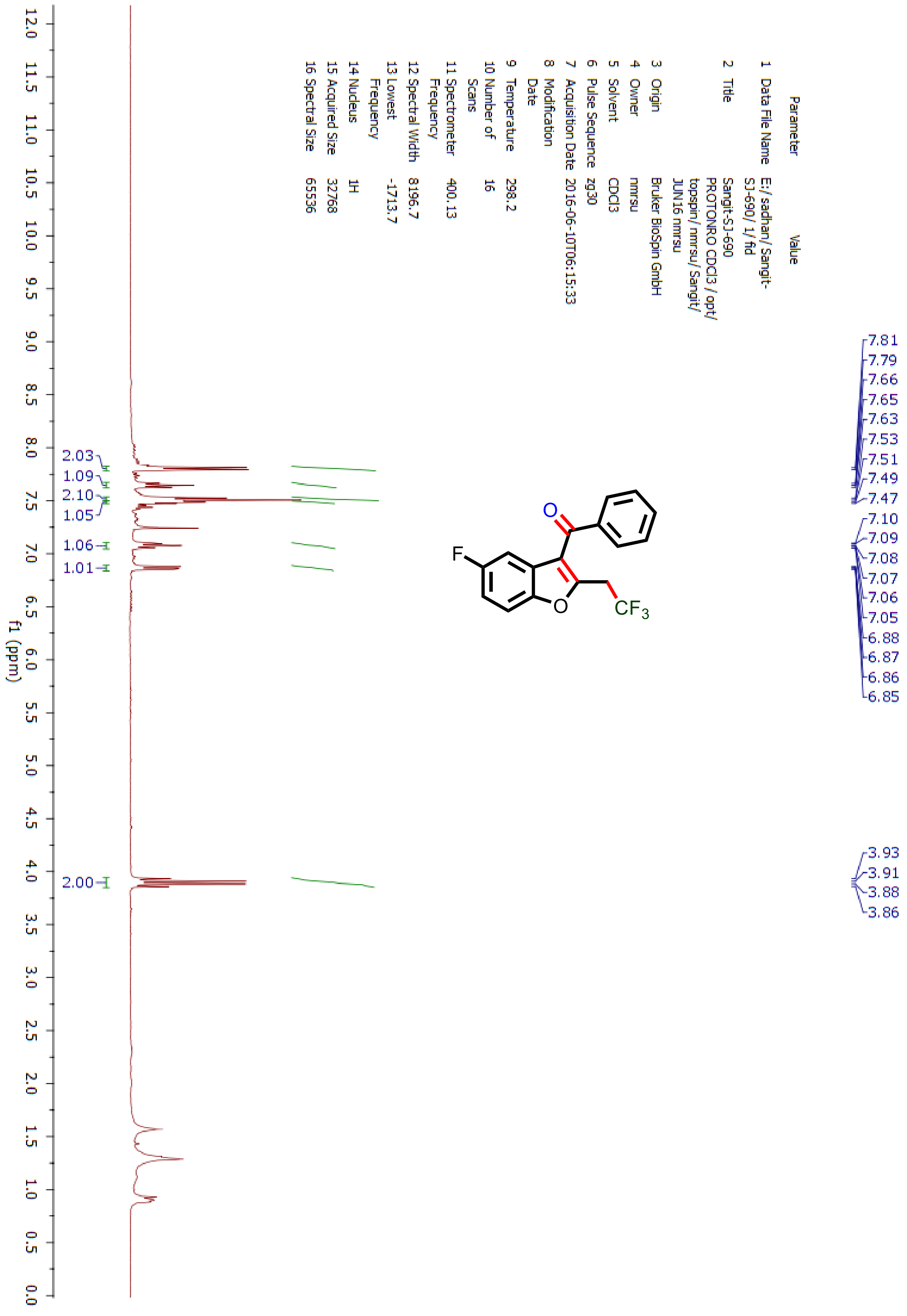

^{13}C of (5-fluoro-2-(2,2,2-trifluoroethyl)benzofuran-3-yl)(phenyl)methanone (**2r**)

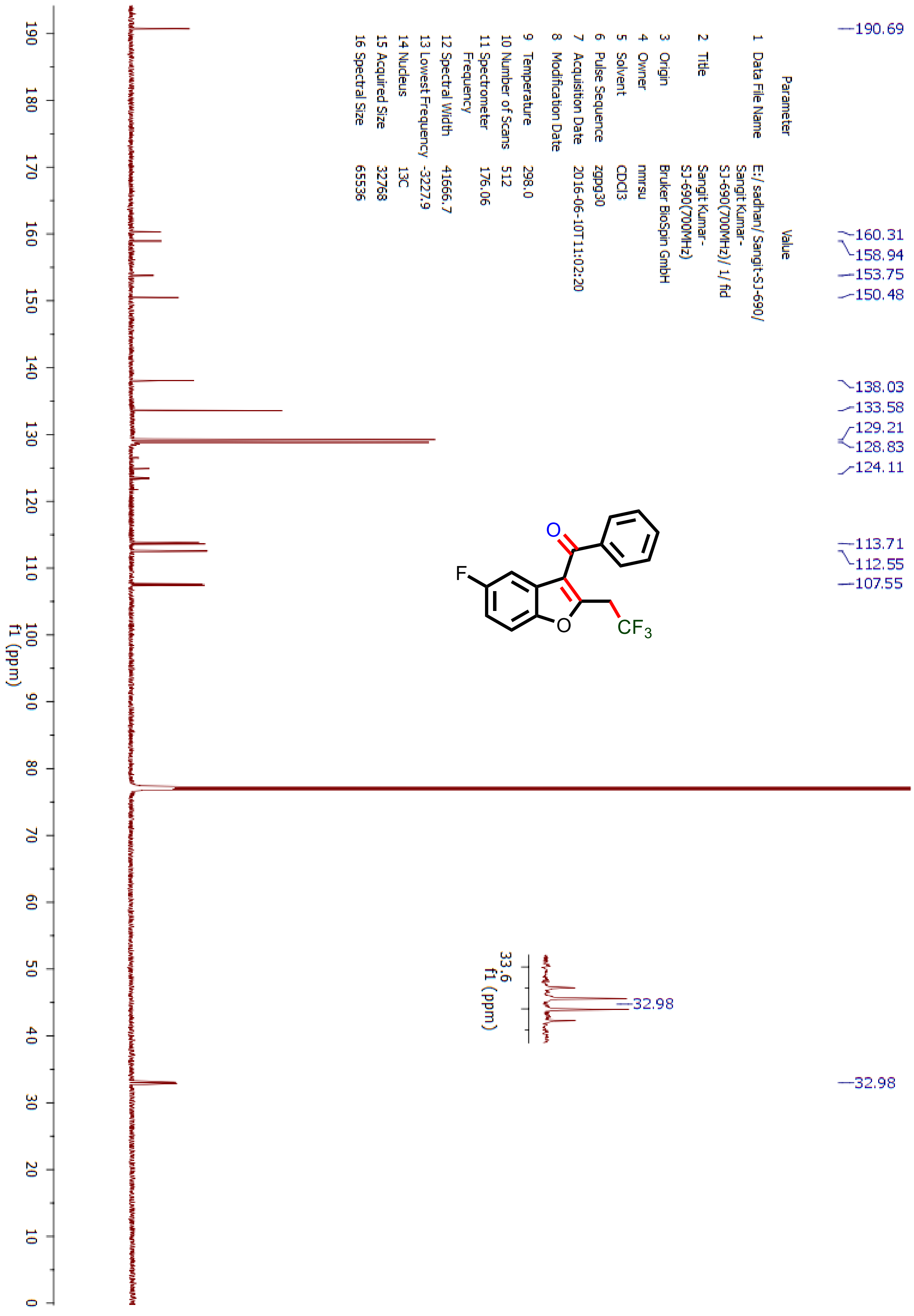

^{19}F of (5-fluoro-2-(2,2,2-trifluoroethyl)benzofuran-3-yl)(phenyl)methanone (**2r**)

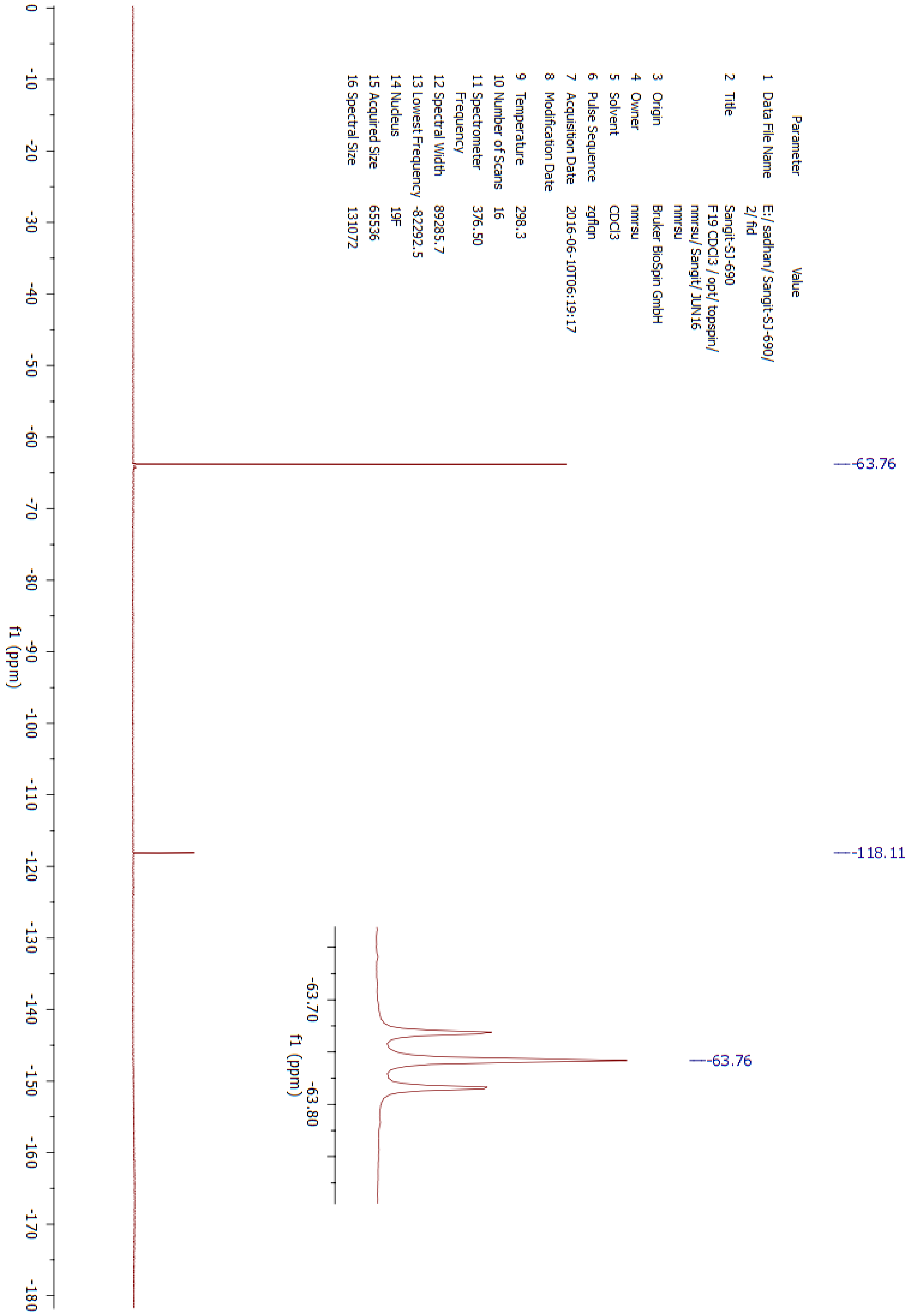

HRMS of (5-fluoro-2-(2,2,2-trifluoroethyl)benzofuran-3-yl)(phenyl)methanone (**2r**)

Display Report

Analysis Info

		Acquisition Date	9/12/2016 11:20:37 AM
Analysis Name	D:\Data\user data\2016\SEPT-2016\12-09-2016\Dr.S.Kumar-SJ-690_1-A,4_01_7365.d		
Method	hrlcms_pos_mid_tunemix.m	Operator	DIMPLE
Sample Name	Dr.S.Kumar-SJ-690	Instrument	micrOTOF-Q II 10330
Comment			

Acquisition Parameter

Source Type	ESI	Ion Polarity	Positive	Set Nebulizer	0.3 Bar
Focus	Active	Set Capillary	4500 V	Set Dry Heater	200 °C
Scan Begin	50 m/z	Set End Plate Offset	-500 V	Set Dry Gas	4.0 l/min
Scan End	3000 m/z	Set Collision Cell RF	450.0 Vpp	Set Divert Valve	Waste

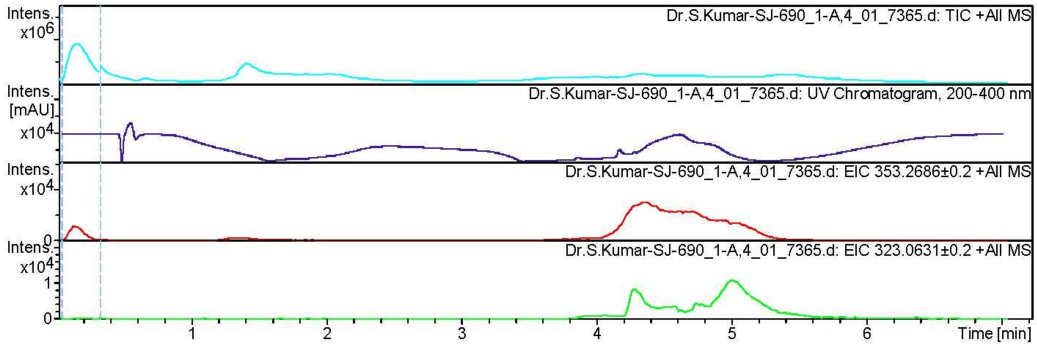

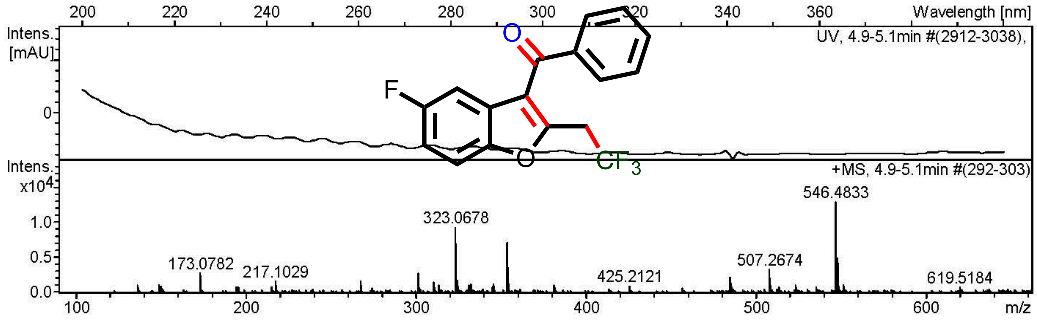

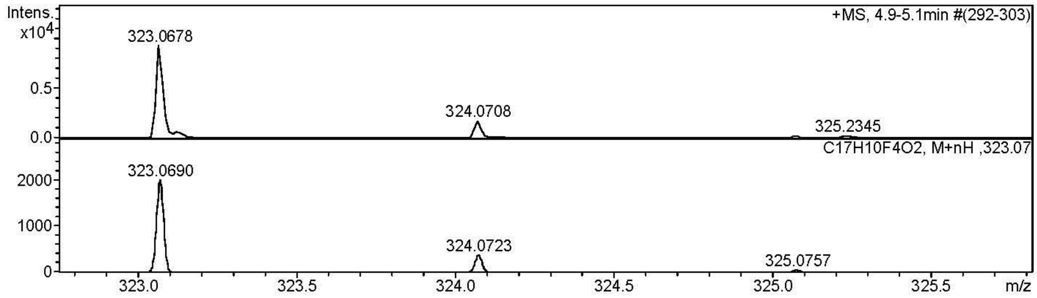

Bruker Compass DataAnalysis 4.0 printed: 9/12/2016 12:51:17 PM Page 1 of 1

^{1}H NMR of phenyl(2-(2,2,2-trifluoroethyl)benzo[*b*]thiophen-3-yl)methanone (**4a**)

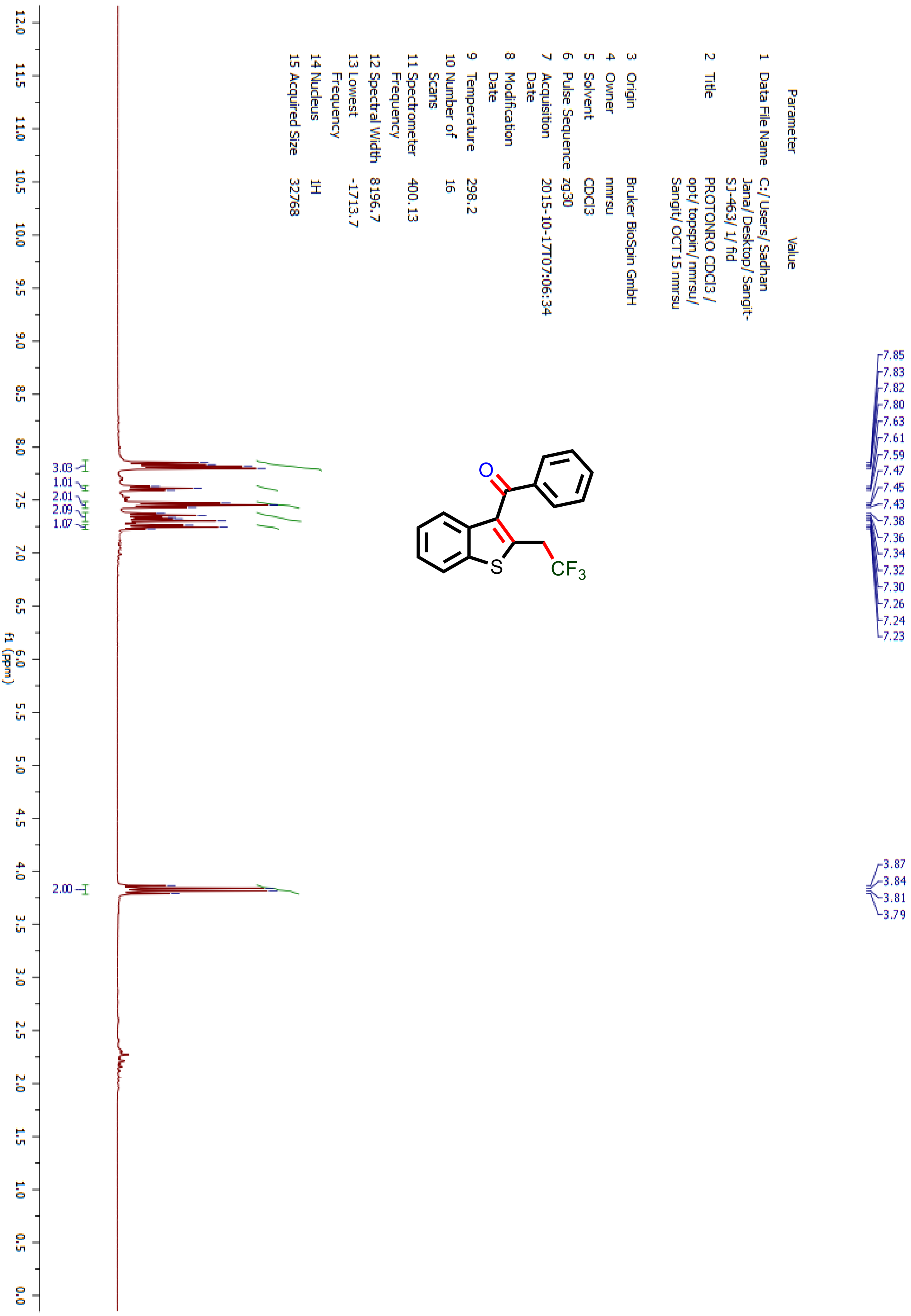

^{1}H NMR of phenyl(2-(2,2,2-trifluoroethyl)benzo[*b*]thiophen-3-yl)methanone (**4a**)

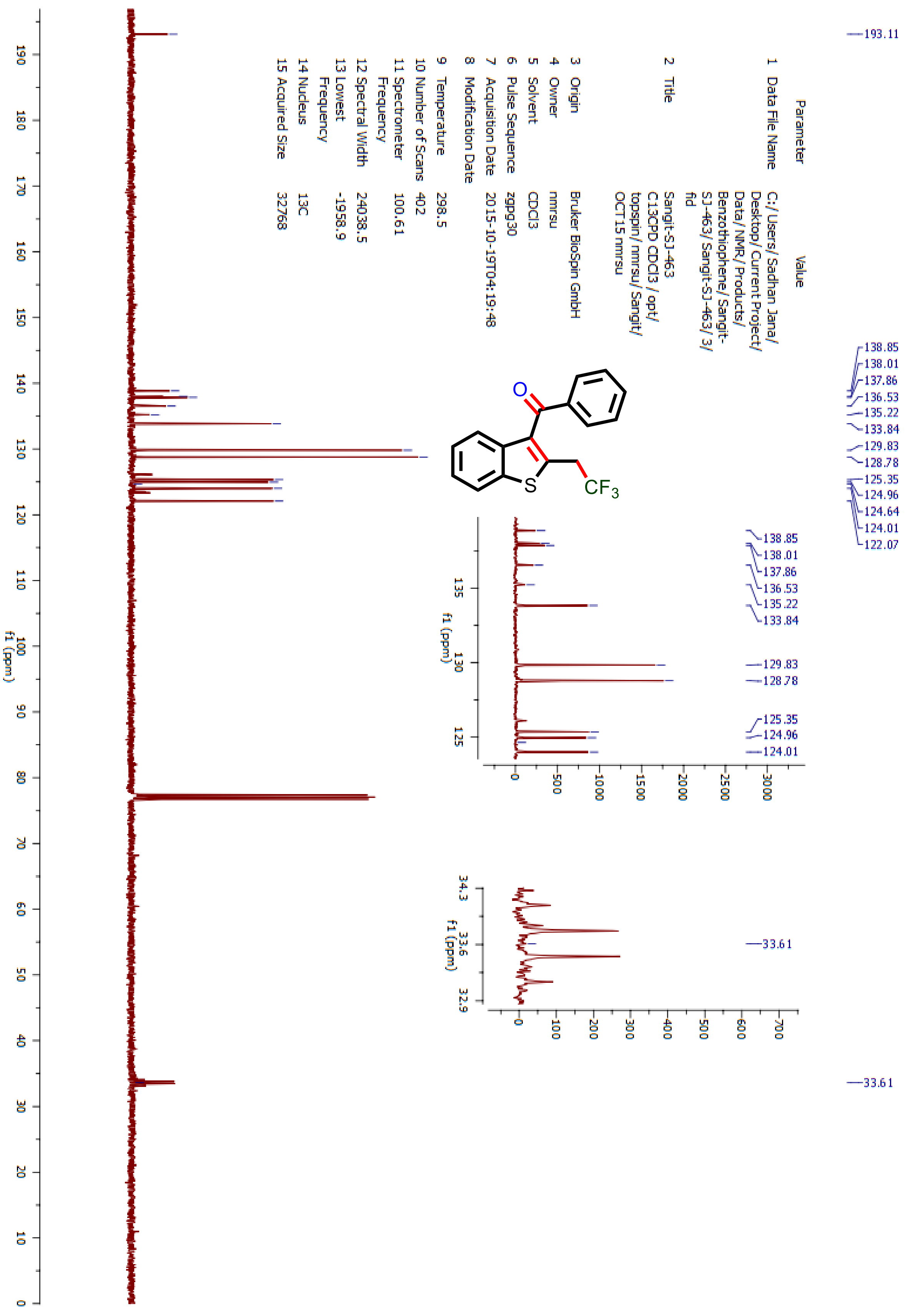

^{19}F NMR of phenyl(2-(2,2,2-trifluoroethyl)benzo[*b*]thiophen-3-yl)methanone (**4a**)

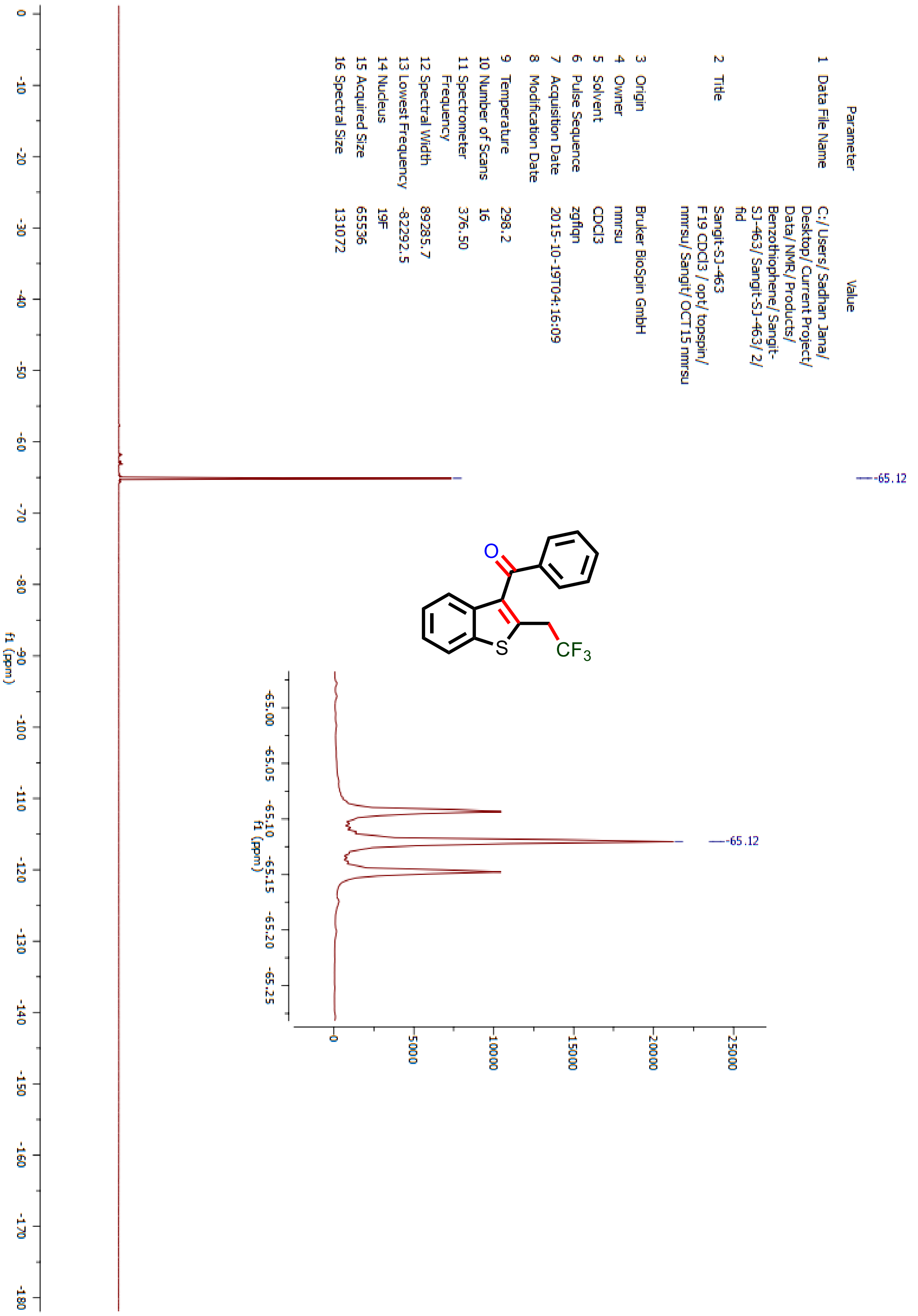

HRMS of phenyl(2-(2,2,2-trifluoroethyl)benzo[*b*]thiophen-3-yl)methanone (**4a**)

Display Report

Analysis Info

Analysis Name	D:\Data\user data\2015\Oct-2015\19-OCT-2015\Dr.S.Kumar-SJ-463_1-C,2_01_4041.d	Acquisition Date	10/19/2015 5:27:11 PM
Method	HRLCMS-20 Sept.m	Operator	RUCHI
Sample Name	Dr.S.Kumar-SJ-463	Instrument	micrOTOF-Q II 10330
Comment			

Acquisition Parameter

Source Type	ESI	Ion Polarity	Positive	Set Nebulizer	1.2 Bar
Focus	Active	Set Capillary	4500 V	Set Dry Heater	200 °C
Scan Begin	50 m/z	Set End Plate Offset	-500 V	Set Dry Gas	7.0 l/min
Scan End	3000 m/z	Set Collision Cell RF	130.0 Vpp	Set Divert Valve	Waste

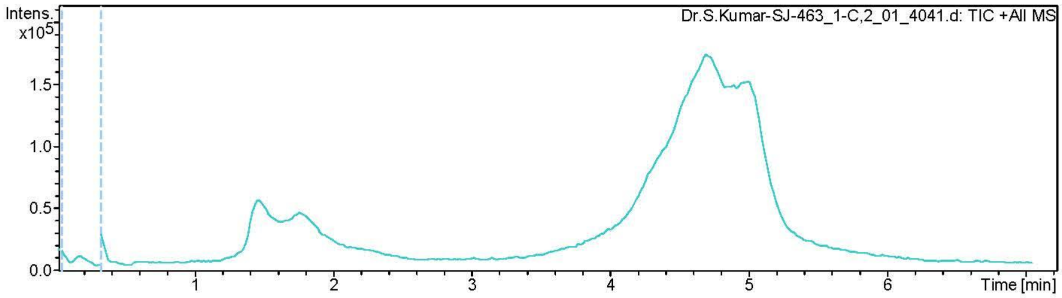

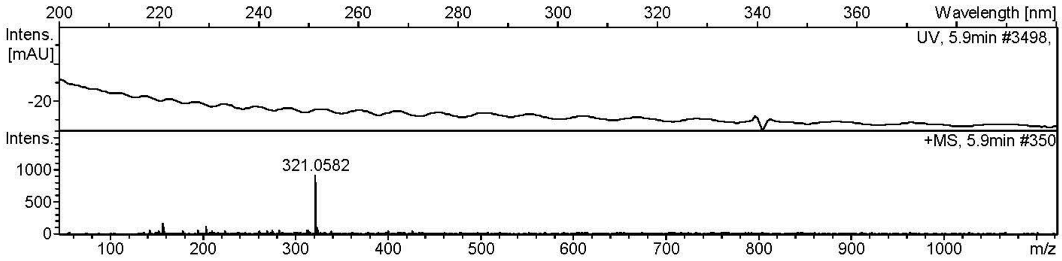

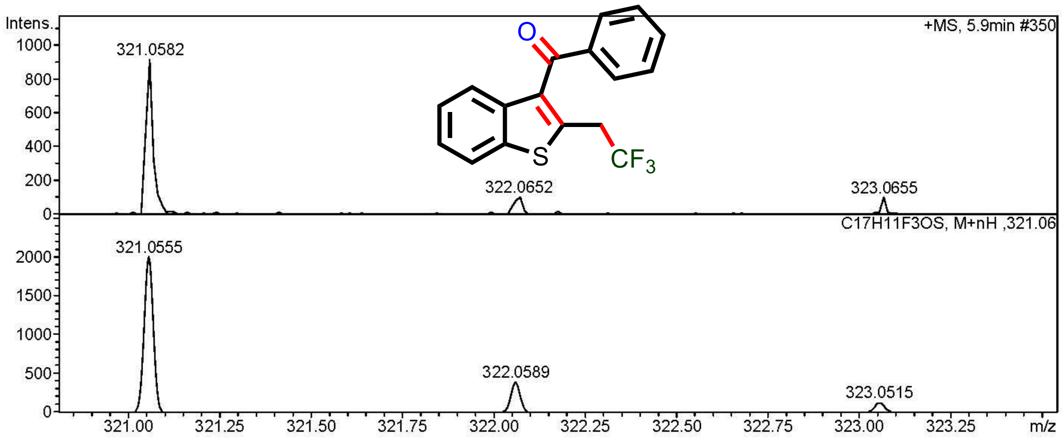

^{1}H NMR of (4-chlorophenyl)(2-(2,2,2-trifluoroethyl)benzo[*b*]thiophen-3-yl)methanone (4b)

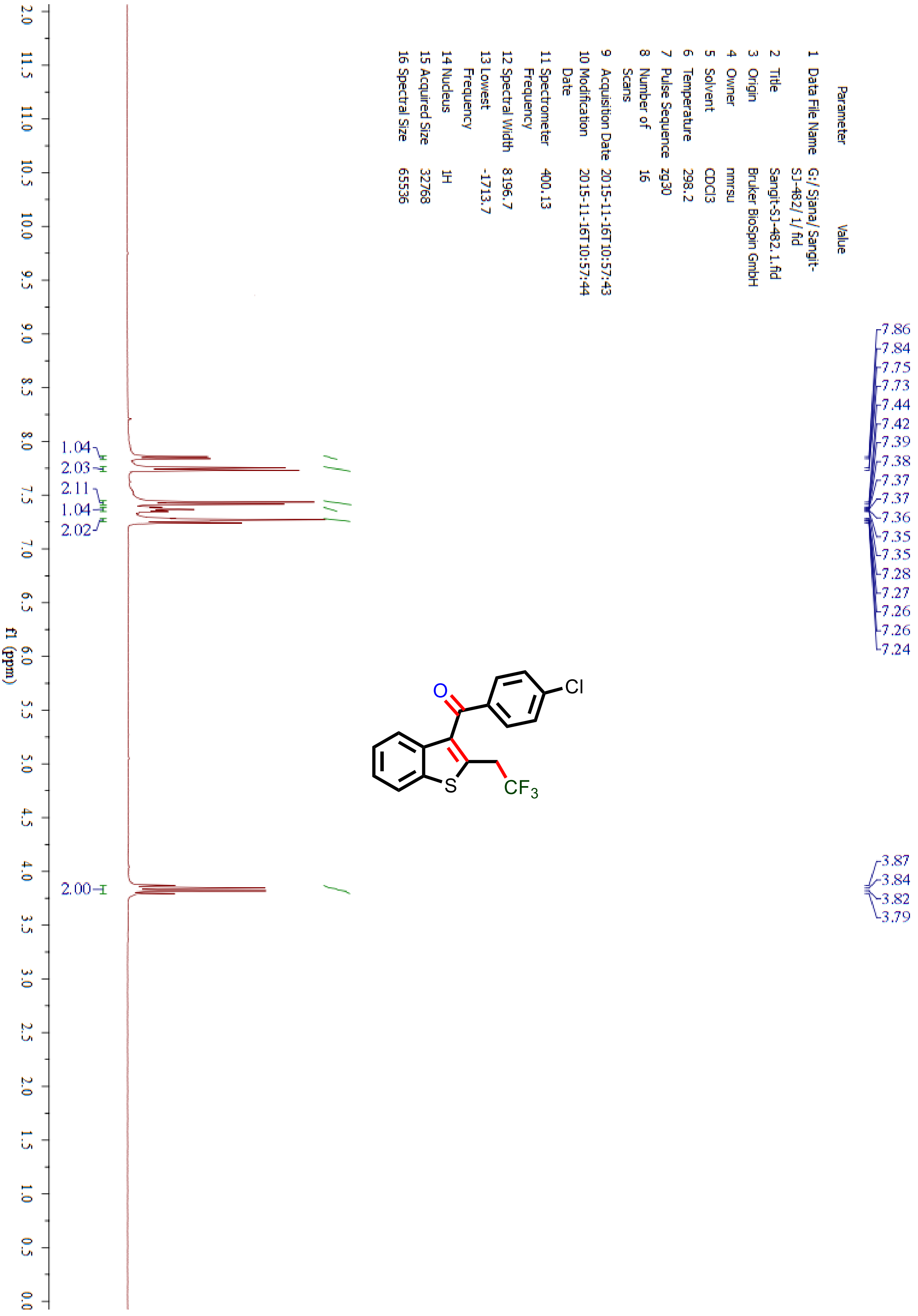

^{13}C NMR of (4-chlorophenyl)(2-(2,2,2-trifluoroethyl)benzo[*b*]thiophen-3-yl)methanone (4b)

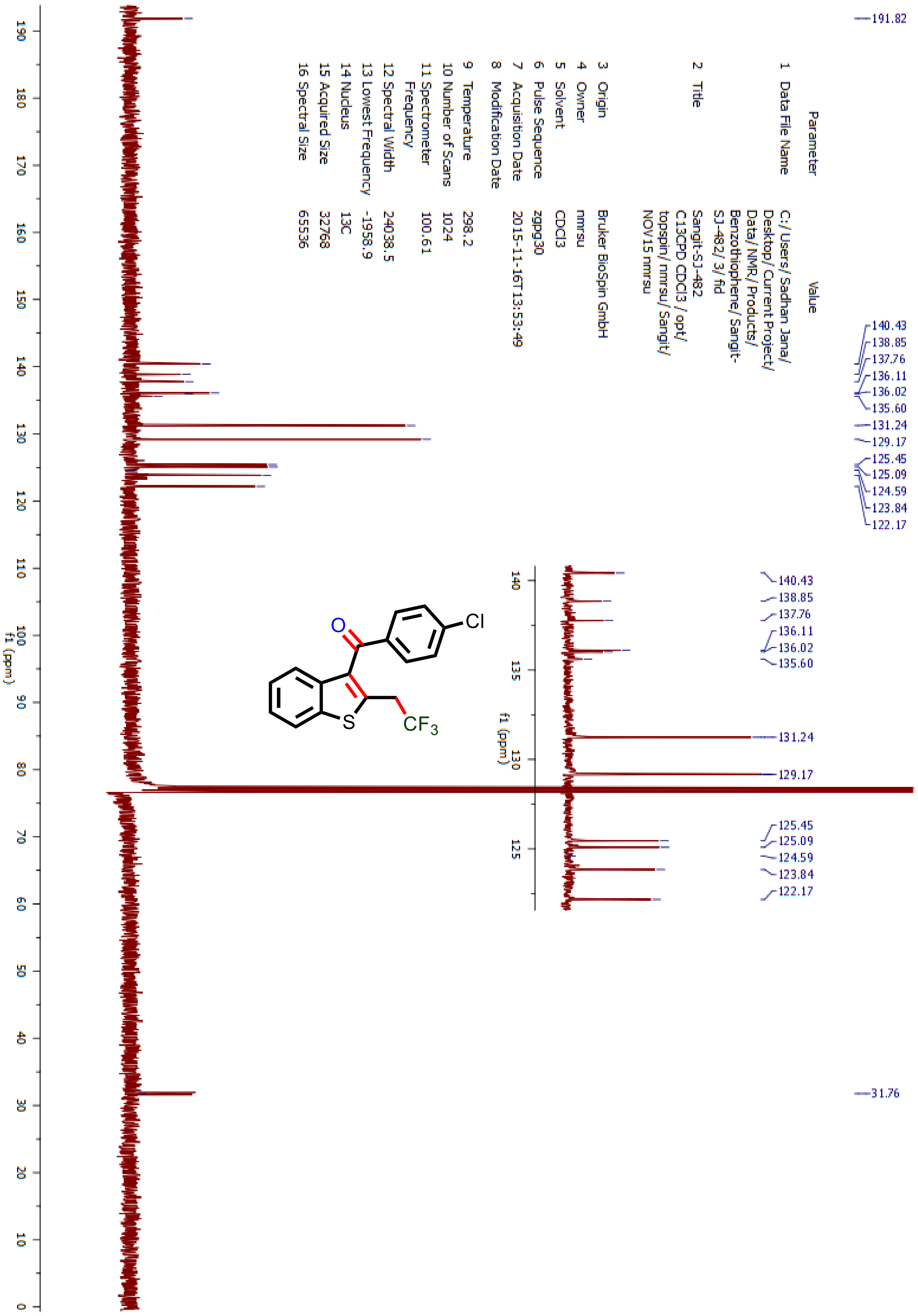

^{19}F NMR of (4-chlorophenyl)(2-(2,2,2-trifluoroethyl)benzo[*b*]thiophen-3-yl)methanone (4b)

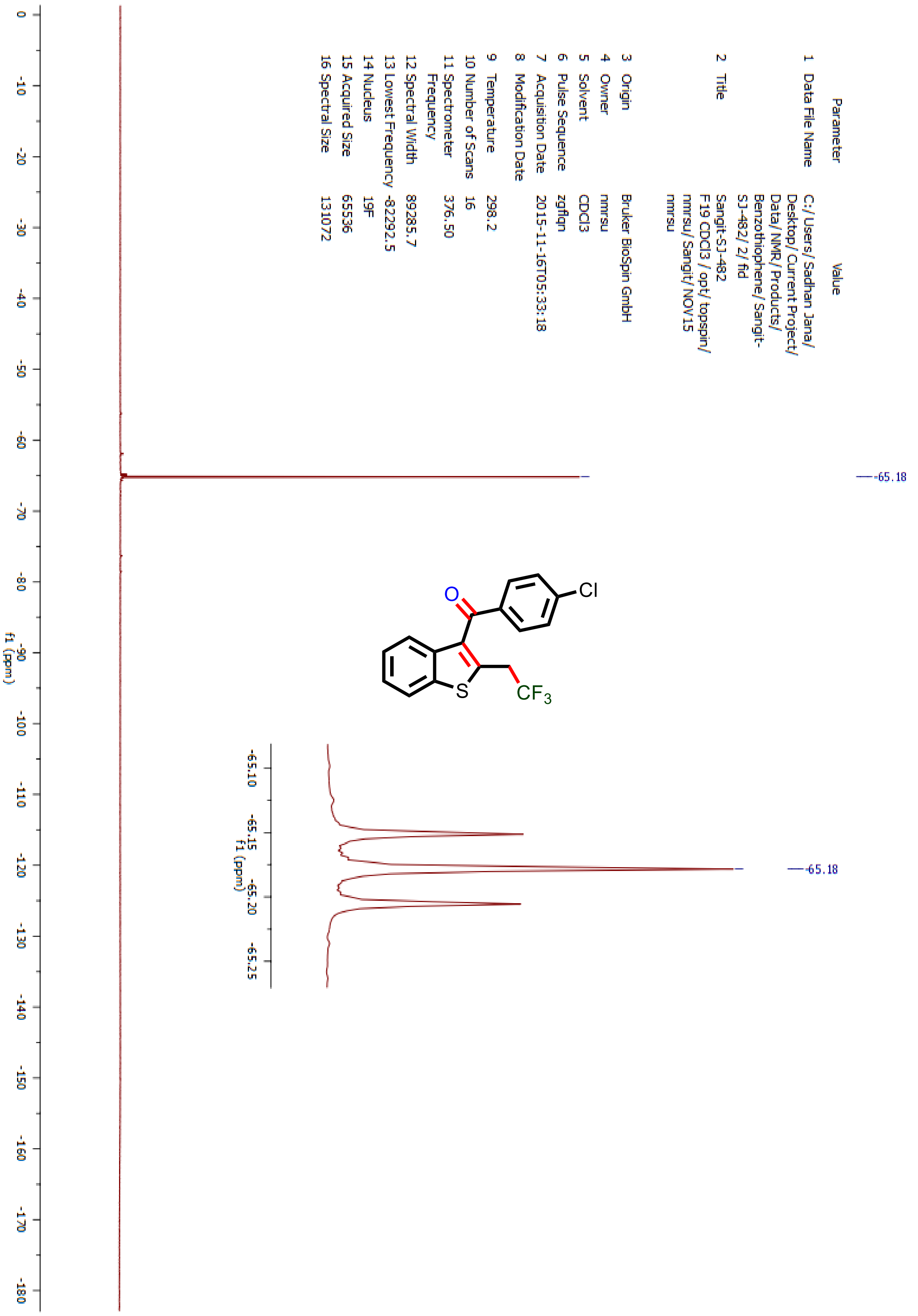

HRMS of (4-chlorophenyl)(2-(2,2,2-trifluoroethyl)benzo[*b*]thiophen-3-yl)methanone (4b)

Display Report

Analysis Info

		Acquisition Date	11/27/2015 4:37:25 PM
Analysis Name	D:\Data\user data\2015\NOV-2015\27-NOV-2015\Dr.S.Kumar-SJ-482_1-B,3_01_4357.d		
Method	HRLCMS-20 Sept.m	Operator	RUCHI
Sample Name	Dr.S.Kumar-SJ-482	Instrument	micrOTOF-Q II 10330
Comment			

Acquisition Parameter

Source Type	ESI	Ion Polarity	Positive	Set Nebulizer	1.2 Bar
Focus	Active	Set Capillary	4500 V	Set Dry Heater	200 °C
Scan Begin	50 m/z	Set End Plate Offset	-500 V	Set Dry Gas	7.0 l/min
Scan End	3000 m/z	Set Collision Cell RF	130.0 Vpp	Set Divert Valve	Waste

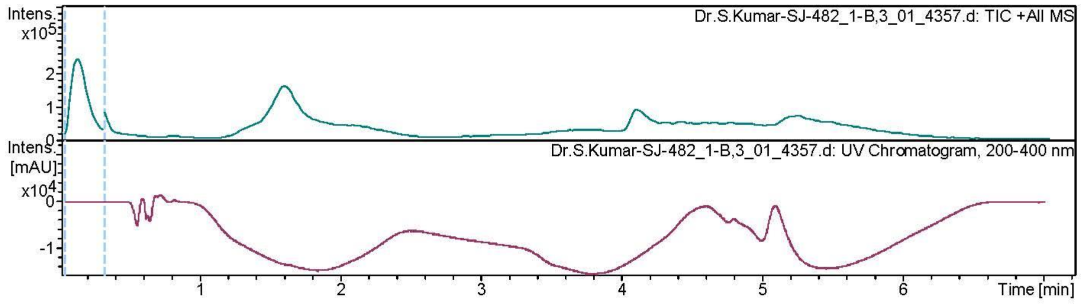

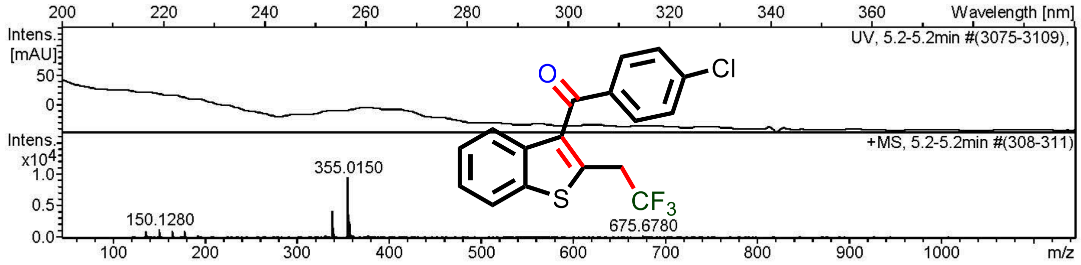

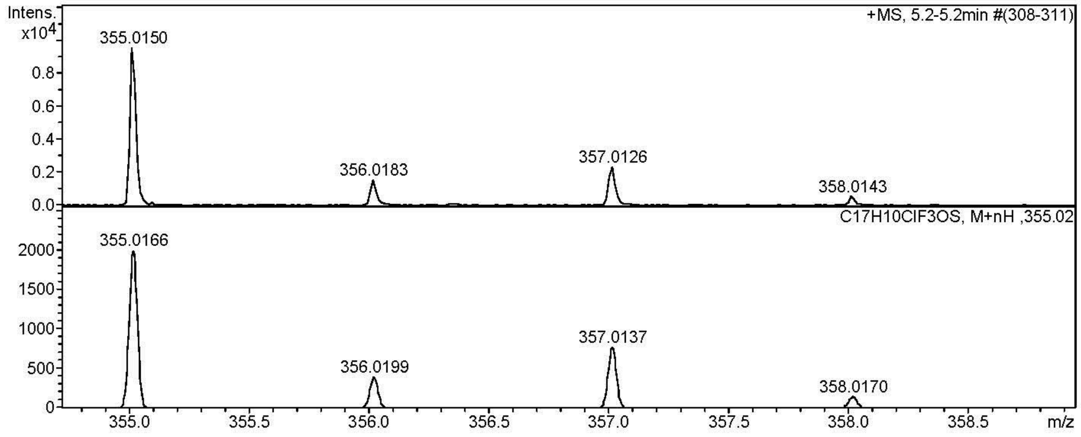

Bruker Compass DataAnalysis 4.0 printed: 11/27/2015 5:01:48 PM Page 1 of 1

^{1}H NMR of p-tolyl(2-(2,2,2-trifluoroethyl)benzo[*b*]thiophen-3-yl)methanone (4c)

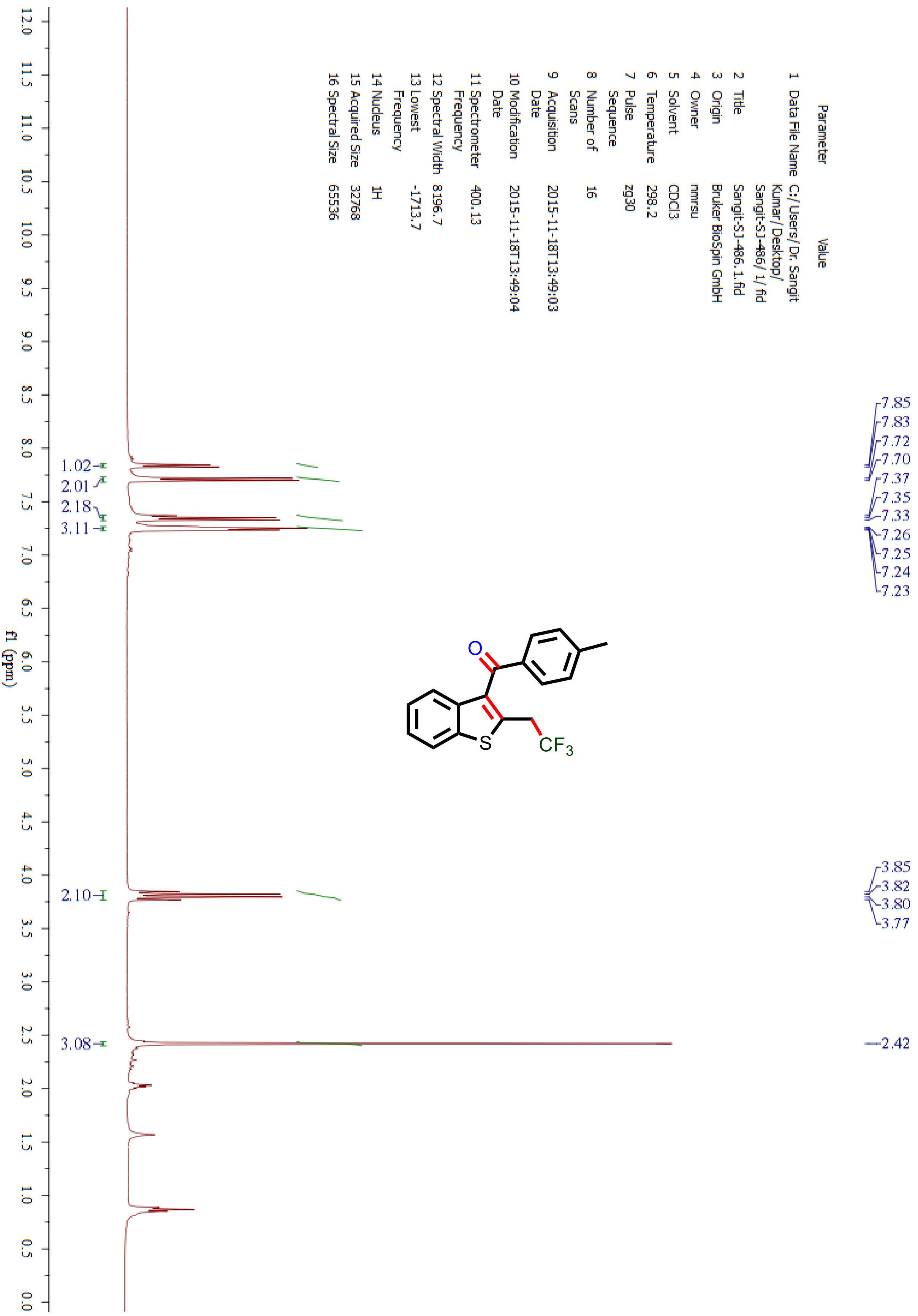

^{13}C NMR of p-tolyl(2-(2,2,2-trifluoroethyl)benzo[*b*]thiophen-3-yl)methanone (4c)

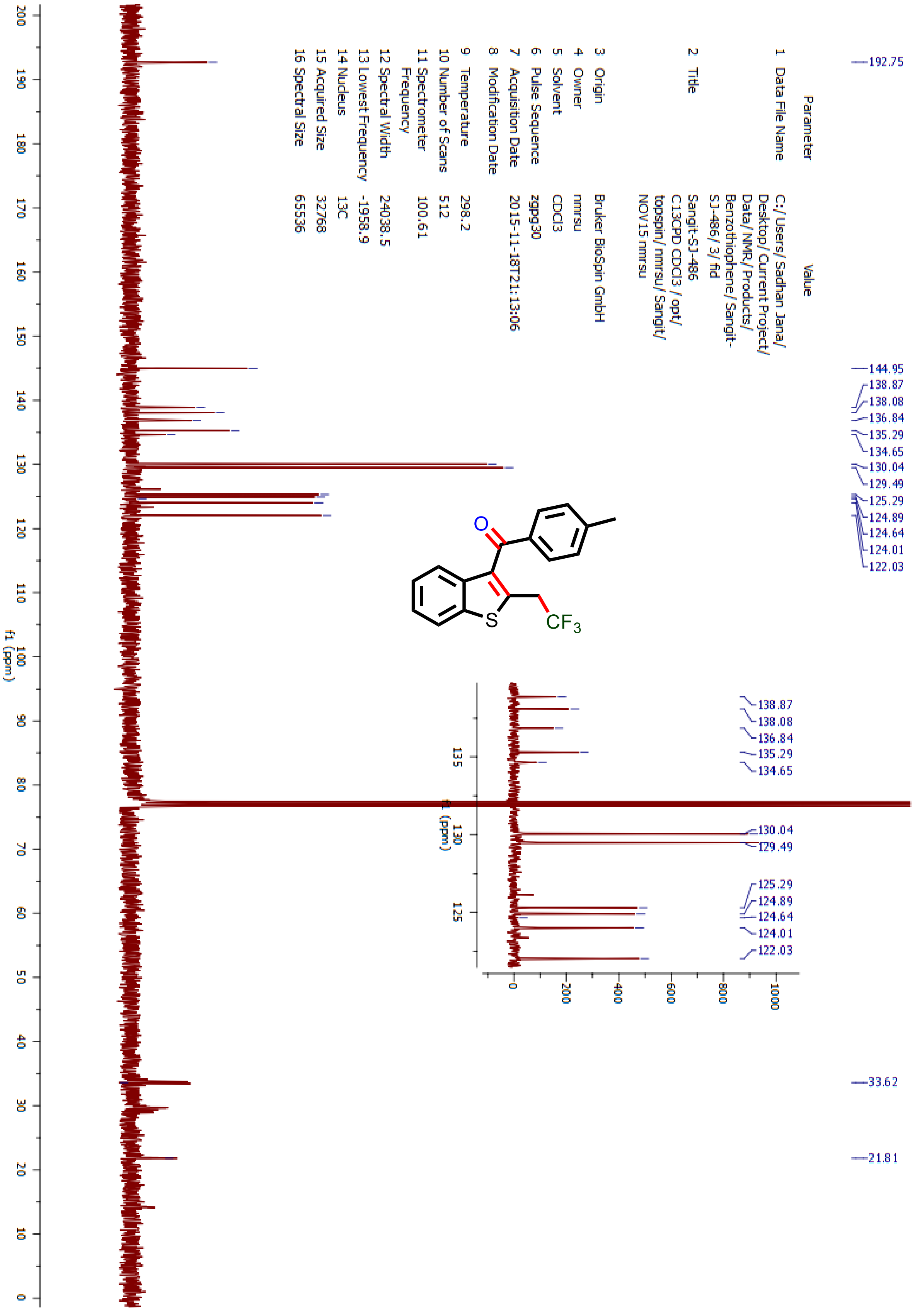

^{19}F NMR of p-tolyl(2-(2,2,2-trifluoroethyl)benzo[*b*]thiophen-3-yl)methanone (4c)

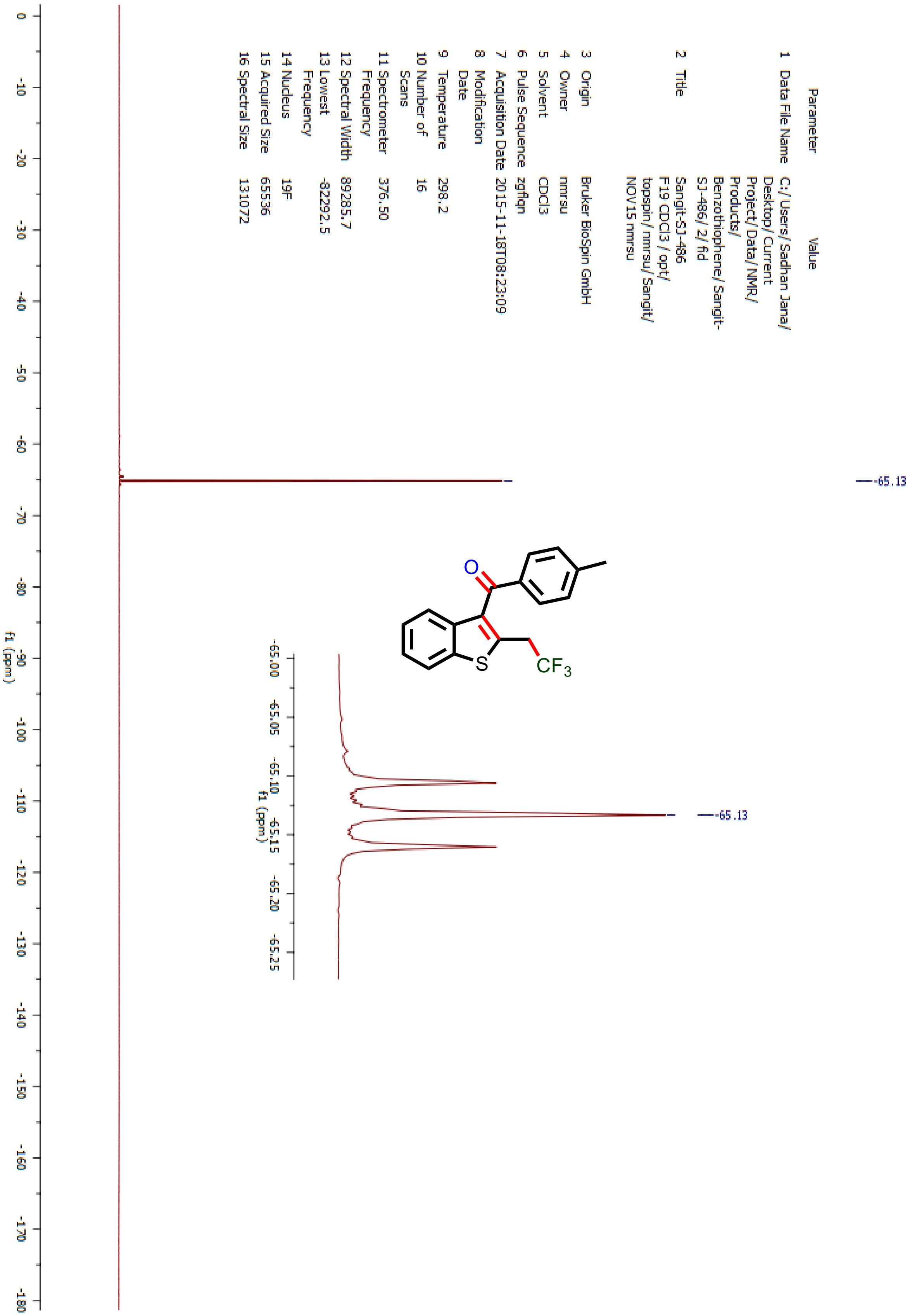

HRMS of p-tolyl(2-(2,2,2-trifluoroethyl)benzo[*b*]thiophen-3-yl)methanone (4c)

Display Report

Analysis Info

		Acquisition Date	11/27/2015 4:45:34 PM
Analysis Name	D:\Data\user data\2015\NOV-2015\27-NOV-2015\Dr.S.Kumar-SJ-486_1-B,4_01_4358.d		
Method	HRLCMS-20 Sept.m	Operator	RUCHI
Sample Name	Dr.S.Kumar-SJ-486	Instrument	micrOTOF-Q II 10330
Comment			

Acquisition Parameter

Source Type	ESI	Ion Polarity	Positive	Set Nebulizer	1.2 Bar
Focus	Active	Set Capillary	4500 V	Set Dry Heater	200 °C
Scan Begin	50 m/z	Set End Plate Offset	-500 V	Set Dry Gas	7.0 l/min
Scan End	3000 m/z	Set Collision Cell RF	130.0 Vpp	Set Divert Valve	Waste

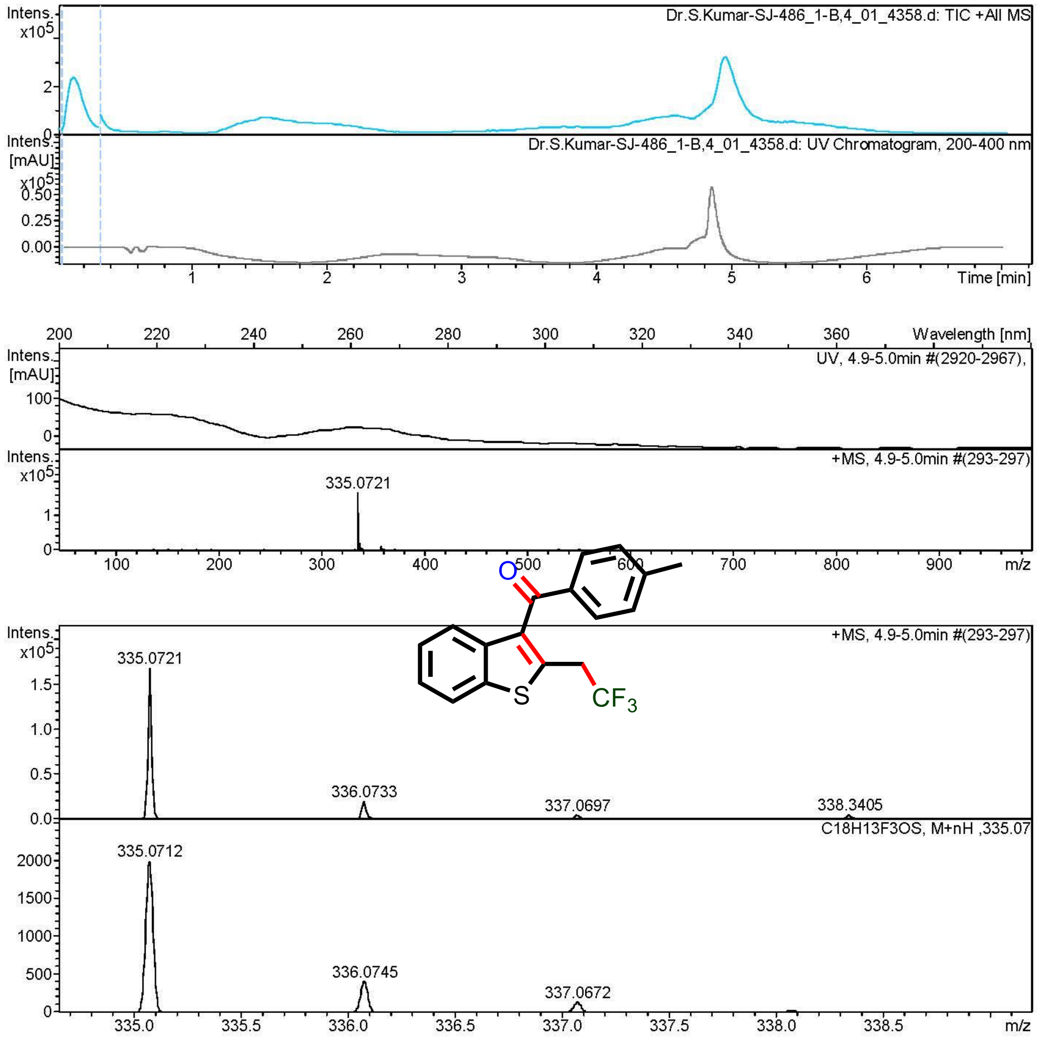

^{1}H NMR of (3-fluorophenyl)(2-(2,2,2-trifluoroethyl)benzo[*b*]thiophen-3-yl)methanone (4d)

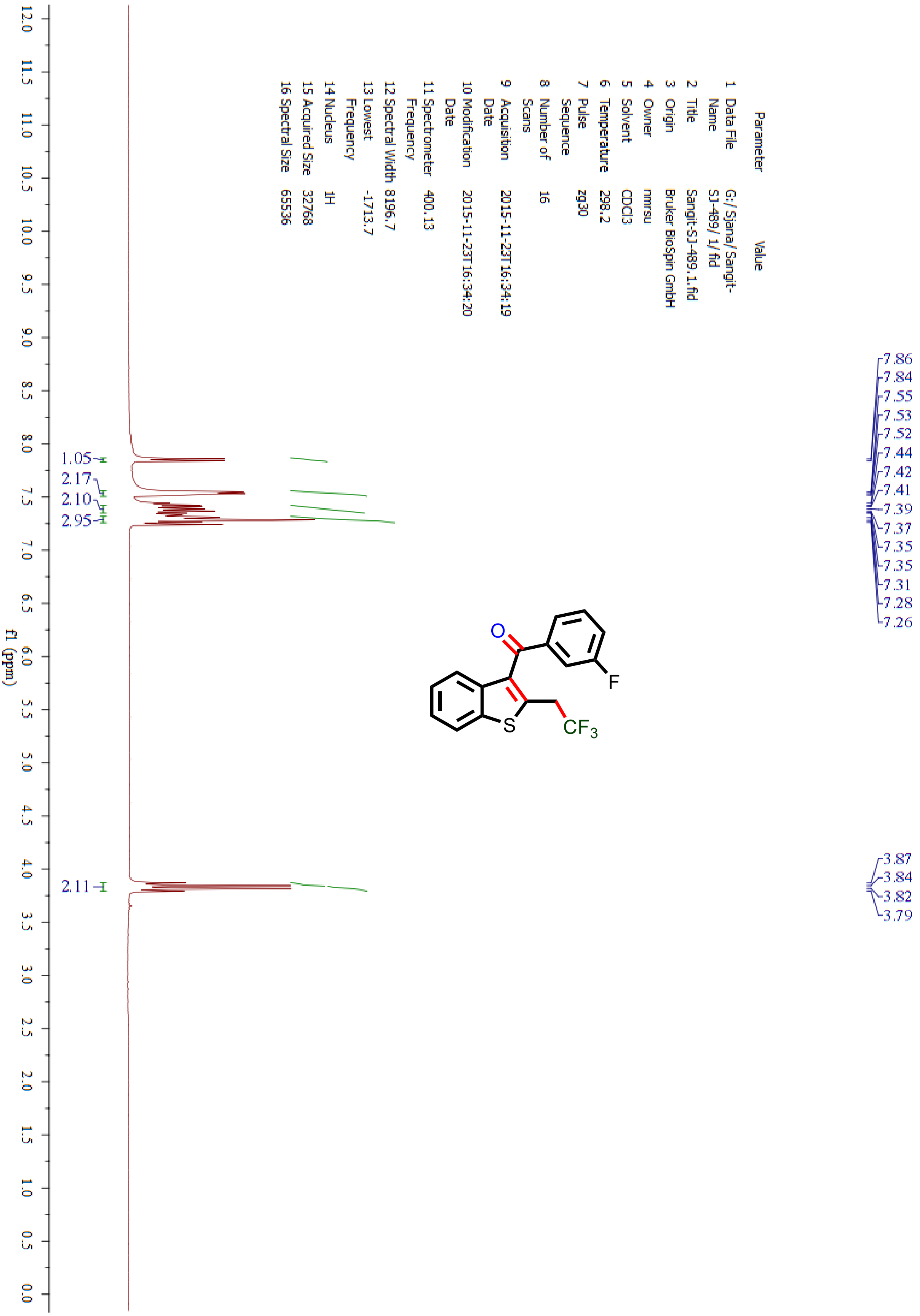

^{13}C NMR of (3-fluorophenyl)(2-(2,2,2-trifluoroethyl)benzo[*b*]thiophen-3-yl)methanone (4d)

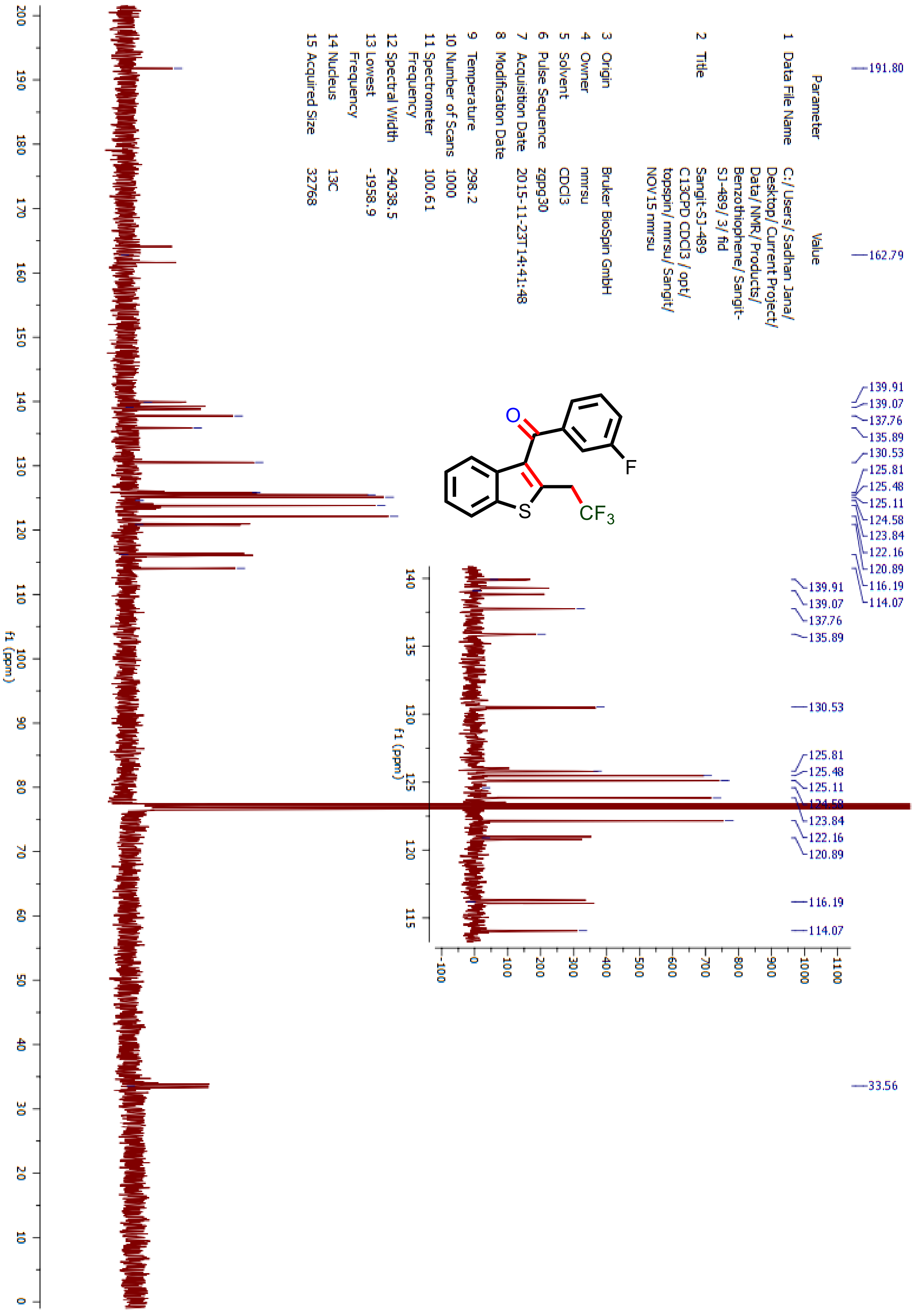

^{19}F NMR of (3-fluorophenyl)(2-(2,2,2-trifluoroethyl)benzo[*b*]thiophen-3-yl)methanone (4d)

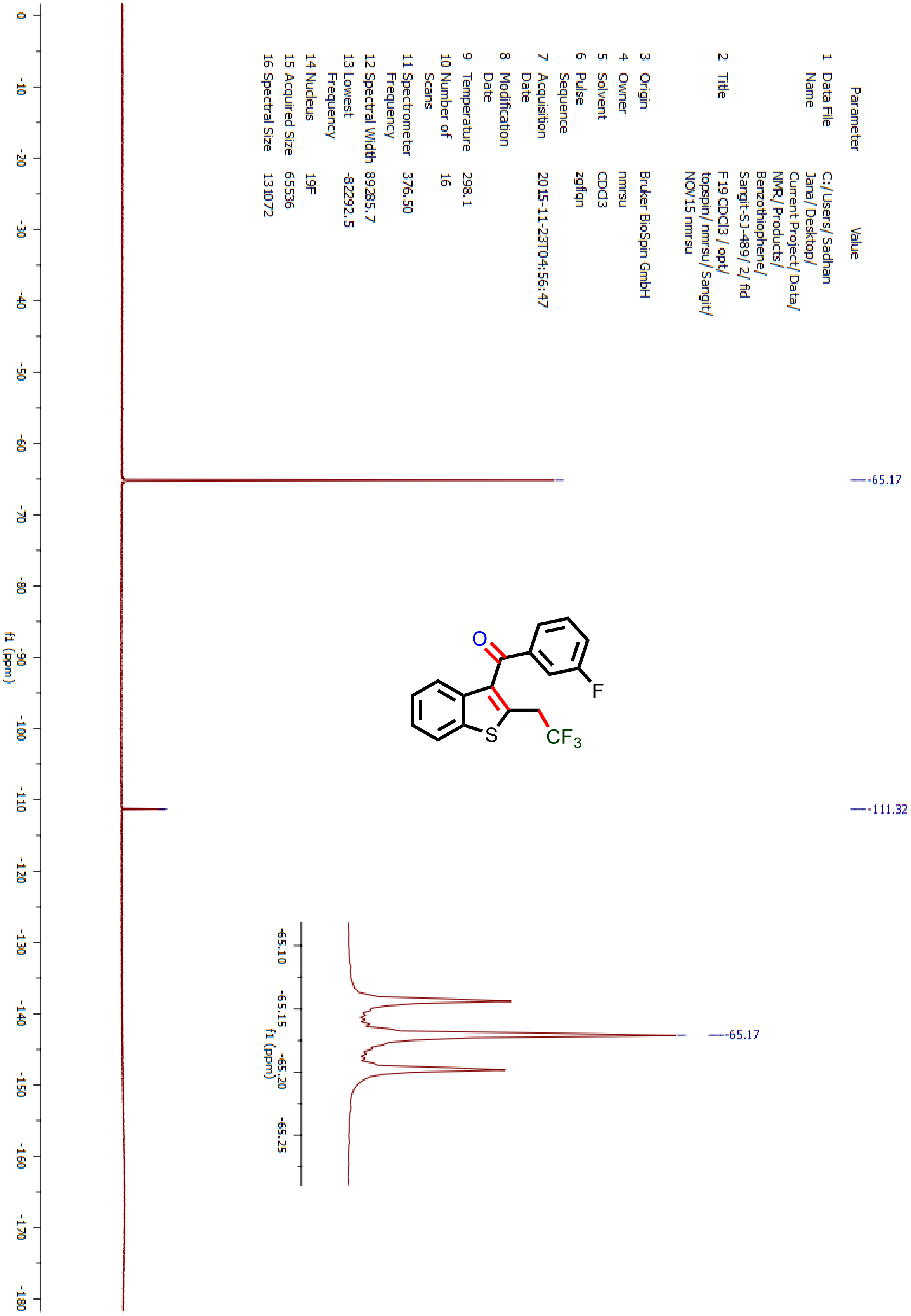

HRMS of (3-fluorophenyl)(2-(2,2,2-trifluoroethyl)benzo[*b*]thiophen-3-yl)methanone (4d)

Display Report

Analysis Info

Analysis Name	D:\Data\user data\2015\NOV-2015\27-NOV-2015\Dr.S.Kumar-SJ-489_1-B,5_01_4359.d	Acquisition Date	11/27/2015 4:53:44 PM
Method	HRLCMS-20 Sept.m	Operator	RUCHI
Sample Name	Dr.S.Kumar-SJ-489	Instrument	micrOTOF-Q II 10330
Comment			

Acquisition Parameter

Source Type	ESI	Ion Polarity	Positive	Set Nebulizer	1.2 Bar
Focus	Active	Set Capillary	4500 V	Set Dry Heater	200 °C
Scan Begin	50 m/z	Set End Plate Offset	-500 V	Set Dry Gas	7.0 l/min
Scan End	3000 m/z	Set Collision Cell RF	130.0 Vpp	Set Divert Valve	Waste

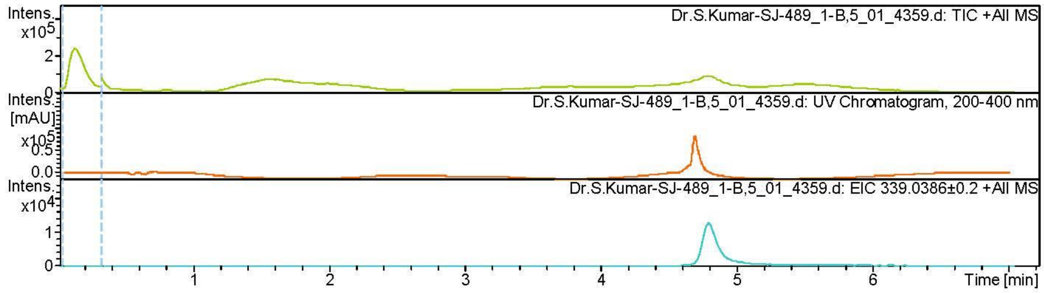

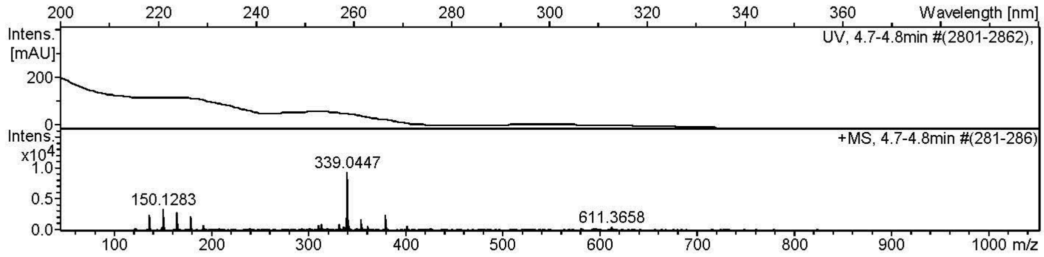

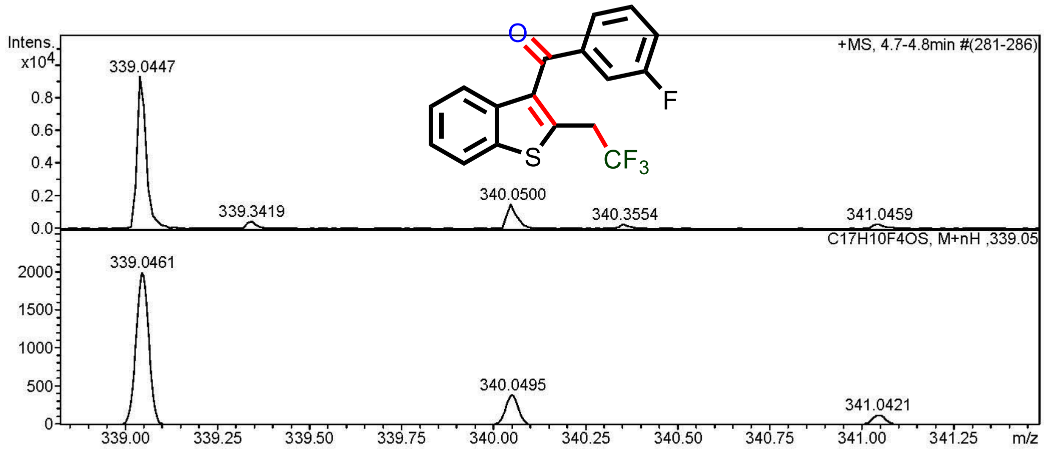

^{1}H NMR of (3-Methoxyphenyl)(2-(2,2,2-trifluoroethyl)benzo[*b*]thiophen-3-yl)methanone (4e)

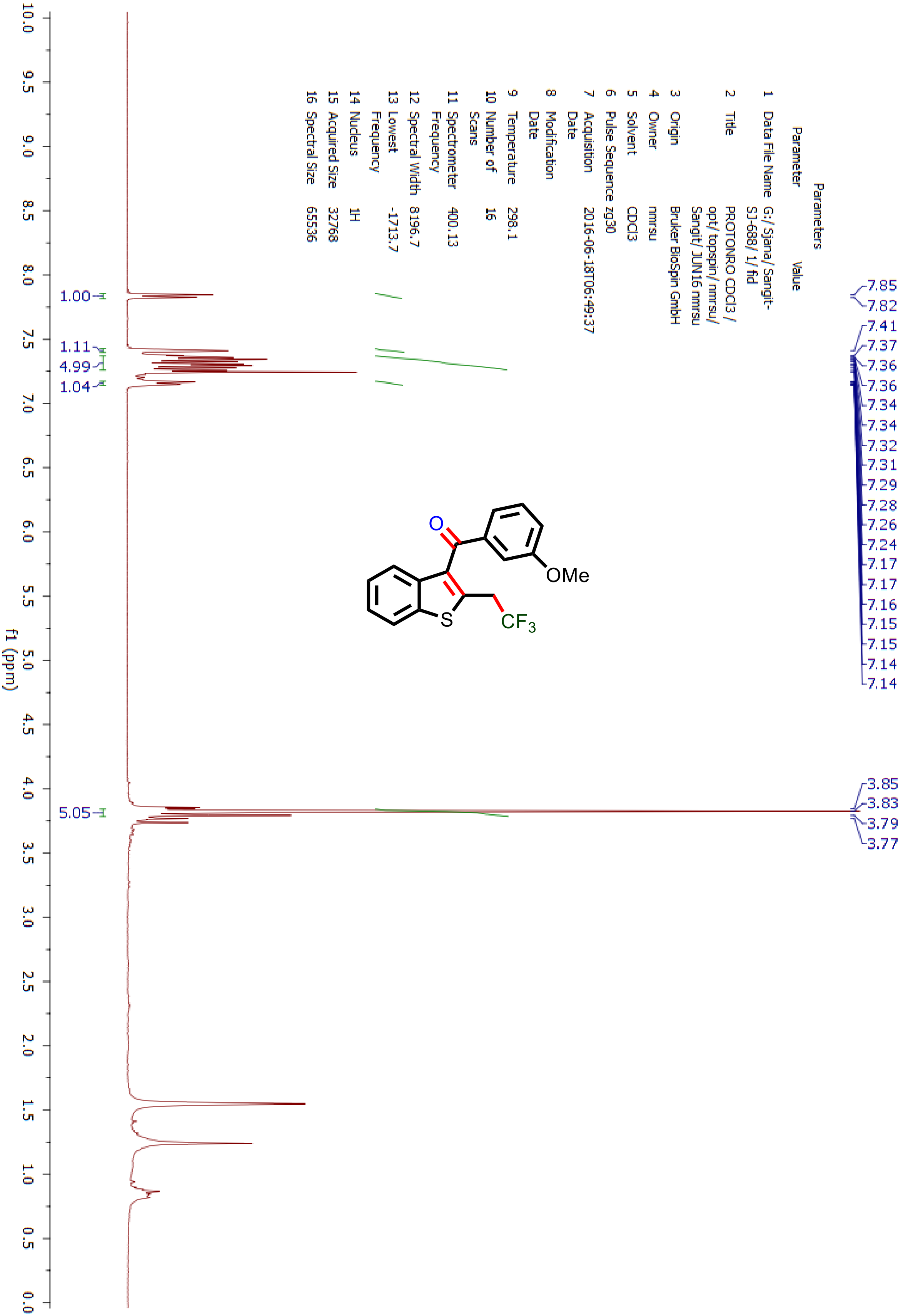

^{13}C NMR of (3-Methoxyphenyl)(2-(2,2,2-trifluoroethyl)benzo[*b*]thiophen-3-yl)methanone (4e)

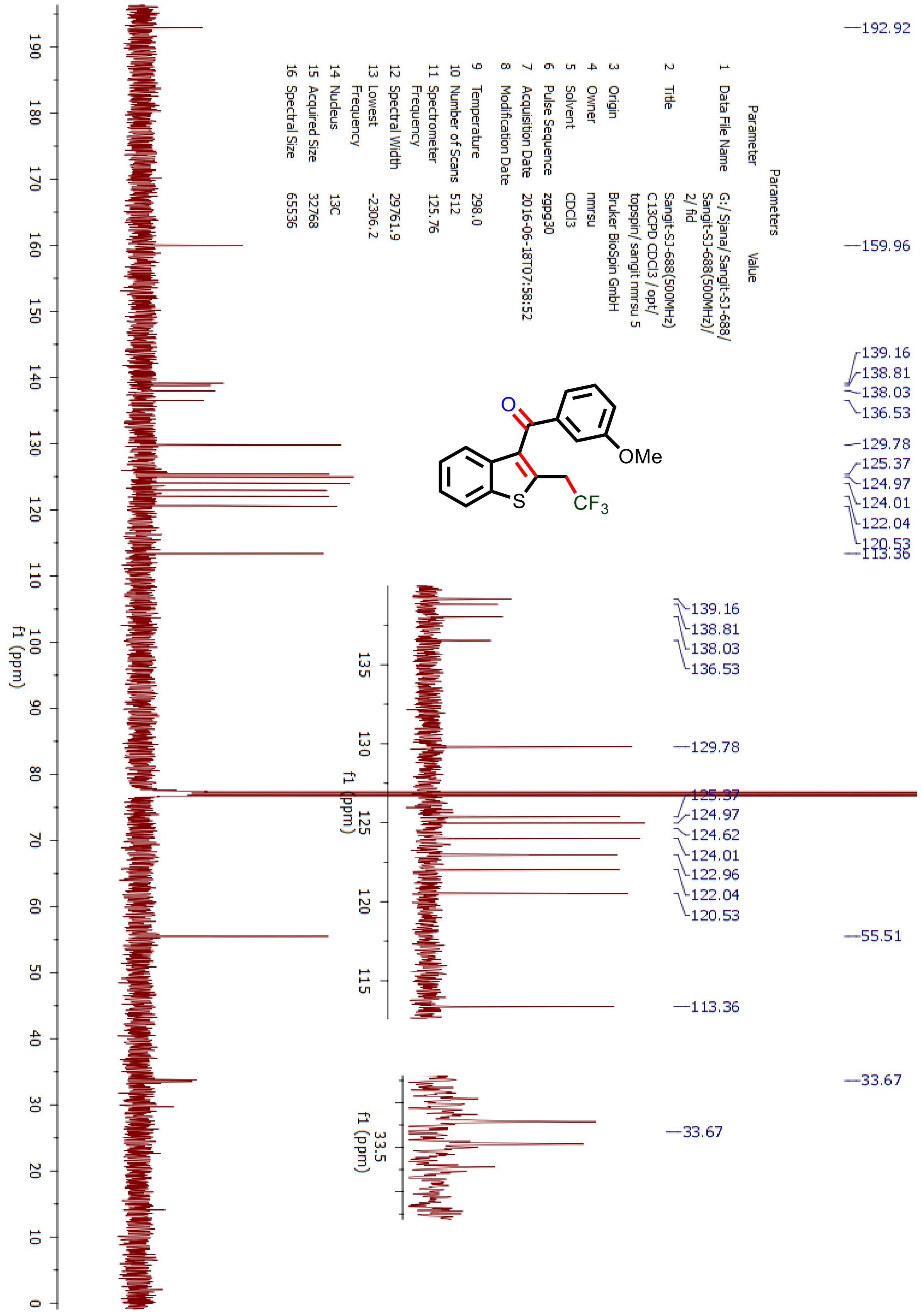

^{19}F NMR of (3-Methoxyphenyl)(2-(2,2,2-trifluoroethyl)benzo[*b*]thiophen-3-yl)methanone (4e)

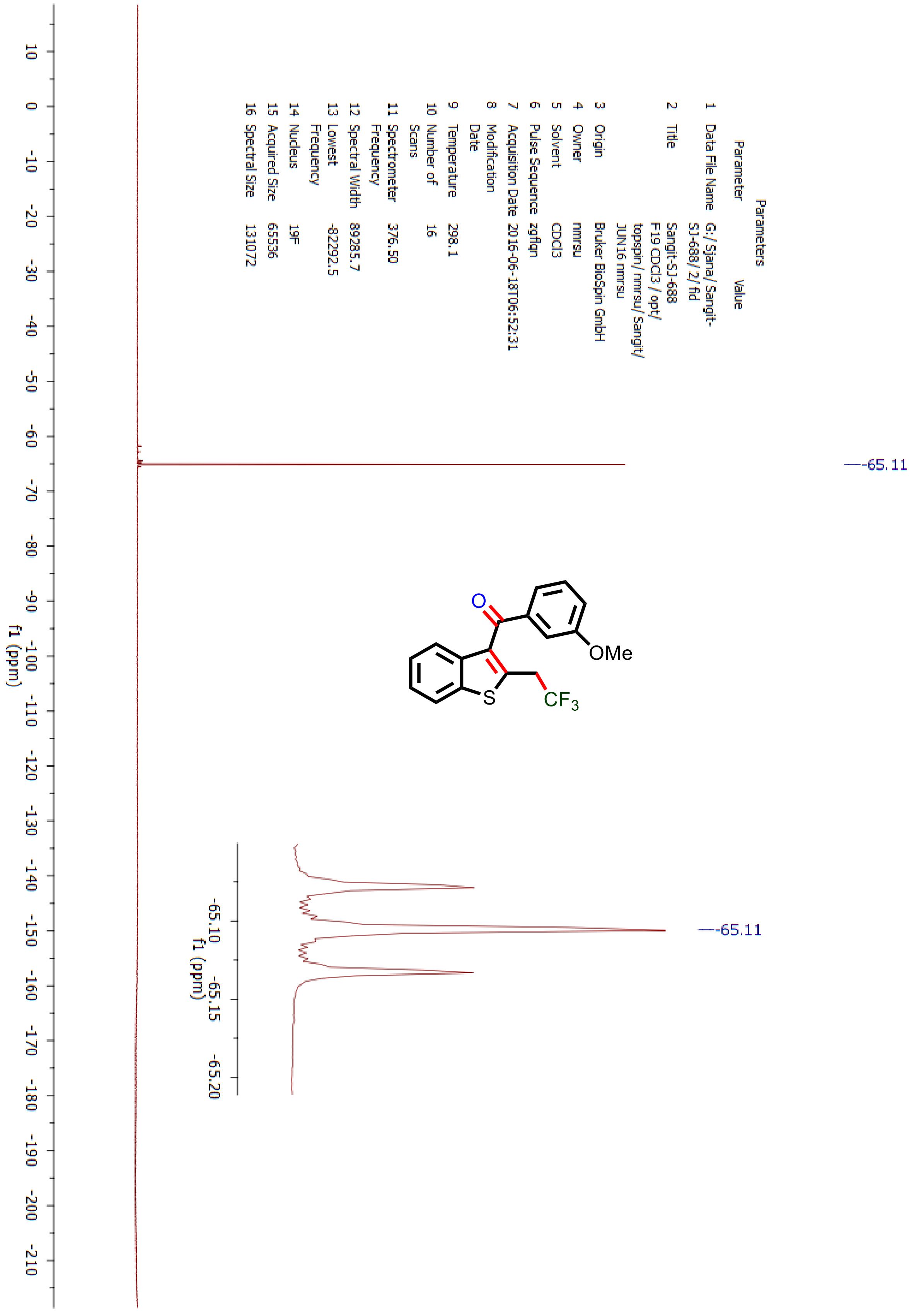

HRMS of (3-Methoxyphenyl)(2-(2,2,2-trifluoroethyl)benzo[*b*]thiophen-3-yl)methanone (4e)

Display Report

Analysis Info

Analysis Name	D:\Data\user data\2016\SEPT-2016\12-09-2016\Dr.S.Kumar-SJ-688_1-A,3_01_7364.d	Acquisition Date	9/12/2016 11:12:27 AM
Method	hrlcms_pos_mid_tunemix.m	Operator	DIMPLE
Sample Name	Dr.S.Kumar-SJ-688	Instrument	micrOTOF-Q II 10330
Comment			

Acquisition Parameter

Source Type	ESI	Ion Polarity	Positive	Set Nebulizer	0.3 Bar
Focus	Active	Set Capillary	4500 V	Set Dry Heater	200 °C
Scan Begin	50 m/z	Set End Plate Offset	-500 V	Set Dry Gas	4.0 l/min
Scan End	3000 m/z	Set Collision Cell RF	450.0 Vpp	Set Divert Valve	Waste

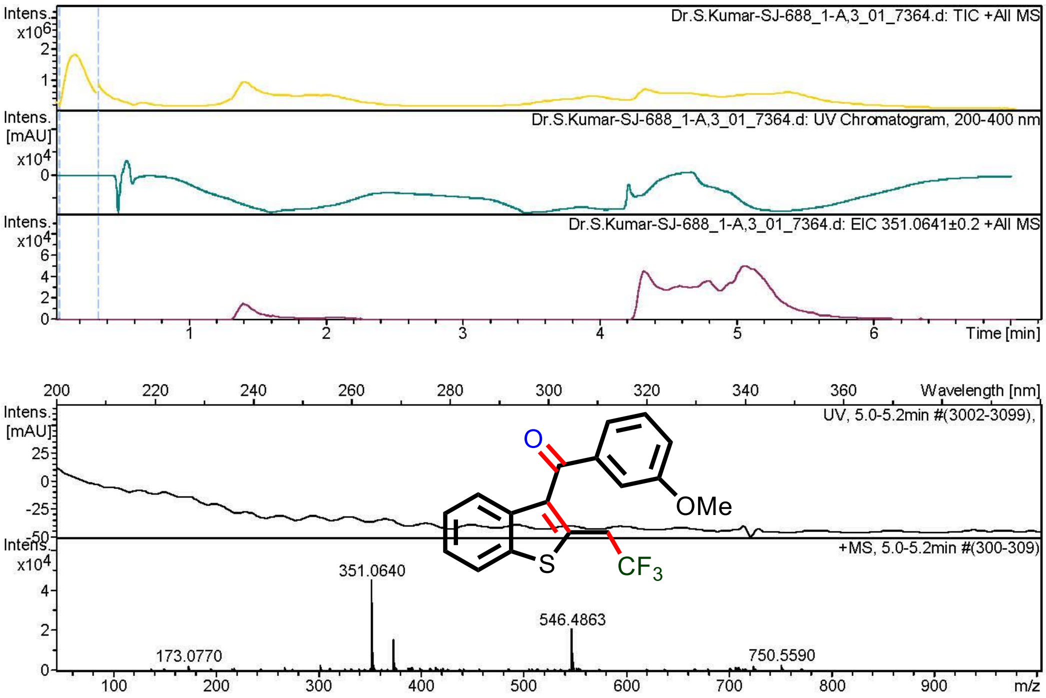

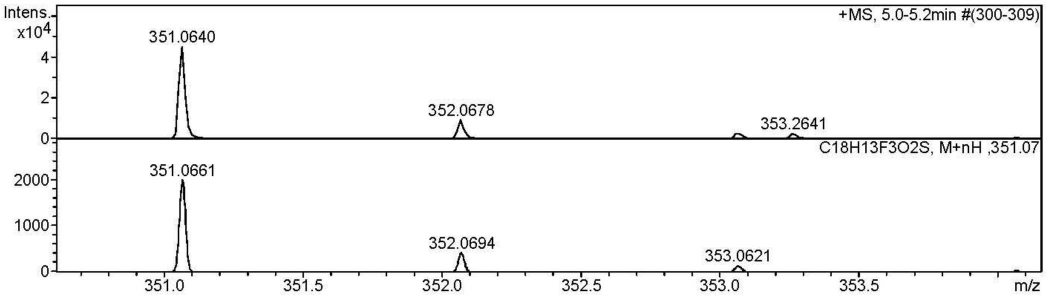

^{1}H NMR of (2-(2,2,2-Trifluoroethyl)benzo[*b*]thiophen-3-yl)(2-(trifluoromethyl)phenyl)methanone (4f)

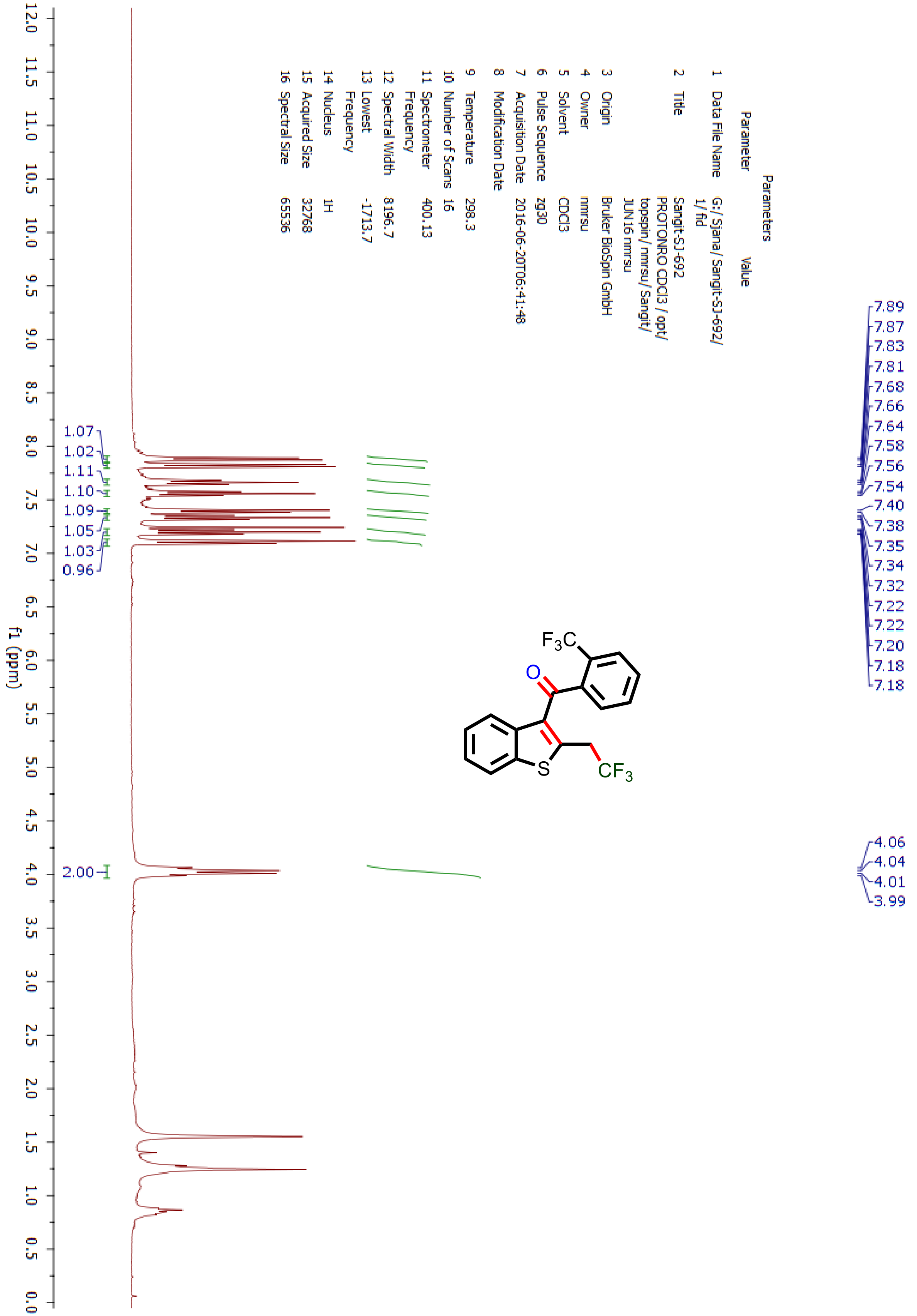

^{13}C NMR of (2-(2,2,2-Trifluoroethyl)benzo[*b*]thiophen-3-yl)(2-(trifluoromethyl)phenyl)methanone (4f)

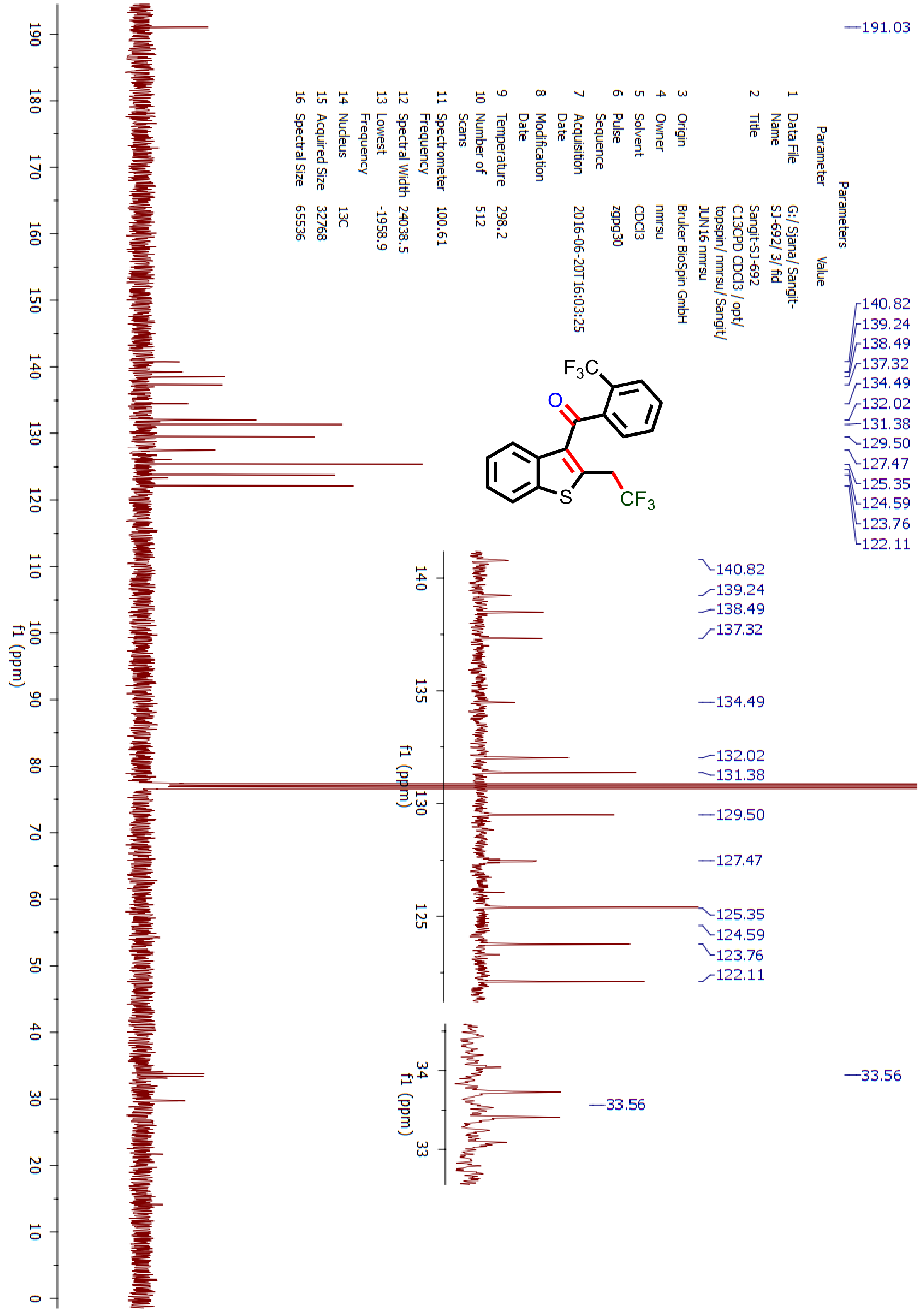

^{19}F NMR of (2-(2,2,2-Trifluoroethyl)benzo[*b*]thiophen-3-yl)(2-(trifluoromethyl)phenyl)methanone (4f)

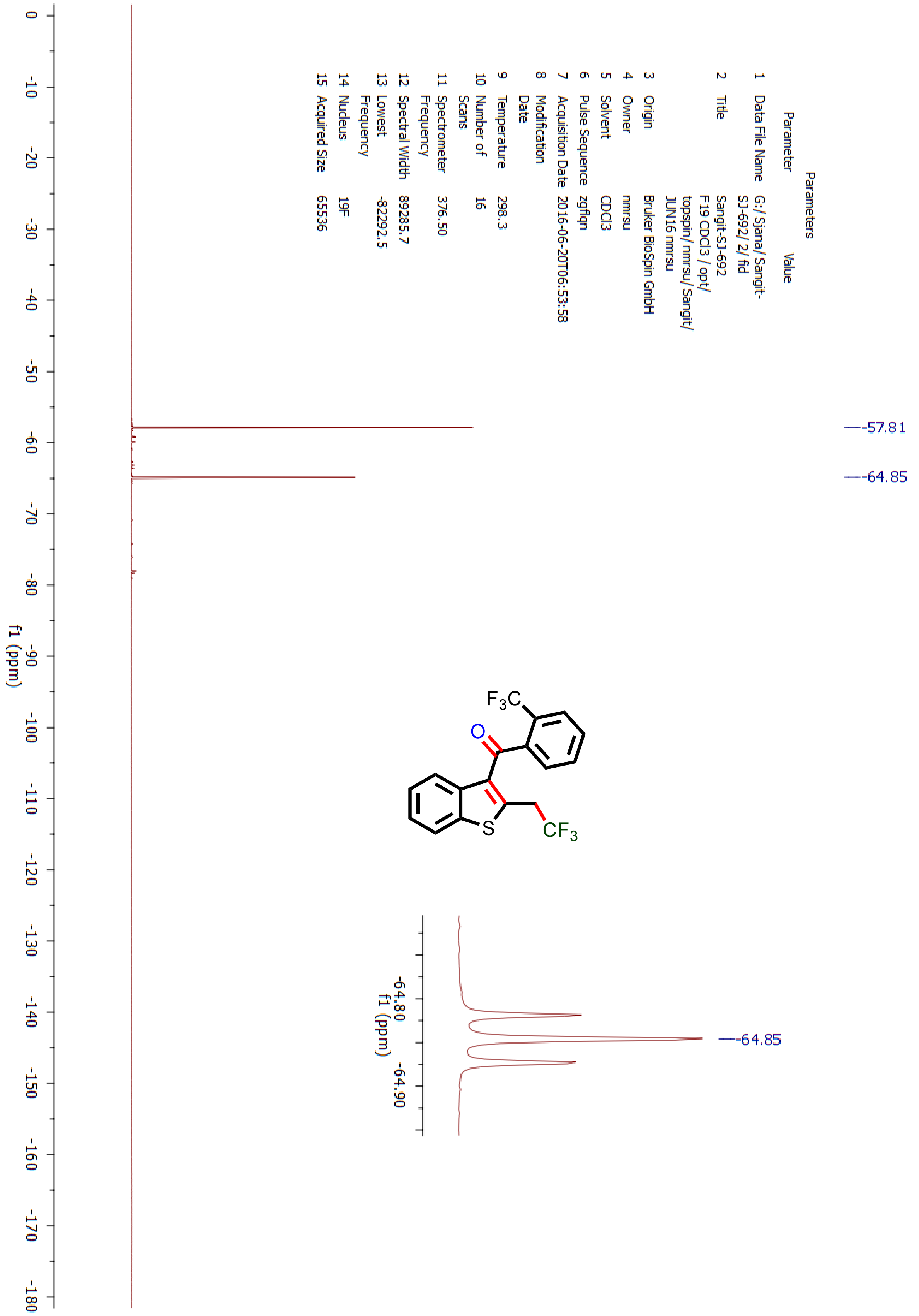

HRMS of (2-(2,2,2-Trifluoroethyl)benzo[*b*]thiophen-3-yl)(2-(trifluoromethyl)phenyl)methanone (4f)

Display Report

Analysis Info

		Acquisition Date	9/12/2016 11:04:17 AM
Analysis Name	D:\Data\user data\2016\SEPT-2016\12-09-2016\Dr.S.Kumar-SJ-692_1-A,2_01_7363.d		
Method	hrlcms_pos_mid_tunemix.m	Operator	DIMPLE
Sample Name	Dr.S.Kumar-SJ-692	Instrument	micrOTOF-Q II 10330
Comment			

Acquisition Parameter

Source Type	ESI	Ion Polarity	Positive	Set Nebulizer	0.3 Bar
Focus	Active	Set Capillary	4500 V	Set Dry Heater	200 °C
Scan Begin	50 m/z	Set End Plate Offset	-500 V	Set Dry Gas	4.0 l/min
Scan End	3000 m/z	Set Collision Cell RF	450.0 Vpp	Set Divert Valve	Waste

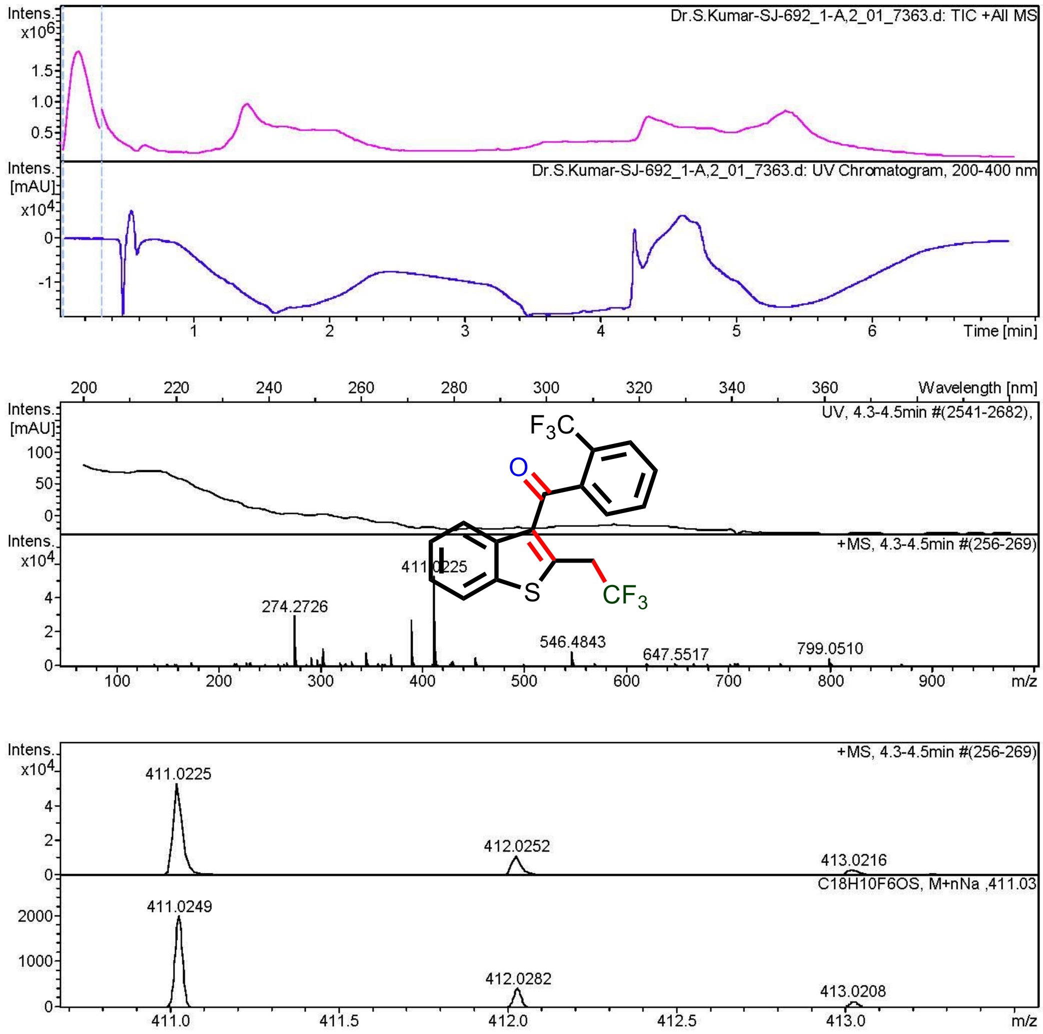

Bruker Compass DataAnalysis 4.0   printed: 9/12/2016 12:53:25 PM   Page 1 of 1

^{1}H NMR of *o*-Tolyl(2-(2,2,2-trifluoroethyl)benzo[*b*]thiophen-3-yl)methanone (4g)

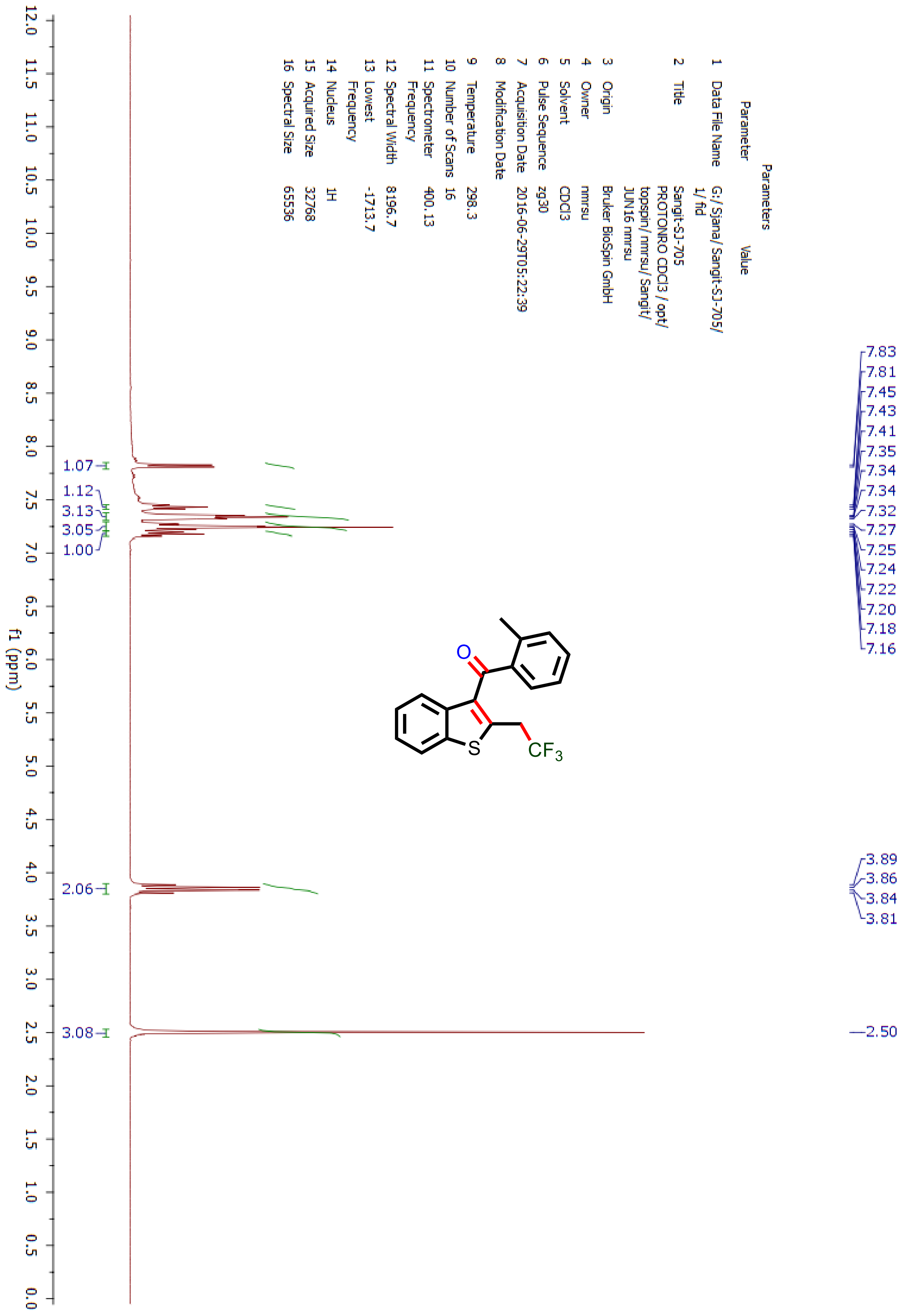

^{13}C NMR of *o*-Tolyl(2-(2,2,2-trifluoroethyl)benzo[*b*]thiophen-3-yl)methanone (4g)

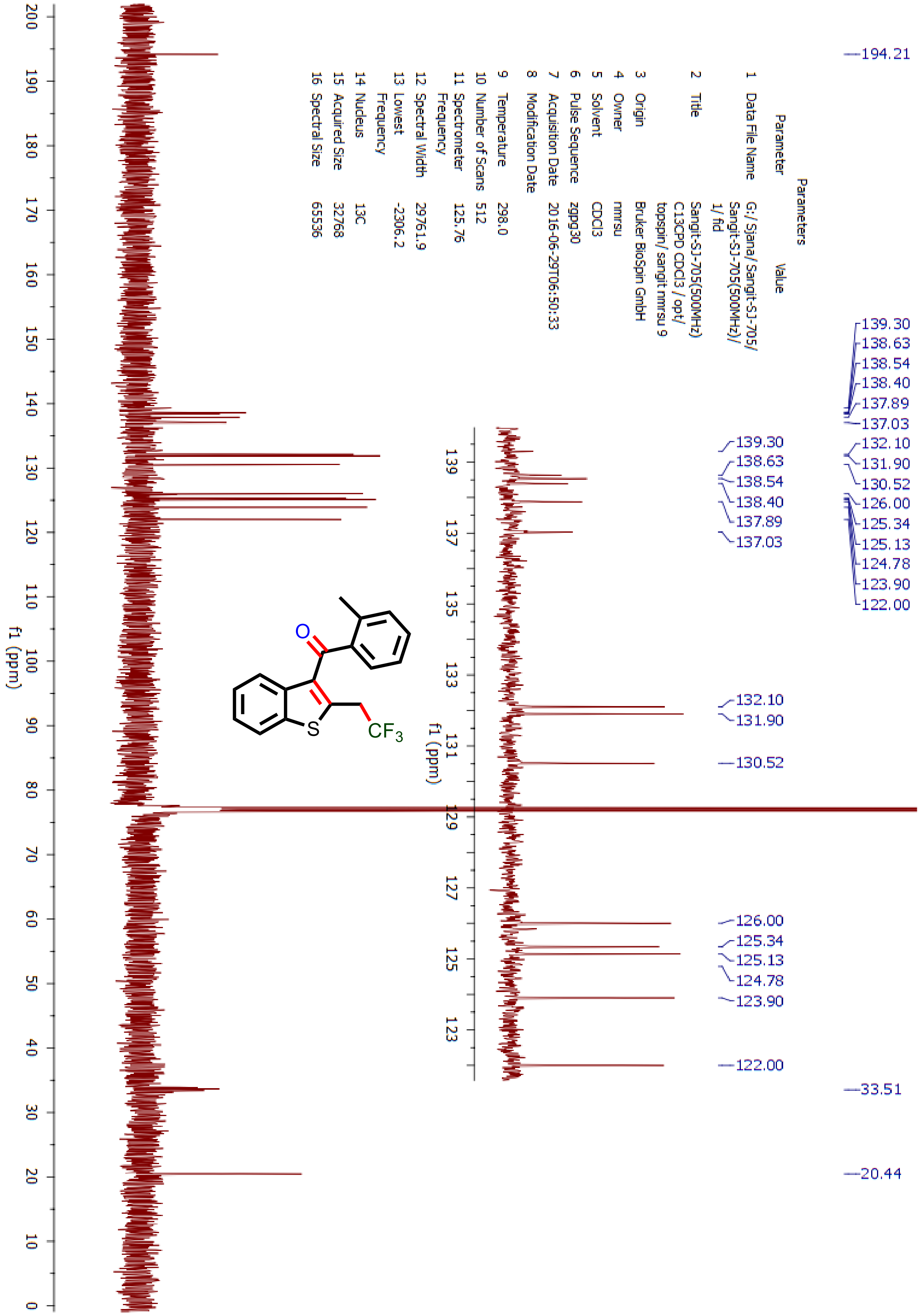

^{19}F NMR of *o*-Tolyl(2-(2,2,2-trifluoroethyl)benzo[*b*]thiophen-3-yl)methanone (4g)

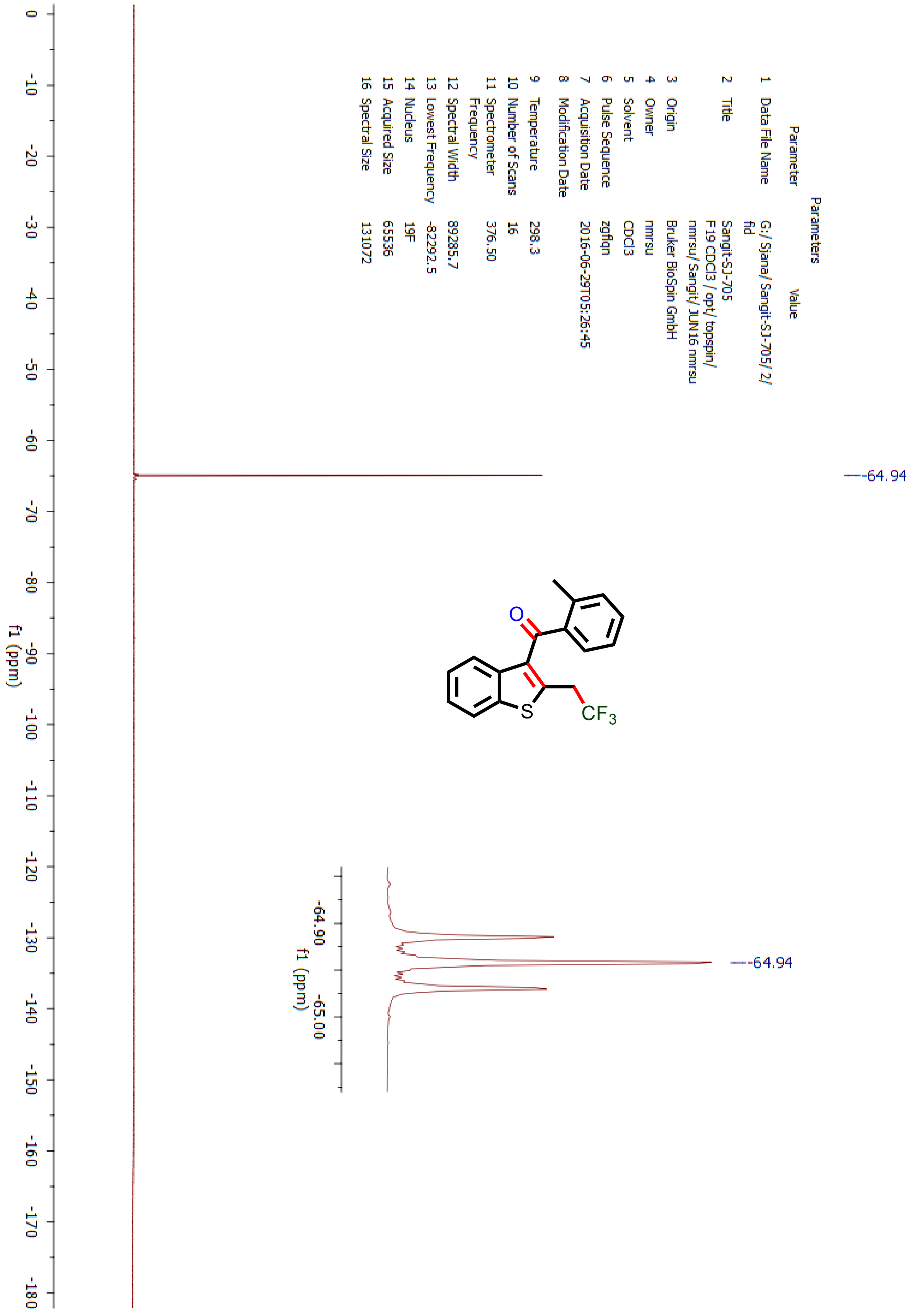

HRMS of *o*-Tolyl(2-(2,2,2-trifluoroethyl)benzo[*b*]thiophen-3-yl)methanone (4g)

Display Report

Analysis Info

Analysis Name	D:\Data\user data\2016\SEPT-2016\09-sep-2016\Dr S. Kumar-SJ-705_1-A,5_01_7343.d	Acquisition Date	9/9/2016 1:10:33 PM
Method	hrlcms_pos_low_tunemix.m	Operator	DIMPLE
Sample Name	Dr S. Kumar-SJ-705	Instrument	micrOTOF-Q II 10330
Comment			

Acquisition Parameter

Source Type	ESI	Ion Polarity	Positive	Set Nebulizer	1.0 Bar
Focus	Active	Set Capillary	4500 V	Set Dry Heater	250 °C
Scan Begin	50 m/z	Set End Plate Offset	-500 V	Set Dry Gas	7.0 l/min
Scan End	3000 m/z	Set Collision Cell RF	130.0 Vpp	Set Divert Valve	Waste

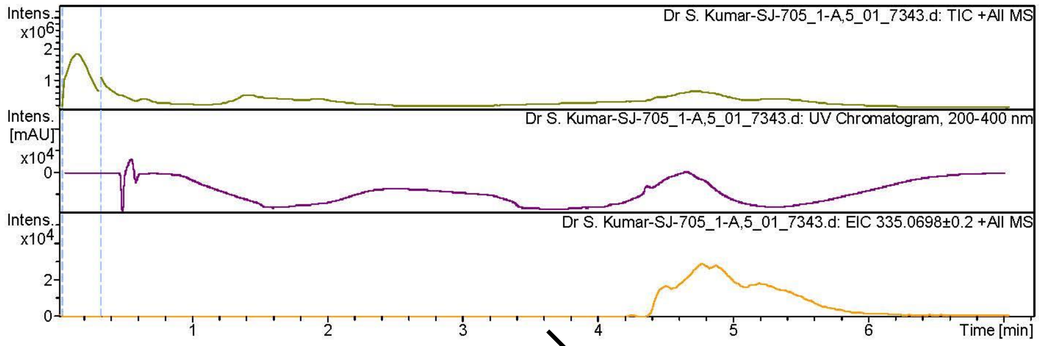

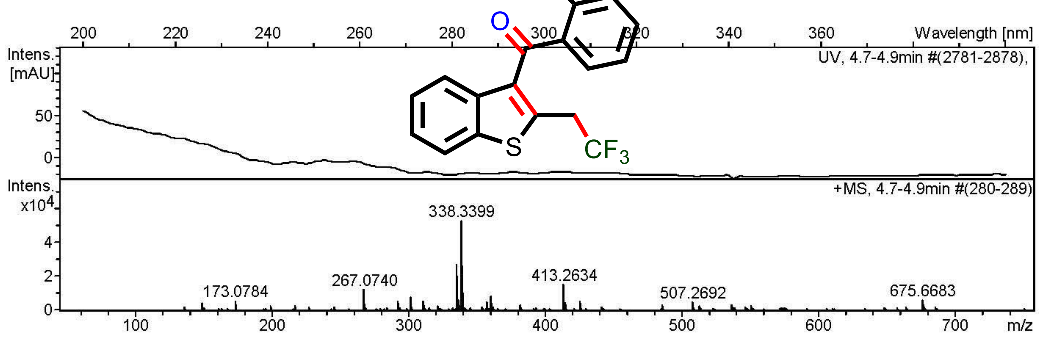

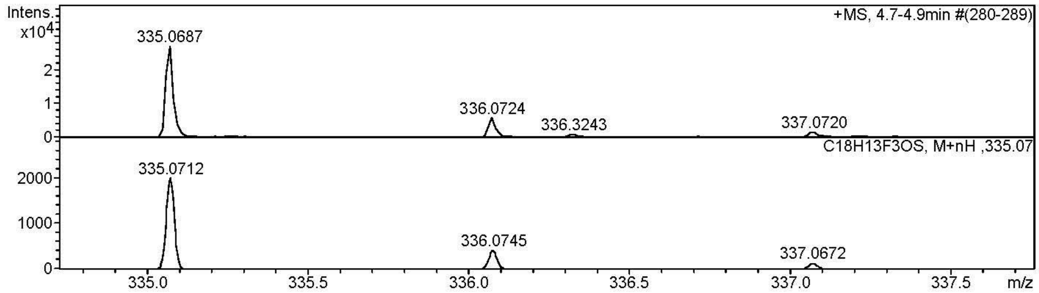

Bruker Compass DataAnalysis 4.0 printed: 9/9/2016 4:13:37 PM Page 1 of 1

^{1}H NMR of Benzo[*d*][1,3]dioxol-5-yl(2-(2,2,2-trifluoroethyl)benzo[*b*]thiophen-3-yl)methanone (4h)

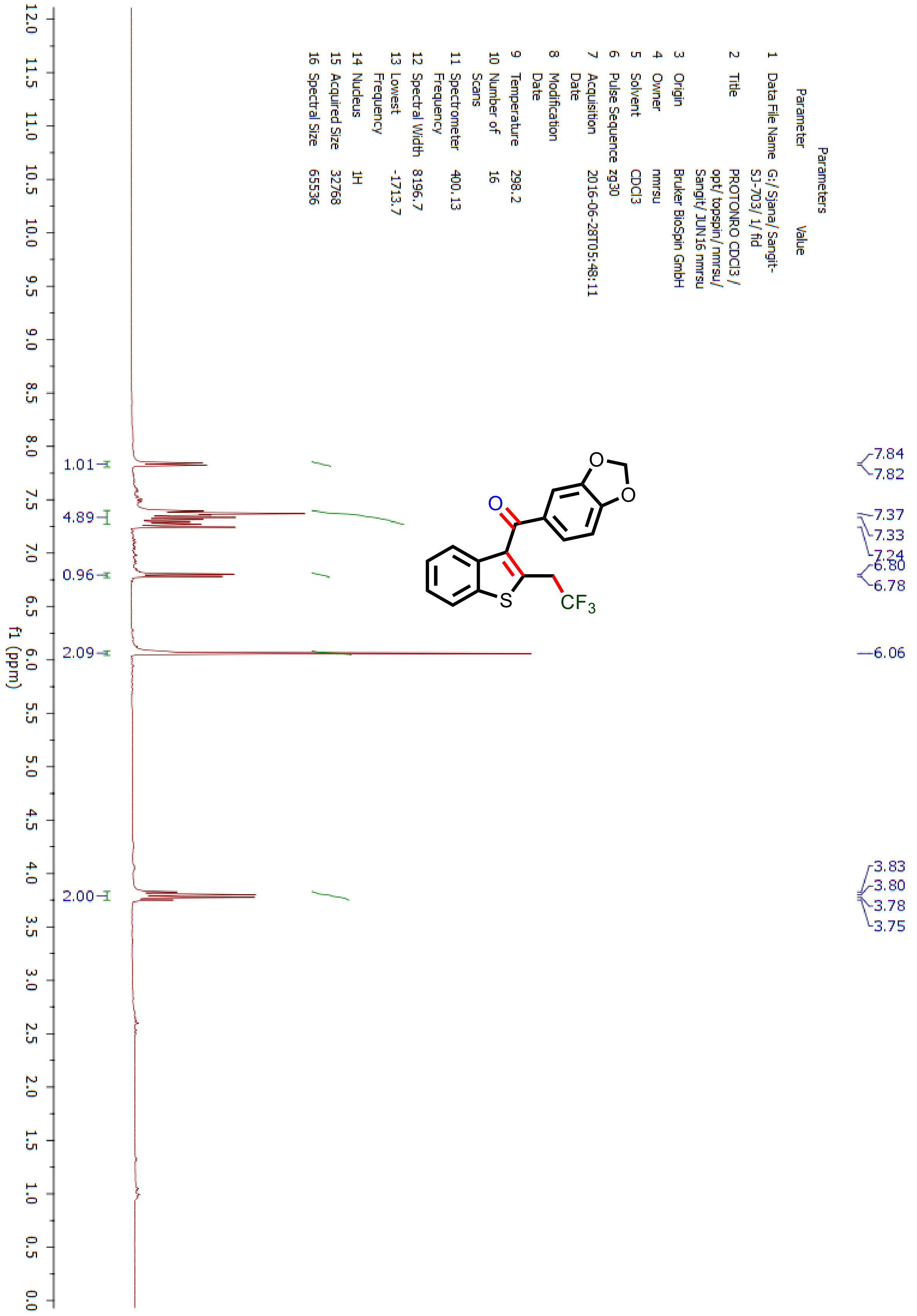

^{13}C NMR of Benzo[*d*][1,3]dioxol-5-yl(2-(2,2,2-trifluoroethyl)benzo[*b*]thiophen-3-yl)methanone (4h)

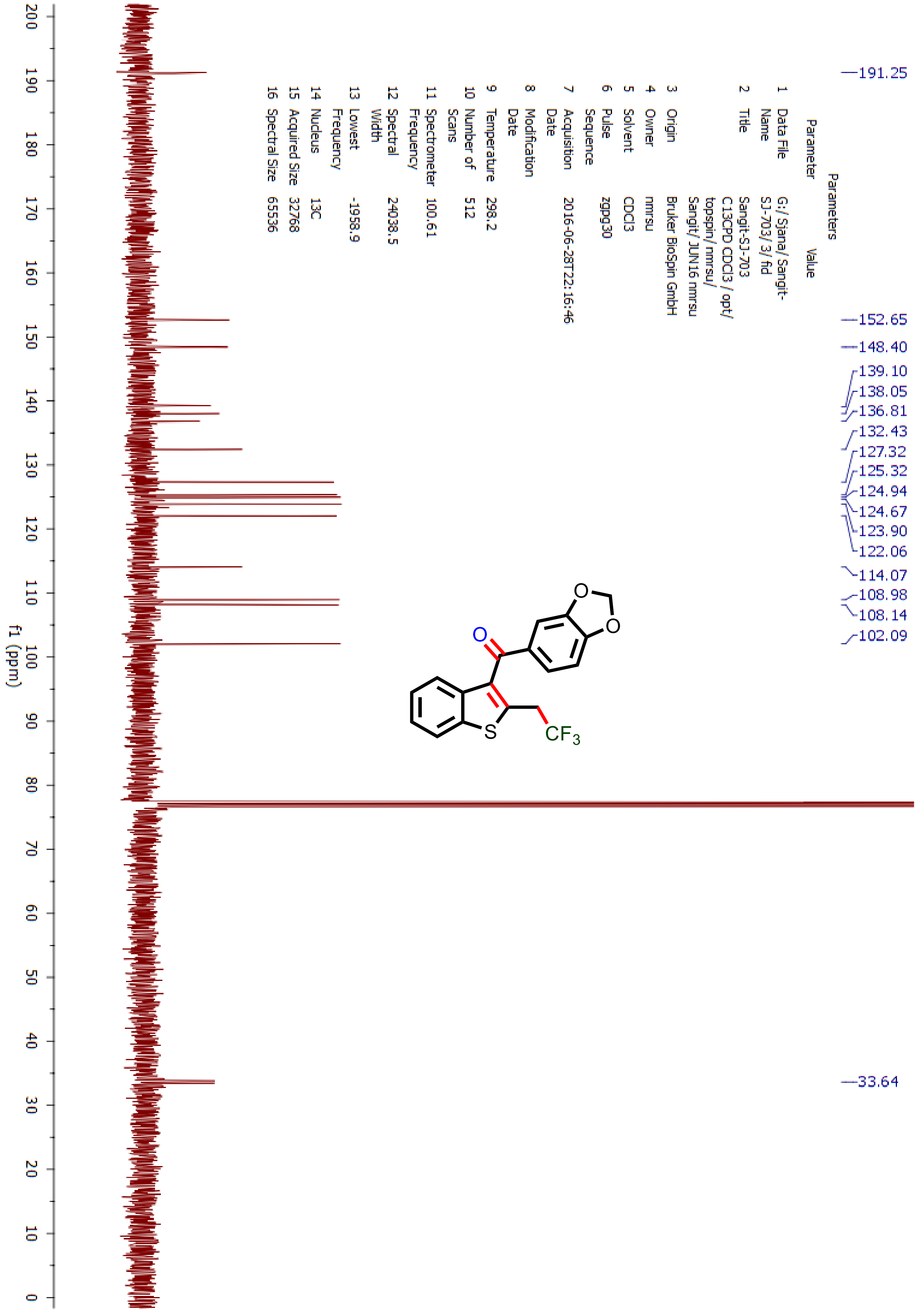

^{19}F NMR of Benzo[*d*][1,3]dioxol-5-yl(2-(2,2,2-trifluoroethyl)benzo[*b*]thiophen-3-yl)methanone (4h)

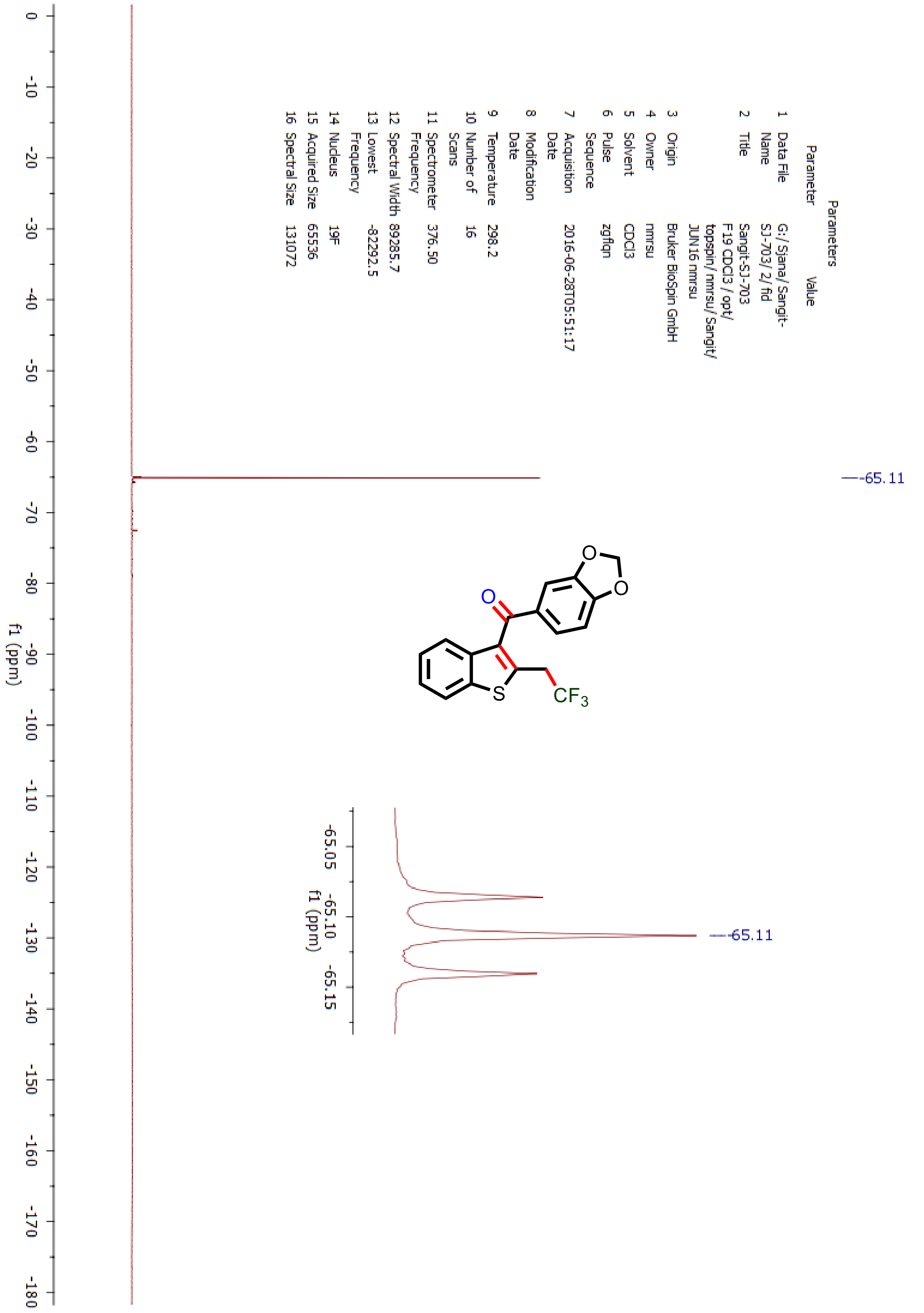

HRMS of Benzo[*d*][1,3]dioxol-5-yl(2-(2,2,2-trifluoroethyl)benzo[*b*]thiophen-3-yl)methanone (4h)

Display Report

Analysis Info

		Acquisition Date	9/9/2016 1:02:24 PM
Analysis Name	D:\Data\user data\2016\SEPT-2016\09-sep-2016\Dr S. Kumar-SJ-703_1-A,4_01_7342.d		
Method	hrlcms_pos_low_tunemix.m	Operator	DIMPLE
Sample Name	Dr S. Kumar-SJ-703	Instrument	micrOTOF-Q II 10330
Comment			

Acquisition Parameter

Source Type	ESI	Ion Polarity	Positive	Set Nebulizer	1.0 Bar
Focus	Active	Set Capillary	4500 V	Set Dry Heater	250 °C
Scan Begin	50 m/z	Set End Plate Offset	-500 V	Set Dry Gas	7.0 l/min
Scan End	3000 m/z	Set Collision Cell RF	130.0 Vpp	Set Divert Valve	Waste

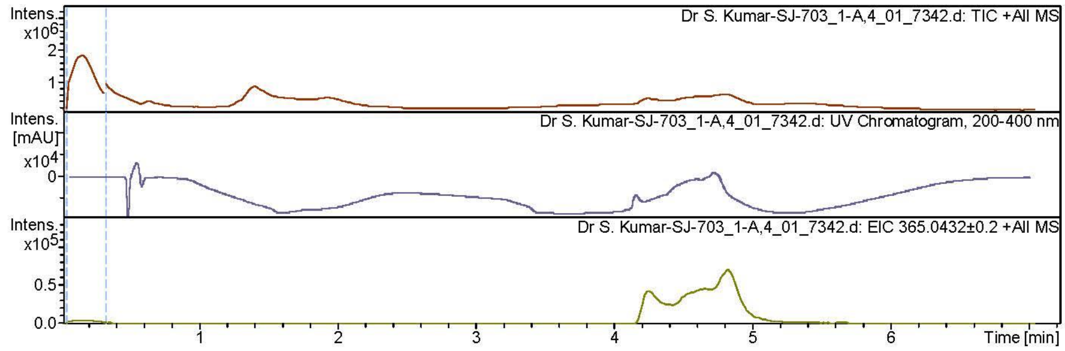

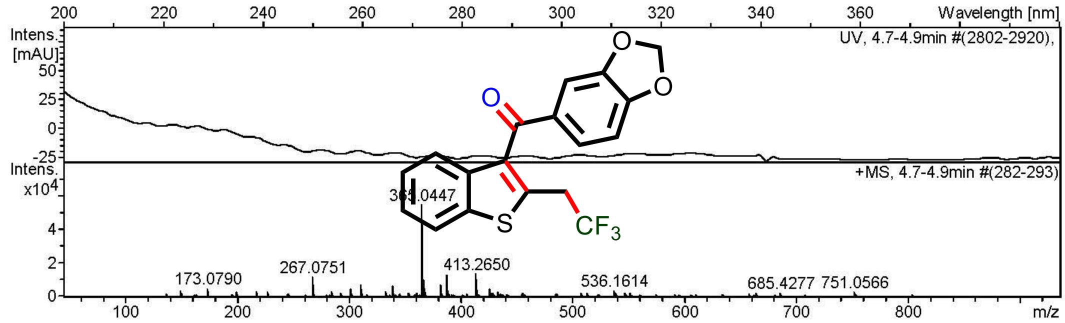

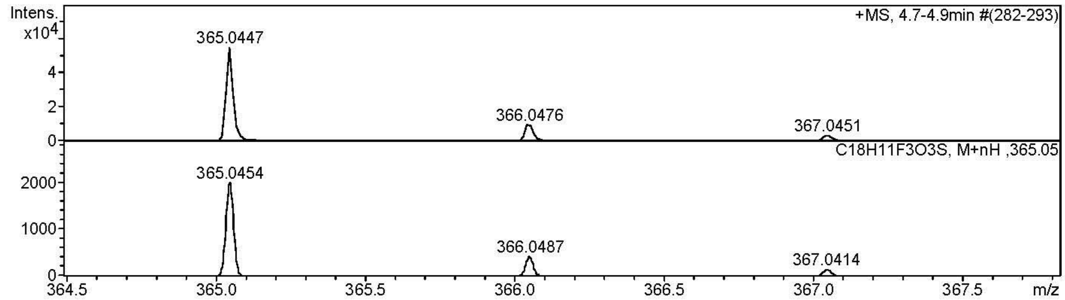

Bruker Compass DataAnalysis 4.0 printed: 9/9/2016 4:12:17 PM Page 1 of 1

^{1}H NMR of (2-(2,2,2-Trifluoroethyl)benzo[*b*]thiophen-3-yl)(3,4,5-trimethoxyphenyl) methanone (4i)

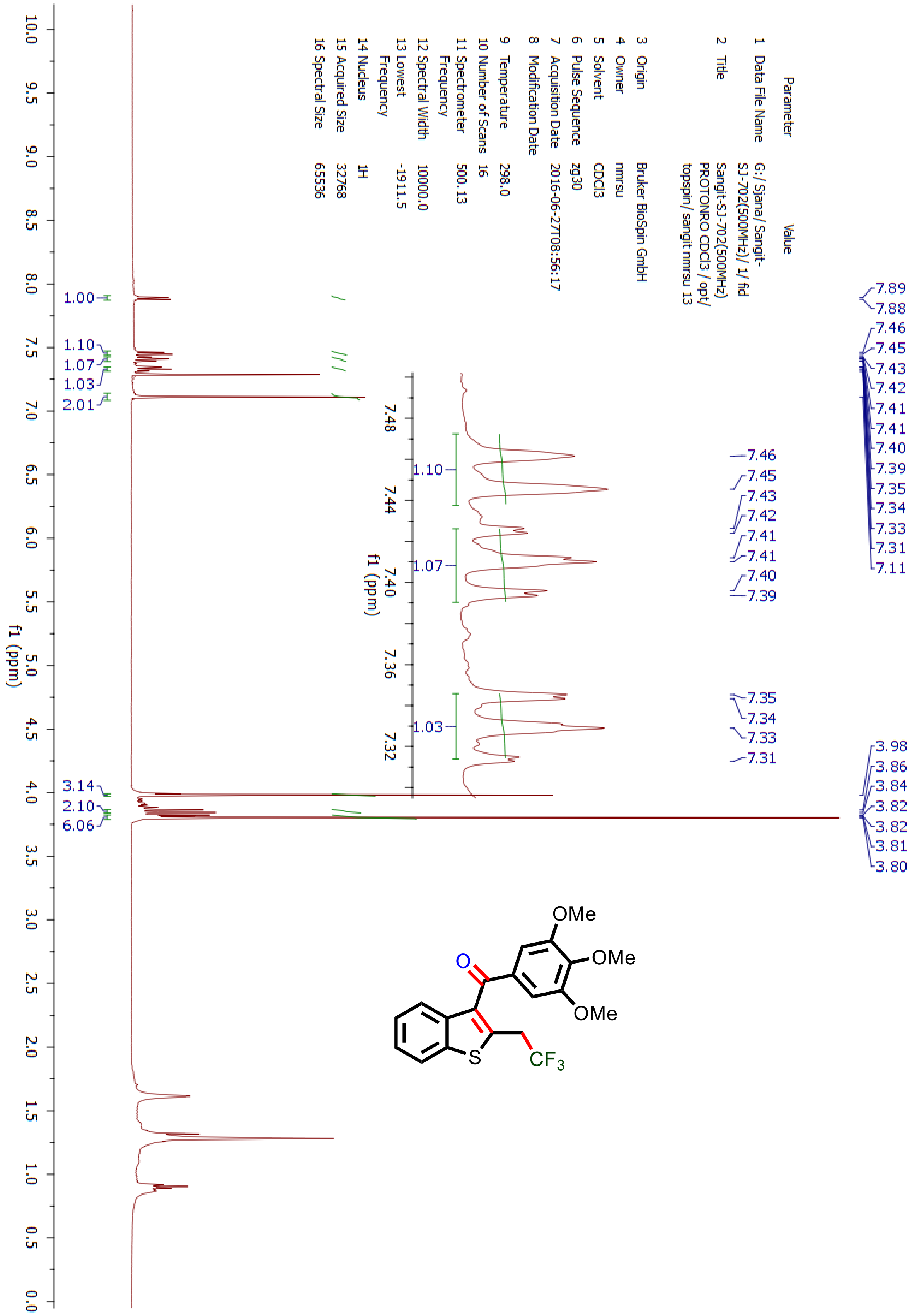

^{13}C NMR of (2-(2,2,2-Trifluoroethyl)benzo[*b*]thiophen-3-yl)(3,4,5-trimethoxyphenyl) methanone (4i)

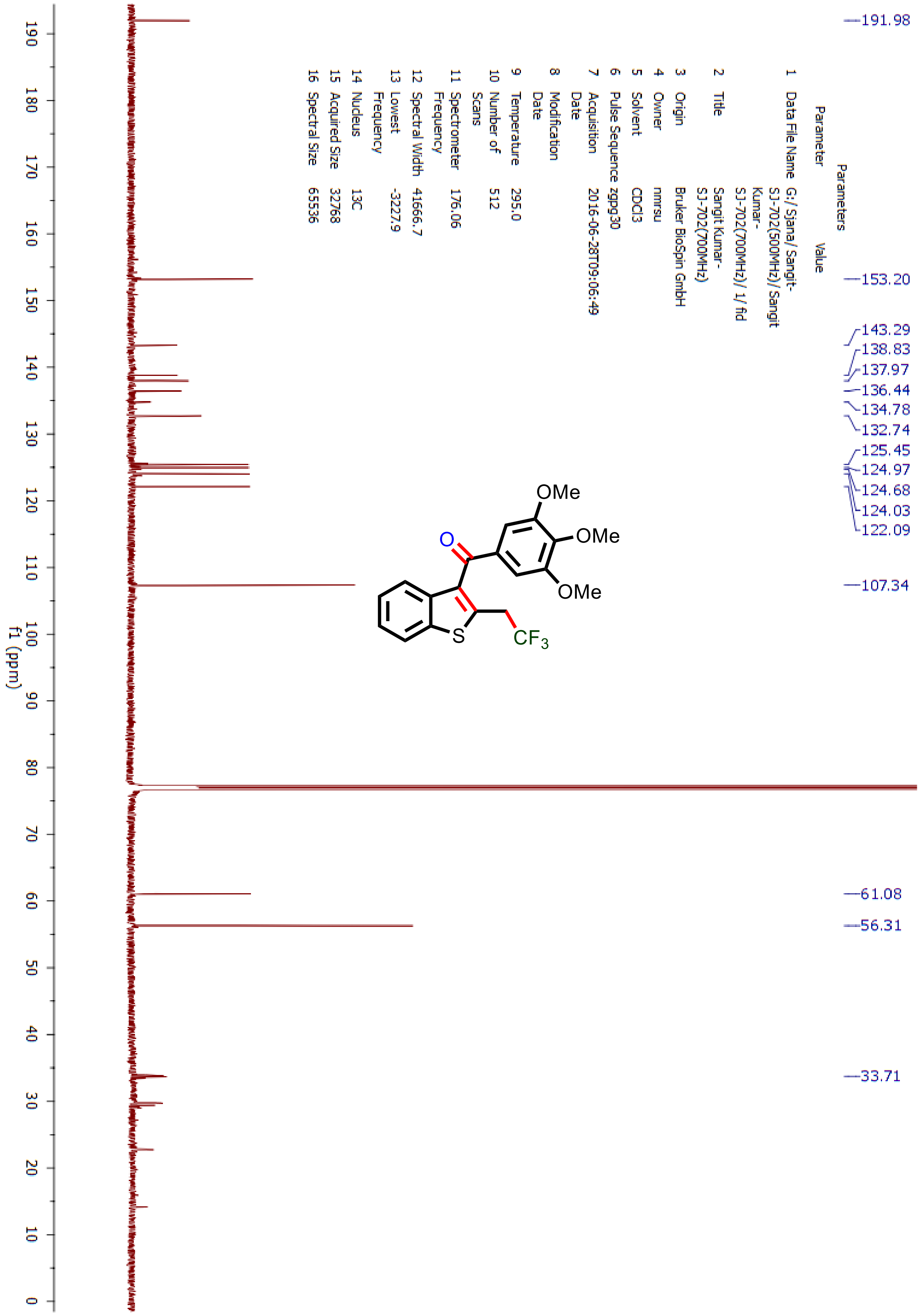

^{19}F NMR of (2-(2,2,2-Trifluoroethyl)benzo[*b*]thiophen-3-yl)(3,4,5-trimethoxyphenyl) methanone (4i)

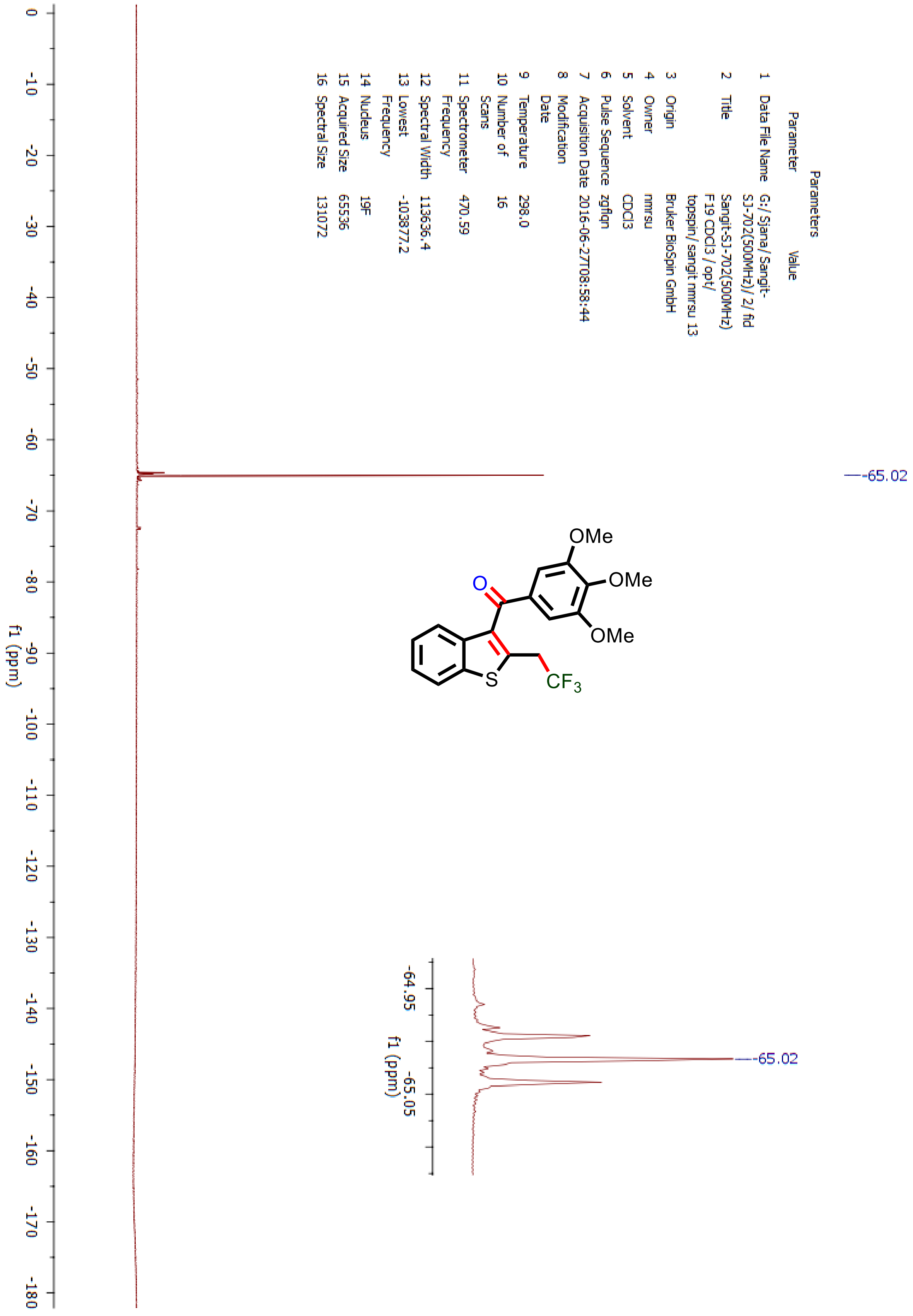

HRMS of (2-(2,2,2-Trifluoroethyl)benzo[*b*]thiophen-3-yl)(3,4,5-trimethoxyphenyl) methanone (4i)

Display Report

Analysis Info

		Acquisition Date	9/9/2016 12:46:02 PM
Analysis Name	D:\Data\user data\2016\SEPT-2016\09-sep-2016\Dr S. Kumar-SJ-702_1-A,3_01_7340.d		
Method	hrlcms_pos_low_tunemix.m	Operator	DIMPLE
Sample Name	Dr S. Kumar-SJ-702	Instrument	micrOTOF-Q II 10330
Comment			

Acquisition Parameter

Source Type	ESI	Ion Polarity	Positive	Set Nebulizer	1.0 Bar
Focus	Active	Set Capillary	4500 V	Set Dry Heater	250 °C
Scan Begin	50 m/z	Set End Plate Offset	-500 V	Set Dry Gas	7.0 l/min
Scan End	3000 m/z	Set Collision Cell RF	130.0 Vpp	Set Divert Valve	Waste

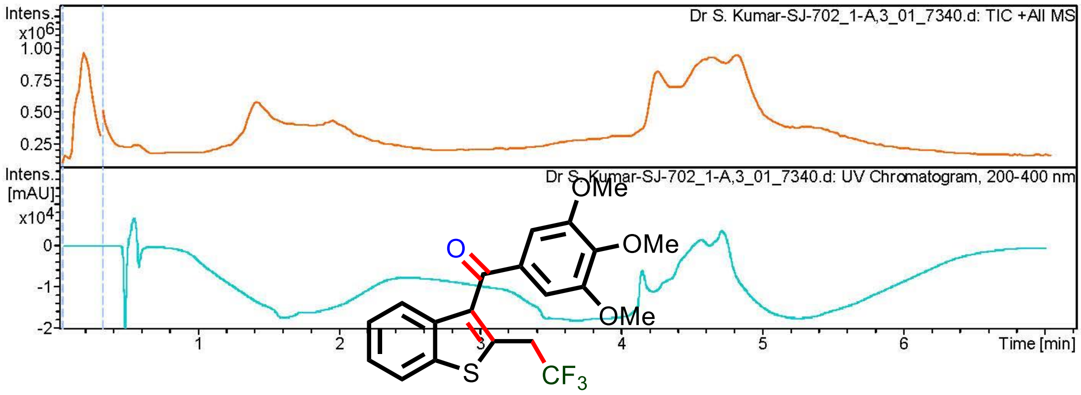

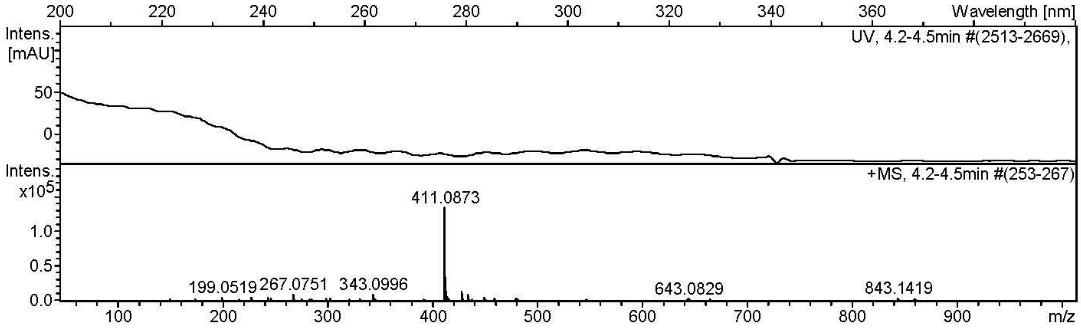

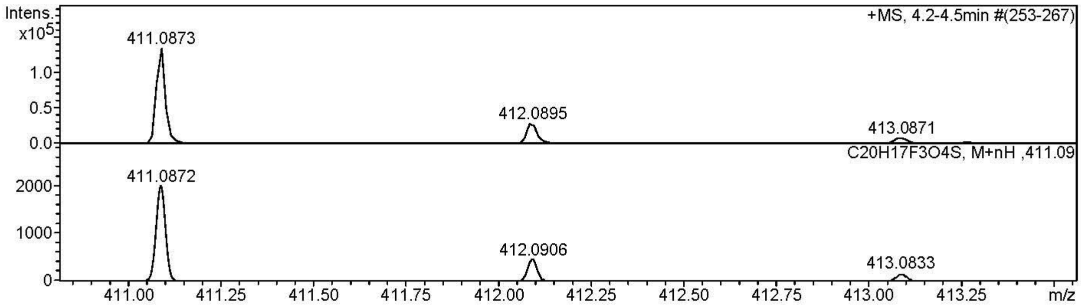

^{1}H NMR of Thiophen-3-yl(2-(2,2,2-trifluoroethyl)benzo[*b*]thiophen-3-yl)methanone (4j)

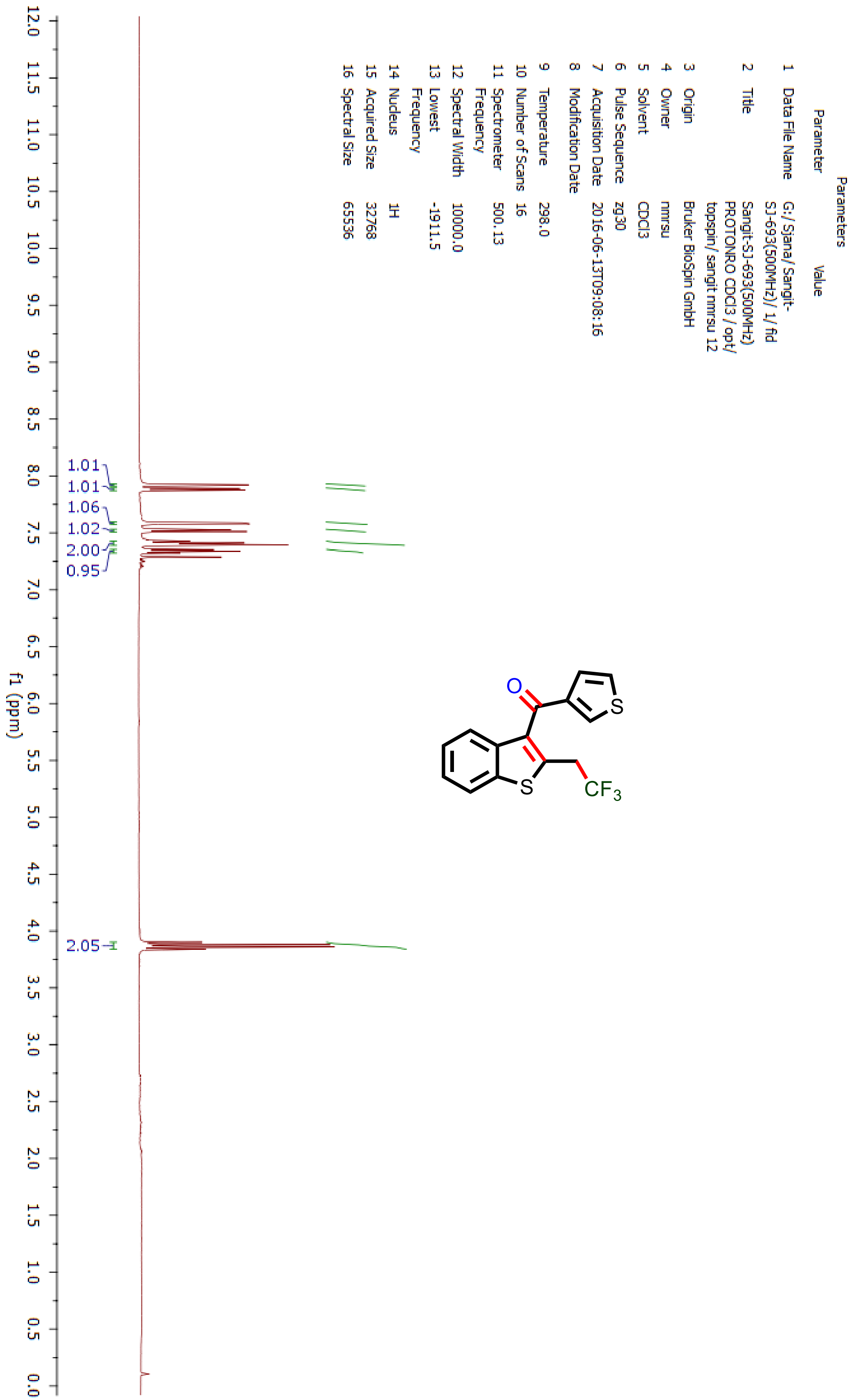

^{13}C NMR of Thiophen-3-yl(2-(2,2,2-trifluoroethyl)benzo[*b*]thiophen-3-yl)methanone (4j)

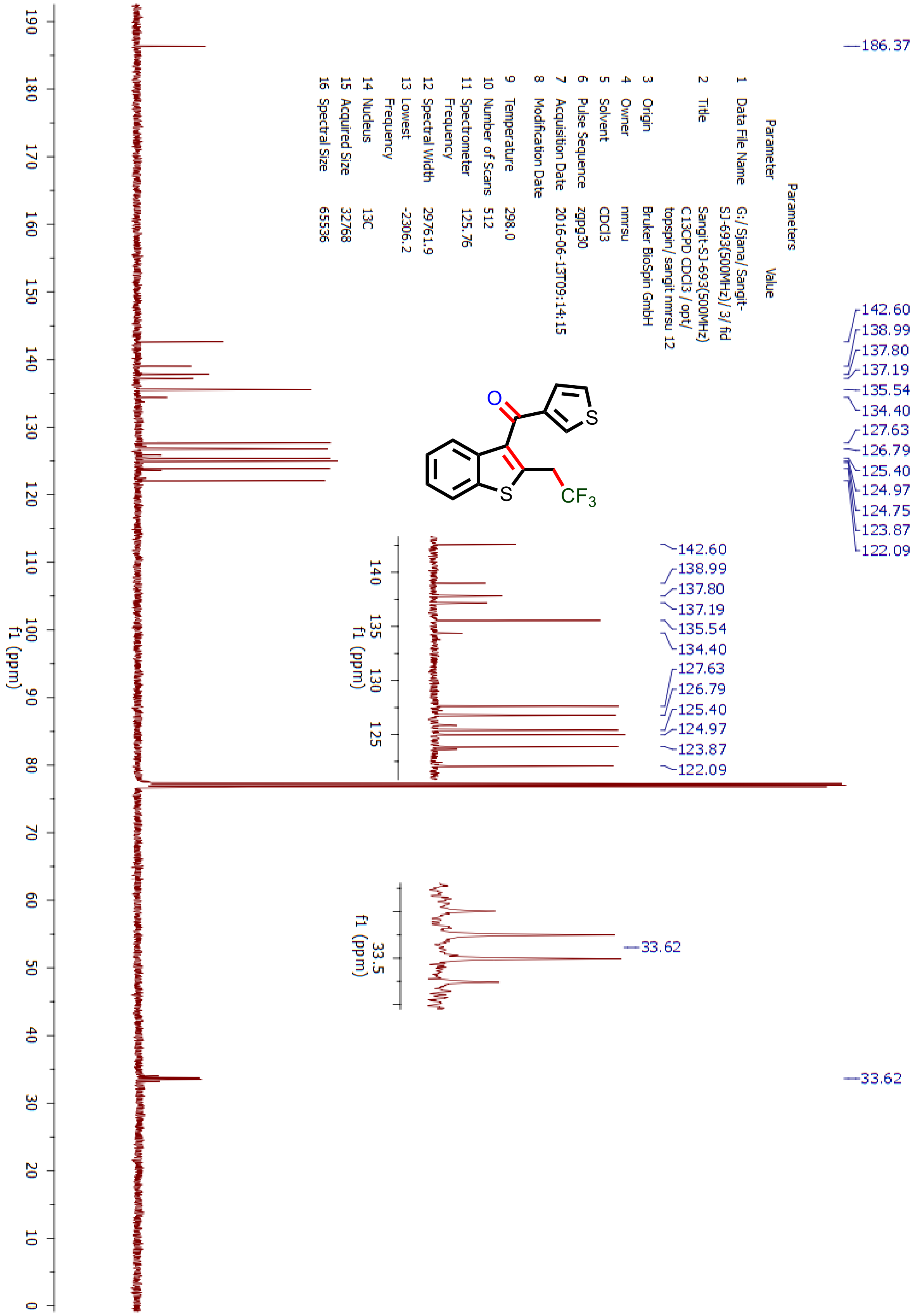

^{19}F NMR of Thiophen-3-yl(2-(2,2,2-trifluoroethyl)benzo[*b*]thiophen-3-yl)methanone (4j)

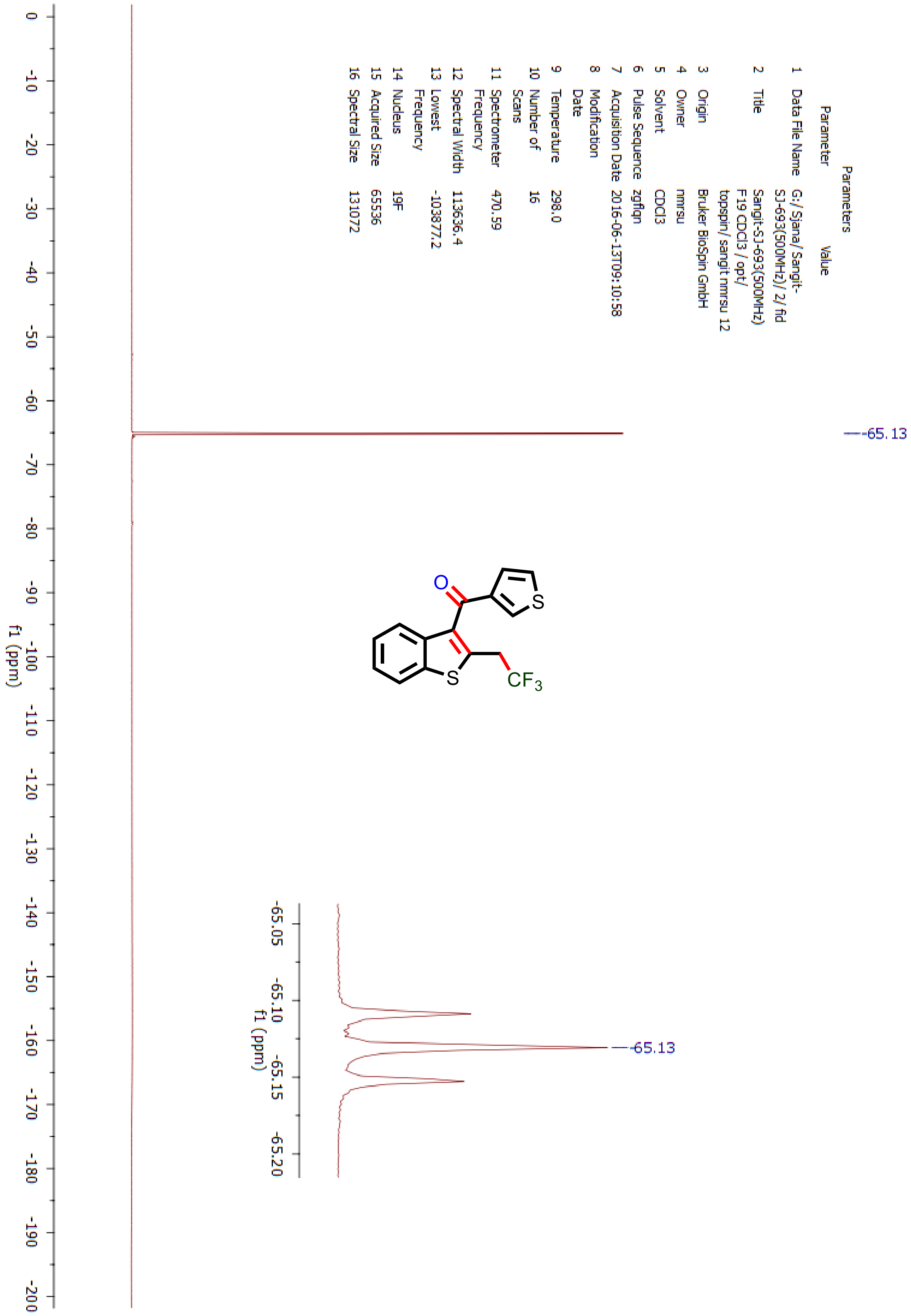

HRMS of Thiophen-3-yl(2-(2,2,2-trifluoroethyl)benzo[*b*]thiophen-3-yl)methanone (4j)

Display Report

Analysis Info

Analysis Name	D:\Data\user data\2016\SEPT-2016\09-sep-2016\Dr S. Kumar-SJ-693_1-A,2_01_7339.d	Acquisition Date	9/9/2016 12:37:54 PM
Method	hrlcms_pos_low_tunemix.m	Operator	DIMPLE
Sample Name	Dr S. Kumar-SJ-693	Instrument	micrOTOF-Q II 10330
Comment			

Acquisition Parameter

Source Type	ESI	Ion Polarity	Positive	Set Nebulizer	1.0 Bar
Focus	Active	Set Capillary	4500 V	Set Dry Heater	250 °C
Scan Begin	50 m/z	Set End Plate Offset	-500 V	Set Dry Gas	7.0 l/min
Scan End	3000 m/z	Set Collision Cell RF	130.0 Vpp	Set Divert Valve	Waste

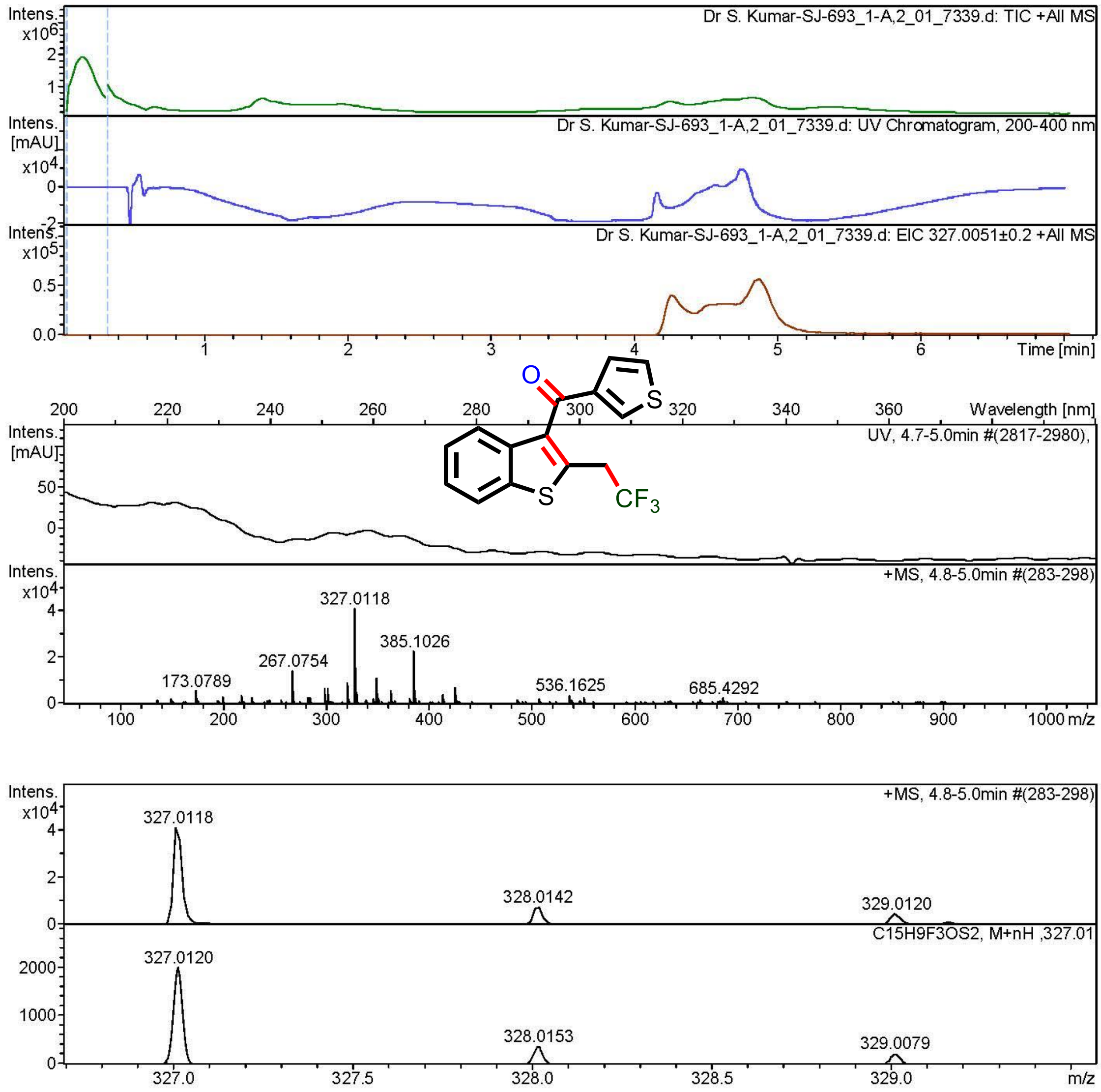

^{1}H NMR of Naphthalen-2-yl(2-(2,2,2-trifluoroethyl)benzo[*b*]thiophen-3-yl)methanone (4k)

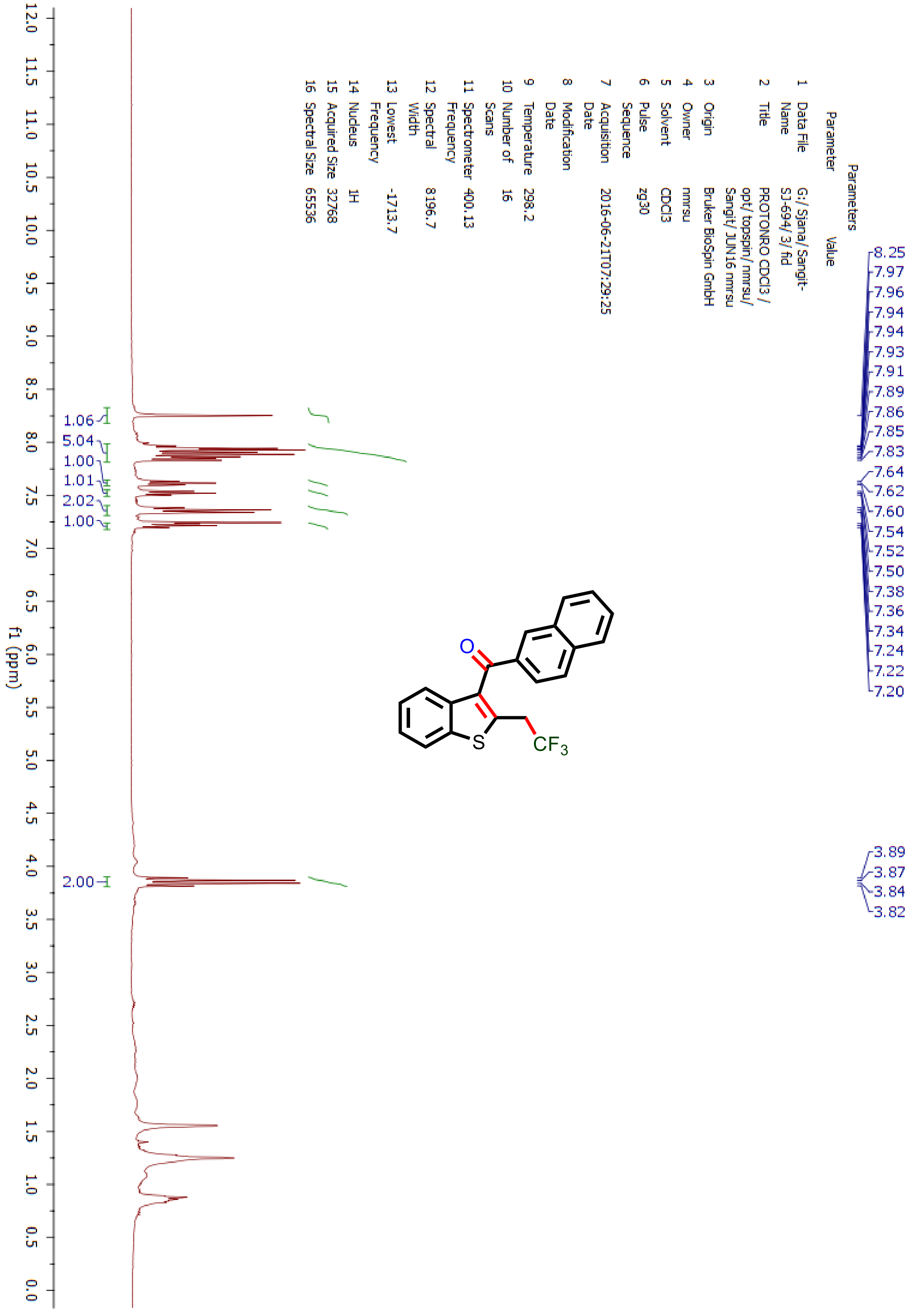

^{13}C NMR of Naphthalen-2-yl(2-(2,2,2-trifluoroethyl)benzo[*b*]thiophen-3-yl)methanone (4k)

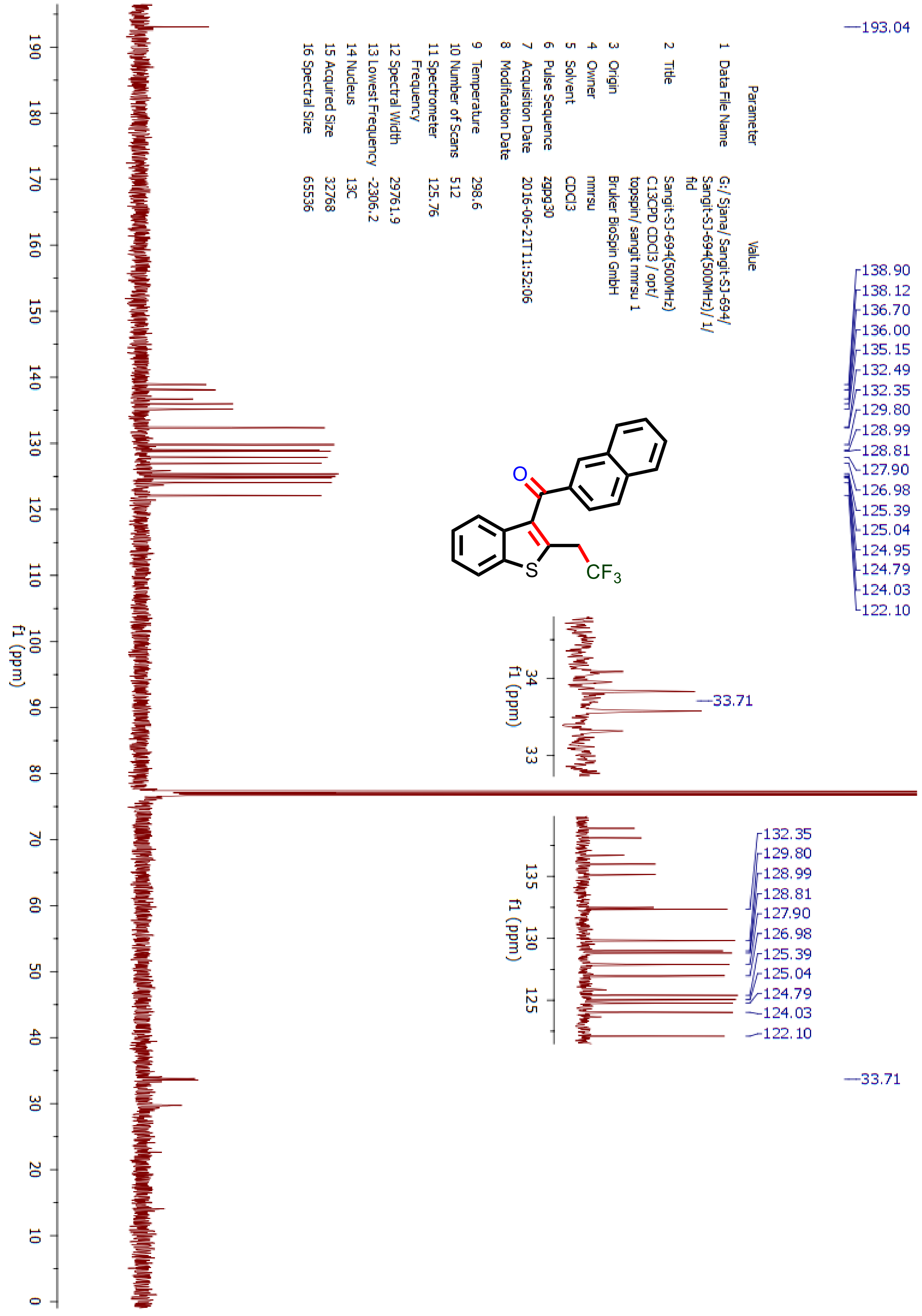

^{19}F NMR of Naphthalen-2-yl(2-(2,2,2-trifluoroethyl)benzo[*b*]thiophen-3-yl)methanone (4k)

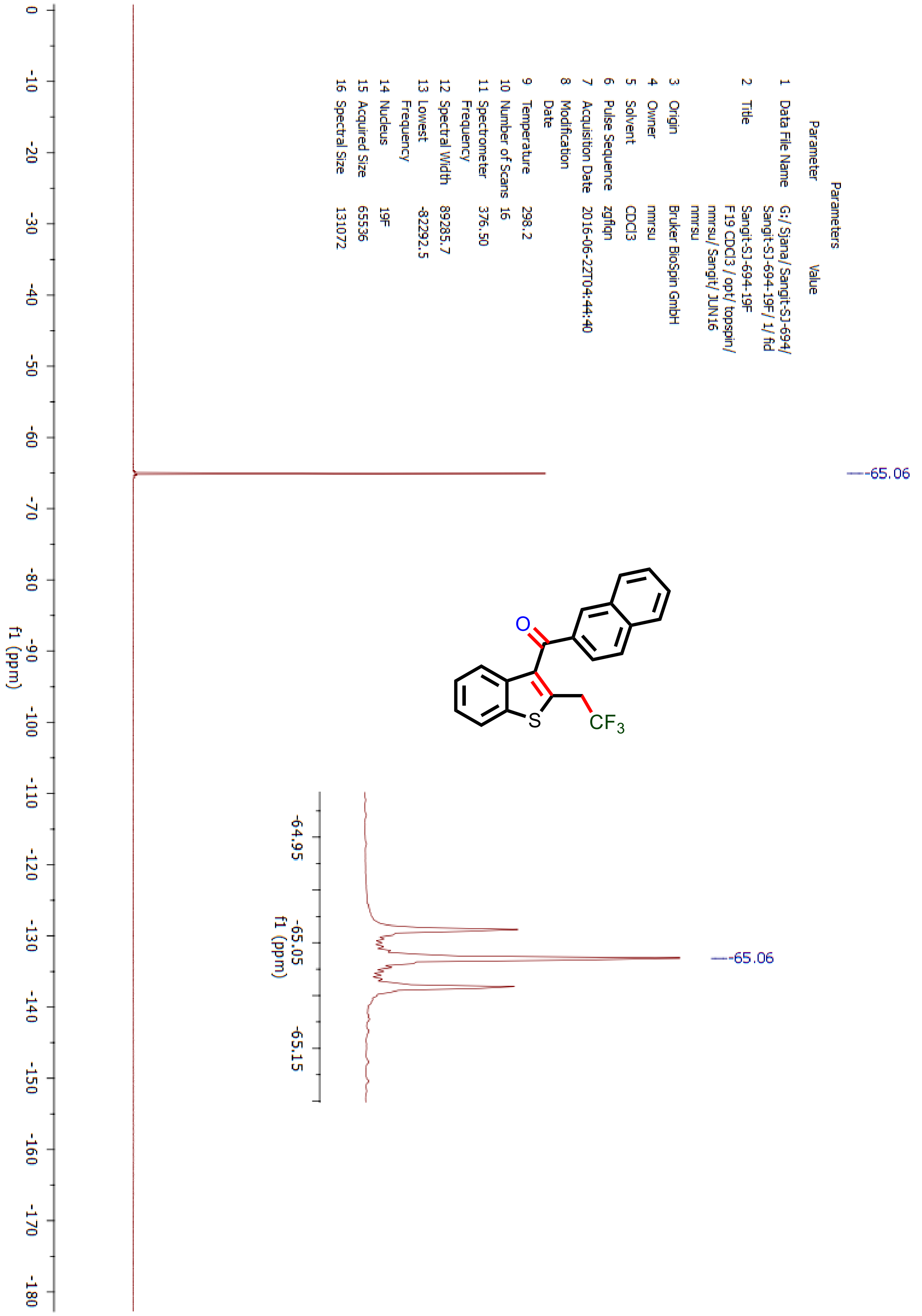

HRMS of Naphthalen-2-yl(2-(2,2,2-trifluoroethyl)benzo[*b*]thiophen-3-yl)methanone (4k)

Display Report

Analysis Info

		Acquisition Date	9/8/2016 12:55:06 PM
Analysis Name	D:\Data\user data\2016\SEPT-2016\08-sep-2016\Dr S. Kumar-SJ-694_1-B,6_01_7333.d		
Method	hrlcms_pos_low_tunemix.m	Operator	DIMPLE
Sample Name	Dr S. Kumar-SJ-694	Instrument	micrOTOF-Q II 10330
Comment			

Acquisition Parameter

Source Type	ESI	Ion Polarity	Positive	Set Nebulizer	1.0 Bar
Focus	Active	Set Capillary	4500 V	Set Dry Heater	250 °C
Scan Begin	50 m/z	Set End Plate Offset	-500 V	Set Dry Gas	7.0 l/min
Scan End	3000 m/z	Set Collision Cell RF	130.0 Vpp	Set Divert Valve	Waste

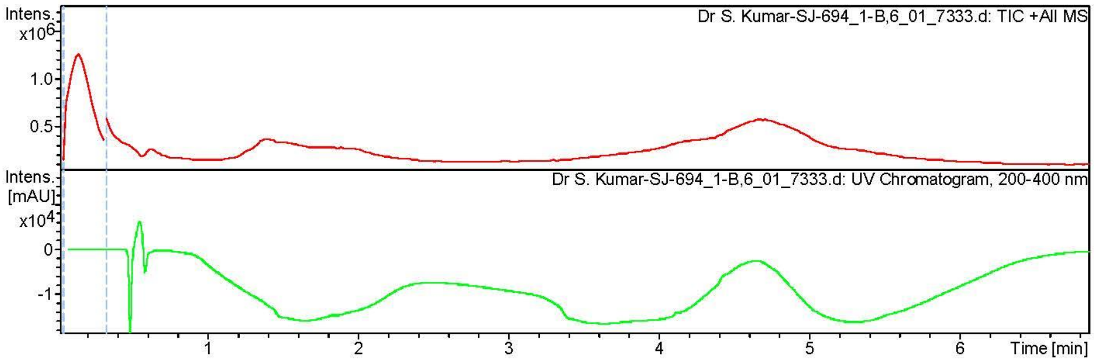

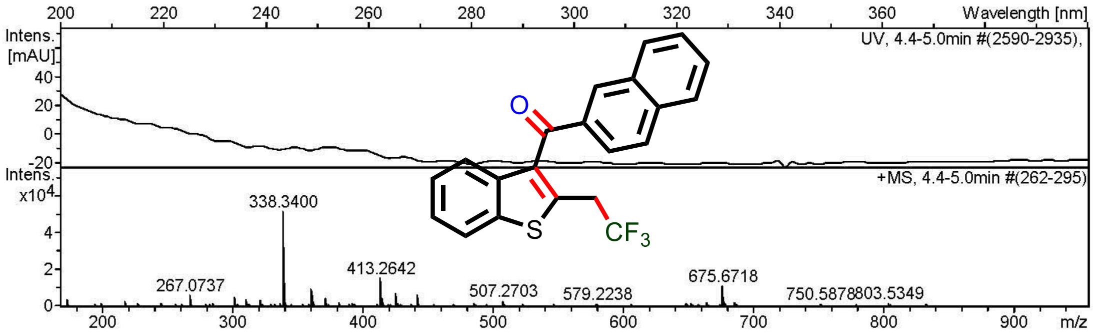

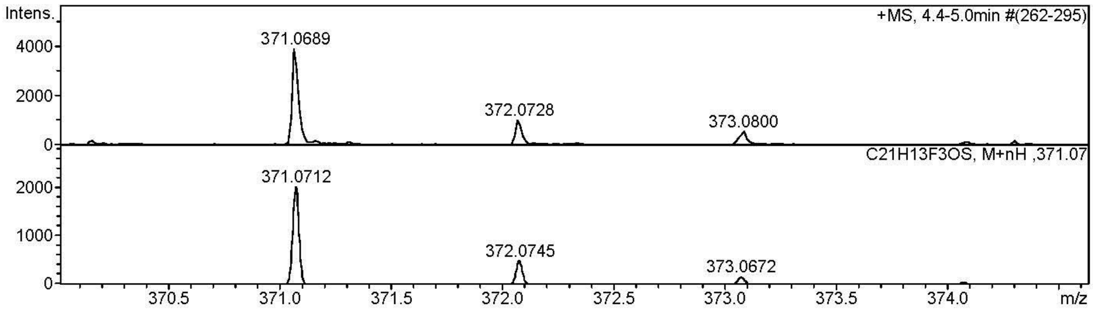

Bruker Compass DataAnalysis 4.0 printed: 9/8/2016 2:26:02 PM Page 1 of 1

^{1}H NMR of 4,4,4-trifluoro-1-(2-(2,2,2-trifluoroethyl)benzo[b]thiophen-3-yl)butan-1-one (4l major)

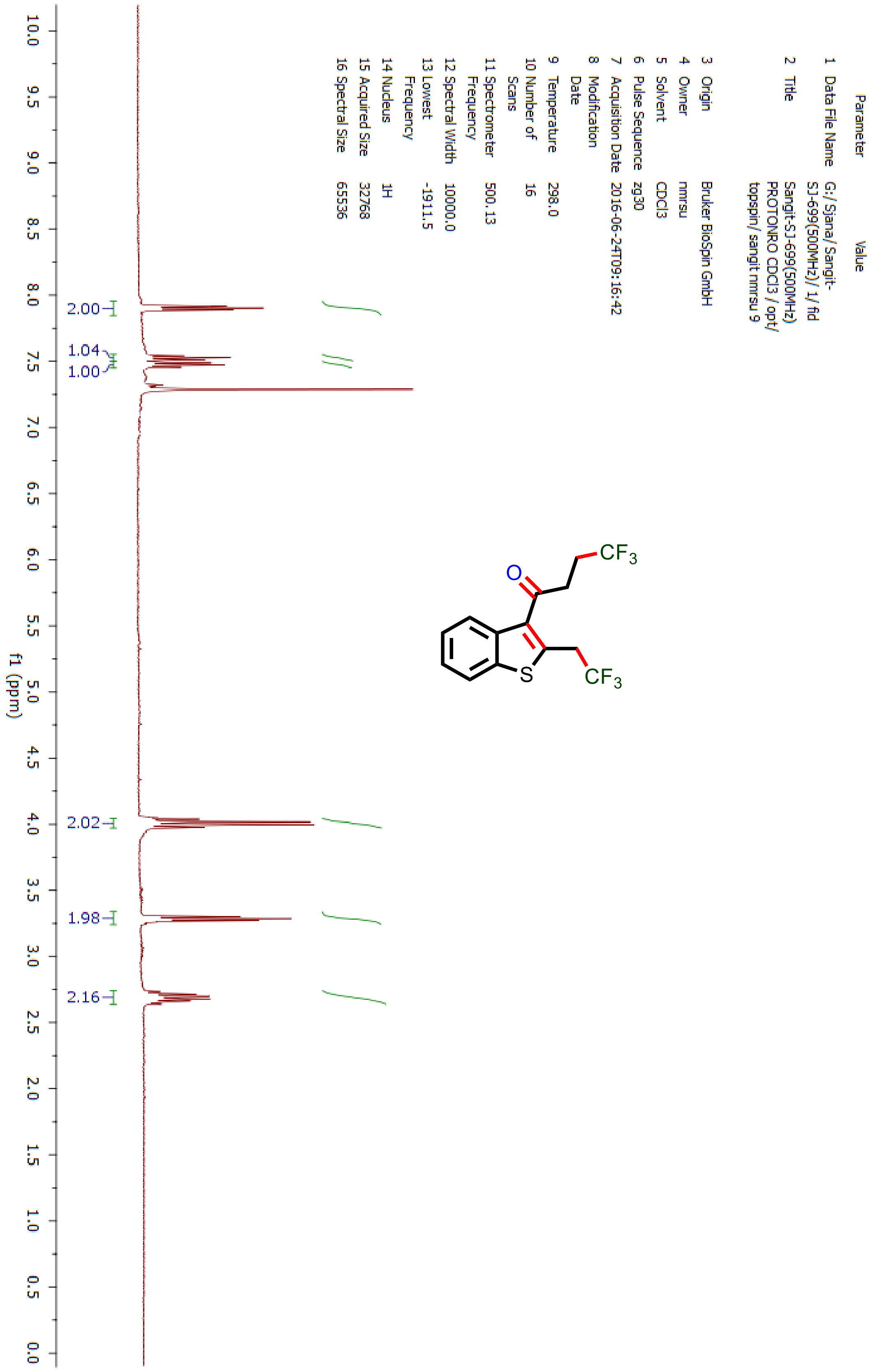

^{13}C NMR of 4,4,4-trifluoro-1-(2-(2,2,2-trifluoroethyl)benzo[b]thiophen-3-yl)butan-1-one (4l-major)

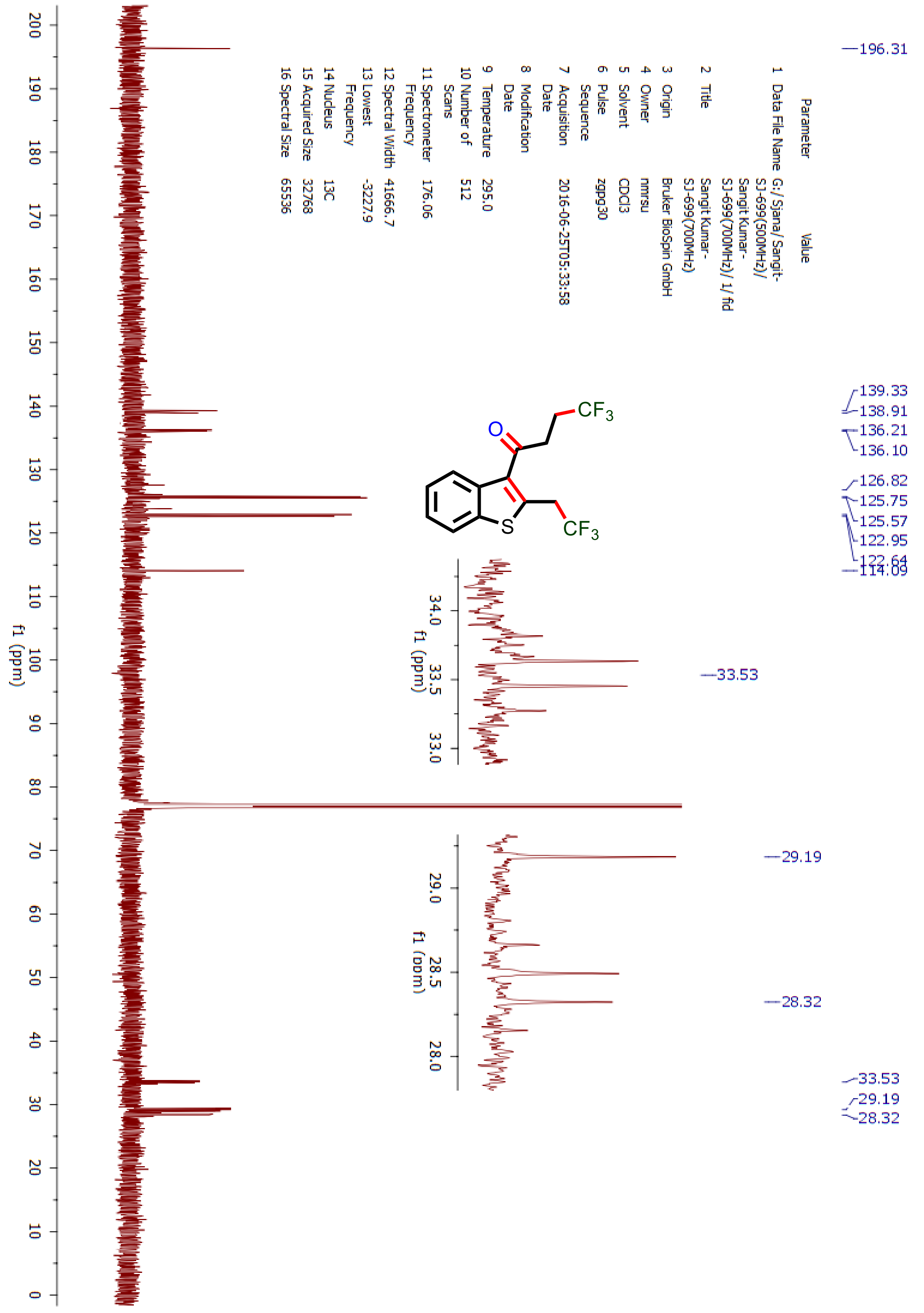

^{19}F NMR of 4,4,4-trifluoro-1-(2-(2,2,2-trifluoroethyl)benzo[b]thiophen-3-yl)butan-1-one (4l-major)

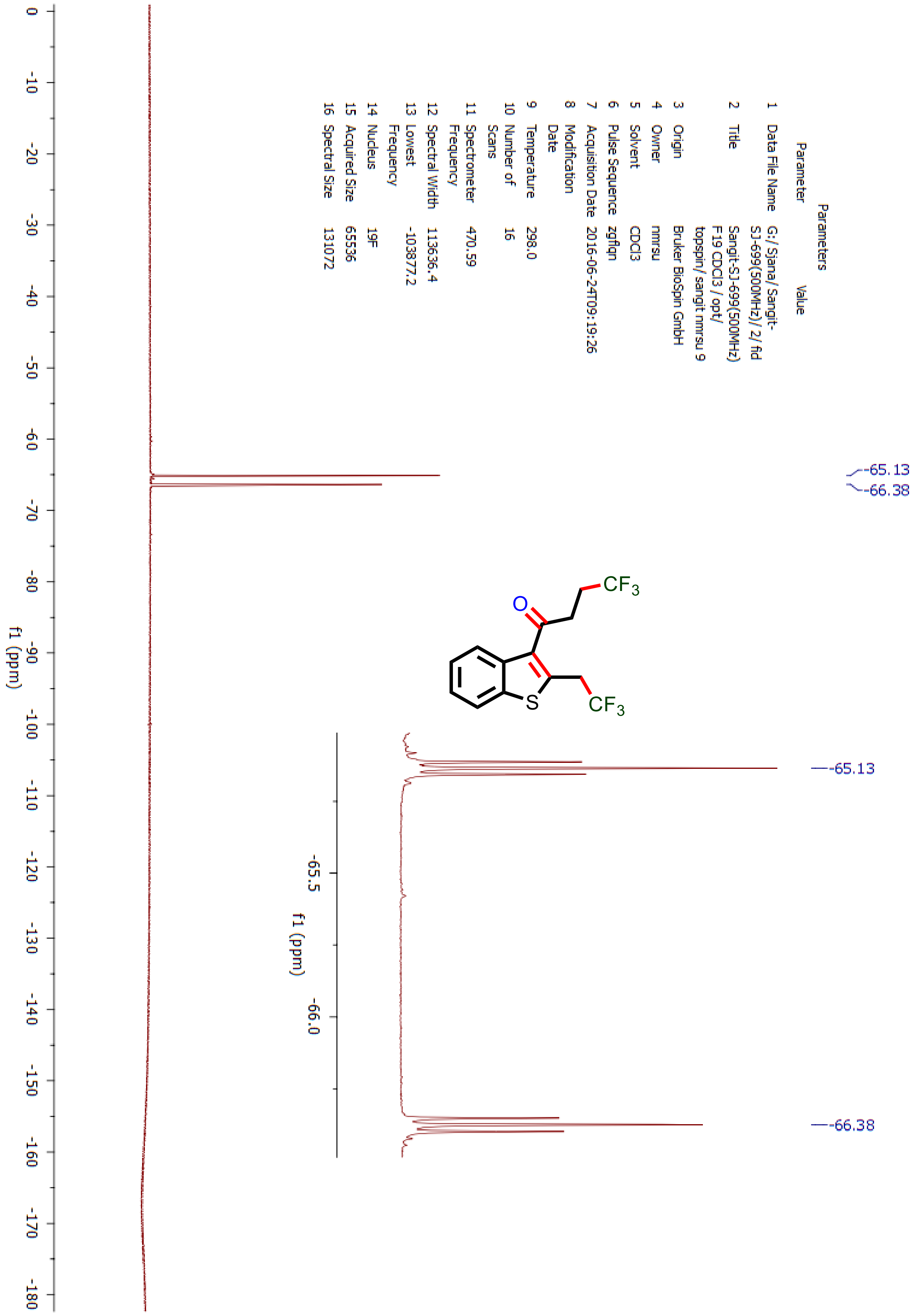

HRMS of 4,4,4-trifluoro-1-(2-(2,2,2-trifluoroethyl)benzo[b]thiophen-3-yl)butan-1-one (4l-major)

Display Report

Analysis Info

Analysis Name	D:\Data\user data\2016\SEPT-2016\08-sep-2016\Dr.S.Kumar-SJ-699.d	Acquisition Date	9/8/2016 4:33:25 PM
Method	tune_low_APCI.m	Operator	DIMPLE
Sample Name	SJ-699	Instrument	micrOTOF-Q II 10330
Comment			

Acquisition Parameter

Source Type	APCI	Ion Polarity	Positive	Set Nebulizer	3.1 Bar
Focus	Not active	Set Capillary	4500 V	Set Dry Heater	200 °C
Scan Begin	50 m/z	Set End Plate Offset	-500 V	Set Dry Gas	8.0 l/min
Scan End	3000 m/z	Set Collision Cell RF	130.0 Vpp	Set Divert Valve	Waste

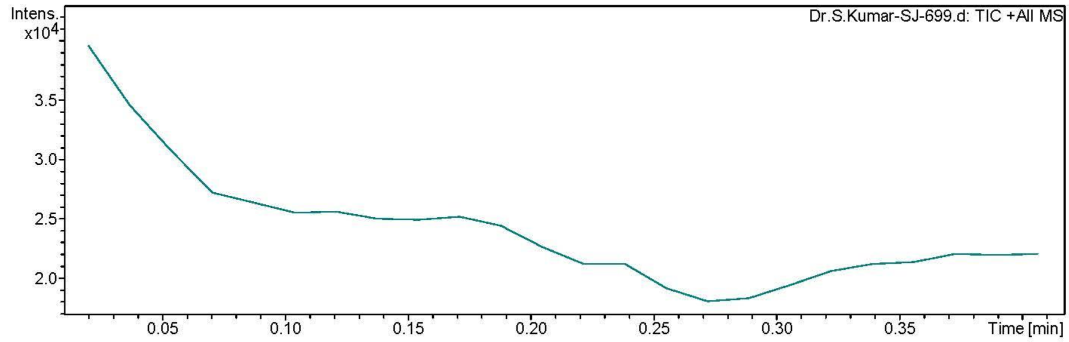

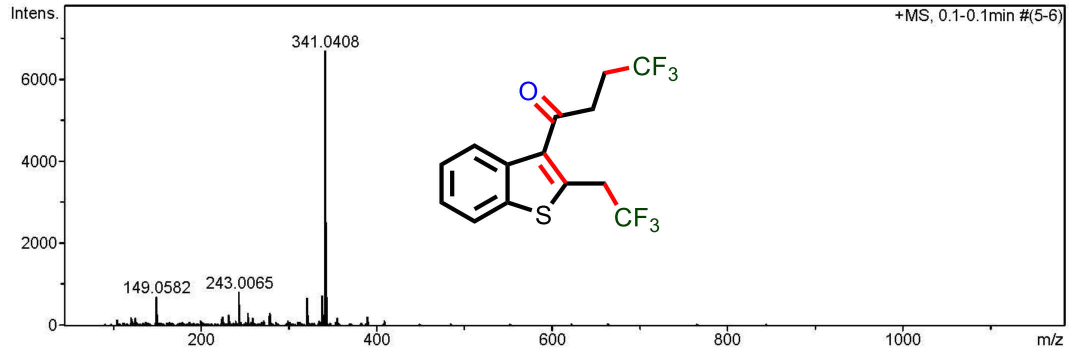

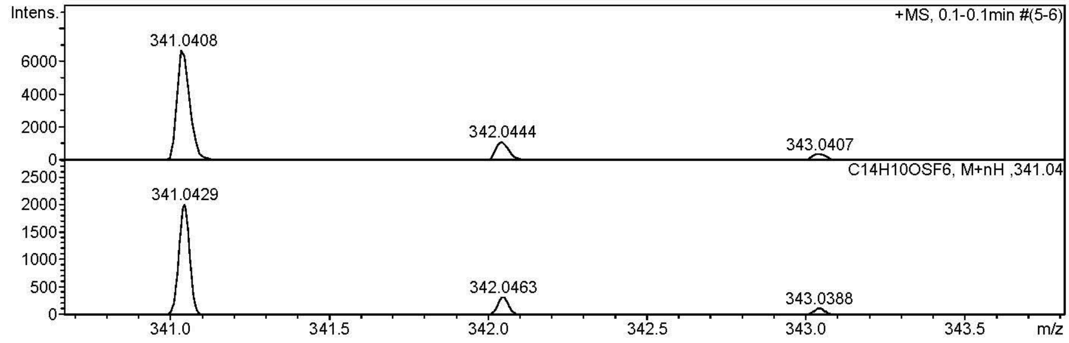

Bruker Compass DataAnalysis 4.0 printed: 3/7/2017 10:56:57 AM Page 1 of 1

HRMS of 5,5,5-trifluoro-1-(2-(2,2,2-trifluoroethyl)benzo[b]thiophen-3-yl)pentan-1-one (4l-minor)

Display Report

Analysis Info

Analysis Name	D:\Data\user data\2016\SEPT-2016\08-sep-2016\Dr.S.Kumar-SJ-699.d	Acquisition Date	9/8/2016 4:33:25 PM
Method	tune_low_APCI.m	Operator	DIMPLE
Sample Name	SJ-699	Instrument	micrOTOF-Q II 10330
Comment			

Acquisition Parameter

Source Type	APCI	Ion Polarity	Positive	Set Nebulizer	3.1 Bar
Focus	Not active	Set Capillary	4500 V	Set Dry Heater	200 °C
Scan Begin	50 m/z	Set End Plate Offset	-500 V	Set Dry Gas	8.0 l/min
Scan End	3000 m/z	Set Collision Cell RF	130.0 Vpp	Set Divert Valve	Waste

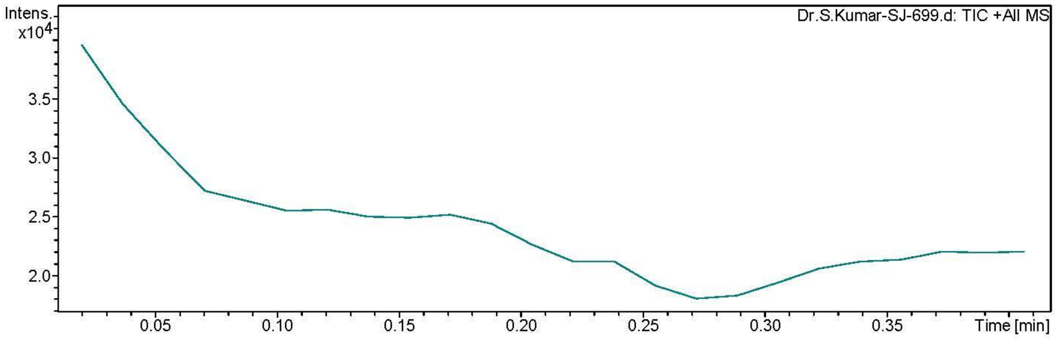

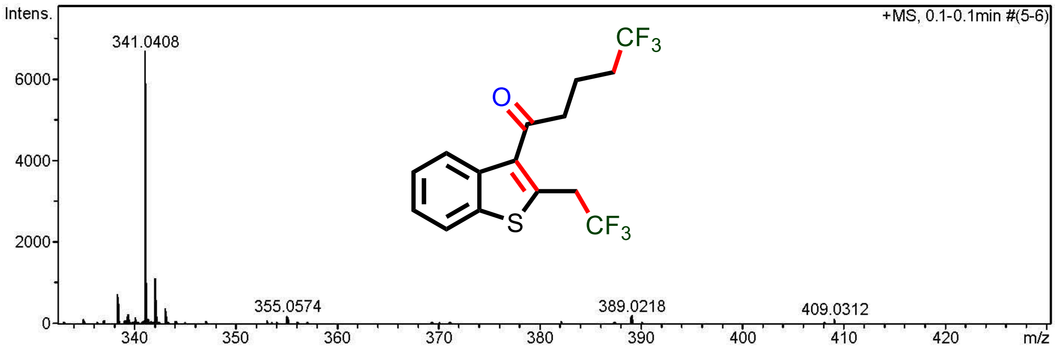

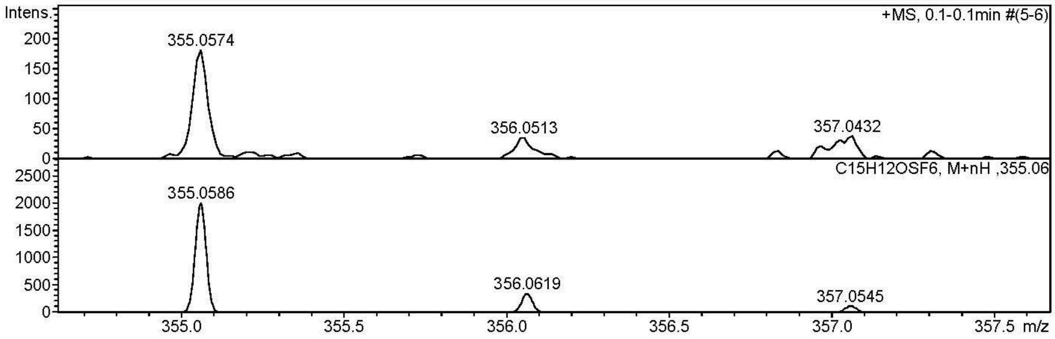

Bruker Compass DataAnalysis 4.0 printed: 3/7/2017 10:58:35 AM Page 1 of 1

^{1}H NMR of 1-(2-(2,2,2-trifluoroethyl)benzo[*b*]thiophen-3-yl)ethan-1-one (4m)

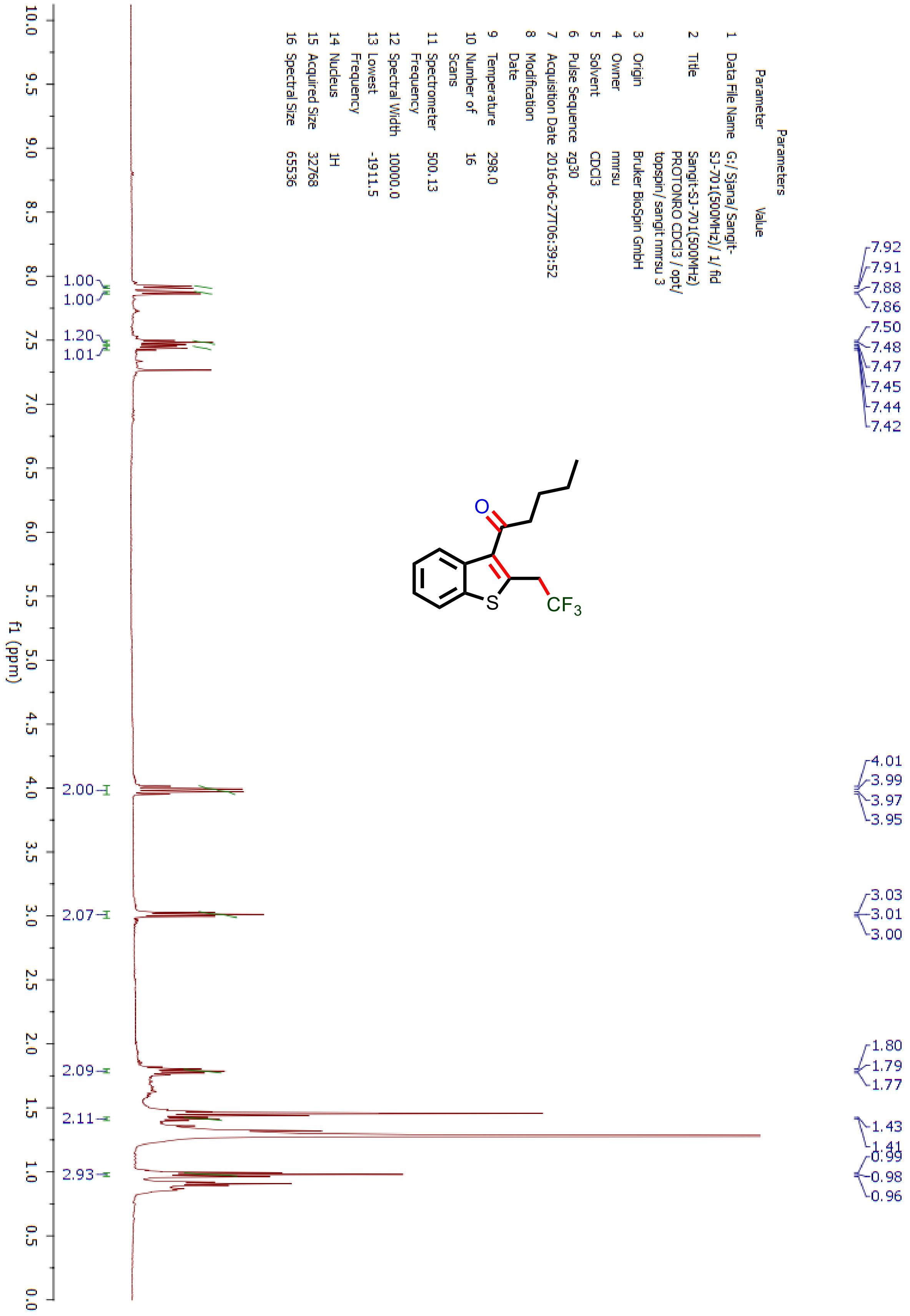

^{13}C NMR of 1-(2-(2,2,2-trifluoroethyl)benzo[*b*]thiophen-3-yl)ethan-1-one (4m)

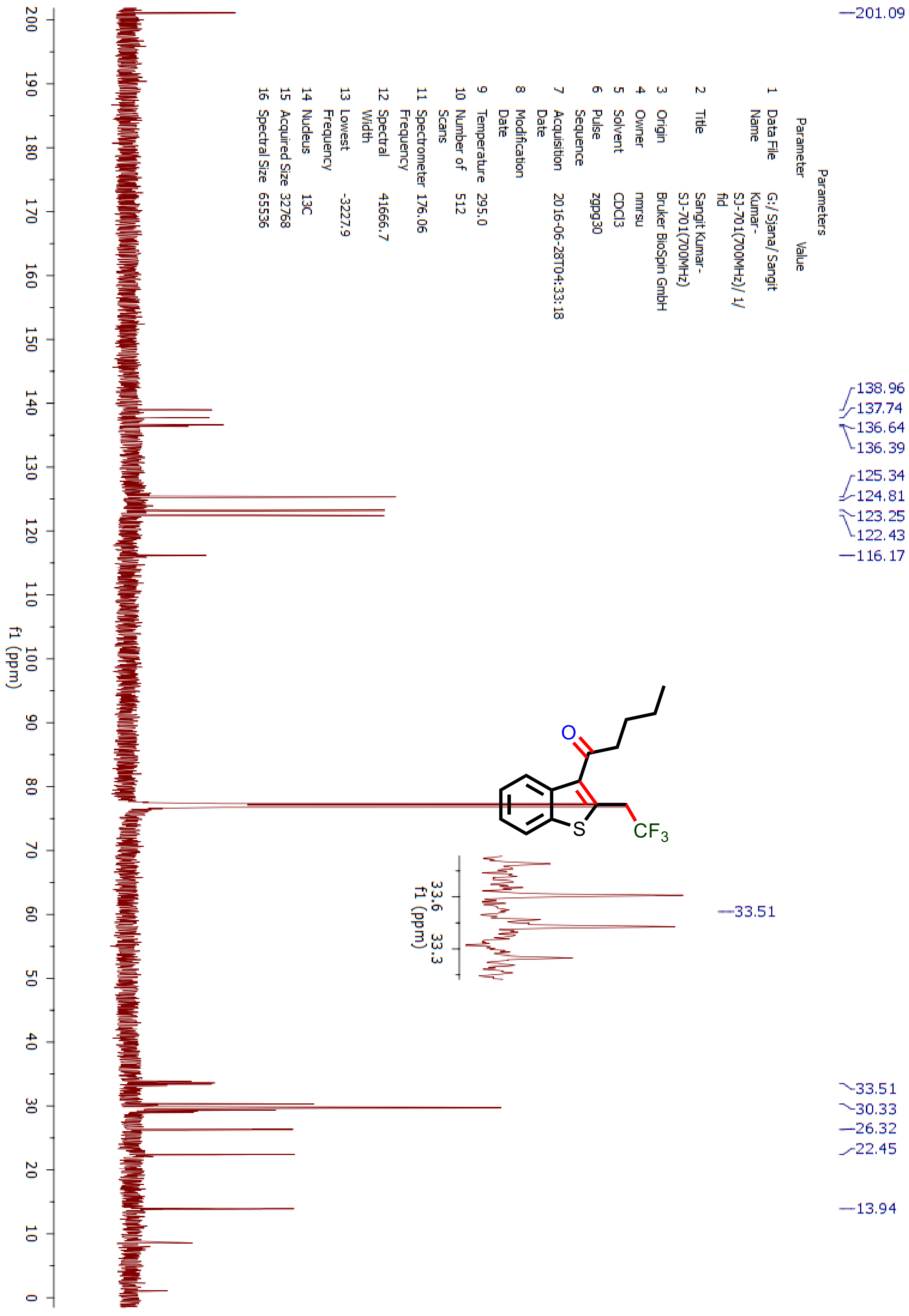

^{19}F NMR of 1-(2-(2,2,2-trifluoroethyl)benzo[*b*]thiophen-3-yl)ethan-1-one (4m)

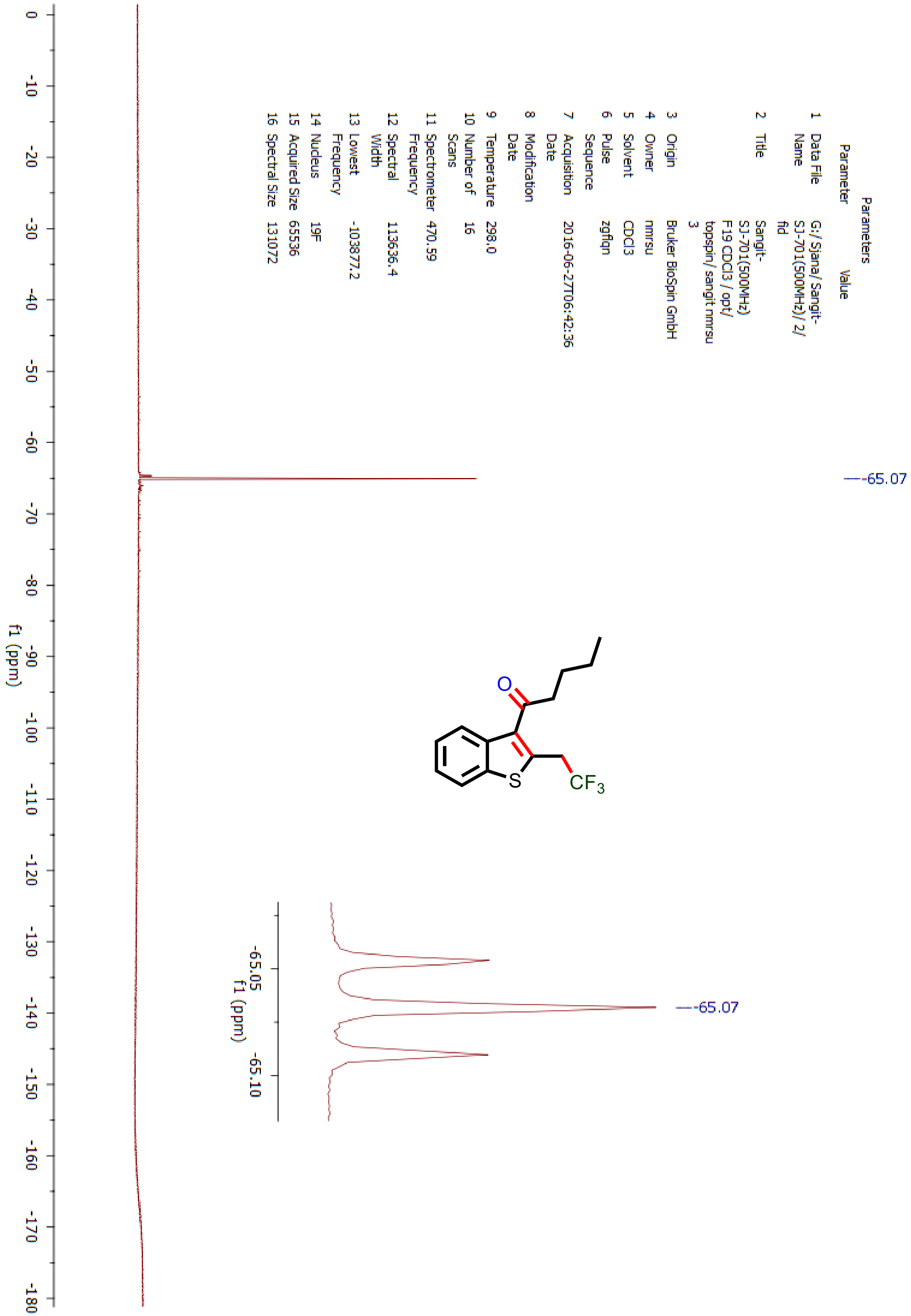

HRMS of 1-(2-(2,2,2-trifluoroethyl)benzo[*b*]thiophen-3-yl)ethan-1-one (4m)

Display Report

Analysis Info

Analysis Name	D:\Data\user data\2016\SEPT-2016\08-sep-2016\Dr.S.Kumar-SJ-701.d	Acquisition Date	9/8/2016 4:30:22 PM
Method	tune_low_APCI.m	Operator	DIMPLE
Sample Name	SJ-701	Instrument	micrOTOF-Q II 10330
Comment			

Acquisition Parameter

Source Type	APCI	Ion Polarity	Positive	Set Nebulizer	3.1 Bar
Focus	Not active	Set Capillary	4500 V	Set Dry Heater	200 °C
Scan Begin	50 m/z	Set End Plate Offset	-500 V	Set Dry Gas	8.0 l/min
Scan End	3000 m/z	Set Collision Cell RF	130.0 Vpp	Set Divert Valve	Waste

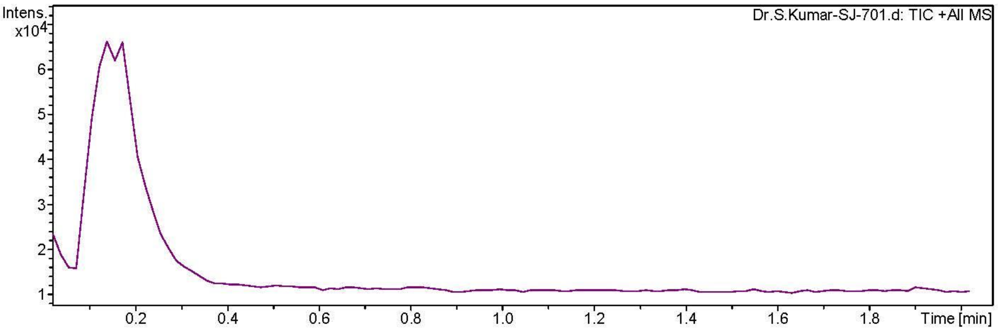

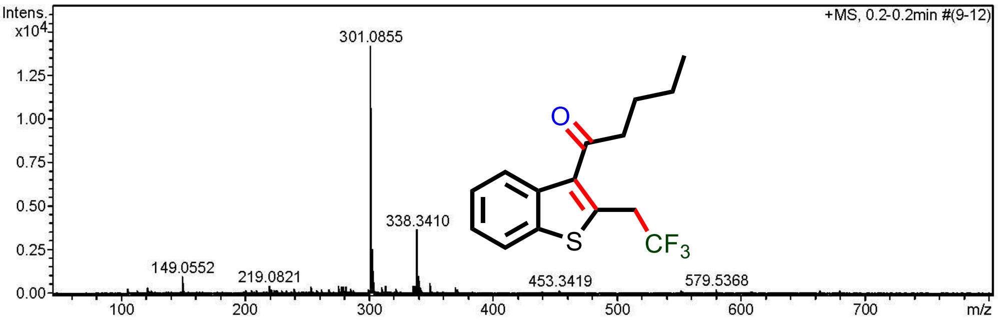

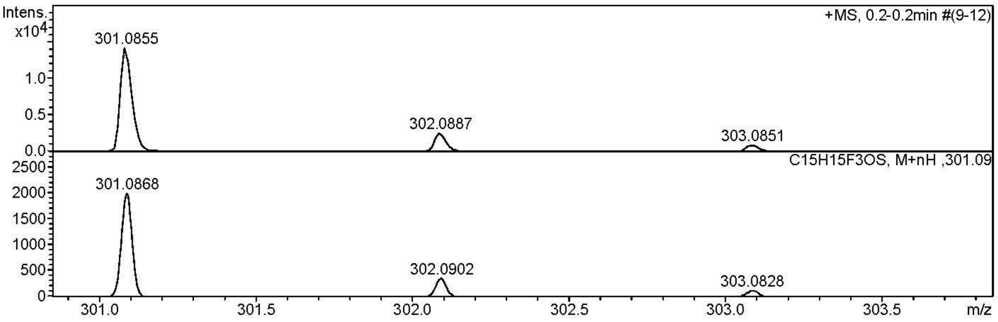

Bruker Compass DataAnalysis 4.0 printed: 9/8/2016 4:39:27 PM Page 1 of 1

^{1}H NMR of Phenyl(1-tosyl-2-(2,2,2-trifluoroethyl)-1H-indol-3-yl)methanone (6a)

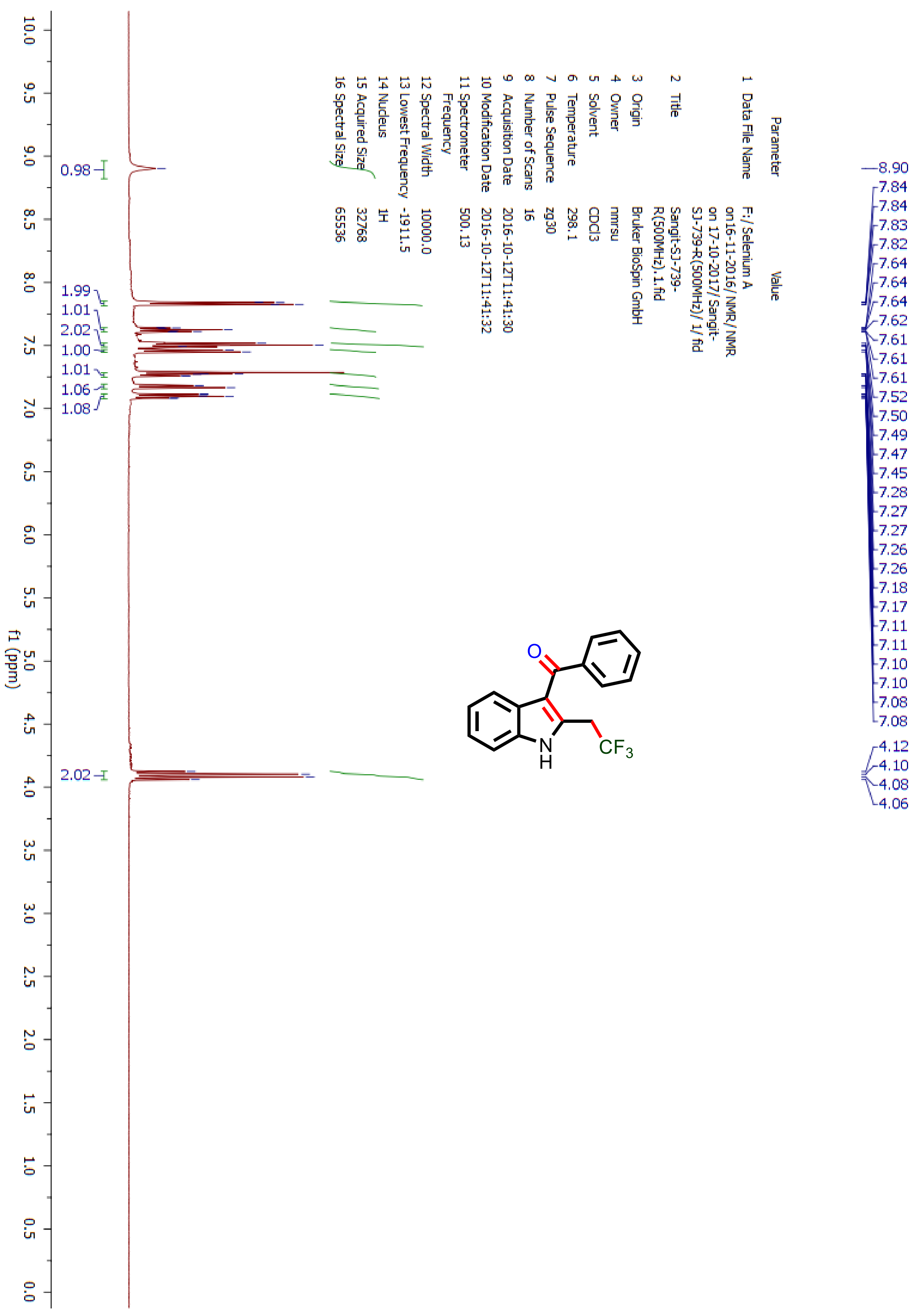

^{13}C NMR of Phenyl(1-tosyl-2-(2,2,2-trifluoroethyl)-1H-indol-3-yl)methanone (6a)

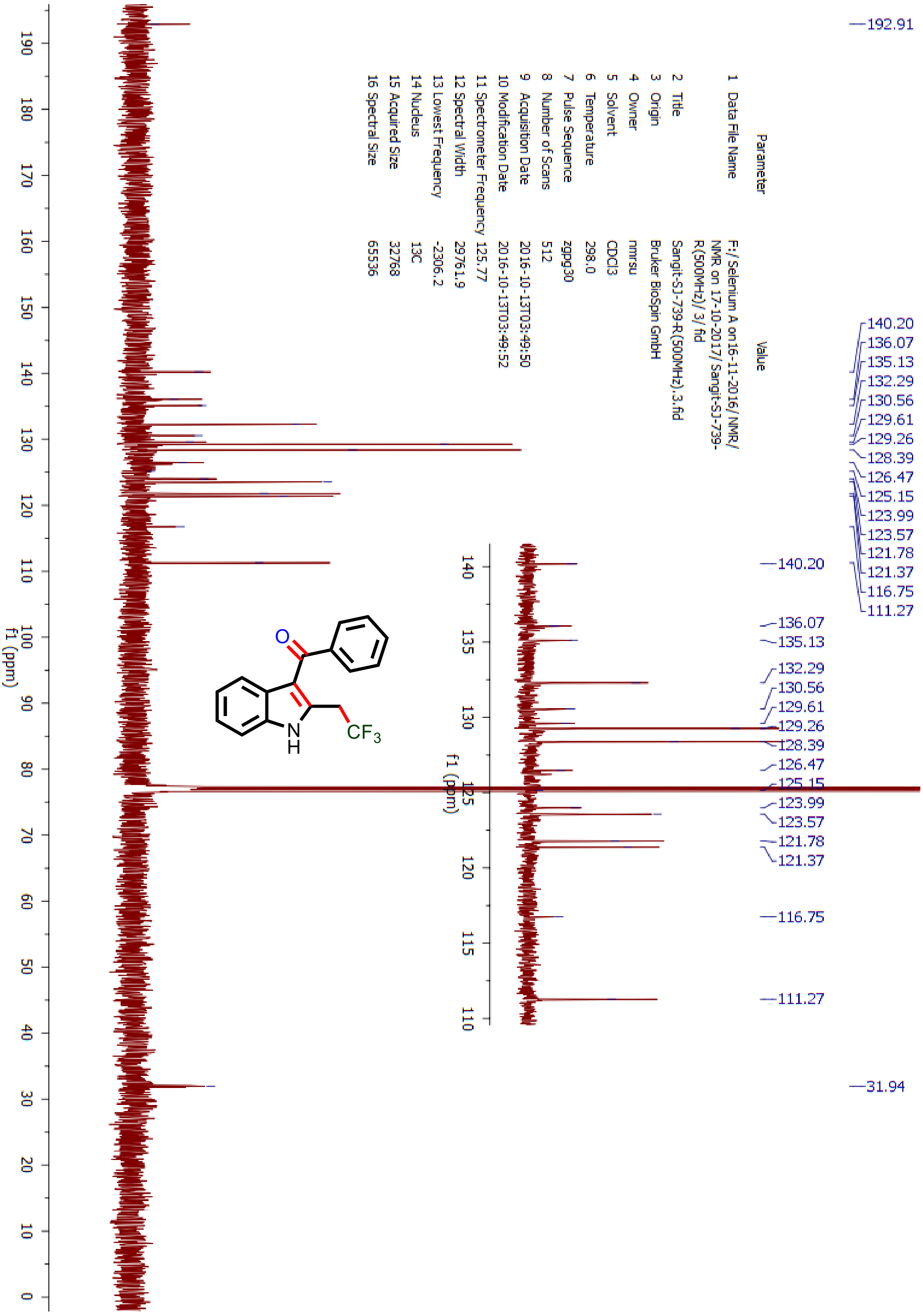

^{19}F NMR of Phenyl(1-tosyl-2-(2,2,2-trifluoroethyl)-1H-indol-3-yl)methanone (6a)

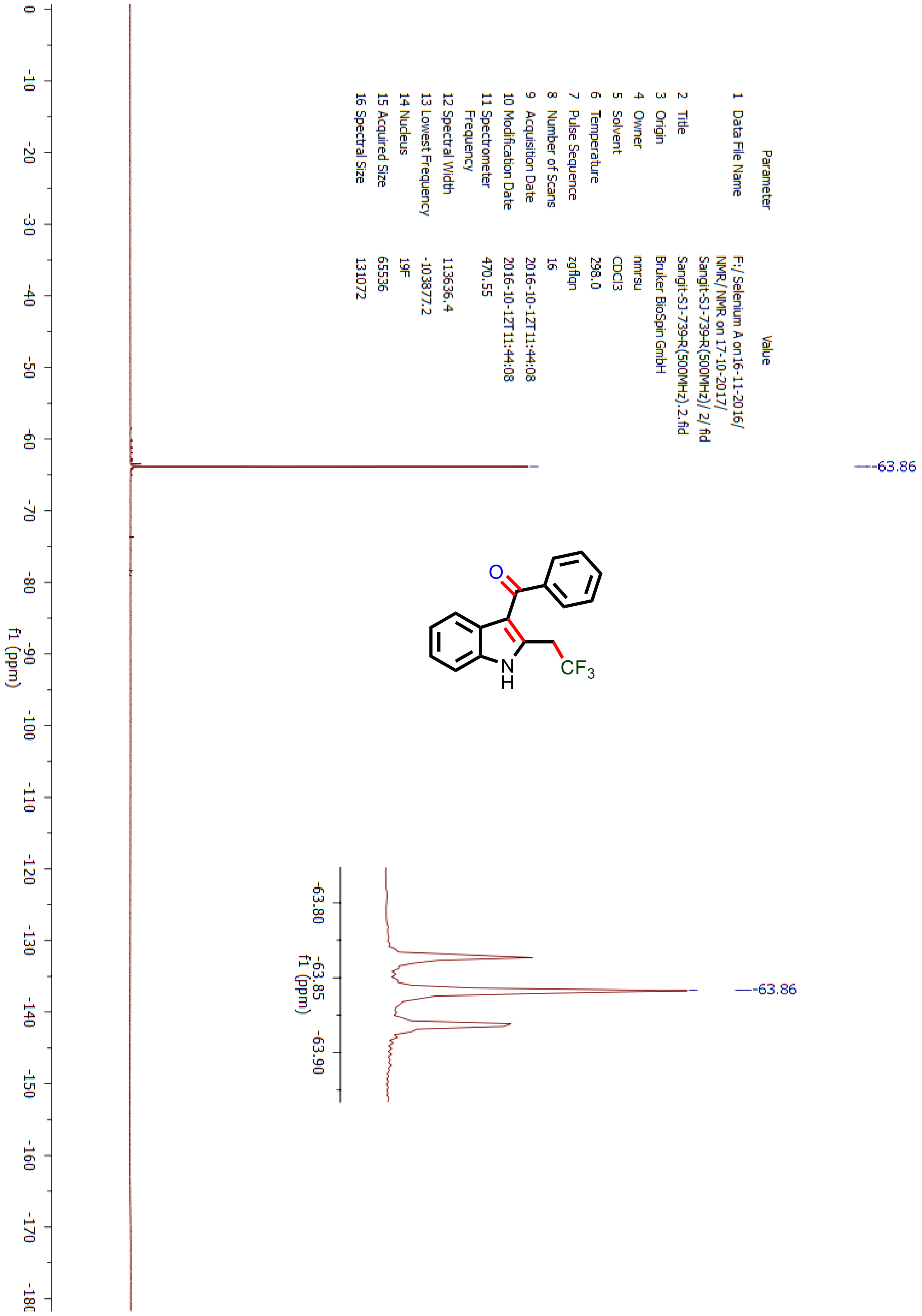

HRMS of Phenyl(1-tosyl-2-(2,2,2-trifluoroethyl)-1H-indol-3-yl)methanone (6a)

Display Report

Analysis Info

Analysis Name	D:\Data\user data\2016\october 2016\19-10-2016\Dr.S.Kumar-SJ-739_1-C,6_01_7751.d	Acquisition Date	10/19/2016 11:57:09 AM
Method	hrlcms_pos_mid_tunemix.m	Operator	DIMPLE
Sample Name	Dr.S.Kumar-SJ-739	Instrument	micrOTOF-Q II 10330
Comment			

Acquisition Parameter

Source Type	ESI	Ion Polarity	Positive	Set Nebulizer	0.3 Bar
Focus	Active	Set Capillary	4500 V	Set Dry Heater	200 °C
Scan Begin	50 m/z	Set End Plate Offset	-500 V	Set Dry Gas	4.0 l/min
Scan End	3000 m/z	Set Collision Cell RF	450.0 Vpp	Set Divert Valve	Waste

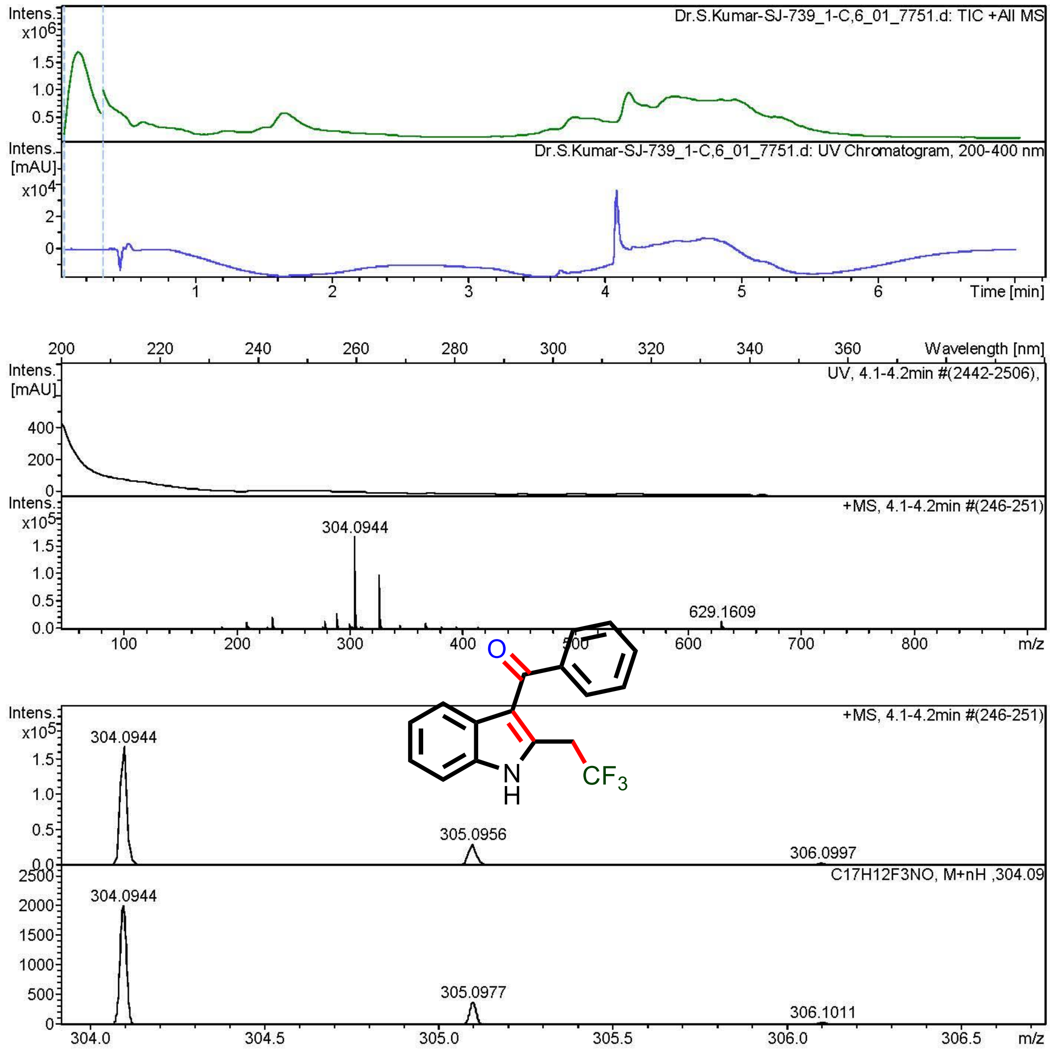

^{1}H NMR of *p*-Tolyl(1-tosyl-2-(2,2,2-trifluoroethyl)-1H-indol-3-yl)methanone (6b)

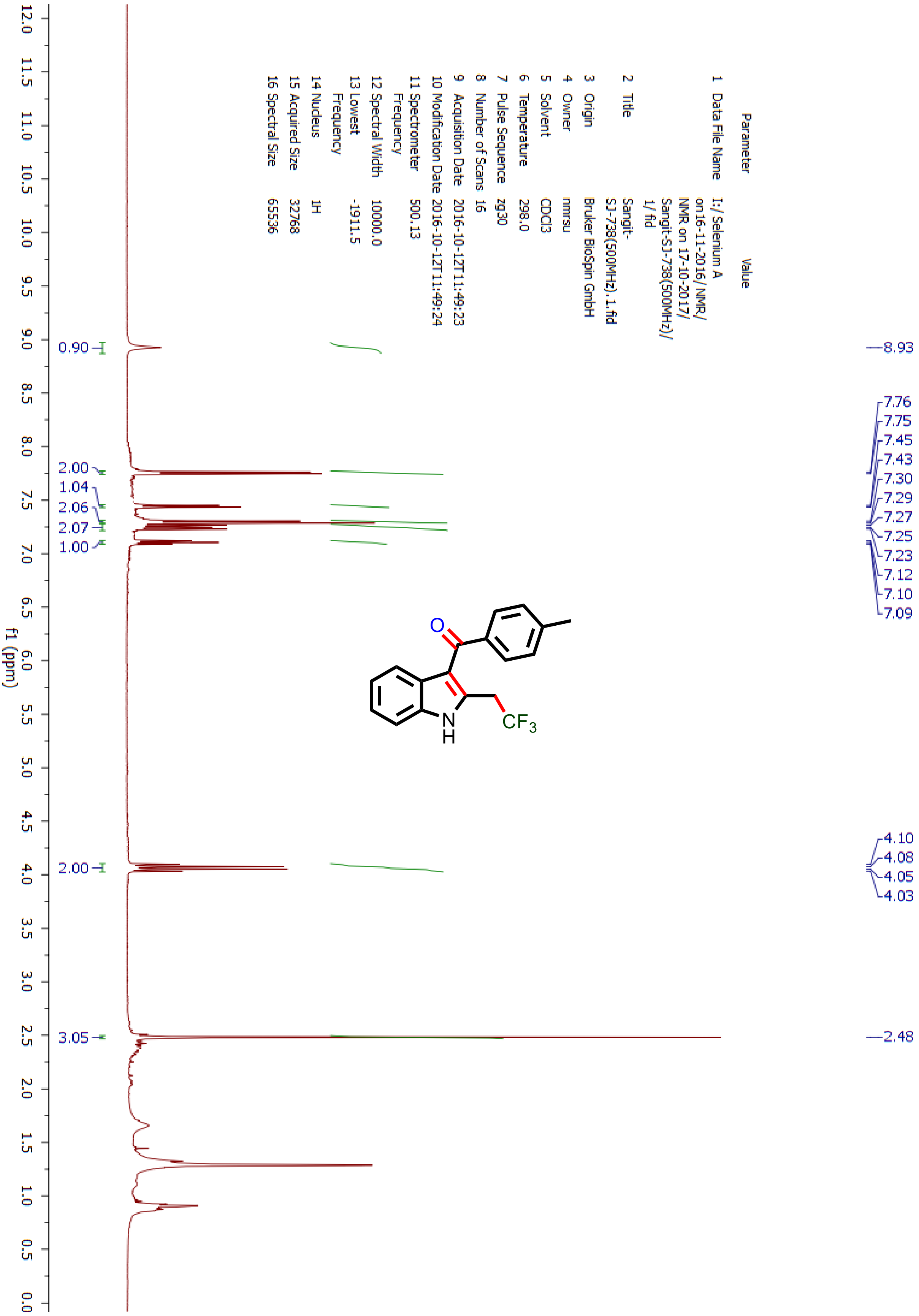

^{13}C NMR of *p*-Tolyl(1-tosyl-2-(2,2,2-trifluoroethyl)-1H-indol-3-yl)methanone (6b)

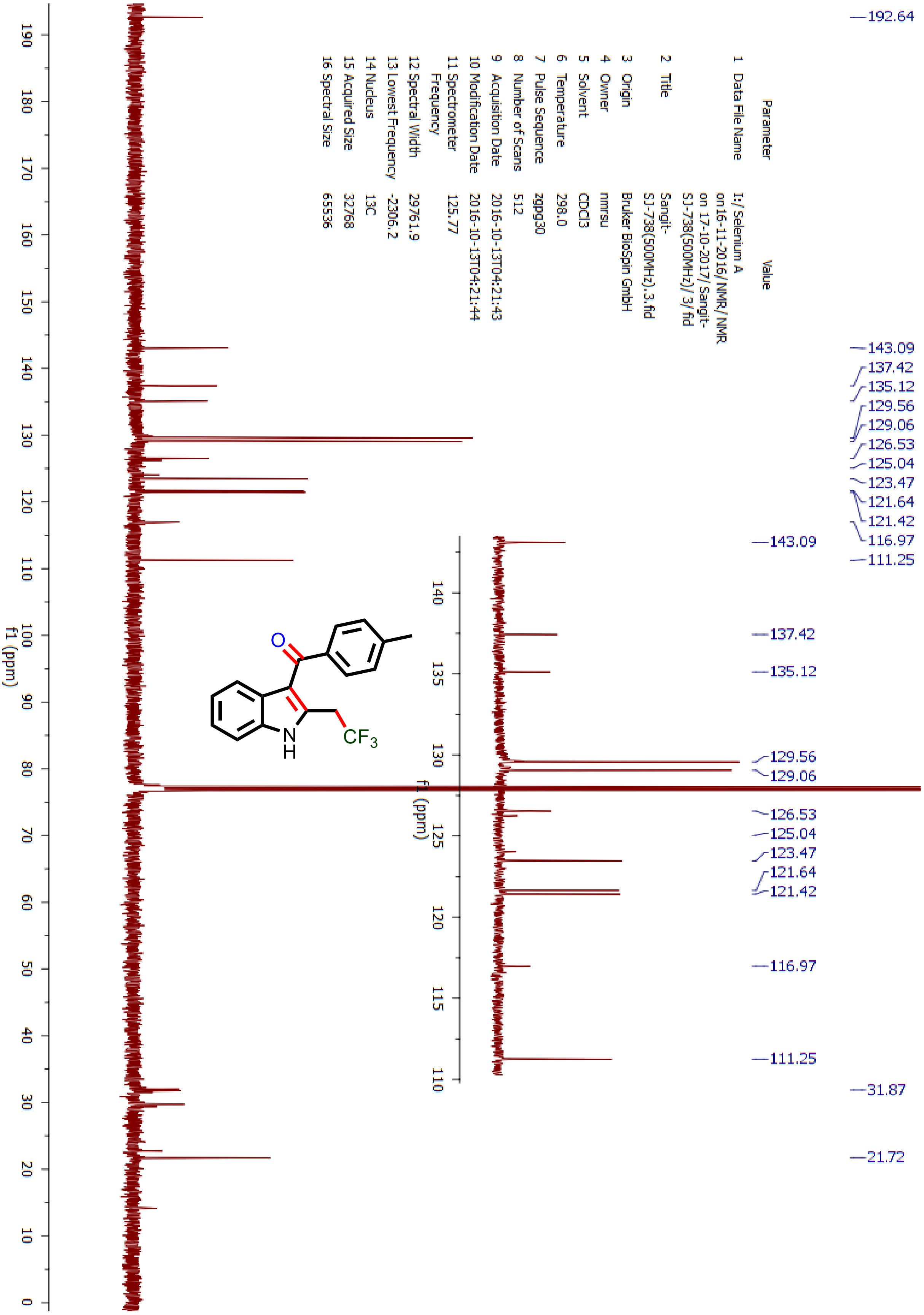

^{19}F NMR of *p*-Tolyl(1-tosyl-2-(2,2,2-trifluoroethyl)-1H-indol-3-yl)methanone (6b)

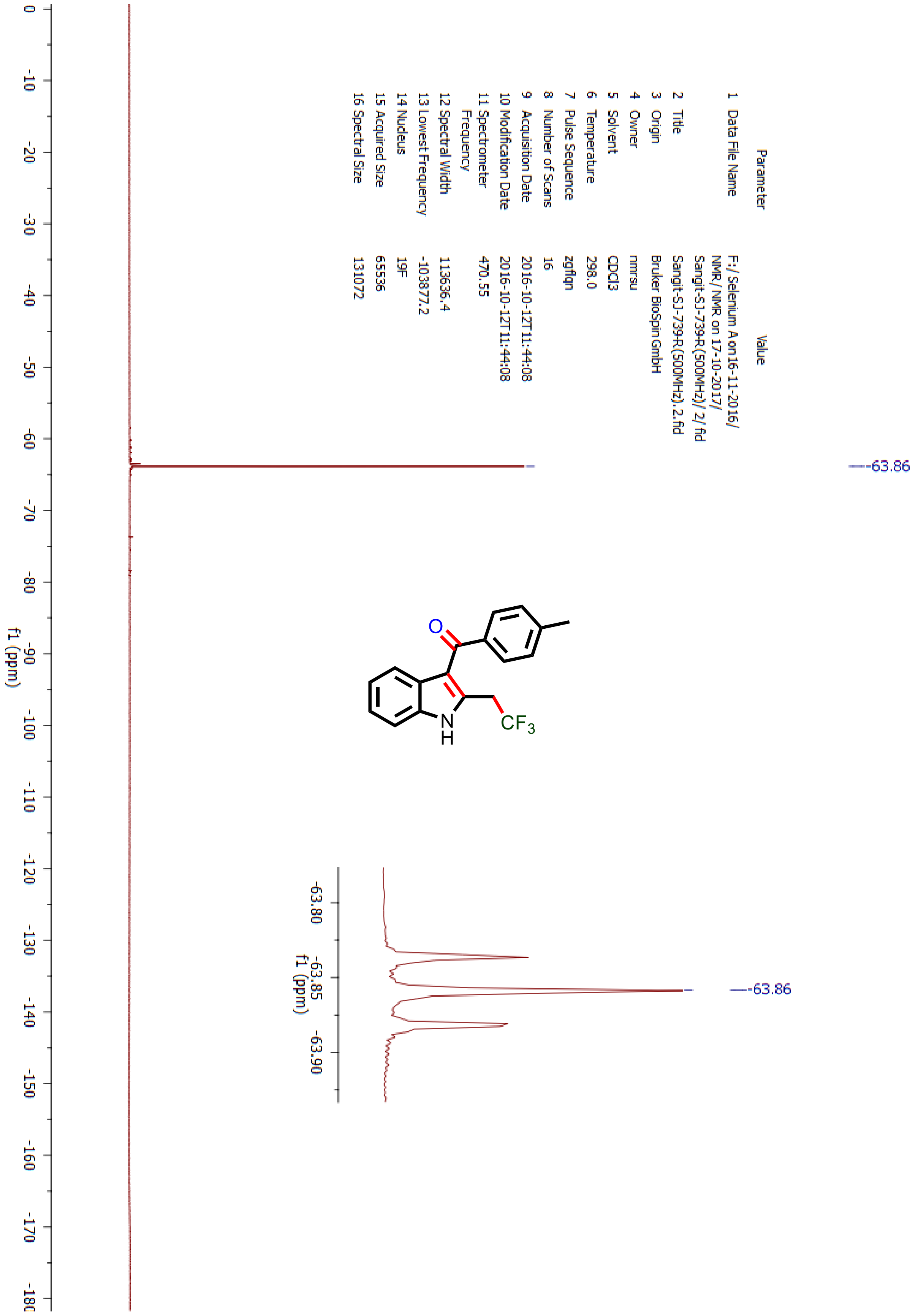

HRMS of *p*-Tolyl(1-tosyl-2-(2,2,2-trifluoroethyl)-1H-indol-3-yl)methanone (6b)

Display Report

Analysis Info

		Acquisition Date	10/21/2016 3:36:40 PM
Analysis Name	D:\Data\user data\2016\october 2016\20-10-2016\Dr.S.Kumar-SJ-738.d		
Method	Pos_LOW_tunemix.m	Operator	DIMPLE
Sample Name	SJ-738	Instrument	micrOTOF-Q II 10330
Comment			

Acquisition Parameter

Source Type	ESI	Ion Polarity	Positive	Set Nebulizer	0.4 Bar
Focus	Active	Set Capillary	4500 V	Set Dry Heater	200 °C
Scan Begin	50 m/z	Set End Plate Offset	-500 V	Set Dry Gas	4.0 l/min
Scan End	3000 m/z	Set Collision Cell RF	130.0 Vpp	Set Divert Valve	Source

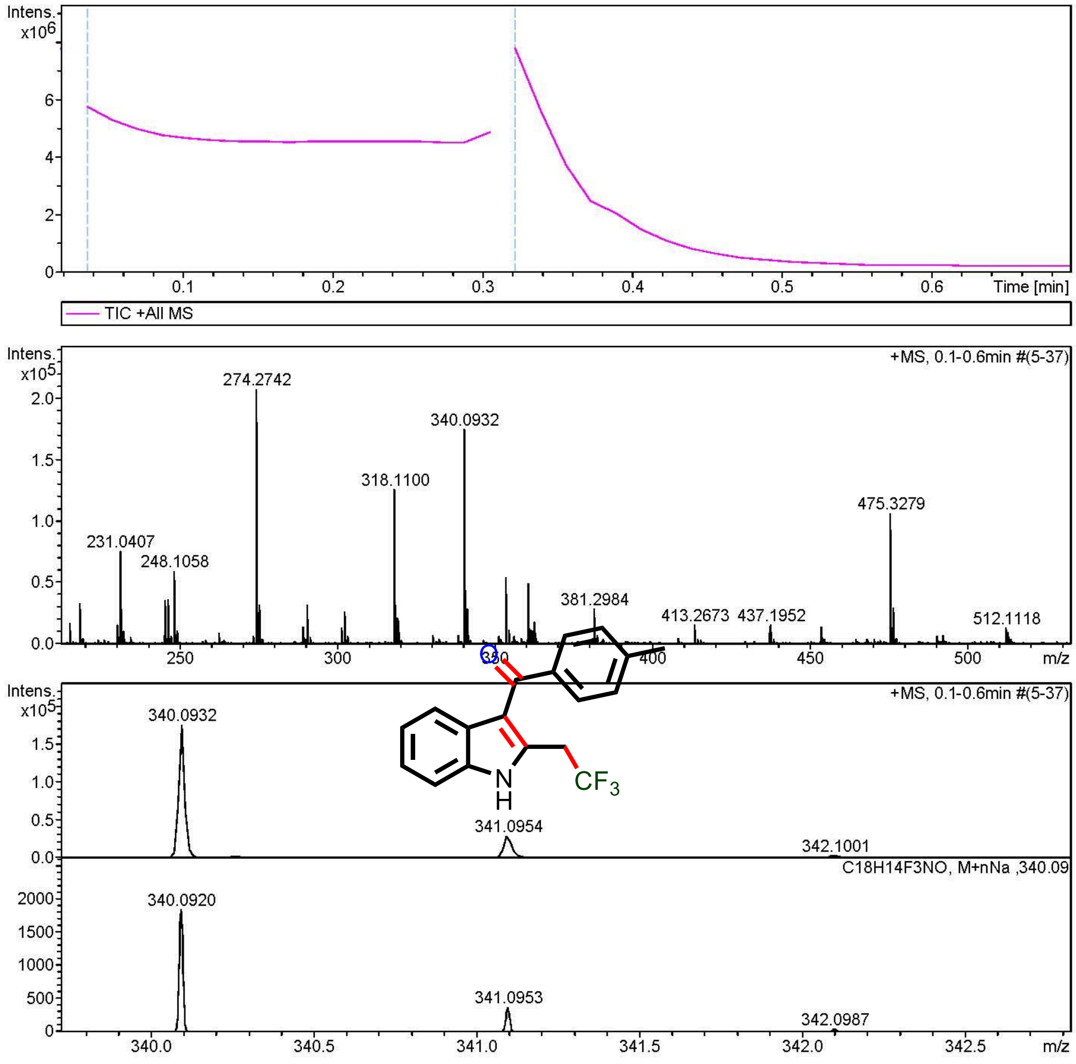

^{1}H NMR of (4-Bromophenyl)(1-tosyl-2-(2,2,2-trifluoroethyl)-1H-indol-3-yl)methanone (6c)

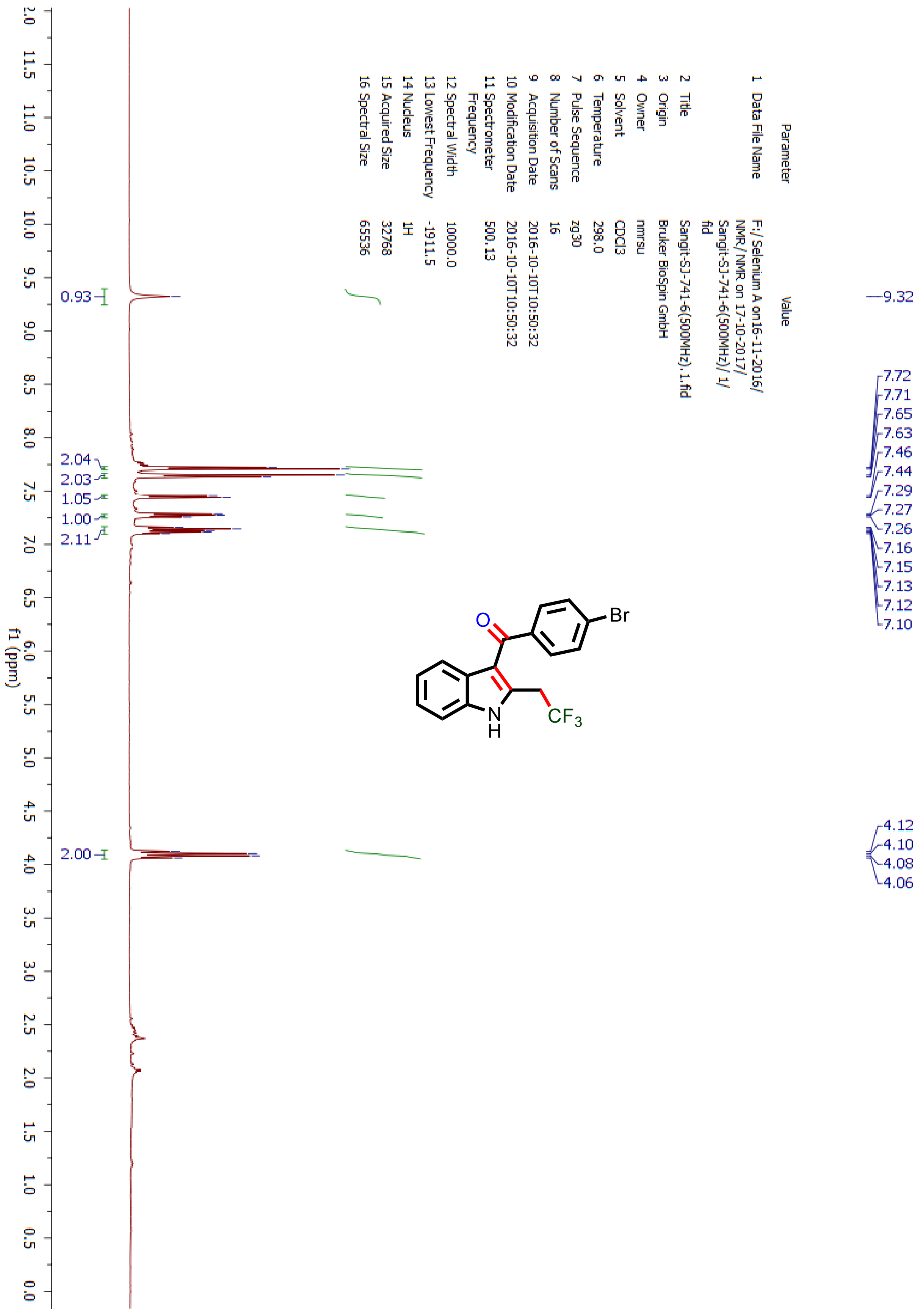

^{13}C NMR of (4-Bromophenyl)(1-tosyl-2-(2,2,2-trifluoroethyl)-1H-indol-3-yl)methanone (6c)

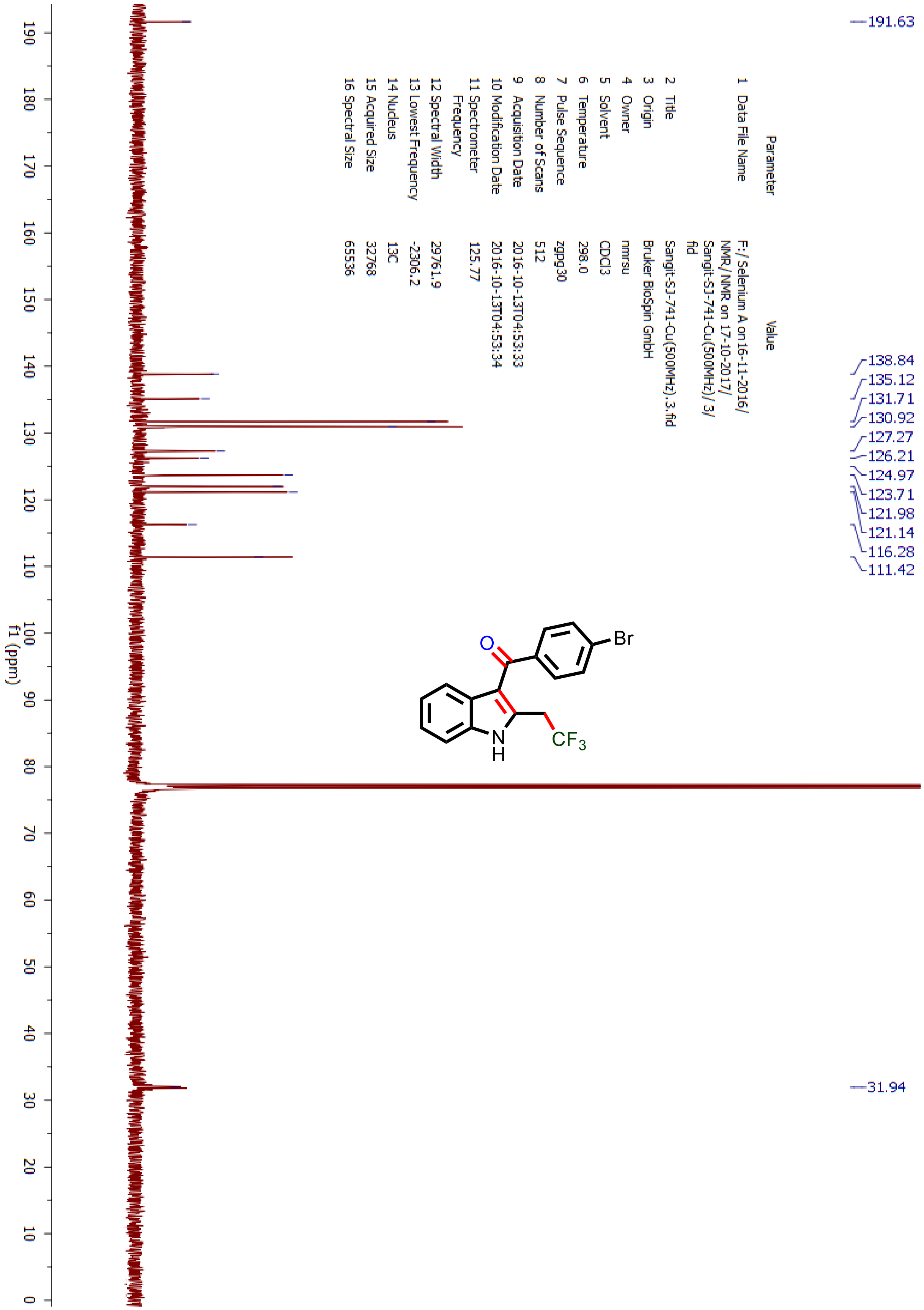

^{19}F NMR of (4-Bromophenyl)(1-tosyl-2-(2,2,2-trifluoroethyl)-1H-indol-3-yl)methanone (6c)

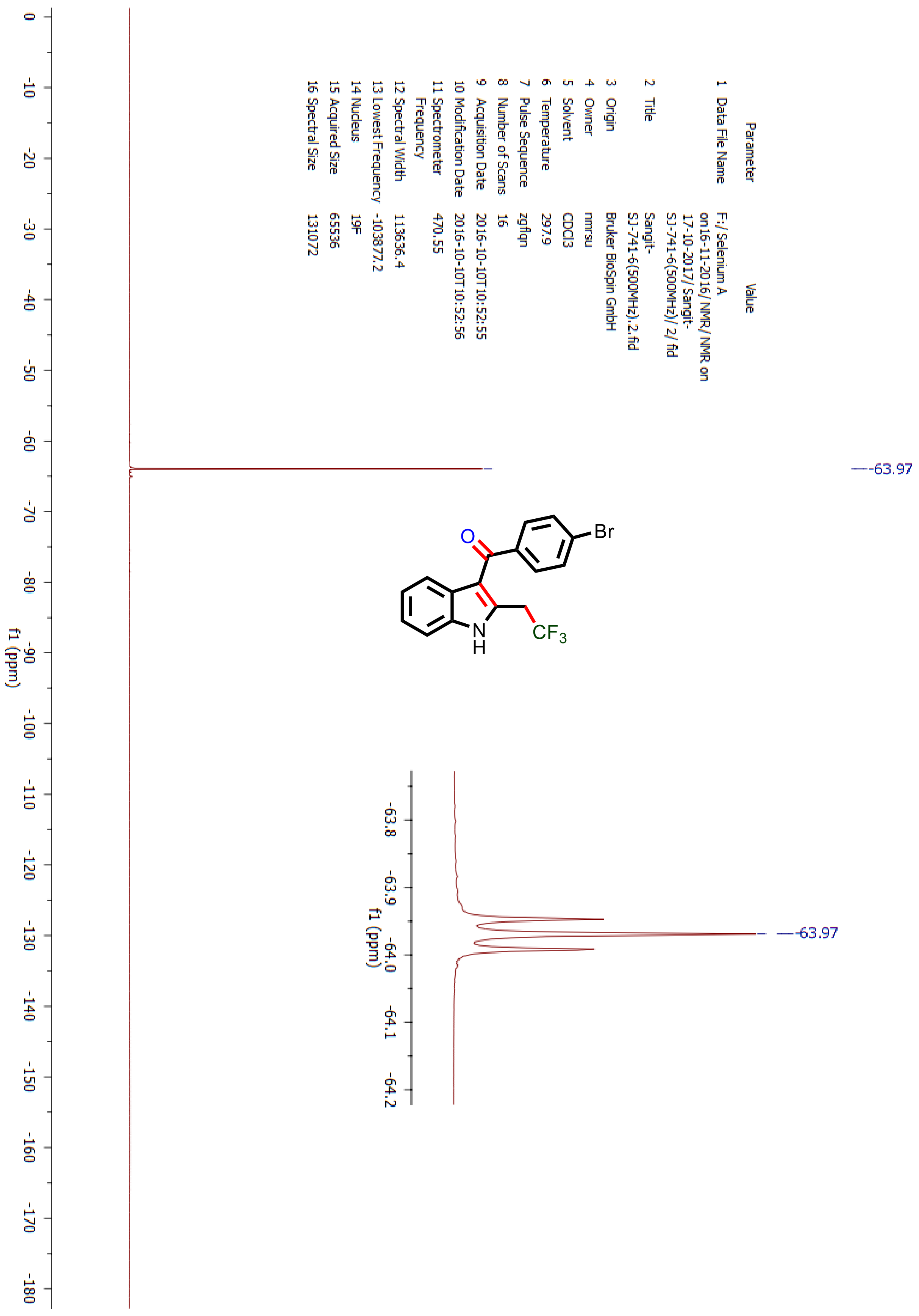

HRMS of (4-Bromophenyl)(1-tosyl-2-(2,2,2-trifluoroethyl)-1H-indol-3-yl)methanone (6c)

Display Report

Analysis Info

		Acquisition Date	10/19/2016 12:13:34 PM
Analysis Name	D:\Data\user data\2016\october 2016\19-10-2016\Dr.S.Kumar-SJ-741_1-C,7_01_7753.d		
Method	hrlcms_pos_mid_tunemix.m	Operator	DIMPLE
Sample Name	Dr.S.Kumar-SJ-741	Instrument	micrOTOF-Q II 10330
Comment			

Acquisition Parameter

Source Type	ESI	Ion Polarity	Positive	Set Nebulizer	0.3 Bar
Focus	Active	Set Capillary	4500 V	Set Dry Heater	200 °C
Scan Begin	50 m/z	Set End Plate Offset	-500 V	Set Dry Gas	4.0 l/min
Scan End	3000 m/z	Set Collision Cell RF	450.0 Vpp	Set Divert Valve	Waste

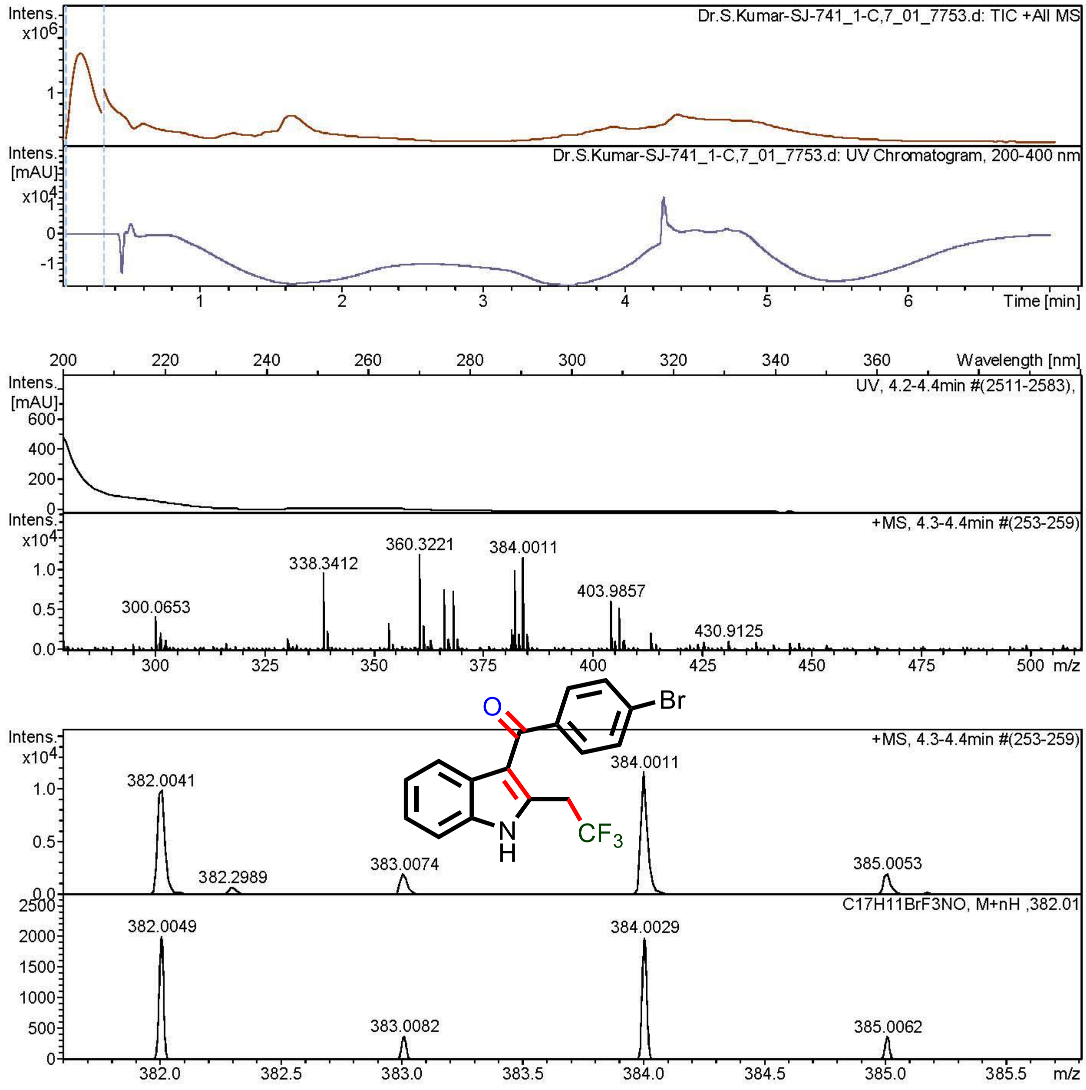

Bruker Compass DataAnalysis 4.0 printed: 10/19/2016 2:58:16 PM Page 1 of 1

^1H NMR of (4-methoxyphenyl)(2-(2,2,2-trifluoroethyl)-1H-indol-3-yl)methanone (6d)

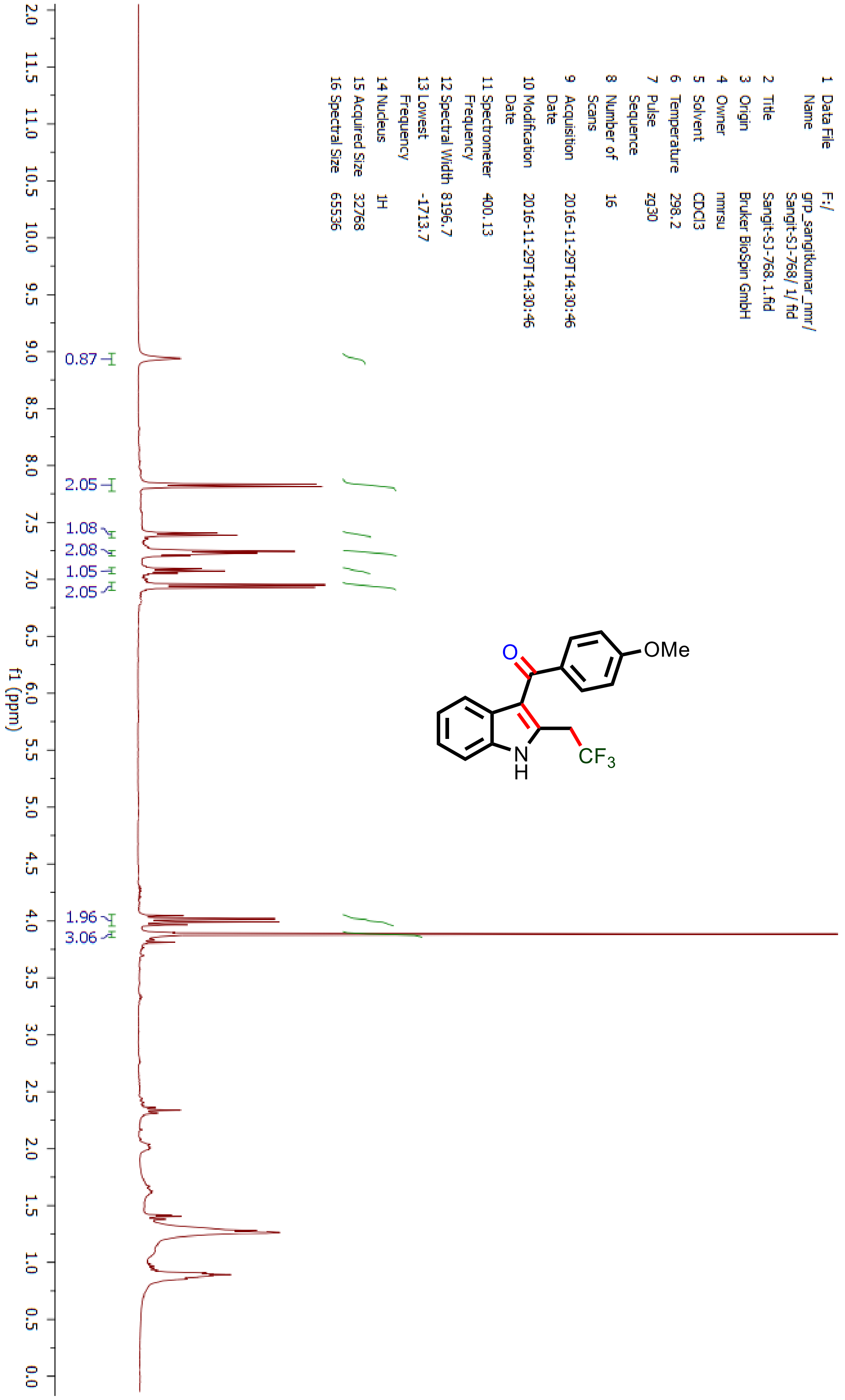

^{13}C NMR of (4-methoxyphenyl)(2-(2,2,2-trifluoroethyl)-1H-indol-3-yl)methanone (6d)

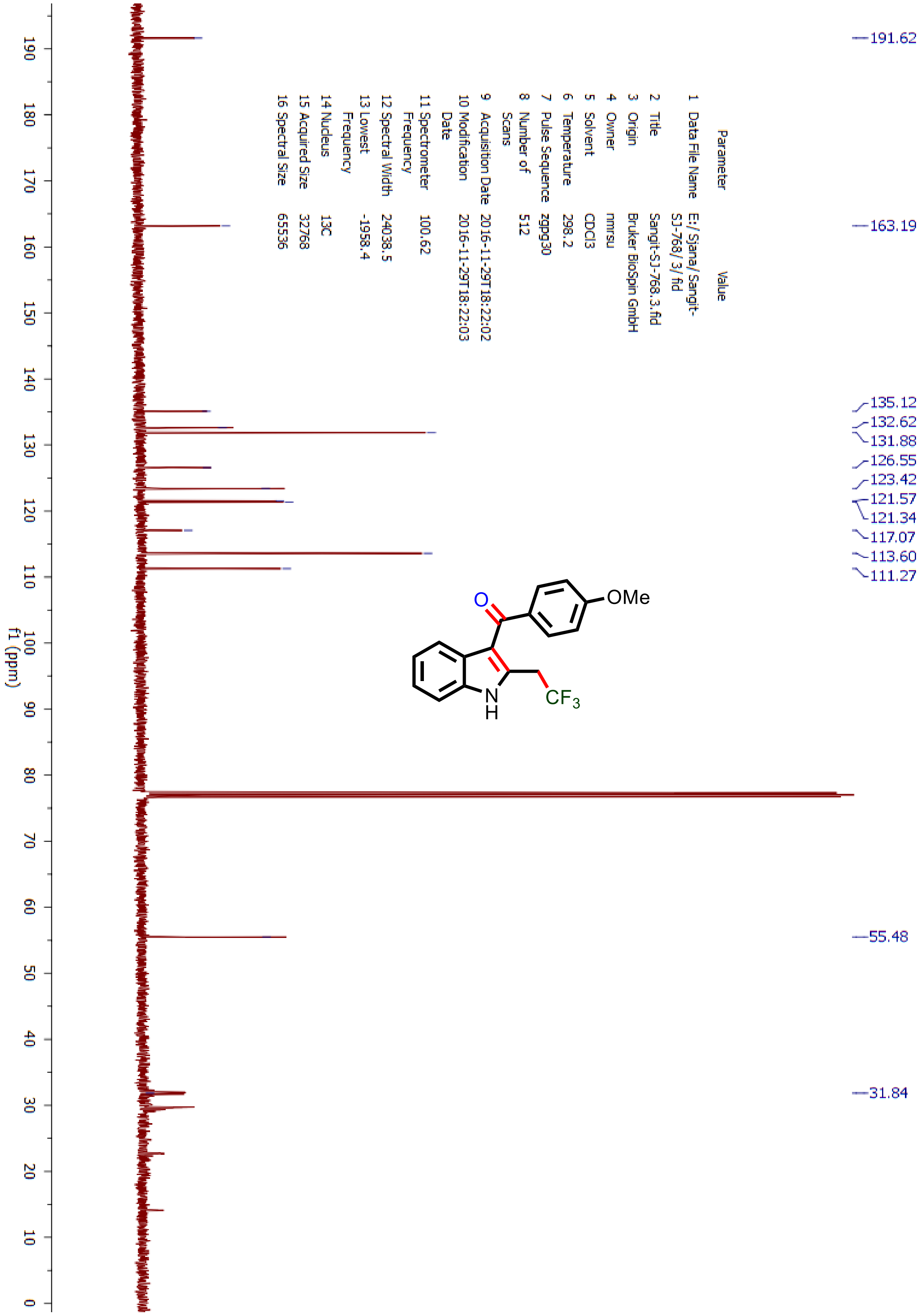

^{19}F NMR of (4-methoxyphenyl)(2-(2,2,2-trifluoroethyl)-1H-indol-3-yl)methanone (6d)

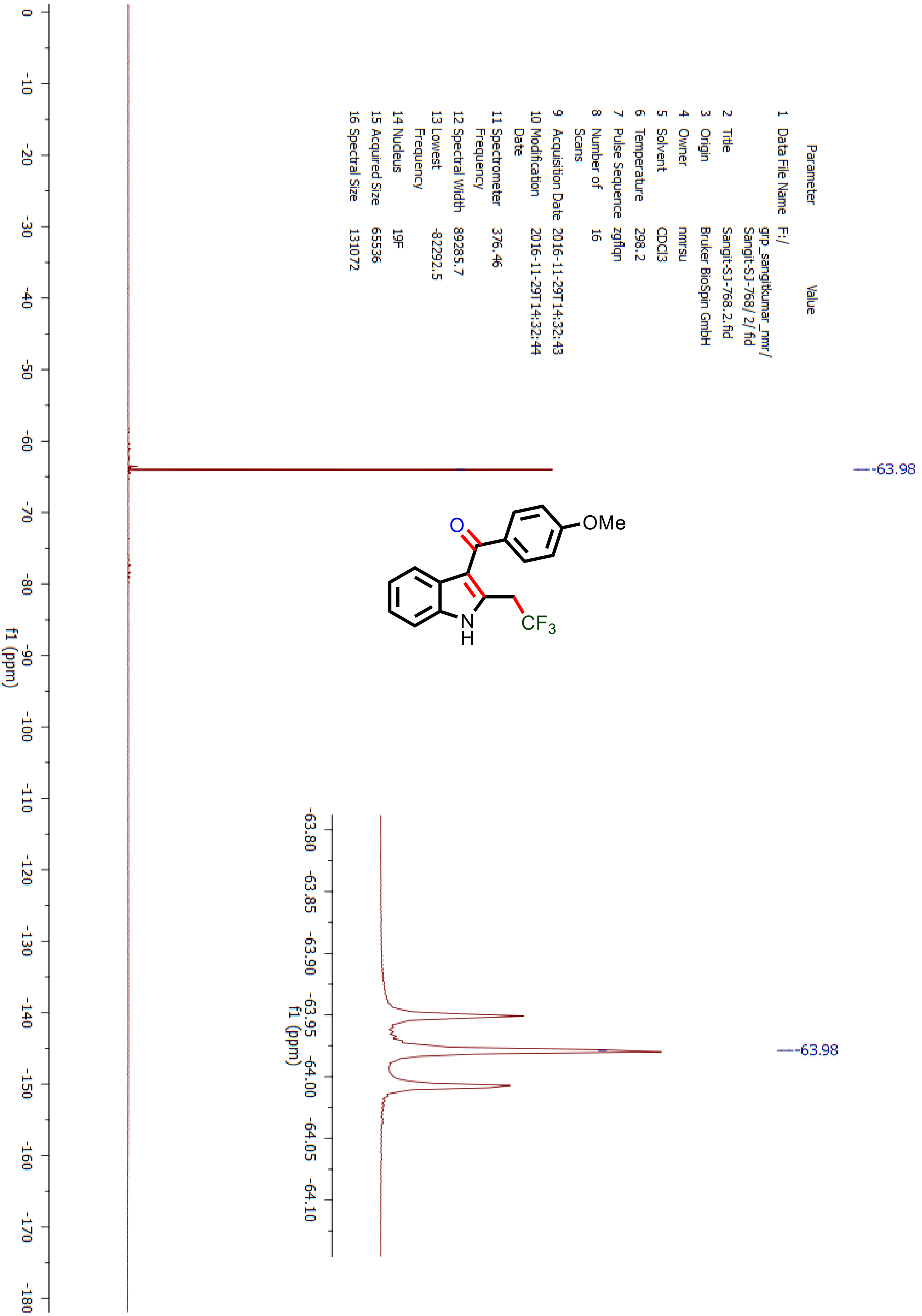

Parameter	Value
1 Data File Name	F:/grp_sangitkumar_nmr/Sangit-SJ-768/2/fid
2 Title	Sangit-SJ-768.2.fid
3 Origin	Bruker BioSpin GmbH
4 Owner	nmrsu
5 Solvent	CDCl3
6 Temperature	298.2
7 Pulse Sequence	zgflqn
8 Number of Scans	16
9 Acquisition Date	2016-11-29T14:32:43
10 Modification Date	2016-11-29T14:32:44
11 Spectrometer Frequency	376.46
12 Spectral Width	89285.7
13 Lowest Frequency	-82292.5
14 Nucleus	19F
15 Acquired Size	65536
16 Spectral Size	131072

HRMS of (4-methoxyphenyl)(2-(2,2,2-trifluoroethyl)-1H-indol-3-yl)methanone (6d)

Display Report

Analysis Info

Analysis Name	D:\Data\user data\2016\DEC-2016\06 DEC 2016\Dr.S.kumar-SJ-768_1-A,2_01_55.d	Acquisition Date	12/6/2016 11:46:16 AM
Method	HRLCMS-20 Sept.m	Operator	RUCHI SHRIVASTAVA
Sample Name	Dr.S.kumar-SJ-768	Instrument	micrOTOF-Q II 10330
Comment			

Acquisition Parameter

Source Type	ESI	Ion Polarity	Positive	Set Nebulizer	1.2 Bar
Focus	Active	Set Capillary	4500 V	Set Dry Heater	200 °C
Scan Begin	50 m/z	Set End Plate Offset	-500 V	Set Dry Gas	7.0 l/min
Scan End	3000 m/z	Set Collision Cell RF	130.0 Vpp	Set Divert Valve	Waste

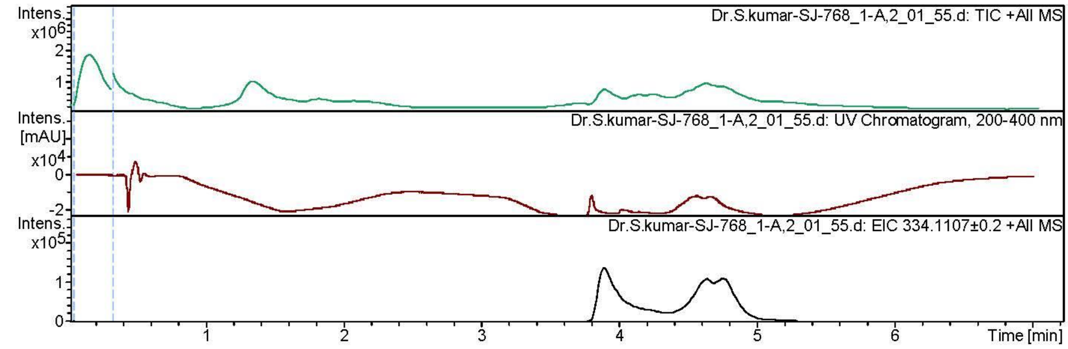

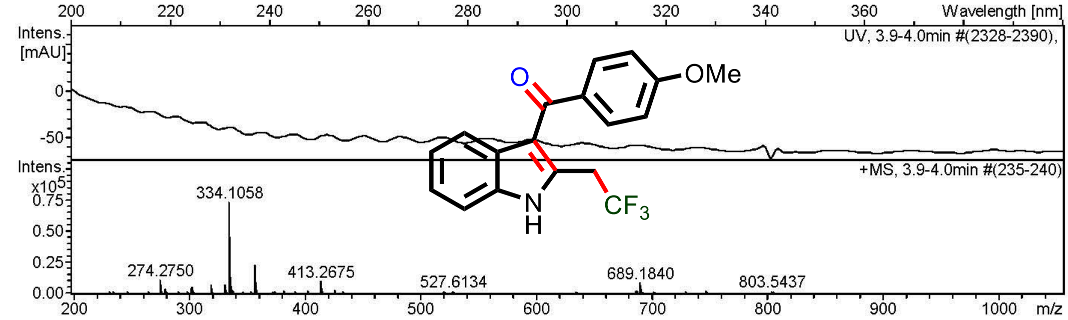

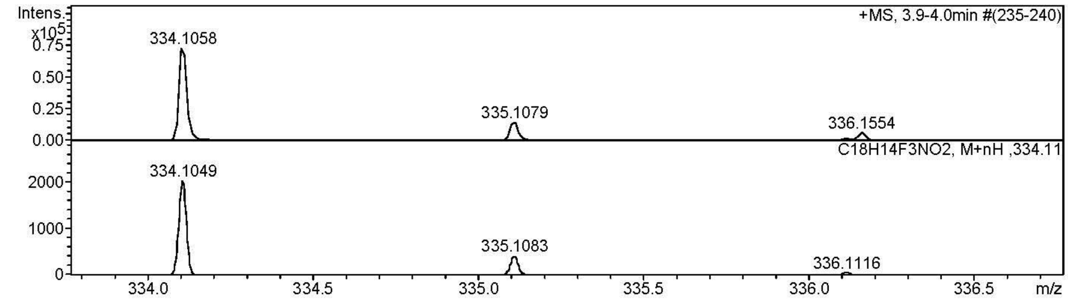

^{1}H NMR of Thiophen-3-yl(1-tosyl-2-(2,2,2-trifluoroethyl)-1H-indol-3-yl)methanone (6e)

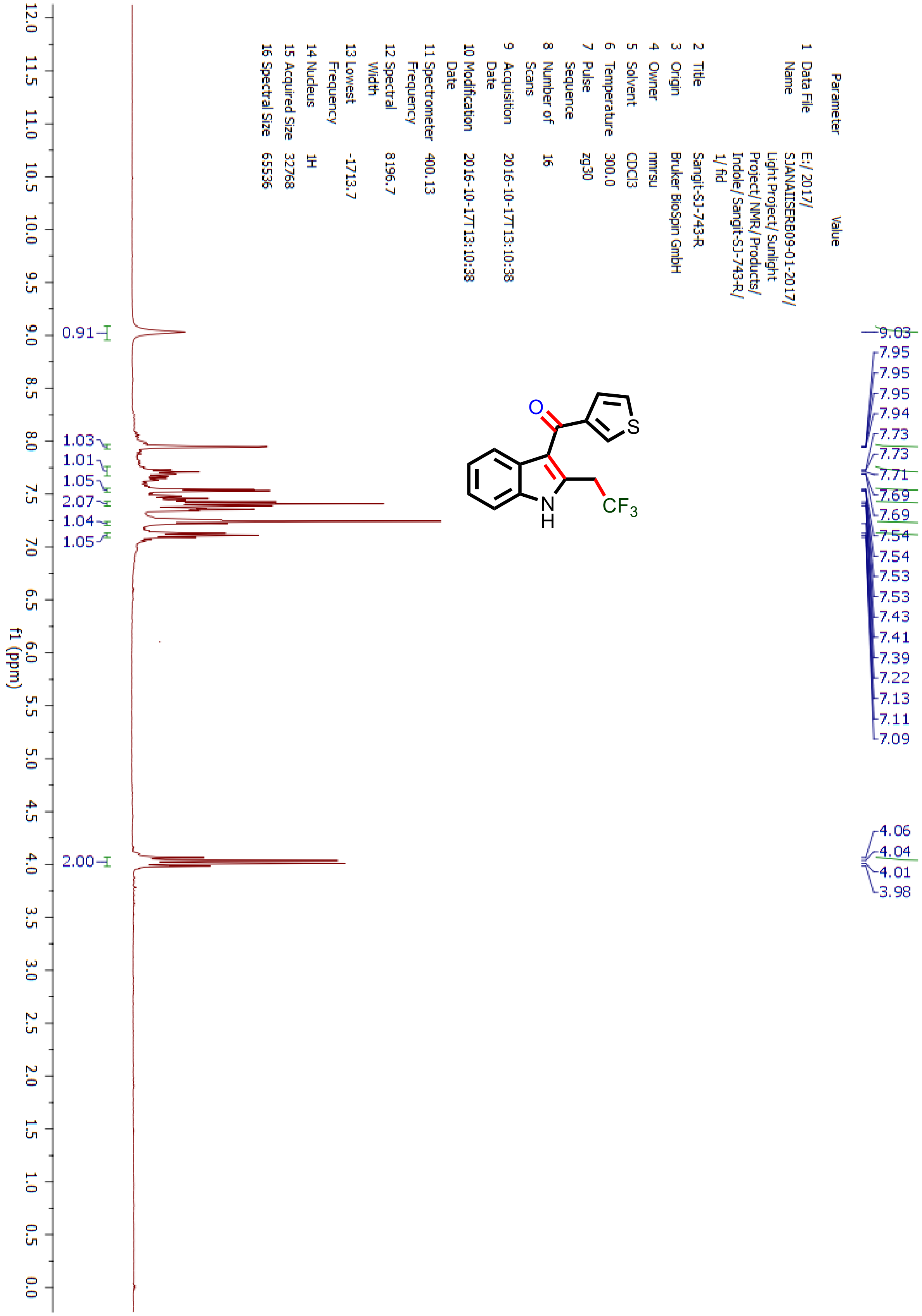

^{13}C NMR of Thiophen-3-yl(1-tosyl-2-(2,2,2-trifluoroethyl)-1H-indol-3-yl)methanone (6e)

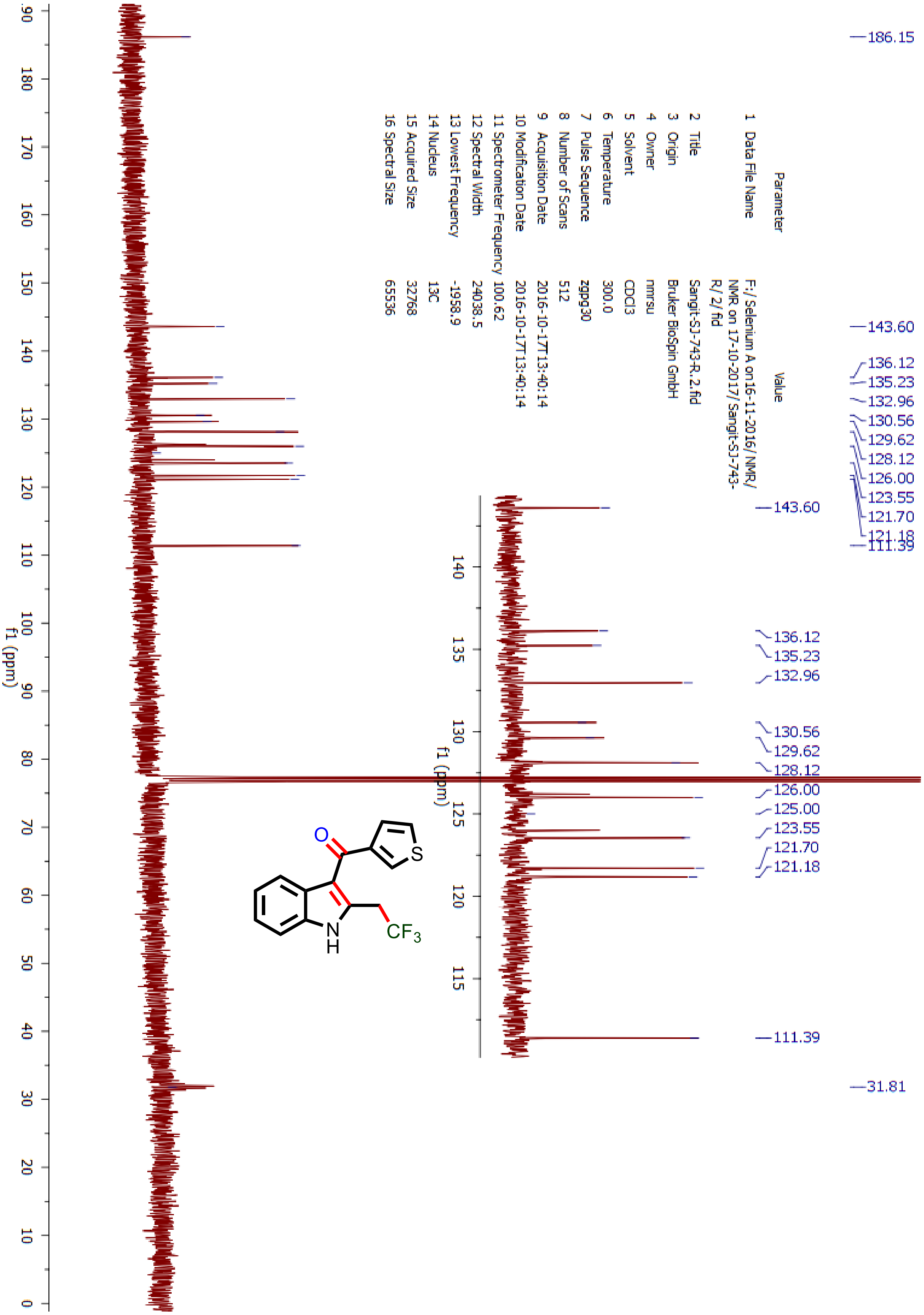

^{19}F NMR of Thiophen-3-yl(1-tosyl-2-(2,2,2-trifluoroethyl)-1H-indol-3-yl)methanone (6e)

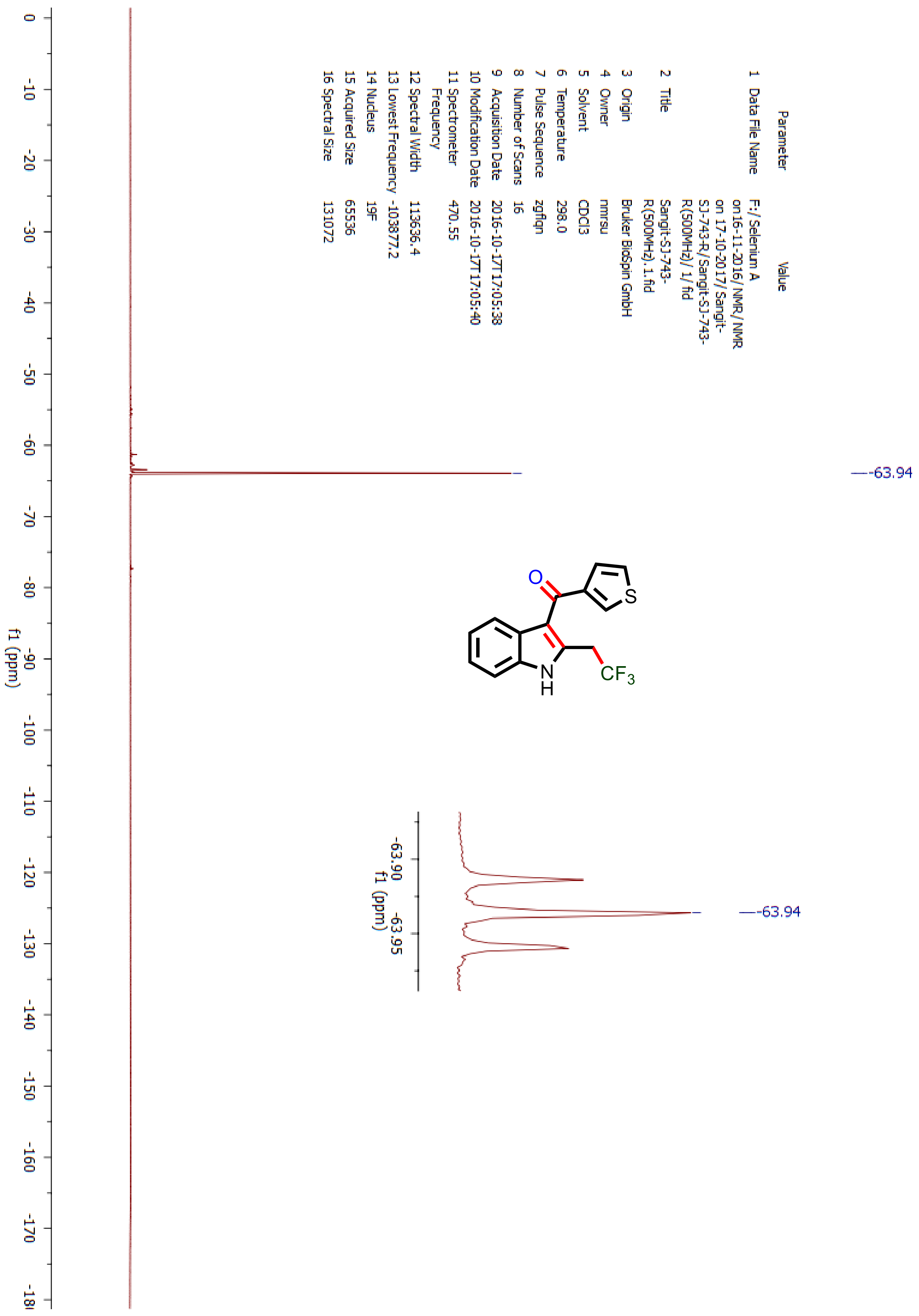

HRMS of Thiophen-3-yl(1-tosyl-2-(2,2,2-trifluoroethyl)-1H-indol-3-yl)methanone (6e)

Display Report

Analysis Info

		Acquisition Date	10/19/2016 12:21:44 PM
Analysis Name	D:\Data\user data\2016\october 2016\19-10-2016\Dr.S.Kumar-SJ-743_1-C,8_01_7754.d		
Method	hrlcms_pos_mid_tunemix.m	Operator	DIMPLE
Sample Name	Dr.S.Kumar-SJ-743	Instrument	micrOTOF-Q II 10330
Comment			

Acquisition Parameter

Source Type	ESI	Ion Polarity	Positive	Set Nebulizer	0.3 Bar
Focus	Active	Set Capillary	4500 V	Set Dry Heater	200 °C
Scan Begin	50 m/z	Set End Plate Offset	-500 V	Set Dry Gas	4.0 l/min
Scan End	3000 m/z	Set Collision Cell RF	450.0 Vpp	Set Divert Valve	Waste

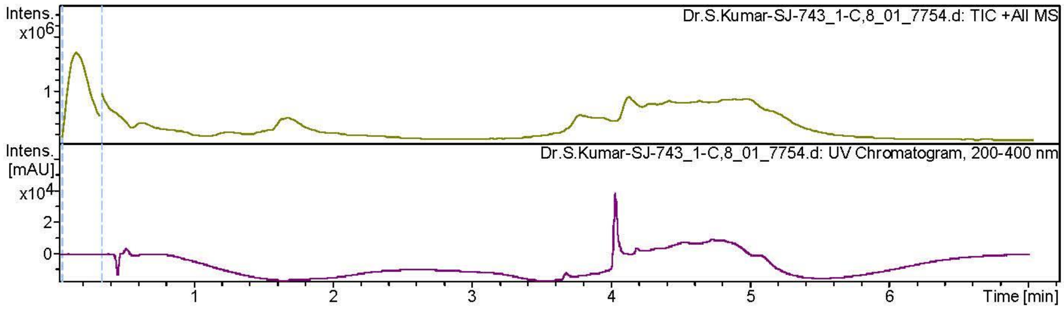

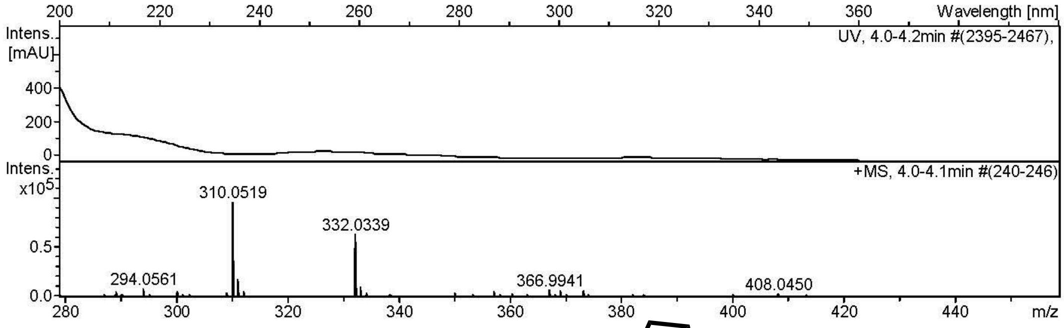

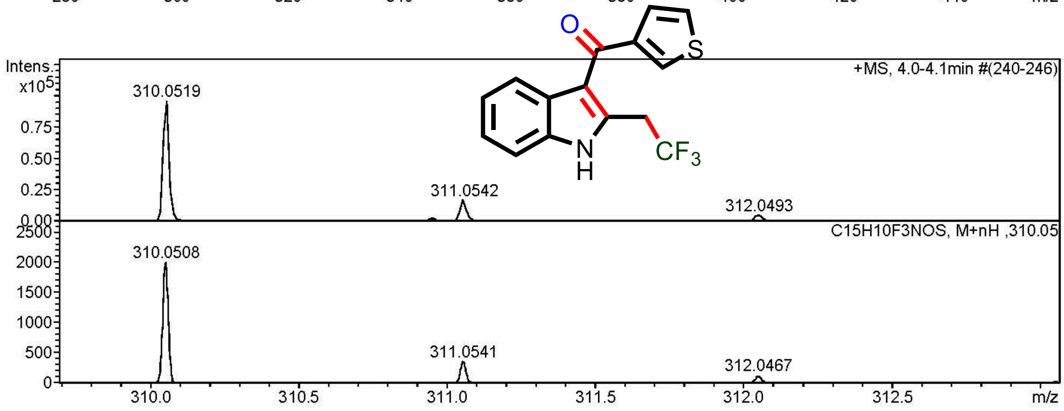

^{1}H NMR of Naphthalen-1-yl(2-(2,2,2-trifluoroethyl)-1H-indol-3-yl)methanone (6f)

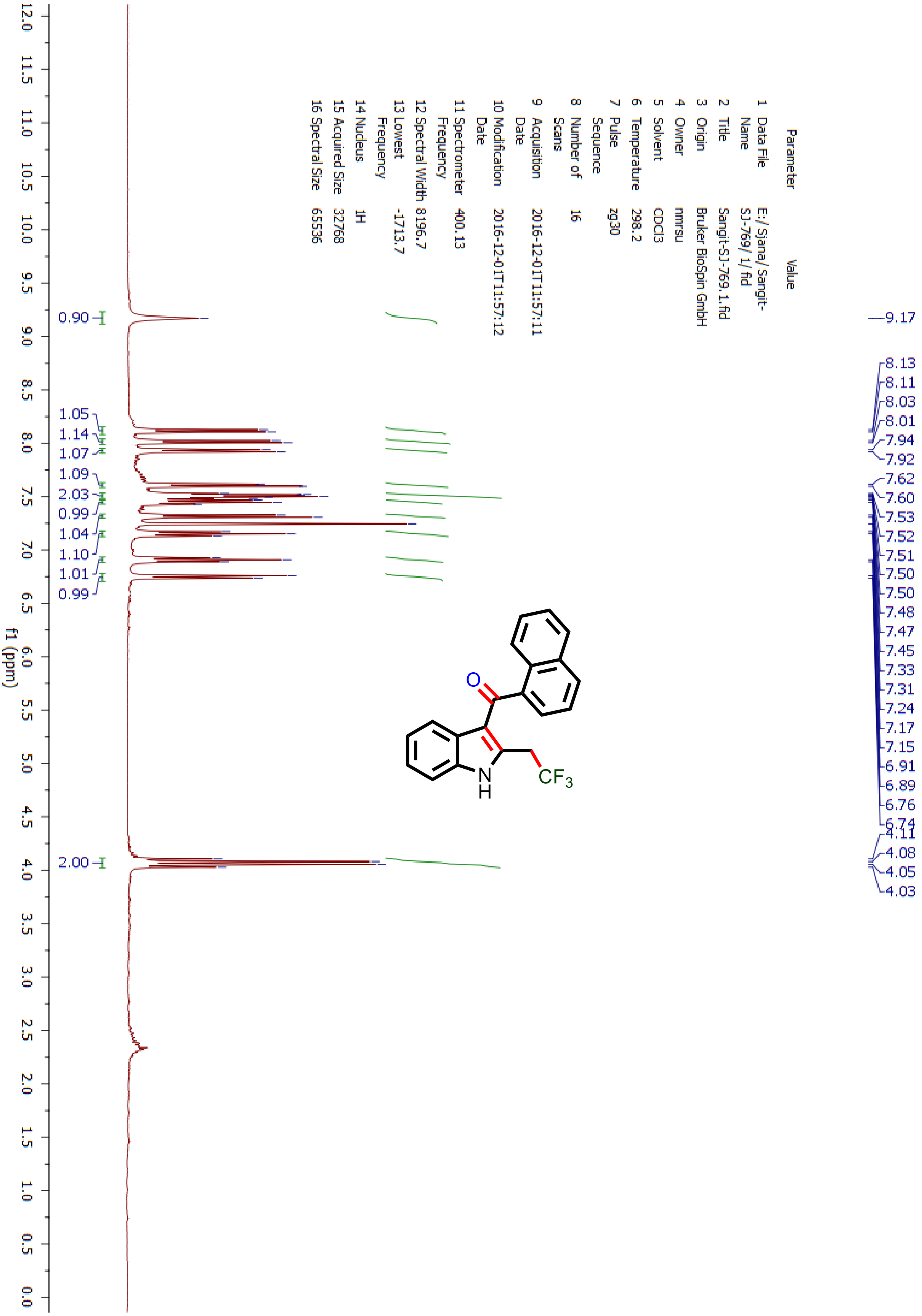

^{13}C NMR of Naphthalen-1-yl(2-(2,2,2-trifluoroethyl)-1H-indol-3-yl)methanone (6f)

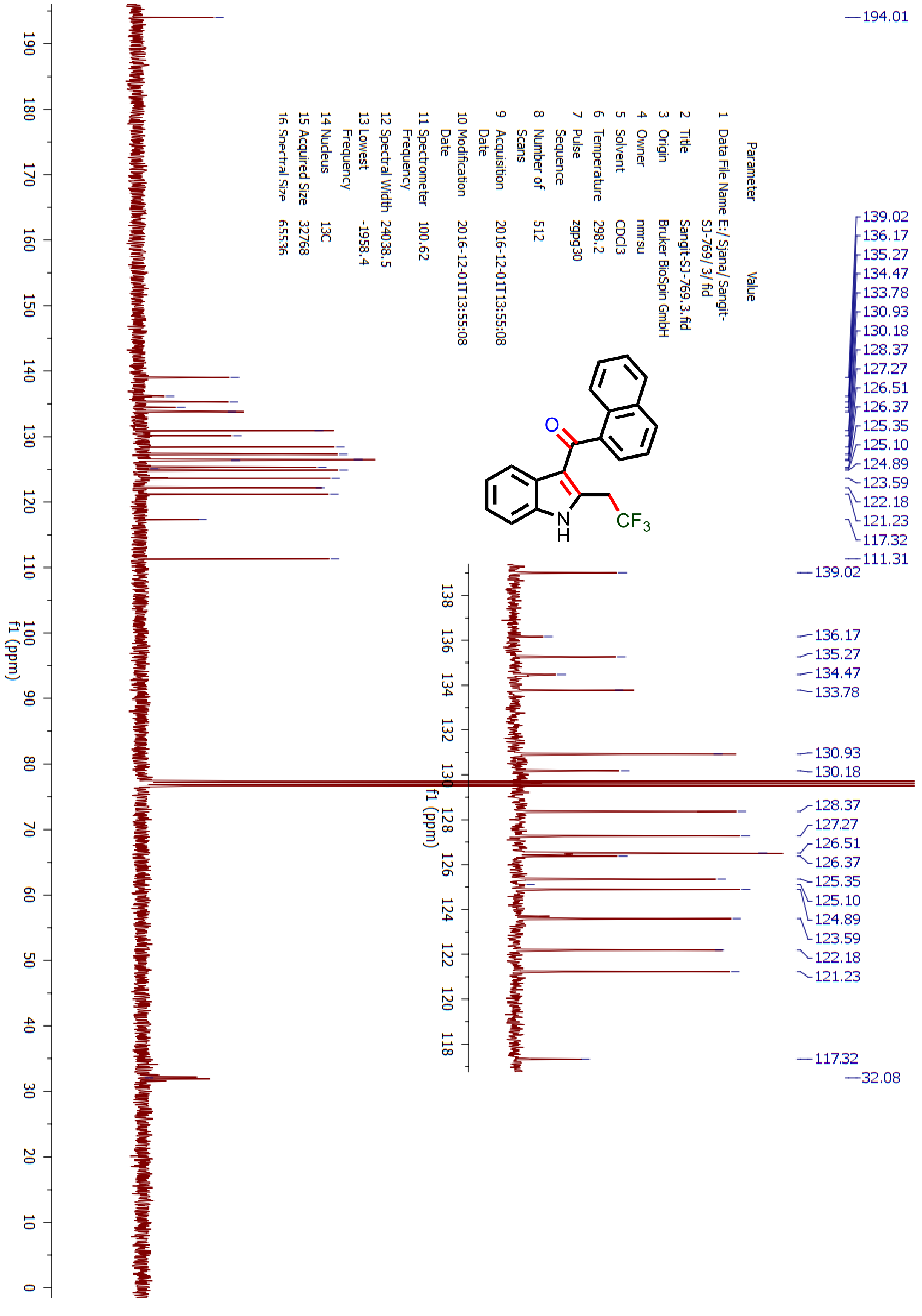

^{19}F NMR of Naphthalen-1-yl(2-(2,2,2-trifluoroethyl)-1H-indol-3-yl)methanone (6f)

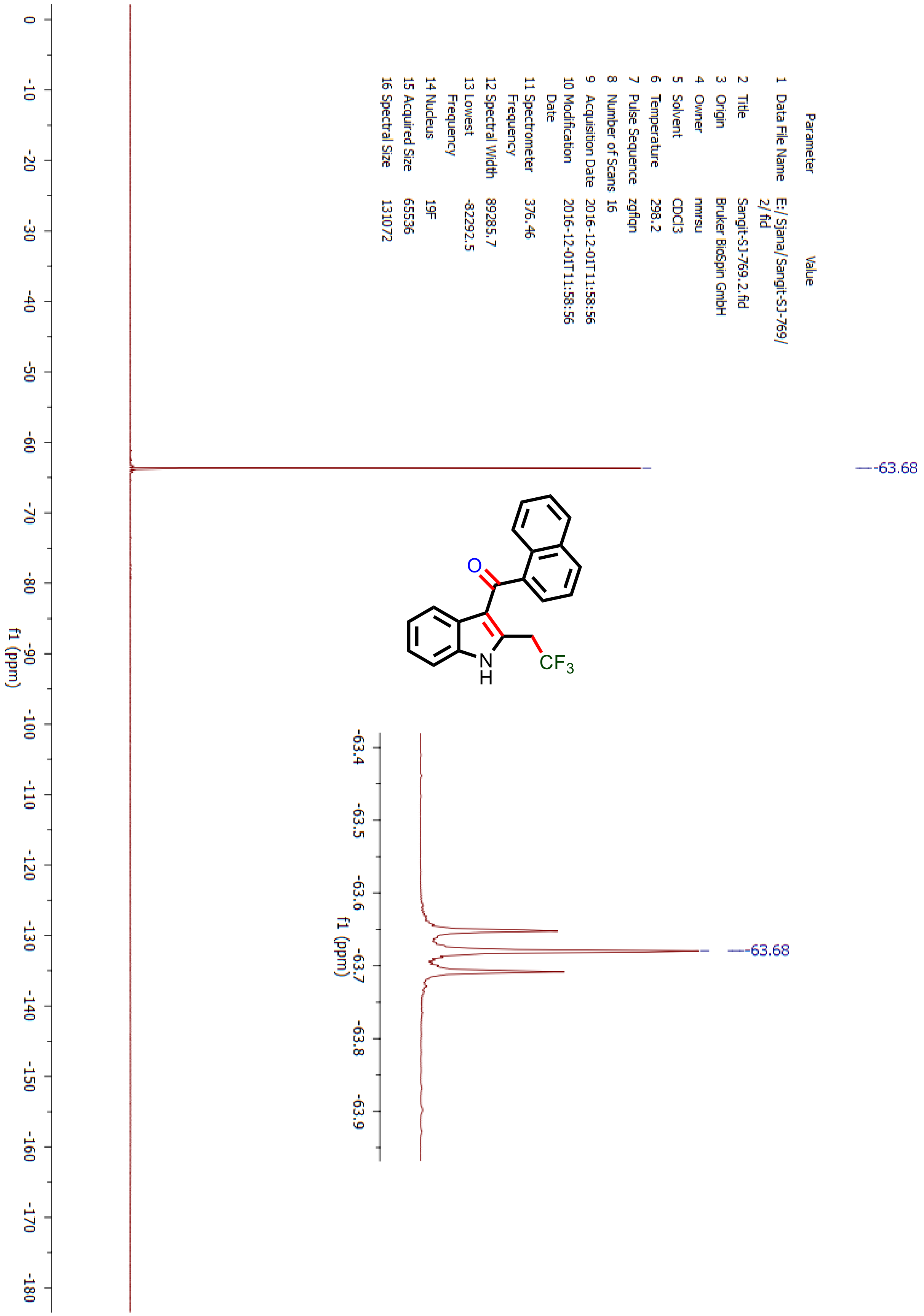

HRMS of Naphthalen-1-yl(2-(2,2,2-trifluoroethyl)-1H-indol-3-yl)methanone (6f)

Display Report

Analysis Info

		Acquisition Date	12/6/2016 11:54:26 AM
Analysis Name	D:\Data\user data\2016\DEC-2016\06 DEC 2016\Dr.S.kumar-SJ-769_1-A,3_01_56.d		
Method	HRLCMS-20 Sept.m	Operator	RUCHI SHRIVASTAVA
Sample Name	Dr.S.kumar-SJ-769	Instrument	micrOTOF-Q II 10330
Comment			

Acquisition Parameter

Source Type	ESI	Ion Polarity	Positive	Set Nebulizer	1.2 Bar
Focus	Active	Set Capillary	4500 V	Set Dry Heater	200 °C
Scan Begin	50 m/z	Set End Plate Offset	-500 V	Set Dry Gas	7.0 l/min
Scan End	3000 m/z	Set Collision Cell RF	130.0 Vpp	Set Divert Valve	Waste

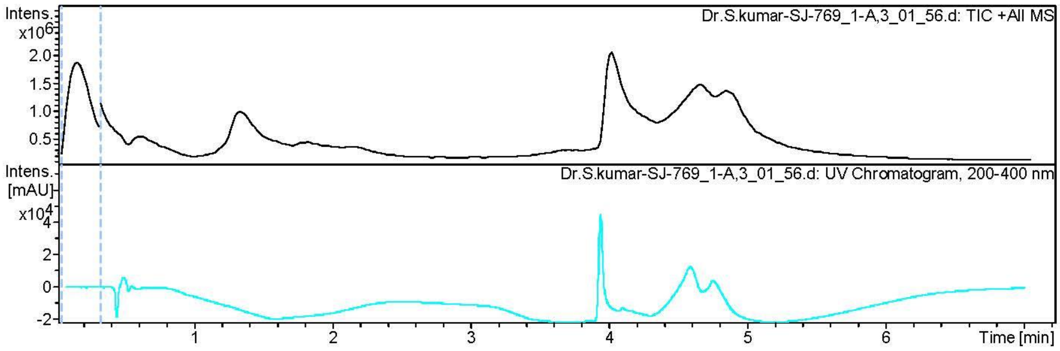

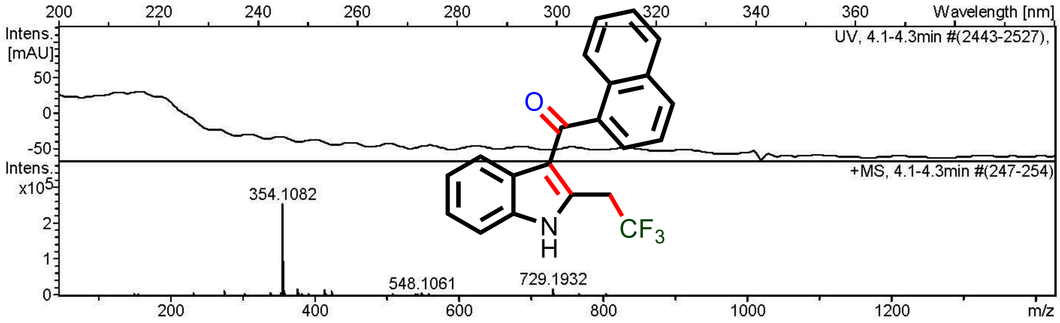

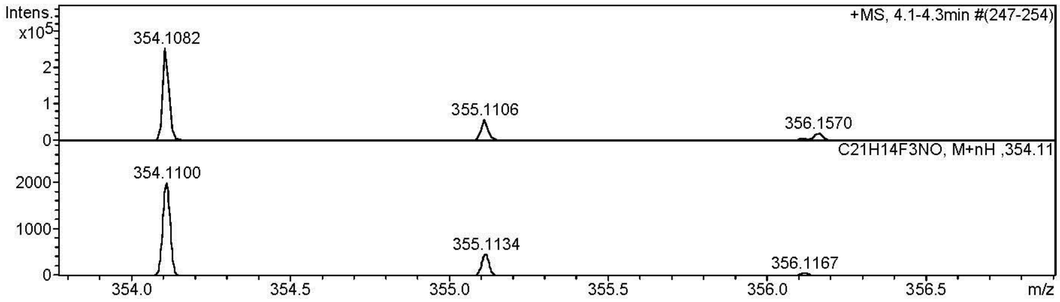

^{1}H NMR of (5-Fluoro-2-(2,2,2-trifluoroethyl)-1H-indol-3-yl)(phenyl)methanone (6g)

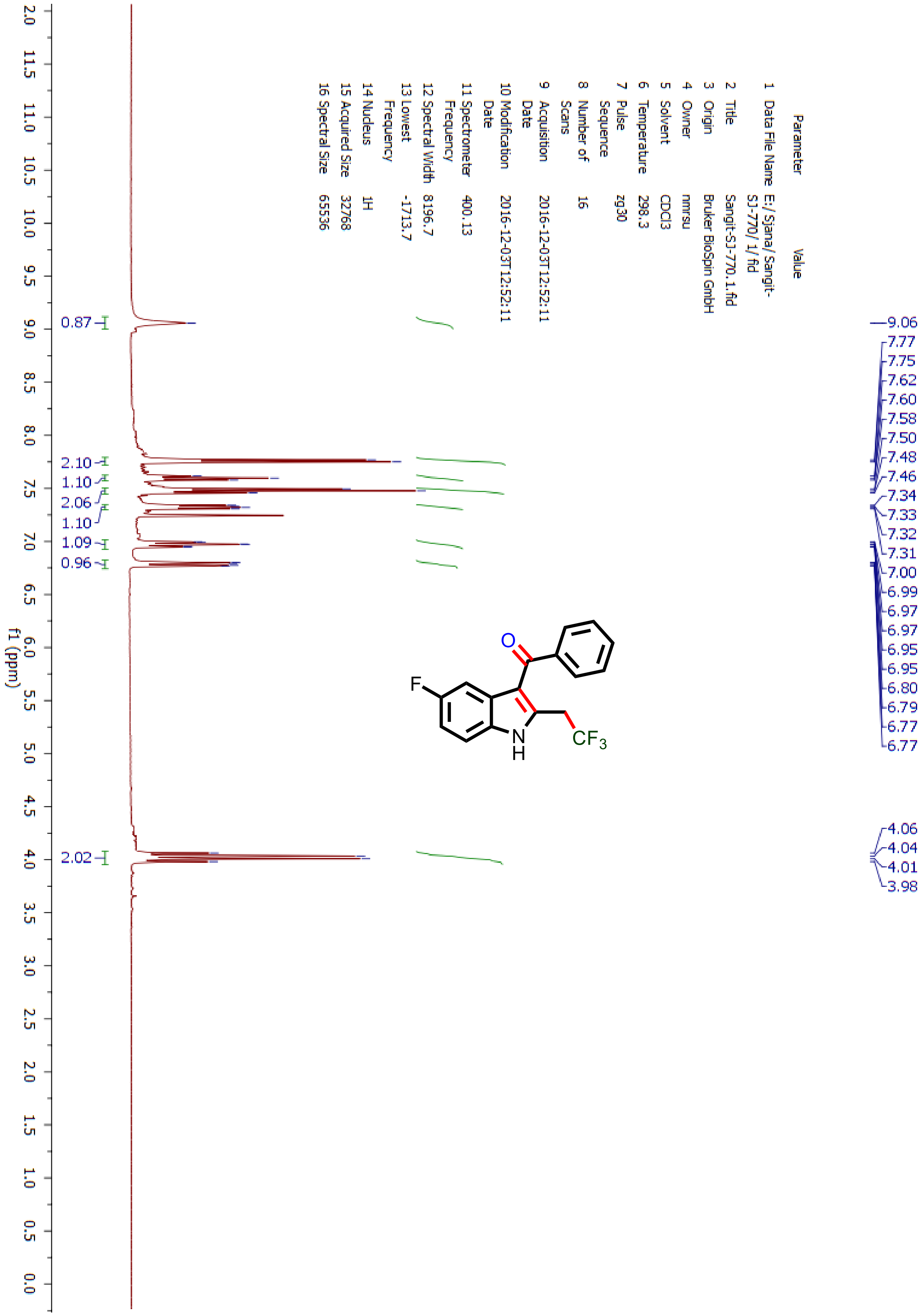

^{13}C NMR of (5-Fluoro-2-(2,2,2-trifluoroethyl)-1H-indol-3-yl)(phenyl)methanone (6g)

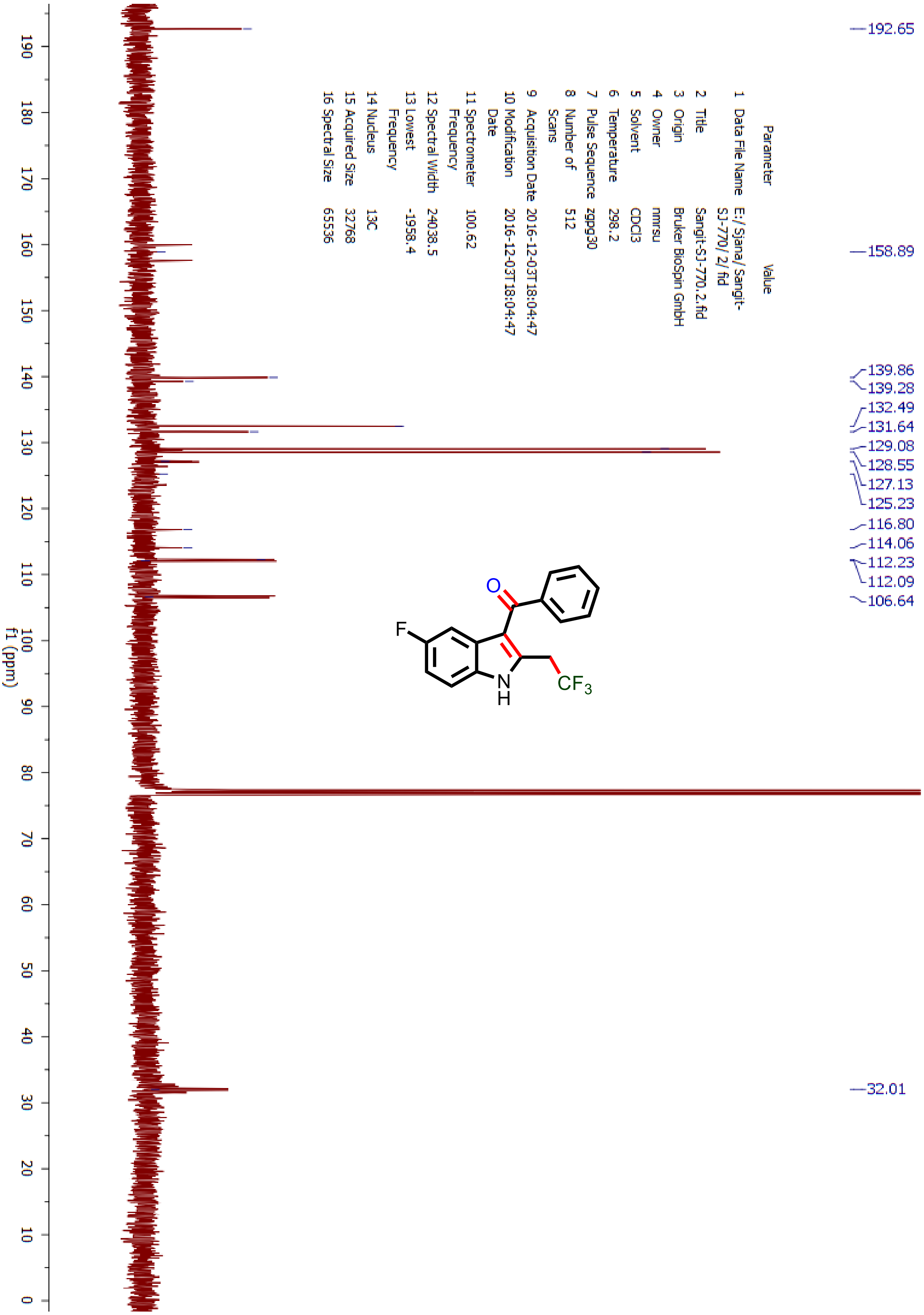

^{19}F NMR of (5-Fluoro-2-(2,2,2-trifluoroethyl)-1H-indol-3-yl)(phenyl)methanone (6g)

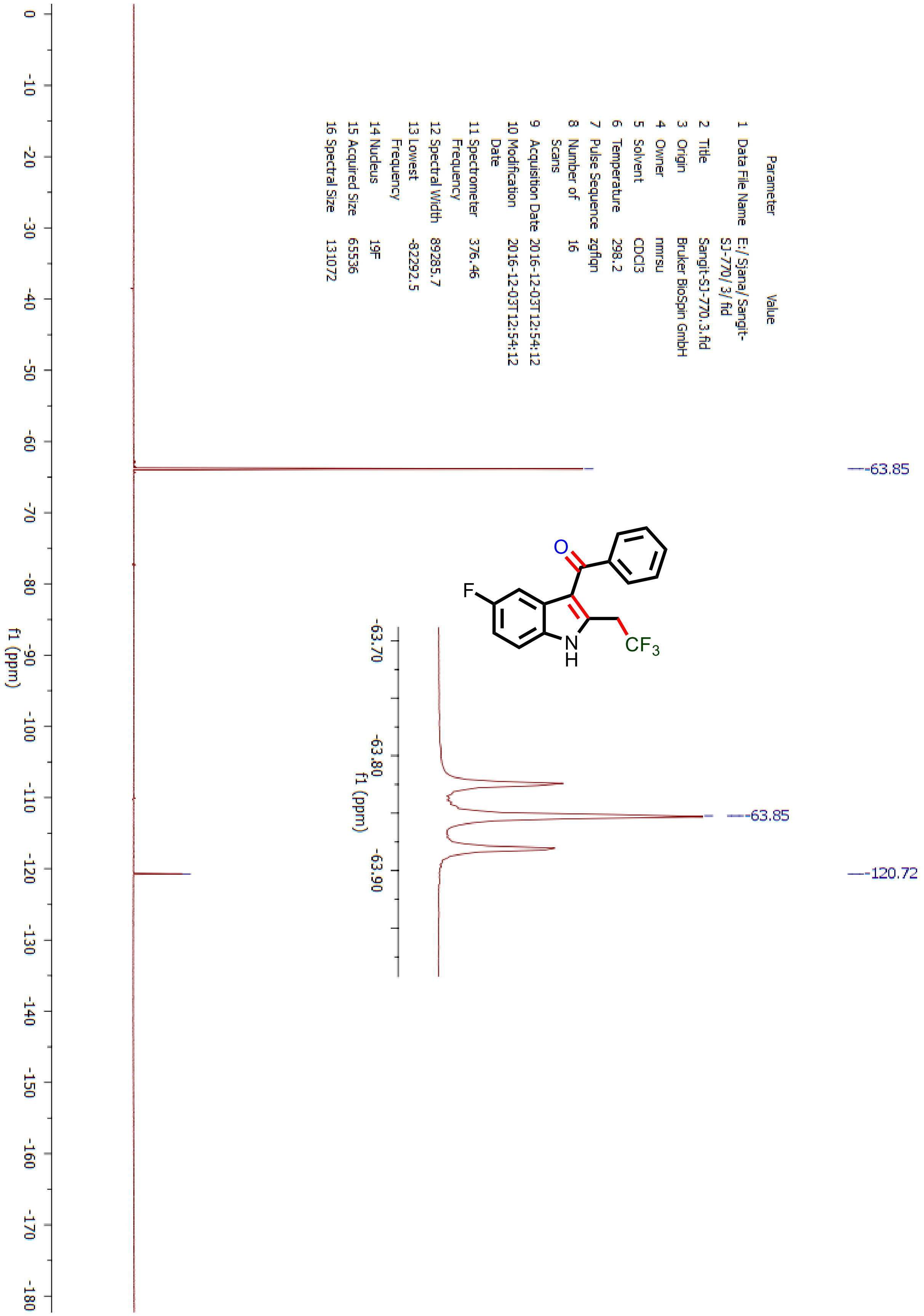

HRMS of (5-Fluoro-2-(2,2,2-trifluoroethyl)-1H-indol-3-yl)(phenyl)methanone (6g)

Display Report

Analysis Info

		Acquisition Date	12/6/2016 12:02:36 PM
Analysis Name	D:\Data\user data\2016\DEC-2016\06 DEC 2016\Dr.S.kumar-SJ-770_1-A,4_01_57.d		
Method	HRLCMS-20 Sept.m	Operator	RUCHI SHRIVASTAVA
Sample Name	Dr.S.kumar-SJ-770	Instrument	micrOTOF-Q II 10330
Comment			

Acquisition Parameter

Source Type	ESI	Ion Polarity	Positive	Set Nebulizer	1.2 Bar
Focus	Active	Set Capillary	4500 V	Set Dry Heater	200 °C
Scan Begin	50 m/z	Set End Plate Offset	-500 V	Set Dry Gas	7.0 l/min
Scan End	3000 m/z	Set Collision Cell RF	130.0 Vpp	Set Divert Valve	Waste

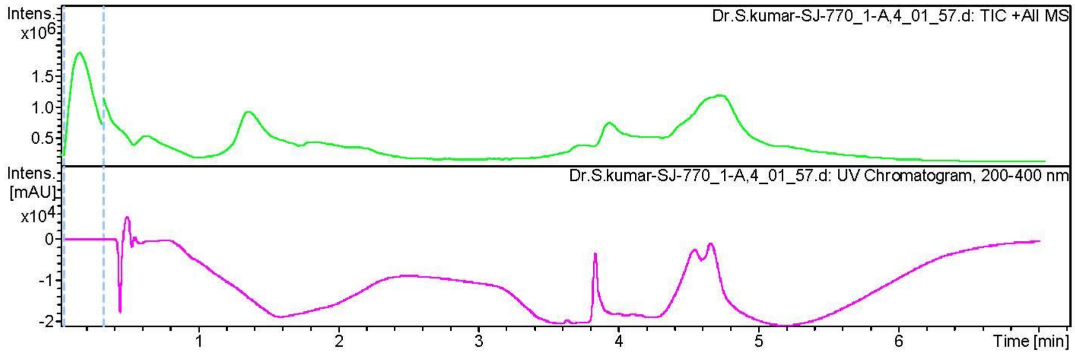

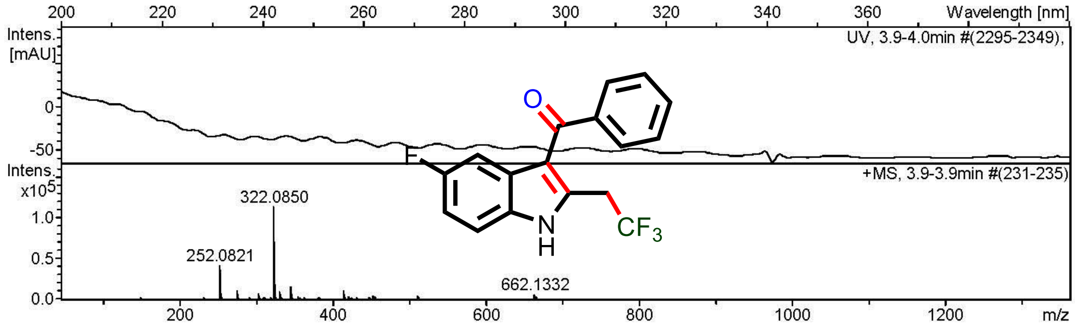

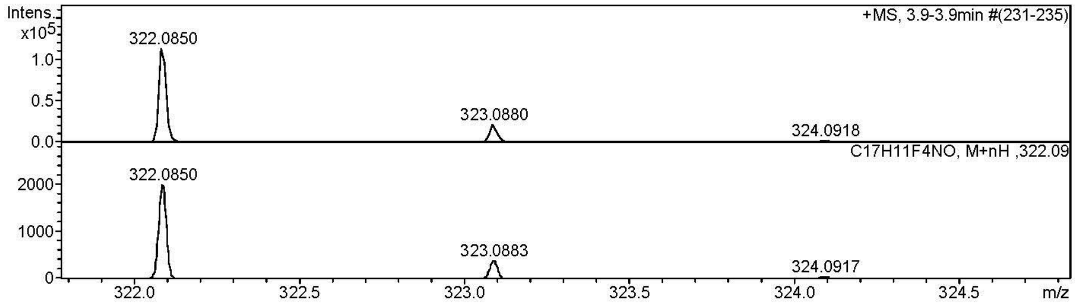

Bruker Compass DataAnalysis 4.0 printed: 12/6/2016 4:12:14 PM Page 1 of 1

^{1}H NMR of *tert*-Butyl 3-benzoyl-2-(2,2,2-trifluoroethyl)-1H-indole-1-carboxylate (6h)

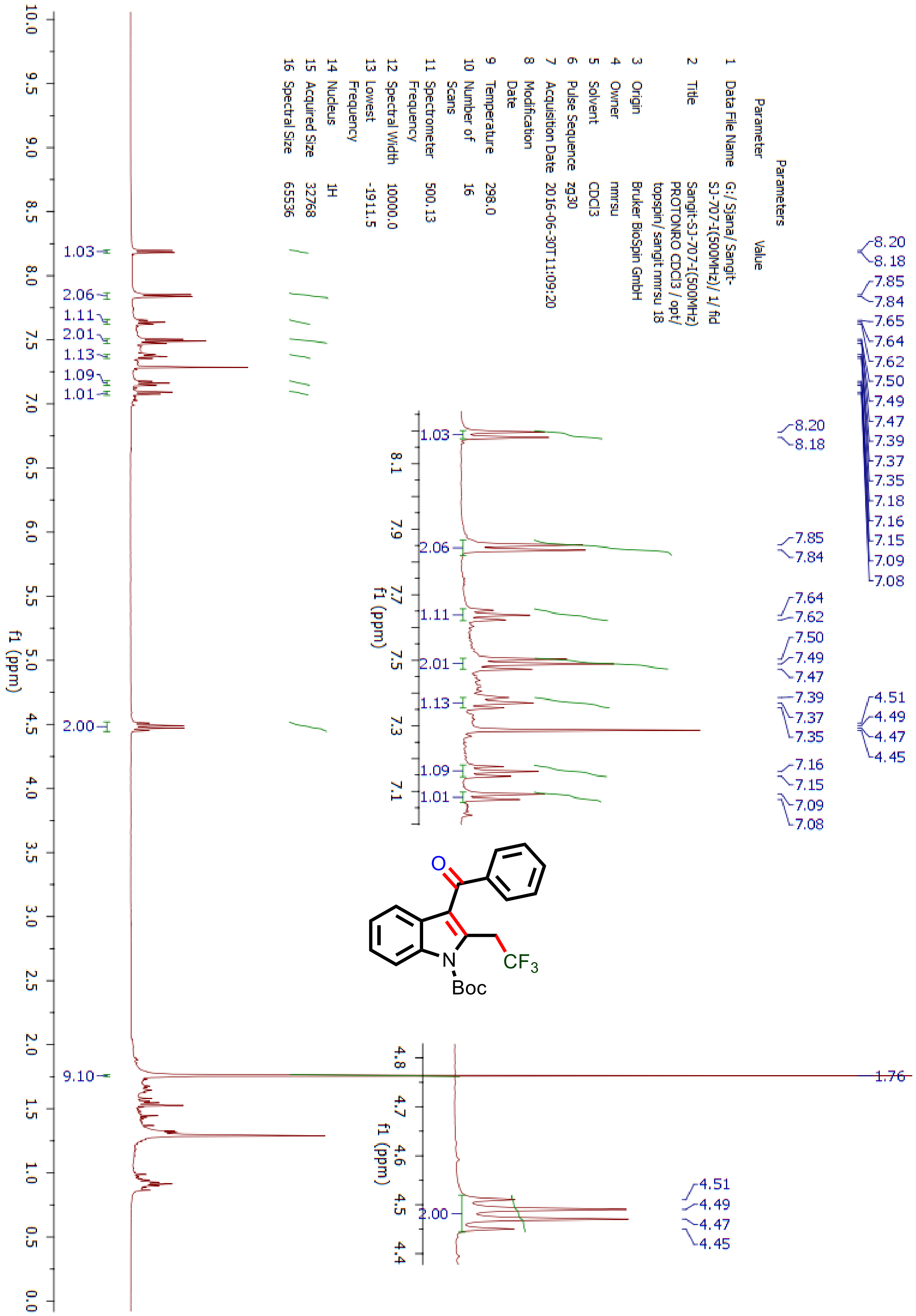

^{13}C NMR of *tert*-Butyl 3-benzoyl-2-(2,2,2-trifluoroethyl)-1H-indole-1-carboxylate (6h)

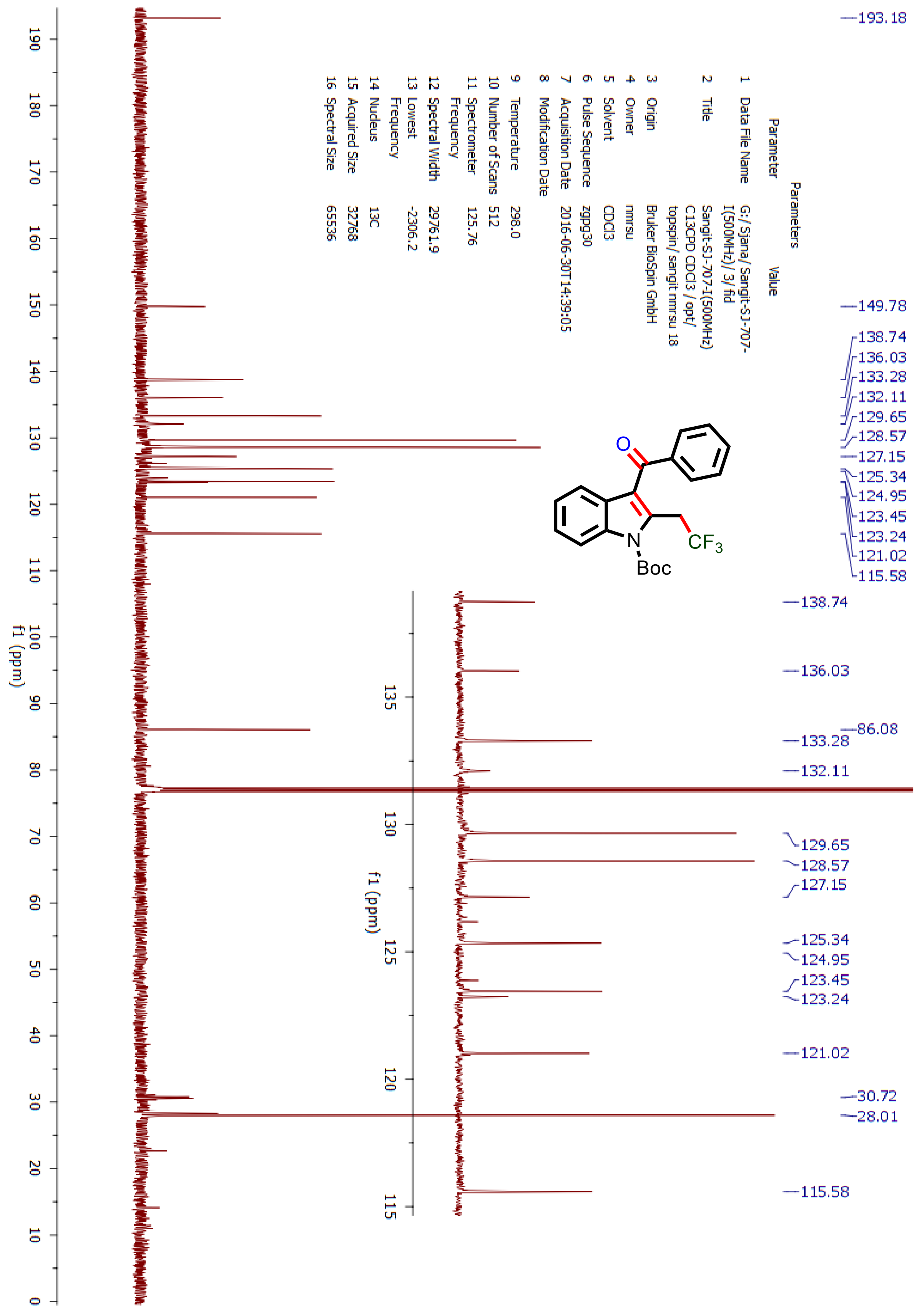

^{19}F NMR of *tert*-Butyl 3-benzoyl-2-(2,2,2-trifluoroethyl)-1H-indole-1-carboxylate (6h)

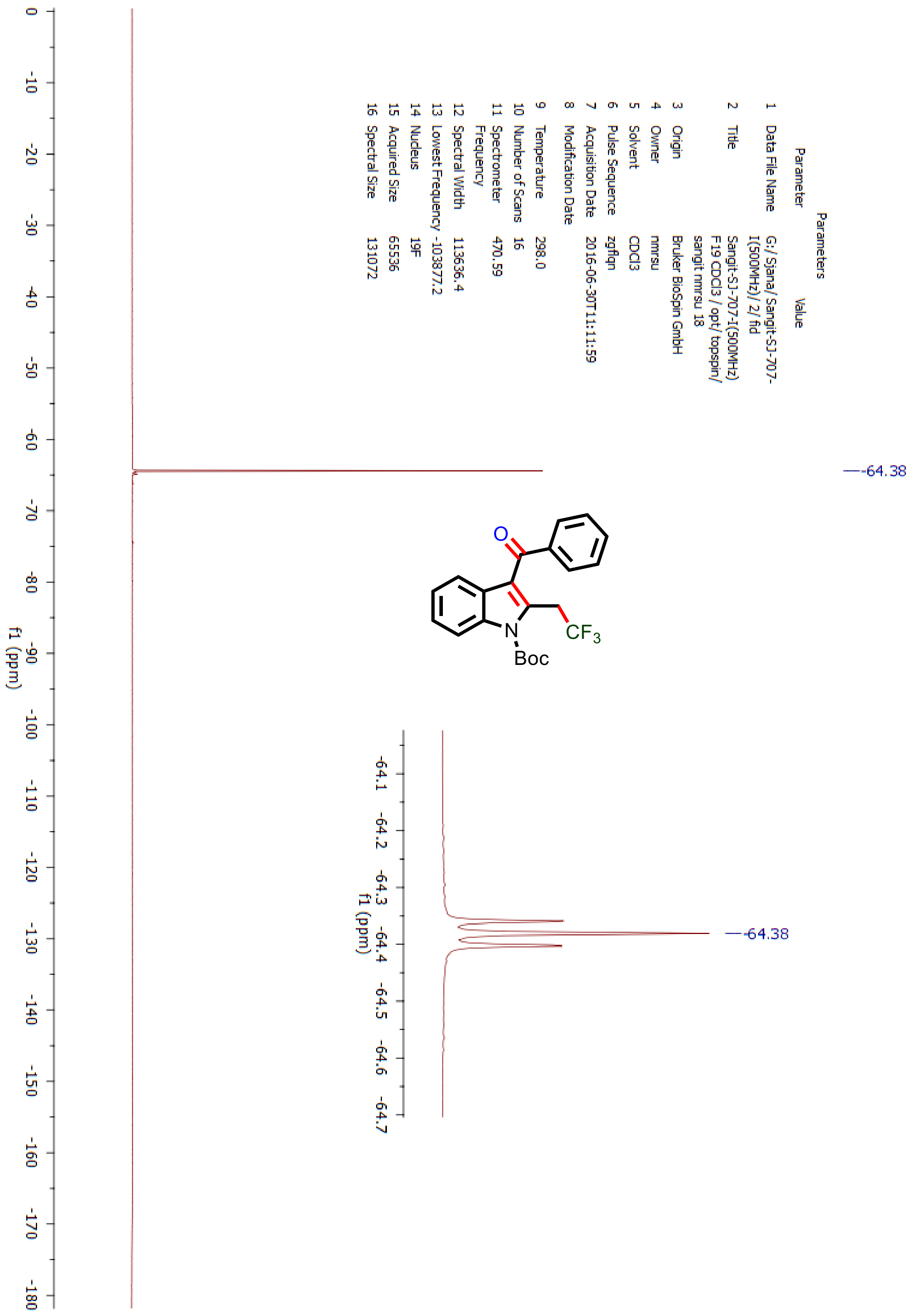

HRMS of *tert*-Butyl 3-benzoyl-2-(2,2,2-trifluoroethyl)-1H-indole-1-carboxylate (6h)

Display Report

Analysis Info

		Acquisition Date	9/8/2016 12:22:27 PM
Analysis Name	D:\Data\user data\2016\SEPT-2016\08-sep-2016\Dr S. Kumar-SJ-707-I_1-B,3_01_7329.d		
Method	hrlcms_pos_low_tunemix.m	Operator	DIMPLE
Sample Name	Dr S. Kumar-SJ-707-I	Instrument	micrOTOF-Q II 10330
Comment			

Acquisition Parameter

Source Type	ESI	Ion Polarity	Positive	Set Nebulizer	1.0 Bar
Focus	Active	Set Capillary	4500 V	Set Dry Heater	250 °C
Scan Begin	50 m/z	Set End Plate Offset	-500 V	Set Dry Gas	7.0 l/min
Scan End	3000 m/z	Set Collision Cell RF	130.0 Vpp	Set Divert Valve	Waste

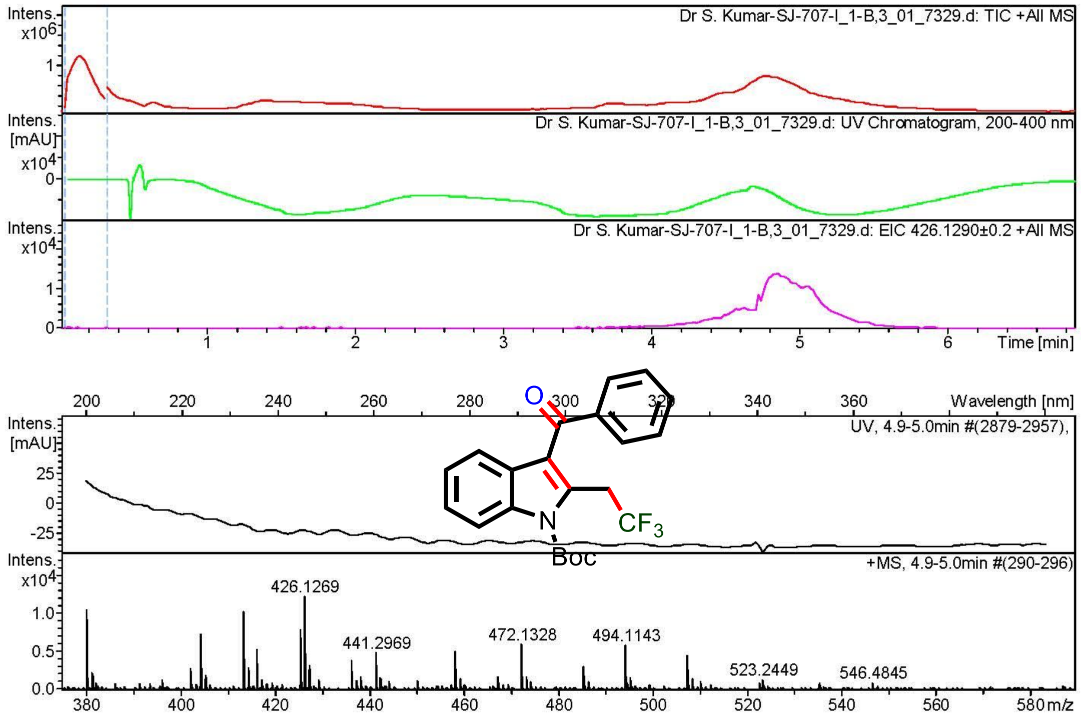

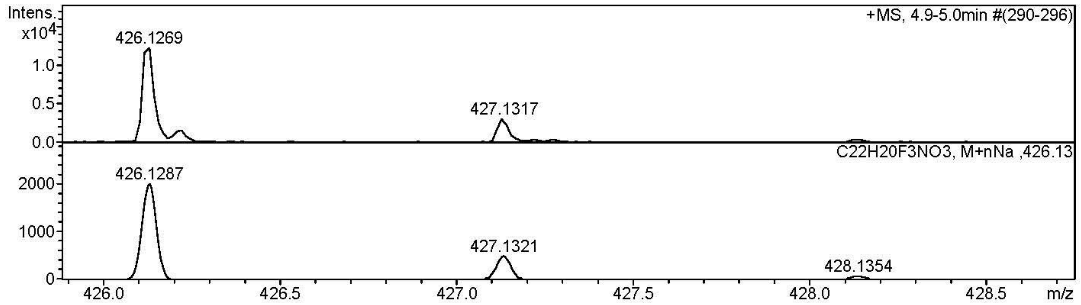

Bruker Compass DataAnalysis 4.0 printed: 9/8/2016 12:40:40 PM Page 1 of 1

^{1}H NMR of *tert*-butyl 3-(3-methoxybenzoyl)-2-(2,2,2-trifluoroethyl)-1H-indole-1-carboxylate (6i)

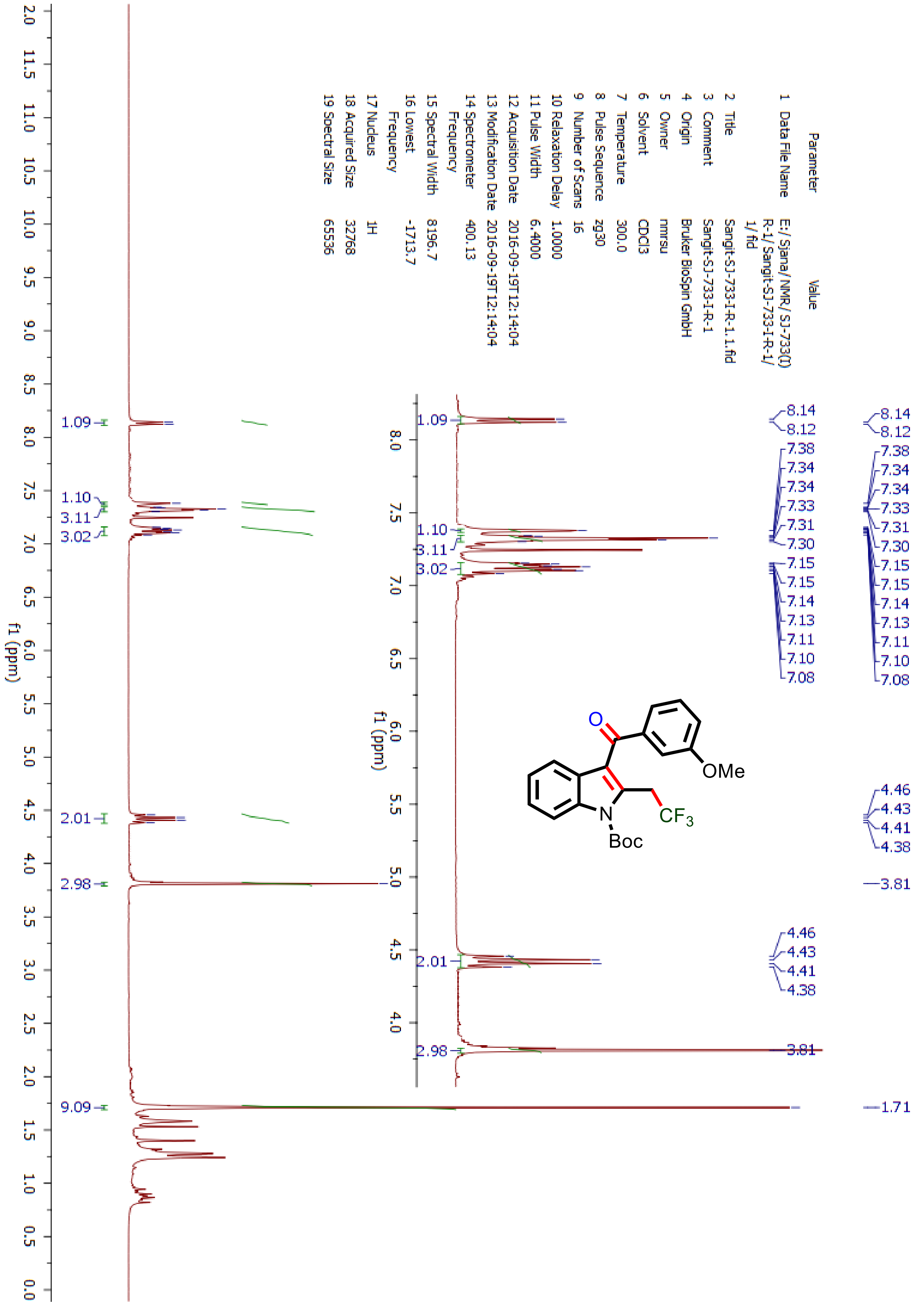

^{13}C NMR of *tert*-butyl 3-(3-methoxybenzoyl)-2-(2,2,2-trifluoroethyl)-1H-indole-1-carboxylate (6i)

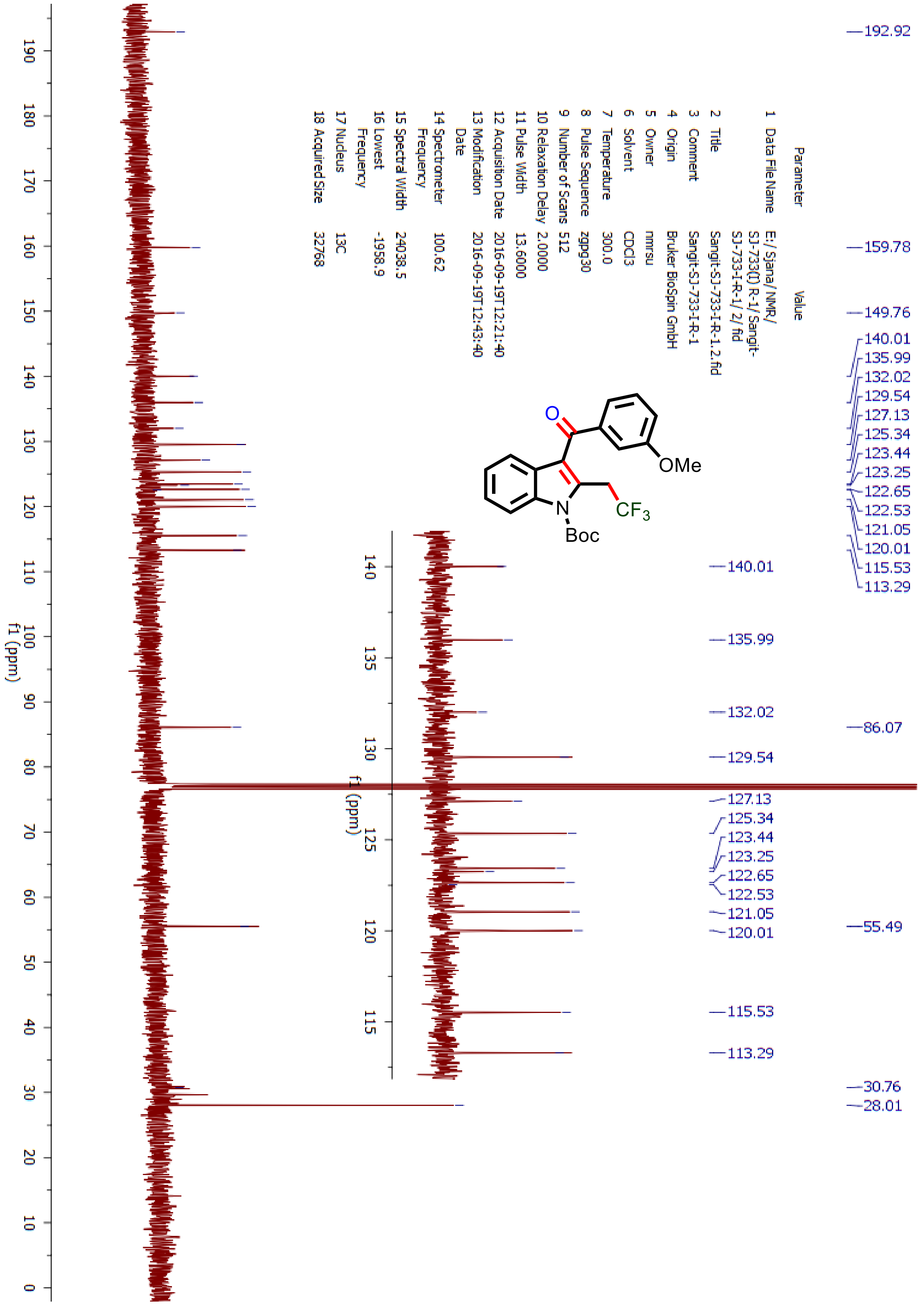

^{19}F NMR of *tert*-butyl 3-(3-methoxybenzoyl)-2-(2,2,2-trifluoroethyl)-1H-indole-1-carboxylate (6i)

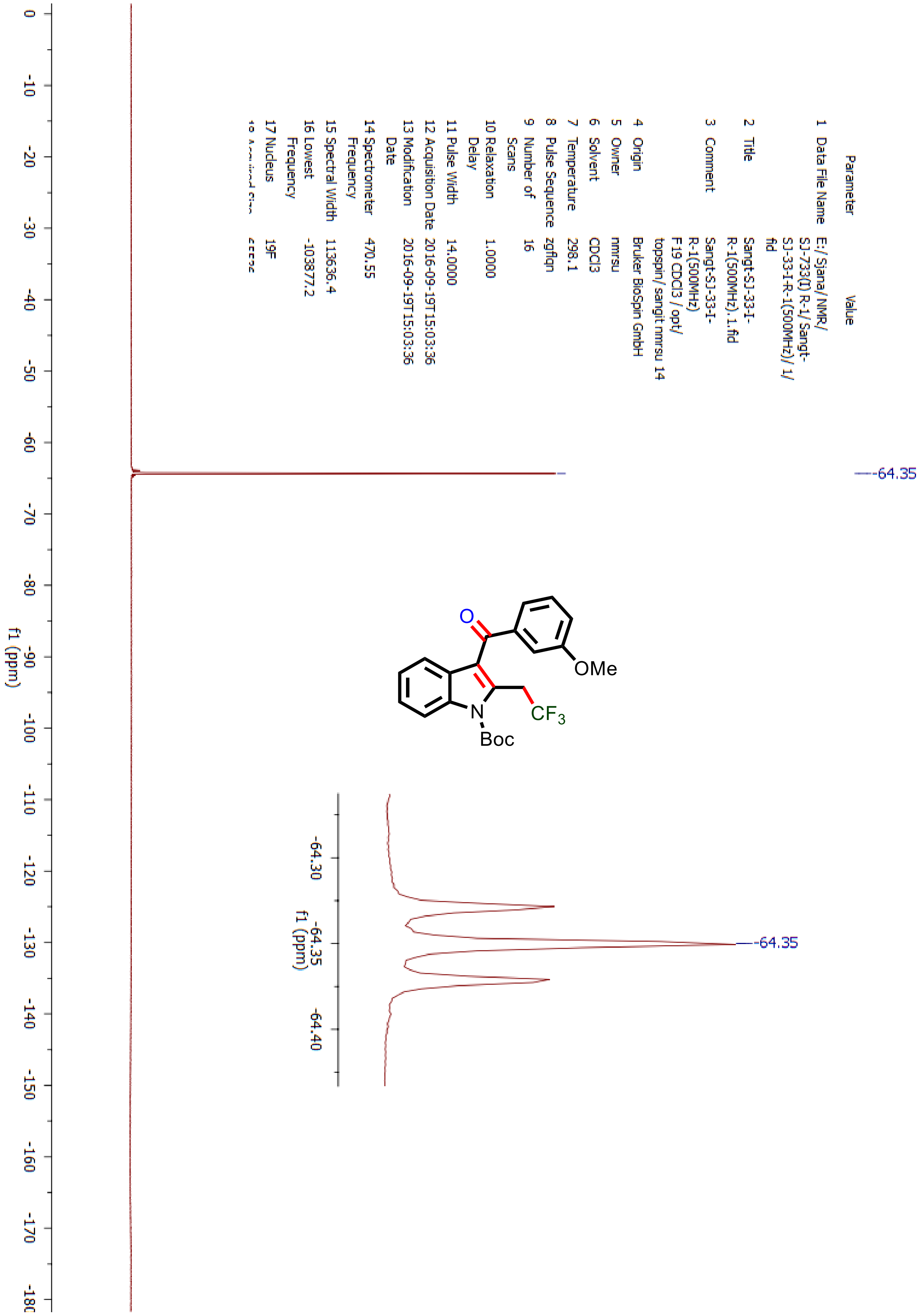

HRMS of *tert*-butyl 3-(3-methoxybenzoyl)-2-(2,2,2-trifluoroethyl)-1H-indole-1-carboxylate (6i)

Display Report

Analysis Info

Analysis Name	D:\Data\user data\2016\SEPT-2016\19-sep-2016\Dr S Kumar-SJ-733-I_1-D,8_01_7442.d	Acquisition Date	9/19/2016 1:41:09 PM
Method	hrlcms_pos_mid_tunemix.m	Operator	DIMPLE
Sample Name	Dr S Kumar-SJ-733-I	Instrument	micrOTOF-Q II 10330
Comment			

Acquisition Parameter

Source Type	ESI	Ion Polarity	Positive	Set Nebulizer	0.3 Bar
Focus	Active	Set Capillary	4500 V	Set Dry Heater	200 °C
Scan Begin	50 m/z	Set End Plate Offset	-500 V	Set Dry Gas	4.0 l/min
Scan End	3000 m/z	Set Collision Cell RF	450.0 Vpp	Set Divert Valve	Waste

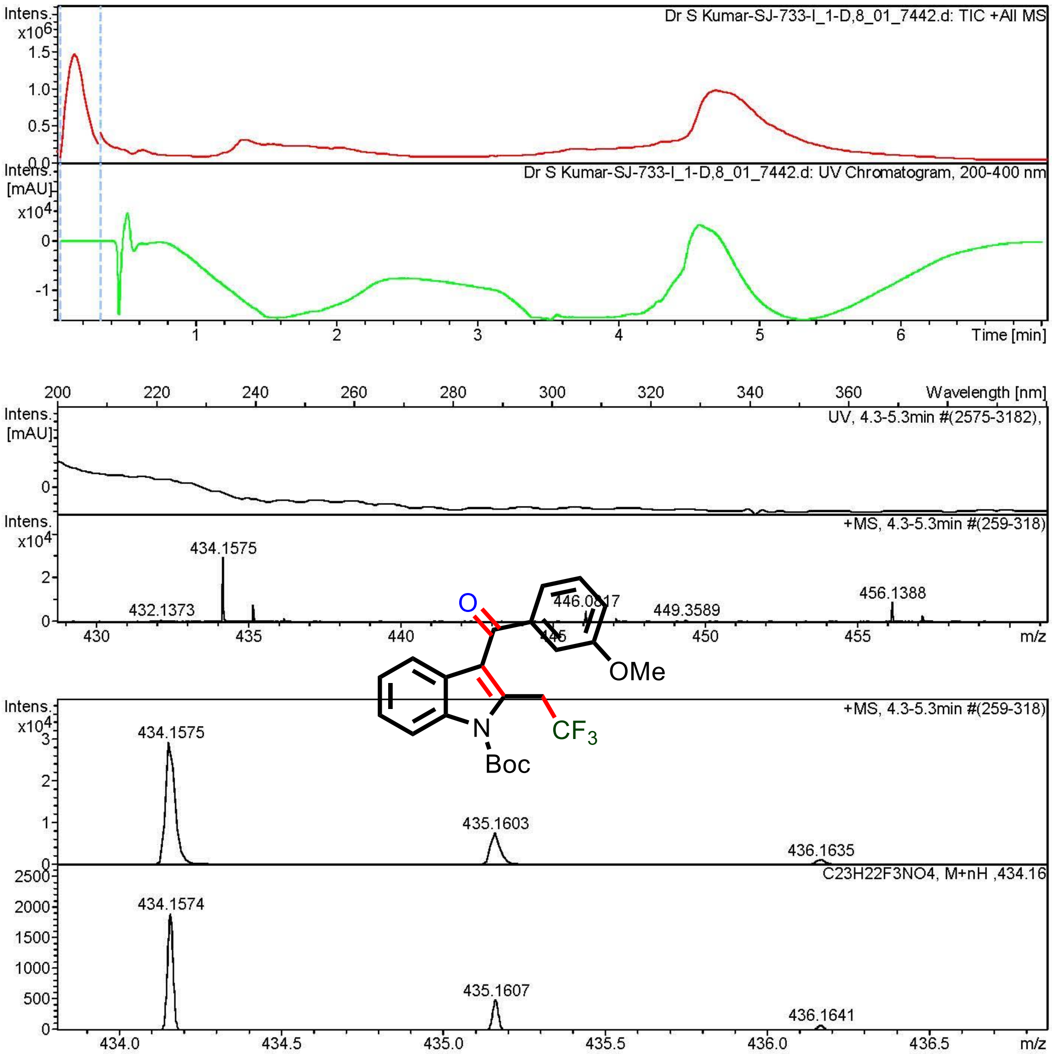

Bruker Compass DataAnalysis 4.0 printed: 9/19/2016 4:01:14 PM Page 1 of 1

^{1}H NMR of (2-Bromophenyl)(2-(2,2,2-trifluoroethyl)-1H-indol-3-yl)methanone (6j)

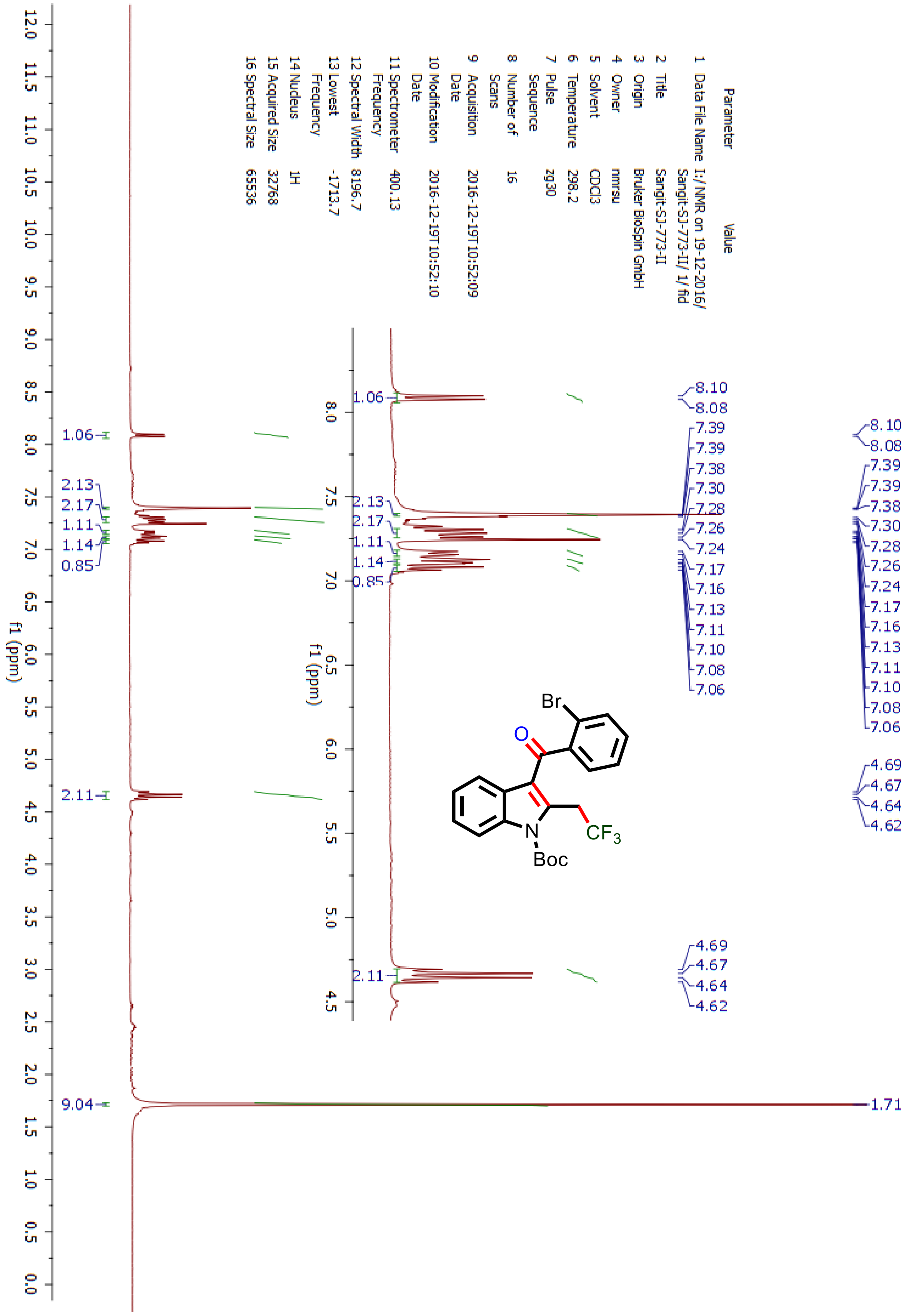

^{13}C NMR of (2-Bromophenyl)(2-(2,2,2-trifluoroethyl)-1H-indol-3-yl)methanone (6j)

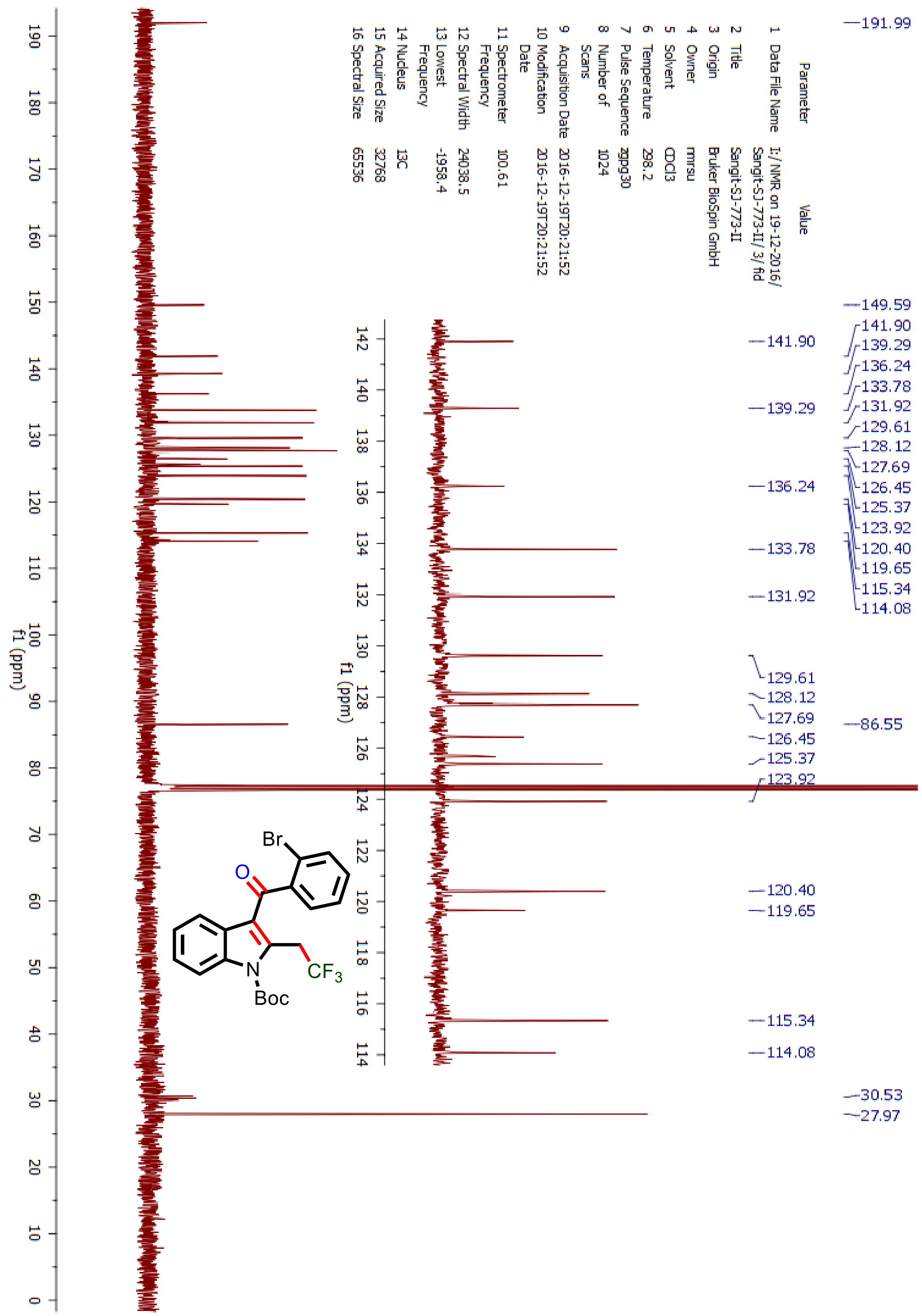

^{19}F NMR of (2-Bromophenyl)(2-(2,2,2-trifluoroethyl)-1H-indol-3-yl)methanone (6j)

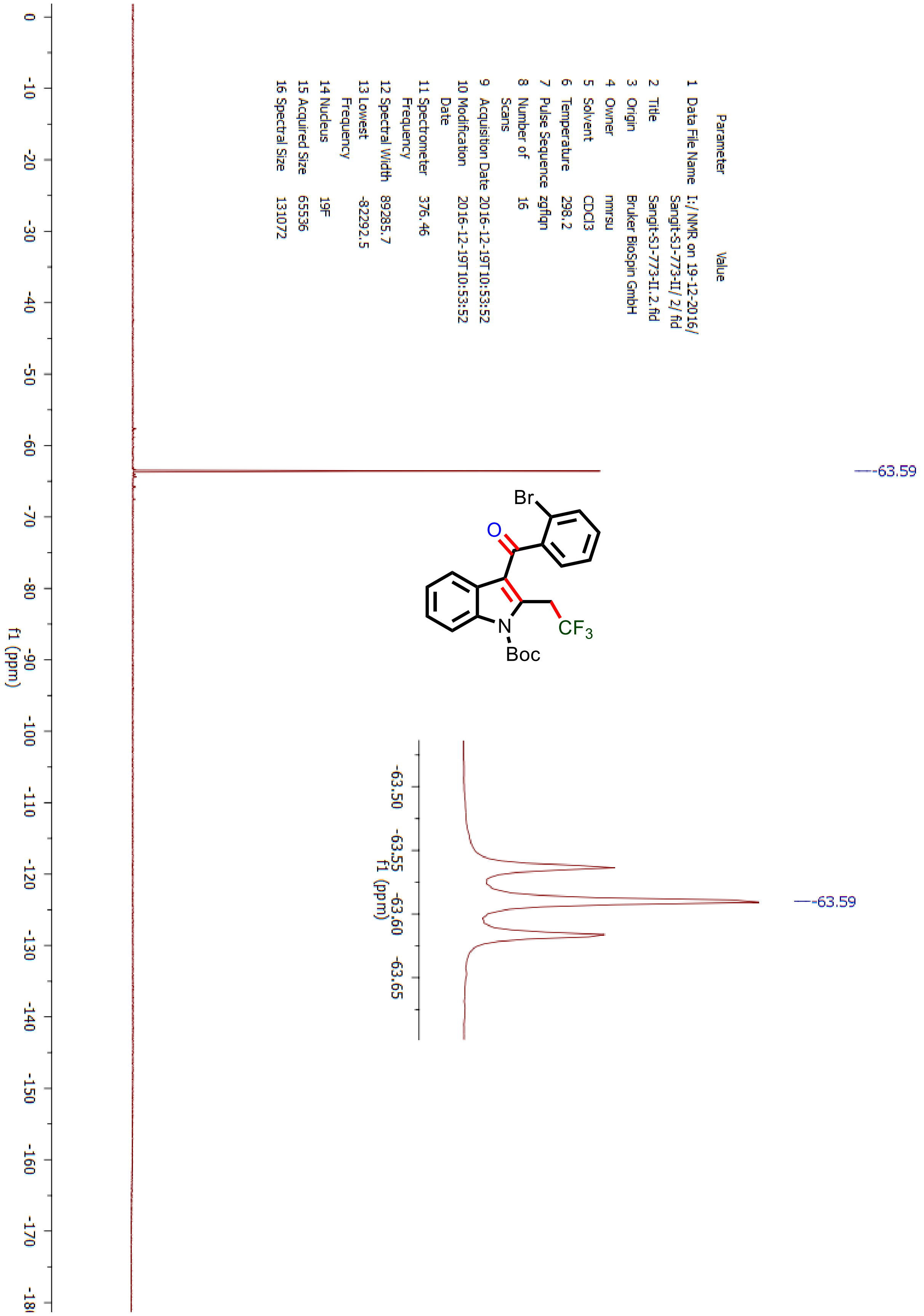

HRMS of (2-Bromophenyl)(2-(2,2,2-trifluoroethyl)-1H-indol-3-yl)methanone (6j)

Display Report

Analysis Info

		Acquisition Date	1/2/2017 12:39:41 PM
Analysis Name	D:\Data\user data\2017\JAN 2017\02 JAN\Dr S Kumar-SJ-773-II_1-D,1_01_369.d		
Method	HRLCMS-20 Sept.m	Operator	RUCHI SHRIVASTAVA
Sample Name	Dr S Kumar-SJ-773-II	Instrument	micrOTOF-Q II 10330
Comment			

Acquisition Parameter

Source Type	ESI	Ion Polarity	Positive	Set Nebulizer	1.2 Bar
Focus	Active	Set Capillary	4500 V	Set Dry Heater	200 °C
Scan Begin	50 m/z	Set End Plate Offset	-500 V	Set Dry Gas	7.0 l/min
Scan End	3000 m/z	Set Collision Cell RF	130.0 Vpp	Set Divert Valve	Waste

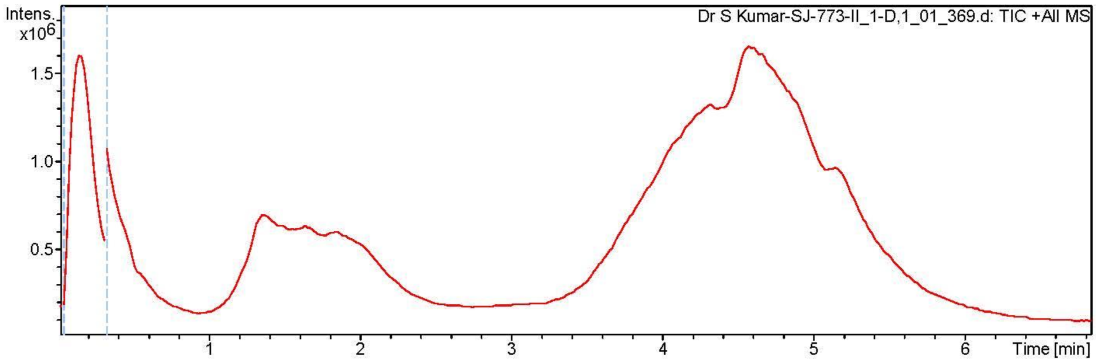

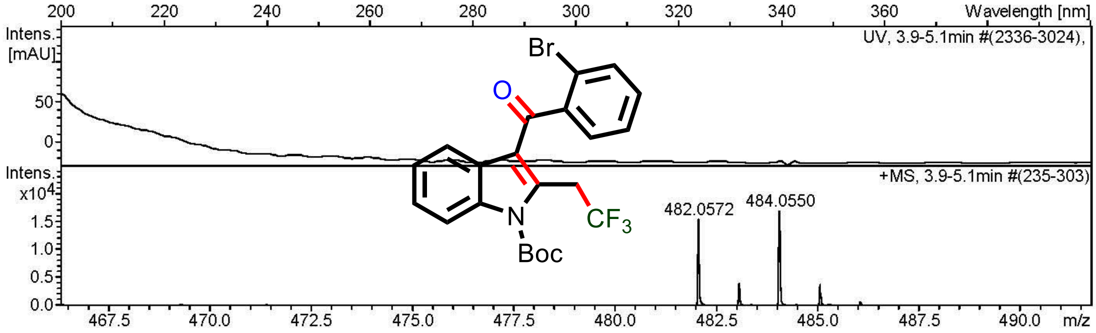

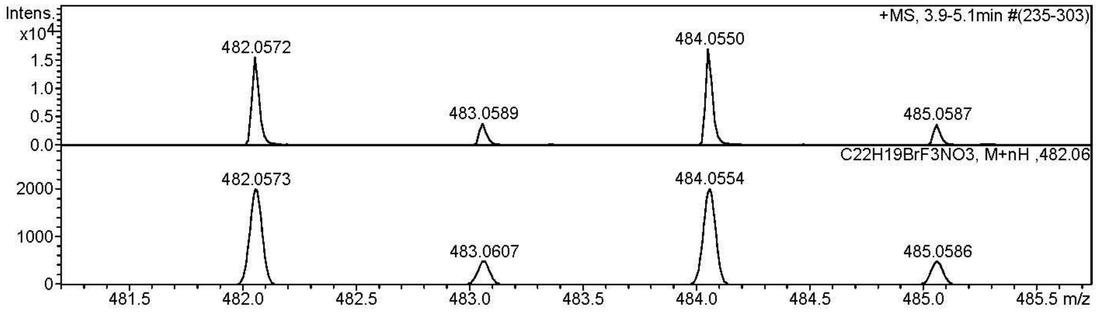

Bruker Compass DataAnalysis 4.0 printed: 1/2/2017 1:42:48 PM Page 1 of 1

^{19}F NMR of *tert*-butyl 3-(3,5-difluorobenzoyl)-2-(2,2,2-trifluoroethyl)-1H-indole-1-carboxylate (6k)

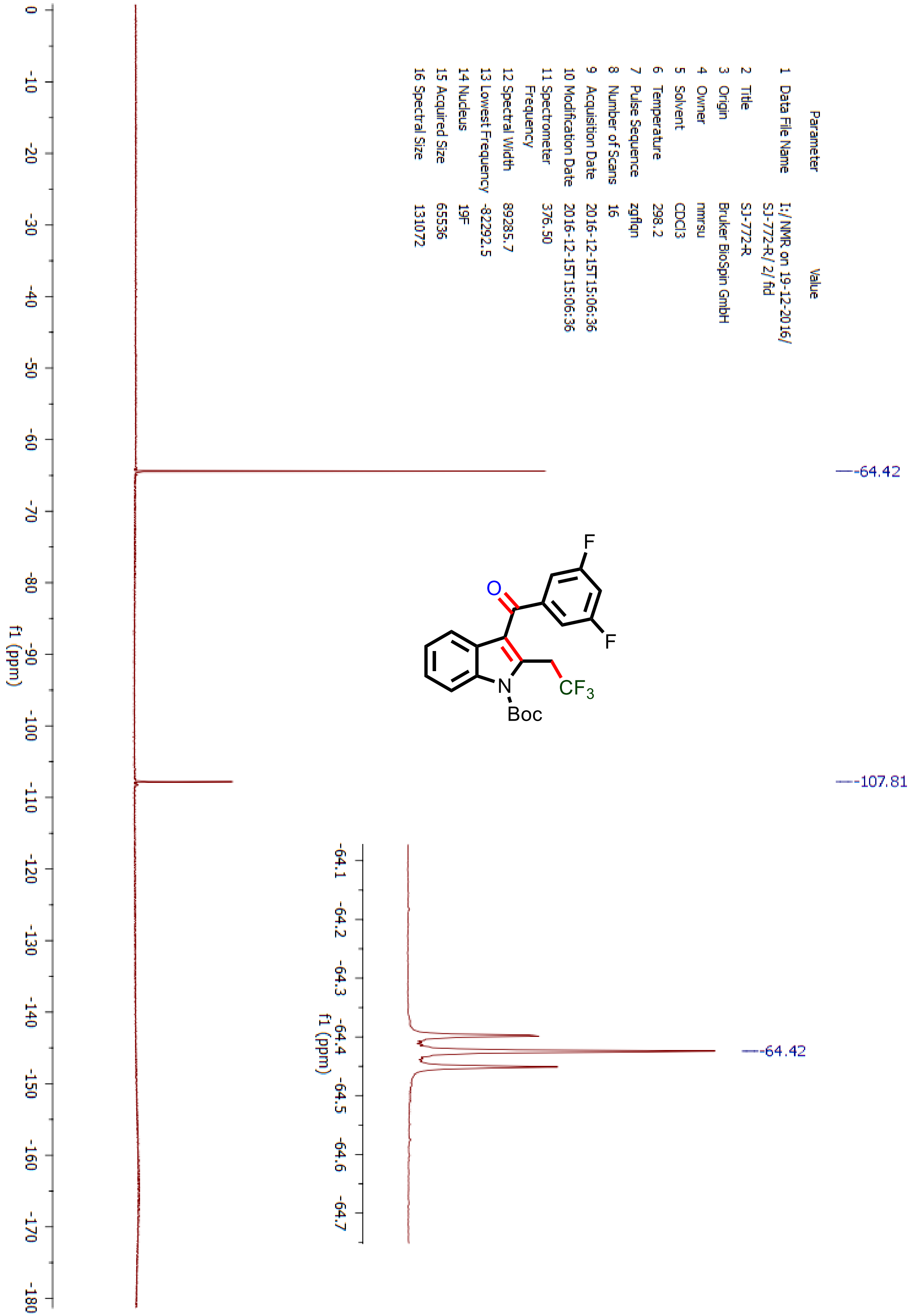

HRMS of *tert*-butyl 3-(3,5-difluorobenzoyl)-2-(2,2,2-trifluoroethyl)-1H-indole-1-carboxylate (6k)

Display Report

Analysis Info

Analysis Name	D:\Data\user data\2017\JAN 2017\02 JAN\Dr S Kumar-SJ-772-R_1-D,2_01_370.d	Acquisition Date	1/2/2017 12:47:50 PM
Method	HRLCMS-20 Sept.m	Operator	RUCHI SHRIVASTAVA
Sample Name	Dr S Kumar-SJ-772-R	Instrument	micrOTOF-Q II 10330
Comment			

Acquisition Parameter

Source Type	ESI	Ion Polarity	Positive	Set Nebulizer	1.2 Bar
Focus	Active	Set Capillary	4500 V	Set Dry Heater	200 °C
Scan Begin	50 m/z	Set End Plate Offset	-500 V	Set Dry Gas	7.0 l/min
Scan End	3000 m/z	Set Collision Cell RF	130.0 Vpp	Set Divert Valve	Waste

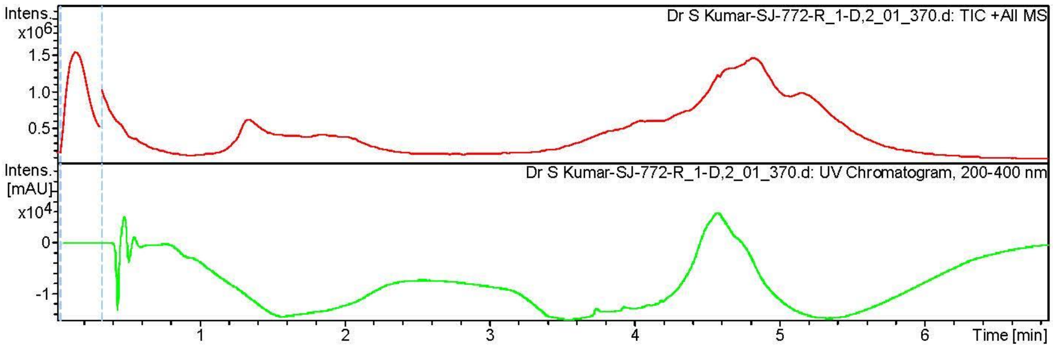

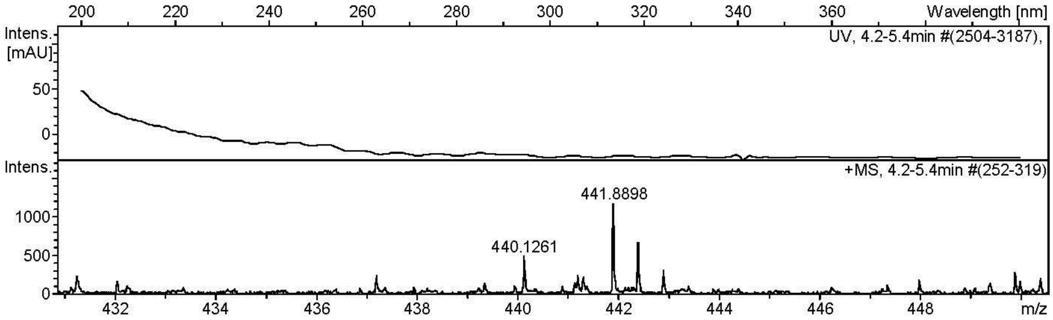

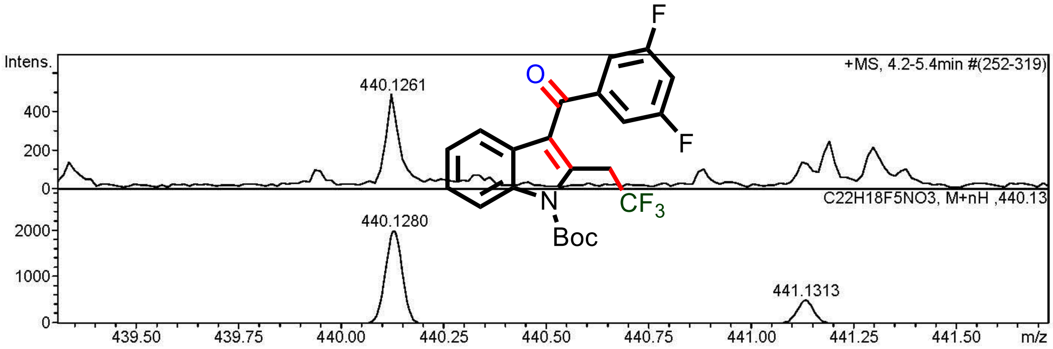

^{19}F NMR of Pyridin-3-yl(2-(2,2,2-trifluoroethyl)-1H-indol-3-yl)methanone (6l)

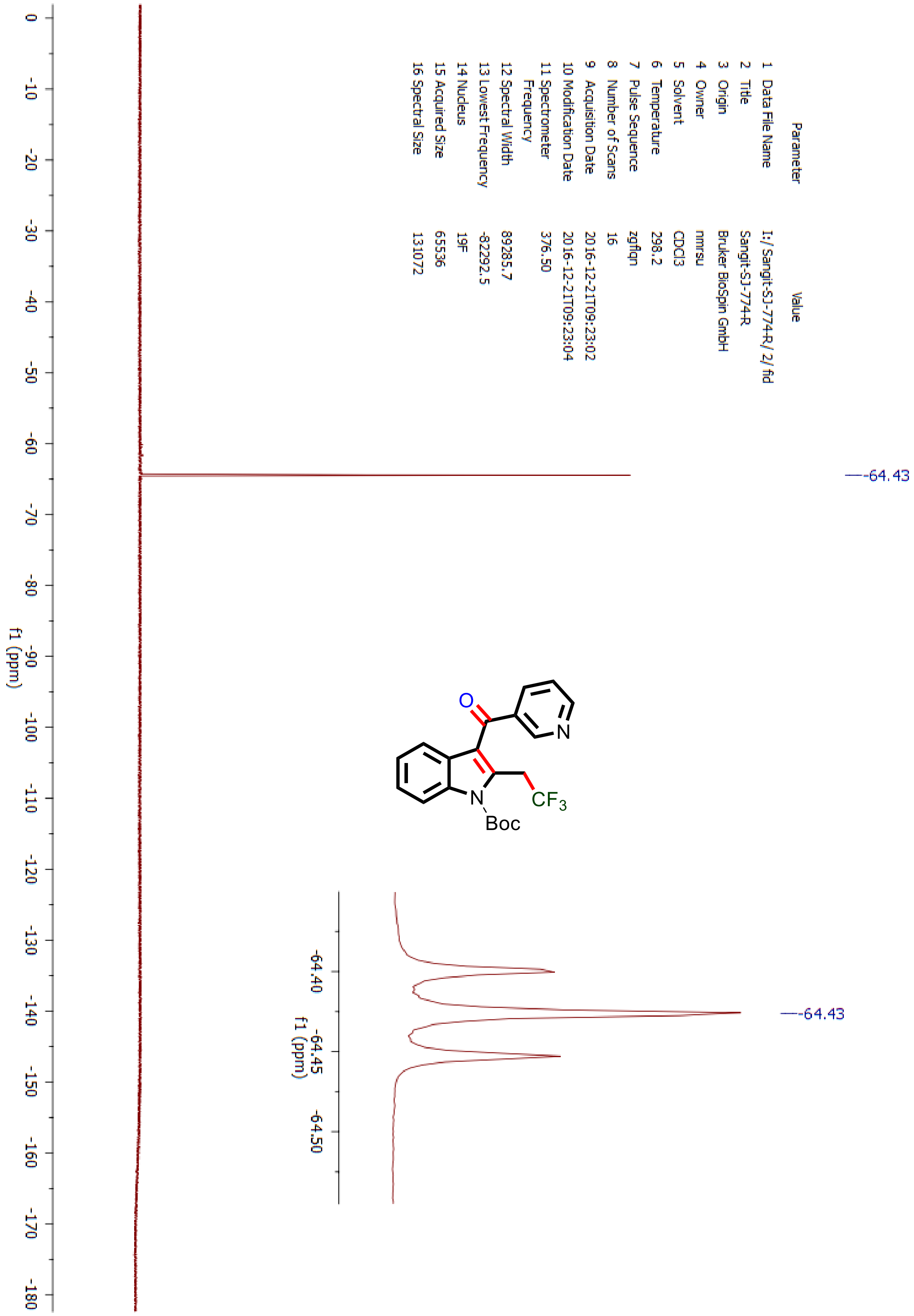

HRMS of Pyridin-3-yl(2-(2,2,2-trifluoroethyl)-1H-indol-3-yl)methanone (6l)

Display Report

Analysis Info

		Acquisition Date	1/2/2017 1:12:21 PM
Analysis Name	D:\Data\user data\2017\JAN 2017\02 JAN\Dr S Kumar-SJ-774-R_1-D,3_01_373.d		
Method	HRLCMS-20 Sept.m	Operator	RUCHI SHRIVASTAVA
Sample Name	Dr S Kumar-SJ-774-R	Instrument	micrOTOF-Q II 10330
Comment			

Acquisition Parameter

Source Type	ESI	Ion Polarity	Positive	Set Nebulizer	1.2 Bar
Focus	Active	Set Capillary	4500 V	Set Dry Heater	200 °C
Scan Begin	50 m/z	Set End Plate Offset	-500 V	Set Dry Gas	7.0 l/min
Scan End	3000 m/z	Set Collision Cell RF	130.0 Vpp	Set Divert Valve	Waste

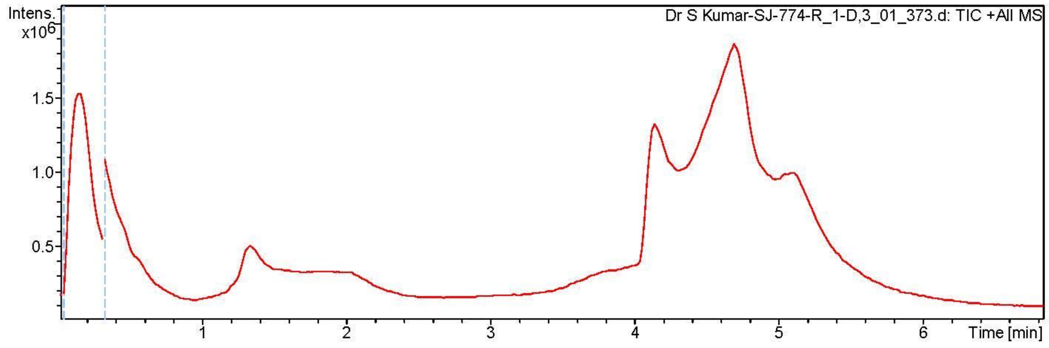

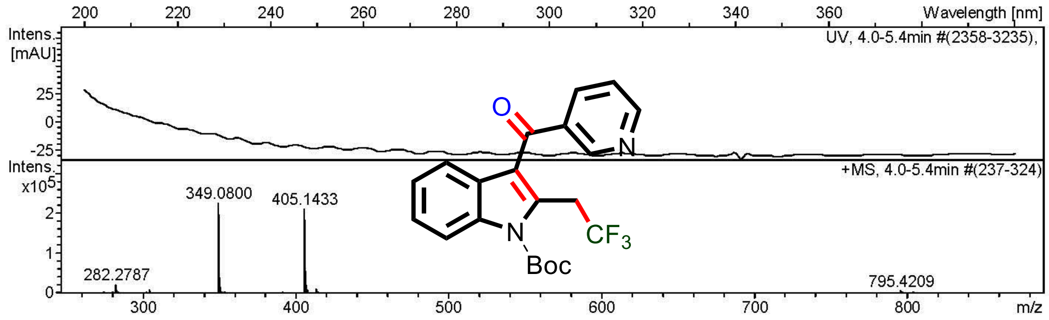

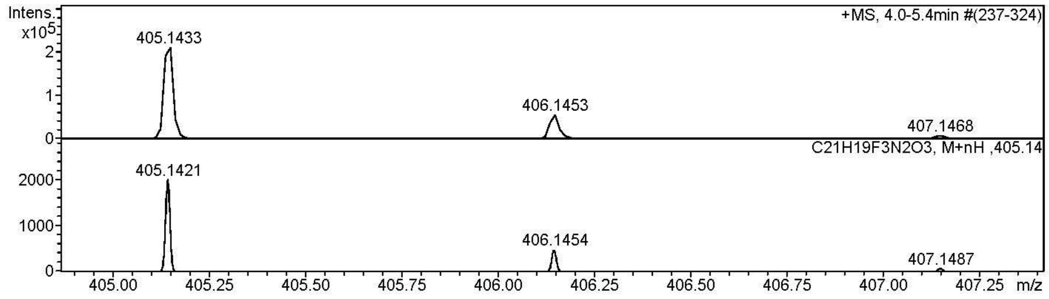

Bruker Compass DataAnalysis 4.0 printed: 1/2/2017 1:47:49 PM Page 1 of 1

^{1}H NMR of (3,5-dibromo-4-hydroxyphenyl)(2-(2,2,2-trifluoroethyl)benzofuran-3-yl)methanone (7a)

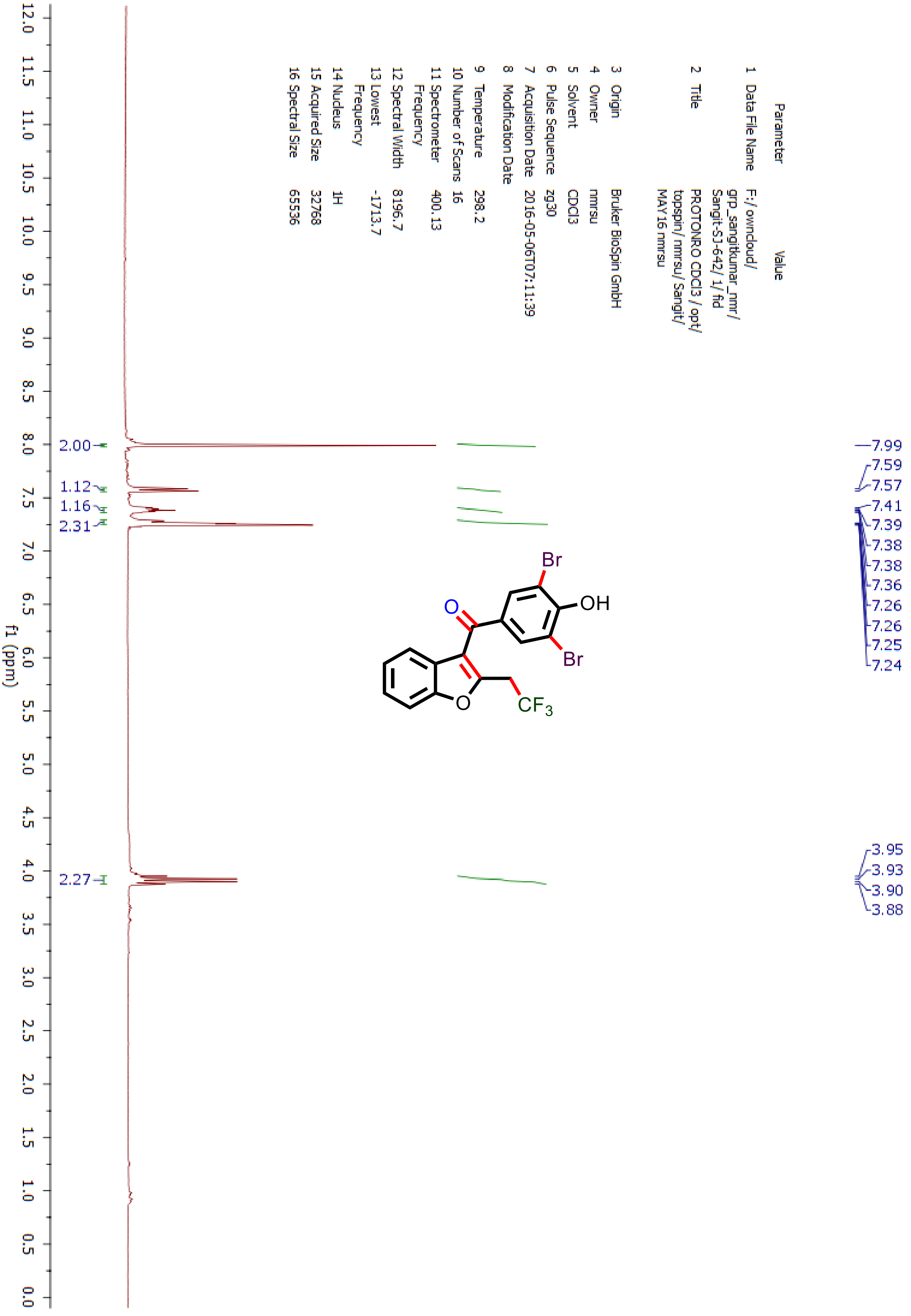

^{13}C NMR of (3,5-dibromo-4-hydroxyphenyl)(2-(2,2,2-trifluoroethyl)benzofuran-3-yl)methanone (7a)

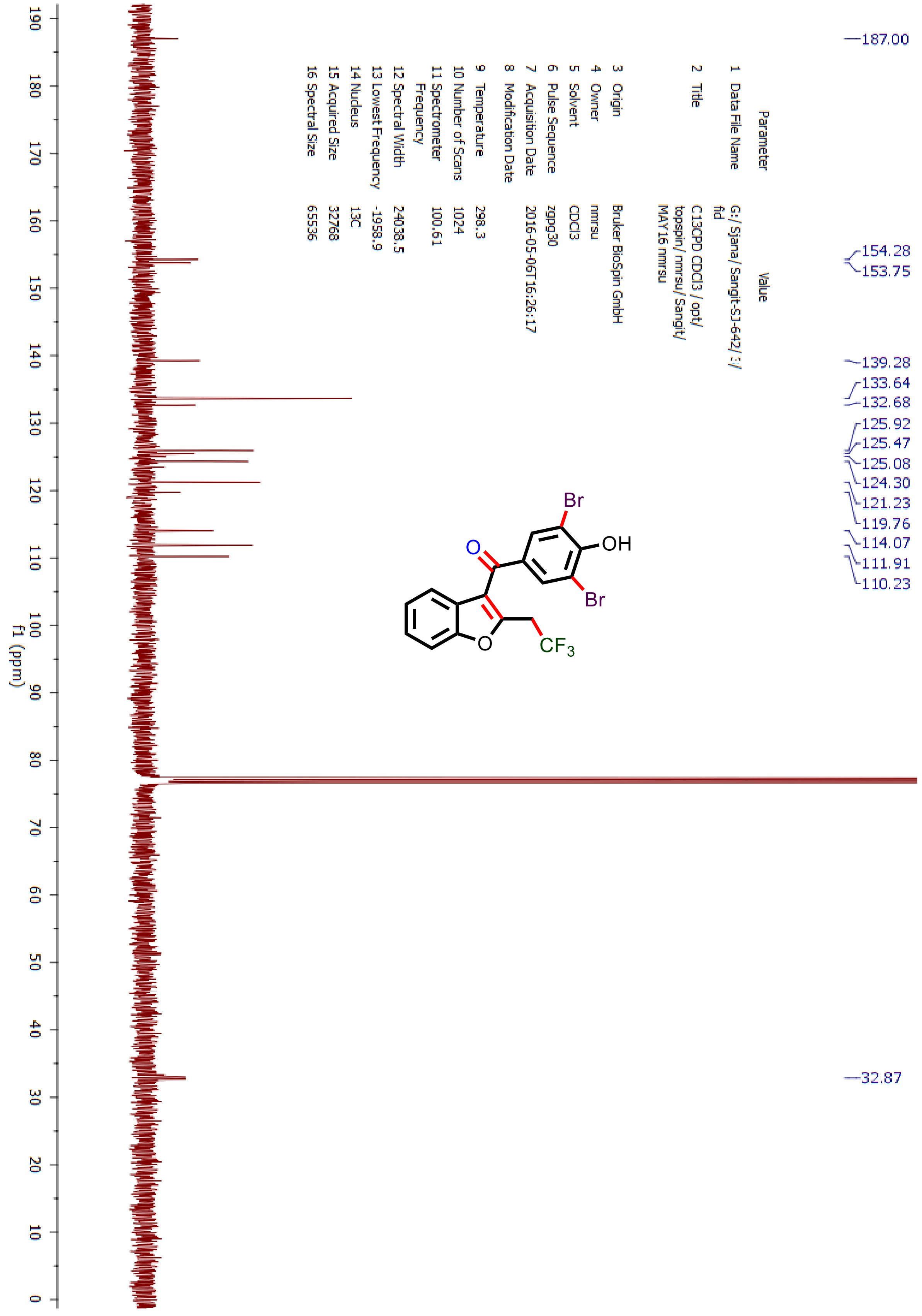

^{19}F NMR of (3,5-dibromo-4-hydroxyphenyl)(2-(2,2,2-trifluoroethyl)benzofuran-3-yl)methanone (7a)

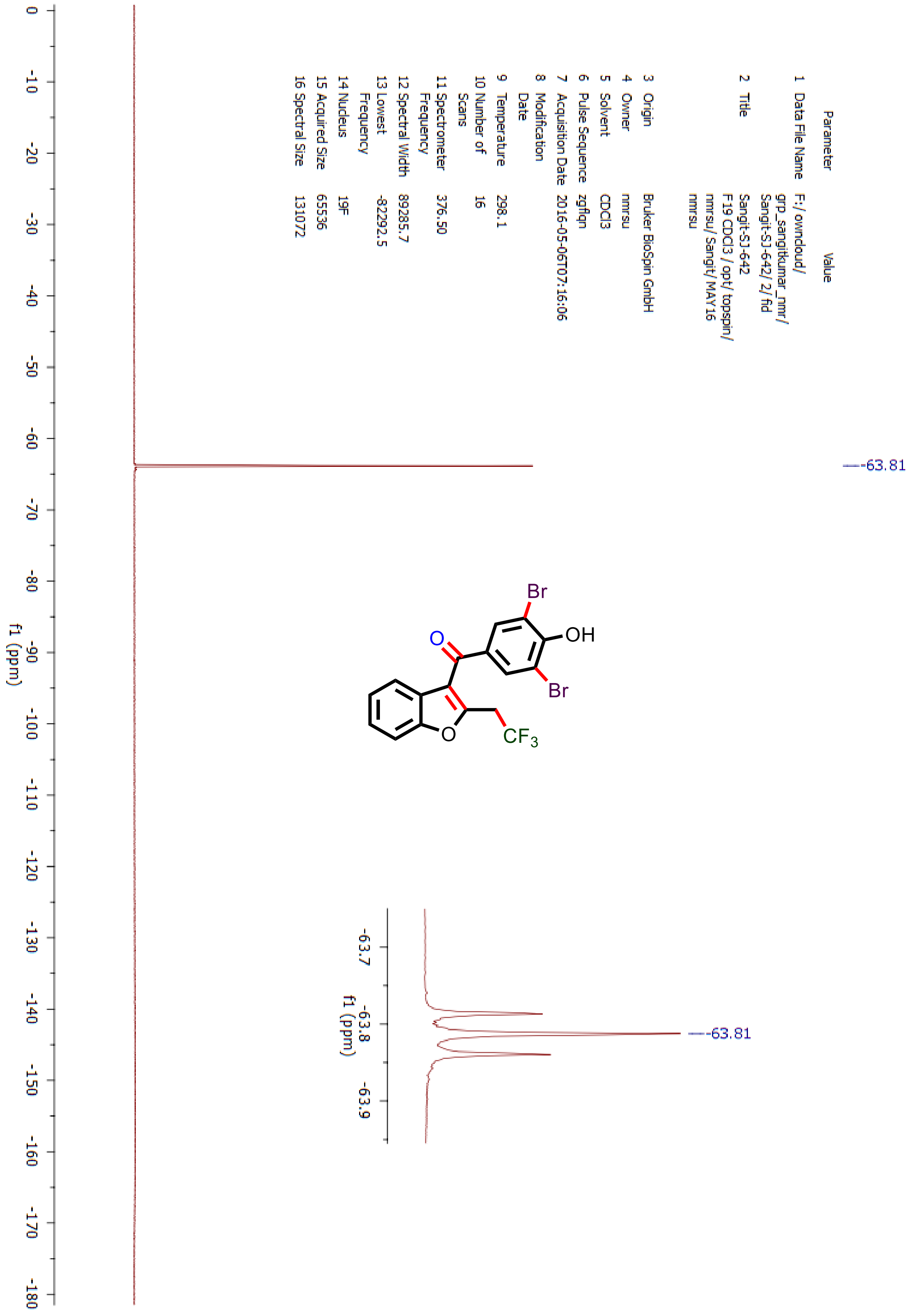

HRMS of (3,5-dibromo-4-hydroxyphenyl)(2-(2,2,2-trifluoroethyl)benzofuran-3-yl)methanone (7a)

Display Report

Analysis Info

		Acquisition Date	9/19/2016 3:04:36 PM
Analysis Name	D:\Data\user data\2016\SEPT-2016\19-sep-2016\Dr S Kumar-SJ-642_1-D,7_01_7446.d		
Method	hrlcms_pos_mid_tunemix.m	Operator	DIMPLE
Sample Name	Dr S Kumar-SJ-642	Instrument	micrOTOF-Q II 10330
Comment			

Acquisition Parameter

Source Type	ESI	Ion Polarity	Positive	Set Nebulizer	0.3 Bar
Focus	Active	Set Capillary	4500 V	Set Dry Heater	200 °C
Scan Begin	50 m/z	Set End Plate Offset	-500 V	Set Dry Gas	4.0 l/min
Scan End	3000 m/z	Set Collision Cell RF	450.0 Vpp	Set Divert Valve	Waste

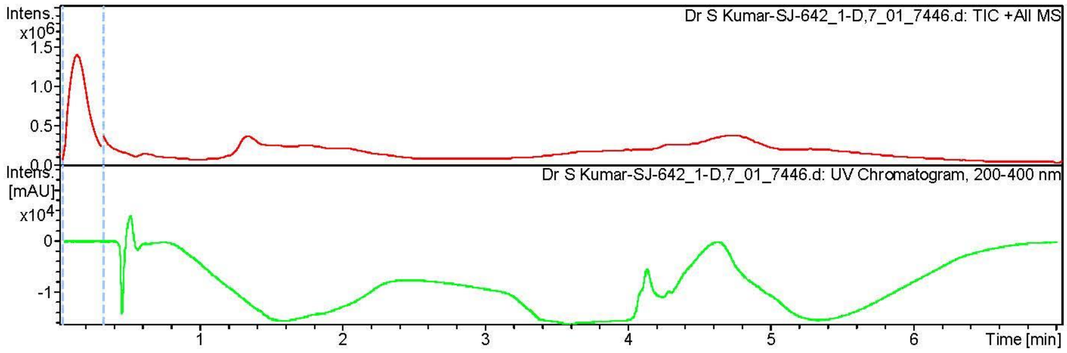

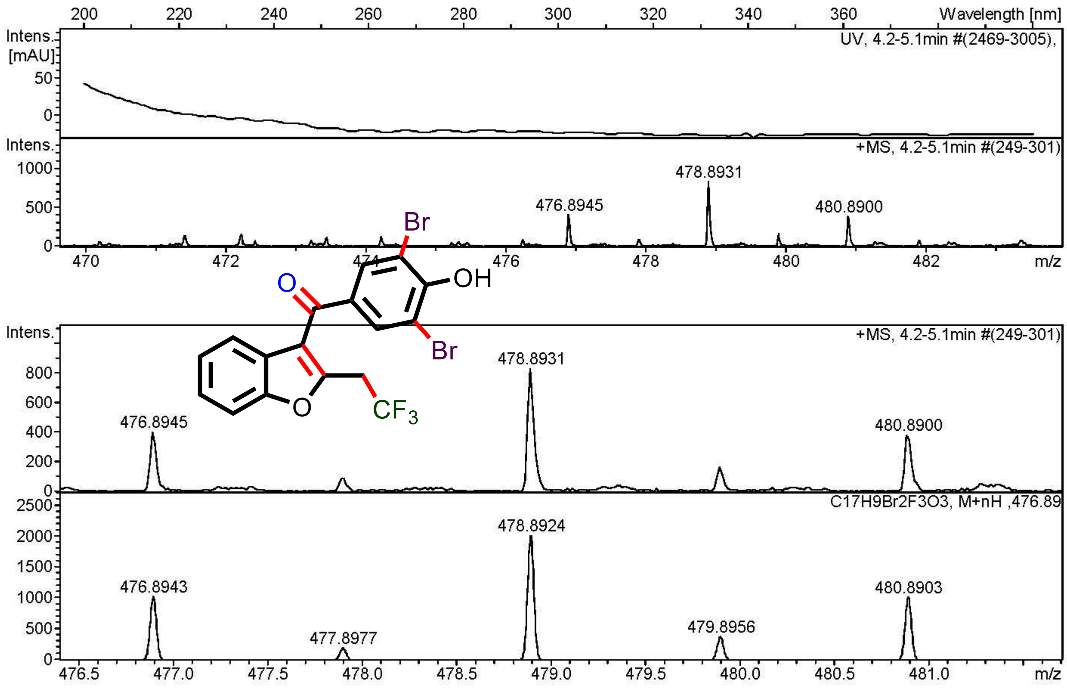

^{1}H NMR of Naphthalen-1-yl(1-propyl-2-(2,2,2-trifluoroethyl)-1H-indol-3-yl)methanone (7b)

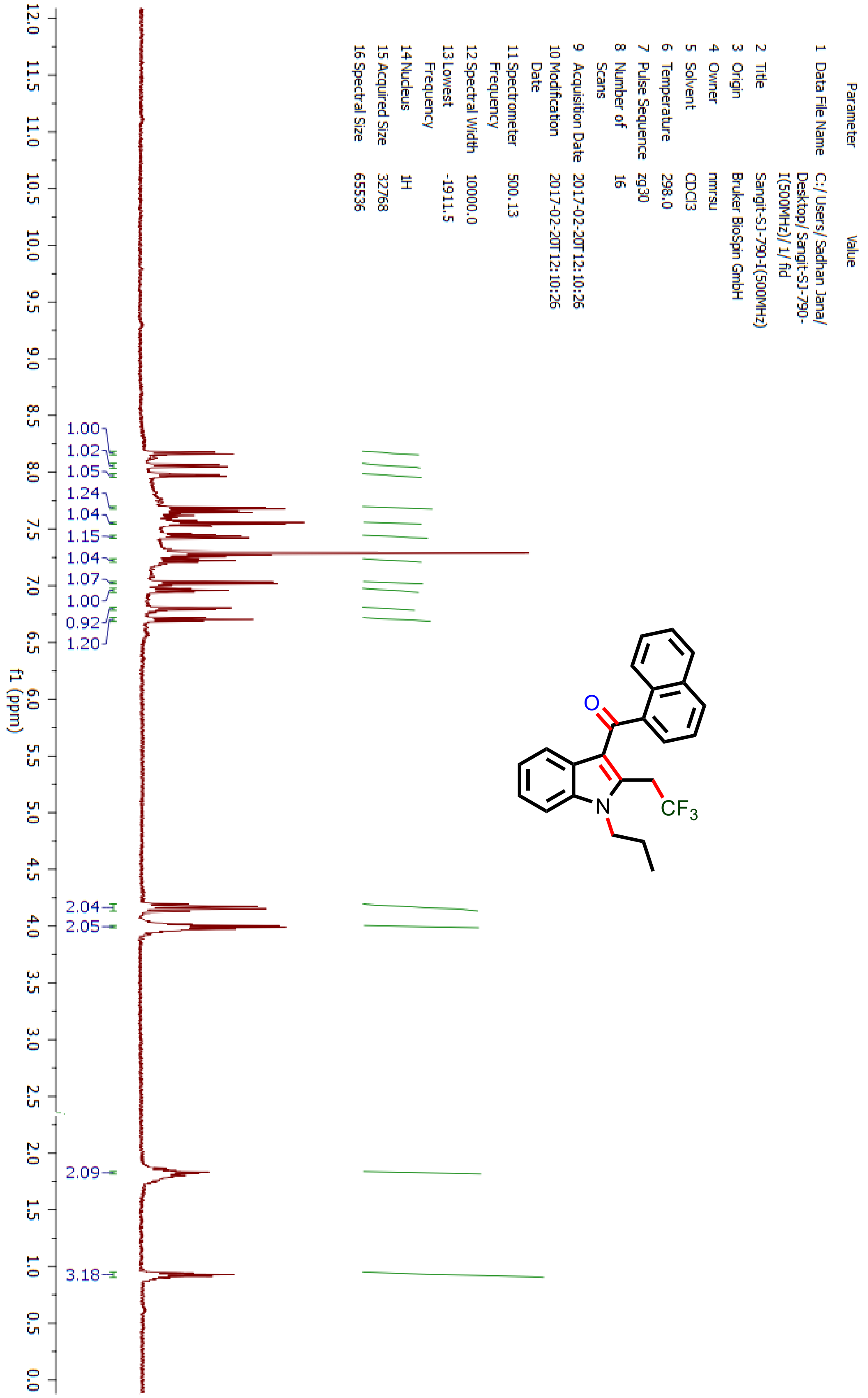

^{13}C NMR of Naphthalen-1-yl(1-propyl-2-(2,2,2-trifluoroethyl)-1H-indol-3-yl)methanone (7b)

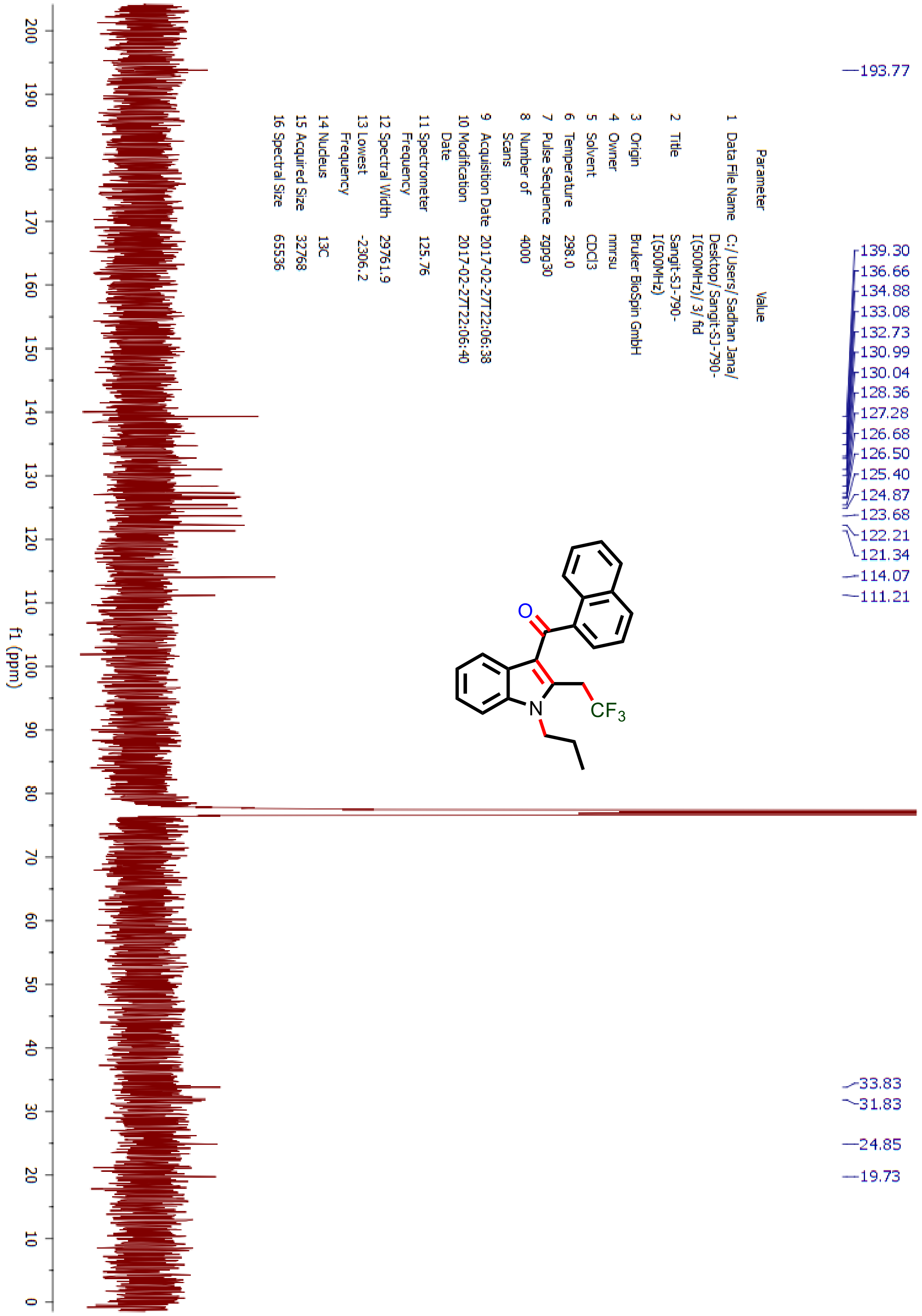

^{19}F NMR of Naphthalen-1-yl(1-propyl-2-(2,2,2-trifluoroethyl)-1H-indol-3-yl)methanone (7b)

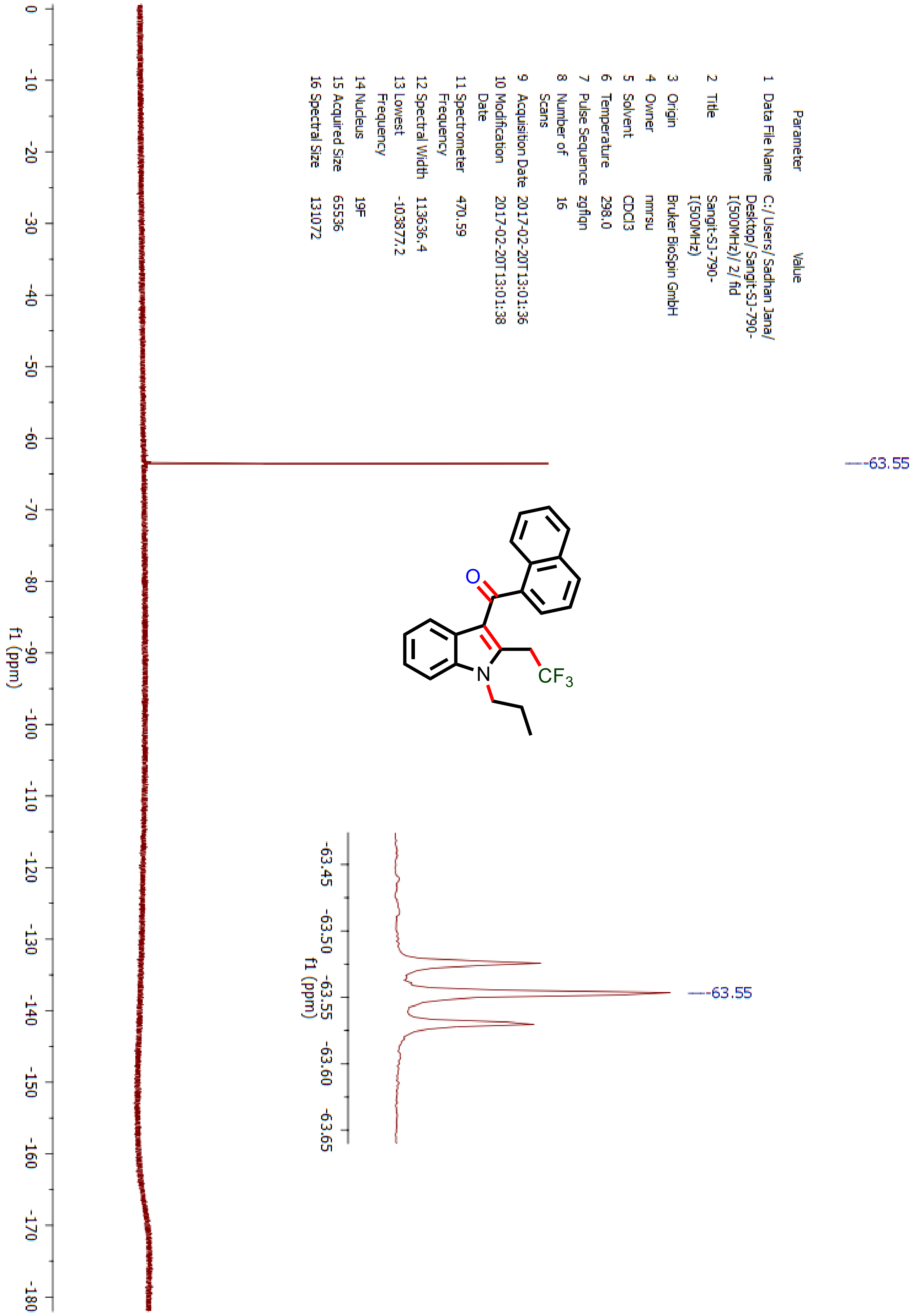

HRMS of Naphthalen-1-yl(1-propyl-2-(2,2,2-trifluoroethyl)-1H-indol-3-yl)methanone (7b)

Display Report

Analysis Info

Analysis Name	D:\Data\user data\2017\FEB 2017\22 FEB\Dr S Kumar-SJ-790-VE.d	Acquisition Date	2/22/2017 3:05:45 PM
Method	Neg_LOW_tunemix.m	Operator	RUCHI SHRIVASTAVA
Sample Name	SJ-790-VE	Instrument	micrOTOF-Q II 10330
Comment			

Acquisition Parameter

Source Type	ESI	Ion Polarity	Negative	Set Nebulizer	0.4 Bar
Focus	Active	Set Capillary	2500 V	Set Dry Heater	200 °C
Scan Begin	50 m/z	Set End Plate Offset	-500 V	Set Dry Gas	4.0 l/min
Scan End	3000 m/z	Set Collision Cell RF	130.0 Vpp	Set Divert Valve	Waste

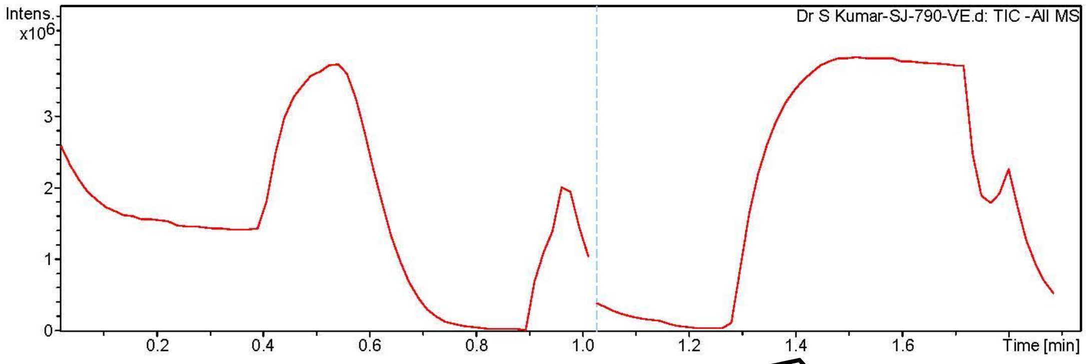

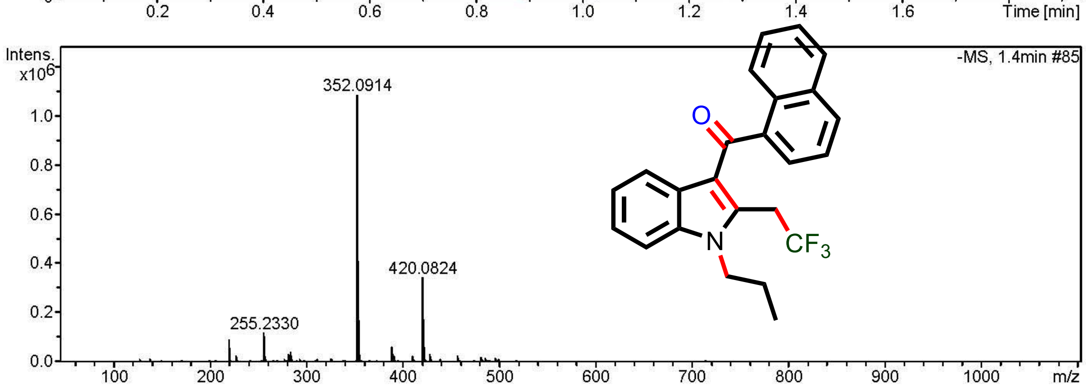

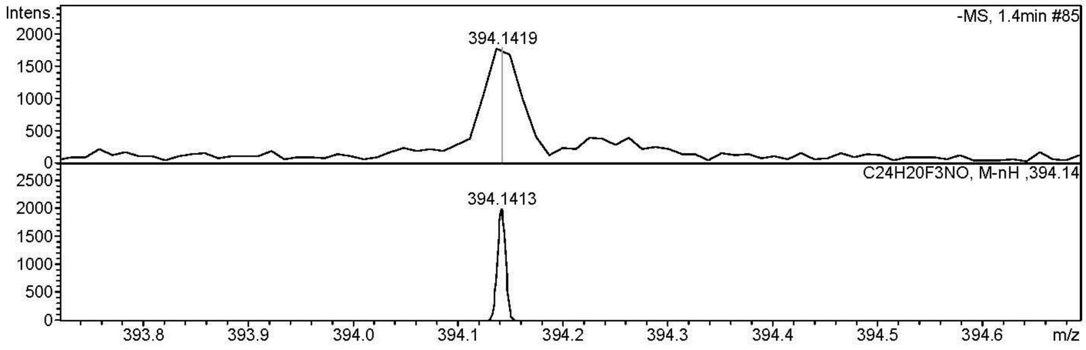

Supplement: Supplementary file 2 [file SC-008-C7SC02556D-s002.pdf]
